# Supplementary material for: No prediction without prevention: A global qualitative study of attitudes toward using a prediction tool for risk of developing depression during adolescence
Source: Glob Ment Health (Camb). 2025 Jan 3;11:e129. doi: 10.1017/gmh.2024.136 (PMC11704374; doi:10.1017/gmh.2024.136)
Supplement: Kohrt et al. supplementary material [file S2054425124001365sup001.zip › Supplemental File 1_COREQ Checklist_IDEA.pdf]

Consolidated criteria for reporting qualitative studies (COREQ): 32-item checklist

Developed from:
Tong A, Sainsbury P, Craig J. Consolidated criteria for reporting qualitative research (COREQ): a 32-item checklist for interviews and focus groups. *International Journal for Quality in Health Care*. 2007. Volume 19, Number 6: pp. 349 – 357

Full details on the IDEA qualitative study methodology are available in the published protocol: Wahid, S. S., Pedersen, G. A., Ottman, K., Burgess, A., Gautam, K., Martini, T., Viduani, A., Momodu, O., Lam, C., Fisher, H. L., Kieling, C., Adewuya, A. O., Mondelli, V., & Kohrt, B. A. (2020). Detection of risk for depression among adolescents in diverse global settings: protocol for the IDEA qualitative study in Brazil, Nepal, Nigeria and the UK. *BMJ Open*, 10(7), e034335. https://doi.org/10.1136/bmjopen-2019-034335

**No. Item**	**Criteria description**	**Study Information**
**Domain 1: Research team and reflexivity**		
Personal Characteristics		
1. Interviewer/ facilitator<br>2. Credentials<br>3. Occupation<br>4. Gender<br>5. Experience and training	Which author/s conducted the interview or focus group?<br>What were the researcher's credentials? E.g. PhD, MD<br>What was their occupation at the time of the study?<br>Was the researcher male or female?<br>What experience or training did the researcher have?	Brazil: In Brazil, the qualitative study was implemented by a team from the Universidade Federal do Rio Grande do Sul. Data collection efforts was led led by five researchers from IDEA Brazil team: one psychologist, two psychology undergraduate students, and one with social communication training (all females); and two medical doctors (both males), which includes an IDEA principal investigator.<br>Nepal: The data collection efforts in Nepal was implemented by the Transcultural Psychosocial Organization Nepal (TPO Nepal), one of Nepal's leading psychosocial organizations for providing clinical care and conducting mental health research. TPO interviewers and FGD facilitators included two female researchers with graduate level training in Public Health and one male researcher with graduate training in management studies, under the guidance of a senior TPO psychiatrist and researcher.<br>Nigeria: In Nigeria, the study was managed by the Lagos State University, College of Medicine, Department of Behavioral Medicine. Data collection efforts were led by a team of two female doctors, a consultant psychiatrist, and a medical officer under guidance of a senior professor and psychiatrist, who is an IDEA project co-investigator.<br>United Kingdom: In the UK, the data collection efforts were implemented by King's College London. The primary data collector was a female social scientist with

No. Item	Criteria description	Study Information
		graduate training in nursing and psychology, with experience in conducting qualitative research. The UK activities are guided by a co-investigator of the IDEA project, who has a doctorate in Psychology.
Relationship with participants		
6. Relationship established<br>7. Participant knowledge of the interviewer<br>8. Interviewer characteristics	Was a relationship established prior to study commencement?<br>What did the participants know about the researcher? e.g., personal goals, reasons for doing the research<br>What characteristics were reported about the interviewer/facilitator? e.g., Bias, assumptions, reasons and interests in the research topic	Brazil: There was no relationship between interviewers and participants prior to the study. The exception was policymakers, most of whom were known to the PI of the Brazil team. However, the interviewers themselves did not have direct prior interaction with the policymakers. All interviewers were female, so there was no gender matching with participants.<br>Nepal: Some adolescents with lived experience of depression and parents of adolescents with lived experience were recruited through TPO-Nepal's advisory group. For other participants, there were no direct relationships between interviewers and participants. The PI in Nepal knew the policymakers, but the PI did not conduct any interviews. All interviewers were female, so there was no gender matching with participants.<br>Nigeria: There were no relationships between participants and interviewers. The PI in Nigeria knew the policymakers, but he did not personally conduct the interviews. The interviewers were all women, so there was no gender-matching for male respondents.<br>United Kingdom: There was one interviewer who had no prior relationship with participants.
**Domain 2: study design**		
Theoretical framework		
9. Methodological orientation and Theory	What methodological orientation was stated to underpin the study? e.g. grounded theory, discourse analysis, ethnography, phenomenology, content analysis	Excerpt from IDEA qualitative protocol paper (https://doi.org/10.1136/bmjopen-2019-034335): *The IDEA qualitative study is structured according to the social ecological model of health and Singer and Baer's world system theory on the social origins of disease. Using these two guiding theoretical frameworks, we seek to understand the role of individual, interpersonal, institutional, community and policy factors and their interrelations, in depression risk and identification in adolescence. Informed by George Engels' classic model, we will elicit biopsychosocial risk and protective factors of depression within and between each ecological stratum. We will utilize Kleinman's Explanatory Model framework to explore the lived experience of depression at the individual level, including culturally driven local idioms of distress. We will further explore how these*

No. Item	Criteria description	Study Information
		explanatory models are influenced by relationships at the interpersonal and primary group levels (family and friends), and cultural and social norms at the community level. At the institutional level, we will examine mental health services for depression identification and management, and acceptability and feasibility of risk detection at schools, primary health care, and social services. At the policy level, we will seek to understand challenges and opportunities to facilitate better depression detection and management. Additionally, in the LMIC sites, we will explore institutional capacity for conducting biological psychiatry research (i.e. biological specimen collection, storage, and testing capacity; and research capacity of universities and staff) and policy level considerations for ethical research governance that can support sensitive biological psychiatry research.
Participant selection		
10. Sampling<br>11. Method of approach	How were participants selected? e.g. purposive, convenience, consecutive, snowball<br>How were participants approached? e.g. face-to-face, telephone, mail, email	Brazil: The recruitment process will utilize connections of the IDEA Brazil team to local, relevant, institutions. The key informant interviews (KII) took place in private and convenient locations for each participant, and the focus group discussions (FGD) was held in Hospital de Clínicas de Porto Alegre.<br>Nepal: Recruitment for the study was conducted by reaching out to institutions offering adolescent mental health services in Kathmandu district in Nepal. KIIs were conducted in private locations at health facilities, schools, stakeholder offices, TPO Nepal offices, and Ministry offices. The FGDs were conducted at TPO offices. KIIs and FGDs were conducted in Nepali & English.<br>Nigeria: Recruitment occurred through flyers distributed to social workers, health care providers and the teachers in both public and private schools in Lagos. Letters were written to the permanent secretaries of the ministries of health, education, social services and the health service commission requesting the nomination of civil servants for policy-maker interviews of the respective ministries. Interviews were done at the convenience of the participant, and the timing and the location was flexible. Interviews were held at the office of the researchers or at the offices of the person being interviewed. The FGDs were held at Department of Behavioural Medicine at The Lagos State University Teaching Hospital and at the Child and Adolescent Mental Health Service of The Federal Neuropsychiatry Hospital Oshodi Annex.<br>United Kingdom: Participants were recruited using an opportunistic sampling method, with stakeholder specific adverts being circulated via professional networks, social media, and placed in key locations in the community, including

No. Item	Criteria description	Study Information
		local NHS hospitals. The KIIs occurred at King's College London campuses, or at stakeholder's office spaces where appropriate, and over the telephone.
12. Sample size	How many participants were in the study?	**See Table 1 in the manuscript**
13. Non-participation	How many people refused to participate or dropped out? Reasons?	Brazil: None<br>Nepal: None<br>Nigeria: None<br>United Kingdom: None
Setting		
14. Setting of data collection	Where was the data collected? e.g., home, clinic, workplace	Brazil: The key informant interviews (KII) took place in private and convenient locations for each participant, and the focus group discussions (FGD) was held in Hospital de Clínicas de Porto Alegre.<br>Nepal: KIIs were conducted in private locations at health facilities, schools, stakeholder offices, TPO Nepal offices, and Ministry offices. The FGDs were conducted at TPO offices. KIIs and FGDs were conducted in Nepali & English.<br>Nigeria: The FGDs were held at Department of Behavioural Medicine at The Lagos State University Teaching Hospital and at the Child and Adolescent Mental Health Service of The Federal Neuropsychiatry Hospital Oshodi Annex.<br>United Kingdom: The KIIs occurred at King's College London campuses, or at stakeholder's office spaces where appropriate, and over the telephone.
15. Presence of non-participants	Was anyone else present besides the participants and researchers?	Brazil: No<br>Nepal: No<br>Nigeria: No<br>United Kingdom: No
16. Description of sample	What are the important characteristics of the sample? e.g., demographic data, date	See **Table 1** for a breakdown by stakeholder group.
Data collection		
17. Interview guide	Were questions, prompts, guides provided by the authors? Was it pilot tested?	This multi-site qualitative study utilized key-informant interviews (KIIs) and focus-group discussions (FGDs) with adolescents and other relevant stakeholders to explore views on the feasibility and acceptability of an online risk calculator for adolescent depression. An initial deductive, cross-country interview guide was created and piloted with different stakeholders across each site. Interviewers maintained debriefing forms which captured observations of the context, emerging researcher insights, and observations on the structural implementation of the

No. Item	Criteria description	Study Information
		interviews. Data from preliminary KIIs and debriefing forms in each country were used to revise the guides for contextual sensitivity and modified for type of respondent (e.g., adolescents vs. policymakers). All subsequent interviews were constructed around a mock-up of the risk calculator.
18. Repeat interviews	Were repeat interviews carried out? If yes, how many?	Brazil: No<br>Nepal: No<br>Nigeria: No<br>United Kingdom: No
19. Audio/visual recording	Did the research use audio or visual recording to collect the data?	Brazil: Audio recording<br>Nepal: Audio recording<br>Nigeria: Audio recording<br>United Kingdom: Audio recording
20. Field notes	Were field notes made during and/or after the interview or focus group?	Brazil: Yes<br>Nepal: Yes<br>Nigeria: Yes<br>United Kingdom: Yes
21. Duration	What was the duration of the interviews or focus group?	60-90 minutes in all countries
22. Data saturation	Was data saturation discussed?	As study samples of specific subgroups (adolescents, caregivers, teachers, etc.) in some sites were small, it was not possible to aim for or examine data saturation at the site level or for minor themes. For the presence of major themes, data saturation was achieved in the total/cross-country sample.
23. Transcripts returned	Were transcripts returned to participants for comment and/or correction?	Brazil: No<br>Nepal: No<br>Nigeria: No<br>United Kingdom: No
**Domain 3: analysis and findings**		
Data analysis		
24. Number of data coders	How many data coders coded the data?	Transcripts were coded in NVivo version 12 (QSR International, 2017) by nine IDEA researchers from Brazil, Nepal, UK, and USA. A minimum inter-rater reliability of 0.7 (*Cohen's kappa*) was required by coders before moving on to independent coding (McHugh, 2012).
25. Description of the coding tree	Did authors provide a description of the coding tree?	Four parent codes: beneficial; understandable; confidential; and actionable. See **Supplemental File 4** for a breakdown of child codes for each parent code.

No. Item	Criteria description	Study Information
26. Derivation of themes	Were themes identified in advance or derived from the data?	Both. Pre-developed thematic coding was initially used, and inductive coding was then used to identify additional codes.
27. Software	What software, if applicable, was used to manage the data?	NVivo version 12 (QSR International, 2017)
28. Participant checking	Did participants provide feedback on the findings?	Participants did not provide feedback on the findings.
Reporting		
29. Quotations presented	Were participant quotations presented to illustrate the themes/findings? Was each quotation identified? e.g. participant number	See **Supplemental File 2** for more detailed quotes and tabulation by country and theme.
30. Data and findings consistent	Was there consistency between the data presented and the findings?	See **Supplemental File 2** for more detailed quotes and tabulation by country and theme; **Supplemental File 4** include anonymized direct code queries by country.
31. Clarity of major themes	Were major themes clearly presented in the findings?	Yes (major headings in the results section)
32. Clarity of minor themes	Is there a description of diverse cases or discussion of minor themes?	Yes (sub-headings in the results section)
