# Supplementary material for: No prediction without prevention: A global qualitative study of attitudes toward using a prediction tool for risk of developing depression during adolescence
Source: Glob Ment Health (Camb). 2025 Jan 3;11:e129. doi: 10.1017/gmh.2024.136 (PMC11704374; doi:10.1017/gmh.2024.136)
Supplement: Kohrt et al. supplementary material [file S2054425124001365sup001.zip › Supplemental File 2_Exerpts of mock-up of risk calculator.pdf]

Risk Calculator for Depression

1. Language

Mark only one oval.

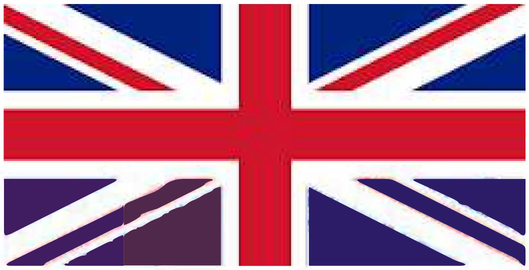

- English (UK)

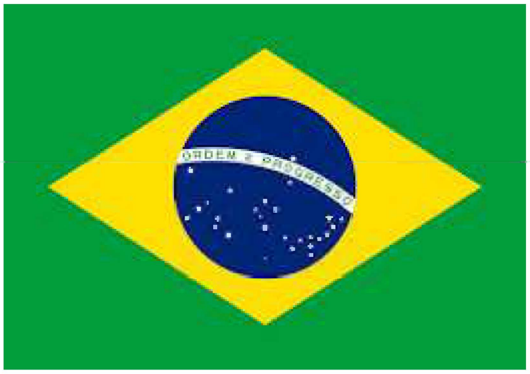

- Portuguese

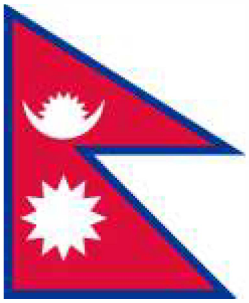

- Nepali

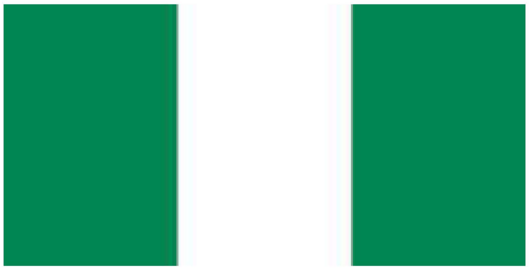

- English (Nigeria)

2. Gender *

Mark only one oval.

- Male
- Female
- Other: ____________________

3. Age

Mark only one oval.

- 14 years old
- 15 years old
- 16 years old
- 17 years old

4. Ethnicity

This will need to be adapted for each country.

Mark only one oval.

- White
- Black
- Other

Family experiences

5. How is your relationship with your mother?

Mark only one oval.

- Very good
- Good
- Regular
- Bad

6. How is your relationship with your father

Mark only one oval.

- Very good
- Good
- Regular
- Bad

7. How is the relationship between your parents

Mark only one oval.

- Very good
- Good
- Regular
- Bad

8. Have you ever been separated from your parents for a long time and cared for by someone else?

Mark only one oval.

- Yes
- No

Items #9-16 removed for public distribution.

17. In the past year, have you gotten into a physical fight in which someone got hurt?

Mark only one oval.

- Yes
- No

Other experiences

18. Did someone ever try to touch you in sexual way or ask you to touch them against your will?

Mark only one oval.

- Yes
- No

19. Have you ever drank alcohol (beer, wine, liquor), smoked cigarettes or marijuana, or used any other drugs?

Mark only one oval.

- Yes
- No

Depression risk

20. Depression risk

Mark only one oval.

- High Risk *Skip to section 6 (HIGH RISK OF DEPRESSION)*
- Low Risk *Skip to section 7 (LOW RISK OF DEPRESSION)*

HIGH RISK OF DEPRESSION

High Risk

Research has shown that items you answered are associated with future risk of depression.
If any of the questions you responded positively to are causing you distress, it is possible to seek help. You can talk about your distress with an adult your trust, a school counselor, or a health professional.

* Child Line: 1-888-888-8888, www.childline.com
You can also contact the following:

To learn more about the symptoms of depression, prevention of depression, and treatment, go to www.preventingdepression.com

LOW RISK OF DEPRESSION

Low Risk

Research has shown that you have low risk of depression. With low risk, it is still important to take of your mental health and wellbeing. You can go to www.healthyteens.com to learn more about healthy sleep, diet, and behavior.

If you know someone with several of these risks in this survey, encourage them to seek help from an adult they trust, health worker or school counselor.

This content is neither created nor endorsed by Google.

Google Forms
