# Supplementary material for: No prediction without prevention: A global qualitative study of attitudes toward using a prediction tool for risk of developing depression during adolescence
Source: Glob Ment Health (Camb). 2025 Jan 3;11:e129. doi: 10.1017/gmh.2024.136 (PMC11704374; doi:10.1017/gmh.2024.136)
Supplement: Kohrt et al. supplementary material [file S2054425124001365sup001.zip › Supplemental File 3_Anonymized code queries.pdf]

1. Code Query Output

<Arquivos\\ > - § 2 referências codificadas [0,65% Cobertura]

Referência 1 - 0,44% Cobertura

Eu acho que, de uma forma, vamos supor que eu fizesse o teste. Eu acho que eu acreditaria mais porque é algo centífico dizendo para mim que eu vou ter depressão do que alguém chegar pra mim e falar que eu tenho. Porque, como a gente falou ontem, tem gente que tem dificuldade de aceitar e de acreditar. Então, eu acho que assim, a forma de tu convencer a pessoa de que ela precisa se cuidar mais, porque ela está com alto risco de depressão é melhor.

Referência 2 - 0,21% Cobertura

Essa minha amiga, ela não gostaria, ela deve, acho que ela só ia dizer "ai, não acredito nisso, bobagem".
Acho que ela não...
Moderadora: Não ia acreditar.
Entrevistado 5: É, também acho.

<Arquivos\\ > - § 1 referência codificada [0,90% Cobertura]

Referência 1 - 0,90% Cobertura

I don't know, because I don't know how it would diagnose someone. I think it's so much stuff, so many combinations, that sometimes, it's impossible to diagnose. But depending on how it's used, it would be something good.

<Arquivos\\ > - § 2 referências codificadas [0,14% Cobertura]

Referência 1 - 0,10% Cobertura

Pessoas que tiverem baixo risco vão se sentir bem e talvez vão procurar ajudar outras pessoas. E as pessoas com alto risco talvez se cuidem melhor.

Referência 2 - 0,04% Cobertura

Prevenir suicídios. Acho que seria tipo...

<Arquivos\\ > - § 1 referência codificada [0,42% Cobertura]

Referência 2 - 0,42% Cobertura

Entrevistado : Acho que talvez alguns não, não sei.
Moderador : Talvez alguns não? Mas por que tu acha que eles não concordariam?
Entrevistado : Ah, diz "ai, não quero saber disso", só... "Sou uma pessoa normal", já vi dizer: "ah, não preciso de ajuda" não sei o que.

<Arquivos\\ > - § 4 referências codificadas [0,59% Cobertura]

Referência 1 - 0,41% Cobertura

É porque... Tipo, se tu não sabe que tu tem depressão... Através disso tu pode, tipo, ter uma ideia porque, que nem minha colega, ela falou que não tinha, mas ela foi chamada para vir aqui.

Referência 2 - 0,18% Cobertura

Porque, tipo, se tu... Tu pode, se for alto risco, tu pode prevenir isso antes.

<Arquivos\\ > - § 4 referências codificadas [1,55% Cobertura]

Referência 1 - 0,31% Cobertura

Ah, tá. Eu acho legal, tipo, na internet, também, eu gostei das respostas. E também nas escolas para a pessoa saber, tipo, como está e tal.

Referência 2 - 0,56% Cobertura

De ver e tratar antes que tenha algo... Antes de ter a depressão, porque geralmente as pessoas procuram quando já estão muito mal. E daí... Isso aqui é pra ver, tipo, quem tem risco, né? Então, seria legal para as pessoas começarem a tratar antes que aconteça.

Referência 3 - 0,22% Cobertura

Entrevistador: E você vê alguma consequência negativa de usar algo como isso?
Entrevistado: Negativa? Acho que não. Acho que só seriam pontos positivos.

Referência 4 - 0,46% Cobertura

Eu achei muito legal o que está escrito nas respostas, mas, talvez, tipo, deixar mais visível que é para... Não, mas... É, aparece bem, procurar ajuda de um profissional da saúde. Porque acho que é muito importante.

<Arquivos\\ > - § 4 referências codificadas [1,26% Cobertura]

Referência 1 - 0,06% Cobertura

Muito boa.

Referência 2 - 0,54% Cobertura

É a mesma que... Não é a mesma coisa, é a maioria que tem depressão, é os sintomas que a maioria tem que tem depressão e não sabe... Que daí, com isso, pode ajudar bastante. Daí influencia a procurar ajuda, e tudo mais.

Referência 3 - 0,23% Cobertura

Para as pessoas, para os adolescentes, principalmente. Pro futuro, não ter depressão.

Referência 4 - 0,44% Cobertura

Moderador: E que que você acha que poderia... Tu acha que poderia ser colocado, tu acha que poderia ter alguma consequência negativa para usar essa calculadora de risco?
Entrevistado: Não.

1. Code Query Output

<Arquivos\\ > - § 1 referência codificada [0,36% Cobertura]

Referência 1 - 0,36% Cobertura

Acho que seria um tipo de segurança, porque tu está mostrando pra alguém, como é que eu vou dizer, estou mostrando para a minha mãe, está mostrando para ela que eu preciso de ajuda. Mas eu me sentiria... Não, eu não daria muita bola, eu acho. Mas tempessoas, eu acho, que não se sentiriam confortável. Eu imagino pessoas deixando de fazer porque vai ter alguém olhando.

<Arquivos\\ > - § 2 referências codificadas [2,49% Cobertura]

Referência 1 - 0,90% Cobertura

[Os adolescents] Preencherem em um lugar reservado, que tenha só eles, né. Não, tipo, com alguém, porque vai que alguém olhe, daí a pessoa não vai responder. "Ah, ela vai olhar, então nem vou responder se sim." Entendeu? Ou, então, que vá perguntar "ah, tu respondeu sim ou não?". Que até aconteceu na minha escola isso. "Tu respondeu sim?" Aí eu falei "ah, não sei, questionário é meu, né.

Referência 2 - 0,26% Cobertura

Mas tem gente que fica... Não é assim, tem gente que acaba falando, e isso se torna desconfortável.

Referência 3 - 0,96% Cobertura

Porque apresenta fatos que a maioria dos adolescentes acontece, e provavelmente não contam para ninguém. Então, se tu, por exemplo, ela é um negócio de confidencialidade, se tu falar pra mim que não vai contar para ninguém, a pessoa vai confiar em ti, então, provavelmente, ela vai responder todas com verdade, né. Não vai mentir nada, não vai negar nada, porque vai estar confiando nisso, entende.

Referência 4 - 0,37% Cobertura

Então, é perguntas que não são todas que para qualquer um se fala a verdade. Por medo, ou algo assim. Mas é muito bom, é o suficiente para saber.

1. Code Query Output

<Arquivos\\ > - § 3 referências codificadas [0,78% Cobertura]

Referência 1 - 0,51% Cobertura

Baseado nessas perguntas, eu acho até que sairia que ela não tem risco nenhum, porque o relacionamento com a mãe dela é bom, apesar da desavença que eles verem por questão dela ser lésbica e a mãe dela não aceitar, agora elas estão de boas. Com o pai dela também, as coisas melhoraram. Entre os dois, a relação dos dois é maravilhosa. Os pais dela, apesar de serem separados... Ou seja, com as respostas aqui, as respostas dela seriam maravilhosas. Ela não passou por nada disso daqui e ainda assim ela... Tem depressão.

Referência 2 - 0,22% Cobertura

Não sei, eu acho que poderia ter, assim... Não vou dizer que é ruim, assim, esse teste, mas até poderia ter, assim, frases de apoio, né, "porque você é bonita, você é especial", frases pra deixar a pessoa mais feliz.

Referência 3 - 0,05% Cobertura

Ou pra encorajar mesmo pra ela procurar ajuda.

<Arquivos\\ > - § 6 referências codificadas [0,52% Cobertura]

Referência 1 - 0,04% Cobertura

É, não é tão impreciso mas também não é muito preciso.

Referência 2 - 0,18% Cobertura

Olha, quando veio o nome calculadora de risco, eu pensei, "hm, vai ser uma calculadora, vai aparecer a pergunta e tu só clica no botão sim e no botão não, e no final aparece a porcentagem de risco". Foi a primeira ideia que veio na minha cabeça, tipo uma calculadora mesmo.

Referência 3 - 0,18% Cobertura

Acho que devia ter dois lembretes: o primeiro é de que as pessoas devem ser totalmente sinceras em relação a isso. E segundo é que não se deve levar em consideração a resposta, que você tem que viver a vida da sua maneira. Não mude sua rotina por causa de um examezinho.

Referência 4 - 0,04% Cobertura

Acho que seria procurar um hobby, um passatempo.

Referência 5 - 0,05% Cobertura

Procure se... olhar ao redor pra ver se não tem uma pessoa...

Referência 6 - 0,03% Cobertura

Com sintomas de alta depressão.

<Arquivos\\ > - § 2 referências codificadas [0,30% Cobertura]

Referência 1 - 0,21% Cobertura

Tá, um guri... Deixa eu pensar em alguém. Uma garota que eu, uma colega minha. É que ela não é muito branca, acho que vou botar amarelo.

Referência 2 - 0,09% Cobertura

Achei a pergunta interessante, boa, sim, para saber mais.

<Arquivos\\ > - § 3 referências codificadas [0,50% Cobertura]

Referência 1 - 0,28% Cobertura

Essa parte aqui eu acho que... Sei lá. Por causa que acho que não tem sexo, nem idade, nem cor, nem raça para ter depressão.

Referência 2 - 0,14% Cobertura

As outras... Essas aqui eu respondi na escola já.

Referência 3 - 0,08% Cobertura

Achei tranquilo.

<Arquivos\\ > - § 4 referências codificadas [1,52% Cobertura]

Referência 1 - 0,31% Cobertura

Eu pensei que fosse... Como eu tô pensando na minha amiga, eu sei que de física ela não teve, mas de moral... Moral não sei se é a palavra.

Referência 2 - 0,44% Cobertura

Eu achei bem legal, mas eu acho que tem poucas perguntas. Não sei se tipo... Tá, as perguntas que tem são bem desenvolvidas para descobrir isso e tal, mas talvez pudesse ter mais perguntas, um pouco mais.

Referência 3 - 0,68% Cobertura

Acho que sobre a autoestima, se se acha... Como se vê, se gosta, tanto físico como mentalmente. Se se acha capaz de fazer as coisas... E, talvez, algo sobre a família, tipo, ah, como... É, na verdade, pergunta sobre isso, é, então, só isso. E as respostas, eu achei bem legal, tipo, estaria na internet essa pesquisa, também?

Referência 4 - 0,08% Cobertura

É, destacar mais essa parte.

<Arquivos\\ > - § 2 referências codificadas [0,22% Cobertura]

Referência 1 - 0,04% Cobertura

Bom.

Referência 2 - 0,18% Cobertura

É o suficiente para saber se a pessoa tem ou vai ter depressão.

1. Code Query Output

<Arquivos\\ > - § 6 referências codificadas [1,37% Cobertura]

Referência 1 - 0,11% Cobertura

Acho que pelos colégios, pelas escolas.
Eu acho que poderia ser pelas escolas, mesmo.

Referência 2 - 0,20% Cobertura

Talvez supervisor, alguém que... Fosse mais de confiança, depende também.
Entrevistado 2: Ah, eu ia dizer isso, dependendo da pessoa... (fala ininteligível)
Moderadora 2: Orientador.

Referência 3 - 0,36% Cobertura

Acho que seria um tipo de segurança, porque tu está mostrando pra alguém, como é que eu vou dizer, estou mostrando para a minha mãe, está mostrando para ela que eu preciso de ajuda. Mas eu me sentiria... Não, eu não daria muita bola, eu acho. Mas tem pessoas, eu acho, que não se sentiriam confortável. Eu imagino pessoas deixando de fazer porque vai ter alguém olhando.

Referência 4 - 0,17% Cobertura

Moderadora 2: E se fosse no celular? No aplicativo, onde é escondidinho, onde ninguém veria? Tu acha que isso facilitaria?
Entrevistado 2: Eu acho. Acho que facilitaria.

Referência 5 - 0,08% Cobertura

Porque tem gente que é mais solto, quando está só. Quando está sozinho, assim, sabe?

Referência 6 - 0,44% Cobertura

Moderadora 2: E vocês pensam mais alguma coisa sobre essa calculadora, mais alguma... O que diz aqui, o que vocês conseguem pensar para o uso delas... Vocês acham que seus colegas responderiam essa calculadora?
Entrevistado 5: Acho que sim.
Entrevistado 3: Eu acho.
Entrevistado 2: Sim, até eles responderam, quando vocês foram lá na escola, eles responderam.
Moderadora 2: O questionário?
Entrevistado 2: Isso.

<Arquivos\\ > - § 7 referências codificadas [0,51% Cobertura]

Referência 1 - 0,10% Cobertura

Se as escolas de modernizarem um pouco mais, dá pra fazer ele nessa fonte mesmo. Um aluno por vez. Tem escola... Quase toda escola tem um computador.

Referência 2 – 0,02% Cobertura

Sim, acho que... Tá bom.

Referência 3 – 0,05% Cobertura

Eu acho que deveria ficar com, em posse dos psicólogos da escola.

Referência 4 – 0,07% Cobertura

Sim, porque eles, por conhecerem os alunos, eles acabam reparando detalhes que muitos amigos deixam passar.

Referência 5 – 0,05% Cobertura

Os professores, talvez. Mas em posse dos pais, acho que não.

Referência 6 – 0,07% Cobertura

É, acho que teria que ser feito uma pesquisa com os alunos para ver se eles aceitariam, né, porque...

Referência 7 – 0,15% Cobertura

É, porque, não vão ser os professores que serão aplicadas as calculadoras. Vão ser nos alunos. Eles ficariam meio... assim, se sentindo excluídos da decisão, como muitas vezes já aconteceu na escola e eu me senti excluído de decisões.

‹Arquivos\\ › – § 2 referências codificadas [1,25% Cobertura]

Referência 1 – 0,59% Cobertura

Moderador: [Seus colegas] Responderiam esse questionário. Se ele fosse de verdade, assim.
Entrevistado: Acho que sim.
Moderador: Acha que sim? E tu acha que seria tranquilo para eles, eles não teriam nenhuma ressalva? Porque algumas perguntas são pessoais, né. E aí, que que tu acha, que que tu acha sobre as perguntas, assim?
Entrevistado: Legal, acho que sim, acho que eles concordariam.

Referência 2 – 0,66% Cobertura

Moderador: Mas quem tu acha que poderia usar essa calculadora?
Entrevistado: Ah, alguns colegas.
Moderador: Alguns colegas? Mas tu acha que eles deveriam procurar a calculadora sozinhos ou um professor colocaria para fazer na sala de aula, alguém em um serviço de saúde?
Entrevistado: É, acho que um... Algum professor mostrar, ou ele mesmo, assim, querer saber mais. Querer saber sobre a calculadora. Essa calculadora.

‹Arquivos\\ › – § 3 referências codificadas [0,59% Cobertura]

Referência 2 – 0,09% Cobertura

Acho que na escola mesmo.

<Arquivos\\ > - § 2 referências codificadas [1,29% Cobertura]

Referência 1 - 0,55% Cobertura

Entrevistado: Ah, tá. Eu acho legal, tipo, na internet, também, eu gostei das respostas. E também nas escolas para a pessoa saber, tipo, como está e tal.
Moderador: Ah, entendi. Tu pensa nas escolas, isso estar disponível e daí os adolescentes respondem?
Entrevistado: É.

Referência 2 - 0,74% Cobertura

Entrevistado: Se tá na internet, só vai achar quem procurar. As pessoas que não procurarem, daí, e que podem ter algum risco para desenvolver depressão, provavelmente não vão encontrar, não sei, acho que é isso. E, nas escolas, tipo, se partir do governo, é muito difícil de ter, ainda mais que tem muitas que nem tem dinheiro, assim, tipo, para imprimir, para...

<Arquivos\\ > - § 9 referências codificadas [4,15% Cobertura]

Referência 1 - 0,07% Cobertura

Sim, bem viável.

Referência 2 - 0,96% Cobertura

Porque apresenta fatos que a maioria dos adolescentes acontece, e provavelmente não contam para ninguém. Então, se tu, por exemplo, ela é um negócio de confidencialidade, se tu falar pra mim que não vai contar para ninguém, a pessoa vai confiar em ti, então, provavelmente, ela vai responder todas com verdade, né. Não vai mentir nada, não vai negar nada, porque vai estar confiando nisso, entende.

Referência 3 - 0,37% Cobertura

Então, é perguntas que não são todas que para qualquer um se fala a verdade. Por medo, ou algo assim. Mas é muito bom, é o suficiente para saber.

Referência 4 - 0,21% Cobertura

Quem? A minha amiga, meu irmão, até minha mãe. Uns amigos meus da escola...

Referência 5 - 0,06% Cobertura

Nas escolas.

Referência 6 - 0,13% Cobertura

Apresentando o questionário, que nem esse.

Referência 7 - 0,90% Cobertura

Preencherem em um lugar reservado, que tenha só eles, né. Não, tipo, com alguém, porque vai que alguém olhe, daí a pessoa não vai responder. "Ah, ela vai olhar, então nem vou responder se sim." Entendeu? Ou, então, que vá perguntar "ah, tu respondeu sim ou não?". Que até aconteceu na minha escola isso. "Tu respondeu sim?" Aí eu falei "ah, não sei, questionário é meu, né.

Referência 8 - 0,15% Cobertura

Um questionário. Um site com um questionário online.

Referência 9 - 1,30% Cobertura

Ou, então, algum aplicativo que tenha para ajudar ou algo assim. Porque, hoje em dia, todo mundo usa celular. Praticamente todos os celulares, todos os adolescentes usam celular. Então, se tivesse um aplicativo ou algum link que pudesse entrar para jogar um jogo e que tu possa descobrir sem fazer o questionário... Fazendo isso, distraindo, não só com as perguntas, mas um jogo, algo assim, elaborar, podia ser legal também. Não tem tanta pressão assim, sabe, que nem as perguntas, que tem, tipo, sim ou não. Podia ser uma coisa mais elaborada.

11. Code Query Output – Use of Risk Calculator Results

POLICY MAKERS (FPP)

<Arquivos\\ > - § 2 referências codificadas [0,39% Cobertura]

Referência 1 - 0,16% Cobertura

Mas eu acho que, nesse caso, tem muito adolescente que mente, né. Não vai contar a verdade. Eu acho interessante que a escola pudesse - porque a escola observa, né? Se há um bom relacionamento com o pai, com a mãe... Se há questões de violência doméstica...

Referência 2 - 0,23% Cobertura

Sim. Isso é... Falam assim, "ah o teu pai nunca quis saber, nunca te criou", sabe, "não te criou, não é teu pai." Só um pouquinho. Aí eles preferem chamar o companheiro da mãe de pai. Só que, daí, quando a mãe troca de companheiro, troca de pai? Não, ele se torna ou afetivo ou afetuoso àquela outra pessoa, lá. Tu cria mais um problema ainda, sabe... Mas eu achei muito legal.

<Arquivos\\ > - § 4 referências codificadas [2,50% Cobertura]

Referência 1 - 0,19% Cobertura

Eu acho, eu acho... A minha resposta não é muito animadora, mas eu vou falar a verdade, que eu acho que é o que vocês querem de mim. Eu acho que é completamente não factível no sistema de saúde público.

Referência 2 - 1,10% Cobertura

Os motivos são: primeiro, eu acho muito grande, né, acho que é difícil alguém conseguir fazer um questionário tão grande. Segundo, conceitualmente, eu acho que isso aqui só funcionaria se o operativo positivo, ou seja, se a pessoa que preenchesse tivesse uma chance altíssima de ter um quadro grave. E eu pensaria algo do tipo assim, suicídio, né. Não depressão, assim.

Acho que hoje, depressão, o que a gente tem que fazer é tratar a depressão, a gente nem trata a... Por isso que a... O ponto, assim: a gente nem trata direito os deprimidos, aí a gente quer tratar os riscos para depressão. Os tratamentos são menos consolidados na literatura, a gente não sabe o que realmente trata. Então, o meu...

A minha questão... E outro ponto é: um profissional da saúde da rede pública ele não está... Ele não está preocupado com o desfecho. Ele está preocupado com múltiplos desfechos. Ele não está preocupado com a depressão, ele está preocupado com depressão, com TDAH... Então, eu também não acho que uma calculadora de risco específica para o transtorno mental, né, é algo possível, assim, no sentido de factível para o sistema de saúde de uma forma geral.

Referência 3 - 0,71% Cobertura

Uma calculadora de risco... Eu tenho dúvidas para uma calculadora de risco para transtornos mentais gerais, se seria possível porque eu não vejo nem a gente tratando as coisas mais básicas. A gente está muito antes, a gente está muitos passos antes de começar a pensar no risco. Outra coisa que me preocupa é a prevalência. Então, depende muito da calculadora de risco, depende muito da capacidade preditiva dela. O quanto ela, assim... Eu estou assumindo, assim, que ela é super preditiva. Mas isso vai depender muito de quantas pessoas são incluídas nesse grupo. Então, por exemplo, se o risco... Se a calculadora começa a classificar em risco 30% da população, é completamente não factível a gente fazer intervenções em 30% da população.

Referência 4 - 0,49% Cobertura

Ou seja, do meu sistema de saúde para poder fazer o targeting, ou priorizar as ações para um grupo e não para o outro. Faz sentido para mim. Um cara fez um episódio depressivo, ver se ele tem... A chance de continuar tendo episódios depressivos ou a chance dele ficar bem. Faz sentido para mim. Uma calculadora de risco geral para a população não faz nenhum sentido para mim, né, porque eu acho que está ampliando a nossa necessidade de atenção e cobertura do sistema de saúde que já nem cobre quem está deprimido.

<Arquivos\\ > - § 4 referências codificadas [2,39% Cobertura]

Referência 1 - 0,93% Cobertura

Eu acho, assim, vendo os pontos aqui abordados, eu acho que ele é amplo, que ele pega questões familiares, pega as questões escolares e outros aspectos relacionados ao abuso sexual, baixa autoestima, e eu acho que é interessante, inclusive para a autoaplicação, monitoramento, se for... E pensando, já que a gente tinha falado na própria questão dos agentes comunitários, eu acho... Depende, também, tem que ver qual é o ponto de corte, né. Para ver, para não ser algo que todo mundo cai aqui dentro ou que ninguém entre, né, então, vendo de acordo com um ponto de corte para identificar quem é de alto risco, eu acho que como screening, assim, como rastreamento, me parece válida essa ferramenta aqui.

Referência 2 - 0,32% Cobertura

Moderador: E como é que tu percebe, assim, a gente dizer para alguém ou tu ler sobre ti mesmo e dizer "tu tens alto risco para tal coisa", assim, né?
Entrevistado: Eu acho, algumas vezes, é uma forma de ajudar um insight das pessoas.

Referência 3 - 0,90% Cobertura

Mesma coisa que fazer o risco cardiovascular. A gente pega ali os dados fuma, qual é a idade, qual é o colesterol, como é que está o nível da pressão, olha. Teu risco é isso aqui, mas tem que explicar, né, que que é isso aqui, qual é o risco disso? Isso é uma calculadora, que não vai definir, mas ela te dá uma chance maior de 10 anos, tu ter um infarto, tu ter um derrame... Se mudar isso, isso e isso, são pontos que olha, a gente pode trabalhar, fazer acompanhamento aqui, quem sabe, ver os pontos... Acho que ele é uma ferramenta que auxilia para o profissional de saúde poder trabalhar uma situação que às vezes as pessoas não, negam, ou não se dão conta, mesmo, não sabem.

Referência 4 - 0,25% Cobertura

Uhum. Ele ajuda tanto para o indivíduo quando para o profissional de saúde. Que também vai poder, a partir da ferramenta, discutir. Se quiser pegar pontos específicos para discutir.

<Arquivos\\ > - § 3 referências codificadas [1,74% Cobertura]

Referência 1 - 0,04% Cobertura

Acho fundamental.

Referência 2 - 0,51% Cobertura

Eu achei fantástico. Eu quero te dar esse protocolo porque as meninas foram (?) aqui, e a gente quer implementar nas escolas, pelo menos em algumas inicialmente, o projeto piloto. Que que é o protocolo? Ele te dá situações que podem, né... Quando acontecer, quais são os protocolos que a gente deve usar? É o que tu traz aqui, de certa maneira . Esse questionário, dá uma modificada...

Referência 3 - 1,19% Cobertura

Não, o lado... O assunto suicídio é uma situação que... Tem muitos assuntos que a gente não fala na adolescência, que ainda achando que com isso a gente vai incentivar os jovens. Eu não sei quem é que usa um exemplo de que fazer a campanha pela Brigada Militar numa escola, para que não fizessem trote. Aí, no dia seguinte, os trotes triplicaram. Então, existe essa situação ainda, né, vamos falar de drogas? Não, mas a

gente vai incentivar o uso, né. Eu sou contra isso, acho que quando a gente não fala, a gente corre o risco de acontecer as coisas. Mas eu... Né, eu sou a favor. Mas isso tem que ser... Para tu intervires numa escola, tem que ser aceito pela Secretaria do Município, que é uma, tem que ser uma discussão com eles, mas eu acho que vocês tem que ter uma metodologia de proposta para isso, como que a gente pode usar a calculadora, se a gente está de certa forma incentivando um assunto tão sensível.

<Arquivos\\ > - § 4 referências codificadas [2,05% Cobertura]

Referência 1 - 0,93% Cobertura

Sim. Eu fico pensando assim qual seria a conduta, como modificaria a conduta do profissional de saúde perceber que um paciente adolescente está com um alto risco, né. Por exemplo, em avaliações de risco cardiovascular ou tabagismo, a partir de escores a gente tem condutas já meio protocolares, já meio definidas, que tipo de abordagem. Talvez fosse importante ter, quanto tu recebes essa certificação... Uma coisa... É que é a orientação para o próprio adolescente, não sei se a pergunta é mais para o adolescente ou para o profissional. Pensando no meu uso, como profissional na prática, eu pensaria em que... Que atitude tomar como profissional agora na avaliação desse adolescente? Eu tenho que talvez ter consultas mais regulares, obter uma avaliação familiar... Ou, aí sim, fazer uma visita à escola ou a escola poder ter um espaço conjunto.

Referência 2 - 0,65% Cobertura

Outra coisa talvez é a pré-escola, a escola fez o questionário, identificou, né, esse adolescente com risco. Qual é a conduta, encaminhar para o serviço de saúde de alto risco? Talvez até pensando no escolar, isso pudesse ser mais útil, ou no profissional, né, um médico que estivesse fazendo essa avaliação, o próprio agente voluntário, o enfermeiro da unidade, né, e vir talvez já atrelado com algumas abordagens que talvez já tenham uma evidência mais clara - não necessariamente medicamentosa - de prevenção da evolução para depressão, ou... Seja vincular espaços de atividade física, artística...

Referência 3 - 0,16% Cobertura

Entrevistado: Talvez a conduta vá mudar de acordo com o profissional.
Moderador: Isso, ter uma orientação de continuidade, de conduta.

Referência 4 - 0,31% Cobertura

Entrevistado: É, e talvez, para o jovem, incluir aqui, além do CVV, a sua unidade básica de saúde como referência porque é onde geralmente se tem o primeiro contato com o sistema. Caso seja necessário encaminhar para um atendimento especializado ou mesmo já fortalecer esse vínculo, né.

<Arquivos\\ > - § 4 referências codificadas [2,13% Cobertura]

Referência 1 - 0,97% Cobertura

Pois é, eu não sei se eu usaria a expressão "alto" e "baixo", né. Não sei. Eu acho que se poderia estabelecer um, essa... Esse escore, né, talvez essas três zonas, digamos, de cores, tipo assim, laranja, vermelho, laranja e amarelo, sei lá. Azul, não sei. E aí, estabelecer esse gradiente, digamos assim, né, dizer "olha, tu tá nesse nível aqui que é o nível que tem maior risco, menor risco." Porque acho que todos têm risco, né. Cada um de nós tem risco. Se isso vai ser maior ou menor estatisticamente, mas esse maior ou menor estatisticamente só serve no agregado, ele não serve para cada indivíduo. Esse é o ponto. Porque a gente pode ter, por exemplo, uma resposta de um jovem, pelo escore, vai ser lá embaixo, então ele tem baixíssimo risco, e ele pode ser o primeiro a desenvolver depressão, entendeu? Porque, porque, porque a estatística, ela só produz noções verdadeiras no agregado. Então, se eu tiver 10 mil jovens que responderam, eu vou chegar no agregado, tem um grupo aqui que tem mais risco. Mas isso não é verdadeira para cada um, é válido para o grupo.

Referência 2 - 0,24% Cobertura

Então, eu devolver para o indivíduo isso aqui não parece bom para mim, entendeu? Eu diria "olha, tu está num grupo aqui que tem um risco x, tem um risco maior ou menor, coisa e tal, mas isso não quer dizer que tu vai ter, nem que tu não vai ter.", entendeu?

Referência 3 - 0,14% Cobertura

É só um alerta. É preciso cuidar isso, coisa e tal, mas cada pessoa vai responder, mesmo de baixo risco, pode ter depressão. Como indivíduo, né.

Referência 4 - 0,78% Cobertura

E cores não muito diferentes entre elas, eu penso em tonalidades mesmo, um gradiente para marcar o seguinte: não há o céu e a terra, entendeu? Não é água e vinho. Há, simplesmente, um gradiente. Para transitar para um ou para o outro, é muito simples. Para a pessoa não ficar "não, eu tenho baixíssimo risco, então, pode não se preocupar." Seria um efeito ruim isso, né. Não, tu tem tais riscos, agora, enfim, na média, está dentro da média, está abaixo da média, tá bem. Eu acho que é isso. Na verdade, o valor estatístico desse instrumento ele existe no agregado para a política pública. Então, por exemplo, se a gente percebe lá que a escola, uma escola tem um grupo grande de alunos, né, e grande parte desse grupo está no escore mais alto, então algo nessa escola não está bem, entendeu, vai ter que ser enfrentado. Mas acho que, pro indivíduo, não deveria ser...

USERS (US) – FOCAL GRUP WITH PARENTS

<Arquivos\\ > - § 7 referências codificadas [1,15% Cobertura]

Referência 1 - 0,19% Cobertura

É um estudo que é, pra mim, acho que é o maior, o mais importante estudo, assim do ser humano é sobre essa doença porque é uma doença silenciosa. Disfarça no sorriso, né, disfarça numa foto do Face [Facebook], não querem conversar. Então, eu acho que falar sobre depressão, ainda mais na adolescência que é tão delicado, né, é importante, fazer questionários, aplicar, depois fazer a pesquisa, né, quantitativa, qualitativa, né.

Referência 2 - 0,05% Cobertura

Mãe 2: O meu responderia.
Mãe 1: Eu acho que o maior desafio seria fazer falar. Exercitar a fala.

Referência 3 - 0,12% Cobertura

Mãe 5: Vamos tratar.
Mãe 2: Vai mexer com a cabeça dele. (inaudível)
Mãe 1: Mas eu não sei se a ideia era trazer pra ele primeiramente, pra esse adolescente, que ele foi supostamente diagnosticado com possíveis possibilidades de depressão.

Referência 4 - 0,04% Cobertura

Mãe 5: Eu acho que tem que falar sim. Eu acho que tem que falar sim "ó, tu não tá bem".

Referência 5 - 0,08% Cobertura

Mãe 2: O meu diria, "Ah, achei frau". É a primeira coisa. Ele diria, "Ah, falaram tal coisa, que frau, né, mãe?" Que é o que ele diz, "que frau". O que que é "frau",[nome do participante]?

Referência 6 - 0,07% Cobertura

E acho que vocês fazem uma vez por ano, né, se tivesse condições desse encontro, de seis em seis meses, até pra ver como tá, a evolução, é um estudo, né.

Referência 7 - 0,60% Cobertura

E acho muito importante, porque acho assim. Talvez ele vendo outras pessoas, ele não vai achar que é besteira. Talvez em grupo, ele veja que a coisa é séria, é mais válido do que sozinho, porque lá ele se sente obrigado a ir naquela consulta, já tá contrariado, já tá revoltado, acho que ela acaba falando coisas que talvez a gente não saiba, admitindo pra ele algumas coisas, admitindo que é um problema, e vê que não está sozinho, e a gente dizer, não, meu filho te abre comigo, toda mãe fala, todo mundo fala. A minha filha tem 28 anos, é casada, tá bem, graças a Deus, bem empregada, e ela se preocupa muito com ele. Então hoje ela tem um condomínio muito bom, tem piscina, ela chama ele pra lá, conversa, e ela me contou um dia, mãe, o [nome do participante] me disse isso, e aí eu pergunto, ah, mas não falou, [nome do participante], daí ele diz, ah, não falei? Então esqueci. Que ele age com muita naturalidade. Talvez seja grave pra mim, pra ele não, e talvez em um grupo ele perceba, não, é sério mesmo. Eu acho que ele não está levando a sério. Ele não debocha, mas ele não tá levando a sério. Ele só diz nada a ver. Suspira, aquela coisa de adolescente revoltado, uma suspirada, e comenta em casa, ai, nada a ver, aquela doutora foi uma coisa nada a ver, que doutora Frau, e aqui eu acredito que ele esteja prestando atenção, que tem mais jovens, bem por aí.

SOCIAL WORKERS (PAS)

<Arquivos\\ > - § 2 referências codificadas [0,46% Cobertura]

Referência 1 - 0,20% Cobertura

Entrevistadora: E quais seriam os benefícios desta ferramenta?
Entrevistada: Será fácil de diagnosticar as causas né, seria interessante.

Referência 2 - 0,26% Cobertura

Eu espero que não, mas acho que não, na equipes que trabalhei não havia isso, no setor saúde é mais tranquilo, mas em outras áreas poderia ter o estigma.

<Arquivos\\ 1> - § 4 referências codificadas [1,71% Cobertura]

Referência 1 - 0,49% Cobertura

Assim, um local, um posto, na escola, a Direção, algo assim. Mais ali identifica que a pessoa já tem pegou, né, porque de repente a pessoa vai pegar lá na minha casa, uma suposição, e vai pegar aqui na UBS e na escola. Então, pra que fazer três, é um só que é pra ser feito. É que tem adolescente que adora escrever mesmo, que adora fazer umas coisinhas, tem uns que gostam, outros não. Eu acho que é onde deveria ter os acessos.

Referência 2 - 0,45% Cobertura

Moderadora: Mas assim, fiquei pensando, tá, o adolescente poderia pegar, tá na internet, vamos dizer, mas daí tá, tu achas que eles chegariam nesse questionário pra ser respondido?
Entrevistada: Não, não. Teria que ter uma visualização para saber que tem aquele questionário. Por que pra nós, teria nós falando, olha, tem esse questionário, então automaticamente, não teria problema nenhum.

Referência 3 - 0,44% Cobertura

Moderadora: Tipo uma campanha para os adolescentes fazerem. Mas tu acha que teria algum problema, por exemplo, um adolescente que recebesse a classificação de alto risco, tu acha que teria algum problema, isso geraria algum desconforto, ou algo assim?
Entrevistada: Não, porque o outro não vai ver.
Moderadora: No sentido então de ter um resultado individual assim, só pra ele.

Referência 4 - 0,33% Cobertura

É, mais é uma conversa. E dentro deste cálculo, dessa pesquisa que vocês querem fazer, particularmente, eu não tenho essa... não tenho esse cunho pra fazer isso. Mas deveria de fazer pros adolescentes. Nós temos saúde mental, mas mais é pros adultos. Fazer pros adolescentes, diferenciados.

<Arquivos\\ 2> - § 3 referências codificadas [1,33% Cobertura]

Referência 1 - 0,22% Cobertura

Mas acho até que pra adolescentes eles conseguem até se abrir, acho também que é algo que não tá sendo falado pra ninguém, talvez eles consigam externar todas as questões pessoais.

Referência 2 - 0,37% Cobertura

Acho que a depressão ainda é um tabu a ser discutido. Então acho que o desafio maior é poder ultrapassar esse tabu, pra que essa ferramenta seja de fato utilizada, da melhor forma possível, e que alcance os adolescentes que precisam passar por esse teste para poderem ser orientados a procurar auxílio.

Referência 3 - 0,74% Cobertura

Acho que os benefícios são poder fazer uma reflexão, uma auto-reflexão do seu comportamento, do que pode estar levando a uma possível depressão, e ser identificado precocemente, porque às vezes são situações corriqueiras do dia a dia, que não se atentam que isso pode ser algo característico de desenvolvimento de algum quadro depressivo, então acho que sim, pode ser algo para ser identificado cedo, algo que possa ser prejudicial, é, talvez, algum sentimento, algo que possa despertar ao responder essas questões, talvez até de choro, de angústia, de... esses sentimentos acho que podem ser despertados.

<Arquivos\\ > - § 6 referências codificadas [1,50% Cobertura]

Referência 1 - 0,56% Cobertura

Moderadora: E tu veria o uso desse instrumento, então, que te daria essa... Por exemplo, o adolescente responderia ou com o profissional ou sem o profissional, enfim, e te daria essas duas possibilidades de risco ou sem... Ou sem o risco, como tu viu ali. Tu verias isso aplicado só em situações de consultório, de posto de saúde, tu verias outro uso para essa, para esse instrumento? Por exemplo, numa escola, numa campanha...
Entrevistado: A única... Uma das... Eu acho interessante, poderia, só que tem que se cuidar muito, porque em escola, principalmente, tem umas perguntas ali, por exemplo, na família, se alguém já te tocou, se alguém já te bateu, que às vezes ela é espalhada pela escola. Então, tem que ter um treinamento, uma orientação porque muitas vezes a família vai lá, explica a situação daquele adolescente, daqui a pouco tá todo mundo sabendo.

Referência 2 - 0,11% Cobertura

Moderadora: Tu estás dizendo, então, que teria que ter um certo...
Entrevistado: Cuidado, eu acho.
Moderadora: Cuidado e treinamento de quem fosse usar.

Referência 3 - 0,08% Cobertura

Entrevistado: É, porque tem as respostas ali que poderiam, né. As pessoas comentam, então, é bem complicado, assim

Referência 4 - 0,37% Cobertura

Para fazer um encaminhamento mais adequado, né, porque, às vezes, o aluno é quietinho, não incomoda e é isso que acontece às vezes nas escolas, vai bem, notas boas. Uma criança quieta. Então, essa criança não tem problema nenhum, né. Nunca deu problema na escola, tá sempre bem. Então, eu acho que tem chance de acontecer essas... Se não é bem trabalhada essa calculadora, né. Mas ela é importante, porque daí a

escola poderia fazer um encaminhamento melhor, né, porque antigamente as escolas encaminhavam, tinha um serviço aqui chamado NASCA, que era um ESCA, tá?

Referência 5 - 0,09% Cobertura

Poderia ser um auxílio, porque daí já vinha para a Unidade Básica de Saúde, que também tem a sua tabela, já vinha meio caminho andado, né.

Referência 6 - 0,28% Cobertura

Seria nas escolas. Do treinamento e atendimento de quem aplica, né. Porque daí, "ah, então ele tá sendo vítima de abuso." A gente já teve situações, assim, que essa questão do abuso veio depois qualquer coisa que aconteça com a criança, "ah, é porque..." A escola, sem querer, vaza a informação, daí os outros alunos falam, daí fica a situação pior, né. Então, tinha que ver isso aí, com um treinamento bom, assim, não divulgar.

<Arquivos\\ > - § 4 referências codificadas [2,74% Cobertura]

Referência 1 - 0,54% Cobertura

Eu acho que bem aquilo que eu te falei, esse é um questionário que, tipo assim, eu posso... Eu vejo ele podendo ser aplicado em escolas, principalmente, uma ferramenta que pode ser trabalhada com escolas principalmente da rede pública, acho que como, apresentar como um protótipo de atenção até para as Secretarias de Saúde, de Educação, porque são, tem que estar nos espaços de circulação deles, o espaço maior de circulação deles é a escola. Então acho que é um mecanismo que ele pode ser disponibilizado para ser feito nas escolas. Ser apresentado como um projeto de intervenção para escolas... Seria o principal... Seria uma ferramenta muito importante, até para a política, mesmo.

Referência 2 - 0,45% Cobertura

Eu acho que o principal é não ser identificado. Para que tu possas ter respostas mais claras. Talvez vocês possam deixar é um campo que vocês podem... Porque geralmente a gente consegue saber... Se tu direciona ele para ser aplicado numa ferramenta, eu não sei se pode se criar um dispositivo em que tu possa identificar, quem é, digamos, esse adolescente que tem alto risco, ele possa ser identificado. E aí, dependendo das respostas, a escola pode acessar esse adolescente. Ou através de um espaço de conversa com os pais, ou alguma uma ferramenta mais, que possa permitir...

Referência 3 - 1,09% Cobertura

Eu acho que ele pode ser trabalhado ou com famílias, ou trabalhado com turmas. Eles podem aplicar numa turma e eles podem perceber que naquela turma eles vão ter três, quatro ou cinco adolescentes com fatores de risco mais... Então, essa turma vai ter algum tipo de discussão e de conversa sobre as questões de tristeza, de depressão, de riscos. E que, daí, tu vai poder dar mecanismos, tu vai poder explicar melhor quem ele pode procurar, quem ele pode falar... Então, poder falar isso, assim. Às vezes, quando tu dá uma resposta, "Procure um adulto que você confia, o professor na escola, o adulto que você confia", muitas vezes, quem é o adulto? Então, explicar para ele, "quem é o adulto que tu confias?". Às vezes, não é teu pai, nem tua mãe, mas ah, tua tia, teu tio, ou teu primo que não sei aonde né, alguém que tu acha que realmente é um amigo da família de vocês, que vocês gostam muito... Então, isso...

E as famílias hoje em dia estão cada vez menores e sem muitas redes de apoio. Então, muitas vezes tu tens famílias monoparentais, assim, que é ou o pai ou a mãe com os adolescentes jovens, e eles não têm essa rede extensa, assim, não tem tio, não tem isso. Mas aí tem alguém da família, um amigo mais próximo, ou um pai de amigos, ou uma família de amigos que eles frequentam juntos na escola. Então, esses são os sujeitos que ele pode trazer de uma forma mais objetiva, assim.

Referência 4 - 0,66% Cobertura

Eu acho que o desafio maior dele é tu transformar ele em um instrumento que realmente dê resolutividade, para percepção disso nos espaços em que a adolescência circula. Acho que o grande desafio dele é para

que ele possa ter este lugar. De ser um instrumento que realmente vá dar resolutividade, ou vá dar principalmente uma ideia, digamos, dentro dum espaço que seja algumas escolas no território de Porto Alegre, tu poder perceber alguns fatores que são mais expostos nessa região de Porto Alegre, pega uma outra região de Porto Alegre, e tu pode aplicar ele em alguns espaços e fazer uma... As primeiras percepções, quer dizer, os componentes talvez de uma escola de periferia sejam outros riscos maiores. Numa escola do bairro de classe média alta vão ter outras dimensões também de percepções sobre essas questões de depressão e tristeza.

<Arquivos\\ > - § 4 referências codificadas [0,83% Cobertura]

Referência 1 - 0,55% Cobertura

Eu acho que o diálogo. No momento que um profissional consegue aplicar com um adolescente isso, consegue ter esse momento de falar sobre essas questões de relações ou de saúde mental do adolescente possibilita com que ele, o instrumento em si, pra mim, ele é um instrumento, né, que vai possibilitar talvez o diálogo entre o profissional e o adolescente e nesse momento tentar estabelecer uma relação de vínculo. Se ele for aplicado de uma forma horizontal, "Não, eu sou o detentor do saber, da saúde, vim dizer como tu deve se adequar e ver se tu vai sofrer e eu vou ter que te medicalizar".

Referência 2 - 0,03% Cobertura

Um instrumento de relação, é.

Referência 3 - 0,16% Cobertura

Pode, pode vir coisas que o profissional não tá preparado pra ouvir. Eu sempre acho que o adolescente tá Preparado, sabe, ele tá disposto a falar. Quem não tá é o profissional.

Referência 4 - 0,09% Cobertura

O profissional talvez não, não queira ouvir que ele foi abusado pelo pai, que é gerente do banco.

<Arquivos\\ > - § 1 referência codificada [0,32% Cobertura]

Referência 1 - 0,32% Cobertura

Sim. Eu acho que poderia ser usada até como um instrumento, mas... esses dados coletados, pra depois cobrar que o atendimento seja mais, porque é muito restrito. Pros nossos que estão em abrigos já é difícil, imagina uma pessoa normal, né, que tem baixa renda e...

<Arquivos\\ > - § 3 referências codificadas [2,29% Cobertura]

Referência 1 - 0,30% Cobertura

Olha só, "Você pode conversar...", isso aqui é aquilo que eu te falei, tem que ter canais de saúde, canais de conversa e de escuta, né. Só que esses profissionais também tem que saber o que fazer, né, para não patologizar, pra não assustar, ou pra dizer, "Ah, não, tudo vai melhorar, deixa disso". E daqui a pouco tu tá devolvendo pra casa um adolescente com risco...

Referência 2 - 1,13% Cobertura

Uau. Eu fico pensando, assim, por exemplo, dentro das políticas públicas, entre as políticas públicas, a gente costuma associar suicídio etc. com saúde mental, com doença, saúde, política de saúde. Só que acho que isso aqui tem que, os professores das escolas tem que conhecer. Eu não sei e daí, [nome da entrevistadora], acho que tu tem que vir lá com o [nome do pesquisador], o coordenador da pesquisa, qual é o risco de se banalizar essa ferramenta, né. Porque, assim, ó, eu não sei se como a escola, a escola é uma fonte, é um espaço extremamente rico, necessário, ele se impõe pra esse trabalho, bom, tô falando de adolescente, onde é que a gente vai encontrar, né? Uma parte deles tá na escola. Então, assim, eu não sei se não seria, a

escola não seria o primeiro lugar que pudesse saber ou que tem essa ferramenta ou... não sei se daria, se seria indicado o uso indiscriminado. Todo adolescente nós vamos aplicar. Não sei. Mas eu acho que a escola é o primeiro espaço porque é lá que eles estão. Né. É lá. Depois, eu acho que os profissionais de saúde... da, da, assim, poderiam ter... essa, serem conhecedores de que existe esse tipo de feramente e que, bueno, quando identifica alto risco, ele é um dos profissionais que tem que ir, não assim, "Agora eu vou te encaminhar pra alguém", não, né. Ele poder ser o profissional e dizer "Bom, deu aqui"... Tu entende, usar no cotidiano de trabalho quando começa a perceber que tem lá uma... um risco de...

Referência 3 - 0,85% Cobertura

Entrevistado: O desafio maior que eu vejo é que, por melhor que estejam feitas as perguntas, elas muitas vezes não vão sinalizar, né, a gente não consegue dar conta. Esses questionários, essas escalas, eles apontam tendências, né. É isso que eu te disse, daqui a pouco... tem perguntas que vocês não conseguiram pensar e que podem... né. Porque a gente não consegue pensar tudo. Eu acho que os adolescentes responderiam na boa, eu acho que eles fariam. Agora... talvez tenha alguma outra, alguma outra tipo de indagação que daria mais... mais condições de, de identificar. Eu, por exemplo, me lembrei dessa coisa da indiferença, porque a gente sempre pensa famílias violentas, famílias, né, que estão em extrema vulnerabilidade... e às vezes elas nem são violentas, nem estão em vulnerabilidade. A não ser que a gente pense que indiferença seja uma violência. Mas, assim, esses dois exemplos que eu te dei são de famílias que não têm violência, pelo menos até onde eu saiba, não tem nenhuma questão econômica, social, não tem abandono, abandono físico, mas talvez tenha abandono efetivo.

<Arquivos\\ > - § 1 referência codificada [0,16% Cobertura]

Referência 1 - 0,16% Cobertura

Isso, isso. Eu acho que é importante. É uma demanda, né. Porque, daí, tu vai para um público alvo de x número, mas traz retorno porque às vezes é uma pontinha, né. Uma pontinha ali do... Quem que... Como é que foi estipulado isso aí? Essas perguntas já vieram do...

<Arquivos\\ > - § 7 referências codificadas [2,67% Cobertura]

Referência 1 - 0,18% Cobertura

Buscar mais. Eu ficaria em torno da notificação. Se adenter mais... no sentido vamos ter que prestar - os (?) diz assim, vamos ter que buscar tanto adolescente quando familiar, mas não digo tornar público, mas...

Referência 2 - 0,15% Cobertura

Pensando o marco regulatório, né, a normativa ali, ECA, né. Primazia do estado, toda aquela situação. Também. E que talvez aqui esteja desencadeando algum sofrimento, né.

Referência 3 - 0,35% Cobertura

Alto risco, baixo risco... É, o diagnóstico, né, agora que vem... tá em alto risco, tá em baixo risco. Eu não sei se só essa ferramenta seria o suficiente já pra algo tão fechado. Alto risco baixo, baixo risco, né. Visto que é todo um contexto, um conjunto de situações. Porque nem sempre o que a pessoa traz aqui, apesar que nós temos que nos embasar nessa fala, nesse manifesto da pessoa, é o suficiente, né.

Referência 4 - 0,12% Cobertura

Pra caracterizar, né. Eu acho que ela vem a complementar. Ela vem a contribuir, ela, ela faz, ela também faz parte do rol, mas não só isso.

Referência 5 - 0,60% Cobertura

Isso, né, eu acho que diante das respostas, sinalizou ali algumas questões, né, que seriam relevantes, né, você, adolescente, dar uma atenção melhor. Visto que, entendendo que, o quanto se tem cientificamente

comprovado que pessoas que fazem uso de droga sintética, e aí trazer. Né. Pois é, tu vivenciou uma situação de violência sexual, o quanto isso, né, impacta na vida das pessoas. Isso é um sinalizador que merece uma atenção, né. E aí não dizendo é alto ou baixo, mas que é um risco. Porque até então a gente também não sabe qual vai ser o de resiliência de cada um pra mim dizer se aquilo foi alto ou baixo – sim, é impactante, mas como é que é o teu processo de resiliência em relação a tudo isso?

Referência 6 – 0,53% Cobertura

Moderadora: Então, vamos assim, tu não classificarias em nem alto, nem baixo. Tu dirias, assim, você apresentou tantas situações associadas à... o problema depressão, ou alguma coisa assim...
Entrevistado: Que pode desencadear, ou já desencadearam. É uma janela ali que tá entreaberta.
Moderadora: Sim, tu não te preocuparias em classificar a pessoa, mas tu dá o, de dá uma, descrever o...
Moderadora: Isso, em função que, como é muito subjetivo e a gente não sabe como é que aquilo diz sobre o de resiliência de cada um, como é que cada um vai lidar com tudo isso, mas que ali é uma janela.

Referência 7 – 0,74% Cobertura

É, porque, aquilo que eu te falei, apesar de eu ver uma pessoa já com aspecto de maturidade, mas eu não saberia, e eu não saber se a medicina teria condições, de fato, de mensurar como é essa tua sistemática de pensamento, como é que tu tá vendo tudo isso, visto que é um adolescente, tá com idade da adolescência, mas será que seu grau de maturidade tá qual nível, né? A gente não tem como, de alguma forma, mensurar isso e também fechar isso dentro de uma caixinha, visto que cada pessoa é um universo, cada um tem o seu, né, suas particularidades. Eu, [nome da entrevistada], não fecharia. Deixaria mais aberto até pra ter mais possibilidades de trabalho, que aqui já vem já de alguma forma me trazendo, trazendo um perfil, eu consigo traçar um perfil, né, mas não enquanto avaliação, mais um perfil. E aquelas aberturas ali no sentindo de, eu acho que aqui , começa a nortear.

<Arquivos\\ > - § 3 referências codificadas [2,14% Cobertura]

Referência 1 – 0,88% Cobertura

Entrevistada: Daí eu vou te fazer uma pergunta. Quando vocês dão esse questionário para esse adolescente, vai estar isso aqui, ou não? Não, né? Vai estar até o 20, né?
Moderadora: Na verdade, ele seria levado ou pra esse, ou pra esse, de acordo com as respostas.
Entrevistada: Eu te entendi. Mas o adolescente vai ler isso aqui? Por que ele vai ver. Uma coisa é ele te responder, só te responder. E aí tu fazer a tua marcação e tu ver. Se essa criança tem alto risco ou baixo risco, o baixo risco a gente pode dizer para a criança, olha, tu tem que te cuidar, assim, assim, assim. Mas quando dá o alto risco, é uma coisa que se ela sabe, já fica preocupante. Então eu acho que essa parte de risco, risco para depressão, pra baixo, isso teria que ficar com vocês.

Referência 2 – 0,52% Cobertura

Se esse paciente, esse adolescente de 14 e 17 anos é da UBS, daí eu acho maravilhosamente maravilhoso ir para o prontuário família. Botarem no sistema, porque o sistema aqui da UBS é único hospital, então onde ele quer que ele for consultar, quando forem ler ali o relatório dessa criança, já vem que ele já respondeu um questionário. O trabalho de vocês vai ser muito melhor para nós, que somos profissionais. Até mesmo para vocês, eu acho maravilhoso.

Referência 3 – 0,75% Cobertura

Moderadora: Mas tu achas preocupante se ele vir que tem alto risco? Por que, tu achas que ele vai se comportar mal, ou vai receber mal...?
Entrevistada: Sim. Sim. Ou ele entra numa depressão profunda porque ele acha que tá com... Ou – o alto risco, tu estás falando? Estás falando do alto risco? –. Eu acho que ele entra numa depressão mais profunda, ou ele cai mais naquilo, por que muitas vezes não quer dizer que é depressão. O alto risco. Ou ele se atira mesmo na droga, ou no álcool, ou na prostituição. Como todo mundo diz, me fizeram a fama, vou deitar na cama. Já disseram para mim que é um alto risco. Então tá deito na cama e deixo que falem.

SCHOOL PROFESSIONALS (PE)

<Arquivos\\ > - § 2 referências codificadas [0,31% Cobertura]

Referência 1 - 0,12% Cobertura
Acho que o risco é assim, eu não posso dizer que a fulana apresentou tais e tais sintomas e já é depressiva, a gente tem isso daí, né? Esse cuidado de ser algo muito precipitado.

Referência 2 - 0,19% Cobertura
Bom, acho que o benefício é se realmente ele tá com todos aqueles sintomas, bom, que bom que veio para me alertar. São duas situações bem delicadas, porque eu, leiga, eu não posso sair dizendo "é, tá assim porque está em depressão". Eu não posso sair dizendo tudo isso.

<Arquivos\\ 0> - § 4 referências codificadas [1,27% Cobertura]

Referência 1 - 0,23% Cobertura

Pra o adolescente responder precisa confiar, né, precisa... tá seguro que aquilo vai ser feito de uma forma legal, tem que ter confiança, senão ele responde o inverso muitas vezes, e se vai dar um retorno para ele, se a pesquisa vai retornar, ou se ele tiver alto risco, ele vais ser encaminhado, ele vai receber alguma coisa?

Referência 2 - 0,23% Cobertura

É que a.... preocupação é tão pouca com relação a isso. e a demanda tá tão grande, que eles vão querer dizer mais... se eles encontrarem uma forma de dizer pra alguém, de que alguém vai ajuda-los, ouvi-los, poder relatar pra alguém, eles automaticamente escreveriam, mas aí já seria a segunda etapa, do atendimento, né.

Referência 3 - 0,12% Cobertura

Porque... se ele tem alto risco de desenvolver depressão, ele tá... numa situação de emergência, ele vai precisar de alguém. Eu quero saber se ele vai saber quem procurar.

Referência 4 - 0,69% Cobertura

O que acontece é o seguinte. Então eu tenho a minha... o meu diagnóstico de alto risco de depressão. Mas aí conta muito com... a sorte de que esse indivíduo, nessa situação vá procurar outro recurso, que não o recurso originário (do que respondeu)... seria melhor em buscar, falar "olha, já respondi, eu já coloquei agora... Eu tenho esse risco e tal". Aí o quando dá o CVV, ah, eu tenho um alto risco? Tu entendeu? Eu já respondi, eu já coloquei e por aqui você pode entrevistar, ele mostra ti daqui. Aí vai para o CVV, aí vai pro outro, pro outro, vai acabar indo pra lista de espera... Pode ser uma tiro pela culatra, porque se a pessoa está em alto risco de depressão, já passou por tudo aquilo ali, já viu briga, já foi machucada... e... se eu responder, deu isso, eu vou lá, né... vou no CVV, ah, eu respondi uma questão aqui, deu alto risco.... não sei como o CVV vai... Existem altas dificuldades em manter o CVV inclusive. Então é isso. É muito emergentes essa situação.

<Arquivos\\ > - § 3 referências codificadas [2,08% Cobertura]

Referência 1 - 0,69% Cobertura

E eles praticamente não vão lá em outros horários, especialmente os que têm risco. E tem tanta coisa pra tirar as pessoas da aula... que eu acho que os adultos estão se tornando extremamente pão duros com isso, com tirar da aula. É claro eu acho que uma coisa assim é mais importante do que muitas outras, mas não é a mensagem que as redes dão. As estão mais preocupadas com as provas que vão sair na Folha de São Paulo do que em tirar o aluno para preencher uma coisa que pode envolver um risco de vida. Quer dizer, a preocupação com as próprias crianças, por incrível que pareça, oficial não é tão grande assim.

Referência 2 - 0,95% Cobertura

Ah, eu acho que são muitos. Acho que se tem... tem alguma condição de encaminhamento, e existe.... esse caso que eu te narrei, narrei por isso. A [nome da universidade] tem condições bem precárias de atender. Mas elas existem. E aí porque isso não é detectado, muitas vezes os usuários não têm acesso. Então esse menino teve sua situação resolvida ou encaminhada. Não sei se resolvida porque eu não fiquei lá, mas ele teve a situação dele encaminhada graças a uma detecção. Então se a gente tivesse uma coisa sistemática seria super legal. A [nome da universidade] tem mais condições... tem pouca gente de 17 anos, como é risco futuro, né, mas estou te dando assim como um exemplo. Eu acho que qualquer recurso que exista, é inadmissível que não esteja sendo usado quando a gente está lidando com uma coisa tão séria. E eu acho que é o caso. Então acho que só por isso já valeria.

Referência 3 - 0,44% Cobertura

Nenhuma, eu acho que se a gente está lidando com um adolescente que de fato tem risco de depressão, ele já está estigmatizado, porque os sintomas já vão ser suficientes para essa nossa vida social terrível produzir os estigmas. Então eu não acho que ia assim cutucar as pessoas e torná-las hostis. Se elas tão pra ser hostis, elas já se tornaram porque isso apareceu de uma maneira ou outra.

<Arquivos\\ > - § 4 referências codificadas [2,29% Cobertura]

Referência 1 - 0,50% Cobertura

Eu acredito que teria que ter algum acompanhamento. Por exemplo, o adolescente faz esse teste agora, no primeiro ano do ensino médio, e alguém que acompanhasse essa caminhada, tivesse esse contato, acompanhasse, pelo menos por um ano. Ou nos próximos dois anos. Que ele chegasse lá no terceiro ano, no primeiro trimestre, ou no final do segundo ou terceiro, tivesse uma revisão, um olhar de algum adulto, algum técnico, um serviço de SOE, alguma coisa assim.

Referência 2 - 1,14% Cobertura

Pode até ser usada na escola se tiver alguém preparado lá e orientado, que pode ser o serviço de SOE. E pode inclusive ter professores, independente da área, mas que tenham talvez alguma habilidade para tratar essa situação, que possam até colaborar auxiliar, ajudar, participar de alguma organização, para que aconteça esse teste. Os dados vão para o computador, e aí a pessoa recebe esse texto para ler, e aí no final depende de algum... Ah, então entra em contato com a pessoa X, de referência, ou telefone lugar tal, entendeu... pra que a pessoa tivesse onde, né, agora estou me sentindo mal porque eu li esse texto e fiz a entrevista. Quem eu procuro. Então tivesse esse contato. Eu acredito também que as escolas que têm algum trabalho nesse sentido de depressão, também tragam outros momentos, outros eventos, onde a alegria, a descontração, o envolvimento, seja presente. A gente tem que trabalhar o lado também positivo. Senão a gente acaba criando, a gente cria a própria realidade. Ah, a depressão, então daqui a pouco eu estou deprimido.

Referência 3 - 0,25% Cobertura

Não tenho assim uma ideia de como poderia ser feito. Esse desafio poderia ser alguma coisa que a pessoa chegasse a identificar alguém que está, e aí ganhasse um presente, ou algum um prêmio, algum um bonequinho, alguma lembrança.

Referência 4 - 0,41% Cobertura

É uma forma mais rápida, prática, individual, é pessoal, a resposta, pra pessoa identificar e se dar conta e buscar ajuda. Que de forma geral, não vai acontecer. Acho que muito vai dar ideia, porque vai identificar a pessoa, a pessoa, ah, eu tenho esses caminhos, posso buscar ajuda, ou até mesmo, ah, eu acho que eu estou deprimido, mas vai lá e conversa e não é depressão.

<Arquivos\\ > - § 6 referências codificadas [2,47% Cobertura]

Referência 1 - 0,58% Cobertura

Olha, hoje em dia esse assunto não é mais tão tabu como era antes. Para os adolescentes..., é, ainda é, muitas famílias, os pais têm medo que os filhos vão ser discriminados por isso ou por aquilo, por tal característica, mas eu acredito que nossos adolescentes estão preparados, é um assunto, olha, é difícil passar um dia sem ouvir falar fulano tá com depressão, liga a TV, estão tratando lá de uma pesquisa sobre depressão. Acho que eles estão até mais familiarizados, pelo menos com o assunto.

Referência 2 - 0,11% Cobertura

Entrevistadora: Tu achas que eles não seriam rotulados?
Entrevistada: Não, acho que não. Eles poderão...

Referência 3 - 0,33% Cobertura

Sozinha, contigo, eles diriam. O problema é o grupo, os colegas saberem. Se um professor souber, se o orientador profissional souber, isso não é problema, pra maioria, acredito. O problema é os outros saberem, por que para o adolescente é muitíssimo importante o que os colegas acham

Referência 4 - 0,50% Cobertura

O que pode ser melhorado é aquilo que eu disse, sobre o grupo familiar, puxar mais, obter mais sobre a família, sobre quem mora contigo, como é tua relação com o padrasto, madrasta.... Acho que complementando isso, é uma ferramenta muito boa. Essa dinâmica, assim, ó, o adolescente, uma pessoa, o máximo que pode acontecer é ele sair daqui e dizer, ah, deu tudo errado, a mulher disse que eu tenho alto risco de depressão.

Referência 5 - 0,63% Cobertura

Não!!!! Não vão ficar. Porque eles já têm uma ideia sobre si. Se o questionário disser que ele tem baixo risco ou alto risco, se tem baixo risco, eles nem vão dar bola. Se tem alto risco, alguns vão pensar sobre o assunto, vão comentar em conta, na escola jamais, não vão comentar. Agora pode acontecer e pelo jeito debochado como eles encaram as coisas, vão sair e dizer, ah, aquela mulher disse que eu tenho alto risco de depressão, imagina eu deprimido, QUA, QUA, QUA! Às veze eles nem sabe que já está dentro da doença e não percebe.

Referência 6 - 0,32% Cobertura

Vocês têm alguém assim que esteja convivendo agora com adolescentes, seria interessante que essa pessoa participasse da reelaboração das perguntas, das questões ali, as perguntas, até pela questão do vocabulário, está bom assim, mas talvez alguma explicação das questões.

<Arquivos\\ > - § 1 referência codificada [0,11% Cobertura]

Referência 1 - 0,11% Cobertura

Entrevistado: Talvez sim, talvez não, né. Depende da ignorância desses pais.

<Arquivos\\ > - § 3 referências codificadas [1,32% Cobertura]

Referência 1 - 0,93% Cobertura

Então, assim ó... Então, é interessante que nós pudéssemos ter esse material em mãos também, até para ajudar a somar mais dentro do nosso trabalho na parte de conseguir ajudar esses adolescentes nas escolas. E quanto mais profissionais trabalhando nessa área, como SOE, assim, que é importante, tiver desenvolvendo nas escolas, vocês vão perceber que menos, menos adolescentes problemáticos vão sair daqui, sabe... Vão se formando, vão trocando de escola, até chegar na vida adulta. Mas eles vão melhorando se tiver um profissional junto acompanhando. Que que acontece: às vezes, tem famílias que não gostam de psicólogos nem de psiquiatras, eles consideram... Tem família ainda que têm aquela ideia, pais e mães que

eu já, que eu recebo na minha sala, até hoje, quando eu indico psicólogo ou psiquiatra, dizem "ah, mas o meu filho não é louco.", sabe... "Não vou levar, não, não vou levar, não precisa.", sabe? E não levam. E às vezes eu encaminho e não levam, sabe? E tu vê que o filho precisa. Então, tem uns tabus ainda, sabe, em algumas cabecinhas nas famílias. Sabe? Aqueles tabus antigos de que psiquiatras, psicólogos ainda é para louco. Então... Às vezes, por não ter acesso lá fora, o adolescente, nós fazemos o papel que eles precisam, que é necessário dentro da escola.

Referência 2 - 0,21% Cobertura

Entrevistado: Explicar o que é a depressão para ele entender, para ele saber, para no momento que vocês dizem assim ó, "A pesquisa demonstra que você... A pesquisa demonstra que os itens que você respondeu estão associados com o risco futuro de depressão". Tá, mas, e aí, o que é a depressão?

Referência 3 - 0,18% Cobertura

Se vocês explicam antes de passar isso aqui o que é depressão, fica mais fácil de ele entender. Agora, se bota um questionário direto desses, aí depois diz, "ah, você pode estar associado a uma depressão.", para ele, se ele não conhece...

<Arquivos\\ > - § 3 referências codificadas [1,11% Cobertura]

Referência 1 - 0,48% Cobertura

Acho que o maior desafio na realidade são os adultos. Os adolescentes que eles enfrentariam, sem nenhum problema, mas eu acho que pior são os adultos, principalmente assim, na escola quem vai realmente estar disposto, se tivesse pra todo mundo, quem vai estar disposto a chamar esse aluno, buscar, alguns vão dizer, há, mas não tenho tempo pra fazer isso, vou mandar pro fulano, essa essa coisa que sempre um empurra pro outro, quando não quer assumir. Acho que os adultos seriammais problemáticos que os adolescentes.

Referência 2 - 0,29% Cobertura

Eu acho muito bom, porque realmente nós caminhamos pra cada vez mais adolescentes depressivos. Um contingente de pessoas que parece meio sem perspectiva, caminhando a esmo, então ter algo que faça olhar, realmente vê tem alguém que, se ele liga, ter alguém que possa auxiliar, possa ajudá-lo, eu acho interessante.

Referência 3 - 0,34% Cobertura

Não acredito. Talvez nessa questão sexual, talvez ali esse adolescente ao responder isso, se sofreu abuso, pensar ainda o que poderia trazer de problema para com família. Talvez isso. Mas não acredito que isso seja empecilho totalmente pra todos, pra usar. De repente se tivesse nessa questão aí um "prefiro não responder", sei lá, sabe como são adolescentes.

<Arquivos\\ > - § 5 referências codificadas [3,34% Cobertura]

Referência 1 - 0,88% Cobertura

Entrevistado: Eu acho que vai depender de como essa entrevista vai chegar pra ele. Eu penso que se ela, se antes tiver uma informação, aí que eu já não sei. Porque, claro, a gente sabe que tem uma diferença entre tu responder uma entrevista sem... ter nada ainda, né, nenhuma pré informação, e quando tem. Mas, talvez, se ele entender porque que isso tá sendo feito e ele conseguir ter essa compreensão, lá no final eu acho que é isso que ele deseja mesmo, "Ah, eu quero saber". Né, porque pode estar se questionando, quem sabe, só pensando sobre isso, aí li vai dizer que ele tem um alto risco, tá, então talvez eu tenha que me cuidar, tenha que ser um alerta.
Ou às vezes até para aqueles casos daqueles alunos que usam muito... essas características pra, pra... que talvez nem seja depressão mas que ele gosta de usar pra chamar, tá usando isso pra chamar atenção em casa. Claro, já o chamar atenção já faz parte, mas o adolescente precisa chamar atenção, certo, seja pelo bem ou pelo mal, né. Mal, assim, relativo. Bem também relativo. Mas ele precisa chamar atenção de alguma

maneira. Talvez ele veja, "Ah, mas é baixo risco", aí não sei também, ele pode pensar, "Ah, baixo risco, então eu não tô fazendo a coisa certa. Então eu vou piorar esse negócio aqui", né. Agora que tu falou... pois é... eu acho que ficou muito pronto isso, sabe, muito pronto, assim.

Referência 2 - 0,61% Cobertura

Simplesmente dar esse resultado e deixar pra ele, não dá. Isso não dá pra ser. Porque se não aí sim pode dar margem e aí ele desviar completamente esse resultado em benefício próprio, ou no contexto em que ele vive. Me parece que o problema é a forma como é, bem isso assim, muito técnico pra um assunto que é tão delicado, que existe, tem tantas nuances, que existe tanto diálogo. Eu não sei, né, claro, é a minha característica. Eu vejo tanto por esse lado, eu observo tanto nos, nos diálogos com a feição vai mudando, sabe. A maneira como tu vai falando. Então, isso pode se perder numa... e muitas vezes é, como eu te digo assim, é o jeito que aquele menino me olhou. Talvez por ali eu conseguisse fazer uma intervenção e conversar com ele, né, e levar pra um outro lado. Então, se tu simplesmente dá uma resposta, assim, tão objetiva, eu penso que aí pode ir por água abaixo mesmo. Acho que tá é na forma como o resultado tá apresentado mesmo.

Referência 3 - 0,53% Cobertura

Entrevistado: É, eu acho que sim. Eu não gostei, entende. Eu, [nome da entrevistada], claro, aí, né, não gostei. Sabe o que que parece? Agora quase não existe, mas também existe no Face [Facebook]. Mas aquela, aqueles questionários que tu respondia nas revistas e aí no final te dava assim: ah, se você marcou tantas, tantos As é porque você é assim, tantos Bs... e aí eu li aquilo, eu disse, "Tá, mas eu não tô nem nisso e nem naquilo". Sabe? Daqui um pouco eu não tô nem no alto e nem no baixo, tô num intermediário. E não, não... eu não me identifiquei ali, nessa resposta. Bah, mas se eu não, bah, mas eu leio isso aqui, não tô aqui, não, mas também não tô aqui. Eu tô aonde? Aí pode confundir mais, pode atrapalhar mais, "Ah, não, isso é muito chato".

Referência 4 - 0,47% Cobertura

Entrevistado: Isso. Isso. Um outro escolhe, talvez ter um nível intermediário. Talvez ter 3, 5 não sei. Entendeu? E na hora de dar o resultado... poder conversar, talvez, aí, é que é tudo tão difícil, mas... se fosse eu - eu não sou psicóloga, não sou nada disso, enfim. Eu digo que eu sou psicóloga de novela, que olhar o outro é tão bom. Aí eu diria assim: o que que tu acha que deu no teu escore? Sabe, primeiro questionar. Ó, disso aqui ó, se eu te apresentar essas cinco possibilidades, onde é que tu acha que tu te incluiria? Seria tão bom se ele se identificasse naquele que é exatamente o que o questionário apontou. Muito mais fácil de dialogar, "Pois é, foi exatamente aquilo. Tu tá te enxergando assim, dessa forma..."

Referência 5 - 0,84% Cobertura

Ah, sim. É. Principalmente essa parte final. Talvez ali, né, no questionário em si, não. Mas na hora do resultado, que é o que interessa, que é pra isso que o questionário existe, né, pra dar um resultado, um escore que, que... né, o indivíduo, né, o sujeito possa fazer alguma coisa pra, pra melhorar, né, enfim, pra, pra... pra reverter isso, eu penso que sim. Porque é um assunto muito delicado, sabe, é muito difícil. Eu não tenho histórico de depressão, mas eu tive depressão pós-parto e foi muito difícil. E eu, e olha que eu dei conta, que eu fiquei ali com meu filho, eu não deixei de amamentar, mas aquilo foi sofrido. Então eu imagino que vive isso... Eu acompanho, a minha mãe já teve, claro, a gente sempre tem alguém próximo, mas eu tô falando da experiência minha, eu imagino quem vive aquilo. Eu me senti um ET fora do planeta. Que eu tinha que alimentar aquela criança, isso eu sabia, mas o resto eu não sabia. Imagina alguém que vive isso o tempo inteiro. Né, fora desse mundo, onde é que eu tô, que que eu faço, qual é o meu lugar. Aí dá um cartãozinho e procura essa ajuda, vai lá no site, telefona pra esse lugar que às vezes não atende, tu sabe bem como é que é. Então, eu penso que o final sim, talvez tivesse quer ser mais... mais relacional.

HEALTH WORKERS (PS)

<Arquivos\\ > - § 3 referências codificadas [0,72% Cobertura]

Referência 1 - 0,20% Cobertura

What would be the benefits? Early diagnosis, then it would be easier to revert those symptoms and treat them as soon as possible.

Referência 2 - 0,24% Cobertura

If the tool was used now, I believe yes. If we were able to implement public programs related to this and start taking this theme more seriously, then no.

Referência 3 - 0,27% Cobertura

If schoolteachers were trained to use the tool, could it risk creating negative bias in them towards vulnerable students?   - If they were trained, I don't think so.

<Arquivos\\ > - § 3 referências codificadas [2,80% Cobertura]

Referência 1 - 0,56% Cobertura

É. Mas que buscassem, sei lá, o serviço médico de uma forma geral... Ou que buscasse o grupo da igreja de uma forma geral... Acho que aí já tem uma possibilidade de se entrar de uma forma mais, com pessoas mais preparadas para responder mesmo que elas não estejam em risco. Mesmo que elas sejam de baixo risco, vamos dizer. Mas que estão um pouco mais com a cabeça... Ou com uma possibilidade do tema ser introduzido de uma forma mais...

Referência 2 - 0,58% Cobertura

Não, preparado para o instrumento, eu quero dizer. Eu quero dizer assim não que tenha que ter uma grande preparação, mas eu acho, assim, um adolescente por exemplo que vai lá todo sábado se reunir com a comunidade religiosa... Acho que tem alguma possibilidade maior já de compreensão, de entendimento, de levar a sério o resultado. É. O que me preocupa é eu responder e não levar isso... Se bem que qualquer um pode fazer a interpretação que quiser, né.

Referência 3 - 1,67% Cobertura

Aí, eu... Eu sou muito, assim, de pensar que a informação é sempre boa. No sentido de que as pessoas têm que se confrontar com a verdade. Tipo assim, às vezes, em outras, outras pesquisas, trabalhos que eu participei, grupos que queriam atuar em escolas, por exemplo, vinha sempre uma ideia "a gente não pode falar em transtorno mental. A gente não pode falar em saúde mental, a gente não pode usar o nome..." Eu nunca gostei disso, assim. Sempre achei que a gente tem que usar os nomes e que os preconceitos têm que ir caindo. Claro que vai ter, vai ter gente que vai ficar assustado com o resultado. Acho que vai ter, tem a possibilidade de ter um adolescente que se surpreenda. "Ah, mas estão dizendo, então, que eu não sou normal?", por exemplo. Acho que existem aqueles que já estão em sofrimento e se aliviam porque poder ver "tem alguma coisa que explica isso que eu tô sentindo, isso que eu tô passando etc." E tem aqueles que não vão dar nenhuma bola, acham que tudo é possível, mas eu sempre acho que a informação verdadeira, vamos dizer... Verdadeira que eu quero dizer não é que está 100% correta, né, mas que a informação, ela tem que ser transmitida, assim. Se a pessoa se dispôs a preencher, né, sabendo que no final vai ter essa possibilidade de ter um escore ou outro, um risco ou outro...

<Arquivos\\ > - § 8 referências codificadas [3,10% Cobertura]

Referência 1 - 0,23% Cobertura

Ah, entendi. Não, eu não acho que... que seja ruim que ele saiba que ele tem alto risco ou baixo risco, eu só acho... daí... "A pesquisa tem mostrado que tá associada ao risco futuro de depressão". São uma das questões

Referência 2 - 0,04% Cobertura

É um risco futuro. Avalia risco, né.

Referência 3 - 0,05% Cobertura

Eu acho que é uma questão bem preventiva, né.

Referência 4 - 0,16% Cobertura

E eu fico pensando se desse um alto risco, eu acho, assim... não seria de daí... de ser, daí, comunicado pros pais? Já que existe um risco detectado.

Referência 5 - 0,43% Cobertura

Entrevistado: Porque se eu detecto que aquela criança tem risco de...
Moderadora: Tem um alto risco... Sim.
Entrevistado: Eu acho, eu não deixaria isso com um adolescente tomar a iniciativa, assim, né. 4
Moderadora: Sim, eu veria também, tu apontaria...
Entrevistado: Eu apontaria ou pra escola...
Moderadora: Ou envolvimento familiar.
Entrevistado: Ou chamaria, é.

Referência 6 - 0,18% Cobertura

Porque eu não tô dando um diagnóstico, mas eu indicaria, assim, olha, estamos indicando que seja feita uma avaliação, mas aí fica a responsabilidade com a família, tá.

Referência 7 - 1,37% Cobertura

Sim, porque o risco da depressão, vamos dizer assim, a gente pode tá levantando várias questões aí, que são vistas no questionário, que pra mim já indicariam questões preventivas de depressão, né. Ali, ó: violência familiar, conflitos familiares. Pra mim, se eles já buscassem, se marcar que tem risco, pra mim já seria indicação pra ir pra um tratamento na UBS, pelo menos conversar com o médico. Isso para mim já seria sinalizador, assim, da pessoa e a família fica, "opa, meu filho tá se sentindo mal com essas brigas". Isso já está afetando a saúde mental dele, não quer dizer que ele esteja com uma depressão, mas opa, né. Opa, que legal, né. Acho que é porque as famílias não têm a menor noção, que nem isso, dorme todo mundo, nem sabe que isso faz mal pra eles, entende? A gente briga na frente, a gente fala das finanças tudo na frente das crianças. Às vezes a gente diz assim, "Gente, não discutam as finanças porque as crianças ficam mal". É coisas tão básicas que a gente fala às vezes pros pais que eles "ah, tá, tudo bem". Que a criança depois fica deprimida porque, assim, meu pai não tem dinheiro, a minha mãe não sei, eles ficam deprimido porque os pais tão com problemas financeiros. Mas a criança não tem que saber disso. Entende? Então às vezes só tu dizer "olha"...

Referência 8 - 0,64% Cobertura

Não, eu acho que é isso, assim. Eu acho que seria bem legal essas campanhas na mídia, eu acho que esse questionário é muito legal também. Acho que avalia bem, assim, né, esse risco. Só que, detectado o risco, como eu disse, acho que é legal depois que possa se encaminhar pra se conversar na escola sobre isso ou na, numa... numa atenção básica, em algum espaço, né, pra orientação dessa família. Não quer dizer que tenham que fazer tratamento. E que ele tenha, que essa tenha mais espaço saudáveis, né, que não seja só se conversar na rede, que eles acabam, né, se conversando só pela rede social.

<Arquivos\\ > - § 3 referências codificadas [3,04% Cobertura]

Referência 1 - 1,38% Cobertura

É, a primeira frase, ainda mais sendo a primeira, né, "a pesquisa tem demonstrado que os itens que você respondeu estão associados com risco futuro de depressão". Depende de quem lê, não sei se seria o próprio respondente ou a própria responder, ela é uma questão... que pode assustar e que pode ser uma...

antecipação. "Se alguma das questões que você respondeu positivamente estão causando sofrimento, é possível procurar ajuda." Acho que isso sim. "Você pode conversar sobre isso com um adulto em que você confia, um professor na..." Professor, professora, que em geral são a maioria, as professoras, né, "na escola ou um", uma, "professional da saúde". "Você também pode contatar..." Certo... Tá, então, os contatos, o CVV... "Para saber mais sobre prevenção visite..." Acho que, é, visitar sozinhos um portal pode ser também, né, assim, bom, um adolescente que chegou lá, né, no seus 14 anos vai abrir o portal e vai enxergar uma série de coisas que pode remeter justamente, associados a um risco futuro de depressão, então... A informação é fundamental, mas acho que ela precisa de um acompanhamento, né. Tá, depois eu vou... voltar aqui só pra ver "Baixo risco. A pesquisa indicou que você apresenta..." É, aqui, esse site eu não conheço, então, né... Acho que... ele trata... "aprender mais sobre sono, dieta e comportamentos saudáveis". Sim, essa ideia né, de "você conhece alguém... fatores de risco... encoraje..." Olha aqui, tá, ó, "encoraje ele ou ela"...

Referência 2 - 1,09% Cobertura

Acompanhamento, assim, pra poder dialogar, porque ele pode se assustar dependendo de como ele realmente se encontra em termos de risco, ele pode se assustar com a informação. A informação, ela precisa ser livre, acessível, né, completa pra todo mundo. Mas em se tratando dessa faixa etária, ter companhia é fundamental pra poder falar sobre isso, né, pra acompanhar, pra situar, "Olha". E se a gente remete, remete, né, imediatamente já com enfoque na doença - eu acho que essa é uma grande questão, né, o nosso enfoque em geral tá sendo a doença, né. A gente vem direcionando - claro que é o que nos interessa, a gente quer cuidar disso - mas o quanto tá difícil pensar nas potencialidades, naquilo que a adolescente ou o adolescente podem e do quanto a gente não vai ficar sabendo, assim, do que ele mais gosta, do que ele sente falta, de quantos espaços ele poderia imaginar e que a gente nunca vai disponibilizar porque ele, não ouvimos o que elas e eles têm a dizer e porque não nos ocorreu. Sei lá, se tiver uma pracinha, uma praça muito legal no território, esse pode ser um espaço muito importante. Mas, né, tô dizendo uma coisa, assim, parece muito simples.

Referência 3 - 0,57% Cobertura

Sim, acho bem importante, se eu puder, né, retornar aqui um pouquinho... "Você pode contatar..." Voltando aqui ao alto risco, né, que é o que nos preocupa. Mas tem que nos preocupar da mesma forma o que se oferece pra uma adolescência... adolescência é um negócio pra todos... com saúde, né. Então, botar assim: políticas públicas com adolescentes e que a gente pense na saúde, né, com saúde. Mas aqui, "pra saber mais sobre sintomas", a gente... agora incluindo aqui políticas públicas, a gente precisa, em primeiro lugar, digamos, oferecer algo que é de direito, que o SUS... pelo menos, né... previsto em lei.

<Arquivos\\ > - § 3 referências codificadas [0,53% Cobertura]

Referência 1 - 0,32% Cobertura

Em um primeiro momento eu ia sugerir as escolas, porque acho que é onde eles estao o tempo todo. Tem que ter um cuidado pra nao supervalorizar os resultados dela, teria que ter o ponto de vista crítico. Deixar bem claro que aquilo seria uma triagem, que nao é nada que defina o diagnostico sozinho, que é só uma suspeita. Já tem aplicativos pra celular que fazem esse tipo de coisa. Os adolescentes costumam gostar também.

Referência 2 - 0,09% Cobertura

Sim, a pessoa achar que é capaz de diagnosticar e tratar só com isso. Sem ter alguma formação específica pra isso.

Referência 3 - 0,12% Cobertura

Ajudar a detectar mais gente a alertar mais os profissionais em geral que poderiam ajudar esses adolescentes que talvez seja mais frequente que eles imaginam.

<Arquivos\\ > - § 5 referências codificadas [2,26% Cobertura]

Referência 1 - 0,46% Cobertura

Mas, assim, as perguntas são pertinentes, acho que é legal e interessante. Acho que teria que ter um pouco mais de consequência para o alto risco. Acho que só o CVV... Por quê? Porque tanto o CVV quanto o portal saúde, eles exigiriam uma iniciativa, e eu acho que não é suficiente. Uma menina de 14 anos que preencher, que dê alto risco, ela não vai procurar o CVV, ela não vai procurar nada, então, talvez algum tipo de alerta, né, combinado previamente. Então, tá, se der alto risco... Ou não, alguma coisa mais genérica do tipo, bom, aparecendo alto risco, isso será... Você será... Você terá algum tipo de acesso ajuda, e aí, a partir daí tem algum tipo de alerta para a unidade básica, alguma coisa, porque sozinhos não vão procurar.

Referência 2 - 0,64% Cobertura

Claro, a gente não pode tutelar tudo, mas como é adolescente, não adulto, deveria, né, até os 17 anos pelo menos deveria ter algum tipo de alerta para alguém. Se é numa escola, que um alerta ao diretor para que possa minimamente, enfim. Porque da iniciativa do adolescente com risco de... Claro, é um risco para o futuro que ele aponta, mas na verdade respondendo assim às perguntas de risco aponta praticamente uma depressão no momento, né. Eu não sei também, talvez seja importante, esse futuro o quão vago ele é, porque se um adolescente de 14 preencher que já tomou bebida alcoólica, que já, que já, que já foi criado por outra pessoa por um tempo... Algumas perguntas ali já apontariam para hoje ou para amanhã, entende? O futuro fica vago nesse sentido porque pode ser daqui a três anos, mas pode ser daqui a uma semana ou presente porque, dependendo de como elas forem preenchidas, já é uma depressão. Já tem fatores suficientes para uma depressão. Então, né, nesse sentido que futuro seria esse também, acho que teria que pensar.

Referência 3 - 0,72% Cobertura

Unidade básica. Acho que como ajuda... Assim, hoje em dia o portal SUS, né, o sistema de telessaúde, ele já tem, por exemplo, os enfermeiros já têm acesso aos SNAP, que é aquela escala já autorizada para avaliar déficit de atenção e hiperatividade e oposição, que se entrega, o próprio enfermeiro ou o pediatra imprime, entrega pra família e a família entrega um na escola, um para a mãe, um para a avó e as pessoas preenchem a escala e aquela escala é necessária para o encaminhamento para um neuropediatra. Então, assim, isso é focado, o lugar disso no sistema de saúde seria a unidade básica. Daí, talvez, com essa adaptação desse futuro mais próximo, assim, né, e daí não poderia ser anônimo, né, nesse sentido. A não ser que tenha só uma orientação ali, se for para um uso do sistema de saúde, precisa ser, não, essa... Essa menina está, eu estou aqui com a Fulana, ela vai preencher, deu risco pra depressão, eu vou encaminhar ao pediatra para uma avaliação melhor ou eu vou fazer uma entrevista de saúde mental e vou ver se já mando ou não para atendimento. Aí, não pode ser para fins de pesquisa anônima, né, tem que ser já para educação e prevenção.

Referência 4 - 0,24% Cobertura

Não. A escola poderia ter, mas, assim, a escola, daria uma responsabilidade à escola que eu não sei o que eles fariam com isso, né, porque como eu te disse, acho que o mais importante da escala, do questionário é que a consequência do risco futuro, nenhum adolescente deve conseguir seguir se ele já está deprimido, né, que é procurar o CVV ou procurar o portal para ter informação.

Referência 5 - 0,20% Cobertura

Ele acabaria criando demanda de atendimento, que seria um desafio atender, mas eu acho que isso não é um desafio pra vocês, assim. Acho que é um desafio para os gestores e acho um bom desafio, né, tem que ter. Não adianta a gente não saber e daí, então, não atender, né. Acho que principalmente pelo caráter preventivo mesmo.

<Arquivos\\ > - § 1 referência codificada [0,85% Cobertura]

Referência 1 - 0,85% Cobertura

Não, eu acho que essa ferramenta, eu achei bem interessante a ferramenta, assim, para estar dizendo assim, o que que pode, ou se tu podes ter alguns sinais. Talvez, mostrando isso para os alunos, para os adolescentes - olha, não quer dizer que tu vais, mas tu podes, tu tá propenso a desenvolver... Que estratégias também ele pode estar utilizando para melhorar isso, no final, entendeu? E não só dizendo, olha, tu tens risco. Tá, e daí, o que eu faço com isso? Entendeu? Então, acho importante essa questão. Tu dás estratégias, dá algo que ele possa estar utilizando de ferramentas para prevenir que isso ocorra. Não adianta dizer “tu tens risco” – ah, que bom que eu tenho risco, eu faço o que com isso? Entendeu? Então, talvez, na própria depois no fechamento, ter essa estratégia de melhoria, facilitadora, digamos assim. Acho que isso ia ajudar.

<Arquivos\\ > - § 5 referências codificadas [1,26% Cobertura]

Referência 1 - 0,60% Cobertura

Entrevistado: É, acho que sim. Porque eu acho que a, a... a depressão é a doença do momento, né. Ela é silenciosa, porque, ok, o câncer mata, mata mais ainda, na atualidade ainda é o câncer que mais mata, né. Mas, mas a depressão tá muito perto, né. Tá muito perto e as pessoas, no geral, parece que tem um preconceito maior com questões mentais, né. Então procura recursos mais tarde. Só vai procurar quando realmente aperta.
Moderadora: Como se fosse aquelas barreiras que a gente estava falando na pergunta anterior, né, o preconceito seria uma barreira.

Referência 2 - 0,33% Cobertura

Entrevistado: Exatamente. Trabalhando na introdução da calculadora, colocando... o que que se, se, se quer com relação à saúde mental, quem tá exposto, como se pode... que hoje em dia qualquer um pode estar exposto. As pessoas sabendo o que é uma depressão, da onde que vem, já para entender melhor as questões, já vai atenuar a resposta lá atrás.

Referência 3 - 0,07% Cobertura

Entrevistado: Tem mais outra coisa. Tem grupo de escoteiros, grupo de... sabe?

Referência 4 - 0,14% Cobertura

Entrevistado: Tem jovens que fazem grupos até de grupos de, de religião, de igreja, que fazem grupo de jovens. Por que não introduzir esse assunto?

Referência 5 - 0,12% Cobertura

Entrevistado: Onde tiver um núcleo...
Moderadora: Existe uma possibilidade de trabalhar questão de saúde mental.

<Arquivos\\ > - § 3 referências codificadas [0,25% Cobertura]

Referência 1 - 0,07% Cobertura

Entrevistado: Não, é super viável. Só não sei se tu... Vocês acham que avalia depressão? O risco, baixo risco, alto risco?

Referência 2 - 0,09% Cobertura

Entrevistado: Ai, poderia ser até nas escolas, né. Acho que seria muito show. Poderia usar associado aos meus trabalhos de bullying, poderia ser bem legal.

Referência 3 - 0,09% Cobertura

Entrevistado: Será que isso não vai ser? Porque eu não acho que os jovens vão se negar a responder. O que pode acontecer é a resistência das escolas e dos pais.

<Arquivos\\ > - § 2 referências codificadas [1,66% Cobertura]

Referência 1 - 0,46% Cobertura

Tem que achar as redes em que eles estão agora, né. Tu tem esse problema deles responderem qualquer coisa ou só zoarem mesmo, assim, responderem tudo ruim só para... Né. Só para... Porque o comportamento do adolescente pode ser isso, né, essa coisa meio de (?), ou vamos juntar uma galera e fazer aqui só para gozar. Então, não sei se tu vai ter tão fidedigno assim. E aí, mascara quem realmente está...

Referência 2 - 1,20% Cobertura

É que tu não vais usar na pesquisa, se tu tens um trabalho clínico, já vai ser direcionado para... Eu não sei, assim, que outro espaço tu vais usar isso, entendeu? Porque o próprio adolescente pode saber, assim, se ele está com depressão, eu acho que isso aqui não vai responder. Porque só porque usou, experimentou maconha, isso não... Não quer dizer que esteja em depressão ou a questão também se os pais são separados, não quer dizer que vá... Então, assim, se tu vai usar para ele identificar que ele tem um alto risco, isso aqui pode mais, é, talvez despertar outras questões e não a depressão em si, porque, na verdade, está falando mais de comportamentos sociais do que o que ele está sentindo, eu senti isso, né. Ah, tu se sentes triste... Tu falou que vai usar outros instrumentos, mas, se o adolescente pega só isso aqui, ele não responde. Quantas vezes por mês você ficou triste? Quantas vezes... Sabe? São coisas que, ele é mais um instrumento social, social, respostas sociais do que efetivamente do comportamento depressivo.

<Arquivos\\ > - § 5 referências codificadas [5,28% Cobertura]

Referência 1 - 0,99% Cobertura

Entrevistado: E aí já vem a questão do uso. Eu não vejo hoje como a gente poderia aplicar esse escore no mundo real ainda. Ele é um primeiro passo numa série de passos que teriam que ser tomados nessa avenida de prevenção de risco, como em outras áreas da medicina. O que tem que acontecer antes: as pesquisas têm que ser desenvolvidas usando esse escore para, identificando as pessoas de risco, vê quais as intervenções que fazem a diferença ou não. Porque a gente sabe que cada um vai ter uma solução para prevenir depressão - pode ser exercício físico, pode ser mais contato social, pode ser questões religiosas... Cada um tem a sua teoria, a gente não pode aplicar todas porque o adolescente não tem todo o tempo do mundo, mas ao mesmo tempo, talvez tenha uma que valha mais a pena - ou algumas - que outras. Isso a gente só vai saber depois de fazer um estudo clínico.

Referência 2 - 0,97% Cobertura

Entrevistado: Então não é que o escore não tenha uso nenhum, mas ele é um primeiro passo. Depois um estudo clínico vai poder usar o escore para separar as pessoas de alto risco das de alto risco. Porque até então, sem o escore, a gente tem estudos tentando fazer estratégias preventivas, mas eles simplesmente não conseguem encontrar um efeito provavelmente porque eles pegam todo mundo - quem tinha baixo risco também, e acaba tendo muita dificuldade de encontrar um efeito por causa disso. E isso é o que se viu em outras áreas da medicina, o Framingham... Primeiro se desenvolveu o Framingham, que é um instrumento de escore de risco para evento cardiovascular, infarto, AVC, depois se fez ensaios clínicos com aspirina, outras medicações, para prevenir esses eventos cardiovasculares.

Referência 3 - 0,40% Cobertura

Entrevistado: Retomando, eu não vejo, hoje, uma aplicabilidade prática que a gente possa fazer fora do âmbito de pesquisa nesses locais que eu estou dizendo, atualmente eu ficaria confuso se eu fosse um médico assistente talvez eu pudesse fazer uso com muita cautela, mas a nível mais informativo, se eu fosse na ponta, se eu fosse um médico da UBS.

Referência 4 - 1,30% Cobertura

Entrevistado: Ao mesmo tempo, é uma questão de realidade prática que no momento, pelo menos no Brasil, mas acredito que no mundo, a gente não tem – tem uma falta muito grande de profissionais de saúde mental, que dirá no nosso país, então a gente mal e mal consegue identificar casos já em depressão, já com a síndrome, e fazer o tratamento adequado. Falta medicação, falta psicoterapia, aos montes, e falta identificação de casos, falta olhar para os casos que a gente já tem. Então é muito difícil a gente conseguir – claro que prevenção segue sendo importante, porque possivelmente diminui essa sobrecarga, mas ao mesmo tempo, em termos de Brasil, é muito difícil a gente conseguir imaginar ainda. Acho que isso está mais para questões de políticas públicas do que para questão do médico assistente fazer esse trabalho, porque talvez o trabalho hoje do médico de família, do médico da ponta, seja tratar os casos – e isso já é difícil – e o trabalho das políticas públicas seja diminuir o número de casos que vão aparecer através de uma estratégia preventiva como a gente está propondo, usando o escore, enfim, que ainda não tem essa questão.

Referência 5 – 1,61% Cobertura

Entrevistado: As questões éticas eu acho sempre uma preocupação muito grande. É muito difícil para o ser humano em geral trabalhar com probabilidade, a gente não é bom probabilista em geral, e a gente... Quando a gente fala que alguém tem alto risco a gente tem que sempre lembrar que, primeiro, a gente pode estar muito bem errado, segundo, a gente te que cuidar com questões de profecias autorrealizáveis, né? Ah, então tu tem um alto risco para ficar deprimido? Bom aí mesmo que fica. Então a gente tem que avaliar se isso não acontece, e qual é o efeito – porque talvez a própria, a gente aplica o escore, a gente faz uma classificação, mas será que isso não tem um efeito deletério? Então até mesmo talvez, assim como a medicação tem efeito colateral, classificar alguém como alto e baixo risco possa ter um efeito colateral. Então essas coisas são muito difíceis de avaliar, e a gente vai precisar de estudos clínicos. Na prática, não adianta, por enquanto a gente está em âmbitos ainda mais, de novo, nos primeiros pilares dessa avenida que a gente está construindo nesse sentido de prevenção. É uma avenida que é muito válida, ela tem um bom... Acho que ela é muito bem intencionada, mas a gente não pode esquecer das questões éticas que estão implicadas em qualquer intervenção de saúde.

USERS (US): PARENTS

<Arquivos\\ > - § 1 referência codificada [0,31% Cobertura]

Referência 1 – 0,31% Cobertura

Calculadora de risco? Tipo um instrumento? Ah, acho que psicólogos, pediatras, psicanalistas... Profissionais da saúde. Seriam eles os mais habilitados.

<Arquivos\\ > - § 6 referências codificadas [1,51% Cobertura]

Referência 1 – 0,42% Cobertura

Eu achei uma boa forma de a gente constatar, né. Uma forma de ajuda para a gente ver se a pessoa tem ou não depressão. Porque são perguntas simples, mas bem diretas, né, e que a gente consegue, com as respostas, verificar realmente se a pessoa precisa de ajuda ou não. Bem legal, gostei.

Referência 2 – 0,50% Cobertura

Eu tenho terapeuta e psiquiatra, também. Então... Já passei muito por psicóloga, também, então, já conheço um pouco, assim, o meu histórico. No começo, é difícil a gente aceitar, né, que a gente tem o risco para depressão. Seria bom a gente não ter, né. Receber o baixo risco. Mas, se recebe o alto risco, a gente tem que aceitar ajuda.

Referência 3 – 0,15% Cobertura

Entrevistador: E tu vê esse instrumento como uma possibilidade de ajudar?
Entrevistado: De ajudar, achei.

Referência 4 - 0,05% Cobertura

Não. Não vi nenhuma dificuldade.

Referência 5 - 0,17% Cobertura

Acho que seria... Tentar me botar no lugar de um adolescente. Bem difícil tu receber um diagnóstico de alto risco.

Referência 6 - 0,22% Cobertura

Conversa. Acredito que seja conversar, conversar, conversar e conversar. Tentar esclarecer, explicar. Acho que isso ajuda bastante. Acho que isso.

<Arquivos\\ > - § 3 referências codificadas [1,91% Cobertura]

Referência 1 - 0,50% Cobertura

Ah, que que eu imagino, que às vezes falando não sai. Mas talvez a pessoa marcando aqui, é uma forma de ela se comunicar, de ela fazer sei lá, vou fazer chegar em alguém, o meu sentimento, a minha angústia. Acho que pode ser por aí.

Referência 2 - 0,76% Cobertura

Eu acho que seria o ideal, mas não sei se surtiria efeito. Não sei se não, se isso não faria talvez eles... Não digo mentirem, mas trocarem a resposta, entendeu. Por estar com o nome identificado. Talvez não identificando, eles seriam mais verdadeiros nas respostas. Mas aí também, tu fica naquela de como é que eu identifico agora quem foi para mim poder sanar o problema.

Referência 3 - 0,65% Cobertura

É, o que eu vou colocar aqui é praticamente... Essa parte de procurar um adulto que confiem, eu ia dizer para eles assim, que de todo mundo, pode confiar em muitas pessoas, mas com certeza pode confiar em pai e mãe, isso que eu diria, sabe? Em pai e mãe tu confia, busque ajuda com eles que com certeza vai encontrar.

<Arquivos\\ > - § 5 referências codificadas [3,02% Cobertura]

Referência 1 - 0,47% Cobertura

Acho que sim, eu achei importante, assim. É um alerta, né. É um alerta. Porque vai assinar realmente o que sente, né, é uma coisa, assim, que tu tem que estar bem pensando, assim, como se diz para... Para ti ver direitinho, se conhecer, né, se autoconhecer. Então, se tu tá bem, tu tá bem, né. Se tu não tá, tu tem que procurar alguma ajuda, né.

Referência 2 - 0,79% Cobertura

Porque as crianças, ultimamente, elas não são abertas, né. A maioria se... Eu sei por mim, né, no caso. A gente se guarda, então, hoje em dia, como eu aprendi bastante a botar para fora, falar o que você está sentindo na hora, até com briga com meu marido, coisa assim, com a minha irmã, quando eu não gosto de alguma coisa, eu falo "ó, Fulana, não gostei disso, tem como a gente resolver?" Os adolescentes, eles não têm essa entrada, então o questionário, assim, é ótimo. É uma autoavaliação já de começo aí, a pessoa já vê, já... Bom, um alerta, né. Já vamos chamar para conversar.

Referência 3 - 0,85% Cobertura

Eu acho que eu chamaria, no caso, chamaria os pais. Primeiro, o aluno. Depois, chamaria os pais. Para ver a primeira fala, como se diz, né. O adolescente falar a respeito, o que é que está acontecendo, até a questão

de colégio... E os pais para, tipo, dar um auxílio e ajudar, né, os adolescentes, o adolescente que tiver se sentindo, porque, de vez em quando, a gente não tá. Na correria do dia-a-dia, a gente não dá importância pros filhos, alguma atitude que toma, né, "ah, está lá no quarto.", né? "Ah, tá lá no quarto, está estudando, está fazendo qualquer coisa." Tu não tá lá olhando. Tem que chamar. Tu tem que chamar para...

Referência 4 - 0,42% Cobertura

Para reconhecer, para ver, "ó, Fulano, tu tem isso, tu tem aquilo, me ajuda a fazer isso, me ajuda..." Então, tu tem que conversar. O adolescente é uma coisa que tu tem que falar 20 vezes para ele te escutar uma vez. E é isso que eu to aprendendo, né. Então, é importante isso, eu acho que é muito importante...

Referência 5 - 0,49% Cobertura

Sim, é, bastante. Eu acho que vale a pena, teria que levar mesmo. Eu não... Eu falo pela situação que eu me encontro agora porque eu não imaginava, né, eu falo assim... Porque é uma pena, eu pensei durante o início do ano, eu pensei em ir no colégio, conversar. Talvez alguma coisa no colégio poderia me dar uma luz, né, "não, a gente vê a Nicole muito sozinha...".

<Arquivos\\ > - § 3 referências codificadas [1,96% Cobertura]

Referência 1 - 0,19% Cobertura

Para o questionário não é o desafio, o desafio é o interesse pelo tratamento.

Referência 2 - 0,73% Cobertura

O questionário é atrativo, interessante, as crianças vão querer responder, por ser tecnológico, não ser em papel, é virtual, o problema é onde apresenta o grau de risco, é procurar ajuda. Atualmente é muito mais constrangedor tu procurar ajuda e aceitar ela, do que continuar do jeito que está.

Referência 3 - 1,04% Cobertura

Eu vejo. Acho que ajudaria muito. Tem crianças que se encorajam com isso, e tem crianças mais tímidas. Eu levei dois anos para convencer que havia uma doença. Porque ele não aceitava a doença, era anormalidade. E eu dizia você não é o único, iguais a ti tem vários, cada um do seu jeito. Depressão é uma doença, tem tratamento e não é contagioso. É um estado físico e emocional simples, só aprender a conviver com isso.

<Arquivos\\ > - § 1 referência codificada [0,46% Cobertura]

Referência 1 - 0,46% Cobertura

Eu acho que sim, porque aí a gente vai tirar uma base do que eles estão passando, né. Pior tu ficar sem saber, eles não te falam, tu pergunta e não te respondem, aí tu não tem como fazer, não tem o que fazer. Não tem como agir. Pelo menos se disser, "não mãe, é alguém que tá me assediando", "não mãe, alguma coisa tá me..." Aí tu já pode tomar uma providência e reclamar, como eu digo pra eles, "não, vocês tem que falar porque aí a gente vai poder falar e não adianta..."

1. Code Query Output – Content of the Risk Calculator

POLICY MAKERS (FPP)

<Arquivos\\ > - § 2 referências codificadas [0,19% Cobertura]

Referência 1 - 0,13% Cobertura

Achei que está bem objetivo, é aquela ali mesmo, é ou não é, eles não têm que passear muito, sabe... Podem, sim, omitir a verdade, né. Mas se ele for verdadeiro, te repetindo, eu acho que a ficha cai e cai forte.

Referência 2 - 0,06% Cobertura

Eu não sei se não teria que colocar - porque ele fala só se já usou maconha, né.

<Arquivos\\ > - § 2 referências codificadas [1,18% Cobertura]

Referência 1 - 0,25% Cobertura

Aham. Não, e ele avalia questões de suporte familiar, questões de... Um pouco de autoestima, questão de rede de apoio... Aham. Sim, acho que a maioria, se marcar a maioria "sim"...

Referência 2 - 0,93% Cobertura

Eu acho, assim, vendo os pontos aqui abordados, eu acho que ele é amplo, que ele pega questões familiares, pega as questões escolares e outros aspectos relacionados ao abuso sexual, baixa autoestima, e eu acho que é interessante, inclusive para a autoaplicação, monitoramento, se for... E pensando, já que a gente tinha falado na própria questão dos agentes comunitários, eu acho... Depende, também, tem que ver qual é o ponto de corte, né. Para ver, para não ser algo que todo mundo cai aqui dentro ou que ninguém entre, né, então, vendo de acordo com um ponto de corte para identificar quem é de alto risco, eu acho que como screening, assim, como rastreamento, me parece válida essa ferramenta aqui.

<Arquivos\\ > - § 5 referências codificadas [1,93% Cobertura]

Referência 1 - 0,27% Cobertura

Talvez, aqui, onde fala dos relacionamentos, uma coisa a se ponderar fosse da criança que não teve contato com mãe, com pai, talvez tivesse que ter uma opção de "Não tive contato com mãe, não tive contato com pai". Especialmente com pai.

Referência 2 - 0,24% Cobertura

É, de pensar, porque eu acho que aqui pode encontrar uma dificuldade, "ah, mas eu não conheci meu pai." ou "Meu pai me abandonou quando era pequeno. Como é que eu me encaixo?", né, ruim? Ou era inexistente?

Referência 3 - 0,43% Cobertura

Entrevistado: Eu talvez pensaria em separar em dois itens, também, aqui a questão da (?). Claro que, talvez, para risco, tenha o mesmo valor, mas às vezes gera confusão quando a gente coloca duas questões juntas, né. "Já aconteceu de ter brigas na sua casa com agressão física entre adultos, ou de um adulto agredir uma criança?" Não sei o quanto é importante separar, mas talvez fosse uma ideia.

Referência 4 - 0,68% Cobertura

Entrevistado: Uma outra coisa que surge bastante dentro dessa última frase aqui das escolas, é a dificuldade com a questão do sono do adolescente em função dos eletrônicos justamente. Tu sabe que o adolescente tem uma tendência, uma dificuldade, por questões até fisiológicas de acordar cedo... Mas, especialmente em família, a gente percebe a dificuldade de estabelecer algumas regras, de ter algumas combinações. O sono

alterado ou a troca do dia pela noite é uma coisa muito comum. Acabou aparecendo aqui na orientação, mas não sei o quanto seria importante talvez aparecer no questionário alguma questão sobre sono e também...

Referência 5 - 0,31% Cobertura

É, e talvez, para o jovem, incluir aqui, além do CVV, a sua unidade básica de saúde como referência porque é onde geralmente se tem o primeiro contato com o sistema. Caso seja necessário encaminhar para um atendimento especializado ou mesmo já fortalecer esse vínculo, né.

<Arquivos\\ > - § 9 referências codificadas [4,24% Cobertura]

Referência 1 - 0,08% Cobertura

Entrevistado: Tá. Eu não usaria, acho que não deve se usar a palavra mulato, mulata, nunca, tá?

Referência 2 - 0,60% Cobertura

É uma questão da comunidade negra que compreende essa expressão como preconceituosa porque ela seria - nem sei se isso é verdadeiro -, mas ela seria um derivado de mula, que é o híbrido do cavalo e do burro. Não sei se é verdade, não sei se não tem outra origem, mas isso não importa, o fato é que a comunidade negra não gosta, então... Bom, no caso do IBGE, nós temos... O IBGE que usava a expressão negro, não preto, né, então, negro e pardo. Só que depois, quando vai se contar, quando vai se somar estatisticamente, se soma negros e pardos porque pardos são negros claros e negras são negros escuros, né. Então, só essa ideia para seguir os critérios do IBGE.

Referência 3 - 0,71% Cobertura

Experiências familiares... Relacionamento com a mãe... Aqui, para evitar também uma opressão, eu acho que "alguma vez você já foi separado de seus pais para ser cuidado por outra pessoa?" Essa ideia de "separado dos pais" só tem sentido se a gente estabelecer uma linha de tempo, um período, tempo, né? Porque, eu penso assim, alguém que ficou na casa da avó durante uma tarde, ela foi separada do pai e da mãe, mas foi uma tarde, mas foi uma separação, né. E a pessoa ou o adolescente pode se confundir aqui, um grande número de respostas "sim" quando, na verdade, não houve separação. Foi só um cuidado lá de alguém da família durante um turno, digamos, né. Eu não sei como fazer, mas eu colocaria uma pergunta de... Temporal, né, sei lá, pelo menos uma semana, um mês, lá, não sei.

Referência 4 - 0,64% Cobertura

É, essa aqui da 12, que é comida suficiente e vestir roupas sujas porque não tinha outras, eu separaria essas duas porque acho que são experiências totalmente diferentes e acho que a história de não ter comida suficiente parece tão mais grave do que não ter roupa, não sei. Acho que valeria a pena talvez separar. Fazer duas em vez de uma. Eu acho que a expressão, na número 18, a expressão "coisas sexuais" me parece muito aberto, também. Que que seriam coisas sexuais? Um beijo é uma coisa sexual. Um beijo contra a vontade, digamos, de um primo, é uma coisa. Mas intercurso sexual é outra coisa, é muito mais grave, muito mais... Né? Não sei. Talvez fosse o caso também de deixar mais explícito o que que se quer saber.

Referência 5 - 0,40% Cobertura

É, é. E se é adolescente que vai responder, acho que pode usar mesmo a expressão, eu usaria a expressão que eles usam, né, "alguém já tentou transar com você contra a sua vontade?". "Alguma vez você já tomou bebida de álcool"... A gente vai perguntar... A pergunta é sobre álcool e depois sobre maconha. Eu aproveitaria e perguntaria sobre mais algumas drogas, ou marcaria isso em aberto, uma questão de múltipla escolha, várias opções.

Referência 6 - 0,47% Cobertura

Mais opções: álcool, maconha, cocaína, crack. Valeria a pena ter um levantamento também de experiências com outras drogas, né, mas enfim. Risco para depressão... É. Por que não se incluiu uma pergunta sobre

ideação suicida claramente? Se a gente está falando de depressão, a ideação suicida acho que é uma característica importante. Não sei, eu colocaria uma pergunta, "já pensou em se matar alguma vez?". A gente sabe também, na pesquisa, que não faz nenhum mal perguntar; pelo contrário, até ajuda. Eu perguntaria.

Referência 7 - 0,56% Cobertura

Pois é, deixa só eu falar mais uma coisinha aqui. Eu acho que tem duas, tem três questões que adolescentes experimentam muito, especialmente adolescentes mais pobres, que é o preconceito social mesmo, da escola, dos colegas, da sociedade e da comunidade, enfim. Por conta da pobreza deles, que eu acho que é uma coisa diferente, uma coisa é... Essa pergunta, tá, quando pega aqui a história da comida, roupa, tá pegando o problema do carecimento material, legal, mas talvez pior do que carecimento material seja o fato da pessoa se sentir discriminada na sociedade por ser pobre. E vários desses adolescentes se sentem assim.

Referência 8 - 0,60% Cobertura

Eu acho. O tema, por exemplo, o tema do álcool e da droga, eu acho que vale como... Para se ter levantamento mesmo de incidência, coisa e tal. Mas é muito enganador, né, porque eu tenho, vai aparecer um monte de adolescentes que já usaram bebida alcóolica porque adolescentes já usaram bebida alcóolica. E tu vai ter aí 99% de usuários que nunca, não teriam depressão. E o mesmo vale para a maconha, né, ou para qualquer outra droga. Então, também, a opção da droga no escore eu acho meio complicado - a não ser que a gente separasse na droga o uso contínuo, o uso, tipo assim, o abuso da droga, entende? Então, já bebeu? Já. Já fumou? Já. Isso não me diz nada.

Referência 9 - 0,19% Cobertura

Agora, a frequência. O cara fuma maconha todos os dias, o cara bebe todo final de semana, toma trago... Bom, aí sim, acho que isso é fator de risco. Não sei se como está aí vai ser muito útil, sabe?

USERS – FOCAL GROUP WITH PARENTS

<Arquivos\\ > - § 6 referências codificadas [0,66% Cobertura]

Referência 1 - 0,01% Cobertura

Perguntas bem pertinentes.

Referência 2 - 0,13% Cobertura

Só queria chamar atenção pra uma coisa. São fases diferentes, né, são momentos diferentes. Década de oitenta, setenta, e aí vem esse momento, né. E o questionário abordou só uma pergunta da questão de droga. Entorpecentes considerado droga, né. Nós tivemos só dois. Por que só dois, né?

Referência 3 - 0,10% Cobertura

Sim, nós vamos ter o álcool como entorpecente, nós vamos ter a maconha que é entorpecente. Mas não são só essas as drogas que existem. Tem medicação, né, nós vamos ter, como é que é, sintéticas, nós vamos ter outras, e não aborda.

Referência 4 - 0,16% Cobertura

Isso. No sentido porque... é como mais se tivesse mais generalizado. Só o álcool e a maconha. E as outras são permissíveis, né. Não... trazendo ela também, chamando atenção, porque nesse rol de drogadição, nós vamos ter as lícitas e as ilícitas. E aqui nós trouxemos só as lícitas, né. Pra adolescente. E, assim mesmo, muito pouco. Eu acho, pelo menos.

Referência 5 - 0,20% Cobertura

Eu achei que faltou sobre a sexualidade. O [nome do adolescente] tá convivendo com uma colega, que até quando ele mudou pra essa escola ano passado, foi a menina que gostou, assim, de primeira, e a menina conversava com ele direto, viraram melhores amigos. Dos melhores amigos começaram a passar por um algo a mais. Ele queria ficar com ela, mas ela não queria ficar com ele. Daqui a pouco a menina, do nada assim, de um dia pro outro, cortou o cabelo

Referência 6 - 0,05% Cobertura

e "Agora sou lésbica". E aí, "Mãe, que que tá acontecendo?", e aí começou a beber, começou a fumar, começou a usar droga

SOCIAL WORKERS (PAS)

<Arquivos\\ 0> - § 1 referência codificada [0,47% Cobertura]

Referência 1 - 0,47% Cobertura

Eu achei muito bom. Achei bom, achei muito interessante porque diretamente aqui e diretamente aqui se trata muito do, traz muito do contexto do núcleo familiar, que é onde diretamente a maioria das vezes acontece... E, não muito diferente dessas perguntas, na maioria delas, a gente também atua no conselho, assim, perguntando assim, talvez com alguma diferença ou outra, eu achei bem qualificado. Achei muito bom as perguntas.

<Arquivos\\ 1> - § 1 referência codificada [0,21% Cobertura]

Referência 1 - 0,21% Cobertura

Bacana, nota 10. Não é uma coisa muito extensa, pesada, que o adolescente lê, e cansa, e é uma coisa fácil de responder, é só marcar. Na verdade, bem boas as perguntas, bem rápidas.

<Arquivos\\ 2> - § 3 referências codificadas [1,79% Cobertura]

Referência 1 - 0,04% Cobertura

Achei as perguntas bem pertinentes.

Referência 2 - 1,32% Cobertura

Talvez haja algum desconforto em responder algumas perguntas bem íntimas. Talvez o adolescente que não para pra refletir sobre essas questões, quando se depara com alguma coisa e reflete, isso possa causar alguma associação. Mas acho que é um caminho assim pra se se atentar a isso, é muito importante, da mesma forma acho que também acho que teriam bastante interesse, porque como eu falei nesse programa da saúde da escola a gente vai conversar sobre alguns assuntos de saúde em geral dos adolescentes, reprodução sexual, questões de doenças sexualmente transmissíveis, de conversar sobre outros temas, como o bullying, como relações interpessoais. Claro que a gente trabalha no coletivo, mas já teve situações que alguns vieram procurar e falar sobre uma situação específica assim, que sentiram provocados ou o tema que foi tratado foi um disparador para algo que estava incomodando. Então acho que é algo que seria bem interessante, levado às escolas, alguma oficina, alguma coisa, seria algo para poder mapear um número grande de situações que poderiam ser identificadas.

Referência 3 - 0,43% Cobertura

Acho que antes de iniciar tem que ter uma instrução do que tá por vir, que algumas perguntas podem gerar algum incômodo, mas que são necessárias. Talvez orientar para fazer com alguma pessoa em quem confie, algum amigo, alguém com quem se sinta melhor para poder dar apoio no caso de uma situação que possa fazer com que o adolescente se sinta mal.

<Arquivos\\ > - § 2 referências codificadas [0,83% Cobertura]

Referência 1 - 0,55% Cobertura

"Como é o relacionamento com a sua mãe?" Geralmente, tem problemas, assim, até na relação ou uma relação muito ligada, assim, que isso faz uma simbiose, né, com essa mãe. Ou não tem uma relação boa, ou foi abandonado, então, né... Então, é bem... A questão familiar geralmente vem, vem junto, assim, né. Com o pai, é a mesma coisa, relacionamento com pai, quando a relação não é boa, é um... Mas o pior é quando não com a mãe, assim. Também
tem problema familiar, "como é o relacionamento entre seus pais?", geralmente o casal ou está separado ou tem uma história de agressão ou dificuldades, assim, de relacionamento que fazem essa dificuldade, assim, bem clara para esse adolescente, né. "Você já foi separado de seus pais para ser cuidado por outra pessoa?" Tem bastante... Isso em relação às crianças que a gente atende aqui?

Referência 2 - 0,28% Cobertura

Eu acho que sim. Eu acho que poderia ter também essa, essa... Já teve vontade de morrer, de sumir? Porque isso também é importante, né, porque, às vezes, ele não tem plano, não pensou como é que eu vou fazer, mas já teve essa vontade. Já tive vontade de morrer, mas vontade, plano, com ideação, assim, um planejamento da ação, aí já é mais complicado, né. Vou tomar, minha mãe tem remédio, eu vou tomar...

<Arquivos\\ > - § 6 referências codificadas [1,31% Cobertura]

Referência 1 - 0,07% Cobertura

Entrevistado: Acho que vocês podem colocar 12 anos.
Moderadora: Ah, sim, aumentar a idade.

Referência 2 - 0,25% Cobertura

É, é mais ou menos aquilo que tu vai perceber a questão das negligências e dos abandonos: alguma vez você já foi separado, segurado por alguém, tem brigas com agressão física... Alguém te bater de um jeito que te deixou machucado... Ótimo. Já fugiu de casa... Privação, é. Vocês podem colocar nessa pergunta “ou outras”, né.

Referência 3 - 0,16% Cobertura

Gente, é ótimo, excelente. Eu acho que ele contempla bem as questões que eu sempre... Coloco como risco, né, que é essas questões das negligências e dos abandonos na infância e também na própria adolescência.

Referência 4 - 0,07% Cobertura

Ah, ótimo de responder. As perguntas são breves, são curtas, elas são bem identificadas.

Referência 5 - 0,59% Cobertura

Eu acho que talvez alguns outros lugares também, poder talvez dimensionar alguns serviços que ele possa procurar ajuda urgente na cidade. Uma coisa mais ampliada do que... Esse 188, né. Poder falar de espaços como conselhos, delegacias da criança e do adolescente, emergências de saúde mental, acho que poder falar um pouco do mecanismo, né, de maior descrição, assim. Acho que essa frase final, "encoraje ele ou ela a procurar ajuda de um adulto que confie, professor ou profissional de saúde". Talvez explicar, quando fala de profissional de saúde, tu pode dizer, assim, ah, pode ser o médico do posto de saúde perto da tua casa, pode ser o último médico que tua mãe te levou, pode ser, se tu tem, né, pode conversar ou não, pode ser teu dentista.

Referência 6 - 0,17% Cobertura

É, de referência, quando ele conhece. Se bem que a gente não consegue falar muito com o dentista. Eles que falam mais, por isso que a gente acaba não gostando muito de dentista, porque eles passam o tempo todo falando.

<Arquivos\\ > - § 6 referências codificadas [1,29% Cobertura]

Referência 1 - 0,29% Cobertura

Não, o questionário, ele, ele é de um tamanho adequado, não é, não é, muitas perguntas acho que são objetivas, o adolescente não tem muito saco de... de ficar lendo, ficar respondendo. E não sei se seria legal botar uma questão em aberto: "Você tem algo pra dizer que não disse aqui, que não foi perguntado?"

Referência 2 - 0,13% Cobertura

É, eu acho bacana. Eu fiquei pensando aqui, será que se definir gênero, seria legal botar masculino e feminino ou botar só "Qual seu gênero?"

Referência 3 - 0,26% Cobertura

E, assim, eu participo muito, não sou do movimento negro, não, não sou, não... não tenho lugar de fala nesse lugar. Mas... mulata e pardo é pejorativo, né. Amarelo é pejorativo. Eu tiraria, né. Eu botaria branco, negro, indígena. E não, eu tiraria essas... acho termos pejorativos.

Referência 4 - 0,33% Cobertura

Mas acho que é isso. Acho que aborda o que... Essas questões de relacionamento, assim, tá "Alguma vez você já foi separado de seus pais para ser cuidada por outra?" Sim, não. Eu não sei se eu botaria "seu pai" e "sua mãe" porque eu acho que restringe muito... por exemplo, sei lá, eu atendo muitos adolescentes que são criados pela tia desde que nasceram.

Referência 5 - 0,19% Cobertura

A mãe - é, ou pelos avós - bom, foram a óbito. Tem alguns adolescentes que não foram, não foram separados por uma questão, né. Eu, eu botaria "seu responsável legal, seu cuidador principal". Nesse sentido.

Referência 6 - 0,09% Cobertura

Não, acho que tá bem bom. Ela tá objetiva, sucinta, só sugeri uma pergunta mais aberta no final.

<Arquivos\\ > - § 4 referências codificadas [1,19% Cobertura]

Referência 1 - 0,60% Cobertura

Eu achei bom, assim, mas... O tamanho é bom, ok. É compreensível, também, mas eu acho que... Eu penso no caso dos meus alunos, se eles tivessem, eu acho que geraria muitas, sei lá, muitas inquietações. É que daí o vínculo já tá rompido, né, então se for pensar em alguém que ainda não rompeu o vínculo com a família, eu acho que talvez seria adequado isso. Mas... no meu caso, acho que seria, geraria muitos gatilhos, muitas coisas, sabe. As perguntas, porque são bem relacionadas ao que eles vivem.

Referência 2 - 0,21% Cobertura

É, eu acho que poderia. Mas eu acho que todas as possibilidades ali que apareceram pra dar alto risco, eu concordo assim. A questão da família, da agressão. Da fome, também.

Referência 3 - 0,10% Cobertura

Eu pensei agora, até. Eu colocaria gravidez na adolescência. Também acho que...

Referência 4 - 0,29% Cobertura

Eu acho que no alto risco teria que ter uma explicação um pouco melhor de como recorrer ou procurar algum serviço ou alguma coisa, sabe. Acho que um pouquinho mais detalhado, assim, .. de como seria os trâmites. Baixo risco acho que não.

<Arquivos\\ > - § 4 referências codificadas [2,40% Cobertura]

Referência 1 - 0,72% Cobertura

Tem mais chance de eles responderem sim, "Tu já sentiu que alguém te odeia?", acho que todo adolescente acha, né, que, "Ai, me odeiam", "Odeio todo mundo". Sabe o que que me ocorreu aqui? É porque assim, ó, tem, tem coisas aqui, talvez essa aqui seria, aqui se aproxima mais do que eu penso. Porque, assim, ó, aqui tem perguntas bem, digamos assim, bem pontuais, bem objetivas, né. "Já teve briga na sua casa?", "Tu já foi separado?, "Já aconteceu de um adulto te bater?", "Tu já fugiu?", mas tem aquela coisa da indiferença familiar. Por exemplo, "Você já pensou ou sentiu que o seu pai ou a sua mãe não queriam...", tá. Isso é uma coisa, a outra coisa é assim, ó, não tem briga, não tem roupa rasgada, não tem machucado, mas tem uma indiferença que, às vezes, eu escuto dos jovens, sem trabalhar, eu não estou abordando eles, mas, assim, da conversa dizendo, "Ah, mas a pior é a fulana que a mãe dela nem, nem...

Referência 2 - 0,54% Cobertura

Ele dá dinheiro pra tudo que ela precisa, mas ele não... não passa nenhum, não, não, não convidar ela pra nada. E a coisa tava, digamos, chegou pra mim nessa conversa porque agora o filho do pai com a atual esposa tava de aniversário e ele não convidou essa filha para conversar porque a atual esposa não gosta dela. Então, não tem briga, não tem roupa rasgada, mas tem uma diferença, tem uma não opção para o filho. E essa menina fez quadros de depressão... e teve internada tomar Rivotril em excesso etc. etc. Então, talvez devesse ter alguma pergunta aqui que não é dessa coisa tão dramática porque tem famílias que não vai ter violência, que não vai ter brigas, mas que não vai ter nada.

Referência 3 - 0,98% Cobertura

Eu acho que a pior coisa é, é, é a indiferença. Há muito tempo atrás eu tava trabalhando na disciplina de família em sala de aula, estávamos discutindo justamente essa coisa da indiferença... que são, assim, que tem um autor argentino que chama os órfãos de pais vivos. E daí uma aluna trouxe o exemplo do sobrinho dela que, que sumiu de casa, sobrinho de 9, 10 anos, sei lá, sumiu de casa e a mãe ficou que nem enlouquecida procurando, horas depois achou ele na casa de um amigo e bateu no guri. Deu uns tapa nele, na bunda, sei lá onde. E ela disse que a irmã de, a irmãzinha desse, desse menino disse pra ele, "Ah, tu não tem vergonha tu já com 10 anos apanhando da mãe?", e ele respondeu, "O pior é..." o amigo outro que também tinha sumido de casa, [nome do menino], sei lá que nome tinha, que a mãe dele nem fez nada. Então, assim, não é ser a favor da palmada nem nada, mas ele entendeu que a mãe dele tava desesperada porque não achava ele e quando achou, descarregou, talvez não do jeito mais adequado, mas mostrou da ansiedade. E a mãe do amiguinho também tava procurando pelo amiguinho e nem fez nada, ou seja, essa indiferença... que eu não sei que forma de pergunta poderia ter aqui porque tem alguns que iam dizer, "Não, olha."

Referência 4 - 0,16% Cobertura

Deixa eu ver... "Já pediu...", "Costuma se encontrar com os amigos...", "No último ano, já entrou em alguma briga...", "Alguém já tentou..." Bom, isso aqui vocês vão encontrar um monte.

<Arquivos\\ > - § 3 referências codificadas [0,76% Cobertura]

Referência 1 - 0,38% Cobertura

Eu achei que foi muito bom. Até porque, assim, ali fala o que eu te falei por último, porque muitas vezes tem a questão do abuso. E ali fala sobre a questão se alguém teve contigo, né... No caso, com a gente, a questão de ato sexual, uma coisa que a gente não gostou e que foi imposto, né. Acho que tem a ver também com a questão de uso de droga. Uma ou duas ou três, quatro, cinco vezes, pelo o que a gente detecta com algumas

jovens lá que desencadearam depressão e usaram droga, depois não quiseram mais, pararam. Teve aquele vazio, a questão sexual... Assim, as perguntas são muito boas, assim, eu acho que o propósito... Adequadas, muito adequadas.

Referência 2 - 0,28% Cobertura

E aquela pergunta ali que fala ali, né, da gente usar coisa dos outros também, né. Isso eles trazem para a gente. Tem uma pergunta ali que "já usou roupa velha, já usou coisa...", tem ali, aquilo ali, eu me lembro, assim, que eles trazem pra gente, sabia? De ter que estar usando, que eles só usaram roupa velha, que ganhavam dos outros, assim. Teve ummonte de coisa ali que eu fiquei aqui, que eu respondi "não", mas se eles respondem, eles falam com a gente isso.

Referência 3 - 0,10% Cobertura

Isso. A questão ali das brigas também. O que tem de pai e mãe, mau trato, essas coisas, assim, tudo. Olha, esse questionáio aí é bem, bem, bem, chega muito perto, bem legal.

<Arquivos\\ > - § 3 referências codificadas [2,23% Cobertura]

Referência 1 - 0,52% Cobertura

Acredito eu, tá, que quando a gente pergunta "Como é o relacionamento com sua mãe?" e já trazendo algumas opções de resposta, eu acho ideal elas virem, né, mas... elas tão muito influenciadas em conceitos. Que que é o conceito de "ótimo"? O que é ótimo pra mim... o que é ótimo pro adequado de ótimo pode não ser ótimo pra aquilo que aquele adolescente entende quanto ótimo. Ponto forte. Eu dizer "ótimo" ainda, acredito eu, que ainda fica, né... E, então, uma vez aberta uma aba de "ótimo", mas explique melhor o que que é esse teu ótimo... parece que a gente consegue se adentrar mais dentro desse universo.

Referência 2 - 1,36% Cobertura

O que que é o ótimo, né, explique melhor. Se for bom, o que que é esse bom, né?. Se for ruim, o que que é esse ruim? Porque é aquilo, adolescentes entendem que alguns comportamentos de familiares é péssimo. Mas são comportamento, às vezes, exigindo dele questões pra que ele possa evoluir enquanto qualquer pessoa, né. Construção de vida. Mesmo que cada um tem seu tempo. Então, ele me dizer que é ruim tem um conceito, né, e assim vai com os outros perguntas subjetivas, né. "Pais, entre os pais uma vez, alguma vez já foi separado...?" É de volta pra essa coisa subjetiva, né. "Já pensou ou sentiu... que mãe ou pai não queriam que você tivesse nascido?", né, abrir isso, né, porque quando é pergunta de coisa subjetiva, novamente eu volto pros conceito. "Você já pensou ou sentiu", né, volta pro conceito. "Você repetiu no ano alguma vez na escola?" Eu perguntaria... Quando tá com amigos, né, para conversar, jogos ou fazer alguma coisa, sobre o que vocês conversam? Exploraria mais essa questão aqui em caso de sim, motivação da briga, se ele entrou em briga, né, e se machucou ou outro se machucou, né, eu exploraria mais no sentido só, só uma aberturinha pra dizer um pouquinho mais, o motivo da briga. Porque são expressões, né, da pessoa trazendo alguma questão, né. É, aqui, não sei, tá, vou só trazer um pensamento; "Alguém já tentou fazer coisas sexuais com você a sua vontade, contra a sua vontade, te ameaçando, te machucando?", se tratando de adolescente, menor de idade, uma resposta sim... O que que nós vamos fazer com essa resposta? Vamos notificar, não vamos notificar?

Referência 3 - 0,35% Cobertura

Aqui, essa questão da droga, a questão da maconha, como vem bem específica. Tem adolescentes que fazem uso de outros tipos de entorpecente e não fazem uso da maconha. Como drogas sintéticas. E é bastante, eu fiquei assustada quando eu me deparei com isso. Que entre eles rolava isso. E não fazem uso da maconha. Então ficar só preso na maconha como se a maconha fosse a centralidade, fosse só ela fator de risco.

<Arquivos\\ > - § 3 referências codificadas [2,87% Cobertura]

Referência 1 - 1,38% Cobertura

Viu, bem da idade que eu falei, dos 14 até os 18, é adolescente. Também nessa pergunta, na 5, que vocês fazem, Como é o relacionamento com sua mãe. Ainda hoje eu estava conversando com uma colega que está com um problema com a sua filha, eu também tenho filhas que já são adultas, que passaram pela pré adolescência, por que eu passei por todas as fases. Então essa minha colega chegou e me disse assim, ai [nome da entrevistada], o que que eu faço com a minha filha, ela 16 anos, se envolveu jovem também, ela se separou e agora ela está em depressão, não sei o quê, não sei o que. Eu converso com ela. Eu já falei que se ela quiser deixar a filha dela comigo para sair. Eu escutei minha colega, daí cheguei e olhei pra cara da minha colega e disse, vou te fazer só uma pergunta. Ela tem amigos? Ela disse, não, por que ela se envolveu, se apaixonou. Eu digo, começa pelo seguinte: nós somos mães, não somos amigas, a gente quer tratar os nossos filhos como amigos, mas nós somos mães. Não é contigo que ela vai falar as coisas que ela quer falar. Então aqui, Como é o relacionamento com sua mãe? depende como. Um relacionamento bom, porque a minha mãe é uma pessoa legal , tá, mas a minha mãe é uma pessoa boa pra mim desabafar?

Referência 2 - 0,61% Cobertura

Vocês teriam que fazer duas perguntas em uma pergunta: uma, é saber o relacionamento familiar. Aí sim aqui ele vai poder te responder, é bom, é ruim, muito bom, ótimo. Mas é o outro, o relacionamento de amizades. Eu acho que tem que botar esse detalhe, entendeu? Até onde esse adolescente vê os pais como amigos. Essa é a questão. Ah, relacionamento com os pais. Acho que está dentro do que eu estava acabando de dizer agora, eu não tinha lido isso aqui. Mesmo assim, acho que o adolescente não vai dizer. Não vai responder o certo.

Referência 3 - 0,89% Cobertura

Essa pergunta é muito boa. A oito. Se foi separado dos pais para ser cuidado por outra pessoa. Isso aqui, quando tu vê o sim, tu pode saber que que essa criança tem algum ressentimento, alguma mágoa. Ficou marcada no passado, por algum motivo. A pergunta que o adolescente sempre se faz é por que eu, que futuramente essa pergunta, quem
responde o sim, tu já pode botar um sinal vermelho, que futuramente vai ter algum problema. Uma pergunta muito boa. Acho que para mim por enquanto, de todas as que eu li, é a melhor. É, a 8, a 9, deixa eu ler a 10. Olha, da 8 até a 14, eu tiro o chapéu. Vocês pegaram no calo da ferida da pessoa. Ou eu estive conversando com vocês ou vocês estiveram conversando comigo, porque praticamente o que eu falei é o que vocês estão perguntando aqui.

SCHOOL PROFESSIONALS (PE)

<Arquivos\\ 0> - § 1 referência codificada [0,09% Cobertura]

Referência 1 - 0,09% Cobertura

Mas alguns escreveram além do sim e não alguma coisa... porque o questionário dá essa possibilidade de escrever espontaneamente?

<Arquivos\\ 1> - § 3 referências codificadas [1,64% Cobertura]

Referência 1 - 0,59% Cobertura

Eu achei que talvez... fosse legal, considerando as crianças de periferia, a pergunta 8 vir antes das perguntas sobre mãe e pai. Porque daí, quando eles vão ler que “para ser cuidado por outra pessoa”, eles vão entender que mãe e pai é mãe pai. [no caso, pai e mãe biológico]. Eles... não vão pular pra responder... nunca vão pular pra responder sobre quem eles chamam de mãe... ou quem eles... Eu diria assim que em um ou outro caso, isso poderá gerar uma confusão aqui, que talvez fosse melhor botar a pergunta 8 antes.

Referência 2 - 0,36% Cobertura

Eu não achei grande demais, e também essa coisa de só responder sim e não, as respostas são leves, não acho que tem problema aí. Eu achei difícil essa parte aqui, de que só tem [como marcar] um tique. É estranho não ter escolha, se é sempre uma escolha, mas enfim. Vocês só aplicariam isso com gente dessas idades, eu suponho.

Referência 3 - 0,69% Cobertura

Pois é. Não me ocorre, porque o que me ocorre que faz falta são as informações locais. Tipo assim, procure o fulano. Tipo, o fulano ou um adulto... [uma coisa mais localizada pro ambiente...] É, de repente tu tem uma psicologia ou uma orientação educacional lá na escola que é sensível, é um canal... ou seja, acho que esse jovem precisa saber que ele não tem que ir pro SOE -- mas deve saber que existe. Se fica só na base do alguém que você confia, de repente, eu confio em alguém? Não sei. Mas aí não é uma coisa que falta aqui no geral, é uma coisa que precisaria de complementação em cada ambiente de aplicação.

<Arquivos\\ 2> - § 3 referências codificadas [2,11% Cobertura]

Referência 1 - 0,12% Cobertura

E as perguntas são bem interessantes, bem simples, mas que acaba possibilitando uma identificação. Muito legal.

Referência 2 - 1,25% Cobertura

Olha, eu gostei... Tem uma aqui que eu não sei se me chamou atenção. Ah, fala aqui se ele tentou fugir de casa e poderia.... onde que é que está... Ah, você já fugiu de casa... Na verdade se tivesse uma outra pergunta ou alguma coisa sobre se ele pensou, mas também fica mais forte o fugiu, se ele realmente fugiu... Porque às vezes temos muitas situações na escola, que a gente diz assim, ó, não quero mais viver nessa casa, mas não consegue fugir, porque não pode fugir, porque não tem para onde fugir mais essa. Imagina então a situação que tá. Pra mim, se essa se essa situação não se resolver, leva a uma situação de risco, porque aí há uma pressão interna. Às vezes fugir de casa desafoga. Enfrenta desafios e tal, e aí tem que voltar, dá um problema, alguém vai dizer não não te quero agora mais em casa. Basta mudar e ver a vida de outra forma. Agora, e se isso não acontece, fica só dentro da pessoa. Essa é a única questão que eu... se você pensou já seria mais, algo interno para se refletir né. Mas as questões bem interessantes. Agora, a questão talvez, bebida alcoólica, maconha... Sim, porque eles usam... é o que mais é comum, né.

Referência 3 - 0,74% Cobertura

Acredito que não há necessidade, talvez algum desenho, alguma uma imagem criativa, e uma apresentação visual. Alguma coisa assim, ah, duas brincadeiras, Emoji, qual a carinha que identifica mais, aí ele aperta a carinha que ele está triste, entendeu, e aí já dispara, opa, como é que você se sente, tem cinco opções, aí, e agora, nesse momento, entendeu. E aí você faz duas situações dessas, já consegue identificar e levar pro texto de alto risco, porque ele vê a imagem, qual a carinha que você se vê melhor, se sente, né... Ou uma pergunta, também, alguma coisa que, onde você tá aqui e agora, o que é que você... como é que esse mundo reage em você. Se sente bem? Se sente mal?

<Arquivos\\ > - § 1 referência codificada [0,42% Cobertura]

Referência 1 - 0,42% Cobertura

Eu acho que poderia ter esse médio risco, porque... eu fiz... Eu respondi aleatoriamente ali, como se eu fosse uma adolescente, mas ali pergunta, assim, você sofreu, tipo, um abuso? Mas não com essas palavras. E aí diz assim: "Você saíria de casa..." e aí eu respondi que não. Eu acho que ou sim ou não, então... são perguntas que são bem direcionadas pra adolescentes, são bem claras mesmo. Mas eu acho que a resposta poderia ser... Baixo, médio e alto risco porque às vezes os alunos têm uma determinada situação, mas não tem outra, que isso não impeça, que não leva aquilo ali a um grau de depressão, a menos que ele diga sim em todas as questões. Eu acho que pra tu teres um alto risco de depressão, tu tem que responder todas as questões positivas, né?

<Arquivos\\ > - § 2 referências codificadas [1,32% Cobertura]

Referência 1 - 0,83% Cobertura

– Acho que pode ser mais perguntas que o adolescente traga em relação à sua família, grupo familiar, não só a mãe e o pai, mas como é a relação dele com o grupo familiar. Isso é muito importante. Porque eu não vejo muito, a minha filha é adotada, ela foi adotada bebê, então não teve menor interferência os pais biológicos. E o que aconteceu. Ela me imita, ela faz muita coisa que eu faço. Essas coisas me emocionam. Eu costumo deixar minha pantufa ali naquele lugar, e quando eu vejo, a pantufa dela está ali onde eu costumo deixar a minha. Acredito que a relação com o grupo familiar, que forma a autoestima. Por exemplo – a sua mãe demonstra equilíbrio, você confiaria seus segredos à sua mãe, isso....

Referência 2 - 0,50% Cobertura

O que pode ser melhorado é aquilo que eu disse, sobre o grupo familiar, puxar mais, obter mais sobre a família, sobre quem mora contigo, como é tua relação com o padrasto, madrasta.... Acho que complementando isso, é uma ferramenta muito boa. Essa dinâmica, assim, ó, o adolescente, uma pessoa, o máximo que pode acontecer é ele sair daqui e dizer, ah, deu tudo errado, a mulher disse que eu tenho alto risco de depressão.

<Arquivos\\ > - § 6 referências codificadas [1,54% Cobertura]

Referência 1 - 0,16% Cobertura

Entrevistado: Eu achei bem interessante, são questões que envolvem basicamente o âmbito familiar mesmo, né.

Referência 2 - 0,16% Cobertura

Entrevistado: São bem claras, bem objetivas. Não achei excessivo, assim, questionários aqueles longos que...

Referência 3 - 0,52% Cobertura

Entrevistado: Eu acho que por falar sobre drogas... Então, porque os alunos chegam em casa e contam, né, o que aconteceu para os pais e contam do jeito deles, né. Então, de repente, pensando numa mãe que é evangélica, "ah, foi questionada a questão da maconha..." Poderia acontecer de um pai ir até na escola e perguntar sobre isso. Ainda tem essa barreira.

Referência 4 - 0,15% Cobertura

Entrevistado: Olha, porque, na verdade, foi só um questionamento, não foi um incentivo algum ao uso, né.

Referência 5 - 0,15% Cobertura

Entrevistado: Eu pensei na questão da identidade sexual, também, poderia ser interessante perguntar.

Referência 6 - 0,41% Cobertura

Entrevistado: Não, acho que não, porque, de repente, o excesso de possibilidades de resposta também até confunda um pouco, né, o adolescente tenha uma tendência a dar uma mascarada, assim, na sua resposta. A gente espera que ele seja respondido com verdade, né. Mas não sei, né.

<Arquivos\\ > - § 4 referências codificadas [0,85% Cobertura]

Referência 1 - 0,34% Cobertura

Entrevistado: E quando, assim... Vocês falam muito em relacionamento com os pais. Pai e mãe... Só que a maioria, já posso dizer agora, mudou tanto que agora, a maioria das famílias, os adolescentes não estão mais com pai e mãe em casa. Então, assim, ou só estão com a mãe... Muitos estão só com a mãe, mas quem cria é a avó, a avó materna, ou... Ou tios, tias, ou o padrasto, madrasta... Mas é raro adolescente que diga, assim, "ah, eu moro com o pai e a mãe." É muito raro.

Referência 2 - 0,12% Cobertura

Moderador: E o que que a senhora achou, tipo, do tamanho do questionário?
Entrevistado: Achei bem razoável, assim... Claro, é bem resumido.

Referência 3 - 0,04% Cobertura

Entrevistado: Achei bom. As perguntas também boas.

Referência 4 - 0,35% Cobertura

Alguma coisa dentro da sexualidade, eu vi que não tem assunto relacionado a isso aqui. E para eles é importante isso. Eles precisam, sabe, tipo assim, uma pergunta, perguntas que eles possam refletir, tipo, dentro desse assunto da sexualidade. Como ele se sente, se ele já se identificou na área da sexualidade, se ele... Se a cabecinha dele está precisando de ajuda dentro desse assunto ou se ele já está bem posicionado em relação a esse assunto, assim, não precisa de...

<Arquivos\\ > - § 2 referências codificadas [0,85% Cobertura]

Referência 1 - 0,05% Cobertura

Ah, legal isso aí.

Referência 2 - 0,80% Cobertura

Eu acho que talvez poderia ser mais colocado, poderia ter se colocado itens, um ou mais itens, relacionados ao grupo de amigos. Porque adolescentes, eles, às vezes, o grupo de amigos - às vezes não, acho que na maioria das vezes - o grupo de amigos é muito mais referência do que pai mãe. Então, nesse sentido, como é que tu te sente na tua escola, com teus colegas e tu tem um grupo de amigos... Não só a questão de se eles jogam ou não com alguém. Acho que essa coisa dos pares é bem importante. Questões relacionadas aos pares seriam bem importantes

<Arquivos\\ > - § 9 referências codificadas [1,94% Cobertura]

Referência 1 - 0,11% Cobertura

Entrevistado: Ah, que eu tava falando ali da primeira, né. Ai, a cor. Será que é preciso colocar a cor?
Moderador: Tá, entra no processo de calculadora de risco. Tu acha que é ruim?
Entrevistado: Ai, eu não sei...

Referência 2 - 0,18% Cobertura

Não sei se precisaria essa parte. Não sei qual vai ser a diferença ou talvez vocês estejam procurando também em se dar a depressão... Essa parte toda ali dos níveis, mais em determinadas raças, isso tá influenciando, isso também, vocês querem saber isso. E qual raça que tá mais dando ou que vocês veem mais os sintomas... Se está sendo na parda, na amarela, na indígena...

Referência 3 - 0,20% Cobertura

Não, capaz. Uma criança vendo isso aqui... Um adolescente... Porque, talvez, eu pensando como adolescente, né. "Como é o relacionamento com seu pai?" Aí, ele "depende, quando meu pai tá bêbado, é assim; quando o meu pai chegou do trabalho, é assado. Meu pai no final de semana, pô, é legal", é bem relativo isso, né. Porque não é um relacionamento, acredito que não seja uma coisa constante, assim. Isso também...

Referência 4 - 0,26% Cobertura

Entrevistado: É, daquele momento. "De férias, o meu pai é uma coisa, quando ele está trabalhando, ele é outra." O que eu vou dizer aqui, o adolescente é...

Moderador: Quando eu briguei com o meu pai, eu vou responder mal.
Entrevistado: É, tudo isso, tem mais isso ainda. "Ah, meu pai não quis dar o dinheiro hoje pro meu tênis, maldito, ele é ruim." Mas ele não sabe que é para educar, que é pra de disciplinar, ai, guri ruim. "A minha mãe, a minha mãe hoje não fez batata frita, ruim." Aí, eu pensando, né, gurias, não sei se você concordam com isso...

Referência 5 - 0,17% Cobertura

Entrevistado: Ó, como é entre os seus pais... É, quando a mãe acorda, o pai acorda, eles se dão um beijo, quando eles vão sair, é, então, vou colocar aqui.
Moderador: A gente está falando num geral, né...
Entrevistado: É, no geral, no geral, como eles veem, né, sim, sim.
Moderador: É uma questão bem perguntada pelos adolescentes essa...

Referência 6 - 0,19% Cobertura

Entrevistado: E, às vezes, para o adolescente é muito... Esse negócio aqui da família. Você já sentiu que alguém da família o odiasse? Porque, para eles...
Moderador: Isso acontece muito. Sim.
Entrevistado: É, porque a tia que... Ou o tio, ou o pai, né, que não compra o que eles querem e não faz o que eles querem é que odeia eles. Muitas vezes, é isso. Mas odiar é tão forte, né? Eu acho.

Referência 7 - 0,12% Cobertura

Entrevistado: É. O ódio, ai, ódio para mim é coisa... Como adolescente... Aqui. "Alguém já tentou fazer coisas sexuais?", eu pensaria "Alguém já tentou..." Não é atos... Coisas... Porque aqui, coisas sexuais...
Moderador: É muito amplo.

Referência 8 - 0,50% Cobertura

O baixo risco. Tá, então, indicou que apresentará baixo risco para desenvolver depressão. Mesmo assim, ainda é importante que você seja... Siga cuidado, cuidando de sua saúde mental e de seu bem estar. Você pode buscar para aprender mais sono, dieta e comportamento saudáveis. É, também é, é que a gente entra numas, assim, né, gurias, que dieta e sono, ou o comportamento saudável mesmo tendo... Até esses dias a gente estava conversando no almoço. Mesmo as pessoas tendo alguns comportamentos saudáveis, né, retirando um pouco do sal, fazendo a sua caminhada ou andar de bike, a pessoa pode ter uma morte súbita do coração ou pode morrer de algum câncer de mama, mesmo tendo uma vida saudável. Aí, a pessoa que dorme bem ou que dorme, aquela pessoa que dorme mal, que toma café, ou que se alimenta mal, ela chega nos 80 anos bem boa ainda, bem disposta bem, bem... É bem difícil, né. Aí acho que talvez entre a genética, talvez se tenha alguma resposta, porque mesmo eu maltratando meu corpo, mesmo isso, aquilo, aquele outro porque eu digo, assim, do meu irmão.

Referência 9 - 0,22% Cobertura

Talvez, assim, mais questionamentos de como... Como você lida com uma frustração? Ou você lidaria com uma frustração... Ou com... É, com uma frustração ou um... Como é, não é um fora, não é, dizer assim, como você lidaria com um pai que está se negando a lhe dar tal coisa, sabe? Jogar essas coisas para eles, daí, faria, a sua reação seria de tristeza, de entendimento, entenderia seus pais? Tipo isso, fazer essas questões voltadas para eles como eles são em casa.

<Arquivos\\ > - § 3 referências codificadas [0,52% Cobertura]

Referência 1 - 0,26% Cobertura

Eu acho que não tem nada demais, acho que quem vai em busca de alguma coisa, vai... São poucas questões, não são em excesso, de fácil entendimento, e o sim e o não é bem a cara deles mesmo, que isso não gostam de escrever muita coisa, acho que está bem acessível, bem fácil.

Referência 2 - 0,10% Cobertura

Porque aí vocês já têm um site, né, já têm um lugar também pra ligar, então, no momento eu acho que tá bom.

Referência 3 - 0,17% Cobertura

Pra alguém que é de baixo risco, já oferece locais pra ele pesquisar. Então eu acredito que tendo lugares onde se acessa alguma coisa pra prevenção, eu acho que já é o suficiente.

<Arquivos\\ > - § 2 referências codificadas [0,93% Cobertura]

Referência 1 - 0,48% Cobertura

Que ninguém vai ver, só pra ele. Sabe? Porque... às vezes eu penso que é difícil, me lembro, né, de mim quando era jovem também, se alguém tivesse que, "Ah, mas eu vou dizer isso e que que vai ser" e tal. Então, não sei se aquilo ali às vezes... mas isso também sou eu, né. Que tem uma realidade completamente diferente, claro, pensando no jovem ali da minha realidade, talvez um jovem classe média, né, numa outra condição talvez não se importe tanto. Ali não sei se não causaria algum constrangimento, mas... eu acho que das outras perguntas... É, eu penso que foi bem adequado, perguntou das relações com o pai, com a mãe, com a família. Se já sofreu alguma agressão, se já faltou, né... comida, alimento.

Referência 2 - 0,46% Cobertura

É, muito objetivo. Muito alto e baixo. E aí ele já vai ler aquilo ali, eu acho que ele, sendo jovem, pode se sugestionar por isso, "Ah não, mas eu sou baixo, então eu tô bem. É o risco que tá apresentando, nem é tão ruim assim. Mesmo que diga ali que eu tenho que buscar uma ajuda, mas então acho que eu tô bem", sabe? Eu, eu, eu penso que talvez, em relação às perguntas, eu penso que sim, mas talvez a forma como traga pode haver outras possibilidades entre o alto e o baixo. Talvez algum intermediário... alguma coisa que, ou então quando for conversar com esse adolescente, mesmo que seja baixo, destacar "Ó, mas tem evidências aqui que apontam, que também dizem que tu pode. Então vamos..."

HEALTH WORKERS (PS)

<Arquivos\\ > - § 2 referências codificadas [1,21% Cobertura]

Referência 1 - 0,71% Cobertura

Tá, primeira parte, então. Eu acho que contempla várias coisas aqui que a gente até falou, né, sobre relacionamento com pai, com mãe, o relacionamento familiar de uma forma geral, né, entre os pais. A questão de não ter podido ser cuidado pelos pais. E aí, entram outros itens que a gente não tinha comentado, né, de abandono entre aspas, não necessariamente um abandono, mas precisar ter a figura materna ou paterna substituída. Questões de traumas, agressividade, né. Dificuldade de relacionamento na família... Então, acho que faz muito sentido, assim.

Referência 2 - 0,50% Cobertura

Os itens fazem muito sentido. Mesmo essas questões de traumas na escola ou a possibilidade de relacionamento com os amigos... E outras experiências aí, também, envolvimento com substâncias, né, com álcool e maconha. Acho que, sim, faz muito sentido. E acho que é bastante fácil, assim, de ser aplicado. As perguntas não são de difícil entendimento, não é um instrumento muito longo, então...

<Arquivos\\ > - § 2 referências codificadas [1,01% Cobertura]

Referência 1 - 0,31% Cobertura

Eles falam muito dessas questões de relacionamento familiar, né. Mas, assim, aqui geralmente existe muito a questão de famílias, face à grande maioria, monoparentais. Tem algumas que têm com padrastos, mas sim, dificuldade de dinheiro... história de violência na família, de abuso. Isso sim.

Referência 2 - 0,70% Cobertura

Eu acho que é uma questão bem preventiva, né. "Se alguma das questões que você respondeu positivamente estão causando sofrimento, é possível procurar ajuda. Então, você pode conversar sobre isso com um adulto, um professor, um profissional de saúde. Você também pode contatar a CVV." Eu só acho que até poderia colocar, assim, um profissional de saúde poderia colocar... ou a sua unidade de saúde básica mais próxima, né, porque às vezes eles dizem assim, "ah, mas eu não tenho dinheiro pra ir no médico, no psiquiatra ou no psicólogo", então, assim, falar com um professor na escola ou um profissional de saúde ou a sua unidade de saúde básica mais próxima.

<Arquivos\\ > - § 14 referências codificadas [7,21% Cobertura]

Referência 1 - 0,16% Cobertura

Aqui, a questão 4 "que cor, raça", a gente tem usado o IBGE, mas... Sim, pardo, é... É um debate ainda em aberto também, né, em geral o IBGE tem sido a referência, né.

Referência 2 - 0,60% Cobertura

É, por isso a autodeclaração. É, aqui dependendo já... "Como é o relacionamento com a mãe, com o pai, entre os pais?" Só tô vendo se tem, porque aqui nem sempre cuidador ou cuidadora vai ser mãe, pai, né. Então, por exemplo, crianças que estão em acolhimento institucional não vão ter essa referência desse pai, dessa mãe. Talvez mais adiante esteja contemplado, né. Estou pensando aqui, mãe e pai, entre os pais... mãe, a gente tem que considerar que relações homoafetivas, né, então, entre pais vai ser entre... mãe e pai ou a gente pode considerar pai-pai, mãe-mãe? E como é que a gente vai nomear isso? É uma questão importante, né.

Referência 3 - 1,33% Cobertura

Entrevistado: E muito presente, e ela pode, né, de alguma forma, trazer uma ideia de que, ah, mas, então, como eu não tenho pai e mãe e eu tenho só... enfim, uma outra referência, que pode ser a tia ou a vó, ou eu tenho dois pais que me adotaram, dois homens que me adotou, uma série de coisas e eu já tô fora.
Moderadora: Algo tá mal.
Entrevistado: Algo tá mal e o próprio instrumento aqui poderia indicar já um problema meu, né. Então, entre seus pais aqui fica, bom, pais considerando aqui as diferentes configurações familiares para que a gente não induza, assim, né, olha, você, então, já tem um problema. Porque... "Separado dos seus pais"... "Já aconteceu de ter brigas..." Aqui imaginando, eu não sei se é pensado para crianças em geral nessa faixa etária, aqui a gente tem, né, experiência em geral que muitas crianças que estão nas escolas, nos projetos, elas estão em acolhimento institucional, estão em abrigos, né. Então, quando elas vão, por exemplo, vão responder, e a gente tem que pensar em todo mundo, né, quando elas vão responder, então, parece que já não é bem pra elas. Porque "ter brigas na sua casa" pode "ter brigas no abrigo", né, que é uma casa. Uma casa lar, enfim. Então o quanto elas vão se sentir contempladas, "ah, eu faço parte desse grupo de 14 e 17, sou uma adolescente como a minha colega, só que a minha casa é diferente, não é uma casa com pai, mãe." Não sei se eu...

Referência 4 - 0,28% Cobertura

Então, aqui de um adulto ou adultos, né. Então, essa seria a 8, a 9, né. "Já aconteceu de um adulto que estava cuidando de você..." Aqui de novo, "de um adulto da sua família ou alguém que estava cuidando de você"... Pode ser familiar, né, enfim. Acho que vai na mesma...

Referência 5 - 0,22% Cobertura

É, direção. "Machucado", essa questão da violência, né. "Fugiu de casa" aqui, casa, o abrigo, que é uma situação de evasão, né, que acontece. A mesma coisa "casa", bom, a casa aqui vai tá, então, perpassando todas as questões, né.

Referência 6 - 1,03% Cobertura

De outra dimensão, de outra órbita. "Você já pensou ou sentiu que seu pai ou sua mãe não queriam que você tivesse nascido?" É, aqui talvez sim, porque... Alguém no sentido de cuidador, que vai ser a referência principal, né, "Você já pensou ou sentiu que alguém..." que é a principal pessoa que cuida de você, ou que é responsável por você, que poderia ser, aqui, um avô, que alguém da sua família te odeia, mas também pode ser alguém que ela não conheceu como familiar, parente mas... que é do abrigo, digamos, do acolhimento institucional, então, né. Essas questões. Assistências sociais. É, eu tava pensando quanto a 16 abrange, mas a ideia é encontrar com amigos, né. Acho que... estamos falando, assim, do questionário, né. A questão de gênero, ela acaba sendo uma... uma discussão também que tem perpassado todos os materiais. Então, quando a gente tá colocando, por exemplo, "com amigos", a gente sempre tá priorizando o gênero masculino. Então seria "Você costuma se encontrar com amigos, amigas..." Alguns, alguns... digamos pesquisadores, autores têm usado até outras formas, né, o X, o ES.

Referência 7 - 0,32% Cobertura

Mas pelo menos começar a cuidar pra que a gente não deixe só o gênero masculino em evidência, né. Então, encontrar com amigos, amigas ou de de alguma forma. Aí, claro, né... é uma questão... "No último ano, você se encontrou em alguma briga em que alguém ficou machucado?" Acho que só... Talvez "Você encontrou alguma briga em que alguém...?

Referência 8 - 0,21% Cobertura

"Alguém já tentou fazer coisas sexuais..." É que "coisas sexuais" talvez tenha que ser... "coisas sexuais" pode deixar... dúvidas. "Alguém já tentou fazer coisas com você, contra você... com você contra sua sua vontade?"

Referência 9 - 0,22% Cobertura

Eu, tô, tô pensando alto aqui que "coisas sexuais"... existem, existem alguns materiais, né, até, assim, pra trabalhar com crianças, enfim, tipo carícias ou, né, gestos. Mas eu agora, assim, não, não vou lembrar exatamente o material.

Referência 10 - 0,45% Cobertura

Principalmente esse "já tentou fazer coisas sexuais". Porque há uma série de indícios, que talvez não sejam tão sexuais, mas que vão remeter, né, assédio ou violência, abuso. "Você já tomou bebida de álcool?" É, eu acho que aqui, centrar só no álcool e na maconha... é, é, assim, a gente precisa diferenciar as drogas lícitas, as drogas ilícitas. Entre as drogas, nós temos várias lícitas, né, medicamentos em geral, psicofármacos, todos eles se classificam-se, né, na... na...

Referência 11 - 0,54% Cobertura

Né, numa concepção, uma... de drogas lícitas. Então, aqui ficou, imagino, bastante direcionado a algo que comumente na sociedade a gente pensa, "ah, adolescente..." - eu acho que esse é um problema pra gente, problematizar, né, adolescente. Álcool e drogas. Drogas igual a maconha. E acho que é bem mais quando a gente tá vendo que existe um uso de medicamentos cada vez maior... prescrito para crianças e adolescentes. E talvez a gente tem que avaliar se eles não estão... utilizando mais psicofármacos do que a própria maconha, né, e outras drogas. É uma questão bem complexa...

Referência 12 - 0,39% Cobertura

Delicada, assim, né, porque demanda uma discussão, posicionamentos. Mas acho que centrar em álcool e maconha que são drogas de ordem diferentes, uma é lícita, a outra é ilícita. Elas não são as únicas drogas, né. Algumas a gente não percebe como drogas, pensando até em senso comum, né, no sentido, né, de que a gente acaba reproduzindo, enquanto profissionais e pesquisadores, algo que não está mais sendo debatido...

Referência 13 - 1,38% Cobertura

É, a primeira frase, ainda mais sendo a primeira, né, "a pesquisa tem demonstrado que os itens que você respondeu estão associados com risco futuro de depressão". Depende de quem lê, não sei se seria o próprio

respondente ou a própria responder, ela é uma questão... que pode assustar e que pode ser uma... antecipação. "Se alguma das questões que você respondeu positivamente estão causando sofrimento, é possível procurar ajuda." Acho que isso sim. "Você pode conversar sobre isso com um adulto em que você confia, um professor na..." Professor, professora, que em geral são a maioria, as professoras, né, "na escola ou um", uma, "professional da saúde". "Você também pode contatar..." Certo... Tá, então, os contatos, o CVV... "Para saber mais sobre prevenção visite..." Acho que, é, visitar sozinhos um portal pode ser também, né, assim, bom, um adolescente que chegou lá, né, no seus 14 anos vai abrir o portal e vai enxergar uma série de coisas que pode remeter justamente, associados a um risco futuro de depressão, então... A informação é fundamental, mas acho que ela precisa de um acompanhamento, né. Tá, depois eu vou... voltar aqui só pra ver "Baixo risco. A pesquisa indicou que você apresenta..." É, aqui, esse site eu não conheço, então, né... Acho que... ele trata... "aprender mais sobre sono, dieta e comportamentos saudáveis". Sim, essa ideia né, de "você conhece alguém... fatores de risco... encoraje..." Olha aqui, tá, ó, "encoraje ele ou ela"...

Referência 14 - 0,11% Cobertura

É. "A procurar ajuda de um... adulto em que confie... professora ou profissional... também, né, profissional de saúde".

<Arquivos\\ > - § 4 referências codificadas [1,09% Cobertura]

Referência 1 - 0,17% Cobertura

Entrevistado: Não tem uma pergunta... Aqui, para mãe e para o pai, não tem, não posso responder? Tipo não tenho pai, não tenho mãe, né.
Moderador: É, não... No caso, não...
Entrevistado: Aí fica tudo como ruim, provavelmente. Quem não tem contato com o pai, por exemplo.

Referência 2 - 0,08% Cobertura

Bah, fugir de casa... Aparece às vezes. Então, outra pergunta interessante, encontrar onde, né? Em chats ou pessoalmente, né?

Referência 3 - 0,39% Cobertura

Acho que tem perguntas pertinentes. Acho só que tem isso, assim, de supor um pai ou uma mãe que... Eu botaria uma outra pergunta, não sei o quanto vocês vão ter autonomia, mas que é... Uma outra pessoa cria você? Tem uma ali que é muito importante, que é se, em algum momento, você já foi criado por outra pessoa... Isso é fundamental, quase todos os casos têm isso. Mas eu acho que tem isso às vezes, que não é. Talvez você está sendo criada, não sei se ajudaria muito, né, por outra pessoa que não sejam seus pais. Acho que tem que ter ali no pai e mãe uma que é não tenho contato com a mãe, não tenho contato com o pai...

Referência 4 - 0,45% Cobertura

Tem uma frase, não lembro bem ali, mas que fala "ah, tem questões na sua vida que te trazem angústia"... Então, eu acho que talvez desenvolver um pouquinho ali nisso, até por uma questão de conscientização e motivação mínima, assim, tipo, processo que tu está passando, não tá te fazendo, né. Talvez esteja te trazendo problemas. Talvez alguma coisa que falasse sobre como é importante e significativo esse momento da adolescência. Mais para que possa ver minimamente uma conexão dessas que eu falo, "bom, talvez eu precise de ajuda", né? Algo que pudesse construir melhor ali uma ideia de produzir ali uma demanda de atendimento minimamente não precisa seja alta mas algo que possa deixar bem claro e aberta a necessidade.

<Arquivos\\ > - § 2 referências codificadas [1,04% Cobertura]

Referência 1 - 0,31% Cobertura

Uma outra questão, viu, eu não sei nem o que que vem por aqui, mas a autoimagem. Autoimagem é bem importante, também, pra gente estar pesquisando e talvez deva aparecer nesse protótipo. Nem sei se aparece, mas a questão da autoimagem, assim, é algo bem importante com adolescente, que a gente trabalha bastante.

Referência 2 - 0,72% Cobertura

Tamanho do questionário tá ok. Talvez tivesse que ter alguma questão relacionada à autoimagem, que eu acho que é uma questão importante. A questão do próprio sono mesmo, como aparece aqui, sono, tipo de alimentação... São questões que a gente acaba colocando também na consulta de enfermagem, né, como é que é o sono? Como é que ele... Uma pergunta que a gente faz é o que que ele vê quando se olha no espelho, se ele gosta daquilo que ele vê, né, e relacionado a essa questão de auto imagem. Então, acho que talvez precisasse isso porque para o adolescente isso faz uma diferença imensa. Até para ele conseguir conviver no grupo, sabe. Mas, em termos, assim, do protótipo, de tamanho eu acho que sim, acho que tá bem bom.

<Arquivos\\ > - § 5 referências codificadas [0,71% Cobertura]

Referência 1 - 0,40% Cobertura

Aqui eu também não tô vendo coisas, assim, referente às mídias, que é uma coisa que os nossos jovens estão muito, muito expostos. Até aquele suicídio coletivo, né, aquela coisa orientada por internet. Aquela história da baleia azul e não sei o quê. Não sei se em algum desses aí contemplaria. "Costumam encontrar amigos para conversar, jogar, ou fazer outras coisas"? De repente seria outras coisas? Seria isso?

Referência 2 - 0,14% Cobertura

Mais específico de mídia, né. Esse aqui é bem importante. Agora recentemente tive uma paciente que adorava os Marvels, Marvels...

Referência 3 - 0,08% Cobertura

Tranquilo, porque é bem objetivinho, né. É pão, pão, queijo, queijo, né.

Referência 4 - 0,05% Cobertura

Fácil, fácil. Sim, fácil de compreender.

Referência 5 - 0,03% Cobertura

Fácil de compreender.

<Arquivos\\ > - § 4 referências codificadas [0,85% Cobertura]

Referência 1 - 0,05% Cobertura

Achei curto. Achei super curto, não sei se não está muito, com muita pouca...

Referência 2 - 0,26% Cobertura

É. Porque até me chamou a atenção aquelas coisas ali do tipo "já faltou comida?" e tal, entendo, a população de baixa renda, mas tipo eu fiz pensando no meu filho e, tá, não faltou comida, mas... As outras coisas, poderia ter agressões em casa, poderia sentir que os pais não quisessem que ele tivesse nascido, isso tudo acho que poderia, mas será que não poderia ter mais coisas ainda? Tem do relacionamento com os pais, mas não tem muito dos amigos, né?

Referência 3 - 0,08% Cobertura

Tudo bem, tudo bem. Vocês que são os especialistas. Bem fácil de responder. É mega fácil, e acho que para os adolescentes também.

Referência 4 - 0,45% Cobertura

Não, mas eu acho, assim, que se for trabalhar com os adolescentes para eles responderem, eu acho que tem que ser uma linguagem mais direta, menos palavras... Bem informal. E, não sei. Será que rolaria fazer uma pequena definição de depressão, até para tirar essa história dos mitos? Tem que ver se isso não vai dar um viés nos questionários. Se for dar, acho que não. Só, assim, "ah, a gente está aqui, quer conhecer um pouco vocês e tal." Acho que assim é melhor. Não acho que é antiético, também. E acho que com os pais e com os professores se eles forem responder que nem eu fiz - eu fiz pensando no meu filho -, se eles forem responder assim, daí eu acho que cabe, assim, dar um conceito de depressão, dar uma contextualização da importância, dizer que tem tratamento...

<Arquivos\\ > - § 7 referências codificadas [2,07% Cobertura]

Referência 1 - 0,51% Cobertura

Eu acho que bem importante perguntar da relação com os pais. A gente sabe que tem muitos estudos que falam dessa questão do abandono, né, o abandono emocional principalmente da figura paterna. Também associada à questão de ser testemunha de violência quando criança, né, essa coisa do... Dos pais separarem, violência física ou até alienação parental, também, né, é importante. É, essa coisa da comida, às vezes pode ser uma negligência também.

Referência 2 - 0,21% Cobertura

Achei bem interessante isso, assim, da questão da violência. Vocês não falaram da questão do abuso sexual, e abuso sexual, assim, tem estudos que apontam que todas as crianças...

Referência 3 - 0,16% Cobertura

Ah, tá. Porque tem estudos que falam que todas as crianças que sofreram abuso sexual vão desenvolver na idade adulta algum tipo de depressão.

Referência 4 - 0,10% Cobertura

Por que que vocês não perguntaram sobre "você já pensou em se matar?", alguma coisa assim?

Referência 5 - 0,25% Cobertura

Eu acho que ele está bem bom em termo de número de questões, porque são... Né, são 21, mas elas são bem simples de responder, então, acho que não tem essa coisa que demore, porque os adolescentes não têm paciência, né...

Referência 6 - 0,65% Cobertura

Olha, gente... Eu estou na comissão estadual de prevenção, valorização da vida e prevenção do suicídio. Então, a gente tem uma alta demanda e o serviço... Não tem serviço. Então, a gente tem que ter o cuidado de... Não de não divulgar, mas, né... Eu acho que talvez a questão de unidades de saúde perto da sua casa. Ou colocar os CAPS infantil ou de adolescentes, o CAPS AD, né, de adolescentes. Não, o CAPSi. Né, procurem o CAPS, os CAPS não são portas abertas, né, mas pelo menos é uma referência, ele bate lá e vão dizer "não, vai no teu posto de saúde e volta".

Referência 7 - 0,18% Cobertura

Eu acho importante talvez colocar os CAPS ou... Procure uma unidade de saúde, sabe, alguma referência para ele saber onde é que ele pode entrar na porta.

<Arquivos\\ > - § 1 referência codificada [0,64% Cobertura]

Referência 1 - 0,64% Cobertura

Em relação ao conteúdo e aplicabilidade, eu acho que essa foi uma preocupação bem legítima nossa quando a gente estava pensando e preparando esse instrumento no sentido de que a gente pensou em algo simples, barato, que pudesse ser aplicado por praticamente qualquer profissional, não necessariamente um psicólogo, um psiquiatra ou alguém ligado à saúde mental, e isso porque a gente pensou que um instrumento de screening, um instrumento para identificar risco, tem que estar nas escolas, nas UBS, quando for usado numa perspectiva do mundo real.

USERS (US): PARENTS

<Arquivos\\ > - § 1 referência codificada [0,54% Cobertura]

Referência 1 - 0,54% Cobertura

As perguntas são bem... Como é que eu diria, bem diretas, ao ponto, assim, né, realmente abordam temas que hoje em dia são bem... Corriqueiros, ou são difundidos, eu diria, né, acho que talvez sempre existiram, porém hoje em dia são bem difundidas, né.

<Arquivos\\ > - § 3 referências codificadas [1,56% Cobertura]

Referência 1 - 0,50% Cobertura

Foram perguntas até simples, não foram perguntas que mexessem com o psicológicos deles. Achei interessante, não tem perguntas tão invasivas, que vai levar o adolescente a recuar e não querer responder.

Referência 2 - 0,33% Cobertura

É um questionário que não cansa, é bem interessante. Poderia até ter mais perguntas mais invasivas, que fosse levar mesmo a resposta.

Referência 3 - 0,73% Cobertura

O questionário é atrativo, interessante, as crianças vão querer responder, por ser tecnológico, não ser em papel, é virtual, o problema é onde apresenta o grau de risco, é procurar ajuda. Atualmente é muito mais constrangedor tu procurar ajuda e aceitar ela, do que continuar do jeito que está.

1. Code Query Output – Delivery of the Risk Calculator

POLICY MAKERS (FPP)

<Arquivos\\ > - § 2 referências codificadas [0,63% Cobertura]

Referência 1 - 0,34% Cobertura

Entrevistado: Eu vou te dizer que a escola que tem orientadora educacional, é um instrumento maravilhoso. Porque o orientador educacional é aquele... O orientador é o elo entre o professor e o aluno, é o mediador. Então, tem muita coisa que ele sabe da família através do aluno, e que o aluno pode omitir ao preencher o questionário. Então a escola tem um orientador educacional que já conhece o histórico deste aluno, porque daí o orientador ele chama o pai ou a mãe pra conversar, chama a avó, vem a vizinha lá e faz um relato da briga que deu na casa no fim de semana ou...

Referência 2 - 0,29% Cobertura

Entrevistado: Por isso que eu digo, assim: a orientadora educacional da escola, se ela tem acesso a essa ferramenta, e aí dá para ver mais ou menos o que ele respondeu e o que ela colocou que ela sabe - porque às vezes é um aluno que tá na escola desde o primeiro ano. Ele cresceu dentro da escola. Conhece toda a evolução. Então, se na... Com 16, ele omite alguma situação, ela vai dar embasamento que lá, quando ele tinha 8 anos, já acontecia alguma coisa que era um sinal, uma luzinha piscando.

<Arquivos\\ > - § 3 referências codificadas [1,26% Cobertura]

Referência 1 - 0,05% Cobertura

Em outros settings, assim, tipo no consultório privado?

Referência 2 - 0,43% Cobertura

Na escola, acho que é uma péssima ideia, né, acho que a escola, acho que a escola... tem que lidar com saúde. Acho que a escola tem que poder trabalhar com promoção de saúde de uma forma geral, redução de fatores de risco e promoção de fator de proteção geral para a saúde. Acho que classificar as crianças nas escolas para risco para depressão... Eu acho que tem um risco de causar estigma de uma forma geral, tem um risco de preocupar, de forma...

Referência 3 - 0,78% Cobertura

As pessoas ficam preocupadas quando elas têm um risco para algo, né. Eu não ia gostar de saber que eu tenho um risco super alto para uma coisa enquanto eu posso não ter aquela coisa, né. Então, por exemplo, um risco super alto para Alzheimer, por exemplo, pô, mas eu não tenho nada para fazer para isso e eu... E eu... É um desfecho longe. Não é um bom exemplo, né, mas tipo, sei lá, um alto risco para depressão. Não, mas eu não tenho nada, tô aqui na minha vida, estou feliz aqui, etc. e tal. Eu me preocupo com essa com essa abordagem, já acreditei muito nessa abordagem no passado. Depois de ter passado pelo sistema de saúde, eu não acho que o setting da escola ou um setting de um consultório privado faça sentido. Eu acho que faria sentido para pessoas que já têm um alto risco do tipo, eu já tive um episódio.

<Arquivos\\ > - § 1 referência codificada [0,93% Cobertura]

Referência 1 - 0,93% Cobertura

Eu acho, assim, vendo os pontos aqui abordados, eu acho que ele é amplo, que ele pega questões familiares, pega as questões escolares e outros aspectos relacionados ao abuso sexual, baixa autoestima, e eu acho que é interessante, inclusive para a autoaplicação, monitoramento, se for... E pensando, já que a gente tinha falado na própria questão dos agentes comunitários, eu acho... Depende, também, tem que ver qual é o ponto de corte, né. Para ver, para não ser algo que todo mundo cai aqui dentro ou que ninguém entre, né, então, vendo

de acordo com um ponto de corte para identificar quem é de alto risco, eu acho que como screening, assim, como rastreamento, me parece válida essa ferramenta aqui.

<Arquivos\\ > - § 3 referências codificadas [1,31% Cobertura]

Referência 1 - 0,23% Cobertura

Tem que preencher algo autoaplicável, sem necessariamente ter um profissional de saúde junto, né? Porque, com essa orientação aqui, já está até passando para onde a pessoa pode buscar essa ajuda, né.

Referência 2 - 0,65% Cobertura

Outra coisa talvez é a pré-escola, a escola fez o questionário, identificou, né, esse adolescente com risco. Qual é a conduta, encaminhar para o serviço de saúde de alto risco? Talvez até pensando no escolar, isso pudesse ser mais útil, ou no profissional, né, um médico que estivesse fazendo essa avaliação, o próprio agente voluntário, o enfermeiro da unidade, né, e vir talvez já atrelado com algumas abordagens que talvez já tenham uma evidência mais clara - não necessariamente medicamentosa - de prevenção da evolução para depressão, ou... Seja vincular espaços de atividade física, artística...

Referência 3 - 0,42% Cobertura

Entrevistado: Sim, a gente tem, nesses outros tipos de avaliação de risco que a gente comentou, a gente usa bastante no cotidiano, a questão do tabagismo, o alcoolismo, avaliação de risco vascular. Hoje, o Telessaúde é um programa que tem abrangência internacional. A gente trabalha com muitos aplicativos dele, acho que um escore de risco como esse seria bem interessante de poder adaptar.

<Arquivos\\ > - § 1 referência codificada [0,52% Cobertura]

Referência 1 - 0,52% Cobertura

Não, aí, tá, eu acho que isso poderia ser aplicado em escolas, mas poderia se aplicar no ambiente de trabalho, entendeu? Por exemplo, sei lá, entre alunos aprendizes, entre estagiários, entre não sei, entendeu? Vários outros setores onde existam jovens, né, de alguma forma acho que talvez fosse interessante até mesmo talvez em universidades, eu não sei se essa faixa etária, enfim, se é específico para essa faixa etária, não entrei bem... Mas ter uma escala pra se medir isso de depressão em os jovens, né, envolveria jovens adultos também, acho que seria super útil, né.

USERS – FOCAL GROUP WITH PARENTS

<Arquivos\\ > - § 6 referências codificadas [1,25% Cobertura]

Referência 1 - 0,05% Cobertura

Mãe 1: É, eu acho que responder eu acho que até vai, porque é mais privado.
Mãe 5: Não precisa colocar o nome.

Referência 2 - 0,03% Cobertura

Mãe 5: Eles pensam assim, né, "Ai, ninguém vai descobrir que fui eu".

Referência 3 - 0,01% Cobertura

Numa consulta.

Referência 4 - 0,17% Cobertura

O meu, acho que na consulta não, porque como eu disse, quando fala em consulta ele já fica meio revoltado. Mas, assim, eu acho que em grupos, assim como ele fez na escola, que ele respondeu o questionário que foi

o que me trouxe aqui a primeira vez, que já é a segunda vez que eu venho aqui, né. E eu até me surpreendi por ele ter respondido o questionário. Eu acho que em grupo.

Referência 5 - 0,67% Cobertura

É, tem que ser em grupo eu acho. Tem que ser em grupo. Aonde eu moro tem um posto de saúde, eles têm um grupo de adolescente. E os profissionais foram até a escola pra convidar os adolescentes pra irem nesse grupo, né. Tanto que quando a [nome da adolescente] iniciou toda essa questão, assim, que não quer mais a boneca, já começou a querer mexer no cabelo, penteava mais. E aí eu comecei a bater na tecla do grupo dos adolescentes, grupo dos adolescentes. Quando veio a questão do namorado, mais ainda, grupo dos adolescentes, vai ser lá que vai ser abordado toda essa questão de sexualidade. Eu acho que os profissionais vão der falar com muito mais apropriação do que eu a questão dos anticoncepcionais, quais são alternativas que existem hoje, aquela toda aquela coisa. E ela adorava aquele grupo. Ela adorava, as colegas, era comentário da escola o grupo de adolescentes que tinha no posto de saúde. Adoraram. E daí a doutora e as enfermeiras faziam lá um coquetel lá pra elas. Assim, era o point das guria ir pro grupo dos adolescentes. E eu vejo esse, tô falando enquanto meninas né, mas porque os meninos também querem um espaço de fala. Não tendo a oportunidade de ser ouvido, só as meninas tem bastante questões, mas os meninos também. Mas os meninos não tinham, eram só as meninas. Mas eu acredito que, nesses espaços, eu acho que essa ferramenta, ela viria muito porque aqui também envia as perguntas, os questionamentos, né. "Ah, mas essa pergunta aqui, que", né?

Referência 6 - 0,32% Cobertura

Isso. Se eu coloco na sala de aula que a gente, claro, por uma questão de escala, numa escola tu consegue abarcar um número maior, né, mas a qualidade não seria tão apropriada quanto os profissionais da saúde. Com certeza iriam emergir questões da saúde que os profissionais iam ter condição de responder. E, em sala de aula, os professores não, não é a área. Professor de Matemática falar de anatomia? Né, que é o que vai vim, questões sentimentais, sexualidade, é o que vai vim. Que chama a curiosidade dos adolescentes, acredito eu, que é o que é atrativo a eles, né, que é essa fase, assim, do descobrir o beijo, transar, aquela coisa toda, e eu acho que o profissional da saúde tá mais apropriado.

SOCIAL WORKERS (PAS)

<Arquivos\\ > - § 3 referências codificadas [0,82% Cobertura]

Referência 1 - 0,14% Cobertura

Médicos psiquiatras conseguem avaliar isto de uma maneira boa, e psicólogos também.

Referência 2 - 0,13% Cobertura

Nas equipes que atendem nesta questão da depressão, em prontos atendimentos.

Referência 3 - 0,55% Cobertura

Moderador: E se os professores de escolas fossem treinados para utilizar, teria algum preconceito ou algo negativo que poderia afetar os adolescentes?
Entrevistada: Acho que não, quanto mais informação o profissional tiver, melhor é. Além da calculadora, se houver a sensibilização dos profissionais seria bom, minimizaria o estigma.

<Arquivos\\ 0> - § 10 referências codificadas [5,37% Cobertura]

Referência 1 - 0,53% Cobertura

Eu acredito que sim. Tudo com base em cima dos atendimentos. Eu acredito que mais de 90% responderia. Talvez com alguma restrição ao uso da maconha. Talvez, talvez com alguma restrição, pelo fato de... E da forma também com que... De repente com o que seria exposto isso, né. Mas acredito que essa seria a maior

dificuldade, assim, dentro deste, destas perguntas, acredito que talvez essa seria a pergunta com maior talvez restrição ou com uma resposta não real, assim, uma mentira.

Referência 2 - 0,90% Cobertura

Eu acho que, por exemplo, num espaço de proteção, por exemplo, no ambiente escolar, seria muito útil, assim, algo que não tivesse exposição, não tivesse exposição assim porque a gente sabe que tem muitas, muitas, mesmo tu estando dentro do problema ou da dificuldade, né, passando por aquele contexto... Alguns não gostam de expor, assim, se for um espaço mais, mais sigiloso, algo mais protetivo, assim, algumas... Algumas das crianças, dos adolescentes conseguem expor mais essa situação, assim, talvez também, em determinado momento, este questionário, assim, calculadora ela serve uma, ela serve, assim, como um pedido de socorro, assim. Ela serviria, né, como pedido de socorro, assim, bom, talvez ninguém detectou aquilo, ou seja, o início de um problema depressivo... Eu acredito que isso seria muito bem utilizado.

Referência 3 - 0,60% Cobertura

Eu acredito que, muitas das vezes, mediada por um adulto, até acho que seria ideal, até assim para ter uma avaliação posteriormente, assim, de que, bom, dentro de... Depois de responder, talvez seja necessário chamar uma conversa, assim, ou alguém, sabe, que pudesse estar identificando, pudesse até estar avaliando o perfil do adolescente e talvez colocando à disposição dele o próprio serviço, assim, né, olha, talvez, pelo que a gente observou aqui, é necessário buscar uma ajuda, sabe? Sugerir, assim, algo que pudesse ser feito.

Referência 4 - 0,02% Cobertura

Bastante.

Referência 5 - 0,34% Cobertura

Bastante. Olha, acho que... A gente sabe que, por exemplo, eu não tenho conhecimento, talvez tu até possa me responder, mas eu não tenho conhecimento de que foi feito ou já utilizado esse tipo de questionário, assim, em algum espaço. Se já foi feito alguma coisa no espaço de proteção ou em alguma escola...

Referência 6 - 0,52% Cobertura

Não? Então, eu vejo, assim, avalio como um meio que um bicho papão, assim, né. Se tratando de um adolescente, assim, porque é uma novidade, né. É uma novidade, toda a novidade, uma mudança, ela gera um certo tempo para poder se assimilar, para poder entender que, bom, é algo positivo mesmo que o risco seja baixo, mas que é algo positivo. Então, eu vejo muita dificuldade de inserção, assim, inserir nos espaços, assim. Essa acredito que essa seria, assim, a maior...

Referência 7 - 0,63% Cobertura

Não, acredito que dos profissionais até não, pelo fato de que a gente sabe que é um dever de todo cidadão fazer o seu papel e zelar pela garantia, bom, entendi que avalia um risco e acredito que, neste sentido, acredito que os profissionais não muito mais dos adolescentes porque seria benéfico também para os profissionais. Claro que tendo estrutura para poder desenvolver esse trabalho mas seria benéfico porque eu também acredito que aumentaria a qualidade de vida, né, desse adolescente e a qualidade nos estudos. Eu acredito que só teria, só teria a contribuir, assim.

Referência 8 - 1,08% Cobertura

Vamos ver... Não, acredito que talvez pudesse acrescentar alguma... talvez alguma outra ferramenta, mas eu achei bem qualificado, assim, de imediato, assim, não consigo... não consigo, assim, talvez até propor assim, né, mas... eu vi que na questão de... tem duas opções, né. A primeira seria através do serviço de internet, né, e a segunda através do, do, de telefone. Mas... talvez... talvez até especificar, talvez, um pouquinho mais aqui quando se trata assim do sofrimento com... Isso eu tô te dizendo pelo público que nós atendemos, né, de vulnerabilidade baixa, onde na maioria das vezes, assim, não tem... não tem o acesso a um, quando traz aqui o profissional da saúde. Abrangendo um todo. Mas talvez específico de uma unidade de saúde ou algo

parecido, sabe? Porque se tratando assim do profissional, talvez, assim, ficou bom, mas me parece uma coisa meio que só um particular profissional da saúde, sabe. Entende, eu quis dizer... Trazendo isso em cima do público que nós atendemos.

Referência 9 - 0,24% Cobertura

Exato. Exato. Porque têm dificuldades, né, no acesso à internet, mesmo com essa realidade hoje que todo mundo tem telefone, né, mas tem algumas situações ainda que é bem precárias, né, que não tem...

Referência 10 - 0,51% Cobertura

Exato. Entender, olhar, assim, talvez citarem alguns exemplos, ou esse próprio, por exemplo, o próprio questionário, daqui um pouco estar à disposição, sabe? Bom, tem aqui à disposição, daqui um pouco, claro que a gente sabe que a mídia social era tanto usada, né, para o bem, benéfico, quanto para fazer maldade. A gente sabe que talvez alguns dados não seriam reais, assim como todo (?), mas que também teria essa opção ali de poder estar fazendo.

<Arquivos\\ 1> - § 9 referências codificadas [3,40% Cobertura]

Referência 1 - 0,14% Cobertura

Moderadora: Você acha que os adolescentes responderiam?
Entrevistada: Com certeza. Os meus eu fazia responder.

Referência 2 - 0,24% Cobertura

Eu acho que poderia ser em todas as ocasiões. A gente ter acesso, ter acesso de repente na internet, por que eles usam internet, pode não tem computador em casa, mas eles usam, ter um local onde eles possam pegar...

Referência 3 - 0,49% Cobertura

Assim, um local, um posto, na escola, a Direção, algo assim. Mais ali identifica que a pessoa já tem pegou, né, porque de repente o pessoa vai pegar lá na minha casa, uma suposição, e vai pegar aqui na UBS e na escola. Então, pra que fazer três, é um só que é pra ser feito. É que tem adolescente que adora escrever mesmo, que adora fazer umas coisinhas, tem uns que gostam, outros não. Eu acho que é onde deveria ter os acessos.

Referência 4 - 0,52% Cobertura

Entrevistada: Eu, pra mim, falaria pra ele que a contagem ficaria toda ficaria a critério da pessoa que deu pra ele preencher, do profissional, entendeu?
Moderadora: Sim, então não ter as respostas.
Entrevistada: É, essa parte aqui. O profissional olha. O teu deu tanto, o teu deu aqui um alto risco, a gente vai te encaminhar, vamo vê o que dá pra gente fazer, temos tratamento pra isso, talvez não precisa tratar com medicação, ter uma boa conversa...

Referência 5 - 0,22% Cobertura

É que nem o do HIV, aquele que tu faz o teste imediato, na hora, é só tu e a enfermeira. Mas tu responde logo depois também né. Assim, tu responde um monte de coisa, mas é só tu e a enfermeira.

Referência 6 - 0,58% Cobertura

Com certeza. Daí de repente aqui, vamos supor, aqui, ó, alguém já tentou fazer coisas sexuais com você contra sua vontade, te ameaçando ou te machucando? Ele bota, vamos supor, não, e daí o profissional, vamos supor eu, posso ler de novo e ele pensar. De repente porque ele estava com outra pessoa e não quis responder. Ou se a mãe viu e ele não pode responder. Porque isso aqui não vai pra tua pasta, não vai pra nada. Isso aqui é só pra uma pesquisa, mas a gente vai tentar te ajudar, perante essa pesquisa.

Referência 7 - 0,33% Cobertura

É, mais é uma conversa. E dentro deste cálculo, dessa pesquisa que vocês querem fazer, particularmente, eu não tenho essa... não tenho esse cunho pra fazer isso. Mas deveria de fazer pros adolescentes. Nós temos saúde mental, mas mais é pros adultos. Fazer pros adolescentes, diferenciados.

Referência 8 - 0,64% Cobertura

Entrevistada: Diferenciado, eles gostam mais de...
Moderadora: Tipo uma coisa mais gráfica?
Entrevistada: é, eles gostam de Hip Hop, eles gostam mais de, sabe, uma coisa que chama atenção.
Moderadora: Que engajasse mais o que eles gostam.
Entrevistada: Ah, o que tá passando hoje na cabeça, ah, eu faço um desenho, pode ser qualquer rascunho, mas é aquilo que está na minha cabeça, e eu vou dizer que é uma árvore linda e maravilhosa de outro planeta. E quem é que vai dizer que é o contrário? Alguém foi no planeta pra ver?

Referência 9 - 0,24% Cobertura

Moderadora: Sim. Então na verdade dar isso pra eles de uma forma mais adequada com o que eles gostam, digamos.
Entrevistada: Sim, sim. Que eles trabalhem junto com vocês, no caso, e pra melhora deles.

<Arquivos\\ 2> - § 5 referências codificadas [3,44% Cobertura]

Referência 1 - 1,32% Cobertura

Talvez haja algum desconforto em responder algumas perguntas bem íntimas. Talvez o adolescente que não para pra refletir sobre essas questões, quando se depara com alguma coisa e reflete, isso possa causar alguma associação. Mas acho que é um caminho assim pra se se atentar a isso, é muito importante, da mesma forma acho que também acho que teriam bastante interesse, porque como eu falei nesse programa da saúde da escola a gente vai conversar sobre alguns assuntos de saúde em geral dos adolescentes, reprodução sexual, questões de doenças sexualmente transmissíveis, de conversar sobre outros temas, como o bullying, como relações interpessoais. Claro que a gente trabalha no coletivo, mas já teve situações que alguns vieram procurar e falar sobre uma situação específica assim, que sentiram provocados ou o tema que foi tratado foi um disparador para algo que estava incomodando. Então acho que é algo que seria bem interessante, levado às escolas, alguma oficina, alguma coisa, seria algo para poder mapear um número grande de situações que poderiam ser identificadas.

Referência 2 - 0,52% Cobertura

Acho que sim, acho que ele é..., talvez vá ser bem aceito porque, especialmente se for trabalhado em plataformas digitais, e também porque os adolescentes são muito curiosos, às vezes gostam de responder questionários, já tem muitos, principalmente no Facebook, esses jogos que tu responde e sai algum perfil do que tu pode ser, do que tu pode chegar, e é bem aceito. Então talvez por esse meio seja algo que desperte interesse.

Referência 3 - 0,42% Cobertura

Só em relação à..., talvez pensar em uma outra forma de elaboração das perguntas, apesar de saber que tem questões que não tem como fugir, devem ser perguntadas... Mas acho até que pra adolescentes eles conseguem até se abrir, acho também que é algo que não tá sendo falado pra ninguém, talvez eles consigam externar todas as questões pessoais.

Referência 4 - 0,66% Cobertura

Acho que talvez, como eu falei, por meio de oficinas nas escolas, qualificar, treinar professores também para poder usar essa calculadora, também profissionais de saúde, como... a gente tem esse programa que a gente também faz algumas atividades, mas acho que também ele pode ser autodidático. Ser lançado algum tipo de

programa enfim, talvez mudar um pouco a formulação dele para ter uma cara mais de jogo, talvez mudar um pouco dessa seriedade, que acaba gerando... acho que seria também autodidático, buscado de forma espontânea também.

Referência 5 - 0,51% Cobertura

Acho que sim. Como falei, até daqui a pouco a própria ferramenta da calculadora possa ser utilizada também como uma plataforma de mídia social para ter um alcance maior, ou ser também, criar um desafio a alguma coisa, porque a gente sabe que estão sendo ligados nesse meio, então seria um captador muito grande de adolescentes que estão fazendo uso, que estão depressivos ou que estão prestes a desenvolver o quadro.

<Arquivos\\ > - § 5 referências codificadas [0,55% Cobertura]

Referência 1 - 0,39% Cobertura

Entrevistado: Eu achei interessante, assim, para as pessoas, para uma pessoa que está no atendimento ou no posto de saúde ou num local, assim, preencher para ter mais uma ideia, né, porque o que a gente vê, assim, é que muitos não se aproximam, os profissionais, dessas perguntas porque não querem ouvir. Porque, se ouvirem, eles não vão saber o que fazer. Então, nem perguntam, por exemplo, o pediatra, o herbiatra, ele vai centrar mais nas questões relativas ao clínico, né, a dor onde é que tá, como é que foi, se teve febre. Esse resto aí, eu não quero nem saber, é o que a gente sente aqui.

Referência 2 - 0,01% Cobertura

Moderadora:

Referência 3 - 0,03% Cobertura

Moderadora: Cuidado e treinamento de quem fosse usar.

Referência 4 - 0,08% Cobertura

Entrevistado: É, porque tem as respostas ali que poderiam, né. As pessoas comentam, então, é bem complicado, assim.

Referência 5 - 0,04% Cobertura

Seria nas escolas. Do treinamento e atendimento de quem aplica, né.

<Arquivos\\ > - § 1 referência codificada [0,54% Cobertura]

Referência 1 - 0,54% Cobertura

Eu acho que bem aquilo que eu te falei, esse é um questionário que, tipo assim, eu posso... Eu vejo ele podendo ser aplicado em escolas, principalmente, uma ferramenta que pode ser trabalhada com escolas principalmente da rede pública, acho que como, apresentar como um protótipo de atenção até para as Secretarias de Saúde, de Educação, porque são, tem que estar nos espaços de circulação deles, o espaço maior de circulação deles é a escola. Então acho que é um mecanismo que ele pode ser disponibilizado para ser feito nas escolas. Ser apresentado como um projeto de intervenção para escolas... Seria o principal... Seria uma ferramenta muito importante, até para a política, mesmo.

<Arquivos\\ > - § 5 referências codificadas [1,99% Cobertura]

Referência 1 - 0,33% Cobertura

Pois então... em grupos de... Eu não consigo pensar em outro espaço a não ser a escola, infelizmente. Mas grupos de adolescentes são muito poucos em Porto Alegre, né. Acho que a assistência tem o ProJovem, que

é uma instituição... até não sei se vocês vão chamar o pessoal do ProJovem pra estar aqui, eles trabalham exclusivamente com adolescentes.

Referência 2 - 0,26% Cobertura

Moderadora: Acho que sim.
Entrevistado: Acho que são grupos que tem em toda cidade, por exemplo, e são... às vezes com 20-30 adolescentes e trabalha a noção da cultura, do teatro, né. E são mais geralmente adolescentes em situação de vulnerabilidade. Mas, fora isso...

Referência 3 - 0,14% Cobertura

É, fora isso, não vejo, porque a ideia é aplicar com adolescentes, obviamente, né. E daí é vocês irem ou os profissionais distribuir pra rede, fazer?

Referência 4 - 0,19% Cobertura

Bom, a questão da atenção primária, eu acho bem importante. As escolas. E eu acho que na política de assistência, também, pra trabalhar com adolescentes, o Projovem, seria interessante esses profissionais.

Referência 5 - 1,08% Cobertura

Os instrumentos precisam fazer sentido para os profissionais, senão eles engavetam, né. E outra: os profissionais precisam... se autorizar a chegar perto de adolescentes pra além da doença, né. Se relacionar com os adolescentes, né, de uma forma não prescritiva. Tem uma família que eu acompanho que são nove adolescentes e uma idosa que cuida, por exemplo, é uma república. Cada vez que eu vou lá é uma muvuca. Então, cada vez que eu levo um residente junto comigo, ele sai em sofrimento porque os adolescentes tão... um passa por ti e grita, daí tá fumando lá em cima e daí o baile funk tá pegando e daí... E esse é o funcionamento daquela família, eles não vão parar, sentar no sofazinho pra te ouvir falar. E isso o profissional não suporta, principalmente o profissional, o profissional da saúde. Ele não suporta que as pessoas não parem pra ouvi-lo de uma forma padronizada, né. E, e eles estão escutando do jeito deles, eles estão interagindo contigo. Então, pra mim, o principal desafio são os profissionais, eles conseguirem se permitir entrar no mundo do adolescente e não... que é o padrão, fazer com que o adolescente adeque ao padrão de atendimento.

<Arquivos\\ > - § 2 referências codificadas [0,70% Cobertura]

Referência 1 - 0,56% Cobertura

Eu vejo mais nós, cuidadores, usando do que os adolescentes. Porque daí tu conhece já a história, tu já consegue... e tu vai acessar isso, vai ter uma noção, né, do que que vai aparecer, mas daí ele não vai ter muito contato com isso. Eu até acho que não seria bom os adolescentes acessarem, também, seria uma coisa mais pra adultos porquê... até às vezes pode não ser e ele vai acabar acreditando que é uma coisa que não é e criando uma paranoia, uma coisa.

Referência 2 - 0,15% Cobertura

Não, eu acho até que não. Eu acho que seria mais recomendado pros adultos, mesmo, que já tem um olhar um pouquinho mais...

<Arquivos\\ > - § 3 referências codificadas [1,94% Cobertura]

Referência 1 - 1,13% Cobertura

Uau. Eu fico pensando, assim, por exemplo, dentro das políticas públicas, entre as políticas públicas, a gente costuma associar suicídio etc. com saúde mental, com doença, saúde, política de saúde. Só que acho que isso aqui tem que, os professores das escolas tem que conhecer. Eu não sei e daí, [nome da entrevistadora], acho que tu tem que vir lá com o [nome], o coordenador da pesquisa, qual é o risco de se banalizar essa ferramenta, né. Porque, assim, ó, eu não sei se como a escola, a escola é uma fonte, é um espaço extremamente rico,

necessário, ele se impõe pra esse trabalho, bom, tô falando de adolescente, onde é que a gente vai encontrar, né? Uma parte deles tá na escola. Então, assim, eu não sei se não seria, a escola não seria o primeiro lugar que pudesse saber ou que tem essa ferramenta ou... não sei se daria, se seria indicado o uso indiscriminado. Todo adolescente nós vamos aplicar. Não sei. Mas eu acho que a escola é o primeiro espaço porque é lá que eles estão. Né. É lá. Depois, eu acho que os profissionais de saúde... da, da, assim, poderiam ter... essa, serem conhecedores de que existe esse tipo de feramente e que, bueno, quando identifica alto risco, ele é um dos profissionais que tem que (?), não assim, "Agora eu vou te encaminhar pra alguém", não, né. Ele poder ser o profissional e dizer "Bom, deu aqui"... Tu entende, usar no cotidiano de trabalho quando começa a perceber que tem lá uma... um risco de...

Referência 2 - 0,76% Cobertura

Né. Quando, quando acende o sinalzinho vermelho, aquele, né, o radar diz ó, tá, a coisa tá ficando perigosa, então, ele poder ser o profissional e já pensar alguma ação efetiva, se vai atender com outros profissionais, se vai vincular esse adolescente a alguma atividade no posto de saúde, na comunidade pra ficar em observação, né, não perder esse adolescente do radar. Então. Agora, escola em primeiro lugar, acho que a política de saúde nos postos de saúde etc. E... não sei. Aí eu fico muito, pela minha profissão, eu fico muito vinculada à política pública, né. Talvez nas universidades, mas acho que sempre tem que ter cuidado de não... bom, agora vamos todo mundo vai preencher um questionário e os que derem tanto vai pra um lado, os que derem vão pro outro. E daí vira uma, uma... forma de discriminar. Mas se é uma ferramenta que dá algum retorno, estamos todos precisando dela.

Referência 3 - 0,04% Cobertura

Eu acho que eu te dei aí uma lista.

<Arquivos\\ > - § 4 referências codificadas [0,75% Cobertura]

Referência 1 - 0,26% Cobertura

Porque me toca essas coisas, assim, ali do que fala, do que fala, né. E principalmente a questão do relacionamento com mãe e pai. Ali, que eu acho que é importante, a questão ali do que acontece comigo. O que pode acontecer comigo ali. Eu acredito que o jovem de hoje responderia, não mentiria. Não teria por que mentir, as perguntas objetivas, claras e trazem um... Traz, assim, ummovimento para a gente poder se avaliar e pedir ajuda.

Referência 2 - 0,16% Cobertura

Moderador: Sim. E pra... Tu acha que o ideal é responder sozinho, o adolescente sozinho, ou com um profissional junto que possa ajudar ele a entender melhor o que acontece?
Entrevistado: Eu acho que poderia ser com um profissional junto também, né. De confiança.

Referência 3 - 0,20% Cobertura

Moderador: Alguém da escola, assistente social...
Entrevistado: Isso, isso. Eu acho que é importante. É uma demanda, né. Porque, daí, tu vai para um público alvo de x número, mas traz retorno porque às vezes é uma pontinha, né. Uma pontinha ali do... Quem que... Como é que foi estipulado isso aí? Essas perguntas já vieram do...

Referência 4 - 0,14% Cobertura

Enfim. Esse, esse questionário aí, o adolescente também pode responder sozinho, claro, ele tem autonomia e tem todas as condições, né. Mas é importante também, assim, às vezes ter um monitor, alguém, assim, que possa poder auxiliar.

<Arquivos\\ > - § 4 referências codificadas [3,62% Cobertura]

Referência 1 - 0,40% Cobertura

Por que se ele veio de uma consulta, vamos supor assim, uma consulta com psicólogo, ou uma consulta com um médico de família. Ah, fulano, eu vou te dar um questionário para tu responder, ele está dentro de um consultório. Ele vai ter medo de responder. Ele vai responder aqui o que tu queres ler, ele não vai responder aqui o que está sentindo de coração.

Referência 2 – 1,34% Cobertura

Tá, vamos supor que seja PSE. Vai ter em alguns momentos algumas coisas que a gente vai trabalhar com esses alunos individualmente. Não no coletivo. No individual, falar com professora, hoje vou pegar a turma 13. Tem 21 alunos. Chama um por um, olha a gente está fazendo um trabalhinho, não sei o que, vou te deixar sozinha, tu responde isso aqui pra mim? É um momento dele, dentro de uma sala de aula, ele vai responder e eu acho que ele vai ser mais sincero, do que responder dentro de um consultório, ou do que responder no coletivo, por que daí o colega pode olhar o que ele está respondendo e dependendo do que ele responder, minha mãe é boa, é ruim, ah, se a mãe do fulano é ótima, a minha também é ótima. Nesse sentido então, ele tem que ser sozinho, mas não dentro de um consultório, nem num coletivo dentro de uma sala de aula. Existe uma forma de a gente fazer. Por exemplo eu levaria esse questionário pra [escola] e eu, nessa conversa que estou tendo com essas adolescentes, eu posso abrir uma pasta e dar para esse adolescente reponder. Tranquilamente, porque ele não me vê como médico. Ele me vê como um amigo. Que está tentando ajudar ele de alguma forma.

Referência 3 – 0,58% Cobertura

Te aconselho até de repente tu poder levar para os teus superiores, que isso também seja uma coisa que vocês possam dar para os agentes trabalharem nas suas comunidades ou com seus adolescentes, que nem todos os agentes trabalham em escolas, mas também tem adolescentes. Como eu, por exemplo, atualmente não estou trabalhando em escolas. Já trabalhei muito tempo em escola, hoje eu trabalho com os adolescentes só da Juliano Moreira. Mas sim, já fiz muito trabalho escolar. Tá. Deixa eu continuar lendo aqui.

Referência 4 – 1,30% Cobertura

É, isso que tu falou agora, dentro de um cenário, um cenário do profissional que conheça onde ele está pisando. Porque uma coisa é eu pegar esse questionário e fazer o questionamento com algum paciente no qual eu domino. Outra coisa é o outro profissional, que não conhece o meu paciente, que não conhece nada, querer mostrar esse questionário para ele, porque esse questionário vai ser bom, dependendo da forma que ele for abordado pra pessoa, para poder preenchê-lo. Dependendo do profissional que for passar, e se não souber passar, se não souber abordar, é muito ruim. Aí é uma coisa que vira até contra nós, dependendo. Ah, querem que eu faça umas respostas... Podem levar para os pais, com outro olhar, ai mãe, olha só, me perguntaram isso. E isso pode estar acontecendo dentro da casa mesmo. Mas a criança já vê com outro olhar. Não ve como o olhar da proteção, ele vê com um olhar assim – ó, tão dizendo que tu está fazendo isso, tão até pedindo pra eu responder um questionário. Aí, quando tu vê, vem a mãe aqui, perguntando quem são vocês pra estarem fazendo isso. Então em vez de a gente... vai ficar O criminoso, em vez de ajudar.

SCHOOL WORKERS (PE)

<Arquivos\\ > - § 2 referências codificadas [0,29% Cobertura]

Referência 1 – 0,19% Cobertura

Quem seria o profissional que poderia usar esse tipo de calculadora para identificar um risco de depressão? Geralmente são os professores, porque às vezes vem um relatório dos psicólogos e a gente pede para os professores. Porque ele está no dia a dia do aluno, tem mais convívio.

Referência 2 – 0,09% Cobertura

Ah, eu acho que é no lugar onde ele convive mais, que daí tem as pessoas que observam... Que pode ser vários lugares, não só na escola.

<Arquivos\\ 1> - § 4 referências codificadas [1,28% Cobertura]

Referência 1 - 0,20% Cobertura

Pois é. Eu fico me perguntando como é que seria a questão de sigilo. Considerando situações que garantissem sigilo, qual seria a viabilidade em termos de praticidade numa escola.

Referência 2 - 0,39% Cobertura

Escola grande em especial... Porque eu veria esses meninos e meninas preenchendo isso aqui num lugar mais reservado. E como é que uma escola... eu vejo isso na coisa de fazer entrevistas na pesquisa sempre. É tri difícil tirar os alunos pra um lugar que tu possa entrevistar porque as escolas só estão com os alunos quando eles têm que estar em aula.

Referência 3 - 0,39% Cobertura

Então teria que ser uma diretriz assim que desse condições para eles terem um momento mais reservado para isso. Mas eu vejo que a escola seria o lugar para isso, porque eu duvido que a gente pudesse ter acesso a essa população em outro lugar. Esse é o lugar em que eles estão, infelizmente não tem outros aparelhos do Estado que atendam eles...

Referência 4 - 0,30% Cobertura

Então eu acho que... tinha que ser no colégio, mas tinha que ter uma sensibilização envolvida sim sobre isso. Se tu distribuir uma coisa assim dentro da sala todo mundo vai querer olhar o do outro e não pensar no seu próprio. Vai causar uma certa dificuldade. Eu acho.

<Arquivos\\ 2> - § 3 referências codificadas [2,23% Cobertura]

Referência 1 - 0,99% Cobertura

Poderia ser feito no máximo duas vezes no ano. Ou uma vez em cada ano escolar. Independente do período. Acredito que nunca no primeiro trimestre. Porque no primeiro trimestre é a entrada, é a alegria, não tem.... mas a partir do... Poderia ser até no primeiro trimestre, mas não no primeiro mês, por exemplo, mas talvez próximo do primeiro resultado, logo após o primeiro resultado da escola. Eu acho que ele chega na escola e tem toda a novidade, tem os colegas, tem os desafios, tem a mudança de escola, ah, porque eu não vou ficar nessa escola, vou transferir, quero mudar de turma... Então tem todos esses... E aí acaba né. Acho que é bom meio do ano. Ou mais para o final do ano. Mas também, independente, dependente da visão de cada um. Mas que seria interessante sim ser feito pelo menos uma vez a cada ano. Independente do período, ah, se é possível fazer no primeiro trimestre, que se faça. Sem problema.

Referência 2 - 0,50% Cobertura

Eu acredito que teria que ter algum acompanhamento. Por exemplo, o adolescente faz esse teste agora, no primeiro ano do ensino médio, e alguém que acompanhasse essa caminhada, tivesse esse contato, acompanhasse, pelo menos por um ano. Ou nos próximos dois anos. Que ele chegasse lá no terceiro ano, no primeiro trimestre, ou no final do segundo ou terceiro, tivesse uma revisão, um olhar de algum adulto, algum técnico, um serviço de SOE, alguma coisa assim.

Referência 3 - 0,74% Cobertura

Acredito que não há necessidade, talvez algum desenho, alguma uma imagem criativa, e uma apresentação visual. Alguma coisa assim, ah, duas brincadeiras, Emoji, qual a carinha que identifica mais, aí ele aperta a carinha que ele está triste, entendeu, e aí já dispara, opa, como é que você se sente, tem cinco opções, aí, e agora, nesse momento, entendeu. E aí você faz duas situações dessas, já consegue identificar e levar pro texto de alto risco, porque ele vê a imagem, qual a carinha que você se vê melhor, se sente, né... Ou uma pergunta, também, alguma coisa que, onde você tá aqui e agora, o que é que você... como é que esse mundo reage em você. Se sente bem? Se sente mal?

<Arquivos\\ > - § 5 referências codificadas [1,05% Cobertura]

Referência 1 - 0,12% Cobertura

Dá para responder rapidinho, em menos de 5 minutos tranquilamente.

Referência 2 - 0,07% Cobertura

Eu acho que os adolescentes em geral.

Referência 3 - 0,04% Cobertura

Nas escolas.

Referência 4 - 0,45% Cobertura

Eu acho que a própria escola poderia... O professor teria que ser direcionado, né, instruído para fazer essa aplicação até porque o diagnóstico não viria do professor, né, teria que ter uma supervisão clínica, assim, de alguém que entendesse da questão. Mas eu acho que a escola poderia aplicar...

Referência 5 - 0,37% Cobertura

Eu acho que sim, porque se existissem dúvidas a gente não estaria preparado para respondê-las, porque não é a nossa preparação. Mas eu acho que sim, a escola poderia aplicar com uma supervisão de alguém que tenha conhecimento do assunto.

<Arquivos\\ > - § 8 referências codificadas [2,94% Cobertura]

Referência 1 - 0,13% Cobertura

Eu... Eu, sinceramente, nas escolas, as orientadoras educacionais, justamente porque está dentro dessa, desse tema, dessa área e trabalha especificamente com isso também.

Referência 2 - 0,15% Cobertura

Então, assim ó... Então, é interessante que nós pudéssemos ter esse material em
mãos também, até para ajudar a somar mais dentro do nosso trabalho na parte de conseguir ajudar esses adolescentes nas escolas.

Referência 3 - 0,25% Cobertura

E quanto mais profissionais trabalhando nessa área, como SOE, assim, que é importante, tiver desenvolvendo nas escolas, vocês vão perceber que menos, menos adolescentes problemáticos vão sair daqui, sabe... Vão se formando, vão trocando de escola, até chegar na vida adulta. Mas eles vão melhorando se tiver um profissional junto acompanhando.

Referência 4 - 0,72% Cobertura

O que acontece, assim, no processo do questionário, pode acontecer de algum adolescente não querer fazer. Por quê? Porque quando começa a falar a palavra "pai", "mãe", "pais", "família" tem adolescentes que são fechados a esse tipo de assunto. E às vezes para tu poder fazer com que ele seja sincero e responda esse questionário... Até ele pode até responder, mas não quer dizer que ele vá falar... que ele vá ser sincero, se ele não aceitar. Então, tem que existir uma conversa antes. Tem que existir um diálogo, uma preparação na cabecinha dele para que ele entenda que dentro da realidade dele o quanto é importante ele responder isso aqui e o que que pode ajudá-lo. Porque os adolescentes são assim também, eles, tudo o que eles fazem, eles quer saber qual vai ser o retorno, o que que vai favorecer eles, aí tem que existir, então, uma conversa por

completo, "olha, está sendo feito isso, o benefício vai ser esse, vamos fazer aquilo..." Porque pode ele se recusar a fazer.

Referência 5 - 0,61% Cobertura

Eu percebi que vocês não falaram sobre... Assim, eu não sei se antes de vocês aplicarem esse questionário vocês explicam que que é a depressão? Porque isso é importante. Porque, até então, vocês colocam aqui, "ah, você está demonstrando, devido às respostas que você fez, que existe um risco futuro de depressão." Mas o que é exatamente depressão, será que ele sabe? Aí, o que acontece, daí essa questão - se você responder positivamente, estão lhe causando sofrimento, é possível procurar ajuda. Você pode conversar sobre o sofrimento com algum adulto que você confia. Tá, depressão é um sofrimento, isso é óbvio. Mas quais são as, a identificação da doença, sabe? O que que é a depressão? Quais são os sintomas? Além do sofrimento, que ele está vivenciando... Porque o sofrimento também pode ser ocasionado por um rompimento...

Referência 6 - 0,32% Cobertura

Moderador: Então, tu vê mais o uso dessa ferramenta como um uso guiado, assim, com a ajuda de alguém que já...
Entrevistado: Isso, isso. Principalmente. Então, é... Tanto que até isso me fez me lembrar que o meu material também para bullying é um uso guiado, um material guiado. Eu passo um vídeo explicando o que é bullying, converso com ele sobre o que que é bullying e, no final, eu passo um questionário sobre bullying.

Referência 7 - 0,34% Cobertura

Moderador: Claro, claro, sempre um uso guiado. Então, um orientador, por exemplo, que pudesse mostrar para eles essa ferramenta e depois consegui pegar esses resultados e trabalhar com os adolescentes que apresentam maior ou menor risco.
Entrevistado: É, se não for orientador, até um profissional dentro da área, como um psicólogo, que chegue para passar esse trabalho, explique para eles exatamente, mostre vídeos, explique exatamente o que é essa doença.

Referência 8 - 0,42% Cobertura

Então, é uma forma de eles entenderem primeiro, pesquisarem, saber o que que é essa doença, o perigo que causa essa doença, como é que ela chega nas pessoas. O risco final que ela causa se não é tratada. O que que pode acontecer. Existe um diferencial de pessoa para pessoa. Então, assim, tem uns que chegam na loucura e vão parar dentro de sanatórios, tem uns que chegam na loucura e tiram a própria vida. Sabe? Então, até que ponto essa doença, o máximo dela vai, o mínimo. Então, eles têm que entender e saber, conhecer para depois responder o questionário.

<Arquivos\\ > - § 6 referências codificadas [5,07% Cobertura]

Referência 1 - 0,68% Cobertura

Eu vou te dizer que, quando eu respondi, eu pensei no padrão de adolescentes. Não pensei num adolescente específico que tinha a depressão, pensei no padrão, porque tem adolescentes lá que são abusados, tem adolescentes lá que sofrem violência. Mas a média é mais ou menos essa, eles vivem... Para mim, da realidade que eu vim, eu vejo um contexto de violência onde para eles as coisas são normais. Entendeu,
brigas, berros, entre pai e mãe. Eu acho que a pesquisa, o

Referência 2 - 0,17% Cobertura

Pois é, eu pensei assim, ó. Por exemplo, ele está online? O adolescente poderia chegar e fazer essa pesquisa?

Referência 3 - 1,18% Cobertura

É, não é uma coisa que um adolescente vai ter muito interesse, não acho que vá ter um alcance, assim, seria legal ter, por exemplo, vamos supor que pessoas que trabalham com um grupo de adolescentes poderiam

fazer para pensar sobre aquilo, problematizando. Se houvesse uma problematização, um trabalho contextual com o aluno, algum projeto em relação a isso... Onde tu dá suporte de busca, onde... Ali eu vi que fala do CVV, que tem um manual para os alunos se entenderem, legal. Mas eu acho que é muito mais útil, por exemplo, vamos supor que tem uma pessoa, que outra pessoa, pegue o adolescente e comece a conversar com ele. Porque talvez ele não tenha se dado conta e, às vezes, quando a depressão realmente está instalada num adolescente, ele precisa que alguém pegue pela mão. Sozinho é muito difícil ir, sabe.

Referência 4 - 1,32% Cobertura

Entrevistado: É, só o instrumento não. Mas vamos supor... Que alguns professores recebessem um treinamento de como trabalhar com os alunos. Poderiam problematizar a situação... Vamos supor que partisse do tema bullying, por exemplo, que é uma coisa bem comum, que tem que ser trabalhado o tempo inteiro na escola. E não preciso ser trabalhado esse tema. Como depressão, não precisa ser trabalhado esse tema. Tu pode trabalhar, por exemplo, o contrário, a valorização da vida. Trabalha com a valorização da vida e aí entra nessas questões de uma outra forma porque se tu deixar aberto, eles vão falar. Os alunos vão falar, vão trazer coisas, e aí poderia, sim, ser feito o teste e aí conversar, alguém conversa sobre os resultados e a pessoa que tem acesso a esses testes, de repente, chama esses alunos específicos para conversar, conversa com as famílias junto, porque o adolescente não... Precisa da família, sozinho não vai.

Referência 5 - 1,02% Cobertura

Então, tem que orientar. Aproveitei para orientar ela, conversar com ela. Liguei pra escola, falei com a coordenadora o que tinha acontecido, falei que eu fico assustada de uma criança de cinco anos ter esse acesso, às vezes nem os pais viram que a criança está vendo aquilo. E aí, eu vejo, assim, as crianças estão expostas - mesmo a minha filha, que não vê TV a cabo, está exposta por quê? Porque convive com várias outras crianças. As situações vêm. O que importa é como isso é orientado, organizado. Se isso é feito numa criança de 5 anos, acontece isso com uma criança, tu imagina com adolescentes com acesso a tudo o que é informação, que os pais saem de casa para trabalhar e veem a TV, tudo na TV.

Referência 6 - 0,69% Cobertura

Não tem um trabalho. Então, tem que ser, tem que ter uma supervisão, sim. Isso eu digo que às vezes gera confusão: adolescente não é adulto. Adulto é responsável pela sua vida. Criança, não. Adolescente, não. Adolescente está em construção. E, às vezes, não é visto assim como uma fase de transição e é. Eles ainda dependem do adulto que tem que autorizar eles para muitas coisas, para eles se sentirem autônomos, mas estão construindo essa autonomia, eles não têm ela ainda.

<Arquivos\\ > - § 6 referências codificadas [1,17% Cobertura]

Referência 1 - 0,05% Cobertura

Achei bem, bem, bem acessível, calculadora para o risco de depressão, que... Deu, né, aqui terminou?

Referência 2 - 0,30% Cobertura

Eu vejo usando, assim, adolescente, pré-adolescente de 12 anos já seria um bom início. Seria aceito, assim. Eles vão entender, vão compreender. É de fácil, assim, a gente entende o que que querem perguntar, acredito que eles também, eles são bem espertos. Nessa faixa etária de 12 anos, já é um bom início, assim, já dá para aplicar o questionário. E coisas para serem... Agora, assim, não me vem uma pergunta, algum questionamento mais, mais... Ou como, a forma como a criança enxerga, tipo assim, uma pergunta. Qual é a forma que você enxerga um abuso? Ou o que você entende? Claro, daí ele vai ter que responder, né, aí não...

Referência 3 - 0,39% Cobertura

O desafio, talvez, é a resistência de não responderem, né, a resistência em como vocês vão abordar também, como é que vocês vão aplicar, mas o que vocês vão falar pra não... Não é tipo constranger, é para não reprimir... Não é reprimir também, é uma palavrinha, para não inibir a criança de responder. Um questionário, usando outras palavras, que não seja depressão, essa coisa. Né, de ter uma estratégia de como chegar na

criança para ela responder. Isso seria... O obstáculo é a resistência, daí, a resistência dependendo do que vai ser falado pra eles. "Ó, pessoal, eu vou chegar aqui, eu sou da [nome da universidade], eu quero que vocês respondam, assim, assim, assado", acho que isso é importante. Aquela fala ali, tu tem que também comover eles. Ou do tipo, assim, ah, responde quem quer. Aí, já acabou, ninguém vai querer, isso não.

Referência 4 - 0,09% Cobertura

É. Então, essa parte deles escolherem, acho que não existe, é sim e sim - olha eu, bem democrática. Vamos responder. Eu já faria isso. Então, aqui, vou distribuir. E mais é na abordagem.

Referência 5 - 0,25% Cobertura

Que horror isso, né. Tem que mudar isso, gurias. Porque eu sei que é pra um bem para eles. Pode, pode se organizar. Não vai sair, não pode ir no banheiro, toma isso aqui e responde. É assim que eu falo. Mas tem que ser ali, ó. E a resistência deles, assim, também, e para a criança de 12 anos, eles podem ter uma dúvida. Bobinha. Mas aí a pessoa vai "ai, não, tu tem dúvida nisso aqui, guri?" Não assim, né. Daí já desmontou a criança. Porque muitos profs têm essa atitude, assim, de responder já enfatizando que o guri nem...

Referência 6 - 0,10% Cobertura

Moderador: Sim, já com uma punição, né.
Entrevistado: É, então, essa forma, assim, que eu vejo, essa resistência deles... E também, como vocês mandaram para os pais, lembra? Eu não sei se os pais leram.

<Arquivos\\ > - § 3 referências codificadas [0,84% Cobertura]

Referência 1 - 0,35% Cobertura

Interessante, muito bom. Pronto. Eu vou te perguntar, sabe o que seria bom vendo isso, já que estão falando que vai ter psicólogos nas escolas, toda escola pública deveria, a Secretaria de Educação deveria já saber e ter isso, por que ao iniciar a aula, ou se o aluno é novo e chega na escola, a gente já poderia prever com que eles respondessem. Seria bem interessante.

Referência 2 - 0,15% Cobertura

Seria interessante que cada escola pudesse ter e cada adolescente novo que chegar, a gente colocar pra eles responder, já poderia observar, ter um olhar diferente.

Referência 3 - 0,34% Cobertura

Do jeito que hoje em dia existem tantos aplicativos, tantas coisas, porque não? A calculadora, quem sabe um dia ela pode se tornar um aplicativo onde exista de plantão, da mesma forma que existe os plantonistas, tu liga porque tem um incêndio, porque não pode haver alguns profissionais prontos para atender alguém que realmente está pensando em tirar sua vida.

<Arquivos\\ > - § 2 referências codificadas [0,80% Cobertura]

Referência 1 - 0,32% Cobertura

Pois é, não, acho que... Eu penso que pros jovens seria tranquilo, até porque de uma forma geral se, né, gosta de responder questionário e tal. Não sei se aquele da maconha... não corre um risco deles dizerem que não... mais do que, sabe? Talvez pudesse incluir uma fala mais genérica, talvez junto com a bebida, sabe, ou outras drogas. Claro, que às vezes ele só usou álcool e não usou maconha. Mas não sei ali se não ele se constrangeria, mesmo que seja uma calculadora...

Referência 2 - 0,48% Cobertura

Que ninguém vai ver, só pra ele. Sabe? Porque... às vezes eu penso que é difícil, me lembro, né, de mim quando era jovem também, se alguém tivesse que, "Ah, mas eu vou dizer isso (?) que que vai ser" e tal. Então, não sei se aquilo ali às vezes... mas isso também sou eu, né. Que tem uma realidade completamente diferente, claro, pensando no jovem ali da minha realidade, talvez um jovem classe média, né, numa outra condição talvez não se importe tanto. Ali não sei se não causaria algum constrangimento, mas... eu acho que das outras perguntas... É, eu penso que foi bem adequado, perguntou das relações com o pai, com a mãe, com a família. Se já sofreu alguma agressão, se já faltou, né... comida, alimento.

HEALTH WORKERS (PS)

<Arquivos\\ > - § 2 referências codificadas [0,21% Cobertura]

Referência 1 - 0,07% Cobertura

The professional who specializes in depression.

Referência 2 - 0,14% Cobertura

In which places would the use of such a tool be most I don't know, I believe everywhere.

<Arquivos\\ > - § 4 referências codificadas [1,94% Cobertura]

Referência 1 - 0,50% Cobertura

Os itens fazem muito sentido. Mesmo essas questões de traumas na escola ou a possibilidade de relacionamento com os amigos... E outras experiências aí, também, envolvimento com substâncias, né, com álcool e maconha. Acho que, sim, faz muito sentido. E acho que é bastante fácil, assim, de ser aplicado. As perguntas não são de difícil entendimento, não é um instrumento muito longo, então...

Referência 2 - 0,16% Cobertura

Eu acho que mais específico, talvez. Eu fico preocupada que, num contexto mais geral, isso seja visto como um outro testezinho.

Referência 3 - 0,72% Cobertura

"Qual a cor, sei lá, que fala sobre você? Sua cor favorita fala sobre você.", né... "Responda às perguntas para saber qual personagem da série tal você se parece" e que, no final, a mensagem não seja levada tão a sério, assim. Isso me preocupa um pouco. Eu penso, tipo assim, no contexto de rede social, vamos dizer, como se isso pudesse ser visto de uma forma menos importante. Talvez se isso aparecesse em circunstâncias mais selecionadas, quando a gente tivesse, assim, adolescentes que buscassem algum recurso. Mesmo que não fosse um recurso de tratamento...

Referência 4 - 0,56% Cobertura

É. Mas que buscassem, sei lá, o serviço médico de uma forma geral... Ou que buscasse o grupo da igreja de uma forma geral... Acho que aí já tem uma possibilidade de se entrar de uma forma mais, com pessoas mais preparadas para responder mesmo que elas não estejam em risco. Mesmo que elas sejam de baixo risco, vamos dizer. Mas que estão um pouco mais com a cabeça... Ou com uma possibilidade do tema ser introduzido de uma forma mais...

<Arquivos\\ > - § 2 referências codificadas [0,52% Cobertura]

Referência 1 - 0,41% Cobertura

Moderadora: Acho que assim, pensando, assim ó, quem tu pensa, como que a gente poderia e quem tu pensa que poderia responder isso aí? Como eu te falei, a gente...
Entrevistado: Acho que estudar isso em escola... seria um bom...

Moderadora: Tu vê a escola como uma possibilidade?
Entrevistado: Aham, acho que ensino médio, a partir do primeiro ano já dá

Referência 2 - 0,11% Cobertura

Acho que explicar pra ele que é um instrumento que pode avaliar se ele tem risco pra ter depressão.

<Arquivos\\ > - § 2 referências codificadas [1,20% Cobertura]

Referência 1 - 0,10% Cobertura

Tu já apontasse uma situação já de leitura do escore pelo adolescente, a necessidade de ter uma orientação, né.

Referência 2 - 1,09% Cobertura

Acompanhamento, assim, pra poder dialogar, porque ele pode se assustar dependendo de como ele realmente se encontra em termos de risco, ele pode se assustar com a informação. A informação, ela precisa ser livre, acessível, né, completa pra todo mundo. Mas em se tratando dessa faixa etária, ter companhia é fundamental pra poder falar sobre isso, né, pra acompanhar, pra situar, "Olha". E se a gente remete, remete, né, imediatamente já com enfoque na doença - eu acho que essa é uma grande questão, né, o nosso enfoque em geral tá sendo a doença, né. A gente vem direcionando - claro que é o que nos interessa, a gente quer cuidar disso - mas o quanto tá difícil pensar nas potencialidades, naquilo que a adolescente ou o adolescente podem e do quanto a gente não vai ficar sabendo, assim, do que ele mais gosta, do que ele sente falta, de quantos espaços ele poderia imaginar e que a gente nunca vai disponibilizar porque ele, não ouvimos o que elas e eles têm a dizer e porque não nos ocorreu. Sei lá, se tiver uma pracinha, uma praça muito legal no território, esse pode ser um espaço muito importante. Mas, né, tô dizendo uma coisa, assim, parece muito simples.

<Arquivos\\ > - § 6 referências codificadas [1,24% Cobertura]

Referência 1 - 0,23% Cobertura

Quem estivesse treinado - vou dar uma resposta genérica, mas eu não posso dizer 'ah, os psicólogos'. Porque é um psicólogo treinado a lidar, que a gente sabe que são várias especialidades onde não dá conta. Então acho que pessoas treinadas.

Referência 2 - 0,01% Cobertura

Todos.

Referência 3 - 0,52% Cobertura

Genérico também, mas é uma proposta inclusive em que eu trabalhei no doutorado que poderia treinar com o uso de um manual várias pessoas, várias áreas. Então a ideia de que qualquer um que tiver contato com adolescentes poderia ser já aberto a identificar, quanto mais, melhor. Então acho que a gente fechado num segmento só porque eu sei que vou saber usar mais, isso limita demais, e às vezes o adolescente não vai ter contato só com psicólogo, só com psiquiatra. A escola é uma fonte que necessariamente está ali, o adolescente tem que ir para a escola.

Referência 4 - 0,21% Cobertura

Não, acho que não. Porque daí essa calculadora seria uma ferramenta segura, eficaz. Um problema é que daí todo mundo, todo mundo tem depressão e fica aquela coisa - eu acho que quem estiver habilitado a usar calculadora –

Referência 5 - 0,02% Cobertura

E treinado.

Referência 6 - 0,25% Cobertura

Acho que não, porque acaba que as pessoas identificam o comportamento isso vira uma bola de neve muito pior do que ser explicado como um diagnóstico. Tendo um diagnóstico e o que a pessoa precisa saber sobre o diagnóstico, porque ela não precisa saber de tudo.

<Arquivos\\ > - § 2 referências codificadas [0,58% Cobertura]

Referência 1 - 0,26% Cobertura

Qualquer pessoa adequadamente preparada para fazer uma mea culpa, seria fazer um pensamento crítico sobre se aquilo realmente se aplica ou nao e se realmente não é uma falha do sistema de apontar uma coisa que nao é real. Acho que uma vez a pessoa tendo formacao e conhecendo o assunto, pode ser tipo um guideline, uma coisa que realmente ajude.

Referência 2 - 0,32% Cobertura

Em um primeiro momento eu ia sugerir as escolas, porque acho que é onde eles estao o tempo todo. Tem que ter um cuidado pra nao supervalorizar os resultados dela, teria que ter o ponto de vista crítico. Deixar bem claro que aquilo seria uma triagem, que nao é nada que defina o diagnostico sozinho, que é só uma suspeita. Já tem aplicativos pra celular que fazem esse tipo de coisa. Os adolescentes costumam gostar também.

<Arquivos\\ > - § 2 referências codificadas [0,96% Cobertura]

Referência 1 - 0,72% Cobertura

Unidade básica. Acho que como ajuda... Assim, hoje em dia o portal SUS, né, o sistema de telessaúde, ele já tem, por exemplo, os enfermeiros já têm acesso aos SNAPS, que é aquela escala já autorizada para avaliar déficit de atenção e hiperatividade e oposição, que se entrega, o próprio enfermeiro ou o pediatra imprime, entrega pra família e a família entrega um na escola, um para a mãe, um para a avó e as pessoas preenchem a escala e aquela escala é necessária para o encaminhamento para um neuropediatra. Então, assim, isso é focado, o lugar disso no sistema de saúde seria a unidade básica. Daí, talvez, com essa adaptação desse futuro mais próximo, assim, né, e daí não poderia ser anônimo, né, nesse sentido. A não ser que tenha só uma orientação ali, se for para um uso do sistema de saúde, precisa ser, não, essa... Essa menina está, eu estou aqui com a Fulana, ela vai preencher, deu risco pra depressão, eu vou encaminhar ao pediatra para uma avaliação melhor ou eu vou fazer uma entrevista de saúde mental e vou ver se já mando ou não para atendimento. Aí, não pode ser para fins de pesquisa anônima, né, tem que ser já para educação e prevenção.

Referência 2 - 0,24% Cobertura

Não. A escola poderia ter, mas, assim, a escola, daria uma responsabilidade à escola que eu não sei o que eles fariam com isso, né, porque como eu te disse, acho que o mais importante da escala, do questionário é que a consequência do risco futuro, nenhum adolescente deve conseguir seguir se ele já está deprimido, né, que é procurar o CVV ou procurar o portal para ter informação.

<Arquivos\\ > - § 2 referências codificadas [1,52% Cobertura]

Referência 1 - 0,60% Cobertura

Acho que algo mediado. Mediado. Porque talvez uma... Se eles tivessem isso aberto, que eles fossem para, não sei, a gente precisaria pesquisar sobre isso, como o filme, né, porque o filme foi feito, na verdade, pensando em prevenção da questão do suicídio. E quando eu vi essa pesquisa e diz que ela se assemelha a pesquisas de outros locais, me surpreendi, porque, na verdade, ao contrário, eles tinham mais vontade de fazer exatamente aquilo que tava ali e que era a falta de alternativa. Então, exacerbou aqueles sinais eu acho, não sei, acho meio complicado, teria que conversar mais sobre isso.

Referência 2 - 0,91% Cobertura

Se os professores forem capacitados, eles podem estar mediando. Eu acho que sim, acho que os professores das escolas podem estar mediando isso. Claro, grupos de pesquisa podem estar fazendo, mas os professores sendo capacitados eu acredito que sim, sempre de modo muito responsável, não fazer aleatoriamente, toda hora estar aplicando, sabe, que no fim se torna também algo que não é digamos positivo e os adolescentes eles respondem aquilo que tu quer, também, a gente precisa ter um pouquinho de cuidado nesse sentido. Por isso, eu penso nos professores porque eles conhecem os adolescentes que têm, na sua grande maioria. Então, quando eles veem essas respostas, eles vão saber se aquilo está sendo real mesmo, eles conhecem o contexto desses adolescentes, ou se não, se ele tá respondendo àquilo que eu quero que ele responda ou que... Eles sabem o que é correto. Então, esse é um cuidado que se tem também.

<Arquivos\\ > - § 10 referências codificadas [2,60% Cobertura]

Referência 1 - 0,41% Cobertura

É que tem coisas aqui que mesmo, o próprio adolescente, né, essa questão se alguém já tentou fazer algumas coisas sexuais com você, é ele mesmo, né, porque outra pessoa respondendo, ele vai tentar negar ou dizer que não, não foi bem assim, não foi isso que eu te disse, né. Que isso aqui é bem específico pro, pra própria pessoa envolvida responder. Essa questão tudo é mais o eu, mesmo, me coloca na situação do eu.

Referência 2 - 0,13% Cobertura

A vantagem, eu acho que é de identificar. Só que assim, o que eu penso é: em que momento se aplica, onde se pode aplicar isso?

Referência 3 - 0,03% Cobertura

De repente numa escola?

Referência 4 - 0,56% Cobertura

De repente... porque a questão da família é, se a família nega, não vai nem procurar instrumento. Mas se existem informações, se existem projetos de, de informações sobre saúde mental, pelo menos a pessoa vai olhar, né. Aparece, de repente, na mídia, em algum lugar... ou até na televisão. A pessoa vai se atentar pra isso, né, opa, existem questões de saúde mental que a gente pode ver. Será que se aplica aqui em casa? Aí vai buscar, mas aí onde buscaria? Posto de saúde, escolas, clínicas.

Referência 5 - 0,47% Cobertura

Ele respondendo... Acho que sim. Na escola eu acho que é o primeiro desencadeador. E aí eu te diria que seria até interessante, de repente, se... pegar uma faixa etária, tipo assim, adolescência: o quê? Começa com o quê? 10, 11, 12? Com que idade? Com tal idade, aplicar o termo, esse, esse, a calculadora. Tipo assim: você se encaixaria? Digamos em que série seria isso... na, na 7º? Que idade mais ou menos eles têm na 7º? Eles entram com 6 na primeira... 7 na segunda...

Referência 6 - 0,14% Cobertura

E reaplicar, se necessário for, conforme forem os resultados, reaplicar no no segundo semestre ou no início do próximo ano, né.

Referência 7 - 0,41% Cobertura

É, acho que sim. Porque eu acho que a, a... a depressão é a doença do momento, né. Ela é silenciosa, porque, ok, o câncer mata, mata mais ainda, na atualidade ainda é o câncer que mais mata, né. Mas, mas a depressão tá muito perto, né. Tá muito perto e as pessoas, no geral, parece que tem um preconceito maior com questões mentais, né. Então procura recursos mais tarde. Só vai procurar quando realmente aperta.

Referência 8 - 0,16% Cobertura

Eu acho que antes de aplicar o termo teria que se fazer tipo uma aula básica sobre, sobre saúde. Eu não sei se nas escolas tem, atualmente...

Referência 9 - 0,09% Cobertura

Adicional. Acho que sim. Acho que aplicar avulso, sem eles terem a noção...

Referência 10 - 0,19% Cobertura

Essa preparação, exatamente, do que que ta envolvendo esse, esse, essa calculadora, eu acho que é uma coisa em si. Tem que ter uma preparação, uma colocação, um olhar para a saúde mental.

<Arquivos\\ > - § 1 referência codificada [0,09% Cobertura]

Referência 1 - 0,09% Cobertura

Desde que a gente se comprometa, mas isso são outros quinhentos. Desde que a gente se comprometa com os encaminhamentos, com os tratamentos adequados...

<Arquivos\\ > - § 1 referência codificada [0,52% Cobertura]

Referência 1 - 0,52% Cobertura

Eu acho que adolescentes é, assim, é a questão de na sala de aula, pedir para baixar e responder, né. Mandar para eles responderem depois e tal, eles não vão. Então, é uma coisa, assim, meio imediata. Vai lá, divulga, já pede para entrar... Por isso que eu pensei até num aplicativo, uma coisa que eles entrassem... Ou passa o link e eles já respondem... Porque se vai deixar para eles responderem em outros espaços, em outro momento, não vão responder.

<Arquivos\\ > - § 1 referência codificada [0,64% Cobertura]

Referência 1 - 0,64% Cobertura

Em relação ao conteúdo e aplicabilidade, eu acho que essa foi uma preocupação bem legítima nossa quando a gente estava pensando e preparando esse instrumento no sentido de que a gente pensou em algo simples, barato, que pudesse ser aplicado por praticamente qualquer profissional, não necessariamente um psicólogo, um psiquiatra ou alguém ligado à saúde mental, e isso porque a gente pensou que um instrumento de screening, um instrumento para identificar risco, tem que estar nas escolas, nas UBS, quando for usado numa perspectiva do mundo real.

USERS (US): PARENTS

<Arquivos\\ > - § 2 referências codificadas [0,67% Cobertura]

Referência 1 - 0,28% Cobertura

Entrevistado: Bom, em escolas.
Moderador: Escolas, aham.
Entrevistado: Em cursinho, sabe, onde se apresenta maior quantidade de adolescentes, né, que está em risco hoje em dia.

Referência 2 - 0,40% Cobertura

Isso, isso. Seria bom também... Acredito que ou anos iniciais, eu acho que a escola engloba tudo, mais ou menos, assim, né, para os adolescentes, a gente encontra ali uma faixa etária dos 7 aos 18, vamos dizer assim. Então, é bem... É isso acho que mais, na escola, e...

<Arquivos\\ > - § 2 referências codificadas [0,51% Cobertura]

Referência 1 - 0,15% Cobertura

Ah, acho que poderia ser na escola, ensino médio, acho.

Referência 2 - 0,36% Cobertura

Sim, eu creio que poderia... Poderia ser tipo uma palestra preparatória, assim, né, e posterior explicar... Dizer que eles responderiam a essas coisas aqui. Situações..

<Arquivos\\ > - § 1 referência codificada [0,70% Cobertura]

Referência 1 - 0,70% Cobertura

Moderador: E tu consegue imaginar, assim, em que, em que circunstância a gente usaria isso, onde é que a gente aplicaria isso nos adolescentes?
Entrevistado: Em que circunstâncias... Eu acredito que em colégio.
Moderador: Numa escola?
Entrevistado: É, numa escola. Que é onde mais tem... Talvez tenha o Fulaninho lá no canto, que não conversa muito tempo, não converso com ninguém, conversa pouco, só tem um professor, então, é legal chamar todos para fazer uma avaliação assim, né.

<Arquivos\\ > - § 3 referências codificadas [0,65% Cobertura]

Referência 1 - 0,37% Cobertura

Fazer um adolescente preencher um questionário assim...mas de repente seja até mais atrativo para eles, porque é tecnológico, o que é atrai mais eles.

Referência 2 - 0,25% Cobertura

Você acha que os próprios adolescentes usando seria tranquilo?
Entrevistado: Acho que seria tranquilo sim.

Referência 3 - 0,03% Cobertura

Nas escolas.

2. Code Query Output – Ethics

POLICY MAKERS (FPP)

<Arquivos\\ > – § 1 referências codificadas [0,93% Cobertura]

Referência 1 – 0,93% Cobertura

Sim. Eu fico pensando assim qual seria a conduta, como modificaria a conduta do profissional de saúde perceber que um paciente adolescente está com um alto risco, né. Por exemplo, em avaliações de risco cardiovascular ou tabagismo, a partir de escores a gente tem condutas já meio protocolares, já meio definidas, que tipo de abordagem. Talvez fosse importante ter, quanto tu recebe essa certificação... Uma coisa... É que é a orientação para o próprio adolescente, não sei se a pergunta é mais para o adolescente ou para o profissional. Pensando no meu uso, como profissional na prática, eu pensaria em que... Que atitude tomar como profissional agora na avaliação desse adolescente? Eu tenho que talvez ter consultas mais regulares, obter uma avaliação familiar... Ou, aí sim, fazer uma visita à escola ou a escola poder ter um espaço conjunto.

FOCUS GROUP: PARENTS

<Arquivos\\ > – § 2 referência codificada [0,56% Cobertura]

Referência 1 – 0,32% Cobertura

Isso. Se eu coloco na sala de aula que a gente, claro, por uma questão de escala, numa escola tu consegue abarcar um número maior, né, mas a qualidade não seria tão apropriada quanto os profissionais da saúde. Com certeza iriam emergir questões da saúde que os profissionais iam ter condição de responder. E, em sala de aula, os professores não, não é a área. Professor de Matemática falar de anatomia? Né, que é o que vai vim, questões sentimentais, sexualidade, é o que vai vim. Que chama a curiosidade dos adolescentes, acredito eu, que é o que é atrativo a eles, né, que é essa fase, assim, do descobrir o beijo, transar, aquela coisa toda, e eu acho que o profissional da saúde tá mais apropriado.

Referência 2 – 0,12% Cobertura

Mãe 5: Vamo tratar.
Mãe 2: Vai mexer com a cabeça dele. (inaudível)
Mãe 1: Mas eu não sei se a ideia era trazer pra ele primeiramente, pra esse adolescente, que ele foi supostamente diagnosticado com possíveis possibilidades de depressão.

Referência 3 – 0,04% Cobertura

Mãe 5: Eu acho que tem que falar sim. Eu acho que tem que falar sim "ó, tu não tá bem".

Referência 4 – 0,08% Cobertura

Mãe 2: O meu diria, "Ah, achei frau". É a primeira coisa. Ele diria, "Ah, falaram tal coisa, que frau, né, mãe?" Que é o que ele diz, "que frau". O que que é "frau", [nome do adolescente]?

SOCIAL WORKERS (PAS)

<Arquivos\\ > – § 1 referência codificada [0,35% Cobertura]

Referência 1 – 0,35% Cobertura

Quais os riscos envolvidos ao usar uma ferramenta como essa?
Moderadora – Eu penso que é de caso a caso, às vezes tem um protocolo geral, sendo que determinados adolescentes sofrem com coisas específicas.

<Arquivos\\ > - § 1 referência codificada [0,90% Cobertura]

Referência 1 - 0,90% Cobertura

Entrevistado: Eu acho que, por exemplo, num espaço de proteção, por exemplo, no ambiente escolar, seria muito útil, assim, algo que não tivesse exposição, não tivesse exposição assim porque a gente sabe que tem muitas, muitas, mesmo tu estando dentro do problema ou da dificuldade, né, passando por aquele contexto... Alguns não gostam de expor, assim, se for um espaço mais, mais sigiloso, algo mais protetivo, assim, algumas... Algumas das crianças, dos adolescentes conseguem expor mais essa situação, assim, talvez também, em determinado momento, este questionário, assim, calculadora ela serve uma, ela serve, assim, como um pedido de socorro, assim. Ela serviria, né, como pedido de socorro, assim, bom, talvez ninguém detectou aquilo, ou seja, o início de um problema depressivo... Eu acredito que isso seria muito bem utilizado.

<Arquivos\\ > - § 1 referência codificada [1,02% Cobertura]

Referência 1 - 0,58% Cobertura

Com certeza. Daí de repente aqui, vamos supor, aqui, ó, alguém já tentou fazer coisas sexuais com você contra sua vontade, te ameaçando ou te machucando? Ele bota, vamos supor, não, e daí o profissional, vamos supor eu, posso ler de novo e ele pensar. De repente porque ele estava com outra pessoa e não quis responder. Ou se a mãe viu e ele não pode responder. Porque isso aqui não vai pra tua pasta, não vai pra nada. Isso aqui é só pra uma pesquisa, mas a gente vai tentar te ajudar, perante essa pesquisa.

Referência 2 - 0,44% Cobertura

Moderadora: Tipo uma campanha para os adolescentes fazerem. Mas tu acha que teria algum problema, por exemplo, um adolescente que recebesse a classificação de alto risco, tu acha que teria algum problema, isso geraria algum desconforto, ou algo assim?
Entrevistada: Não, porque o outro não vai ver.
Moderadora: No sentido então de ter um resultado individual assim, só pra ele.

<Arquivos\\PAS-2> - § 3 referência codificada [1,21% Cobertura]

Referência 1 - 0,29% Cobertura

Seria nas escolas. Do treinamento e atendimento de quem aplica, né. Porque daí, "ah, então ele tá sendo vítima de abuso." A gente já teve situações, assim, que essa questão do abuso veio depois qualquer coisa que aconteça com a criança, "ah, é porque..." A escola, sem querer, vaza a informação, daí os outros alunos falam, daí fica a situação pior, né. Então, tinha que ver isso aí, com um treinamento bom, assim, não divulgar.

Referência 2 - 0,56% Cobertura

Moderadora: E tu veria o uso desse instrumento, então, que te daria essa... Por exemplo, o adolescente responderia ou com o profissional ou sem o profissional, enfim, e te daria essas duas possibilidades de risco ou sem... Ou sem o risco, como tu viu ali. Tu veria isso aplicado só em situações de consultório, de posto de saúde, tu veria outro uso para essa, para esse instrumento? Por exemplo, numa escola, numa campanha...
Entrevistado: A única... Uma das... Eu acho interessante, poderia, só que tem que se cuidar muito, porque em escola, principalmente, tem umas perguntas ali, por exemplo, na família, se alguém já te tocou, se alguém já te bateu, que às vezes ela é espalhada pela escola. Então, tem que ter um treinamento, uma orientação porque muitas vezes a família vai lá, explica a situação daquele adolescente, daqui a pouco tá todo mundo sabendo.

Referência 3 - 0,08% Cobertura

É, porque tem as respostas ali que poderiam, né. As pessoas comentam, então, é bem complicado, assim

Referência 4 - 0,28% Cobertura

Seria nas escolas. Do treinamento e atendimento de quem aplica, né. Porque daí, "ah, então ele tá sendo vítima de abuso." A gente já teve situações, assim, que essa questão do abuso veio depois qualquer coisa que aconteça com a criança, "ah, é porque..." A escola, sem querer, vaza a informação, daí os outros alunos falam, daí fica a situação pior, né. Então, tinha que ver isso aí, com um treinamento bom, assim, não divulgar.

<Arquivos\\ > - § 3 referências codificadas [1,17% Cobertura]

Referência 1 - 0,45% Cobertura

Eu acho que o principal é não ser identificado. Para que tu possa ter respostas mais claras. Talvez vocês possam deixar é um campo que vocês podem... Porque geralmente a gente consegue saber... Se tu direciona ele para ser aplicado numa ferramenta, eu não sei se pode se criar um dispositivo em que tu possa identificar, quem é, digamos, esse adolescente que tem alto risco, ele possa ser identificado. E aí, dependendo das respostas, a escola pode acessar esse adolescente. Ou através de um espaço de conversa com os pais, ou alguma uma ferramenta mais, que possa permitir...

Referência 2 - 0,37% Cobertura

O uso que eu fiz já de questionário online para pesquisa, geralmente a gente consegue... Tu manda por e-mail, recebe a resposta, tu acaba muitas vezes sabendo, por e-mail tu acaba conhecendo quem é o sujeito da pesquisa. Mas aí entram aquelas questões éticas que tu não vai identificar. Agora, quando se trata de questões que envolvem adolescentes e que é um componente de cuidado e de proteção também é... É algo de responsabilidade da escola, dos pais e responsáveis.

Referência 3 - 0,35% Cobertura

Olha... Ela vai, talvez ela pode ser negativa para algum jovem é... Se ele tiver, se o jovem que responder tiver um componente depressivo já muito grave. Entendeu? É o único risco que acho que talvez, e mesmo assim, ele pode ter um componente e ser muito grave e não... Poder só... Poder dar-se conta de que isso é sério através do questionário. Ou... A única coisa... Igualmente, assim, é muito baixo o risco de que isso venha a acontecer, entendeu.

<Arquivos\\ > - § 2 referências codificadas [0,25% Cobertura]

Referência 1 - 0,16% Cobertura

Pode, pode vir coisas que o profissional não tá preparado pra ouvir. Eu sempre acho que o adolescente tá preparado, sabe, ele tá disposto a falar. Quem não tá é o profissional.

Referência 2 - 0,09% Cobertura

O profissional talvez não, não queira ouvir que ele foi abusado pelo pai, que é gerente do banco.

<Arquivos\\ > - § 3 referências codificadas [1,06% Cobertura]

Referência 1 - 0,60% Cobertura

Eu achei bom, assim, mas... O tamanho é bom, ok. É compreensível, também, mas eu acho que... Eu penso no caso dos meus alunos, se eles tivessem, eu acho que geraria muitas, sei lá, muitas inquietações. É que daí o vínculo já tá rompido, né, então se for pensar em alguém que ainda não rompeu o vínculo com a família, eu acho que talvez seria adequado isso. Mas... no meu caso, acho que seria, geraria muitos gatilhos, muitas coisas, sabe. As perguntas, porque são bem relacionadas ao que eles vivem.

Referência 2 - 0,32% Cobertura

Sim. Eu acho que poderia ser usada até como um instrumento, mas... esses dados coletados, pra depois cobrar que o atendimento seja mais, porque é muito restrito. Pros nossos que estão em abrigos já é difícil, imagina uma pessoa normal, né, que tem baixa renda e...

Referência 3 - 0,14% Cobertura

Eu acho que no adolescente poderia gerar alguma lembrança que não seria tão legal, mas também depende, né, de cada um.

<Arquivos\\ > - § 1 referência codificada [0,76% Cobertura]

Referência 1 - 0,76% Cobertura

Né. Quando, quando acende o sinalzinho vermelho, aquele, né, o radar diz ó, tá, a coisa tá ficando perigosa, então, ele poder ser o profissional e já pensar alguma ação efetiva, se vai atender com outros profissionais, se vai vincular esse adolescente a alguma atividade no posto de saúde, na comunidade pra ficar em observação, né, não perder esse adolescente do radar. Então. Agora, escola em primeiro lugar, acho que a política de saúde nos postos de saúde etc. E... não sei. Aí eu fico muito, pela minha profissão, eu fico muito vinculada à política pública, né. Talvez nas universidades, mas acho que sempre tem que ter cuidado de não... bom, agora vamos todo mundo vai preencher um questionário e os que derem tanto vai pra um lado, os que derem vão pro outro. E daí vira uma, uma... forma de discriminar. Mas se é uma ferramenta que dá algum retorno, estamos todos precisando dela.

<Arquivos\\ > - § 1 referência codificada [1,35% Cobertura]

Referência 1 - 1,35% Cobertura

Aí, vai a ética, né? Porque, daí, assim, ó, que que a gente se questiona: daí, eu digo, eu vou denunciar, eu vou denunciar a questão de maus tratos e daí alguém vai lá ver essas coisas na família, vai ter que aparecer. A professora, ela geralmente, ela faz a denúncia, ela detecta. Eu acho que é uma das melhores. Porque às vezes nós como profissionais, assim, com perdão da palavra, né, se caga. Porque daí, ah, depois eu tenho que ir para audiência, não sei o que. Quando as audiências eu já fui, de um tempo atrás, na cidade de [nome da cidade], bah... Tinha que ir e eu falava a verdade, né, a verdade que dói, é crua, mas, assim, precisava porque o promotor precisava tomar a providência, às vezes nem o conselho tomava a providência. Às vezes o próprio conselho, desculpa, se cagava. Sabe? Que tá indo na comunidade, então, porque... É, isso daí, assim, parece que fica a responsabilidade do profissional da saúde, parece que nós da saúde mental temos que dar conta, tá entendendo? E daí, assim, a gente, depois que a gente conversava, a própria equipe fazia avaliação. A gente não pode sofrer. A gente faz o que, o que está ao nosso alcance, né, porque muitas vezes também, lá na equipe, eu era cobrada da equipe e dizia assim, "tá, [nome da entrevistada], nós vamos ter que ver agora porque vão ter que enviar a denúncia." Eu digo, "não, é tranquilo, pode deixar, né?". Não é pra dizer... Agora, vamos fazer o quê, vamos levar adiante. Vamos levar adiante, porque é uma criança de 9 anos, né? Uma criança de 9 anos, ou a menina de 11 pra 12, ali, apanhando. Apanhando e os pais fazendo várias coisas ali, e daí, assim, como é que tu vai querer que uma menina de 11 pra 12 anos vai pra escola, "ai, tô feliz, hoje eu quero participar, né, essas coisas." A gente falava isso entre nós. E daí, tu sabe que é uma coisa que o psiquiatra dizia pra mim sempre, né... Pra mim e pra psicóloga. "Gurias, nunca levem pro lado, não peguem problema pra vocês." A gente trabalha a situação, não fiquem com problema, não tenha problema, porque a gente vai fazer

o que a gente... O que é possível, né. Existe... Tá existindo, só no TS, o protocolo da gente fazer a denúncia, de, o protocolo da, como é que se chama? Na questão, não existe um protocolo, assim, quando o profissional detecta a depressão, de pegar e encaminhar?

<Arquivos\\ > - § 3 referências codificadas [0,55% Cobertura]

Referência 1 - 0,22% Cobertura

"Alguém já tentou fazer coisas sexuais com você a sua vontade, contra a sua vontade, te ameaçando, te machucando?", se tratando de adolescente, menor de idade, uma resposta sim... O que que nós vamos fazer com essa resposta? Vamos notificar, não vamos notificar?

Referência 2 - 0,18% Cobertura

Buscar mais. Eu ficaria em torno da notificação. Se adentre mais... no sentido vamos ter que prestar - os (?) diz assim, vamos ter que buscar tanto adolescente quando familiar, mas não digo tornar público, mas...

Referência 3 - 0,15% Cobertura

Pensando o marco regulatório, né, a normativa ali, ECA, né. Primazia do estado, toda aquela situação. Também. E que talvez aqui esteja desencadeando algum sofrimento, né.

<Arquivos\\> - § 2 referências codificadas [1,81% Cobertura]

Referência 1 - 0,52% Cobertura

Se esse paciente, esse adolescente de 14 e 17 anos é da UBS, daí eu acho maravilhosamente maravilhoso ir para o prontuário família. Botarem no sistema, porque o sistema aqui da UBS é único hospital, então onde ele quer que ele for consultar, quando forem ler ali o relatório dessa criança, já vem que ele já respondeu um questionário. O trabalho de vocês vai ser muito melhor para nós, que somos profissionais. Até mesmo para vocês, eu acho maravilhoso.

Referência 2 - 1,30% Cobertura

É, isso que tu falou agora, dentro de um cenário, um cenário do profissional que conheça onde ele está pisando. Porque uma coisa é eu pegar esse questionário e fazer o questionamento com algum paciente no qual eu domino. Outra coisa é o outro profissional, que não conhece o meu paciente, que não conhece nada, querer mostrar esse questionário para ele, porque esse questionário vai ser bom, dependendo da forma que ele for abordado pra pessoa, para poder preenchê-lo. Dependendo do profissional que for passar, e se não souber passar, se não souber abordar, é muito ruim. Aí é uma coisa que vira até contra nós, dependendo. Ah, querem que eu faça umas respostas... Podem levar para os pais, com outro olhar, ai mãe, olha só, me perguntaram isso. E isso pode estar acontecendo dentro da casa mesmo. Mas a criança já vê com outro olhar. Não vê como o olhar da proteção, ele vê com um olhar assim - ó, tão dizendo que tu está fazendo isso, tão até pedindo pra eu responder um questionário. Aí, quando tu vê, vem a mãe aqui, perguntando quem são vocês pra estarem fazendo isso. Então em vez de a gente... vai ficar O criminoso, em vez de ajudar.

HEALTH WORKERS (PE)

<Arquivos\\ > - § 3 referências codificadas [1,04% Cobertura]

Referência 1 - 0,23% Cobertura

É que a.... preocupação é tão pouca com relação a isso. e a demanda tá tão grande, que eles vão querer dizer mais... se eles encontrarem uma forma de dizer pra alguém, de que alguém vai ajudá-los, ouvi-los, poder relatar pra alguém, eles automaticamente escreveriam, mas aí já seria a segunda etapa, do atendimento, né.

Referência 2 - 0,12% Cobertura

Porque... se ele tem alto risco de desenvolver depressão, ele tá... numa situação de emergência, ele vai precisar de alguém. Eu quero saber se ele vai saber quem procurar.

Referência 3 - 0,69% Cobertura

O que acontece é o seguinte. Então eu tenho a minha... o meu diagnóstico de alto risco de depressão. Mas aí conta muito com... a sorte de que esse indivíduo, nessa situação vá procurar outro recurso, que não o recurso originário (do que respondeu)... seria melhor em buscar, falar "olha, já respondi, eu já coloquei agora... Eu tenho esse risco e tal". Aí o quando dá o CVV, ah, eu tenho um alto risco? Tu entendeu? Eu já respondi, eu já coloquei e por aqui você pode entrevistar, ele mostra ti daqui. Aí vai para o CVV, aí vai pro outro, pro outro, vai acabar indo pra lista de espera... Pode ser uma tiro pela culatra, porque se a pessoa está em alto risco de depressão, já passou por tudo aquilo ali, já viu briga, já foi machucada... e... se eu responder, deu isso, eu vou lá, né... vou no CVV, ah, eu respondi uma questão aqui, deu alto risco.... não sei como o CVV vai... Existem altas dificuldades em manter o CVV inclusive. Então é isso. É muito emergentes essa situação.

<Arquivos\\PE-11> - § 3 referências codificadas [1,59% Cobertura]

Referência 1 - 0,20% Cobertura

Pois é. Eu fico me perguntando como é que seria a questão de sigilo. Considerando situações que garantissem sigilo, qual seria a viabilidade em termos de praticidade numa escola.

Referência 2 - 0,95% Cobertura

Ah, eu acho que são muitos. Acho que se tem... tem alguma condição de encaminhamento, e existe.... esse caso que eu te narrei, narrei por isso. A [nome da universidade] tem condições bem precárias de atender. Mas elas existem. E aí porque isso não é detectado, muitas vezes os usuários não têm acesso. Então esse menino teve sua situação resolvida ou encaminhada. Não sei se resolvida porque eu não fiquei lá, mas ele teve a situação dele encaminhada graças a uma detecção. Então se a gente tivesse uma coisa sistemática seria super legal. A [nome da universidade] tem mais condições... tem pouca gente de 17 anos, como é risco futuro, né, mas estou te dando assim como um exemplo. Eu acho que qualquer recurso que exista, é inadmissível que não esteja sendo usado quando a gente está lidando com uma coisa tão séria. E eu acho que é o caso. Então acho que só por isso já valeria.

Referência 3 - 0,44% Cobertura

Nenhuma, eu acho que se a gente está lidando com um adolescente que de fato tem risco de depressão, ele já está estigmatizado, porque os sintomas já vão ser suficientes para essa nossa vida social terrível produzir os estigmas. Então eu não acho que ia assim cutucar as pessoas e torná-las hostis. Se elas tão pra ser hostis, elas já se tornaram porque isso apareceu de uma maneira ou outra.

<Arquivos\\ > - § 1 referência codificada [0,33% Cobertura]

Referência 1 - 0,33% Cobertura

Sozinha, contigo, eles diriam. O problema é o grupo, os colegas saberem. Se um professor souber, se o orientador profissional souber, isso não é problema, pra maioria, acredito. O problema é os outros saberem, por que para o adolescente é muitíssimo importante o que os colegas acham

<Arquivos\\> - § 1 referência codificada [0,24% Cobertura]

Referência 1 - 0,24% Cobertura

Entrevistado: Foi só uma pergunta se o adolescente utilizou ou não bebida alcoólica, maconha, então, não foi um incentivo ao uso. Acho que a escola explicaria isso.

<Arquivos\\ > - § 5 referências codificadas [1,07% Cobertura]

Referência 1 - 0,14% Cobertura

De desenvolver porque se apresenta alguns sintomas hoje e a criança não está depressiva e ela apresenta uma coisa que tenha... Que ela falou para nós, falou para ti, falou para alguém, e tu acha "essa aí tem uma predisposição. Então, a gente vai agir agora." Mas sem saber se ela vai desenvolver ou não.

Referência 2 - 0,40% Cobertura

Porque... Sabe que, ouvindo tu falar isso, teve uma palestra aqui na escola que falaram de abuso na infância. Aí, começaram a falar determinados abusos, que isso é abuso, isso é assim, assim, assado. Aí, uma menina levantou e saiu chorando. Aí eu disse "bah, meu Deus do céu, que que eu fiz..." Aí, "sora, eu descobri que eu fui abusada, eu não sabia." Poxa. Será que essa palestra acordou o que ela nem sabia? Ela nem sabia que ela tinha, levava uma vida normal, uma vida dita normal, assim, levando... E, daqui a pouco, ela, com aquela palestra, ela vindo lá da infância do tio, aquela coisa toda, "eu fui abusada". Aí, ela já fica triste, já não fala com os pais, já fica com raiva do tio, já não fala com ninguém, já se sente suja, já se sente injustiçada. Pô, será que tu despertou aquilo ali, tu fez bem a palestra ou tu não fez bem a palestra?

Referência 3 - 0,07% Cobertura

E agora? O que eu fiz para esse adolescente? Será que era melhor ela ter ficado ali, não sei se é anonimato que diz, na dormência, assim, ficar...

Referência 4 - 0,36% Cobertura

É? E o que que eu faço num momento desses? A guria estava tremendo, chorando e, com o meu jeito simplório - agora não vou dizer mais tosco, vou dizer simplório -, "ai, chama a palestrante aqui, então, chama, chama, vem me ajudar." E exatamente numa quarta que eu estava sozinha. Mas ela se acalmou depois. E até pensando, depois eu disse para ela, "olha, Fulana, quem sabe daqui para diante, tu já tem consciência disso, tu não vai deixar nenhuma amiga, nenhuma prima, mais ninguém ser abusada, tu vai orientar, tu vai ajudar, tu vai fazer um bem para todo mundo... Para a tua vizinha, tu vai ver, e tu vai ver que é abuso, e agora tu já sabe o que que é abuso." "Ai, eu fui abusada.", não sei o que, não sei o que, ai, meu Deus do céu, e agora? Mas, enfim...

Referência 5 - 0,09% Cobertura

Tudo isso eu pensei. Não vai prejudicar, não vai prejudicar, vai, não vai. Então, a informação é a melhor coisa. A informação acredito que vêm para o bem, a informação. Como lidar com isso, né.

<Arquivos\\ > - § 1 referência codificada [0,34% Cobertura]

Referência 1 - 0,34% Cobertura

Não acredito. Talvez nessa questão sexual, talvez ali esse adolescente ao responder isso, se sofreu abuso, pensar ainda o que poderia trazer de problema para com família. Talvez isso. Mas não acredito que isso seja

empecilho totalmente pra todos, pra usar. De repente se tivesse nessa questão aí um "prefiro não responder", sei lá, sabe como são adolescentes.

<Arquivos\\ > - § 1 referência codificada [0,88% Cobertura]

Referência 1 - 0,88% Cobertura

Entrevistado: Eu acho que vai depender de como essa entrevista vai chegar pra ele. Eu penso que se ela, se antes tiver uma informação, aí que eu já não sei. Porque, claro, a gente sabe que tem uma diferença entre tu responder uma entrevista sem... ter nada ainda, né, nenhuma pré informação, e quando tem. Mas, talvez, se ele entender porque que isso tá sendo feito e ele conseguir ter essa compreensão, lá no final eu acho que é isso que ele deseja mesmo, "Ah, eu quero saber". Né, porque pode estar se questionando, quem sabe, só pensando sobre isso, aí li vai dizer que ele tem um alto risco, tá, então talvez eu tenha que me cuidar, tenha que ser um alerta. Ou às vezes até para aqueles casos daqueles alunos que usam muito... essas características pra, pra... que talvez nem seja depressão mas que ele gosta de usar pra chamar, tá usando isso pra chamar atenção em casa. Claro, já o chamar atenção já faz parte, mas o adolescente precisa chamar atenção, certo, seja pelo bem ou pelo mal, né. Mal, assim, relativo. Bem também relativo. Mas ele precisa chamar atenção de alguma maneira. Talvez ele veja, "Ah, mas é baixo risco", aí não sei também, ele pode pensar, "Ah, baixo risco, então eu não tô fazendo a coisa certa. Então eu vou piorar esse negócio aqui", né. Agora que tu falou... pois é... eu acho que ficou muito pronto isso, sabe, muito pronto, assim.

HEALTH WORKERS (PS)

<Arquivos\\ > - § 2 referências codificadas [1,20% Cobertura]

Referência 1 - 0,33% Cobertura

Verdadeira que eu quero dizer não é que está 100% correta, né, mas que a informação, ela tem que ser transmitida, assim. Se a pessoa se dispôs a preencher, né, sabendo que no final vai ter essa possibilidade de ter um escore ou outro, um risco ou outro...

Referência 2 - 0,87% Cobertura

É. Eu acho que a informação tem que ser fornecida. E que, depois, vai se pensar, né, cada um vai fazer o que quer com aquela resposta ali. Mas eu acho que, se a gente tem a informação, ela tem que ser oferecida. Eu não me preocupo, me preocupo... Não me preocupo com o que vai acontecer depois, porque daí também é... Às vezes eu acho que a gente se implica muito no resultado. Tipo, "ah, se eu der esse resultado para um adolescente, o que acontecer com ele depois é minha responsabilidade." Acho que até um certo ponto. Se a gente tem o esclarecimento, "ó, você vai responder isso, no final vai ter isso e isso." e a pessoa foi mesmo assim, também acho que tem a participação dela...

<Arquivos\\ > - § 2 referências codificadas [1,60% Cobertura]

Referência 1 - 1,33% Cobertura

Entrevistado: E muito presente, e ela pode, né, de alguma forma, trazer uma ideia de que, ah, mas, então, como eu não tenho pai e mãe e eu tenho só... enfim, uma outra referência, que pode ser a tia ou a vó, ou eu tenho dois pais que me adotaram, dois homens que me adotou, uma série de coisas e eu já tô fora.
Moderadora: Algo tá mal.
Entrevistado: Algo tá mal e o próprio instrumento aqui poderia indicar já um problema meu, né. Então, entre seus pais aqui fica, bom, pais considerando aqui as diferentes configurações familiares para que a gente não induza, assim, né, olha, você, então, já tem um problema. Porque... "Separado dos seus pais"... "Já aconteceu de ter brigas..." Aqui imaginando, eu não sei se é pensado para crianças em geral nessa faixa etária, aqui a gente tem, né, experiência em geral que muitas crianças que estão nas escolas, nos projetos, elas estão em acolhimento institucional, estão em abrigos, né. Então, quando elas vão, por exemplo, vão responder, e a

gente tem que pensar em todo mundo, né, quando elas vão responder, então, parece que já não é bem pra elas. Porque "ter brigas na sua casa" pode "ter brigas no abrigo", né, que é uma casa. Uma casa lar, enfim. Então o quanto elas vão se sentir contempladas, "ah, eu faço parte desse grupo de 14 e 17, sou uma adolescente como a minha colega, só que a minha casa é diferente, não é uma casa com pai, mãe." Não sei se eu...

Referência 2 - 0,28% Cobertura

Então, aqui de um adulto ou adultos, né. Então, essa seria a 8, a 9, né. "Já aconteceu de um adulto que estava cuidando de você..." Aqui de novo, "de um adulto da sua família ou alguém que estava cuidando de você"... Pode ser familiar, né, enfim. Acho que vai na mesma...

<Arquivos\\ > - § 1 referência codificada [1,14% Cobertura]

Referência 1 - 1,14% Cobertura

Pois é, dentro desse treinamento teria que trabalhar com estigma da depressão. Eu sei que tem um trabalho no interior de São Paulo que todas as crianças estavam passando por rastreio, desde muito pequenas, para trabalhar prevenção. Eu não sei qual o impacto disso, porque era uma equipe de psiquiatras e psicólogos de saúde mental que iam nas escolas do munícipio, então todo mundo era rastreado. Eu não sei como está a situação, porque era até que foi de relato de experiência, assim, mas eu não sei como é que - se esse retorno que é dado.. Porque é aquela situação, primeiro a gente vai fazer uma notificação de alguma violência, a gente não vai fazer a investigação, a gente faz a notificação do que é, não precisa nem saber quem é que está causando esse dano à criança, ao adolescente, a gente notifica e vai ter alguém especializado para fazer. Então acho que a escola vai ter um limite, ela não precisa saber se tem diagnóstico, ela precisa saber identificar, e aí encaminhar, acho que ela tem limite para que aí algum profissional especializado nisso, aí entra nossa importância/expertise, vai poder identificar. Aí retorna para a escola sendo limitada, dizendo o que a escola precisa saber para educação.

<Arquivos\\ > - § 2 referências codificadas [0,53% Cobertura]

Referência 1 - 0,32% Cobertura

Em um primeiro momento eu ia sugerir as escolas, porque acho que é onde eles estão o tempo todo. Tem que ter um cuidado para não supervalorizar os resultados dela, teria que ter o ponto de vista crítico. Deixar bem claro que aquilo seria uma triagem, que não é nada que defina o diagnóstico sozinho, que é só uma suspeita. Já tem aplicativos pra celular que fazem esse tipo de coisa. Os adolescentes costumam gostar também.

Referência 2 - 0,21% Cobertura

Pois é, eu tenho um pouco de medo disso. Acho que tem que ser feita uma capacitação, uma conscientização muito boa. Uma “empatização” - vamos dizer assim, sobre a depressão em si e os adolescentes que estão de alguma maneira em risco ou sofrendo pra não gerar esse efeito contrário.

<Arquivos\\> - § 4 referência codificada [2,2% Cobertura]

Referência 1 - 0,38% Cobertura

Depende da divulgação do resultado, né, assim, ah... Por isso que escola é complicado, na escola não existe muita privacidade. As pessoas falam as coisas de uma forma muito inconsequente. Esses dias, eu estava trabalhando com uma escola particular, uma menina vítima de abuso. Todo mundo na escola ficou sabendo, então, assim... Ah, ela preencheu o questionário e deu alto risco. Então, esse tipo de coisa, eu acho que então, né... Acho importante não ser anônimo pela consequência e pelo risco, mas com algum critério muito grande, assim, de privacidade disso. Outro risco, não sei, acho que não, assim, não me ocorre.

Referência 2 – 0,46% Cobertura

Mas, assim, as perguntas são pertinentes, acho que é legal e interessante. Acho que teria que ter um pouco mais de consequência para o alto risco. Acho que só o CVV... Por quê? Porque tanto o CVV quanto o portal saúde, eles exigiriam uma iniciativa, e eu acho que não é suficiente. Uma menina de 14 anos que preencher, que dê alto risco, ela não vai procurar o CVV, ela não vai procurar nada, então, talvez algum tipo de alerta, né, combinado previamente. Então, tá, se der alto risco... Ou não, alguma coisa mais genérica do tipo, bom, aparecendo alto risco, isso será... Você será... Você terá algum tipo de acesso ajuda, e aí, a partir daí tem algum tipo de alerta para a unidade básica, alguma coisa, porque sozinhos não vão procurar.

Referência 3 – 0,64% Cobertura

Claro, a gente não pode tutelar tudo, mas como é adolescente, não adulto, deveria, né, até os 17 anos pelo menos deveria ter algum tipo de alerta para alguém. Se é numa escola, que um alerta ao diretor para que possa minimamente, enfim. Porque da iniciativa do adolescente com risco de... Claro, é um risco para o futuro que ele aponta, mas na verdade respondendo assim às perguntas de risco aponta praticamente uma depressão no momento, né. Eu não sei também, talvez seja importante, esse futuro o quão vago ele é, porque se um adolescente de 14 preencher que já tomou bebida alcoólica, que já, que já, que já foi criado por outra pessoa por um tempo... Algumas perguntas ali já apontariam para hoje ou para amanhã, entende? O futuro fica vago nesse sentido porque pode ser daqui a três anos, mas pode ser daqui a uma semana ou presente porque, dependendo de como elas forem preenchidas, já é uma depressão. Já tem fatores suficientes para uma depressão. Então, né, nesse sentido que futuro seria esse também, acho que teria que pensar.

Referência 4 – 0,72% Cobertura

Unidade básica. Acho que como ajuda... Assim, hoje em dia o portal SUS, né, o sistema de Telessaúde, ele já tem, por exemplo, os enfermeiros já têm acesso aos SNAP, que é aquela escala já autorizada para avaliar déficit de atenção e hiperatividade e oposição, que se entrega, o próprio enfermeiro ou o pediatra imprime, entrega pra família e a família entrega um na escola, um para a mãe, um para a avó e as pessoas preenchem a escala e aquela escala é necessária para o encaminhamento para um neuropediatra. Então, assim, isso é focado, o lugar disso no sistema de saúde seria a unidade básica. Daí, talvez, com essa adaptação desse futuro mais próximo, assim, né, e daí não poderia ser anônimo, né, nesse sentido. A não ser que tenha só uma orientação ali, se for para um uso do sistema de saúde, precisa ser, não, essa... Essa menina está, eu estou aqui com a Fulana, ela vai preencher, deu risco pra depressão, eu vou encaminhar ao pediatra para uma avaliação melhor ou eu vou fazer uma entrevista de saúde mental e vou ver se já mando ou não para atendimento. Aí, não pode ser para fins de pesquisa anônima, né, tem que ser já para educação e prevenção.

<Arquivos\\ > - § 1 referência codificada [0,91% Cobertura]

Referência 1 – 0,91% Cobertura

Se os professores forem capacitados, eles podem estar mediando. Eu acho que sim, acho que os professores das escolas podem estar mediando isso. Claro, grupos de pesquisa podem estar fazendo, mas os professores sendo capacitados eu acredito que sim, sempre de modo muito responsável, não fazer aleatoriamente, toda hora estar aplicando, sabe, que no fim se torna também algo que não é digamos positivo e os adolescentes eles respondem aquilo que tu quer, também, a gente precisa ter um pouquinho de cuidado nesse sentido. Por isso, eu penso nos professores porque eles conhecem os adolescentes que têm, na sua grande maioria. Então, quando eles veem essas respostas, eles vão saber se aquilo está sendo real mesmo, eles conhecem o contexto desses adolescentes, ou se não, se ele tá respondendo àquilo que eu quero que ele responda ou que... Eles sabem o que é correto. Então, esse é um cuidado que se tem também.

<Arquivos\\ > - § 1 referência codificada [0,32% Cobertura]

Referência 1 – 0,32% Cobertura

Eu acho, assim, ó. Adolescente, tu vai entrar em escola, tem que pedir o termo de consentimento dos pais. Então, isso já é um problema. Se tu divulga na rede, aí, assim, tipo assim, não entra na escola mas divulga na rede... Olha, eu sei, assim, que o Facebook eles não usam mais.

<Arquivos\\PS-9> - § 1 referência codificada [1,61% Cobertura]

Referência 1 - 1,61% Cobertura

Entrevistado: As questões éticas eu acho sempre uma preocupação muito grande. É muito difícil para o ser humano em geral trabalhar com probabilidade, a gente não é bom probabilista em geral, e a gente... Quando a gente fala que alguém tem alto risco a gente tem que sempre lembrar que, primeiro, a gente pode estar muito bem errado, segundo, a gente te que cuidar com questões de profecias autorrealizáveis, né? Ah, então tu tem um alto risco para ficar deprimido? Bom aí mesmo que fica. Então a gente tem que avaliar se isso não acontece, e qual é o efeito - porque talvez a própria, a gente aplica o escore, a gente faz uma classificação, mas será que isso não tem um efeito deletério? Então até mesmo talvez, assim como a medicação tem efeito colateral, classificar alguém como alto e baixo risco possa ter um efeito colateral. Então essas coisas são muito difíceis de avaliar, e a gente vai precisar de estudos clínicos. Na prática, não adianta, por enquanto a gente está em âmbitos ainda mais, de novo, nos primeiros pilares dessa avenida que a gente está construindo nesse sentido de prevenção. É uma avenida que é muito válida, ela tem um bom... Acho que ela é muito bem intencionada, mas a gente não pode esquecer das questões éticas que estão implicadas em qualquer intervenção de saúde.

1. Code Query Output

<Files\\KII\\ > - § 12 references coded [7.06% Coverage]

Reference 1 - 0.54% Coverage

P: Uh…Yeah, if they come to know about something like this, a "high risk" or "low risk" thing, that can make them conscious and as written, they can consult to a counsellor or someone.

Reference 2 - 1.08% Coverage

P: The best way to find—I don't think that anyone will actually want to search something like this, so…I don't know what's the best thing. Hm, yeah…It has to be shown up automatically. People are not going to go search for something like this, like *"Oh my God, I have a high risk of depression"* or something. So…it has to be shown up automatically in front of them.

Reference 3 - 0.52% Coverage

I: What do you think about being shown the "high risk" and the "low risk"? If you were taking it and one of these popped up, what would you think about that?

P: Shock? (Laughs)

Reference 4 - 0.35% Coverage

I: Shock! Oh, ok (laughs).

P: Um…Obviously I would be keen to learn more about it. I just would not have stopped there.

Reference 5 - 0.87% Coverage

P: Hmm...maybe I'd want to know about some consultants. And the thing is, who is available to help you? There has to be someone that you can ask more about this. Different people have different confusions about everything, so maybe there has to be someone—a different contact—to ask more about it.

Reference 6 - 0.70% Coverage

P: It's just that the phase you are going through, it's not the only phase of your life. I mean like…you are not the only one to suffer from this. So, this is something that benefits you, I'd say, because you learn about yourself (laughs).

Reference 7 - 0.46% Coverage

P: If someone is actually taking it seriously, like *"Yes, I have to learn about it; Yes, I am one of the persons who need this."* So, yes, it is beneficial.

Reference 8 - 0.54% Coverage

P: Actually, it can give a little tension at that point of time. And…I would search more. I would say it to someone and surely go to a consultant if it showed "high risk" of depression.

Reference 9 - 0.38% Coverage

I: So, you said you would say it to somebody, you'd tell somebody? Who would you tell?

P: I would tell my elder brother about it.

Reference 10 - 0.47% Coverage

P: First there can be tension, as I already said. And, if they don't have somebody to tell—even if that is the case—I guess they will consult someone about this.

Reference 11 - 0.50% Coverage

I: Ok. So, when you say there's "tension" about it, why do you think there's tension?

P: A "high risk" of depression. Depression is a word—it is a tension! (Laughs) So…

Reference 12 - 0.63% Coverage

P: It has to be available freely, but also there has to be someone. Because people would not like to search for something like this by themselves. So, yes, I guess that even people going to them would be beneficial.

<Files\\KII\\ > - § 4 references coded [1.25% Coverage]

Reference 1 - 0.06% Coverage

P: It is very good.

Reference 2 - 0.29% Coverage

P: It is helpful for people with depression. It is helpful for all. It is very good.

Reference 3 - 0.62% Coverage

P: We hear about the people committing suicide due to depression. If they see this, go through the link, take counseling, they might come out of the depression. So, I find helpful.

Reference 4 - 0.28% Coverage

I: So that because of the risk, she does not get into depression later.

P: Yes.

<Files\\KII\\ > - § 8 references coded [6.42% Coverage]

Reference 1 - 0.09% Coverage

P: Yes, it will be good.

Reference 2 - 0.56% Coverage

P: I would be careful considering I have risk of depression. I will try to self-motivate. I try not to take tension. I will try to build positive attitude.

Reference 3 - 0.57% Coverage

P: Sharing with other. If suggestions are not received from other, we can search information on internet. No matter what, we need to keep ourselves positive.

Reference 4 - 0.46% Coverage

P: The negative aspect is that they get scared of the risks. They will get more depressed thinking they have risk of depression.

Reference 5 - 0.46% Coverage

P: I would be shocked. However I would also feel the need of doing something. I would have both negative and positive feelings.

Reference 6 - 0.69% Coverage

P: Parents would start loving their children more. They will analyze their mistakes. Some might get angry as well. Some might cry, take tension. They might think about what they missed out.

Reference 7 - 2.15% Coverage

P: Self hobby should be fulfilled. We should openly talk to others. Firstly, we should love and care own- self. Not being *Nakkali* (fake) more than required, but trying to look beautiful. Seeing own positive side, think to improve the negative side. If somebody is having problems as yours, then not sharing own problems with him/her but while providing solutions to him/her, we might get self-motivated. Motivating others and share things, talking openly. Though speaking alone is

considered to be crazy but when alone in home, if we imagine someone and share our feelings, we will feel light.

Reference 8 - 1.44% Coverage

I: In order to normalize, the information such as depression is simple, not a big deal and moreover, it does not mean that you are in depression but chances are high are if include will be good. Being an adolescent, are there anything in this tool you feel like filling?

P: Yes, I feel. We are going through something but we could not figure out. We become cautious that this is happening to us.

<Files\\KII\\ > - § 1 reference coded [1.60% Coverage]

Reference 1 - 1.60% Coverage

I: What do you think about it? How much easier it would be?

P: Yes. It'll become easier.

I: What do you think are the reasons that made it easier?

P: You'll get to know are you in it or not. Get help on what should we have to do. Get more help on preventing it.

I: How will it help for prevention?

P: Like, what can we do in this stage, Whom to share, how can we be protected

<Files\\KII\\ > - § 5 references coded [3.54% Coverage]

Reference 1 - 0.67% Coverage

P: I think that the tool will be widely used. When the result will come, if the person is at low risk, then that will not make any difference. But if high risk is shown in the people, then they might get scared.

Reference 2 - 0.52% Coverage

I: Yes. And if any adolescent is told that s/he is at high risk, s/he will have the scared or worrying feeling that they are at risk?

P: Yes, they will be in fear.

Reference 3 – 0.19% Coverage

I: At least, some…

P: At least, changes will appear in some.

Reference 4 – 1.04% Coverage

P: It can be used as link (app). It can be carried out as a research in different schools. Talking about the psychological (aspects), people are interested a lot to know about themselves. When psychological aspects are spoken about, people… If it is said that this is confidential, then I think that everybody would fill it up.

Reference 5 – 1.13% Coverage

P: I had a fear. So I did not go. If this type of tool was available, after I am aware I would use it and if I am known that I am at risk, then I would come. When there is no tool, then I think I will continue to remain in a confused state. If people see the percentage in even in small love match-making, then they will certainly see in this sort of thing.

<Files\\KII\\ > - § 2 references coded [2.11% Coverage]

Reference 1 – 0.69% Coverage

P: I felt very happy. [Laughing] I was about to say that it would be very useful. If it would be available, anyone can fill it up. After confirming whether we have it (depression) or not, we can go to the doctors.

Reference 2 – 1.43% Coverage

P: When told that they (patients) at risk, one who has positive vibes, take it positively. And one with negative vibes starts overthinking. He might be more worried. When I was here to talk to sir, for 2/ 3 days, I had become a person who was overthinking. After that I started relaxing, started to understand. After paying attention, I realized that I should not overthink. Then, I started thinking (about it) less. That one thing might happen.

Code Query Output:

<Files\\KII\\ > - § 1 reference coded [2.27% Coverage]

Reference 1 - 2.27% Coverage

I: Ok…

P: Whom are you targeting?

I: We are planning to target adolescents and we have just made this tool as an example of what it is going to look like. And at last what we will do is, calculate the risk of the person and determine whether they are at high risk or low risk of depression. And as you can see (Showing the risk calculator prototype), we have some information popping for those who are at high as well as low risk for depression. (Interviewer reading the information section to participant) Do you think this information is enough? Or do you we have to add something more?

P: Doesn't it seems like an excuse for the person? May be I will be backing out from my job or stop studying and blame my condition and say that it is because I am at risk of depression?

I: Is it?

P: Yes!

I: Yes, it is a challenge! What could we do to minimize it?

P: We have to counsel them from a different approach and tell them that it is not a problem and instead approach their parents and tell them that their children are at high risk of depression.

<Files\\KII\\ > - § 1 reference coded [1.49% Coverage]

Reference 1 - 1.49% Coverage

P: It will be good. If share with him/her, s/he will take tension. If shared with guardians, they will try making him/her feel good. S/he will feel that people care about them. A kind of positive attitude is developed. If shared with others, s/he won't know that s/he is having depression and would not take tension. Others show love knowing this and try to care. Due to this, the negative feelings clear out.

<Files\\KII\\ > - § 1 reference coded [0.59% Coverage]

Reference 1 - 0.59% Coverage

P: In apps as well, there are many pranks related apps. So people will trust if kept in original/authorized websites and that would be easy as well.

<Files\\KII\\ > - § 4 references coded [3.31% Coverage]

Reference 1 - 1.19% Coverage

P: If the confidentiality will not be there then… That is like ignoring their feelings and sharing with others. It is like I gave my (interview) here but the information was given/ leaked somewhere else. So that might not work. They (adolescents) will use more, if the information is kept confidential. Even if 90% does not, 10% might seek help if confidentiality is maintained.

Reference 2 - 0.26% Coverage

I: Confidentiality should be maintained to optimum?

P: Yes, should be maintained.

Reference 3 - 1.04% Coverage

P: It can be used as link (app). It can be carried out as a research in different schools. Talking about the psychological (aspects), people are interested a lot to know about themselves. When psychological aspects are spoken about, people… If it is said that this is confidential, then I think that everybody would fill it up.

Reference 4 - 0.82% Coverage

P: Oh, nothing like that. People trust the internet maximum these days. They have been using it normally as well. So the challenges will not be there but one (issue) can be of confidentiality. When known this is confidential, I think they will not (hesitate).

<Files\\KII\\ > - § 2 references coded [1.04% Coverage]

Reference 1 - 0.76% Coverage

I: Online would be accessible too, right? How much does the confidentiality matter (when using) these tools? The matter of privacy and confidentiality…?

P: I think that might not be possible because if we put our name, email and all.

Reference 2 - 0.29% Coverage

P: We should make them fill-in their name. Some process shall be applied, some security...

<Files\\KII\\ > - § 1 reference coded [1.07% Coverage]

Reference 1 - 1.07% Coverage

I: If you are to fill up this tool, what would you feel like? Do you think this tool is way too long to be filled up and provide self-information? Or do you think that it is good?

P: I think that the risk factors you have mentioned here are not even enough. And about the length of this tool, people won't stress out looking at this. Adolescents are concerned about the things like this and they like to read and won't feel that it's long.

I: Ok. So, this kind of tool will be effective?

P: Yes!

<Files\\KII\\ > - § 2 references coded [0.90% Coverage]

Reference 1 - 0.21% Coverage

P: No, they are really easy, and…the questions are right, they're fine.

Reference 2 - 0.69% Coverage

I: Ok, so—if I'm understanding correctly—you're saying that, you might feel like you're not the only one, if you were to see this and you got those results? And maybe you feel like you know a little bit more information?

P: Yes, yeah.

<Files\\KII\\ > - § 6 references coded [3.20% Coverage]

Reference 1 - 0.18% Coverage

P: Questions are not difficult. It is understandable.

Reference 2 - 0.09% Coverage

P: All questions are good.

Reference 3 - 0.16% Coverage

P: Further addition is not needed. It is fine.

Reference 4 - 0.06% Coverage

P: Yes, enough.

Reference 5 - 2.54% Coverage

P: I think that it does not have questions about relations. I think it has about family only. I have heard about going into depression because of relationships. So, if there would be few questions about relationships, then I think it would be convenient.

I: We talked about high-risk. Now let's talk about low risk. If a person is in low risk, there are some instructions. Apart from this, should there be anything added. For instance, it has instructions like though in low risk, you need to take care of you mental health, good sleep in needed, healthy food, to know what you need, go to the link. It also says that you can consult with reliable counselor. Is there anything to add besides this?

P: No. Need to talk with others. It is fine.

Reference 6 - 0.17% Coverage

I: You think this is enough, don't you?

P: Yes.

<Files\\KII\\ > - § 1 reference coded [0.29% Coverage]

Reference 1 - 0.29% Coverage

P: Solutions should be there. This should also include something that motivates.

<Files\\KII\\ > - § 2 references coded [0.27% Coverage]

Reference 1 - 0.12% Coverage

P: This is fine the way it is.

Reference 2 - 0.15% Coverage

P: I don’t find anything to be added.

<Files\\KII\\ > - § 3 references coded [0.81% Coverage]

Reference 1 - 0.23% Coverage

I: Is there any question you don’t understand?

P: No!

Reference 2 - 0.29% Coverage

P: Providing the links which let you know about the way to calm down…

Reference 3 - 0.30% Coverage

P: Links about how to prevent depression and help to reduce the stress…

<Files\\KII\\ > - § 3 references coded [2.24% Coverage]

Reference 1 - 0.99% Coverage

P: That depends upon the choice of words. When in high risks, rather than just on normal things like, nutrition and help-seeking, content like information that it can be cured should be included. Otherwise, the fear will be there. We are scared to even look at the results when we take normal astrology… [Laughing]

Reference 2 - 0.62% Coverage

P: While giving that link, one for help, another link to understand about their condition… Rather than writing there, putting the link there and further they may visit different websites and then…

Reference 3 - 0.63% Coverage

P: No, it is fine. It is simple. We need not think much. It is simple. That one which requires to tick. The questions are so much simpler. That is a different thing but the setting is simple and good.

<Files\\KII\\ > - § 2 references coded [3.74% Coverage]

Reference 1 - 2.84% Coverage

I: How can we address that? We have said that we will provide those tools to the adolescents so that they can fill it up. What can be done to prevent the negative consequences? What can be the possible solutions to minimize that problem?

P: Nothing exactly has clicked on my mind. [Laughing] Other options... others might also be added.

I: Such as information? Are the information included under high and low risk enough or is additional information...

P: [Interrupting] I think that some more has to be added and questions shall also be added.

I: What kind of information should be added such that...? As you are of the same age-group, your point of view matters the most.

P: Differentiating in percentage. Having this percentage...

I: Instead of only determining high and low (risk), also differentiating the risk in percentage and providing information accordingly would be good?

Reference 2 - 0.89% Coverage

P: Rather than doing that, personal... because s/he might feel shy. If s/he is asked, "Have you made these mistakes?", then s/he will not say that s/he has done the mistake, s/he will say, "No". So, instead of that, s/he should be given to fill-in directly which would be better.

1. Code Query Output

<Files\\KII\\ > – § 2 references coded [1.78% Coverage]

Reference 1 – 0.75% Coverage

P: ahhh… in initial phase due to lack of understanding…things might not be understood easily…but in order to get it have started, it will be good. And at least School authorities or specific person if assigned with specific roles could be effective.

Reference 2 - 1.03% Coverage

I: Are there any associated risk while using the tool?

P: umm..it depends on what kind of information does it require. Or if user or school itself would also be marking various things or would be suspecting. So, in various situation behaviors are also marked. In such condition, these informations can be given but depends of type of markers.

<Files\\KII\\ > - § 6 references coded [11.38% Coverage]

Reference 1 - 1.31% Coverage

I: What will be the consequences of such tool? When an adolescent fill up this tool and know that they are at high risk of depression, will they follow the preventive measures or rather panic and stress thinking that they will be having depression in certain period of time?

P: I don't think that will happen. I don't think there is any such preventive measures because it is all about how you think and perceive. The person could deny and say that depression is something which victimize weak people and try to make themselves strong saying so. If it helps then it is good else it will have such consequences.

Reference 2 - 3.53% Coverage

I: Ok…

P: Whom are you targeting?

I: We are planning to target adolescents and we have just made this tool as an example of what it is going to look like. And at last what we will do is, calculate the risk of the person and determine whether they are at high risk or low risk of depression. And as you can see (Showing the risk calculator prototype), we have some information popping for those who are at high as well as low risk for depression. (Interviewer reading the information section to participant) Do you think this information is enough? Or do you we have to add something more?

P: Doesn't it seems like an excuse for the person? May be I will be backing out from my job or stop studying and blame my condition and say that it is because I am at risk of depression?

I: Is it?

P: Yes!

I: Yes, it is a challenge! What could we do to minimize it?

P: We have to counsel them from a different approach and tell them that it is not a problem and instead approach their parents and tell them that their children are at high risk of depression.

I: So, you mean, after calculating the risk of depression of that individual we have to reveal the result to their parents instead of revealing it in front of the individual themselves?

P: Yes.

I: So, we have to use that approach?

P: Yes!

I: And if we reveal the result to the parents of that individual, how will they perceive it? What would be the consequences?

P: Talking about myself, parents don't show their emotions. In front of us, they try to sow that they are happy. My parents were behaving like there is no any problem and that's why I didn't know about the problem much!

Reference 3 - 1.61% Coverage

I: If I tell your parents that you are at high risk of depression then what will happen to them? How would they feel? You said that your parent were very supportive and understanding that your parents brought you for counselling so that it would help you. But it might not be in others case. Do everyone react the same way or start ignoring their children thinking that they are at high risk of depression and they will anyway have depression after certain period of time?

P: The people who doesn't care about their children will say so. Those who know about it, they will obviously be supportive. Every parents care for their children but those who doesn't know about depression, they deny it and doesn't care…I forget what I was trying to say…

Reference 4 - 2.08% Coverage

I: Ok. So, those who don't know about depression may not care about their children when they know they are at risk of depression. If we tell them about depression, it could be cured from proper medications and we also could prevent it, then would it help?

P: Yes!

I: …

P: If a person knows about depression, then s/he somehow move on but if they doesn't know about it then there is no chance of them panicking and stressing about them being at risk of depression.

I: If you are to fill up this tool, what would you feel like? Do you think this tool is way too long to be filled up and provide self-information? Or do you think that it is good?

P: I think that the risk factors you have mentioned here are not even enough. And about the length of this tool, people won't stress out looking at this. Adolescents are concerned about the things like this and they like to read and won't feel that it's long.

I: Ok. So, this kind of tool will be effective?

P: Yes!

Reference 5 - 1.40% Coverage

I: For now, we have targeted adolescents for this tool. Do you think other people such as parents or teachers on behalf of adolescent can fill-up this tool and provide the information of that adolescent?

P: For that, we can do a trail on everyone and see how the information comes. The way adolescent perceive things, their parents might not. Parents might think that their children are brilliant and obedient as everyone at their home is like that but how actually is the adolescent in real, only the adolescent themselves will know. The answers r the information provided vary from different person's view. I think that, we have to look at both ways.

Reference 6 - 1.44% Coverage

I: That means, we could get some information even if the parents or teachers fill up this tool?

P: Yes!

I: We talked about the benefits of using this kind of tool but could there be some challenges while using this tool among adolescents? Could there be some problem or barriers?

P: No, I don't think there will be any challenges! While using this tool among adolescents, we are not saying the adolescent that we thought they are having problem so we are going to use this tool on them. We will instead tell them that we are using this tool on all other adolescents so they are one of them. No one will deny then.

I: If we do so, won't there be any problem?

P: Yes!

<Files\\KII\\ > - § 9 references coded [4.56% Coverage]

Reference 1 - 0.47% Coverage

P: It would be easy to use. Because uh, for a depressed, isolated person not expressing [themselves] to other people…yes, it is really good. *(incomprehensible*)

Reference 2 - 0.23% Coverage

P: Obviously uh…hm, where it has to be used… Maybe mostly for college students.

Reference 3 - 0.24% Coverage

P: Yeah. Or maybe someone who just dropped out and is now struggling afterwards.

Reference 4 - 0.42% Coverage

P: (Pauses) Um…maybe advertising it in social media, because everyone is using social media nowadays. So, in social media as well as campaigns.

Reference 5 - 0.44% Coverage

I: Ok, ok. What about in certain places—like do you think in schools, or offices? What about those places?

P: Hm, yes, even those can be good ones.

Reference 6 - 0.97% Coverage

I: Do you think this is something that's best used by somebody alone? Or is there somebody else with them when they use it?

P: It depends upon the cases, I guess. Some people—they just want to keep it to themselves. But, there are some people who are actually sharing with other people too. So, both of these are possibilities.

Reference 7 - 0.57% Coverage

P: Umm…I think a search. Because if I would have searched for something like this, I would go directly to Google. And then um…I would search for a consultant nearby. That's all I would have done.

Reference 8 - 0.63% Coverage

I: Yeah, that's true. Where would you think would be the most comfortable place for you to have that information shown to you? In what kind of setting?

P: (Laughs). I said it earlier, I would have directly Googled!

Reference 9 - 0.59% Coverage

I: What about in school or anything? Do you think that would be something that you'd be interested in, like if there was a school program?

P: Yeah, I would have been interested in something like that.

<Files\\KII\\ > - § 13 references coded [5.38% Coverage]

Reference 1 - 0.04% Coverage

P: In home.

Reference 2 - 0.11% Coverage

P: Yes it can be filled on own.

Reference 3 - 0.18% Coverage

I: Have you seen any challenges to fill this?

P: No.

Reference 4 - 0.05% Coverage

P: It is easy.

Reference 5 - 0.27% Coverage

I: You can fill it up yourself. You don't need teacher's help, do you?

P: No.

Reference 6 - 0.54% Coverage

P: People not having mobile phones can ask for it from others. People with depression should ask help from others. If others find out, then they can help them.

Reference 7 - 2.01% Coverage

I: As I mentioned previously as well, this is related to risks. It shows if their activities can be the reasons for depression in future. This is not for people with depression. However, they might be already in depression. By and large, it has been designed to figure out if the past the behavior can put them in the risk of depression in future or not. Everybody can fill it. But if people are uneducated, in addition they do not have mobile in home, if only one uses mobile, then how can it be filled?

P: If only one has, they she/ he should help. If they do not have, you should do.

Reference 8 - 0.13% Coverage

I: Doing it in school is easy?

P: Yes.

Reference 9 - 0.11% Coverage

P: I think it is easy in school.

Reference 10 - 0.26% Coverage

P: Teenagers spent most time in school. I think it is easy to fill in school.

Reference 11 - 0.21% Coverage

I: As teachers are also there.

P: Yes, better than in home.

Reference 12 – 1.09% Coverage

I: How important is it to provide training to teacher on this to make this compulsory in few schools? For instance, how to use it, what to do?

P: Conducting programs in schools. You need to explain if you have tension, go to this place. If we listen in school, we share in home as well. This way the message spreads.

Reference 13 – 0.38% Coverage

P: Mostly, in rural areas, there is a tendency of boy touching girls. So should be done in rural areas as well.

<Files\\KII\\ > – § 3 references coded [3.81% Coverage]

Reference 1 – 0.21% Coverage

P: Filling by self because I know what is happening to me.

Reference 2 – 1.55% Coverage

I: We know the best what we have experienced, don't we? However in several conditions, adolescents cannot fill by themselves. In this situation, how difference it would be if filled by parents or guardians? The information derived would not be right or that would be fine?

P: The information derived won't be that correct. They see one thing but there is something else inside us. Some could be matched and some might not be.

Reference 3 – 2.05% Coverage

P: These days, almost have the Wi-Fi but some might not have. So making available in apps is also good. Or, the forms can be provided in schools as well.

I: You said in schools and apps. Is it good to fill on own or should be accompanied by the near ones? Like I as a researcher have been asking you today, or someone close to you /near to you asking you and filling, what is good?

P: Both are good. The person might not be intending to open up and do not share everything. But even if accompanied by other while explaining it starts getting light from inside.

<Files\\KII\\ > - § 5 references coded [3.79% Coverage]

Reference 1 - 0.25% Coverage

P: This is more than going to the hospital, testing from home.

Reference 2 - 1.12% Coverage

P: Rather than going to hospitals, it can be known from home. It is helpful but some may take it as fake as well because it is online. It could be effective if available on websites but it can be fake. Rather than this, directly visiting the psychologists would be more effective.

Reference 3 - 0.80% Coverage

P: This is easy because we can do it personally. On one hand this is very nice and on other hand due to fake websites, there is the probability of this being faked. However, this is better than others.

Reference 4 - 0.75% Coverage

P: This would be very helpful because we can do it on our own and we do not need. to share with others. We will get to know what is happening to own-self. In that sense, this is very good.

Reference 5 - 0.87% Coverage

P: That would be more good if done in school because do not know about what is happening to students. If they get to know what has happened to students, students will happy as they won't dominate and make them feel bad.

<Files\\KII\\ > - § 7 references coded [4.97% Coverage]

Reference 1 - 0.26% Coverage

I: How easy do you think, it is to fill this up?

P: It's easy

Reference 2 - 0.21% Coverage

P: I don't need anyone to help me to fill it out.

Reference 3 - 0.45% Coverage

I: Why do you think you don't need anyone's help?

P: Because it should be filled up by analyzing our self.

Reference 4 - 0.97% Coverage

I: If we have to use this question on adolescents like you, how easy do you think it would be?

P: Umm, it would be easy. Everybody can openly put their point of view. One can easily mark the things that have happed to them or not.

Reference 5 - 1.61% Coverage

I: According to you, what challenges occur due to this questionnaire?

P: Someone could answer something that's not true or actual and then the result might not be valid. It might show someone is depressed but in actual they might not be!

I: So, how can we avoid that? What should be done to find the correct result?

P: Firstly, they should understand the situation and that's it.

Reference 6 - 0.42% Coverage

P: It will not work out if you do this in a group of people. It should be carried out individually.

Reference 7 - 1.06% Coverage

I: Don't you think it will take more time to do it that way?

P: Yeah, it takes time.

I: So, if we have to this for many groups of people, how do you think this should be done?

P: Different programs should be conducted and things should be made clear.

<Files\\KII\\ > - § 7 references coded [4.50% Coverage]

Reference 1 - 0.59% Coverage

P: Yes, of course. If I have not said anything at my own home, then we cannot assume that other people would also know everything. Thus, that should be given to them (adolescents) only.

Reference 2 - 0.56% Coverage

P: At first, parents should be asked how close their relationship is (with the patients). After knowing about their relationship... How well they have understood? Based on that…

Reference 3 - 1.04% Coverage

P: If they had only looked at my case… Both of my parents are working. I used to live with my grandmother since I was one month old. My mother used to go to work and I used to be with my grandmother. Because of that relationship with her children became weaker. In that situation, even if they fill it up, what (result) would come?

Reference 4 - 1.04% Coverage

P: It can be used as link (app). It can be carried out as a research in different schools. Talking about the psychological (aspects), people are interested a lot to know about themselves. When psychological aspects are spoken about, people… If it is said that this is confidential, then I think that everybody would fill it up.

Reference 5 - 0.39% Coverage

P: If people have even a little suspicion, the first they do is Google. So I think it will come in use, will be very helpful.

Reference 6 – 0.63% Coverage

P: No, it is fine. It is simple. We need not think much. It is simple. That one which requires to tick. The questions are so much simpler. That is a different thing but the setting is simple and good.

Reference 7 – 0.24% Coverage

I: If that happens, that might be useful in our setting, isn't it?

P: Yes.

<Files\\KII\\ > – § 7 references coded [6.83% Coverage]

Reference 1 – 0.26% Coverage

P: The advantage is for the specific person but could be disadvantageous as well.

Reference 2 – 1.17% Coverage

P: I do not think that there would be that much gap because they are their parents. Their mother and father have been observing him/ her very closely. They keep a close eye on them. Now, there are two (conditions). If the family has given him/ her much time, the exact (issue/ situation) can be known. But if the family has not given them time, then perhaps the...

Reference 3 – 2.94% Coverage

I: As you said, negative consequences can be there. One who has negative vibes can perceive badly. Now, the adolescent is given to fill-in all the information but not showing the last section to him/her... (Referring to the risk calculator) How it would be if the adolescent is at high risk, then telling his/her parents that the adolescent is at high risk but not telling the adolescent.

P: It depends upon the age. If s/he is of small age, telling parents is fine. Now talking about the parents too, it depends upon how their nature is. I have seen some parents who beat their children physically, if they do a mistake. They do not talk to them and make them realize that what they did was wrong. Instead of saying, "You should not do this", they beat them. In today's context as most of them are educated, there might not do so. Nevertheless, if both of them were told this to some extent, then it should be good.

Reference 4 – 0.76% Coverage

I: Do you think there any difficulties or barriers or challenges that can appear when using this tool?

P: That might not appear.

I: Using among adolescents...

P: Because there is no chance of this being misused, it might not come up.

Reference 5 – 0.44% Coverage

P: If it could be very convenient. Things to do on this topic... If it could describe what depression is to them, then they would check...

Reference 6 – 0.74% Coverage

I: We have planned to develop it online. Now, we have it as an online form only.

P: These days, social media are widely used such as Facebook, Twitter, putting it up there...

I: Putting it up there would also be good?

P: Yes.

Reference 7 – 0.53% Coverage

P: When doing it through paper, perhaps only certain area... Like some schools, colleges (could be covered) but if it is done online, whoever sees it would fill-in.

1. Code Query Output

<Files\\KII\\ > - § 2 references coded [1.78% Coverage]

Reference 1 - 0.75% Coverage

P: ahhh… in initial phase due to lack of understanding…things might not be understood easily…but in order to get it have started, it will be good. And at least School authorities or specific person if assigned with specific roles could be effective.

Reference 2 - 1.03% Coverage

I: Are there any associated risk while using the tool?

P: umm..it depends on what kind of information does it require. Or if user or school itself would also be marking various things or would be suspecting. So, in various situation behaviors are also marked. In such condition, these information can be given but depends of type of markers.

<Files\\KII\\ > - § 6 references coded [11.38% Coverage]

Reference 1 - 1.31% Coverage

I: What will be the consequences of such tool? When an adolescent fill up this tool and know that they are at high risk of depression, will they follow the preventive measures or rather panic and stress thinking that they will be having depression in certain period of time?

P: I don't think that will happen. I don't think there is any such preventive measures because it is all about how you think and perceive. The person could deny and say that depression is something which victimize weak people and try to make themselves strong saying so. If it helps then it is good else it will have such consequences.

Reference 2 - 3.53% Coverage

I: Ok…

P: Whom are you targeting?

I: We are planning to target adolescents and we have just made this tool as an example of what it is going to look like. And at last what we will do is, calculate the risk of the person and determine whether they are at high risk or low risk of depression. And as you can see (Showing the risk calculator prototype), we have some information popping for those who are at high as well as low risk for depression. (Interviewer reading the information section to participant) Do you think this information is enough? Or do you we have to add something more?

P: Doesn't it seems like an excuse for the person? May be I will be backing out from my job or stop studying and blame my condition and say that it is because I am at risk of depression?

I: Is it?

P: Yes!

I: Yes, it is a challenge! What could we do to minimize it?

P: We have to counsel them from a different approach and tell them that it is not a problem and instead approach their parents and tell them that their children are at high risk of depression.

I: So, you mean, after calculating the risk of depression of that individual we have to reveal the result to their parents instead of revealing it in front of the individual themselves?

P: Yes.

I: So, we have to use that approach?

P: Yes!

I: And if we reveal the result to the parents of that individual, how will they perceive it? What would be the consequences?

P: Talking about myself, parents don't show their emotions. In front of us, they try to sow that they are happy. My parents were behaving like there is no any problem and that's why I didn't know about the problem much!

Reference 3 - 1.61% Coverage

I: If I tell your parents that you are at high risk of depression then what will happen to them? How would they feel? You said that your parent were very supportive and understanding that your parents brought you for counselling so that it would help you. But it might not be in others case. Do every one react the same way or start ignoring their children thinking that they are at high risk of depression and they will anyway have depression after certain period of time?

P: The people who doesn't care about their children will say so. Those who know about it, they will obviously be supportive. Every parents care for their children but those who doesn't know about depression, they deny it and doesn't care…I forget what I was trying to say…

Reference 4 - 2.08% Coverage

I: Ok. So, those who don't know about depression may not care about their children when they know they are at risk of depression. If we tell them about depression, it could be cured from proper medications and we also could prevent it, then would it help?

P: Yes!

I: …

P: If a person knows about depression, then s/he somehow move on but if they doesn't know about it then there is no chance of them panicking and stressing about them being at risk of depression.

I: If you are to fill up this tool, what would you feel like? Do you think this tool is way too long to be filled up and provide self-information? Or do you think that it is good?

P: I think that the risk factors you have mentioned here are not even enough. And about the length of this tool, people won't stress out looking at this. Adolescents are concerned about the things like this and they like to read and won't feel that it's long.

I: Ok. So, this kind of tool will be effective?

P: Yes!

Reference 5 - 1.40% Coverage

I: For now, we have targeted adolescents for this tool. Do you think other people such as parents or teachers on behalf of adolescent can fill-up this tool and provide the information of that adolescent?

P: For that, we can do a trail on everyone and see how the information comes. The way adolescent perceive things, their parents might not. Parents might think that their children are brilliant and obedient as everyone at their home is like that but how actually is the adolescent in real, only the adolescent themselves will know. The answers r the information provided vary from different person's view. I think that, we have to look at both ways.

Reference 6 - 1.44% Coverage

I: That means, we could get some information even if the parents or teachers fill up this tool?

P: Yes!

I: We talked about the benefits of using this kind of tool but could there be some challenges while using this tool among adolescents? Could there be some problem or barriers?

P: No, I don't think there will be any challenges! While using this tool among adolescents, we are not saying the adolescent that we thought they are having problem so we are going to use this tool on them. We will instead tell them that we are using this tool on all other adolescents so they are one of them. No one will deny then.

I: If we do so, won't there be any problem?

P: Yes!

<Files\\KII\\ > - § 9 references coded [4.56% Coverage]

Reference 1 - 0.47% Coverage

P: It would be easy to use. Because uh, for a depressed, isolated person not expressing [themselves] to other people…yes, it is really good. *(incomprehensible*)

Reference 2 - 0.23% Coverage

P: Obviously uh…hm, where it has to be used… Maybe mostly for college students.

Reference 3 - 0.24% Coverage

P: Yeah. Or maybe someone who just dropped out and is now struggling afterwards.

Reference 4 - 0.42% Coverage

P: (Pauses) Um…maybe advertising it in social media, because everyone is using social media nowadays. So, in social media as well as campaigns.

Reference 5 - 0.44% Coverage

I: Ok, ok. What about in certain places—like do you think in schools, or offices? What about those places?

P: Hm, yes, even those can be good ones.

Reference 6 - 0.97% Coverage

I: Do you think this is something that's best used by somebody alone? Or is there somebody else with them when they use it?

P: It depends upon the cases, I guess. Some people—they just want to keep it to themselves. But, there are some people who are actually sharing with other people too. So, both of these are possibilities.

Reference 7 - 0.57% Coverage

P: Umm…I think a search. Because if I would have searched for something like this, I would go directly to Google. And then um…I would search for a consultant nearby. That's all I would have done.

Reference 8 - 0.63% Coverage

I: Yeah, that's true. Where would you think would be the most comfortable place for you to have that information shown to you? In what kind of setting?

P: (Laughs). I said it earlier, I would have directly Googled!

Reference 9 - 0.59% Coverage

I: What about in school or anything? Do you think that would be something that you'd be interested in, like if there was a school program?

P: Yeah, I would have been interested in something like that.

<Files\\KII\\ > - § 13 references coded [5.38% Coverage]

Reference 1 - 0.04% Coverage

P: In home.

Reference 2 - 0.11% Coverage

P: Yes it can be filled on own.

Reference 3 - 0.18% Coverage

I: Have you seen any challenges to fill this?

P: No.

Reference 4 - 0.05% Coverage

P: It is easy.

Reference 5 - 0.27% Coverage

I: You can fill it up yourself. You don't need teacher's help, do you?

P: No.

Reference 6 - 0.54% Coverage

P: People not having mobile phones can ask for it from others. People with depression should ask help from others. If others find out, then they can help them.

Reference 7 - 2.01% Coverage

I: As I mentioned previously as well, this is related to risks. It shows if their activities can be the reasons for depression in future. This is not for people with depression. However, they might be

already in depression. By and large, it has been designed to figure out if the past the behavior can put them in the risk of depression in future or not. Everybody can fill it. But if people are uneducated, in addition they do not have mobile in home, if only one uses mobile, then how can it be filled?

P: If only one has, they she/ he should help. If they do not have, you should do.

Reference 8 - 0.13% Coverage

I: Doing it in school is easy?

P: Yes.

Reference 9 - 0.11% Coverage

P: I think it is easy in school.

Reference 10 - 0.26% Coverage

P: Teenagers spent most time in school. I think it is easy to fill in school.

Reference 11 - 0.21% Coverage

I: As teachers are also there.

P: Yes, better than in home.

Reference 12 - 1.09% Coverage

I: How important is it to provide training to teacher on this to make this compulsory in few schools? For instance, how to use it, what to do?

P: Conducting programs in schools. You need to explain if you have tension, go to this place. If we listen in school, we share in home as well. This way the message spreads.

Reference 13 - 0.38% Coverage

P: Mostly, in rural areas, there is a tendency of boy touching girls. So should be done in rural areas as well.

<Files\\KII\\ > - § 3 references coded [3.81% Coverage]

Reference 1 - 0.21% Coverage

P: Filling by self because I know what is happening to me.

Reference 2 - 1.55% Coverage

I: We know the best what we have experienced, don't we? However in several conditions, adolescents cannot fill by themselves. In this situation, how difference it would be if filled by parents or guardians? The information derived would not be right or that would be fine?

P: The information derived won't be that correct. They see one thing but there is something else inside us. Some could be matched and some might not be.

Reference 3 - 2.05% Coverage

P: These days, almost have the Wi-Fi but some might not have. So making available in apps is also good. Or, the forms can be provided in schools as well.

I: You said in schools and apps. Is it good to fill on own or should be accompanied by the near ones? Like I as a researcher have been asking you today, or someone close to you /near to you asking you and filling, what is good?

P: Both are good. The person might not be intending to open up and do not share everything. But even if accompanied by other while explaining it starts getting light from inside.

<Files\\KII\\ > - § 5 references coded [3.79% Coverage]

Reference 1 - 0.25% Coverage

P: This is more than going to the hospital, testing from home.

Reference 2 - 1.12% Coverage

P: Rather than going to hospitals, it can be known from home. It is helpful but some may take it as fake as well because it is online. It could be effective if available on websites but it can be fake. Rather than this, directly visiting the psychologists would be more effective.

Reference 3 - 0.80% Coverage

P: This is easy because we can do it personally. On one hand this is very nice and on other hand due to fake websites, there is the probability of this being faked. However, this is better than others.

Reference 4 - 0.75% Coverage

P: This would be very helpful because we can do it on our own and we do not need. to share with others. We will get to know what is happening to own-self. In that sense, this is very good.

Reference 5 - 0.87% Coverage

P: That would be more good if done in school because do not know about what is happening to students. If they get to know what has happened to students, students will happy as they won't dominate and make them feel bad.

<Files\\KII\\ > - § 7 references coded [4.97% Coverage]

Reference 1 - 0.26% Coverage

I: How easy do you think, it is to fill this up?

P: It's easy

Reference 2 - 0.21% Coverage

P: I don't need anyone to help me to fill it out.

Reference 3 - 0.45% Coverage

I: Why do you think you don't need anyone's help?

P: Because it should be filled up by analyzing our self.

Reference 4 - 0.97% Coverage

I: If we have to use this question on adolescents like you, how easy do you think it would be?

P: Umm, it would be easy. Everybody can openly put their point of view. One can easily mark the things that have happed to them or not.

Reference 5 - 1.61% Coverage

I: According to you, what challenges occur due to this questionnaire?

P: Someone could answer something that's not true or actual and then the result might not be valid. It might show someone is depressed but in actual they might not be!

I: So, how can we avoid that? What should be done to find the correct result?

P: Firstly, they should understand the situation and that's it.

Reference 6 - 0.42% Coverage

P: It will not work out if you do this in a group of people. It should be carried out individually.

Reference 7 - 1.06% Coverage

I: Don't you think it will take more time to do it that way?

P: Yeah, it takes time.

I: So, if we have to this for many groups of people, how do you think this should be done?

P: Different programs should be conducted and things should be made clear.

<Files\\KII\\ > - § 7 references coded [4.50% Coverage]

Reference 1 - 0.59% Coverage

P: Yes, of course. If I have not said anything at my own home, then we cannot assume that other people would also know everything. Thus, that should be given to them (adolescents) only.

Reference 2 - 0.56% Coverage

P: At first, parents should be asked how close their relationship is (with the patients). After knowing about their relationship... How well they have understood? Based on that…

Reference 3 - 1.04% Coverage

P: If they had only looked at my case… Both of my parents are working. I used to live with my grandmother since I was one month old. My mother used to go to work and I used to be with my grandmother. Because of that relationship with her children became weaker. In that situation, even if they fill it up, what (result) would come?

Reference 4 - 1.04% Coverage

P: It can be used as link (app). It can be carried out as a research in different schools. Talking about the psychological (aspects), people are interested a lot to know about themselves. When psychological aspects are spoken about, people… If it is said that this is confidential, then I think that everybody would fill it up.

Reference 5 - 0.39% Coverage

P: If people have even a little suspicion, the first they do is Google. So I think it will come in use, will be very helpful.

Reference 6 - 0.63% Coverage

P: No, it is fine. It is simple. We need not think much. It is simple. That one which requires to tick. The questions are so much simpler. That is a different thing but the setting is simple and good.

Reference 7 - 0.24% Coverage

I: If that happens, that might be useful in our setting, isn't it?

P: Yes.

<Files\\KII\\ > - § 7 references coded [6.83% Coverage]

Reference 1 - 0.26% Coverage

P: The advantage is for the specific person but could be disadvantageous as well.

Reference 2 - 1.17% Coverage

P: I do not think that there would be that much gap because they are their parents. Their mother and father have been observing him/ her very closely. They keep a close eye on them. Now, there are two (conditions). If the family has given him/ her much time, the exact (issue/ situation) can be known. But if the family has not given them time, then perhaps the...

Reference 3 - 2.94% Coverage

I: As you said, negative consequences can be there. One who has negative vibes can perceive badly. Now, the adolescent is given to fill-in all the information but not showing the last section to him/her... (Referring to the risk calculator) How it would be if the adolescent is at high risk, then telling his/her parents that the adolescent is at high risk but not telling the adolescent.

P: It depends upon the age. If s/he is of small age, telling parents is fine. Now talking about the parents too, it depends upon how their nature is. I have seen some parents who beat their children physically, if they do a mistake. They do not talk to them and make them realize that what they did was wrong. Instead of saying, "You should not do this", they beat them. In today's context as most of them are educated, there might not do so. Nevertheless, if both of them were told this to some extent, then it should be good.

Reference 4 - 0.76% Coverage

I: Do you think there any difficulties or barriers or challenges that can appear when using this tool?

P: That might not appear.

I: Using among adolescents...

P: Because there is no chance of this being misused, it might not come up.

Reference 5 - 0.44% Coverage

P: If it could be very convenient. Things to do on this topic... If it could describe what depression is to them, then they would check...

Reference 6 - 0.74% Coverage

I: We have planned to develop it online. Now, we have it as an online form only.

P: These days, social media are widely used such as Facebook, Twitter, putting it up there...

I: Putting it up there would also be good?

P: Yes.

Reference 7 - 0.53% Coverage

P: When doing it through paper, perhaps only certain area... Like some schools, colleges (could be covered) but if it is done online, whoever sees it would fill-in.

1. **Code Query Output**

<Files\\FGD\\ > - § 7 references coded [4.31% Coverage]

Reference 1 - 0.47% Coverage

I: So, if we develop such a risk prediction tool/risk calculator, will the adolescent fill up the forms properly?

P5: Yes, definitely they will fill up the form but they won't go to the proper place for treatment. They fill up the as the form of experiment but they won't go for treatment follow up.

Reference 2 - 0.98% Coverage

P5: Yes and those who will look will have a curiosity of the risk they might have. And if they come to be in a high risk group, they starts panicking…stressing thinking that now there's nothing left, they are going to die! Those who come to be under the low risk group, they will think that it is fine for them and they can be happy about it. Those who have a bit love for their own life, they might choose a treatment path. Even if they choose the treatment method, they would search for the immediate result. But for this, we have to be on a continuous follow up and maintaining a disciplined life and living in a good environment…

Reference 3 - 0.11% Coverage

P3: I don't think filling this up will help the problem to get minimize.

Reference 4 - 0.63% Coverage

P5: So, I saw the negative parts of this. What we have to do is…we have to tell people that it will be good if we fill it up. As I have seen negative parts of it, I said it because it will help to trace the path and make it better. If we leave the application alone in the floor hoping it will work, then it won't. It is normal application but it's up to us how do we take it and boost up in the society.

Reference 5 - 0.14% Coverage

The answers come up there and people can take the children in need to the help centres.

Reference 6 - 1.00% Coverage

P5: Yes! Like I know I am going to have a problem because there is something in my mind which I am not being able to throw out of there. And that is going to make me in problem. And I even know I need help. And that help comes within the family. And if I don't get that help then that will be difficult for me. I know all this but I can't get help by myself, I need a supporting hands, a strong one and for a long time. If you have problem in a place, you can move out, but if you have problem in your mind, where would you go?

P3: In the night time, the problem are more! (Laughs)

P5: We can't go away from the problems we have within us.

Reference 7 - 0.99% Coverage

M: So, your (Indicating P3) daughter have depression. Do you think if you could have asked your daughter to fill this risk calculator form before she was having depression and it will help? Do you think if she would fill the form and knew she is at risk beforehand would be good?

P3: She also didn't know it was depression. She used to get aggressive and I used to scold her. And then she used to keep quiet. So, if I knew it before that it was sign that she would be depressed…

M: So, if you knew it could be depression…

P3: It would have been better. But it is not necessary that it is early identified in every cases of children.

<Files\\FGD\\ > - § 3 references coded [2.11% Coverage]

Reference 1 - 0.70% Coverage

P4: What I feel is…what I have understood is… this tool (Risk calculator) is not to be filled by anyone but is self-administered. If it is something that they should not show to towers, then they will fill up the questionnaire properly. Children at this point have access to internet and they have a wish to know about their health condition with the help of internet. They like to know how they are. They even watch in YouTube about how beautiful/handsome they are. They use many

remedies to be beautiful which they learn from internet. So, if this tool is to be used by them (Adolescents) then I think they will surely use this.

Reference 2 - 0.74% Coverage

P4: They will surely fill this up. But the question is, what are you going to do if they are at high risk of depression? Suppose I am an adolescent and I come to know that I am at high risk of depression after filling up the form. Now what? The adolescent won't go to their parents and tell them that they are at high risk of depression. Only few of them might go and tell it to their parents that they have been seen at high risk of depression but not everyone will do it. This concept (Of risk calculator) is very good to know the status of risk of adolescent but if the result is mild then we have to think "what's next!"

P7: May be that will be also there…

Reference 3 - 0.67% Coverage

P4: There could be the information about where to go like if you have mentioned it as "You are at high risk of depression so please visit the psychiatrist once", the older ones could visit them but here we have targeted adolescents. Their age group is 10 to 19 or here in your research it is 10 to 24. But the adolescents of age group 10 to 19 would visit the psychiatrist themselves by informing this to their parents? So, for this, you have to think about something, at least a backup. I think so.

P7: There could be some key points…

P4: They won't go (To psychiatrist) if they have problems,

<Files\\KII\\ > - § 8 references coded [6.87% Coverage]

Reference 1 - 0.44% Coverage

Just like before making paracetamol, we test its effectiveness, we also should test the effectiveness and validity of risk calculator. There must be a psychiatrist to check whether the cases detected by the risk calculator are accurate and whether it's valid. But it is not that difficult if we focus and make it better.

Reference 2 - 1.08% Coverage

For the development of this calculator, we would need person who have been working in the field of psychosocial and mental health so as to know the components necessary for developing the calculator. And there must be a psychiatrist to validate the tool. For example, if we use the tool here and identify depression in an individual then we must refer him/her to that psychiatrist so that s/he will again use the tool to know if that individual really has depression. By doing this, we can validate the tool. It's not that only the psychiatrist can use the tool to validate but the reason behind psychiatrists using it is they already have enough knowledge about mental health as they have been working in this field. And for using the tool in mass, the tool must be validated.

Reference 3 - 0.39% Coverage

Yes, We can develop and validate the tool. Till now, we are using the tool that has been developed in other countries as they are the validated tools. But we have not developed our own tools and used it. So, why don't we make such tools by ourselves and use it once we validate it?

Reference 4 - 0.99% Coverage

We have to think about confidentiality as well because if there is another person beside them then they can't open up and administer all of their feelings. It could be interpreted in a wrong manner. It has both risk as well as benefit. Till the time adolescents are filling up the form, they will feel nothing but when the results pop up, there is equal chances of them being positive and negative. You have mentioned about the things that could be done for prevention so may be adolescents will take it positively or maybe they could not! But if we mention that depression is not that major problem and it could be prevented and cured if we follow some of the measures then may be adolescents could be calmed down.

Reference 5 - 1.24% Coverage

We can't link the thing that parents themselves are the risk factor for their children's depression because the adolescent open up with us on the basis of trust and we can't expose his/her feelings with anyone. Taking consent with parents is because what we are doing is a research and from research's perspective, we have to take consent from the parents if their children are below the age of 18. We take consent with parents in such case describe them what we will be doing but we don't open all the questions in front of them. If the adolescent have suicidal ideation and it is all because of their parents then we have to inform their parents about it. We also include these things in our consent form. Though we find out the fact we won't tell the parents that their children are depressed because of them, instead, we provide service for the adolescents and do things to cure their depression.

Reference 6 - 0.61% Coverage

P: Yes! To know the risk is something that could benefit both adolescents and their parents. We have to take consent from the adolescents as well as their parents and there could be some risk factors from among the parents too. We have to provide them the service if needed as this is in our ethics as well. If the depression case is suicidal then we have to inform their parents, else the consent from adolescent will work for doing these all.

Reference 7 - 1.25% Coverage

P: We can't link the thing that parents themselves are the risk factor for their children's depression because the adolescent open up with us on the basis of trust and we can't expose his/her feelings with anyone. Taking consent with parents is because what we are doing is a research and from research's perspective, we have to take consent from the parents if their children are below the age of 18. We take consent with parents in such case describe them what we will be doing but we don't open all the questions in front of them. If the adolescent have suicidal ideation and it is all because of their parents then we have to inform their parents about it. We also include these things in our consent form. Though we find out the fact we won't tell the parents that their children are depressed because of them, instead, we provide service for the adolescents and do things to cure their depression.

Reference 8 - 0.87% Coverage

P: We could write something like this; depression could happen to anyone and it is most prevalent in this age group (Referring to adolescents) or some other age group whichever is the fact, depression could be cured. So if we write something like this then it could help the person normalize the situation. Seeing the result of high risk of depression suddenly, the person might get more curious and after reading such kind of information then s/he could be calmed down and the situation will be normal. Talking about the result of low risk, the information you have put is enough, there won't be anything that could be added.

<Files\\KII\\ > - § 1 reference coded [0.45% Coverage]

Reference 1 - 0.45% Coverage

P: ummm, don't know about the area which could benefit but if we make use of risk calculator we can identify which places has high depression.

<Files\\KII\\ > - § 9 references coded [6.24% Coverage]

Reference 1 - 0.77% Coverage

From my point of view, I think this is great work. I don’t think, in Nepali language, this kind of tool were ever developed. The tool will also be very helpful to those who can read Nepali but does not understand English. This tool will be very helpful to make people understand about kind of problems they are suffering with and at what is the risk level of those kind of problem. Talking about its applicability, in urban areas and where people have high access towards information and technology, with no doubt, I think this tool can be used effectively. But, does information and technology has its access everywhere?

Reference 2 - 0.79% Coverage

You asked very good questions. Let us start with good ones. This tool can be used by anyone, even the person who knows only Nepali can use the tool and benefit from the information. Because of this, positive message will spread in the community. Similarly, at home and family level, it can be useful. As said previously, this kind of tool has not been developed in Nepali so far and this is great step. May be with growing time, when tool will be actually implemented in large scale, challenges associated with this could be explored more and correct accordingly. But at this point of time, to say from our side, I think the tool is very good.

Reference 3 - 0.72% Coverage

From my understanding, the tool can be used in general population i.e., the general population of that age group. When the screening gets completed then people falling under low, moderate and high risk might need (ummm), to say for the reduction of the problem family and community roles can be activated. If anyone is from low to moderate category then he could be provided with health related information's through counseling. Similarly, for if anyone is at high risk then he might need medication. If all these services are provided in an integrated approach, that could be helpful.

Reference 4 - 0.77% Coverage

P: From my point of view, I think this is great work. I don’t think, in Nepali language, this kind of tool were ever developed. The tool will also be very helpful to those who can read Nepali but does not understand English. This tool will be very helpful to make people understand about kind of problems they are suffering with and at what is the risk level of those kind of problem. Talking about its applicability, in urban areas and where people have high access towards information and technology, with no doubt, I think this tool can be used effectively. But, does information and technology has its access everywhere?

Reference 5 - 0.75% Coverage

P: From my point of view, for the sake of information purpose I think anyone can benefit from this but I prefer this tool be filled by person suffering or likely to suffer from depression. And, I like the way you have created separate information box which contain information on help seeking and redirecting to different useful links for further tips. In redirect pages, we can find useful information that has relevant information on symptoms of depression, treatment modalities, medication, counseling, psychotherapy, role of family members which will definitely help people associated with sufferer as well.

Reference 6 - 0.80% Coverage

P: You asked very good questions. Let us start with good ones. This tool can be used by anyone, even the person who knows only Nepali can use the tool and benefit from the information. Because of this, positive message will spread in the community. Similarly, at home and family level, it can be useful. As said previously, this kind of tool has not been developed in Nepali so far and this is great step. May be with growing time, when tool will be actually implemented in large scale, challenges associated with this could be explored more and correct accordingly. But at this point of time, to say from our side, I think the tool is very good.

Reference 7 - 0.48% Coverage

P: This is one kind of survey, public opinion. In future, for good cause, at government's policy level also we can discuss about use of this app and its process and how this can be incorporated in any kind of decision making at government's level. Talking further about obstacles, how can this be useful to illiterate one? This will not be useful in those areas where there is no electricity?

Reference 8 - 0.44% Coverage

P: Yes. Or after giving information to him then he can go to seek services from service providers from different centers on his own. In condition where parents/ guardians needs to be called upon, service provider could ask with the respondent about who he feels comfortable to talk or share his problem with. I think we can go through that method as well.

Reference 9 - 0.73% Coverage

P: From my understanding, the tool can be used in general population i.e., the general population of that age group. When the screening gets completed then people falling under low, moderate and high risk might need (ummm), to say for the reduction of the problem family and community roles can be activated. If anyone is from low to moderate category then he could be provided with health related information's through counseling. Similarly, for if anyone is at high risk then he might need medication. If all these services are provided in an integrated approach, that could be helpful.

<Files\\KII\\ > - § 2 references coded [2.05% Coverage]

Reference 1 - 1.03% Coverage

P: If we look at it positively, we can say that they will be more conscious; they could think about the risk factors and also think about the ways of minimizing them. And again, not everyone's perceptive is same. If someone is anxious then they might get scared after knowing that they are at high risk of depression. They might think about it every time, get scared and may stopped working. There are positive as well as negative parts.

I: What can we do to minimize or reduce such challenge?

P: For reducing this, we have to give them clear statement saying that it is only about the risk factor assessment and it is not sure that you will have depression and also say them that if they want they can go to the experts for preventive measures.

Reference 2 - 1.02% Coverage

P: Disclosing about their children's risk of having depression is in the hands of their parents. And again, it is not sure that they are going to be depressed, it is just about risk. It helps in the prevention of depression. It is far better to give information rather than hiding it from them.

I: And do you think there could be negative consequences in the family because of stigma?

P: I don't think there could be any negative consequences. It is again about the person's personality and the way they perceive things. Parents may also be anxious and may get over involved in their children's life because of fear; they may not allow their children to go out because of fear. But for this, we have to provide them proper information.

<Files\\KII\\ > - § 8 references coded [9.50% Coverage]

Reference 1 - 2.07% Coverage

I: So as far as if, for within that, if we stuck with this idea of a broader generalized tool what about that, so that might still be, it sounds like they are here now. What do you think about

using a tool even like that to predict in the future, do you think that it will be helpful to say if on an individual level an adolescent come and you say, ok based on your environment, and your resources XYG you may have a chance to get depression in your tool and do you think it might help adolescent do things like exercise more or wanna be better or do you think it will be more harmful? Do you think that it could make them feel worse or go home and may be their parents don't, oh! Now you gonna get depression so stay away from me kind of things. What do you think of something like that, something that predicts in the future?

P: Ok, thus is very ideal condition if it was there. If we have enough data and we have certain models that can predict depression that will be very good. So far I don't know, I am not sure about that. Are we there, there is always depression. We don't have evidence for that but still we can say these are the issues and having these issues might develop so let's prevent that can be helpful but this model should be used in caution because sometimes you are trying to pathologies normal life suffering and distress. Just like after earthquake, many newspaper started saying that ok now mental illness is going to rise up (laugh) and ok, PTSD is going to rise. We had to tell everyone even in our association. We set up some guidelines within a saying that ok normalize this fear, this is quite normal, this is normal fear in the time of abnormal situation. So, we have to, we can use this but with care.

Reference 2 - 0.46% Coverage

I: So what are the potential benefits of using such calculator?

P: By using such tool, they could be able to refer themselves to the service centres. Even government have planned to keep one nurse as a school counsellor in each school, where adolescent could go and talk about their risk of having depression once they know about it after using this tool. So, these are the benefits.

Reference 3 - 3.11% Coverage

I: And as you said, for those with high risk, maybe we could provide some information about some interventions for preventing mental health problems and maintaining mentally healthy lifestyles.

P: Yes. According to me, I think its utility could be in school mental health programs where the results could be known by the professionals or the researchers only but still we could provide some kind of general information to every one once they fill out the form. This could be in the case where they might not get to see the result by themselves.

I: Can we do so?

P: Yes!

I: Is there something in research ethics where it says we should reveal the result to the participant or something like that?

P: We could tell them to ask individually and know the result if they want. But we have to tell the generic message to all of them. We can reveal results immediately to those who have access to services near them. For example, if someone is in high risk and they have school counsellor in their school then we can tell them that they are at high risk of depression so that they could immediately go to school counsellor for counselling if needed but where people don't have access to such facilities and yet know the result, they might get scared and might have negative consequences in them. So, for them, we can provide some generic information about mentally healthy life style and preventive measures of depression instead of directly showing them their result of risk. Just imagine, if someone tell you that you are at a high risk of depression then what would be your reaction? How would you feel? You would obviously feel stressed (Laughs) but those who have access to services could be calmed down and might not be a victim of negative consequences. We might also get some cases of high risk of depression where we could look from the parent's or teacher's perceptive and know if they are at risk, because obviously very high risk of depression could be seen from other's view as well. And for the other general population, teachers or nurses or other person could use this tool up on them and know the result but don't reveal it to the adolescent and start the intervention where needed and instead provide generic feedback or message to every one so as to make them aware and prevent depression. Among 100 students at school, there might be 5 of the students who could be at risk but for those 5 we can't risk those 95 students' life because this is a very sensitive issue. We have to choose some way to do this so that no one could be at risk of negative consequences of using this tool.

Reference 4 - 0.38% Coverage

P: By using such tool, they could be able to refer themselves to the service centres. Even government have planned to keep one nurse as a school counsellor in each school, where adolescent could go and talk about their risk of having depression once they know about it after using this tool. So, these are the benefits.

Reference 5 - 0.75% Coverage

P: There is also a different thing. It could be extra trauma for the family. We have said that it is high risk but for parents, it might be very huge issue and they might think that their children are going to suffer a lot. There are both positive as well as negative consequences. We have to see the consequences for the adolescents once when they know that they are at high risk of depression. If I am an adolescent who always comes first or second in class and I am anxiety prone, what would be my reaction after knowing that I am at high risk of depression? Not depression but my anxiety level could increase. So, we have to…

Reference 6 - 0.12% Coverage

I: We have to look at how an adolescent would perceive the result.

P: Yes, it depends on that as well!

Reference 7 - 0.33% Coverage

P: Instead of the result of high risk, we can directly mention some intervention for them and tell them that they might be at risk of depression so they have to follow those approaches. Because we are not sure that s/he would surely have depression even if they are at high risk.

Reference 8 - 2.27% Coverage

P: We could tell them to ask individually and know the result if they want. But we have to tell the generic message to all of them. We can reveal results immediately to those who have access to services near them. For example, if someone is in high risk and they have school counsellor in their school then we can tell them that they are at high risk of depression so that they could immediately go to school counsellor for counselling if needed but where people don't have access to such facilities and yet know the result, they might get scared and might have negative consequences in them. So, for them, we can provide some generic information about mentally healthy life style and preventive measures of depression instead of directly showing them their result of risk. Just imagine, if someone tell you that you are at a high risk of depression then what would be your reaction? How would you feel? You would obviously feel stressed (Laughs) but those who have access to services could be calmed down and might not be a victim of negative consequences. We might also get some cases of high risk of depression where we could look from the parent's or teacher's perceptive and know if they are at risk, because obviously very high risk of depression could be seen from other's view as well. And for the other general population, teachers or nurses or other person could use this tool up on them and know the result but don't reveal it to the adolescent and start the intervention where needed and instead provide generic feedback or message to every one so as to make them aware and prevent depression. Among 100 students at school, there might be 5 of the students who could be at risk but for those 5 we can't risk those 95 students' life because this is a very sensitive issue. We have to choose some way to do this so that no one could be at risk of negative consequences of using this tool.

<Files\\KII\\ > - § 6 references coded [3.96% Coverage]

Reference 1 - 1.04% Coverage

P: What will we suggest the adolescents after s/he completes filling out these questionnaires?

I: If the adolescent is in high risk then there is the information about treatment or intervention and about the referral services for them to use.

P: Do s/he refer themselves to the service providers?

I: Someone is there with them when they complete this set of questionnaire, so, what should be done? Is it better if we display the result to them or not?

P: We have to give them the choice by displaying the results to them.

I2: So, we have to display the end result with them?

P: Yes. S/he may or may not want to go for the treatment, it’s their choice.

Reference 2 - 0.95% Coverage

I: So, for those with high risk, we should go with some modality of treatment? And for those with low risk of depression, what should be done?

P: Rather than treatment, we have to recommend them to the service providers?

I: And what could be done for those with low risk of depression?

P: If s/he is in low risk then why do we need to provide treatment? What is the actual prediction for this? If a person is in high risk then is it that s/he may develop depression or have a risk of having depression?

I: S/he have the likelihood of having depression in near future. It is about the probability.

Reference 3 - 0.25% Coverage

P: We could give it to themselves so that they could look at their risk of depression by themselves. If they are at high risk then they will refer themselves.

Reference 4 - 1.22% Coverage

P: Again, it depends on the purpose of the study you are doing. Are you doing this for research purpose, are you doing this for screening purpose or are you doing this for treatment purpose, these should be predefined before developing the any scales. But whatever the purpose is, if we find someone with high risk of depression, we need to find some way to recommend them to treatment. If we screen and find 50 people having depression, where would we send them for treatment? We don’t have any place for the treatment. Are we going to screen people with depression and then tell them that you don’t have any place to go for treatment? This is also a

question. So generally, if we find that somebody have a high risk of depression then we need to find some ways to help.

Reference 5 - 0.35% Coverage

I: So, for those with high risk, we should go with some modality of treatment? And for those with low risk of depression, what should be done?

P: Rather than treatment, we have to recommend them to the service providers?

Reference 6 - 0.15% Coverage

P: If someone is in high risk of depression then you should screen them and monitor as well.

<Files\\KII\\ > - § 3 references coded [1.40% Coverage]

Reference 1 - 0.85% Coverage

We can do it after training…I cannot be sure. Since this is just a prototype and it's just that my work has to be done. So, I cannot be sure. After doing this also, I think for some time to know that actually there is risk? You need a long of duration of time because you are talking about the future. If someone is said to be in risk now, you have to look for them after 5 years or 6 years and you have to do it with a lot of people. Well that will be research and after that knowing how valid and reliable it is, then after that maybe you can train people.

Reference 2 - 0.16% Coverage

So, we can use such tools. If it is so… in future …

P: If psychometric strong then we can.

I: Yes. Okay.

Reference 3 - 0.39% Coverage

Yah, a lot of people will fit into it. And in such scenario … If a person comes under high risk then a lot of people fall under that category (As in the scenario is same in our countries' setting). So, among those 10 – 15 people how many could have depression?

<Files\\KII\\ > - § 1 reference coded [0.90% Coverage]

Reference 1 - 0.90% Coverage

I: Do you think that such tool would be useful if it is developed?

P: According to what you said, I think the tool would be useful because we can know about the individual's life experiences according to what s/he fill up in the form and find out whether s/he is at risk of depression. So, yes, such tool, if developed, will be useful.

<Files\\KII\\ > - § 4 references coded [5.99% Coverage]

Reference 1 - 1.64% Coverage

I: We talked about showing the result at last. Is it better to show the results to the adolescents who fill the form or should we inform about them to their parents or school?

P: If you reveal the result to the individual, they may not be able to think in correct way at that age of their life. If you can inform school or teachers, it will be even better and we can act for it accordingly. Otherwise, you can inform it to their guardians but if show the result to the children, there can be more negative effects. It means that after the information is filled up, it will be better if we tell the result to the teachers or guardian.

Reference 2 - 0.32% Coverage

I: So, it will be better to reveal the result to the teacher once after the adolescent finish filling up the form?

P: Yes!

Reference 3 - 3.49% Coverage

I: Teachers might be teaching related things in the school or due to being educated and having knowledge, they might understand but what is the possibility that the guardians will have negative effects because their children can have depression in future?

P: There is possibility. It also depends upon how mentally strong the guardians are. In addition, I see the risk of guardians getting into the problem due to their children.

I: What can we do to control such problems? It is sufficient if we keep only that information or should we add anything?

P: Guardians should be aware and given general information about the possible risk of depression, their children has so that the risk level would not increase.

I: Is it sufficient if we inform them last as, “being in high risk, you should take initiative for help” or “you are found to have low risk so, you should have healthy food and follow these instructions” or should we add anything?

P: I think, you should focus the high-risk group more. If I have known more about this, I would have been able to tell on this. Neither we need to teach about depression and nor I know its symptoms; therefore, I cannot say more about it. However, you should tell the low risk ones that they are at low risk of depression and address high-risk group more to say about the initiatives they should take.

Reference 4 - 0.54% Coverage

I: If they are at the high-risk group, I think it is enough if we first of all, make peaceful and then tell that they have high risk of depression and should take following initiatives. Isn’t it?

P: Yes.

<Files\\KII\\ > - § 8 references coded [7.24% Coverage]

Reference 1 - 0.62% Coverage

P: Such tool will definitely be useful as you have tried to include the risk factors for depression and measure the risk of depression of an adolescent in near future. So, it will be useful for sure. It helps to understand issues from nearer.

Reference 2 - 0.73% Coverage

Once a person is aware about what had happened to them, they will explore and try to seek help to minimize the problem within them. They can visit psychologist or other service providers if needed as you have thought to mention about it in the tool through the link or contact number.

Reference 3 - 0.77% Coverage

P: People at suicidal risk are negative to self, society and others. If this is easily available and if there is not any person for monitoring, sometimes the person belonging to risk group can perceive that he or she has depression and thus they can be more negative and practice self-harming behaviors.

Reference 4 - 1.79% Coverage

P: It can be developed similar to the way other check lists like BDI, BAI and other logical tools are made. There might be argument that the tracing could be used even when others are not there but according to my personal understanding, if a person already has negative thoughts, he or she can perform some negative behaviors and thus it can provoke risk taking more than protecting. As the people with suicidal ideations can also take suicidal news positively and so does the research shows. It is said that due to incorrect reporting of suicidal cases suicidal case has increased by more than 40%. Though we are going something for wellness, sometimes there can be negative effects do in such a way.

Reference 5 - 0.63% Coverage

Therefore, after the family is aware, the family members will also look after those who are at high risk or I think it will be better either if we can use them under direct supervision of someone or conduct in the form paper-pencil tests and so on.

Reference 6 - 1.71% Coverage

P: I think the result could be somewhat affected because there is some stigma towards mental health and due to the same, they might not tell the negative consequences. Even teachers might not know the possible consequences of having high risk of mental health problems and thus they can hide the information. Even though they don’t know about which information gives what result but while saying the points of rating scale, they will have already known positive and negative things in a level. Therefore, sometimes biased information might be provided from parents because in our cultural context, somewhere the parents are not aware and they tend to hide the information.

Reference 7 - 0.63% Coverage

P: They might hide the information. Parents might refuse to calculate the risk of their children saying that nothing has happed to them and they do not want to do so and say that their children are completely fine and nothing have happened to them.

Reference 8 - 0.35% Coverage

Similarly, if a person with high risk spend much time in internet and not interact with others, we can aware them about the possible risks.

<Files\\KII\\ > - § 2 references coded [1.81% Coverage]

Reference 1 - 1.50% Coverage

I: Ok, yeah exactly. So let me see…do you see any challenges or any potential negative effects of this questionnaire?

P: No, I think it's even…it's actually more important for them to know about those signs and symptoms. Like, what all can be a factor for depression, we have discussion I saw in this version so…I think those factors might contribute to depression, so they will know about them.

Reference 2 - 0.32% Coverage

I: So you think it could almost be like awareness too, even if they're not—

P: Yep.

<Files\\KII\\ > - § 1 reference coded [0.38% Coverage]

Reference 1 - 0.38% Coverage

P: Well, if it's "high risk," then definitely. I mean, I don't know, maybe it depends. We might need to take an assent from the adolescent. Like, when we start asking them questions, we let them know at the beginning that it's confidential and all, but if we believe that there's a risk to your safety or health and it has to be disclosed, then it might be.

<Files\\KII\\ > - § 1 reference coded [0.74% Coverage]

Reference 1 - 0.74% Coverage

P: It can be able to be used at school and teachers could possibly use it because they might have identified it. Similarly, in the community, there can be such things in the family and we say for some of the children that they have habit of not telling such things. However, family will know more about their children, as extrovert or introvert more than the teachers in school. Therefore, I think we should have 2-way communication with family. We can know it exactly if I can do it in school and then simultaneously in the house. We should have some teacher's view as well as some family's view so that, we can find out the gap.

<Files\\KII\\ > - § 4 references coded [4.95% Coverage]

Reference 1 - 1.53% Coverage

I: We have not worked out the questionnaires yet. We have made a plan to prepare the questionnaires after we assimilate the risk factors and prepare questions with regards to the risk of depression.

P: If all those questionnaires are included in it then I think that it will be beneficial. That is because we are simply asking them and managing it at the moment. We do not have any tool to find out about the depression. I am simply asking them whether they have any of the symptoms common to them. We are doing so because that is the level of knowledge that we have. We are doing so at the moment. If we were to get it in brief then we would know about them in detail. We will get to know more about them and not restrict ourselves to just the 4 things that we might ask them. That is why I think that it will be beneficial.

Reference 2 - 1.33% Coverage

P: They will take the necessary precautions. They will know that they are not supposed to act like that if they learn about it beforehand. They will go for the counselling after they know that they are at risk. This raises the primordial preventions. If we conduct the assessment using the questionnaires that you have prepared, then we will offer them counselling if we learn that they are at risk. They will become aware if we let them know that they are at risk. They will be aware about the things that they should not do after they learn that they are at risk. If we are able to make even a single client who is at the risk of depression aware, then it is a big thing because we will be preventing the loss of a life.

Reference 3 - 0.32% Coverage

P: Yes, this focuses on prevention which is clearly good. Prevention is better than cure This is something that we have been learning and following. That is why this is good.

Reference 4 - 1.77% Coverage

P: First of all, they will become anxious. The children will get scared that they are at high risk fearing that something bad will happen to them. Rather than just giving them the results, we need to explain what the disease is and what the possibility of getting it is. We need to speak positively with them and tell them that there is a chance that they will not suffer from it. When talking to the children, we should go from simpler to complex. That is how we should explain to the adolescents. We need to reassure them. We need to tell them that they are not going to lose their lives if they have it like other physical illnesses. They can take counselling and medication for it. This does not mean that they will have this disease for sure but they are only at its risk. We should tell them about the things that they need to avoid to reduce their chances of getting it. If we are able to counsel them as such then they will be able to relate it a little.

<Files\\KII\\ > - § 2 references coded [0.78% Coverage]

Reference 1 - 0.66% Coverage

P: When talking about awareness, having a discussion in front of everyone in the general open forum will help normalize it. There is a lot of difference in looking at this individually and looking at this collectively in a group. If we normalize this and make them believe that it is a regular illness within a school setup or a family setup, then it does not matter (to the patients) what results they get. There has to be mass awareness.

Reference 2 - 0.11% Coverage

I: So we can use this tool effectively if these things were there?

P: Yes.

<Files\\KII\\ > - § 2 references coded [1.72% Coverage]

Reference 1 - 0.28% Coverage

P: In this way an adolescent can know about them and their condition like normal, high risk or low risk.

Reference 2 - 1.44% Coverage

P: As you told before, if a person is found to be in low risk group, he or she doesn't need the medication, doesn't need to face many problems, can reach the concerned place in time or self-care himself or herself. If someone is in risk, he or she will feel of being in such level and needing someone's assistance. He or she can reach the concerned place and even if he or she is at high risk, he or she can get treatment service. This makes them get more or less *rahat* (relief) from the condition people having medicines have.

<Files\\KII\\ > - § 6 references coded [2.37% Coverage]

Reference 1 - 0.17% Coverage

P: Yes, this would work. We can take a look at their level.

Reference 2 - 0.50% Coverage

P: If they were to select their behaviors correctly [truthfully], then they would get the different aspects (connections) of support. Then, they would be motivated to improve.

Reference 3 - 0.33% Coverage

P: Yes, they would be. After there is the checking of what level they are at, they will try to increase their level.

Reference 4 - 0.67% Coverage

P: If it was measured, then… The treatment that they need according to their level… First of all, the adolescent could be motivated. And after their level has been identified, it will be easier to provide them with further counseling

Reference 5 - 0.28% Coverage

I: To provide counselling at the initial stage?

P: Yes, it will be easier at the initial stage.

Reference 6 - 0.41% Coverage

P: At the initial stage, after it has been implemented it will be effective when calculating. I feel that it will give us positive results.

<Files\\KII\\ > - § 4 references coded [4.37% Coverage]

Reference 1 - 0.29% Coverage

P: I would have answered about it better if I had seen it top to bottom once more. However, according to the design, I think it will be useful.

Reference 2 - 0.35% Coverage

I: We have presented few questions only as an example but we still have to put many things after literature review. Anyway, you think it will be useful, isn't it?

P: Yes

Reference 3 - 1.92% Coverage

P: First, the designed questionnaire could be a convenient tool for us for assessment. Second, a new tool will be developed which is good for professionals. Third, if we see from the prospective of client or those who seek support, they can understand what their problem are and where they can go for the betterment. It contains information about high and middle risk and it contain materials by which they can get information. Rather than doctor's consultation and counseling, they will get preliminary information, which is good in itself. We also talked previously that a person might not know whether he or she is having depression symptoms. So, at least, they will understand about their problems and where they should go. Therefore, this tool will be better because firstly, a new tool will be developed. Secondly, identification is possible and thirdly, patient will also get information about what they could do for reducing their problems.

Reference 4 - 1.81% Coverage

P: As this is a type of individual assessment tool and depression, being a sensitive matter in our checklist or information process, I don't see any difficulties to use it in school but the setting of school should be easy to talk and response. Even family can be a better setting but we can ponder upon how the person stays and so on while collecting information. It can be less useful in the clinical setting because the cases which has already been assessed and the severe cases or high or medium risk cases will reach to the clinical setting whose symptoms are already observed by their family. So, those cases are already diagnosed at a level. For professionals, it might be useful to use the tool for final verification. However, if we are targeting large population, school or house can be a better place to use the tool so that individuals can know about their status and risk level.

<Files\\KII\\ > - § 3 references coded [3.04% Coverage]

Reference 1 - 0.34% Coverage

P: I think that identification becomes easier and effective if we will have such tool, which contains overall things.

Reference 2 - 1.21% Coverage

P: In our society, people feel ashamed to talk about mental health so, if they can find out their problem, the individual can himself seek if he has such problems and what he could do to solve it. He might be able to decrease his problem if he is in low risk. Similarly, he might be able to reach the concerned place for treatment with reference to the contact details you provide, without exposing his problems to others

Reference 3 - 1.49% Coverage

P: Yes, and the concept towards mental illness will also change because if an individual can use the app, he or she will have knowledge that mental health is not bad subject. If someone has to go to a separate place for doing the same thing, he or she might consider that such place is for such patients. If they can do it themselves, they will know about it and accept that a normal person can use that, normal person, can also get have such problem and thus the concept of people towards mental health will change.

<Files\\KII\\ > - § 3 references coded [2.87% Coverage]

Reference 1 - 0.87% Coverage

Well, it can be early pick up and if we do timely counselling we can prevent depression and that is very nice. We can identify depression earlier…

I: Yes

P: We get to know everything beforehand and that is nice thing.

Reference 2 - 1.56% Coverage

Like whatever the problem is first to that only...like…You…like others…if a person has been involved in smoking or substance abuse then at first that person should be informed about their problem and make themselves understand about it. Then only we can ask them to seek help. We have to communicate with them first. This (information in the tool) is generalized, so I think it is not enough.

Reference 3 - 0.44% Coverage

If it is in high risk, it is the issue of tension for everybody. Yes it happens and yes for parents it happens.

<Files\\KII\\ > - § 1 reference coded [0.90% Coverage]

Reference 1 - 0.90% Coverage

P: What are you doing now? We might not be able to reach homes but sometimes during group counseling, family counseling, we can discuss that matter. They should obviously know about the paramedical. There are important indicators for the calculation of risks so that we get to know on time and through preventive measures… Rather than the curative, children should be prevented from the risk of depression through preventive measures.

<Files\\KII\\ > - § 1 reference coded [0.08% Coverage]

Reference 1 - 0.08% Coverage

P: It would be obviously useful.

<Files\\KII\\ > - § 2 references coded [7.01% Coverage]

Reference 1 - 4.67% Coverage

P: The one thing is they will not share immediately when asked. There is problem of doing through quantitative tools with adolescents, it is biased. When you build rapport and maintain close relation, they might be. In urban areas, people might open-up but in rural areas, people do not openly share (their problems). If somebody has fewer problems, they might open-up. After someone opens up, if they find out that s/he is in problem, in risks and realizes that s/he should undergo the treatment, then some may take it positively as well. But if somebody has not realized this and collides suddenly, then they can take it negatively. S/he is not familiar with depression and if we are unable to make him/her understand that depression is just like other physical problems and considered that the mental health problem is like the person being mad, then that will be destructive. But we can convince them that depression is not a problem and it can be cured by taking medicines for certain period of time. Then, that would have positive impact.

Reference 2 - 2.35% Coverage

P: They can conceive negatively. If the reason is not a family, then why should talk within the family. We will find out the reason behind. That might be because of peer. If related to school, then it can be discussed in school. If family is the reason, then we can convince saying these things are out, so be careful. If you tell them for instance, this is because of his personal reasons. He has relation with this kind of girl which is why these things are happening, what if they hide their son away from their own home?

<Files\\KII\\ > - § 1 reference coded [1.13% Coverage]

Reference 1 - 1.13% Coverage

P: I think it would be good, it would aware. It will create an environment to care for oneself about realizing the risk. If the risk is low, considering that “now I am at low risk, if I feel difficulty more than this, I can have a high risk”, one can take precautions. I found it good. It is an innovative idea.

<Files\\KII\\ > - § 2 references coded [2.08% Coverage]

Reference 1 - 0.55% Coverage

P: I found this very impressive. High risked people will know about it before it actually happens, right? In that sense I find it very interesting. I also have some questions but I'm keeping it for later.

Reference 2 - 1.53% Coverage

I: Yeah, we are doing this in a sense that if they are cured due to questions of high risk it will be more effective in the future. Any more suggestions that you want to add to this?

P: So, major one is the same that we found out the problem. But for the solutions, are there any expertise in this country or not. And the fact that there are no expertise in this country is out of our hand. So the problem is figured out but we don't know what should be done next because it's not within our control. It is easy find out the problems it will be hard to find the solutions.

<Files\\KII\\ > - § 3 references coded [2.20% Coverage]

Reference 1 - 0.49% Coverage

P: Maintaining this is the main challenge. What to offer them? Because if we are giving a phone number, link, what would be their role. It is normally referring to go there or they will provide some service there. This would be like a project.

Reference 2 - 1.10% Coverage

P: I think sustainability is the biggest challenge. Getting service is the main thing. The service should be available. We have less. If service is made available, that would be very good. We ask them to go there, that is very good considering that they are not taking service as they were not known. But even if we let them know, they go there and if the service is available or not, how is the charge, time; opening time there, thing about the privacy, how assured they can be, considering all this the matter is if they will go there or not.

Reference 3 - 0.61% Coverage

P: This way, making them come there is a challenge and making the service sustainable is also a challenge. The resources should be available, commitment should be there. There should be a

mechanism to provide the services. That is challenging. I do not know the details but there should be something...

<Files\\KII\\ > - § 1 reference coded [0.43% Coverage]

Reference 1 - 0.43% Coverage

P: There is the issue of high risk and low risk that you had mentioned. Maybe they need to take that medicine their whole lives if they are at high risk…

<Files\\KII\\ > - § 2 references coded [2.24% Coverage]

Reference 1 - 1.49% Coverage

P: It's good. This is not only useful for teenagers. It is useful for everyone even if it's specially focused on teenagers. It relates with everyone's life. Many behavioral things can be small or big. Some teenagers can be under behavior and some can be spoiled due to more love of the parents (*pulpuliyeko*). And some can be depressed because they don't get that love which is needed. So these things which you are trying to cover it tentatively good!

Reference 2 - 0.75% Coverage

P: Like I said earlier, you can add those things. If you do this in schools of villages also through government organizations or any clubs, I think not only the students will learn but many other people can also learn from this.

<Files\\KII\\ > - § 1 reference coded [1.51% Coverage]

Reference 1 - 1.51% Coverage

P: It helps in early detection of mental health. If there's early detection there will be awareness, there will be awareness to the parents, and they will be responsible for the treatments. Everyone will get the information and it is also useful later to make policies and programs in ideal budget.

<Files\\KII\\ > - § 2 references coded [3.02% Coverage]

Reference 1 - 1.99% Coverage

P: If it predicts the risk, then that would definitely be beneficial. The most important aspect is if the risk would be predicted or not. For Example: At present, that can be clinically observed… even if we talk about the disease that can be observed by biochemical parameters such as heart disease, prediction about who is at the risk at the heart attack cannot be done. A few new tools might… It might could be said, how much the person is at high-risk, how much… is, what is the condition of modifiable factors. That is clear-cut. Thing might be about cholesterol and other. Risk prediction… chart… If that could be said in depression, then that is excellent. How possible is that? Let us see what literature say.

Reference 2 - 1.03% Coverage

P: If the thing (tool) is really good, implementation is definite. People are working even when good things are not available. It is fortunate for having a chance to use good, well-stated… We are talking a lot about depression. If any tool can be developed for risk prediction, what would be the bigger achievement than that? However the question is how possible is it.

<Files\\KII\\ > - § 3 references coded [10.41% Coverage]

Reference 1 - 4.94% Coverage

P: When I first heard the word predict, I had thought it would be different. Basically, this is adverse life experiences… What are the adverse life experiences of children at the current time are the proven factors for depression? Further, what I think is score… a total score is at one place but I think the severity of each domain also determines. For example: the person has been through sexual harassment. Everything is fine with him/her but has been through sexual harassment. I have not seen that (risk calculator) in detail. But these sorts of issues should also be given weightage/consideration. This is important. We ask these in clinical history as well. When this ask, children… Are there current symptoms or not… I think this should be weigh/consider with strength. Children’s strength… In a lot of cases, children have excellent coping skills but have stress. What it shows is at high-risk. However, if the child has good psychological strength… That is why I think this should be weighed with strength as well. Along with the risk factors, many supportive factors also exist, right? Should weigh with these things which can negate? That is one thing. The other is these types of tools… I think this should be useful. The referral might increase in our contexts. It might be useful in a sense that though this is not highly specific if could be even sensitive then it should work.

Reference 2 - 2.53% Coverage

P: Now this is like… What it can do is… We have been looking to strengthen the service in regards to child mental health. Our expectation is that the people will come if the service is available

which I think is almost false. Nevertheless, the point is that at least the service should be there. Our current focus is one who comes should get services. We are needed to do that as we do not have services now. Our challenges will be when people do not come despite the services are available. This is needed in the second phase when we will work for 2/4 years and people won't be coming. It means children's rating on own, general people rating in school, I think these complements to promote help-seeking behavior.

Reference 3 - 2.94% Coverage

P: The psychological coping is being so much simplified that it is not necessary to visit health workers all the time. My point is people might not come. In several settings, self-help packages are available. That can be made used too. WHO (World Health Organization) had engaged from TPOs only, you might not know. Developing online-based self-help materials, which might be the YouTube videos, chat-bot and so on. The discussion is on a process to include issues like self-help in evolving digital… targeting urban, and peri-urban adolescents. So in this, children identified the high-risk on own and the further link can be there. If that project comes to us, that might become culture-specific too. We can then directly link those matters… Especially, if adolescents themselves… They will be empowered. They will do that too.

<Files\\KII\\ > - § 4 references coded [4.56% Coverage]

Reference 1 - 1.89% Coverage

P: First of all, this tool should identify the cause of the risks and after the identification of the cause, a policy should be made which addresses the cause. And that should be applied in our behaviours. If that is done, it will definitely have an impact but first it should be found out and that should be shared to our major stake holders. And after sharing there should an environment of warning which means they should work on their programs by addressing it.

Reference 2 - 1.38% Coverage

P: I think only information is not enough! For low risked people it is not a big problem but the high risked people, they should also get the service. Let's suppose you worked this in 5000 people and 100 hundred of them are in high risk. Then he can suicide at any moment, so he should get the service. It should be supported with service.

Reference 3 - 0.41% Coverage

P: The person should be contacted and assured if he took the service or not. Only then, it will work.

Reference 4 - 0.88% Coverage

P: Nothing like that but if you this tool in the health of adolescents and mental health this may support. If any significant result is found by this may be it will be recommended to take it throughout many districts.

<Files\\KII\\ > - § 1 reference coded [1.66% Coverage]

Reference 1 - 1.66% Coverage

P: Let's say the risk is identified. The person maybe in high risk, medium risk or low risk. After knowing that where will the person go and find the service. We talked so much about how there is no service available for depression. That's why what we say in public health is, If you don't have the solution you should not see the problem. If there's no treatment for a disease, there no use of diagnosis.

<Files\\KII\\ > - § 2 references coded [1.36% Coverage]

Reference 1 - 0.41% Coverage

P: I think it can be useful and it's good but it depends upon how truly the adolescents will use it.

Reference 2 - 0.95% Coverage

P: Option should be given after they are in high risk. Further assistance can be provided online to the high risked people and if there can be further counselling about the ways to cope with that situation I think it will be better.

<Files\\KII\\ > - § 1 reference coded [0.10% Coverage]

Reference 1 - 0.10% Coverage

P: It will be useful, of course.

<Files\\KII\\ > - § 6 references coded [3.51% Coverage]

Reference 1 - 0.09% Coverage

P: Yes, I think it will be very useful.

Reference 2 - 0.06% Coverage

P: I find it really good.

Reference 3 - 1.52% Coverage

I: Is it? What do you think are the benefits of this tool then?

P: When my kids will grow up, they will have their own wishes and desire. And because of the things you said it here (Referring to the doctor in *Kanti* children hospital)…if we don't do those things then after they will grow up then they will…some will even become *Pagal* and leave home or their studies...problem in my daughter is the mental illness and it was because of the family environment. And if tomorrow in future if same thing happens, then it will be more difficult for me because her wishes also got vanished and what she wanted to be in future also she can't be. So, it will be quite difficult.

Reference 4 - 0.25% Coverage

I: That means if we know about it early then we could prevent it to go to such level?

P: Yes, we have to do so!

Reference 5 - 1.21% Coverage

I: After they fill this form, the result would come out. In case of children below the age 18, we have to previously take consent from parents so obviously if they are at high risk we would be saying it to their parents. But in case of children above the age of 18, what should be done? Should we tell their parents if they are at high risk?

P: Parents should be informed about it anyhow. At the beginning, we have to tell the parents what we are doing. And if they are at high risk than their parents should be informed about it.

Reference 6 - 0.38% Coverage

I: Should we tell their parents only if they are at high risk or we should tell them even if they are at low risk?

P: We should tell their parents in both the cases.

<Files\\KII\\ > - § 6 references coded [3.80% Coverage]

Reference 1 - 1.23% Coverage

P: (Interrupted by respondent) Laughing, This kind of model is basically developed by NGO/ INGO. I don’t mean to say that this tool is not effective. Through practice, it can be made better. Through this form fill up, a kind of formality is met and documentation is assured. On the basis of this, we can carry on analysis. It is like data research. It does not have negative side. But, sometimes I feel that formality steps are carried more rather than taking actions. Analysis becomes good but when action is required, nothing happens. So, I wish the tool you are trying to develop may not repeat this. Because these are NGO/INGO format which are developed for the sake of money earning. Few might earn in transparent way whereas few might not. Some might have good intention whereas some might not have. It is good to know about your effort. In conclusion what I would like to say is it should have some action.

Reference 2 - 0.63% Coverage

P: I would like to repeat the same. It is for sure that we generally conclude something on the basis of reference and plan action. There is no doubt on this. We cannot say in 100% that this person is at risk but that might be possible through categorization under different levels. I think this can be implemented. Experience is great thing. If experienced (this refers to qualified health personnel or trained person) personnel use this then we can expect good output.

Reference 3 - 0.11% Coverage

I: So, you think this kind of tool if developed is helpful?

P: Yes, it is useful.

Reference 4 - 0.13% Coverage

I: So, you find this useful?

P: Yes. Experience and study plays vital role to succeed in life.

Reference 5 - 0.89% Coverage

P: Yes. It is something that is important to every people in society. It is not ethical to laugh when we find other people suffering from the problem. Anyone can be suffering from the problem

anytime. Therefore, not only at individual level but at social level on daily basis it could be of help. There might be other whose child could be handicapped as well. Everyone should be aware of this. This is social support. Everyone should learn on how to deal with them. Problems could be minimized on daily basis and one could tackle problem easily. This is very good and is beneficial to everyone. There is no doubt.

Reference 6 - 0.80% Coverage

P: Ummm challenges!!! What everyone prefer is to see results. Even total population in politics or education wants output. You should become able to make other clear about activities and associated result. Else people will start questioning "why do we need this? We don't want". This might come. No other issue than this might come. The way you explained me, if you motivate and explain in detail to others like what are output, what are social factors, what effort results in what, what is better, what will be more beneficial, and how future will be better, then this will give good output.

<Files\\KII\\ > - § 2 references coded [1.05% Coverage]

Reference 1 - 0.51% Coverage

P: If I would know it before, I think we would not have to face this much of troubles.

Reference 2 - 0.54% Coverage

P: I think it will be very useful…I think if I would know it earlier…my son would be better.

<Files\\KII\\ > - § 8 references coded [1.84% Coverage]

Reference 1 - 0.13% Coverage

P: Yes. Why not? Everyone would be learning from it. I feel one can learn from that.

Reference 2 - 0.14% Coverage

P: umm, this will help them to know faster and they do not have to face difficulties like us.

Reference 3 - 0.26% Coverage

P: Yes, it has advantage as well as disadvantage. Some might be scared. It have both sides. Some will realize and tries to prevent while others might have negative impacts.

Reference 4 - 0.19% Coverage

I: so, what do you think of potential solution?
P: ummm (long pause). I do not think I can recommend on that (short laugh).

Reference 5 - 0.50% Coverage

I: Now, we have only few questions remaining. Sometimes, the guardians seem to be the contributing factors for their condition. So, how can we support them at such situations as we cannot discuss this with their guardians/parents?
P: umm. This is quite difficult (short laugh). We need to inform/counsel the concerned person on this.

Reference 6 - 0.27% Coverage

I: Then we need to discuss with the person and discuss on what can be done.
P: Yes, it can be happening from guardians as well. Therefore, we need to discuss with the person only.

Reference 7 - 0.29% Coverage

I: So, what do you think about advantages and disadvantages? Do you think advantages outweigh disadvantages or vice versa?
P: I think there are less disadvantages compared to its advantages.

Reference 8 - 0.07% Coverage

I: so, it is good to develop these.
P: Yes.

Code Query Output

<Files\\FGD\\ > – § 1 reference coded [0.53% Coverage]

Reference 1 – 0.53% Coverage

P5: Yes. But also, if we have technology and we can use it, why not use it for better, isn't it? It is important to make such things and help to reach it out to media also. About feedback, I have said it according to my experience. I said so as per my experience and my understanding. It can go completely opposite as I said also (Laughs).

<Files\\FGD\\ > - § 3 references coded [0.68% Coverage]

Reference 1 - 0.34% Coverage

P3: In this tool, are the personal information of individual shared?

I: No, it does not! It is very confidential and only the individual who is going to fill it up will know the details and know where to go or what to do.

P6: If the personal information are not shared then adolescents might fill it up.

Reference 2 - 0.27% Coverage

P3: Yes, here it is. I don't think the ethnicity is necessary here, isn't it?

P6: May be the team wants to know about the relation between ethnicity and the problem; in which ethnic group the problem might be higher. This is a need in Research!

Reference 3 - 0.06% Coverage

P6: Yes, there was even a section to mention their name!

<Files\\KII\\ > - § 10 references coded [6.32% Coverage]

Reference 1 - 0.99% Coverage

We have to think about confidentiality as well because if there is another person beside them then they can't open up and administer all of their feelings. It could be interpreted in a wrong manner. It has both risk as well as benefit. Till the time adolescents are filling up the form, they will feel nothing but when the results pop up, there is equal chances of them being positive and negative. You have mentioned about the things that could be done for prevention so may be adolescents will take it positively or maybe they could not! But if we mention that depression is not that major problem and it could be prevented and cured if we follow some of the measures then may be adolescents could be calmed down.

Reference 2 - 0.61% Coverage

Yes! To know the risk is something that could benefit both adolescents and their parents. We have to take consent from the adolescents as well as their parents and there could be some risk factors from among the parents too. We have to provide them the service if needed as this is in our ethics

as well. If the depression case is suicidal then we have to inform their parents, else the consent from adolescent will work for doing these all.

Reference 3 - 1.24% Coverage

We can't link the thing that parents themselves are the risk factor for their children's depression because the adolescent open up with us on the basis of trust and we can't expose his/her feelings with anyone. Taking consent with parents is because what we are doing is a research and from research's perspective, we have to take consent from the parents if their children are below the age of 18. We take consent with parents in such case describe them what we will be doing but we don't open all the questions in front of them. If the adolescent have suicidal ideation and it is all because of their parents then we have to inform their parents about it. We also include these things in our consent form. Though we find out the fact we won't tell the parents that their children are depressed because of them, instead, we provide service for the adolescents and do things to cure their depression.

Reference 4 - 0.34% Coverage

We can't tell the parents that because of them their children are having depression, even from research's perspective, we can't say so. There could be some conflicts later on their life as well and it could even bring some other risk in future.

Reference 5 - 0.12% Coverage

If we tell them that this is confidential then they will be able to fill it up properly.

Reference 6 - 0.56% Coverage

P: We have to think about confidentiality as well because if there is another person beside them then they can't open up and administer all of their feelings. It could be interpreted in a wrong manner. It has both risk as well as benefit. Till the time adolescents are filling up the form, they will feel nothing but when the results pop up, there is equal chances of them being positive and negative.

Reference 7 - 0.26% Coverage

P: We have to take consent from the parents of adolescents with whom we are doing this and also make them aware about the fact that this is not something that one should be stressed about.

Reference 8 - 0.61% Coverage

P: Yes! To know the risk is something that could benefit both adolescents and their parents. We have to take consent from the adolescents as well as their parents and there could be some risk factors from among the parents too. We have to provide them the service if needed as this is in our

ethics as well. If the depression case is suicidal then we have to inform their parents, else the consent from adolescent will work for doing these all.

Reference 9 - 1.25% Coverage

P: We can't link the thing that parents themselves are the risk factor for their children's depression because the adolescent open up with us on the basis of trust and we can't expose his/her feelings with anyone. Taking consent with parents is because what we are doing is a research and from research's perspective, we have to take consent from the parents if their children are below the age of 18. We take consent with parents in such case describe them what we will be doing but we don't open all the questions in front of them. If the adolescent have suicidal ideation and it is all because of their parents then we have to inform their parents about it. We also include these things in our consent form. Though we find out the fact we won't tell the parents that their children are depressed because of them, instead, we provide service for the adolescents and do things to cure their depression.

Reference 10 - 0.34% Coverage

P: We can't tell the parents that because of them their children are having depression, even from research's perspective, we can't say so. There could be some conflicts later on their life as well and it could even bring some other risk in future.

<Files\\KII\\ > - § 5 references coded [4.99% Coverage]

Reference 1 - 0.68% Coverage

I: Ok. And do you know about the risk that come along while using such risk calculator?

P: For this…

I: While using…

P: If a person do not have sufficient knowledge about it then the identification of depression is perceived negatively. So to minimize this, as we already said, developing skilled human resources and providing them capacity about the things we would want to do, then we can reduce the risk of such tools. We cannot say that there won't be any risks, but what we have to know is, how we are going to manage and minimize those risks.

Reference 2 - 0.48% Coverage

This is one kind of survey, public opinion. In future, for good cause, at government's policy level also we can discuss about use of this app and its process and how this can be incorporated in any kind of decision making at government's level. Talking further about obstacles, how can this be useful to illiterate one? This will not be useful in those areas where there is no electricity?

Reference 3 – 1.00% Coverage

As said previously, there is trend where we fill tool through verbal communication. The way person expresses himself through self-administration technique differs from the way he expresses to any person who asks him questions. Up to some extent it is different. The most effective way is through self-administration technique where we will be able to measure what we want to and it gives concrete output. But when mediator or someone else plays role of middle person, slight difference will be seen. A little difference will be there. That could be one challenge and this is what I feel. Let us try to observe from our side and see how this work in upcoming days and how this challenge can be minimized. We need to work on how we can make this tool be easily usable among both literate/ illiterate or rich/ poor.

Reference 4 – 1.41% Coverage

Agreed. You have also linked already. Instead of revealing the result directly, when one finishes filling the tool we can ask him about how he would like to predict the result based on his experience. We can follow step of neutralizing for cognitive restructuring in gradual manner before result is revealed and when he comes at acceptance level then we can reveal the result. This is for individual level. But as you have also stated previously, in community like ours people generally have negative attitudes towards mental health problems or mental illness. Therefore, I don't think this needs to be revealed to others. Like, if teachers know about this then he could say this person is like this and that. If parents/ guardians come to know then they will also start to say this and that. This also can happen in the community. Therefore, this should be done from individual level. In addition to what you said, in developed nations, this is quite different. The way they perceive about this issue is quite different from ours. But, therein also there is something like privacy of person. Herein also, I think we should maintain the privacy.

Reference 5 – 1.41% Coverage

P: Agreed. You have also linked already. Instead of revealing the result directly, when one finishes filling the tool we can ask him about how he would like to predict the result based on his experience. We can follow step of neutralizing for cognitive restructuring in gradual manner before result is revealed and when he comes at acceptance level then we can reveal the result. This is for individual level. But as you have also stated previously, in community like ours people generally have negative attitudes towards mental health problems or mental illness. Therefore, I don't think this needs to be revealed to others. Like, if teachers know about this then he could say this person is like this and that. If parents/ guardians come to know then they will also start to say this and that. This also can happen in the community. Therefore, this should be done from individual level. In addition to what you said, in developed nations, this is quite different. The way they perceive about this issue is quite different from ours. But, therein also there is something like privacy of person. Herein also, I think we should maintain the privacy.

<Files\\KII\\ > – § 6 references coded [7.69% Coverage]

Reference 1 – 0.46% Coverage

I: Though we talked about the personnel who would use the risk calculator if it is to be developed, are there any risk of using it?

P: If one does not know about the sign and symptoms in the first place then it would be misleading. But once the case is in the final referral place, it won't be that risky because we can identify it.

Reference 2 – 1.26% Coverage

I: So, it is better to take multiple responses for one adolescent. These are the things we talked about the benefit and effectiveness now let's talk about consequences. What will happen if we tell an adolescent that s/he is in high risk of depression? Though you have already said that it is just about risk and it doesn't say that there is the chance of depression, what could be the consequences if we tell them that they are at high risk of having depression in near future?

P: If we look at it positively, we can say that they will be more conscious; they could think about the risk factors and also think about the ways of minimizing them. And again, not everyone's perceptive is same. If someone is anxious then they might get scared after knowing that they are at high risk of depression. They might think about it every time, get scared and may stopped working. There are positive as well as negative parts.

Reference 3 – 0.53% Coverage

I: What can we do to minimize or reduce such challenge?

P: For reducing this, we have to give them clear statement saying that it is only about the risk factor assessment and it is not sure that you will have depression and also say them that if they want they can go to the experts for preventive measures.

I: So, while displaying result, we have to tell them such things?

P: yes.

Reference 4 – 4.31% Coverage

I2: As we said that it is just a prototype tool that is targeted for adolescents. So, would it be better if we display the result in front of adolescent after they have completed filling the form or should we keep the result confidential where only experts will know it and will consult about it with that adolescent's parents? What could be done for confidentiality? As you said, some may be sensitive and some may perceive such things in a positive way. What do you think could be done to make it in a better way?

P: What age group have you defined as adolescents here in your study?

I2: We have defined 10 to 24 years for adolescents in our study…

P: You have extended it up to 24 years (Laughing)?

I: It is for our project but…

I2: The age group for adolescents is usually up to 19 years.

P: Yes, some have defined it from 9 years, 11 or 12 years…

I2: So, what do you think what could be the defined age group for adolescents? For now, we have kept it as open ended question.

P: It must be understandable for the adolescents if we are giving them these self-administered questions. The way a 9 years old understands and 12 years old understands is different. So, I think, age group from 12 years is fine.

I2: So, it won't be a problem if we use self-administered questionnaires?

P: Yes. And like you said, about disseminating the result to them…it is their right to know about it so we can take consent with parents for displaying results to the individuals/adolescents. For adolescents till the age of 18, we have to take consents from parents for this. First of all, we get to know about it and then we let their parents know this. Then what we can do is, we can ask their parents' and let the adolescents know the result.

I: In our society, depression is still stigmatized. If we tell some of the adolescents that they are at high risk of depression, will there be any barriers because of stigma? Will their neighbours stigmatize them because of their risk of having depression in near future?

P: Disclosing about their children's risk of having depression is in the hands of their parents. And again, it is not sure that they are going to be depressed, it is just about risk. It helps in the prevention of depression. It is far better to give information rather than hiding it from them.

I: And do you think there could be negative consequences in the family because of stigma?

P: I don't think there could be any negative consequences. It is again about the person's personality and the way they perceive things. Parents may also be anxious and may get over involved in their children's life because of fear; they may not allow their children to go out because of fear. But for this, we have to provide them proper information.

I2: Because it could help in prevention, it is better to perceive this in a positive way, isn't it?

P: Yes, exactly. We should not hide any kind of information just because of stigma. We have to share the information as much as we can.

I: Yes, and it will help in the prevention of depression?

I2: So, there are more benefit rather than negative consequences.

I: Yes.

Reference 5 - 0.72% Coverage

I2: So, it won't be a problem if we use self-administered questionnaires?

P: Yes. And like you said, about disseminating the result to them…it is their right to know about it so we can take consent with parents for displaying results to the individuals/adolescents. For adolescents till the age of 18, we have to take consents from parents for this. First of all, we get

to know about it and then we let their parents know this. Then what we can do is, we can ask their parents' and let the adolescents know the result.

Reference 6 - 0.41% Coverage

P: Disclosing about their children's risk of having depression is in the hands of their parents. And again, it is not sure that they are going to be depressed, it is just about risk. It helps in the prevention of depression. It is far better to give information rather than hiding it from them.

<Files\\KII\\ > - § 11 references coded [11.36% Coverage]

Reference 1 - 2.07% Coverage

I: So as far as if, for within that, if we stuck with this idea of a broader generalized tool what about that, so that might still be, it sounds like they are here now. What do you think about using a tool even like that to predict in the future, do you think that it will be helpful to say if on an individual level an adolescent come and you say, ok based on your environment, and your resources XYG you may have a chance to get depression in your tool and do you think it might help adolescent do things like exercise more or wanna be better or do you think it will be more harmful? Do you think that it could make them feel worse or go home and may be their parents don't, oh! Now you gonna get depression so stay away from me kind of things. What do you think of something like that, something that predicts in the future?

P: Ok, thus is very ideal condition if it was there. If we have enough data and we have certain models that can predict depression that will be very good. So far I don't know, I am not sure about that. Are we there, there is always depression. We don't have evidence for that but still we can say these are the issues and having these issues might develop so let's prevent that can be helpful but this model should be used in caution because sometimes you are trying to pathologies normal life suffering and distress. Just like after earthquake, many newspaper started saying that ok now mental illness is going to rise up (laugh) and ok, PTSD is going to rise. We had to tell everyone even in our association. We set up some guidelines within a saying that ok normalize this fear, this is quite normal, this is normal fear in the time of abnormal situation. So, we have to, we can use this but with care.

Reference 2 - 0.87% Coverage

I: Are there any benefits beside these? What would happen if we look into the family level or the…

P: There is also a different thing. It could be extra trauma for the family. We have said that it is high risk but for parents, it might be very huge issue and they might think that their children are going to suffer a lot. There are both positive as well as negative consequences. We have to see the consequences for the adolescents once when they know that they are at high risk of depression. If I am an adolescent who always comes first or second in class and I am anxiety prone, what would

be my reaction after knowing that I am at high risk of depression? Not depression but my anxiety level could increase. So, we have to…

Reference 3 – 2.42% Coverage

I: Is there something in research ethics where it says we should reveal the result to the participant or something like that?

P: We could tell them to ask individually and know the result if they want. But we have to tell the generic message to all of them. We can reveal results immediately to those who have access to services near them. For example, if someone is in high risk and they have school counsellor in their school then we can tell them that they are at high risk of depression so that they could immediately go to school counsellor for counselling if needed but where people don't have access to such facilities and yet know the result, they might get scared and might have negative consequences in them. So, for them, we can provide some generic information about mentally healthy life style and preventive measures of depression instead of directly showing them their result of risk. Just imagine, if someone tell you that you are at a high risk of depression then what would be your reaction? How would you feel? You would obviously feel stressed (Laughs) but those who have access to services could be calmed down and might not be a victim of negative consequences. We might also get some cases of high risk of depression where we could look from the parent's or teacher's perceptive and know if they are at risk, because obviously very high risk of depression could be seen from other's view as well. And for the other general population, teachers or nurses or other person could use this tool up on them and know the result but don't reveal it to the adolescent and start the intervention where needed and instead provide generic feedback or message to every one so as to make them aware and prevent depression. Among 100 students at school, there might be 5 of the students who could be at risk but for those 5 we can't risk those 95 students' life because this is a very sensitive issue. We have to choose some way to do this so that no one could be at risk of negative consequences of using this tool.

Reference 4 – 1.26% Coverage

I: Suppose if I am a lay person, will I be able to use this risk prediction tool?

P: Yes, of course! After predicting the risk, the researcher should do according to their ethics. If 5% of the population has high risk of depression, then what should be done should be included in their protocol. There could be only 5% of adolescents who could be at high risk, or let's say 20% would be at high risk after we used this tool among them, but what about those 80% of adolescents who are at normal stressed but not at risk of depression? This is my only concern. For those with high risk as well, we have to tell them that it is normal so there is no any issue for you coming to seek help, we have to convince adolescents this way. There are many people who come to us after they use some self-diagnostic tool from internet and many of them are of anxiety disorder. Usually, students of MBBS and Nursing field feel that they have almost every problems after they study symptoms of each diseases from their course. Teachers of mental health also feels the same.

Reference 5 - 0.93% Coverage

I: Ok. While showing them the result, we can give them some recommendations?

P: Yes. Everyone should get a generic message to make them aware but specific ones who are at high risk should additionally request for their result even if they get that generic message. We are not sure that the prediction really mean that they are going to have depression sooner or later. Among two of the children of one parent, one might say that s/he has been changed in a good way just because of that one slap from his/her parent but the other might be having problem after that slap. This is philosophical question. So, when we ourselves are not confirmed, why to make people panic? This is only my concern. Or else, we can use this in some specific place but hope that people won't misuse it.

Reference 6 - 1.15% Coverage

I: Developing tool only is not enough, we have to use is as well, isn't it?

P: Yes, you can develop a tool but you have to clarify everything to the one who would be using it. One have to be clear about everything that is in the tool before using and such tool should be available in such place where you also have access to services. Sometimes people go and use screening tool among some population and tell them that "Oh my god, you have depression" but don't do anything further. And the other thing we Nepali have is, we are not habituated of reading instructions. So, if you put some precautions, it might work, not to make too much promises, alright (Laughs)?

I: Ok, sir!

P: The questions must be justified. As you have kept vulnerability factor, you have to justify why have you kept that? The one using that tool must have idea about that. Else, it is a good approach. Developing this kind of tool would be a good approach.

I: Ok, thank you so much sir.

Reference 7 - 1.16% Coverage

P: Yes, of course! After predicting the risk, the researcher should do according to their ethics. If 5% of the population has high risk of depression, then what should be done should be included in their protocol. There could be only 5% of adolescents who could be at high risk, or let's say 20% would be at high risk after we used this tool among them, but what about those 80% of adolescents who are at normal stressed but not at risk of depression? This is my only concern. For those with high risk as well, we have to tell them that it is normal so there is no any issue for you coming to seek help, we have to convince adolescents this way. There are many people who come to us after they use some self-diagnostic tool from internet and many of them are of anxiety disorder. Usually,

students of MBBS and Nursing field feel that they have almost every problems after they study symptoms of each diseases from their course. Teachers of mental health also feels the same.

Reference 8 - 0.30% Coverage

P: Those who have chosen to fill up this form are those who have been worried about their health or those who have heard about it from different media. So, if we make it that way where one has to request for knowing their result, it would be better.

Reference 9 - 0.17% Coverage

P: I am really critical about tools that's why I said so. There is a huge responsibility once you develop a tool else it is better if you don't.

Reference 10 - 0.70% Coverage

P: Yes, you can develop a tool but you have to clarify everything to the one who would be using it. One have to be clear about everything that is in the tool before using and such tool should be available in such place where you also have access to services. Sometimes people go and use screening tool among some population and tell them that "Oh my god, you have depression" but don't do anything further. And the other thing we Nepali have is, we are not habituated of reading instructions. So, if you put some precautions, it might work, not to make too much promises, alright (Laughs)?

Reference 11 - 0.30% Coverage

P: The questions must be justified. As you have kept vulnerability factor, you have to justify why have you kept that? The one using that tool must have idea about that. Else, it is a good approach. Developing this kind of tool would be a good approach.

<Files\\KII\\ > - § 20 references coded [17.60% Coverage]

Reference 1 - 1.77% Coverage

I: Is it easy for them to perceive these things easily? Or could it bring some negative thought on people because they are at high risk? And is it easy for the family members to perceive the thing that their children is at high risk of depression? How stigmatized would it be?

P: It is not about stigma. Once it is identified that the person is at high risk then we should try to convince them to take interventions. If not once you have to tell them twice thrice or even more. If you know that somebody is at high risk but because of stigma, you can't hide it from them. What would you do if that person commit suicide in the coming days? We must not identify the risk on the first place, but if we are doing so, we have to tell them about their risk with them.

I: So, if we develop such tool, would it be beneficial?

P: Yes!

I: To every level?

P: Sorry?

I: Where could we use this tool as adolescents are engaged in different settings; home school; community…

P: Where the adolescent could confidentially complete these set of questionnaire, we could use it there.

I: Is it?

P: Yes. It should be confidential.

Reference 2 – 1.47% Coverage

P: Rather than measuring likelihood of depression, why don't we just screen them for depression?

I: We have many tools for screening depression which are in use in the present scenario. But what this risk prediction tool does is, calculate the prediction of risk of having depression in certain period of time. So, it is a bit different.

I2: If we measure the likelihood of having depression in future, then we could use some preventive measures for that.

P: So, it is just a prediction tool?

I2: Yes!

P: And what does that future mean? Is it all over the life, next 6 months, next 2 months or next year? How do we predict this?

I: It will be clarified once it is developed. For now, it is just an example of such tool which could predict the risk of depression in few years.

P: I think its design is a bit problematic. If we find out that a person is in high risk of depression then do we also screen them for depression?

Reference 3 – 3.43% Coverage

I: If this kind of scale shows that a person is in low risk of depression..

P: But first the scale should be validated.

I: Yes, that process should be done in the making of this kind of tool.

P: Without doing so, you can't just say that if someone is in high or low risk of depression. You may be missing a high risk person who is in low risk. Or you are predicting someone of low risk as high risk. First of all, the scale should be systematically validated so that it is predicting what is actually should predict. Its internal validity should also be checked. Before generalizing, you should be sure because this is all assumption.

I: Instead of using the tools made for other countries, we are planning to develop tool for our own…

P: Which ever tool you use, first the tool should be tested and its correlation should be find out. If you are looking at the 10 year prediction, you have to predict the risk for now and follow u for next 10 years. And there comes about the budget for your follow up. The prediction tools are complicated, using screening tool you can compare and see if the person suspected to have depression actually have depression or not, but for prediction tool, you have to wait and see if your prediction really works after your follow up. Then you can say that your scale really works.

I: If we work according to that modality, this can work right?

P: There are lots of risk predictor tools and this kind of tools are really useful. But what I am saying is, you have to validate it from the field tests. For that, you have to do a longitudinal study.

I: Yes!

P: You have to conceptualize the thing that for how long are you doing the longitudinal study; is it 6 months, 1 year or 2 years or are you looking for the immediate prediction. That itself is a different format. You have to be predetermined about what you are really screening; is it the short term depression predictor or a long term predictor or you are also screening for suicide an if it is about 10 years of prediction then you have to follow up for 10 years.

I: Yes! Not only predicting the risk, we also have to see if its end result is accurate or not.

P: Yes.

Reference 4 - 0.88% Coverage

P: Also, I would like to say something more. Now, talking about the population, are you student focused, teachers focused or parents focused and there also comes about the confidential issues, positive response, choice of respondents meaning if I am at high risk of depression but I don't want to do anything for this, it's my choice so are we going to report parents about this or are we going to leave them for their choice. So, these things should be predetermined and develop the questions accordingly.

I: Okay sir, thank you so much for your time.

Reference 5 - 0.36% Coverage

I: Someone is there with them when they complete this set of questionnaire, so, what should be done? Is it better if we display the result to them or not?

P: We have to give them the choice by displaying the results to them.

Reference 6 - 0.24% Coverage

P: Where the adolescent could confidentially complete these set of questionnaire, we could use it there.

I: Is it?

P: Yes. It should be confidential.

Reference 7 - 0.63% Coverage

P: As I said, it depends on the purpose. Everyone could make a scale for something. But one must already define what they are targeting, why they are targeting and all of the other things. *Yetiikai balchi haneko hoina ni ta* (You are not going without a plan). While preparing for a research, you already should have one research question and go accordingly. It's like, I will go to do something then…

Reference 8 - 0.88% Coverage

P: In research, you can't just go randomly and see what you would get. If this happens, then you are going to get a wrong answers. If you think you are giving this to everyone and expecting the answer, there will obviously be something from someone every time. In research, you must have clear questions, for what you are making it, what is our aim and go accordingly. If you ask with everyone then it is obvious that there will be something for sure. But those answers usually cause errors. If you go like this, you will obviously find something positive.

Reference 9 - 0.59% Coverage

P: That's why you must have a clear question about what you are doing, why you are doing and for what purpose, while conceptualizing your research. You must have a clear question but instead, your question is too vague. If you ask us about who could use it, who could be targeted or something like that, there will obviously be some information and it usually becomes wrong.

Reference 10 - 1.58% Coverage

P: There is no any question about the benefits for adolescents with depression. But you should at least be clear about the questions like are you going to target adolescents for this or are you going to target researchers for screening adolescents, are you going to target teachers for screening adolescents, are you going to target parents to screen adolescents, in what settings you

are going to use it, are you going to be anonyms or not, are you going to tell the result in front of the individual or not and if you get positive results then are you going to take consent from the parents about it or not? So, these things…what you are doing is, you are putting all these things alongside and thinking to go accordingly with whatever comes on the way or from the answers of respondents. These are the things that should be pre-determined and according to that you should scale up and proceed. Do you understand what I mean?

I: Yes sir!

P: Your approach and my approach is completely opposite.

Reference 11 - 0.41% Coverage

P: I have to be aware about the research validity and other related things so that I could think about what could be included further. Without knowing all these and just seeing at the questionnaire, I would only be able to say that everything is good for now.

Reference 12 - 1.22% Coverage

P: Again, it depends on the purpose of the study you are doing. Are you doing this for research purpose, are you doing this for screening purpose or are you doing this for treatment purpose, these should be predefined before developing the any scales. But whatever the purpose is, if we find someone with high risk of depression, we need to find some way to recommend them to treatment. If we screen and find 50 people having depression, where would we send them for treatment? We don't have any place for the treatment. Are we going to screen people with depression and then tell them that you don't have any place to go for treatment? This is also a question. So generally, if we find that somebody have a high risk of depression then we need to find some ways to help.

Reference 13 - 0.43% Coverage

P: What I would like to say is, you must first know how accurate is this kind of tool if it says that someone is in high risk of having depression? If someone is predicted to be in low risk of depression then that doesn't mean s/he is really at low risk of depression.

Reference 14 - 0.07% Coverage

P: But first the scale should be validated.

Reference 15 - 0.70% Coverage

P: Without doing so, you can't just say that if someone is in high or low risk of depression. You may be missing a high risk person who is in low risk. Or you are predicting someone of low risk as high risk. First of all, the scale should be systematically validated so that it is predicting what

is actually should predict. Its internal validity should also be checked. Before generalizing, you should be sure because this is all assumption.

Reference 16 – 0.91% Coverage

P: Which ever tool you use, first the tool should be tested and its correlation should be find out. If you are looking at the 10 year prediction, you have to predict the risk for now and follow u for next 10 years. And there comes about the budget for your follow up. The prediction tools are complicated, using screening tool you can compare and see if the person suspected to have depression actually have depression or not, but for prediction tool, you have to wait and see if your prediction really works after your follow up. Then you can say that your scale really works.

Reference 17 – 0.32% Coverage

P: There are lots of risk predictor tools and this kind of tools are really useful. But what I am saying is, you have to validate it from the field tests. For that, you have to do a longitudinal study.

Reference 18 – 0.74% Coverage

P: You have to conceptualize the thing that for how long are you doing the longitudinal study; is it 6 months, 1 year or 2 years or are you looking for the immediate prediction. That itself is a different format. You have to be predetermined about what you are really screening; is it the short term depression predictor or a long term predictor or you are also screening for suicide an if it is about 10 years of prediction then you have to follow up for 10 years.

Reference 19 – 0.16% Coverage

I: Yes! Not only predicting the risk, we also have to see if its end result is accurate or not.

P: Yes.

Reference 20 – 0.80% Coverage

P: Also, I would like to say something more. Now, talking about the population, are you student focused, teachers focused or parents focused and there also comes about the confidential issues, positive response, choice of respondents meaning if I am at high risk of depression but I don’t want to do anything for this, it’s my choice so are we going to report parents about this or are we going to leave them for their choice. So, these things should be predetermined and develop the questions accordingly.

<Files\\KII\\ > – § 2 references coded [3.75% Coverage]

Reference 1 – 1.97% Coverage

: Yes. And what do you feel are its benefits? What could be the potential benefit?

P: One would be the confidentiality. The next would be regarding the self- awareness. Umm… and the high risk… You said that it calculates the risk of depression on its own, didn't you? So, they will be able to know where they are after filling it up on their own. We have information with us in case they seek for help. That is why it will be confidential as well. Umm… next, they will be able to take this if they have the internet access.

I: So you are saying that the questions need to be changed and anyone could use it so that it could be predicted. Do you feel that there are any challenges when using this tool?

P: I have mentioned the issue of language already. And then the other challenges… Thinking about it right now… One thing is that they need to have access to (smart) phones. In order to for it to function well, they need such phones. Only the people who have access to it can use it. There is the chance that it might not be as effective at larger areas sometimes where the risks are greater.

I: Do you mean the entire area?

P: there could be some depression- prone areas for the adolescents. It could also be in some emergency situations because of natural disasters. In such context, if it is not in access to everyone, then others tools could be effective rather than this.

Reference 2 – 1.78% Coverage

I: Right now, the results of the adolescents are revealed after they enter their answers. There are things like stigma that we need to consider when we reveal the results right there at the moment. They might learn that they are at high risk. What do you think is the possibility that this tool will play a negative role rather than a positive role?

P: That is possible. Two close friends might find that one is at high risk whereas the other is at low risk. But there has to be some awareness before this is carried out. There could be sensitization programs that are carried out where all these things are explained combining the issues of stigma also. We need to let people know that this is for our own well- being. They do not need to tell their friends about it if they do not want to. They should not pressure their friends when asking them about it. We need to be sensitive about these things. Such content should be there at those particular

areas. Maybe those things need to be there. Then, they will understand its objective. They will learn how to use it. We will tell them about the process. They need to be told how we will move forward with them after that. If it could be done, then maybe it will be easier to mitigate those challenges.

<Files\\KII\\ > - § 2 references coded [3.80% Coverage]

Reference 1 - 1.78% Coverage

I: So, are there any challenges while filling up the tool even if we implement self-administration technique as you mentioned earlier regarding the limited manpower and time limitation. If so, how can we overcome this?

P: I have said earlier about the manpower and time constraints. As you said about self-administration technique, constraint coming on that would be, ummmm, while filling up the form alone by an individual he/she would have difficulties in understanding few questions. In order to have a proper guidance while filling out the form, a reliable person, let's not say a guardian because guardian may also be influential but totally a neutral person with whom the individual may feel comfortable and confidentiality is ensured, if we find such person, it would be best but that might not be possible always. So, that may remain as one constraint. In the absence of proper guidance, he/she will go on filling up the form and might end up randomly ticking the answers, so the results might be deviated.

Reference 2 - 2.03% Coverage

I: Self administration technique will be effective for literate individual and they can do it by themselves but for illiterate and drop out adolescent, what could be done?

P: Ok. So, now you have raised new problem. (Smile) Initially, we plan to go to school but illiterate individuals won't be found in school. So, it would be quite difficult to approach them. Additionally, next reason for them for not going to school would be because of certain background. As a result of which they wouldn’t be going to the school. This can also be the cause of mental illness, to be truthful. Therefore, to reach them certain community programs should be launched in partnership with Nepal government. Only then, it is possible. FCHVs are present at grassroots level. So, in association with FCHVs such targeted population can be reached. Literate individual can be

reached through school. Through FCHVs, illiterate population can be reached. Informant would be an adolescent themselves but for filling up the forms, adolescent being illiterate we ourselves have to provide the manpower for that. Sometimes, FCHVs are also illiterate. So, we need to mobilize ourselves.

<Files\\KII\\ > - § 2 references coded [0.94% Coverage]

Reference 1 - 0.60% Coverage

I: Do you foresee any challenges for using such tools? If so, how might we address them?

P: We cannot assure that we won't have problem while doing any work. We wish not to face problems. Those who work face problems not others.

Reference 2 - 0.35% Coverage

I: What types of problems might arise while using this tool?

P: I don't think that huge problems will come but I don't know exactly.

<Files\\KII\\ > - § 2 references coded [0.87% Coverage]

Reference 1 - 0.24% Coverage

Along with this, if cultural components is involved in this risk calculator it will be better.

Reference 2 - 0.63% Coverage

Therefore, after the family is aware, the family members will also look after those who are at high risk or I think it will be better either if we can use them under direct supervision of someone or conduct in the form paper-pencil tests and so on.

<Files\\KII\\ > - § 3 references coded [2.78% Coverage]

Reference 1 - 1.08% Coverage

P: So we cannot just decide based on—because even if when we are using these screening tools, we don't rely only on the screening tools. We coordinate it with our own observations. So, I think you have to coordinate with clinical signs and symptoms, that they are clinically correlated.

Reference 2 - 1.37% Coverage

I: Would you say the same for high risk as well?

P: Yes. High-risk you have to be more careful, but we can't just leave out the low-risk.

I: Yep (laughs). Defintely.

P: Because it develops over years. Later on even if you were not depressed, your childhood was very nice—you can have depression when you're in your 20s. So you cannot be so sure about that!

Reference 3 - 0.33% Coverage

I: Ok. So you think there should be some sort of structure to it?

P: Yes, yes exactly.

<Files\\KII\\ > - § 1 reference coded [0.38% Coverage]

Reference 1 - 0.38% Coverage

P: Well, if it's "high risk," then definitely. I mean, I don't know, maybe it depends. We might need to take an assent from the adolescent. Like, when we start asking them questions, we let them know at the beginning that it's confidential and all, but if we believe that there's a risk to your safety or health and it has to be disclosed, then it might be.

<Files\\KII\\ > - § 5 references coded [3.90% Coverage]

Reference 1 - 0.93% Coverage

P: We have to consider how much openness they show when answering it. The challenges would be the number of things that they would hide. These are the challenges that we will face with them. We will have the questionnaire… How honest will they be when answering them? I think that honesty is one of the main challenges. They will not tell us a number of things even though they have such experiences. There is the concern of privacy and honesty that is raised there. I think that these are the challenges.

Reference 2 - 0.75% Coverage

P: Nowadays, the adolescents are such that they are very conscious about their physical appearance rather than their health. They think that others will come to know of the things that they do and they will feel that they are undervalued. These kinds of issues come up. So, there is the issue of reliability. I think that it is a challenge to get reliable answers from them that are close to their reality.

Reference 3 – 1.04% Coverage

P: The main thing is that we need to take their consent first of all. We need to explain all of the things to them. Then, we should avoid storing their names. After that, I feel we do not need to collect information regarding their age if we are going forward with the general assumption that they are adolescents. I think that it can be achieved by not collecting their names and ages. I think that there will be openness if we do not hold their names and ages because there will be no specific identification about whose information we are actually collecting.

Reference 4 – 1.05% Coverage

P: But how reliable would it be if we were to do this? I do not think that it would work if we did not tell the children but only their parents were informed about it. We need to tell both of them. If only the parents are told about it, then how will they handle their children is important. It is not necessary that all the parents are able to understand this. We all know that there are very less parents in Nepal who are at the standard of understanding these things. That is why we need to inform the parents as well the students. It should be done for both of them.

Reference 5 – 0.13% Coverage

P: Both the parents and the children need to be told about these risks.

<Files\\KII\\ > – § 2 references coded [2.03% Coverage]

Reference 1 – 1.06% Coverage

P: Yes, that is one thing. But is the adolescent ready or not? There is the aspect of whether they want to share it with others or not. So instead of giving them the results immediately after they have completed the tool, they will need a supportive figure like you mentioned when disclosing their results. They could be told that it would be easier for them to understand it if they were with someone there. That could also be done. But I think that the issue will still be the same. They might feel that they have ticked those options on their own like game, but the need to show their results to the guardians might limit them. So we need to consider that its impression could be at multiple levels.

Reference 2 – 0.97% Coverage

Even when we talk about the high risk and the low risk, we could use milder terms instead. What are we looking for when we say high risk? We are expecting that they need to go to the counselor now or go meet (psychosocial counselor) somewhere. According to their results, they need to sit down with their guardians and talk to them. It means that their results have to be disclosed in a minor form first. And then we should disclose the major implications when their guardian is with them. They (the patients) will also feel that they need to tell their guardians now. They will feel that it is a decision that they have taken themselves.

<Files\\KII\\ > – § 3 references coded [3.39% Coverage]

Reference 1 – 0.79% Coverage

P: Reliability should be checked. One from filed by the adolescents and other filled by their parents should be checked for the consistency. If reliable, then whatever has been filled shall be accepted. Some people because of biasness, children might have filled randomly. We can figure out by this.

Reference 2 – 1.10% Coverage

P: I told you some adolescent can understand and some cannot. You also take as above 18 and below 18. About adolescent below 18, information should be shared with parents. If above 18, then it is better to let them know by themselves as we assume them as legally matured. But if he is an adolescent of 14- 15 years, he might not have concept. So if he is in high risks, then letting the nearest legal guardian is better.

Reference 3 – 1.50% Coverage

P: If you make them understand before, there could be biasness. If he understood that he has depression and ticks accordingly, then score might get influenced. Some might know about depression, no action is required in this situation. However if somebody does not know then the importance of scoring should be explained to him. If left, there might be confusion. Moreover, some have the nature of complicating simple things. He might be thinking that he has been

suffering from big disease. So it is better to explain him the result and recommends him the place to visit.

<Files\\KII\\ > - § 5 references coded [8.57% Coverage]

Reference 1 - 2.15% Coverage

P: It is about where we validate. If we are asking questions in *Maithali*, then we should take the tools that validate the *Maithali* culture. If we are doing it in *Kathmandu*, then based on *Kathmandu* is fine. If you take *Kathmandu* based tools to *Jumla*, will that work? [Rhetorical question] But what we do is we take same tools throughout the country that has been validated in one place. That is the problem. The tools should be validated in the same place (where it is to be used).

Reference 2 - 1.35% Coverage

P: The tools validated in *Jhapa* do not work for *Morang*. Moreover, which community is there in *Jhapa*? Our problem has been that. Will the tools validated in *Chitwan* work for health facilities in *Jumla*? Until now, we have been doing the same. The right measurement has not been possible because of this.

Reference 3 - 1.94% Coverage

P: Making the tools is not the major thing, what does the tool measure is the important thing. Making tools and throwing it in the market has no meaning. If the tool is used by others and measures in the right way, then that will work much more, if not then has no significance. So first, that should be a quality tool. If there will be tools, then that will work. If the tools can be made as I explained, that would definitely work.

Reference 4 - 2.35% Coverage

P: They can conceive negatively. If the reason is not a family, then why should talk within the family. We will find out the reason behind. That might be because of peer. If related to school, then it can be discussed in school. If family is the reason, then we can convince saying these things are out, so be careful. If you tell them for instance, this is because of his personal reasons. He has

relation with this kind of girl which is why these things are happening, what if they hide their son away from their own home?

Reference 5 - 0.80% Coverage

P: As I told you earlier as well, that (tool) cannot be used. Using it is wrong. Tools must be different for adolescents. Everything is different in adolescents, what is similar?

<Files\\KII\\ > - § 2 references coded [1.41% Coverage]

Reference 1 - 0.61% Coverage

P: But then the other aspect is if s/he would have a problem knowing s/he has risk. There could be a negative impact knowing s/he has a risk of depression in the future.

Reference 2 - 0.80% Coverage

P: Result should be told but rather than saying that s/he is at high risk, in a positive way we should say "don't get panic, for now, you are seen in high risk but you can take help". There should be a pop-up in this way.

<Files\\KII\\ > - § 4 references coded [4.45% Coverage]

Reference 1 - 1.31% Coverage

P: So not as a researcher but me as an adolescent, if I get the opportunity of using this tool I'm thinking what would be on my mind. The first thing that I would stand out is, if I reached the high risk my question would be "what now?" So there is a range of age for adolescent. In that sense, I think different kind of intervention is needed. And there will be a difference between a 12 year old using this tool and a 24 year old using this tool. So, what have you thought about the age?

Reference 2 - 0.98% Coverage

P: So, in that sense, the risk seen in a 14 year old and a 25 year old are taken to the same link like you said or as much as I've understood. You said that guidance is provided through the same link. My question is how much they receive the guidance because there is a big difference between a 14 year old and a 25 year old.

P: Nowadays, it's said from 10 years old

Reference 3 - 1.84% Coverage

P: So there will be more access because everyone has their smartphone in their hands which is good and they will find out easily by staying home. But its downside is that if we have bandwidth or not because there are very limited mental experts, counsellors and psychiatrists in Nepal, Kathmandu. So if this app is used in other places than Kathmandu that access will be lesser. For example I figured out that I'm in high risk and I know where to go but there's no access in my village and there are no expertise to go to. And my society stigmatizes mental illness. That means we helped to find out the problem but solution will not be enough for everyone. And that's what I find risky.

Reference 4 - 0.32% Coverage

I: Anything else?

P: For now, that's what came immediate in my mind. But if we can do this it will be very interesting.

<Files\\KII\\ > - § 3 references coded [1.95% Coverage]

Reference 1 - 0.30% Coverage

P: The other challenge if to remember...

[Audible external noise]

P: TPO is research-based. If it is done, at first most probably piloting is done.

Reference 2 - 0.49% Coverage

P: When it is taken a bit ahead, the process evaluation, how the outcome is, how the impact is, how they have been from services...how effective has it been, how have been used and how effective it has been because it has not been started yet.

Reference 3 - 1.15% Coverage

P: After it is started, something comes out. For that, continuous monitoring and research about it should be continued time and again. Most of the time, the program is done but the program is effective or not. For that, collaboration is needed. After all is done, researcher... Mental health is one public health issues of Nepal. Being it a public health issue, public health researcher, researcher related to mental health, program implementation, research, practice, all should be involved in its program evaluation as well. For service, one team would be sufficient.

<Files\\KII\\ > - § 2 references coded [0.92% Coverage]

Reference 1 - 0.17% Coverage

P: At first, that should be validated, how sensitive is it.

Reference 2 - 0.76% Coverage

P: Definitely, sensitivity accessibility should be there… But the tool which we would be endorsing, that should be well accepted. If that is the screening tool, the sensitivity should be high… So if it fulfills the minimum criteria of tools, there won't be the difficulty.

<Files\\KII\\ > - § 5 references coded [4.97% Coverage]

Reference 1 - 1.09% Coverage

I: If this tool would be validated in our cultural context, can any challenges or barriers come while using in the adolescents' age-group?

P: There won't be any questions of coming. This is good but the government, ministry of health should approve it.

Reference 2 - 1.43% Coverage

I: To incorporate it in the education sector or in other sector from policy level…

P: After you do trial and comes to the ministry of health following the procedures, ministry of health would also consult with psychiatrist or do trial on own. After the approval is made on that basis, that would be the property of the state/country.

Reference 3 – 0.24% Coverage

P: We can use it freely and would be validated as well.

Reference 4 – 0.63% Coverage

P: The tool would be validated from the policy level and do as per you said earlier. What other things can be done to effectively execute this tool?

Reference 5 – 1.57% Coverage

P: We need to see this as a trial first. It won't be final. We can change with the feedback received after the application/execution. It is better that we do not be much ambitious and go simple in the initial phase. Executing it for 1/2 years and depending upon the feedback, it can always be modified. It won't remain same always, will change in accordance to time.

<Files\\KII\\ > – § 1 reference coded [1.78% Coverage]

Reference 1 – 1.78% Coverage

I: If this tool is owned by the policy level or government level, there will be a mass coverage which is very good. So, what should we do to make that happen?

P: First, we should generate the event, we should mention the things that are in our context, and we can't only talk about the foreign context. After the data is available in our context we should analyse it and we should experiment that. Only after that we can make it happen.

<Files\\KII\\ > – § 1 reference coded [4.20% Coverage]

Reference 1 – 4.20% Coverage

P: In case of your study, you made this and you may give it and bring it back and people may communicate with you saying that they are in risk. But some people will not believe you. I think this should be done only by the study. Model should be made to those who can help to assist like; teachers, friends or friend circle because people believe friends. Either that or health workers. Parents might not be cheered so much. There might be a tool for parents and maybe they can understand. You should see who will be the appropriate person to share and collect the information.

Instead of only model you should see different models from school teachers, researchers, friends and family or health workers. Health workers say don't do these works. They only do it after it comes. Where are the places that adolescents go? One is school another is gym, clubs. Those places are important. Before joining the clubs I think it will be better to talk about their mental health status. This should be identified by the study. (Paused)

<Files\\KII\\ > - § 1 reference coded [0.52% Coverage]

Reference 1 - 0.52% Coverage

P: It should be worked out on detailed way. You need to consider the topics which are sensitive, by doing a pilot study may be!

<Files\\KII\\ > - § 5 references coded [3.31% Coverage]

Reference 1 - 0.89% Coverage

P: Yes. It is something that is important to every people in society. It is not ethical to laugh when we find other people suffering from the problem. Anyone can be suffering from the problem anytime. Therefore, not only at individual level but at social level on daily basis it could be of help. There might be other whose child could be handicapped as well. Everyone should be aware of this. This is social support. Everyone should learn on how to deal with them. Problems could be minimized on daily basis and one could tackle problem easily. This is very good and is beneficial to everyone. There is no doubt.

Reference 2 - 0.69% Coverage

P: I understand that this will be given to someone who has no any problem in present. But still, this terminology is different. Herein, he might not be aware of things you are trying to seek from him. Yes or No? He might be able to explain normally about what is going on his life but might fail to explain in relation to terminologies. While filling herein we should focus on terminologies. Because of this, I think, as far as possible, if we fill form after discussion then I think we will get closer to fact.

Reference 3 - 0.74% Coverage

P: It is all about what we consider challenging. Is it to beat others? This is not the one I guess. Our main challenge is whether we succeed in bringing appropriate information from respondent. The challenge would be like to what extent will we be able to probe. It won't be in case of threatening. Convincing and adding details on terms and regulations before filling out this form will be of help. It makes difference on how we approach while filling form. Is it asking someone directly to fill the form or is it in motivating way by explaining all.

Reference 4 - 0.65% Coverage

P: One should be in personal touch and as far as possible should try to probe more for depth understanding. This tool is nice of its kind and will give some base. But my request is not to rely on this 100%. Because, this is merely mathematics kind. But human intellectual is something that we feel. Herein, we cannot write how we feel when we look at each other. On the base of this only, will we become able to make decision for someone's life? I don't think that is possible.

Reference 5 - 0.34% Coverage

I: So, you believe that someone has to be there in whose presence it can be filled out?

P: Yes. It won't be acceptable to me if you are trying to know psychological status by asking the sufferer to fill questionnaire alone. You understood right? (laugh)

<Files\\KII\\ > - § 5 references coded [2.08% Coverage]

Reference 1 - 0.76% Coverage

I: So, is it best practice to let them fill up by themselves?

P: Yes, they can share their views (words). In my view, other than positive and negative aspects, rather than them knowing on their status, if we know then it won't have bad impact.

I: Can you elaborate on that?

P: Like, if they know about high-risk status, those who can withstand will withstand themselves and know what to avoid but those who cannot might think, *"I am already in such status"* and will be more depressed and degrade further.

Reference 2 – 0.36% Coverage

I: So because of this you think that they can fill up this by themselves but the results should be given to their guardian (parents?)?
P: yes, they should not know the result directly. If their family members know, then that is much better.

Reference 3 – 0.08% Coverage

I: If done that way then it will be easier.
P: Yes.

Reference 4 – 0.66% Coverage

P: ummm, even if this is shared online, the risk should not be mentioned and while filling if they found something then they should be instructed to reach out to their guardian and guardian will react that way. If it is done this way, it would be easy. *"If someone has these symptoms then discuss with your guardian and guardian should be able reach out with the given contact details for high risk",* this would be easy if done this way.

Reference 5 – 0.21% Coverage

P: Yes. If talked only with guardians their child might not know that they are suffering from the problem. This way, they will be scared less.

1. Code Query Output

<Files\\FGD\\ > – § 4 references coded [1.69% Coverage]

Reference 1 – 0.18% Coverage

P7: There should be some key points like at last if they come to know that they are at high risk then there should be some key points about self-help techniques.

Reference 2 – 1.25% Coverage

P3: The organization can go to some school and take a class to orient students about the tool. And if we have targeted the class already, then I don't think we should be writing the age group again here, because we almost know their age. We should not keep any identifying information of them. If we do so, then we can get some kind of right information. If we tell them that no any identifying information of them are there and we are just doing a survey, then I think it will be a good idea. If they don't have to give any of their information which would help them to be identified, then they will think properly and fill up the form. We can choose to play some game with them to make it more interesting. And at last of the game, we can introduce the tool in front of them. If we directly introduce the tool then I don't think it will be better, so we can choose some other alternative like taking some other programs and at last introducing the tool to them.

P6: For that as well, coding and decoding will be needed…

P4: Yes, such information here are necessary, I was also about to say the same! (Supporting P6)

Reference 3 - 0.19% Coverage

P3: If we go according to the way that I have discussed just now, then the ethnicity would be a good idea but then we will be already familiar with the age group, isn't it?

Reference 4 - 0.06% Coverage

P6: Yes, there was even a section to mention their name!

<Files\\KII\\ > - § 10 references coded [4.85% Coverage]

Reference 1 - 0.36% Coverage

P: You have included most of the questions and I think it will cover what you are going to find out. There are few things that should be modified such as the question pattern and the sequence or about adding few more questions to cover risk. Otherwise, it is fine.

Reference 2 - 0.50% Coverage

For now, as per the question, adolescents are the one who would be benefited. But, of we make few changes in language, it would be fitted for everyone. The factors that trigger depression varies according to the age so if we make few changes as per required, this tool would benefit all other people. But for now, I see it more relevant and focused for adolescents.

Reference 3 - 0.36% Coverage

P: You have included most of the questions and I think it will cover what you are going to find out. There are few things that should be modified such as the question pattern and the sequence or about adding few more questions to cover risk. Otherwise, it is fine.

Reference 4 - 0.21% Coverage

The factors that trigger depression varies according to the age so if we make few changes as per required, this tool would benefit all other people.

Reference 5 - 0.44% Coverage

You have mentioned about the things that could be done for prevention so may be adolescents will take it positively or maybe they could not! But if we mention that depression is not that major problem and it could be prevented and cured if we follow some of the measures then may be adolescents could be calmed down.

Reference 6 - 0.36% Coverage

P: We have to mention such information for those with high risk. If one suddenly finds out that s/he is in high risk of depression then they might get shocked, so, to normalize the situation, we have to put such kind of information for those with high risk.

Reference 7 - 0.53% Coverage

I: That means, we can display the result in front of adolescents?

P: Yes, of course. Otherwise they will not feel the importance of filling the form. So, in the result section, there should be such information that could calm down adolescents with high risk of depression. It could benefit them. If we are doing this right now, there could be at least a researcher by their side…

Reference 8 - 0.68% Coverage

P: I mean, there could be a researcher who could give this form to the adolescents and ask them to fill up the form and when the result will pop out, then that researcher could normalize the situation. But if the adolescent is alone and filling out the form, there is equal chance of them reacting positive as well as negative after seeing the result. There could be risk or it could benefit them. For minimizing the risk, we could add some more information in the high risk section of result.

Reference 9 - 0.54% Coverage

P: Result is written in two paragraphs there where the result of their risk is written in the first one and the things s/he could do further is written in the 2nd one. So now, what we can do is, keep the first paragraph as it is and add things to calm down and normal the adolescent in the a different paragraph as keep it below the result and at last keep the third paragraph of it as it is.

Reference 10 – 0.87% Coverage

P: We could write something like this; depression could happen to anyone and it is most prevalent in this age group (Referring to adolescents) or some other age group whichever is the fact, depression could be cured. So if we write something like this then it could help the person normalize the situation. Seeing the result of high risk of depression suddenly, the person might get more curious and after reading such kind of information then s/he could be calmed down and the situation will be normal. Talking about the result of low risk, the information you have put is enough, there won't be anything that could be added.

<Files\\KII\\ > – § 18 references coded [9.13% Coverage]

Reference 1 – 0.23% Coverage

Previously we talked about including information related to family and social experiences but can we add about sleep pattern, weight, and dietary intake habit? Is it possible to add or not?

Reference 2 – 1.48% Coverage

Those factors can trigger as well. Sign and symptoms could be on one hand. Few people might experience sudden change in sleep pattern whereas in others reduction in sleep cycle or excessive sleeping habit could be developed and this could be part of the risk. Did you understand or not what I mean to say? (Pause) Instead of keeping that factor under sign and symptoms only, it can also be kept under triggering factor or vulnerability factors.

I: Please explain.

P: reduction in sleep cycle or excessive sleeping pattern observed.

I: The main causes for that are (interrupt).

P: Because of biological reason can't there be any changes in sleep!! Sleep itself is a part of biological factor.

I: So, you think biological factors related content needs to be added?

P: Few questions related to biological factors could be about eating habits, sudden decline in food intake or vice versa, sexual desires (lack of interest). As the tool will be used for adult as well, sex related question also needs to be added like level of interest (previously not having any kind of desire/ interest but suddenly stopping to have those kind of interest. I think these kind of information's are missed out.

Reference 3 - 0.18% Coverage

What I feel is, it has almost incorporated all the important questions but it has missed out biological portion which I think needs to be added.

Reference 4 - 0.83% Coverage

Haven't we included moderate level of risk?

I: We haven't included that so far. What are your thoughts on this?

P: Generally, while categorizing depression, it comes under mild, moderate and severe. We have been disaggregating depression under mild, moderate and severe. When we analyze the severity of any problems, they comes generally under three types. One is mild where the person is suffered but the effect is too minimal. When we fall under moderate category, there are moderate effect seen everywhere. Talking about severe, there is negative effect seen everywhere. Therefore, what I personally think is- we can keep three category like high, moderate and low.

Reference 5 - 0.26% Coverage

Before developing this kind of tool, we need to refer to other's work on how similar kind of tool has been developed. We need to look at their work as well. Generally we can find low, moderate and high categories.

Reference 6 - 0.37% Coverage

it's very good. As I stated previously, if we add one or two component's on biological stuffs then it will be good. In Nepalese community, this tool be helpful in understanding about child and adolescent who are at risk of developing depression. This way, I believe the good work will be going on.

Reference 7 - 0.55% Coverage

P: Those factors can trigger as well. Sign and symptoms could be on one hand. Few people might experience sudden change in sleep pattern whereas in others reduction in sleep cycle or excessive sleeping habit could be developed and this could be part of the risk. Did you understand or not what I mean to say? (Pause) Instead of keeping that factor under sign and symptoms only, it can also be kept under triggering factor or vulnerability factors.

Reference 8 - 0.76% Coverage

P: Because of biological reason can't there be any changes in sleep!! Sleep itself is a part of biological factor.

I: So, you think biological factors related content needs to be added?

P: Few questions related to biological factors could be about eating habits, sudden decline in food intake or vice versa, sexual desires (lack of interest). As the tool will be used for adult as well, sex related question also needs to be added like level of interest (previously not having any kind of desire/ interest but suddenly stopping to have those kind of interest. I think these kind of information's are missed out.

Reference 9 - 0.18% Coverage

P: What I feel is, it has almost incorporated all the important questions but it has missed out biological portion which I think needs to be added.

Reference 10 - 0.69% Coverage

P: Generally, while categorizing depression, it comes under mild, moderate and severe. We have been disaggregating depression under mild, moderate and severe. When we analyze the severity of any problems, they comes generally under three types. One is mild where the person is suffered but the effect is too minimal. When we fall under moderate category, there are moderate effect seen everywhere. Talking about severe, there is negative effect seen everywhere. Therefore, what I personally think is- we can keep three category like high, moderate and low.

Reference 11 - 0.27% Coverage

P: Before developing this kind of tool, we need to refer to other's work on how similar kind of tool has been developed. We need to look at their work as well. Generally we can find low, moderate and high categories.

Reference 12 - 0.67% Coverage

P: In this, we have mentioned about prevention. What I think is, there needs to be information added on what person can do at his personal level for the prevention of symptoms of depression. Therefore, if information's are mentioned under different categories like what can be done at personal level, what can be done at family level and what can be done at community level, then I think that will be better, from my own preference. It says about symptoms (umm), symptoms of depression, prevention, treatment (respondent is reading information)

Reference 13 - 0.47% Coverage

P: Yeah I have understood this. And, I think this is important for all three categories: low, moderate and high. Under many circumstances, when person undergoes treatment then there are many approaches where person can help himself like psycho counselling, psychotherapy, family therapy. These things can be added in formation box as well. I think other information's included are ok.

Reference 14 - 0.34% Coverage

P: (reading information box) Sound sleep, balanced diet and additional information on behavior. (ummm) Sound sleep, balanced diet, behavioral information (pause). I think we need to add few information on different techniques through which positive thoughts can be generated.

Reference 15 - 0.43% Coverage

P: Not only in low but also in high. Both categories. Generally, among those suffered from depression, we encounter pattern of negative thoughts. These negative thoughts results in negative attitude. So, it will be helpful if we mention about different techniques that help to minimize negative thoughts or divert one's thoughts from negativity.

Reference 16 - 0.75% Coverage

P: From my point of view, for the sake of information purpose I think anyone can benefit from this but I prefer this tool be filled by person suffering or likely to suffer from depression. And, I like the way you have created separate information box which contain information on help seeking and redirecting to different useful links for further tips. In redirect pages, we can find useful information that has relevant information on symptoms of depression, treatment modalities, medication, counseling, psychotherapy, role of family members which will definitely help people associated with sufferer as well.

Reference 17 - 0.31% Coverage

P: What we can do here is, we can mention in the last that if person is feeling very difficult then for treatment or additional services he can seek services from following referral centers located in each province or other. This way it could be easier.

Reference 18 - 0.37% Coverage

P: it's very good. As I stated previously, if we add one or two component's on biological stuffs then it will be good. In Nepalese community, this tool be helpful in understanding about child and adolescent who are at risk of developing depression. This way, I believe the good work will be going on.

<Files\\KII\\ > - § 14 references coded [9.20% Coverage]

Reference 1 - 3.17% Coverage

P: Ok. And in that question, you could put *Dam haru* along with *Chinnaharu* (Both Nepali words used to indicate physical mark).

I: Sure.

P: There is also the question asking adolescent that if or not they have left home and ran away somewhere… so, there is a question about the scarcity of food along with the part of clothes, there comes two question alongside but the focus seems in the part of clothes only…

I: So, it is better if we keep both of them as an individual question?

P: Yes but if not, the consequences of both the aspects should be shown.

I: Ok.

P: Talking about the question where it is asked that if your parents or caregiver ever wished you were never born…it should be rephrased…you will do it right? Instead of *Aasha*, you can write *Apekchya* (Different words used in Nepali for wish).

I: Ok.

P: It is relevant. Adolescents sometimes say "you could have not given birth to me" or "I wish I was never born" kind of things…

I: If we use such tool for predicting risk among adolescents, how would it be? Is this questionnaire lengthy or short? What are your view on this?

P: It is quite short (Laughs) for psychological research.

I: Is it? Is it because it won't be enough?

P: There could be something which we can add here in this questionnaire and revise it accordingly.

I: What additional things can we add here?

P: I think that we must add something about the personal factors. If you can print it and give it to me then I can go through it and tell you what other additional information could be added herein. You have also included the section for other experiences and in the section for personal factor, you have one for sex related and other for substance related. These are only about their personal factor that have been included here in this questionnaire. I think that we can add some more about it here.

I: Ok. What more could we add here? Can you please elaborate?

P: Yes. Though you have already included academic, family but what about peer pressure?

I: Ok.

P: Yes, about peer pressure! And there are things about self as well such as hormonal changes or body changes and the effect brought up by those kind of changes. So we can add such things. We can ask them if they are feeling different or some kind of confusion because of their changes.

Reference 2 - 0.92% Coverage

I: You said that you use different version of forms to different respondents. So, are the questionnaire different or…?

P: The possible answers are same but the questionnaire is different. For example, if we give the form to teacher then there are questions about the classroom behaviours of that student or adolescent which is observed by that teacher and for the parents, we give them questionnaire where the home environment are focused while personal information are focused among the adolescents.

I: You link up all of them and give it to them so that the information…

P: And we go for the adolescents for the conclusion at last and use our clinical judgement.

Reference 3 - 2.22% Coverage

P: I think there must be many questions that should be added, I feel that the questionnaire is short.

I2: Like you said, we have to provide relevant recommendation to those who are at high or low risk of depression. We have included things for their treatment and some intervention for the ones in high risk group and for those with low risk of depression, we have included things about their life styles. Do you think this much information is enough or we have to provide some additional information for those with high risk and low risk of depression?

P: Is there the information about what depression is basically? This is what they should know about. And also, you have to put such information about referral as well so that if in doubt, adolescents can visit them. You can put some of the sites or contact details of the key person to whom we may refer to if we have high risk of depression. It is fine then.

I: We have categorized the risk into two categories; high risk and low risk. You may have been familiar with different categories while using such screening tools. Do you think this category is enough or do we have to add some other categories such as mild or moderate?

P: If it was about disease, we could categorize it into mild moderate or severe but it is about risk. High risk or moderate risk could be fine but I don't think it is necessary because it is just about risk factor.

I: So high and low risk are enough for this?

P: Yes.

I2: It is about the risk of having depression in future…

P: When we use the suicide risk assessment, we use high and low risk to define risk of suicide.

Reference 4 - 0.29% Coverage

I2: Are there question about the conflicts they have with their friends and about the causes of such conflicts?

P: No.

I2: Because if we look at depression, it is about such causes or risk of depression…

Reference 5 - 0.18% Coverage

P: Ok. And in that question, you could put *Dam haru* along with *Chinnaharu* (Both Nepali words used to indicate physical mark).

Reference 6 - 0.08% Coverage

P: It is quite short (Laughs) for psychological research.

Reference 7 - 0.14% Coverage

P: There could be something which we can add here in this questionnaire and revise it accordingly.

Reference 8 - 0.69% Coverage

P: I think that we must add something about the personal factors. If you can print it and give it to me then I can go through it and tell you what other additional information could be added herein. You have also included the section for other experiences and in the section for personal factor, you have one for sex related and other for substance related. These are only about their personal factor that have been included here in this questionnaire. I think that we can add some more about it here.

Reference 9 - 0.12% Coverage

P: Yes. Though you have already included academic, family but what about peer pressure?

Reference 10 - 0.39% Coverage

P: Yes, about peer pressure! And there are things about self as well such as hormonal changes or body changes and the effect brought up by those kind of changes. So we can add such things. We can ask them if they are feeling different or some kind of confusion because of their changes.

Reference 11 - 0.14% Coverage

P: I think there must be many questions that should be added, I feel that the questionnaire is short.

Reference 12 - 0.50% Coverage

P: Is there the information about what depression is basically? This is what they should know about. And also, you have to put such information about referral as well so that if in doubt, adolescents can visit them. You can put some of the sites or contact details of the key person to whom we may refer to if we have high risk of depression. It is fine then.

Reference 13 - 0.30% Coverage

P: If it was about disease, we could categorize it into mild moderate or severe but it is about risk. High risk or moderate risk could be fine but I don't think it is necessary because it is just about risk factor.

Reference 14 - 0.07% Coverage

I: So high and low risk are enough for this?

P: Yes.

<Files\\KII\\ > - § 6 references coded [5.97% Coverage]

Reference 1 - 2.31% Coverage

I: So, you have looked at the questionnaire. Who could be able to use this tool? For now, it is targeted for adolescents that means, adolescents themselves can fill up this form or some researchers could ask them the questions and get this done. So, would it be effective if parents/guardian or health workers or teachers fill up this form in behalf of their adolescents?

P: If health workers or parents are the ones to fill of the form then we have to add "don't know" as the option for most of the questions. They may not know most of the things adolescents feel. So, there must be options such as "doesn't apply" or "I don't know".

I: If teachers or someone other than adolescents themselves fill up the form then we should add such options?

P: Yes!

I: If we add such options, then would it effective? Should they fill up the form? Or should this form be filled out only by the adolescents as the target group is adolescent?

P: We can't say this without doing research. If we once go in the filed with this question using this to parents, then at least we can say that we could get some kind of information from parents even this tool is developed targeted to adolescents. Or we can see the information given by adolescents and parents and find out the correlation and say whether or not it is acceptable. We have to change the weightage of each questions accordingly because the way adolescents fill up the form may not be correlated to that of their parents; parents may not know about abuse that had happened with their children or they may not know that their children had fight with someone and they may so no at this point. We could adapt the data accordingly.

I: So, other could also fill up this form on behalf of adolescents?

P: For now, not really!

I: (Laughs) if research shows that then…

P: Doubtful! It could be extended but I won't be able to say that others could fill up the form on behalf of adolescents.

Reference 2 - 1.25% Coverage

I: Could you recommend some other changes? Like you said, the person might react differently after knowing that s/he is at high risk of depression and this is a challenge. So, what could we do to mitigate such challenges?

P: Umm…

I: Like you said, we have put some kind of information for those with high as well as low risk of depression. Do we have to add something to it or is it fine?

P: For the high risk, we can put some information for healthy life styles and include some sites which could help them to be mentally healthy and for those with low risk as well, we can say them that they are fine but still mental health problem is such that anyone could get it and there's way to prevent it by following healthy life styles. We can keep such generic message for both of the category and add few more information for those with high risk. In TPO as well, you have some materials for psychosocial health. You can provide such materials to people and ask them to go through those materials and get informed about the mental health issues.

Reference 3 - 0.31% Coverage

P: If health workers or parents are the ones to fill of the form then we have to add "don't know" as the option for most of the questions. They may not know most of the things adolescents feel. So, there must be options such as "doesn't apply" or "I don't know".

Reference 4 - 0.20% Coverage

P: What we can do is, put an option of high risk and include those who are at very high risk of depression but then form other options for mild or moderate as well.

Reference 5 - 1.12% Coverage

P: Yes. Sometimes, while trying to prevent, the problem might increase. For example, during and after earthquake in our country, we thought people would be having mental stress and that's why everyone were running here and there for providing services or seeking services, but instead people were more resilient to that incident. So, we have to bring the needy ones as well as focus on the natural healing process and normalize people. For that…what have been other sing as the result, is that the same like we are using; high and low risk?

I: … (Trying to speak but respondent continued)

P: In physical illness, it is fine. But in case of mental illness, may be the words have to be changed in some ways. We can say some ways for preventing them from getting depression if we found out that they are at high risk of depression. Instead of high risk and low risk, maybe we can find out other alternatives for making it more constructive.

Reference 6 - 0.78% Coverage

P: For the high risk, we can put some information for healthy life styles and include some sites which could help them to be mentally healthy and for those with low risk as well, we can say them that they are fine but still mental health problem is such that anyone could get it and there's way to prevent it by following healthy life styles. We can keep such generic message for both of the category and add few more information for those with high risk. In TPO as well, you have some materials for psychosocial health. You can provide such materials to people and ask them to go through those materials and get informed about the mental health issues.

<Files\\KII\\ > - § 1 reference coded [0.53% Coverage]

Reference 1 - 0.53% Coverage

P: In general, it is not that good to keep questions like this; few questions in one screen. You should have at least 7 to 8 questions in one screen because clicking "next" and "next" every time is boring. You are giving this to adolescents…nowadays, while making advertisements as well, they are said to make it less than 30 seconds.

<Files\\KII\\ > - § 12 references coded [5.89% Coverage]

Reference 1 - 0.05% Coverage

Whatever it does, it's not enough.

Reference 2 - 0.77% Coverage

Looking at this tool. Looking at the prototype … I don't think it will be useful. Looking at the prototype it looks like… If they go somewhere … it's just to say like they have these things.

I: Ok, yes.

P: It's just use for the information. I think just to get that …

I: Don't you think it can predict risk?

P: No, I don't think it predicts risk … I don't think it gives something else.

I: If questionnaires are included …

P: …because thing…how can you say there is risk or not in coming 5 years.

Reference 3 - 0.48% Coverage

Because you don't know what will happen in those five years. There has to be something to trigger. There has … often there is something to trigger... So, if there is not such things to trigger the person according to this tool but in those five years, the person is triggered by it then? How can you ... How can you…?

Reference 4 - 0.36% Coverage

Of course it's like ... it's important … but this thing is about today. If years after one person might come from *Hetauda* to *Kathmandu* valley. Their relationship with family might change. After that prediction can't be done… I don't know.

Reference 5 - 0.34% Coverage

Because living condition (is same)… what are the chances that all three of them have depression? There is almost no chance that all three of them have depression … *laughs* All of the members in the family cannot have depression.

Reference 6 - 0.12% Coverage

Is this not enough. In your opinion what should be added?

P: Everything!!

Reference 7 - 0.41% Coverage

We diagnose depression and does it change in the personality? Well this was before changing it…

P: Yes … so how only not the risk … where is the risk? Of course broken families …where there is ... rate may be higher … they might say that … But even then I don't know.

Reference 8 - 1.07% Coverage

Okay. What you said is right, I understand. What actually we are trying to make it like is suppose a person have a broken family, or all such things then what might be the risk of the person for having depression in future…this is not the final version of the tool because we have been taking

feedback from you and others also. And we will then modify it according to the need and feedback. So, do you think the risk of depression can be calculated then after?

P: I don't think you can!

I: If we made such a tool then will it be useful or not? What do you think?

P: If it is reliable and valid then yes it will be helpful. So you do the test psychometric and if it comes strong then it will be useful.

Reference 9 - 0.84% Coverage

What do you think, who can use this? We are trying to take it in that way. This is just a prototype now, but here we are trying to ask you is, if we made such tool which would predict the risk of people of having depression, then who can use that?

P: Because you … your family questions like broken family and all that … which is very common in Nepal. Domestic Violence is very common in Nepal. So... but then I don't know … How?

I: Like you told if it fill up all the criteria of validity and all then this could be useful?

P: This could be useful!

Reference 10 - 0.21% Coverage

Everything is mentioned … Broken family, domestic … family ... violence everybody because we don't have any idea about who have depression.

Reference 11 - 0.26% Coverage

If someone says that somebody in the family needs them, then don't you think they should give some focus. For example, *my mom ... my mom hates me* it's very unlikely you know.

Reference 12 - 0.99% Coverage

So can't you focus on the question ... that they have showed themself. So, see if u want to talk about it … Talk about it, can't you offer help. So it seems that you have some difficulties with your mother ... Can we talk about it. And let's see what we can do? This may involve your

mother… something like that. So this way we ask questions to children's rather than telling them to share their problem.

It would be better if we know that they have problem and we offer them a helping hand and ask them to talk to us if required. And tell them that "*You seems to have a problem with your mother/or anyone, let's talk about it, you will feel better*."

<Files\\KII\\ > - § 1 reference coded [1.11% Coverage]

Reference 1 - 1.11% Coverage

I: All right. Is there anything else? It is said that the information for the high risk is this and the information given to the low risk is this. Do you feel that this much information is sufficient for you? Or do you think that the people at high risk need to be told some more things?

P: There is a phone number that has been put here. There are phone numbers and websites that have been mentioned here. These says, we could also add other links that might be there for social media if we have it. If there are toll free numbers then maybe it would make it easier (for patients). It might be easier at times if there are toll free numbers which do not cost anything to call. It would be easier if the contact numbers were separate for the men and the women and the children.

<Files\\KII\\ > - § 1 reference coded [0.99% Coverage]

Reference 1 - 0.99% Coverage

I: So, you have already read the last segment of the risk calculator which is about the information we provide to the ones who are at high and low risk of depression. What do you think of it? Do you think we have to put some additional information there?

P: The information here is very good. I don't think we have to add any more information herein.

I: Is it?

P: Yes.

<Files\\KII\\ > - § 6 references coded [6.37% Coverage]

Reference 1 - 1.45% Coverage

P: (Respondent finishes looking at the tool).

I: For now this is just the prototype. So, after a level of calculation has been done and the score for risk has been known, assuming that you are an adolescent, it reveals whether you are at high risk or low risk. If the result is displayed under the high risk category then you will receive an information like this (showing information box) where you will have this kind of information available in the box. We request you to go through this as we might require your views on this as well. So, if anyone is found out to be at the high risk then these kind of information's are provided. What are your opinion regarding this information? Is it sufficient or we need to add anything more?

P: (respondent reading information) it seems as if we have not written about how to cope?

Reference 2 - 0.81% Coverage

I: That's the reason why we are asking you to have a look at this and what you think is missing out (nervous laugh). For now, from our side we have included this amount of information. What are your views on this?

P: (respondent reading information) Here it is mentioned. Ok. I was worried about getting categorized under the risk but solution regarding how to cope with it was not given. But seeing that the link has been provided in the box below, it is ok.

Reference 3 - 0.66% Coverage

I: This shows information about one being in high risk category. So, what should I talk about first? High risk or low risk?

P: Anything is fine.

I: Ok. This is high risk portion. If at low risk, this is the one that gets displayed.

P: Ok

I: We are planning to provide different information for different risk category established.

P: Ok. Its fine (reading information)

Reference 4 - 1.82% Coverage

I: Ok. After all this is completed, it is to be submitted at the end. So now, as you have looked at the entire tool, what is your initial response towards risk calculator tool as it has been created to predict depression among adolescent. Without being content specific for now, as there might be more content to be added later on, what in general are your thoughts towards this tool?

P: Though not being content specific, I liked the content as well because all clinicians are human mind and few points may be missed out but if we use the risk calculator tool then there won't be chance of missing out anything important. This being the reason, if we have an access to this tool then no domains will be missed out. As I said earlier, looking at the tool, I feel as if I (human brain) have missed a lot of points but is it is normal thing and is obvious to get off our mind. So, if we design a tool, it will definitely help us move forward, including everything without leaving any aspect behind and I liked the way of this categorization.

Reference 5 - 0.40% Coverage

I: Only after the categorization of one falling under high risk and low risk, we take further steps. So, will it be ok to say that your initial response towards the risk calculator tool is satisfactory?

P: Let's say, it's good.

Reference 6 - 1.24% Coverage

I: Previously we discussed that when result is revealed, we will give this kind of information under high risk and that kind of information under low risk category. Do you think such kind of information is adequate?

P: umm… In that, we discussed that under high risk we will be doing this and that kind of things but things mentioned under low risk can also be done under high risk. I think, this was missed out. Like the information mentioned in low risk about taking care of food and environment, the person in high risk also cannot omit that thing. I think, it was missed out for high risk category. I do not know if it is in the link but while having gone through this, I think this was missed out.

<Files\\KII\\ > - § 2 references coded [0.79% Coverage]

Reference 1 - 0.37% Coverage

P: We might need to provide some more information like the one we can highlight that the person at high risk can himself or herself do something.

Reference 2 - 0.42% Coverage

P: Like not staying alone, sharing their issues with their close friend, share network to understand one another or involve in the activities they like to perform.

<Files\\KII\\ > - § 1 reference coded [0.90% Coverage]

Reference 1 - 0.90% Coverage

P: Yeah, this is good in a way that you're…(laughs) the last part I like with "high risk" and "low risk" potential. But uh, even if it is a "low risk" potential we cannot be so sure that it *will* be low risk, because it can change any time.

<Files\\KII\\ > - § 12 references coded [3.84% Coverage]

Reference 1 - 0.11% Coverage

P: I'll go from the bottom to top now. (Rereads section) Ok, there are no questions related to school.

Reference 2 - 0.37% Coverage

P: But, this is like a whole dropout, you know? Dropout is like, extreme. And you're not using…either school withdrawal, or school refusal, or…this is like failure, and a lot of other stuff. So, when we talk about any type of mental health issues, for it to become a disorder— since we're talking about depression, it's a mental health disorder, right?

Reference 3 - 0.38% Coverage

So…there has to be a component of dysfunction. If it's an adult, for their social dysfunction, it could be not being able to perform their roles and responsibilities at work and in the social setting, or in the family setting. So, for the kid, it should be school, assuming that most children go to school. So, any kind of dysfunction in school has to be there.

Reference 4 - 0.16% Coverage

P: But, then factors that could be there in school, you know? Like, bullying-related issues, or corporeal punishment by a teacher, those kinds of stuff—

Reference 5 - 0.12% Coverage

P: Because, these are common stressors that we see for children, you know? Teachers and other friends in school.

Reference 6 - 0.31% Coverage

P: It could be something that's more general or subtler, and then you go deeper. Because family-related issues could be stronger for the child to deal with in the beginning. So, if you ask, "how is your relationship with your mother?"—instead of mother, you probably start with, "your friends."

Reference 7 - 0.04% Coverage

P: And then, you go into the family.

Reference 8 - 0.33% Coverage

P: I think first off…you know, if you go through depression—the chapters on depression, the literature we have—family history of mental illness? That's not there, the question. Maybe we need to have that. If any sibling or family member has had depression, has had suicide, and those kinds of stuff, then possibly?

Reference 9 - 0.81% Coverage

Family's history of substance use and all…so those could be the biological risk factors, which has already started. So, we should not miss that.
Besides that comes the child's own individual personality. Like I talked about the 3 environmental factors, the fourth factor is the child's own intrinsic qualities—which also includes the biological ones. But then, it's how a child usually copes with stress. So, we could ask things like, "if you're stressed, how do you deal with it?" So, we can kind of figure out the coping mechanisms the child has. It could be talking to others, or you know…whether the child sleeps, goes to play? Whether it's distraction or actually finding a solution for a problem, or just venting it out…So, what's the child's coping mechanism.

Reference 10 - 0.33% Coverage

P: And we could probably ask stuff like, "in the past, have you ever"—so, we're screening for future, but depression can come in episodes. So, we can screen for past episodes, whether the child possibly has had any depressive episodes in the past. We could say, "In the past, have you ever felt like (so-and-so)?"

Reference 11 - 0.38% Coverage

P: What we could do is go down both of the risk factors of depression to begin with, and the questions could be tailored based on that. In children, there can be a lot of comorbidities that go along with mental health issues, so…when a child has a pretty big mental health issue, there's a higher chance that the child has comorbidities, and in terms of adults.

Reference 12 - 0.49% Coverage

We could possibly screen for other comorbidities as well. Like, if the child has anxiety issues than the child possibly has higher risk for depression as well. If the child uses substances, then depression. If the child has some disabilities, then high-risk for depression; if a child has some debilitating condition or pain-related illness, then possibly depression. These are established medical conditions which also have depression, so maybe we could add those.

<Files\\KII\\ > - § 5 references coded [2.11% Coverage]

Reference 1 - 0.39% Coverage

P: Upon seeing superficially, I had thought about it. We will ask about the relationship between parents and teachers. I think something about sharing forum is missing there. For example, they have good relationship with their parents but is there environment for sharing or not? I think that question should have been included there.

Reference 2 - 0.29% Coverage

P: For example, whom would they share when they have problem? Is that person, their parents or not? What happens after he or she share the problem? Will the parents listen to their problems or not, etc. I think we should ponder upon that part.

Reference 3 - 0.57% Coverage

Another thing is that, we should also link it with friendship. While having problem or tension, we should also ask what sharing forum he or she has. If he or she does not have any friend, we can suggest him or her to make a friend whom you can share your problems, which can help to reduce her problem even if she is at high risk. Similarly, we can also question parents about how they have been caring their child and whether or not; they are caring about their change in behaviors.

Reference 4 - 0.41% Coverage

P: There are words to be revised in our context. We might also need to link with family rather than only addressing parents because we live in a joint family. Sometimes, they might have good relationship with their parents but the cause of their problems might be the relationship with other family members. We might need to include such things as well.

Reference 5 - 0.45% Coverage

P: Designing such a tool which helps to determine whether he or she is in risk or not and it is good in itself but I think we could add about why there is risk, what the reason of having risk is or is it only family and how to get out of risk. If the message could be two sided like in BDI, which adds social factors and other facts and what his or her responsibility to minimize it is.

<Files\\KII\\ > - § 1 reference coded [0.28% Coverage]

Reference 1 - 0.28% Coverage

P: We are just looking at a few aspects right now. If such a tool is made, then all of these things seem to get included. I think that it would be good.

<Files\\KII\\ > - § 2 references coded [2.43% Coverage]

Reference 1 - 0.58% Coverage

P: It is very easy. The list that you have at the end of it should make it clear about where they are supposed to go with concrete referrals if they were suffering from this, then it would be easier. They would know where they could seek help. Right now, people do not know what they are supposed to do even after identifying that they have depression. That part would have been easier.

Reference 2 - 1.84% Coverage

P: We might have to add because…as I have told you before, we are stuck in the terms. Nowadays, we are grouping everything (every mental health illness) within depression. If we ask patients why

they have come to us, then they say that they suspect they are suffering from depression no matter what has happened to them. So, we see that terms stick here. Now, if an adolescent is termed high risk or low risk, then their impression should not be such that their life is over if they are high risk. They should not even go for problematic behavior if they are low risk. That information has to be clarified.

As you had suggested earlier, we should not bombard them with a lot of information all at once. What is this tool trying to do? If we are trying to treat them with this tool then it is insufficient. But if its target is to only look at the possibilities of what might happen and we are only there to suggest them about the next step from there, then let us only focus on that. It is because we cannot expect it to treat everything. It is only suggesting whether they have to seek help or not. If we give them advice about the places that they have to go to seek help, then the respondent will not be overwhelmed.

<Files\\KII\\ > - § 6 references coded [3.79% Coverage]

Reference 1 - 0.46% Coverage

P: The questions are simple and participants can answer them easily and freely. There are no difficult questions or questions that should not be asked to them.

Reference 2 - 0.91% Coverage

I: You think that the incorporated questions here cover all the things?

P: Yes.

I: Do you think that this could be misused? Or do you seen any negative consequences that it might have?

P: No, I do not see anything as such. There are no personal questions or any other questions that should not be asked to them.

Reference 3 - 0.20% Coverage

I: You think that they can answer them?

P: Yes, they can answer them.

Reference 4 – 1.18% Coverage

I: We have showed you how it can calculate how much problem they might have or not. It shows high risk and low risk and where what is done. What else can be added to it?

P: I think that the family relations is covered in it.

I: Yes.

P: It also covers the school and the social experience.

I: Yes.

P: And then there is the relationship with friends.

I: Yes, there is the aspect of relationship as well.

Reference 5 – 0.62% Coverage

P: Their personal interest, about their hobbies, wishes, and their field of interest can be added, what makes them happy. If these questions are also included, then I feel that it will help to calculate it as well.

Reference 6 – 0.43% Coverage

P: Yes, we need to follow up after this is done. The hotline numbers could be provided to list out the problems that they have and it can be solved.

<Files\\KII\\ > – § 1 reference coded [2.08% Coverage]

Reference 1 – 2.08% Coverage

P: It is obviously that the information provided her is not enough. While giving enough information, probably we (professionals) should design message for them in simple language. For example, if we walk in cold for 2-4 days, we get cold and cough which is natural because our body can get cold and at such time, we need medicines for our body. Similarly, while reading or when

our wishes and desires are not fulfilled, our *man tatinchha, dimag tatinchha* (stress in heart- mind and brain) and *man khinna hunchha* (feeling sad) which is also natural and instead of saying them 'high risk' we can further explain them as their body or heart mind is sick and as we treat cold, we need treatment for our heart mind. If we can give similar simple message in understandable way, so that they can understand in simplified way rather than thinking something huge is happening to them. So, if they can understand in simplified way, they can prevent the possible consequences and go for support. Otherwise, this language is not enough.

<Files\\KII\\ > - § 2 references coded [1.54% Coverage]

Reference 1 - 0.26% Coverage

P: I think we can also include matters related to love affairs, which is also contributor.

Reference 2 - 1.27% Coverage

P: If we do not have *pahuch* (access) to anything, we leave trying to reach it. For example, many people in village die because of not trying to reach medical treatment facilities due to not having easy access. Therefore, if we can open such organization in each district and provide his or her phone number and location and develop the app easy and understandable by everyone as you have therefore used easy language, it can be more effective.

<Files\\KII\\ > - § 2 references coded [1.43% Coverage]

Reference 1 - 0.81% Coverage

Well it depends upon what all is included. Only reading this will not be enough because on individual basis more things can come up. Most of the things may be covered up but certain things should be added.

Reference 2 - 0.62% Coverage

Well in that there is not much. There is only the information about. Seeking help if they are at risk of depression. I think just help seeking is not enough?

<Files\\KII\\ > - § 3 references coded [3.23% Coverage]

Reference 1 – 0.58% Coverage

P: Now, how much we talk with our friends? For instance, we had talked that due to the internet, social surroundings, (adolescents) are being isolated. It is said that we can minimize such problems through sports, sports can be one tool. If it could be customized in your apps…

Reference 2 – 2.33% Coverage

P: We should… How are they involved in decision making, elders… things about participation should be also there, their opinions are being heard at home or not, are they being involved during decision making at home or not. They (adolescents) first learn from home that how do the elders take leadership, how they lead the home, how they implement the decision making the process. Then it might be from child clubs, from schools… how is their participation in school, if the adolescents made involved in meeting or not while making the decision about children and adolescents, if their perspectives are considered or not, which is also important. Like, we do not get to know sometimes. We grew up in such a manner that when we were a child our suggestions were not considered while making a decision about us. Neither was taken at home nor at school. We could not learn how the elders make decisions, how to speak in front of the mass at that age. Now the youth are forward. Certain/some organizations have been engaged to teach about the decision-making process, perhaps the rights to participation come in that too.

Reference 3 – 0.32% Coverage

P: Yes, if these things are there, it … it seems to be a small matter to see but it can be the key role player for life-changing. It can be one indicator.

<Files\\KII\\ > – § 1 reference coded [0.85% Coverage]

Reference 1 – 0.85% Coverage

P: It should be made less technical. More than the written instructions, pictorials can be used which would make them fun to use. If more questions are asked, chances of them leaving in between are more. From the number of questions to how the questions to be asked to the platform which they use much should be considered.

<Files\\KII\\ > - § 4 references coded [3.40% Coverage]

Reference 1 - 1.57% Coverage

P: One good point is "You are at risk. This kind of risk can come in future. There is a phone number. We can help you". I think it would be good if motivating information such as "the risk has been seen, for now, it can be changed" could also be incorporated. For instance, " You might have risk in future but if you choose such behavioral pattern, that might not occur, if you take care of your health and all, that might not happen".

Reference 2 - 0.42% Coverage

I: If that kind of information can be included, s/he can be stopped from going to the negativity, isn't it?

P: Yes.

Reference 3 - 0.61% Coverage

P: If information about how it can be minimized if depression occurs, then how it can be reduced if they fall in risk could be given through link. That would be good.

Reference 4 - 0.80% Coverage

P: Result should be told but rather than saying that s/he is at high risk, in a positive way we should say "don't get panic, for now, you are seen in high risk but you can take help". There should be a pop-up in this way.

<Files\\KII\\ > - § 2 references coded [1.67% Coverage]

Reference 1 - 0.29% Coverage

P: When gone through now, I do not have any... The shorter it would be, the better. It has family experience, school, and social experience.

Reference 2 - 1.39% Coverage

I: More than questions, anything more than that. For instance, you said earlier only giving link is not enough, where the (services) can be taken, how they charge for service. That can also be added.

P: Yes that can be added. How to do that? If need to pay...if said this link, then you need to inform, you might need to pay this amount there or it can be free, this has helped. Information about free might come next but we need to think, what would be the role of TPO. To make it function continuously, what would be the sustainable mechanisms? Who is needed to be involved? I think as a stakeholder if the Ministry of Health and Population would be contacted for this forming a...

<Files\\KII\\ > - § 3 references coded [4.54% Coverage]

Reference 1 - 1.74% Coverage

P: That's right too but another thing is a topic of social media. You should add that too. What's happening in social media? How many people have you tried to contact? How many Facebook friends do you have? Whom do you frequently talk to? What kind of things do you talk about? Have you ever been angry due to those talk or not? So, questions like this can be asked there indirectly. There's no way that technology is not used. But a there is limit of how much it should be used. If you add these things I think it will help too.

Reference 2 - 2.04% Coverage

I: So, we have given the contact numbers of treatments here. If they see that they are in high risk, they can click on the website. And they can find where to go, which doctor to go and what kind of treatment should be done. And if they really are in depression they will know what to do to make it better. Everything is written there. Even if the risk is low, they will what to do if they suffer in the future. So we have given these information. So, what do you think should be added to this?

P: It's good. You have done research about this and it's good. You have made it by discussing it with the counselors, right?

Reference 3 - 0.75% Coverage

P: Like I said earlier, you can add those things. If you do this in schools of villages also through government organizations or any clubs, I think not only the students will learn but many other people can also learn from this.

<Files\\KII\\ > - § 3 references coded [2.56% Coverage]

Reference 1 - 0.37% Coverage

P: So, the symptoms should be kept in the questions than in the link.

Reference 2 - 1.01% Coverage

P: Yes, it will be better to keep the symptoms. It will increase the awareness and it will be better that way because there might be no access for the links. That's why symptoms can make them aware.

Reference 3 - 1.18% Coverage

P: In addition, there should be stigma related questions, pathophysiology should be explained very well about how it can cause mental health problems. Another is environmental factors and malnutrition. These things can also be added.

<Files\\KII\\ > - § 2 references coded [3.11% Coverage]

Reference 1 - 1.38% Coverage

P: It might be useful if developed. However, the other aspect is that depression seems to have been perceived from the psycho-social perspective only. The big challenge is how the biological component would be identified. Perhaps, the family history would come there. Depression in itself is not just a psychological matter, but the biological aspect is also there. Issue about the biological vulnerability too comes, if these aspects could also be addressed by the tools, it would have been easy.

Reference 2 - 1.73% Coverage

P: Many things at once…More than giving a lot of information, the big thing is change brought in their life because of the information provided. Practically, it has no meaning how much information is. The big thing is that how much the information brought the changes in the person's life. So let us give such information which a person can easily accept and implement in life. Knowing a lot of things is not big in itself. I mentioned about Steps. Data of Nepal is indicating… It is high time for us think that if we should only inform people or bringing change in their life through information. This is a big challenge.

<Files\\KII\\ > - § 2 references coded [5.77% Coverage]

Reference 1 - 2.83% Coverage

P: I think that the interesting in it… The interviewer doing it is in one place. Usually, if a child comes to the interviewer… we always say this whenever we train somebody… It is important… need to work for…The other is… The other version of it could be like a self- rater. At the current time, every child, adolescents as have digital mobiles and all, this self- rater should be used to gather self-rating of own and gather own scores. Further, provide the information automatically that they are in high-risk. I think if that can happen, then it will be useful. It is not that hard to develop another version of this. Mainly, it is about identifying the stress domains of adverse life. If could explore cultural specific adverse… that type of is developed, then it can be useful in another form.

Reference 2 - 2.94% Coverage

P: The psychological coping is being so much simplified that it is not necessary to visit health workers all the time. My point is people might not come. In several settings, self-help packages are available. That can be made used too. WHO (World Health Organization) had engaged from TPOs only, you might not know. Developing online-based self-help materials, which might be the YouTube videos, chat-bot and so on. The discussion is on a process to include issues like self-help in evolving digital… targeting urban, and peri-urban adolescents. So in this, children identified the high-risk on own and the further link can be there. If that project comes to us, that might become

culture-specific too. We can then directly link those matters… Especially, if adolescents themselves… They will be empowered. They will do that too.

<Files\\KII\\ > - § 2 references coded [3.63% Coverage]

Reference 1 - 2.58% Coverage

P: I think one about mobile should be added. Mobile has created isolation in/from the family. There used to be the family gathering earlier. As interaction used to happen, there use to be sharing as well. These days, everyone is into Facebook, mobile everyone is single/alone/isolated. Nobody cares for anybody/others. We can read in news too that the risk of depression is more in near future. So there should be a few questions about mobiles and awareness should be created abut that these can happen through mobiles. This matter should be included in such a way that some data could be derived too.

Reference 2 - 1.05% Coverage

I: If there is anything you want to add, for instance, if this could have been added in identification and management, aspect on mental health could have been taken forward effectively….

P: No. I said about adding about mobiles within the tool.

<Files\\KII\\ > - § 1 reference coded [2.30% Coverage]

Reference 1 - 2.30% Coverage

P: For the present context there should be questions like; how much are they involved in outdoor activities, how much do they study, how much do they use the gadgets and how much of the time do they spend with the family. If these questions are also incorporated there it can also give some ideas. For example a child goes to school from 10 to 4 o'clock but after returning from the school what is his schedule? In some schedules they might watch TV after returning or only use gadgets and doesn't go anywhere. So these things can also be included as the risk factors.

<Files\\KII\\ > - § 2 references coded [4.65% Coverage]

Reference 1 - 1.54% Coverage

P: I think maybe the questionnaires should be increased. As I have seen you have focused mostly in the relation with family. Maybe you can add the questions related to their career, bullying, humiliation, feeling lonely, crying etc. I think that kind of questions should be asked. Or have your parents ever scolded you in front of your friends due to your study? Also about friends. I think these kind of things are the main problem about today's adolescents. These things are pushing them towards depression but I think we are missing to address these things.

Reference 2 - 3.11% Coverage

P: I don't think only this can figure out if the person is in risk in the future. Depression of each person has their own reason. Everyone may not write the real reason behind their depression. For example; has there been any accidents that has affected you for a long time? Or anything related to friends. What happens here is section is changed and the two friends are separated. So when we see that from outside it looks like a small thing but from inside, when a friend since so many years is separated, they feel very sad. So that kind of questions should also be added in this. It's about how to perceive the same thing. It is okay in case of family related questions and there are also some things that needs to be improvised.

The part of counseling from the teachers is very important in the country like ours where we have not done anything to address mental health problems. In western countries, they are very aware of the mental health. They have also contributed money it. But we have not done anything, we are doing it in a very less amount. Even if someone uses the word psychiatrists they say that they are not mad.

<Files\\KII\\ > - § 2 references coded [0.58% Coverage]

Reference 1 – 0.10% Coverage

P: Here are only the basic things…

Reference 2 – 0.48% Coverage

I: Do you think we have to add anything in this tool? What do you think those could be?

P: I don't think that is needed. I think these are only the topics…

<Files\\KII\\ > – § 1 reference coded [0.64% Coverage]

Reference 1 – 0.64% Coverage

P: So, she have been asked to do some exercises which makes her mind well. She have to do such things also. Some needs medicine also sometimes...there could be things that doesn't have impact on children's mind…how can we make them better, such kind of information should be there.

<Files\\KII\\ > – § 2 references coded [0.75% Coverage]

Reference 1 – 0.66% Coverage

P: ummm, even if this is shared online, the risk should not be mentioned and while filling if they found something then they should be instructed to reach out to their guardian and guardian will react that way. If it is done this way, it would be easy. *"If someone has these symptoms then discuss with your guardian and guardian should be able reach out with the given contact details for high risk",* this would be easy if done this way.

Reference 2 – 0.09% Coverage

P: ummm, I do not know more about that, I thinks its fine.

1. Code Query Output

<Files\\FGD\\ > – § 7 references coded [4.33% Coverage]

Reference 1 – 0.40% Coverage

P3: But would they fill this up if we ask them to?

P5: They know that they are having depression. They will obviously look at the risk they have.

P3: Yes, but they might now seek for help because…I think some of the children wouldn't even look at the tool…

Reference 2 - 1.04% Coverage

I: As I already said, rather than solving the problem, it is about having the risk of depression in future because of their present activities. And if they are in risk, we can early provide them support and help them. This is the major objective of this tool. So, will they fill up the form? And if required help, will they seek the help or not?

P3: No!

P5: Most people won't fill it up.

P3: Because not every parents know about it. If their children get angry, it doesn't mean they are having depression. And parents might not know that what their children are filling up. It is not necessary that everyone is literate. So, I think it is better if we go it directly.

Reference 3 - 0.23% Coverage

Children might not fill it up by themselves so we can talk about it in the society and then people there can encourage children to fill up the form.

Reference 4 - 0.63% Coverage

Sometimes, friends can tell each other about it and tell them to fill it up as it will be good for them. We are doing this for good. It is for depression, there is I guess for alcoholism and drug addict also. I guess there are such applications. We can…there are some applications for online counselling also. But even though it is available, alcoholism and drug addiction are still present in the society.

Reference 5 - 0.79% Coverage

But since when people know that there are such application available, people can tell it to others that such applications are available and we can know if we need help or not. Though the choice is per the individual that if they want to seek the help, you can at least show them the path. Like if people want to jump from the cliff, we can be a medium to tell such people that even if they want to jump, somewhere down there the landing point is available if the application (Risk calculator) is developed.

Reference 6 - 1.00% Coverage

P5: Yes! Like I know I am going to have a problem because there is something in my mind which I am not being able to throw out of there. And that is going to make me in problem. And I even know I need help. And that help comes within the family. And if I don't get that help then that will be difficult for me. I know all this but I can't get help by myself, I need a supporting hands, a strong one and for a long time. If you have problem in a place, you can move out, but if you have problem in your mind, where would you go?

P3: In the night time, the problem are more! (Laughs)

P5: We can't go away from the problems we have within us.

Reference 7 - 0.23% Coverage

I: In case of your children, do you think they will fill up this type of tool?

P3: My daughter will surely fill this up and I don't know about others…

<Files\\FGD\\ > - § 10 references coded [6.19% Coverage]

Reference 1 - 1.58% Coverage

P6: I would like to share my experience being based on the topic here. I had developed a set of questionnaire including the behavior of child, things about the classroom as well as their home. Each question had some weightage. But what happened was, children were too smart that they didn't say about their behavior (Everyone laughs). They analyzed the question and thought about the question which would help them score the highest mark, they would tick that answer that questions there. I was not able to differentiate the level of those children. I tried to do exactly like this! I don't know all the questions here as we went surfacely, but in this case as well, children will show you that they are smart. Normal children will answer what exactly they feel but who are a bit clever, would tick in such a way which will indicate his behavior balanced. I have experienced this. I didn't get the result as I was expecting. If I had, then I would have asked other teachers to do it as well but I didn't so I leave that as it was. Another teacher also used an approach something like this, he asked students about the positive things about other teachers but then the student said that they don't like anyone! (Laughs) I think negativism is more in their attitude.

P4: Yes, exactly!

(Everyone agrees)

P3: They like to show that they are smart, in any possible ways.

Reference 2 - 0.46% Coverage

P6: I was totally puzzled as I was trying to do something like this. Students in mathematic class tell that they know everything but then secure only 2 in exams! What might have that child understood?

P4: (Not clear)

P6: I think the questions must be in a way so 3that the options would not be that different so that if the child is to select the answer, s/he must feel like s/he is feeling exactly the same!

Reference 3 - 0.17% Coverage

P7: I think this is what the purpose of risk calculator is!

P6: Yes!

P4: It is to be used by self, isn't it?

P7: The result is known to them only…

Reference 4 - 0.34% Coverage

P3: In this tool, are the personal information of individual shared?

I: No, it does not! It is very confidential and only the individual who is going to fill it up will know the details and know where to go or what to do.

P6: If the personal information are not shared then adolescents might fill it up.

Reference 5 - 0.20% Coverage

P6: I don't think going through teachers would be a good idea. The gap between the teacher and the student will always be there so we should not go through it (Everyone agrees)…

Reference 6 - 1.21% Coverage

P4: What I think is… sorry I interrupted here…As I am also working in eHealth at the moment, at the beginning, during the identifying phase…there will obviously a need of backups and all…but at the beginning…we cannot choose to go through the help of teachers or even the family. We, organization, can orient students keeping them together and ask them to fill the form. We also should ensure that the information will be confidential. But even though the information will be confidential, there should be at least some information as a output because, in server, there might be some information about which mobile has been used to fill up the data or something like this. If we don't find any problem among the adolescents, it is fine. But if we find some cases of high risk among the group, then there should be a medium from where we can reach out to the individual. In the severe case of depression, you might also know is clearly mam (Indicating to P7), suicidal ideation and thoughts have been seen more. So, we must have a pathway to reach out to the individual in such a case.

Reference 7 - 1.08% Coverage

P3: The organization can go to some school and take a class to orient students about the tool. And if we have targeted the class already, then I don't think we should be writing the age group again here, because we almost know their age. We should not keep any identifying information of them. If we do so, then we can get some kind of right information. If we tell them that no any identifying information of them are there and we are just doing a survey, then I think it will be a good idea. If they don't have to give any of their information which would help them to be identified, then they will think properly and fill up the form. We can choose to play some game with them to make it more interesting. And at last of the game, we can introduce the tool in front of them. If we directly introduce the tool then I don't think it will be better, so we can choose some other alternative like taking some other programs and at last introducing the tool to them.

Reference 8 - 0.27% Coverage

P4: From my experience, what I feel and I am sure is, they will fill this up. What sir (Indicating P6) did was, he gave students the questionnaire, and students already knew him and it is the reason they didn't fill the questionnaire properly.

Reference 9 - 0.67% Coverage

P4: …and talking about today's condition, depression among adolescents are supposed to be very high…because the prevalence is really high. So, there should be some medium to find them out. The only thing is, the questionnaire should not be introduced to them by any person who is familiar with the adolescents. If any person who the adolescent don't know is the one to introduce this tool then…it is very easy for us to share things with the person we don't know…we even can take medicine in their prescription then sharing is also very easy with the person who is not familiar with us... (Laughing)

Reference 10 - 0.21% Coverage

P6: Changing the venue; not doing this in school could also be another good option…

P4: Yes, exactly!

P6: We should change the venue as well, because the adolescents will feel free then…

<Files\\KII\\ > - § 25 references coded [13.97% Coverage]

Reference 1 - 0.61% Coverage

Yes, we can prevent the negative impacts of depression. It would be better if we make such tools but we have to look at its validity and feasibility. For example, we have to know what to use in context of rural as well as urban area as the daily functioning of the people in these two different settings are different. We have to focus on its validation and also focus more on this, it is not that easy to develop and use the risk calculator.

Reference 2 - 0.36% Coverage

P: You have included most of the questions and I think it will cover what you are going to find out. There are few things that should be modified such as the question pattern and the sequence or about adding few more questions to cover risk. Otherwise, it is fine.

Reference 3 - 1.14% Coverage

Most of the adolescents now are going to school and only few are not. So, if we let them fill up the form by themselves then it would be good. If we tell them that this is confidential then they will be able to fill it up properly. If we ask them all these questions, they won't respond properly so it is better to ask them to fill it up by themselves. And if you use mobile application for this as well, it won't be a problem. This generation is very forward in using smartphones so we can use through that as well. The first time I saw this tool, I thought it was made for mobile application. If we want to do a big survey, we could go through that model as well but may be you don't need to do that all. Still, you can think of using mobile phones for this. It would be beneficial. The other option is self-administration.

Reference 4 - 0.50% Coverage

For now, as per the question, adolescents are the one who would be benefited. But, of we make few changes in language, it would be fitted for everyone. The factors that trigger depression varies according to the age

so if we make few changes as per required, this tool would benefit all other people. But for now, I see it more relevant and focused for adolescents.

Reference 5 - 1.31% Coverage

It is better if the targeted one fills up these questionnaires but if someone on behalf of them should fill up these then it would be difficult I think. Things will come from assumptions if someone else fill up these questionnaires on behalf of adolescents. Sometimes, may be some of the information could come because parents or teachers may know few things that adolescents go through but exact information won't come. If I am the one who feel it, then only I can say it the best. Adolescents shares only few things with others. If there is a best friend of that adolescent then s/he can administrate that adolescent but teachers, health workers or parents won't be able to. I have seen people visiting Primary health Care Centre (PHCC) for consultation with psychiatrists but not in other health facilities. So, the health worker may not know the details of the adolescents to fill it up in their behalf so as the teachers. It will be hypothetical.

Reference 6 - 0.20% Coverage

If we have to administer every triggering factor here in the form, then it won't be effective if others except the targeted one fills up the form.

Reference 7 - 0.99% Coverage

We have to think about confidentiality as well because if there is another person beside them then they can't open up and administer all of their feelings. It could be interpreted in a wrong manner. It has both risk as well as benefit. Till the time adolescents are filling up the form, they will feel nothing but when the results pop up, there is equal chances of them being positive and negative. You have mentioned about the things that could be done for prevention so may be adolescents will take it positively or maybe they could not! But if we mention that depression is not that major problem and it could be prevented and cured if we follow some of the measures then may be adolescents could be calmed down.

Reference 8 - 0.35% Coverage

We have to mention such information for those with high risk. If one suddenly finds out that s/he is in high risk of depression then they might get shocked, so, to normalize the situation, we have to put such kind of information for those with high risk.

Reference 9 - 0.43% Coverage

Yes, of course. Otherwise they will not fill the importance of filling the form. So, in the result section, there should be such information that could calm down adolescents with high risk of depression. It could benefit them. If we are doing this right now, there could be at least a researcher by their side…

Reference 10 - 0.68% Coverage

I mean, there could be a researcher who could give this form to the adolescents and ask them to fill up the form and when the result will pop out, then that researcher could normalize the situation. But if the adolescent is alone and filling out the form, there is equal chance of them reacting positive as well as negative after seeing the result. There could be risk or it could benefit them. For minimizing the risk, we could add some more information in the high risk section of result.

Reference 11 - 0.49% Coverage

If we are targeting adolescents for this and going among the random population it won't be any challenging. If we were to use this tool among the depressed adolescents then may be if would be difficult for us to find the ones who are depressed but as we are targeting random population who are adolescents and not others so, I don't see any challenge here.

Reference 12 - 0.26% Coverage

We have to take consent from the parents of adolescents with whom we are doing this and also make them aware about the fact that this is not something that one should be stressed about.

Reference 13 - 0.61% Coverage

Yes! To know the risk is something that could benefit both adolescents and their parents. We have to take consent from the adolescents as well as their parents and there could be some risk factors from among the parents too. We have to provide them the service if needed as this is in our ethics as well. If the depression case is suicidal then we have to inform their parents, else the consent from adolescent will work for doing these all.

Reference 14 - 0.54% Coverage

P: Result is written in two paragraphs there where the result of their risk is written in the first one and the things s/he could do further is written in the 2nd one. So now, what we can do is, keep the first paragraph as it is and add things to calm down and normal the adolescent in the a different paragraph as keep it below the result and at last keep the third paragraph of it as it is.

Reference 15 - 0.87% Coverage

P: We could write something like this; depression could happen to anyone and it is most prevalent in this age group (Referring to adolescents) or some other age group whichever is the fact, depression could be cured. So if we write something like this then it could help the person normalize the situation. Seeing the result of high risk of depression suddenly, the person might get more curious and after reading such kind of information then s/he could be calmed down and the situation will be normal. Talking about the result of low risk, the information you have put is enough, there won't be anything that could be added.

Reference 16 - 0.20% Coverage

P: Most of the adolescents now are going to school and only few are not. So, if we let them fill up the form by themselves then it would be good.

Reference 17 - 0.82% Coverage

If we ask them all these questions, they won't respond properly so it is better to ask them to fill it up by themselves. And if you use mobile application for this as well, it won't be a problem. This generation is very forward in using smartphones so we can use through that as well. The first time I saw this tool, I thought it was made for mobile application. If we want to do a big survey, we could go through that model as well but may be you don't need to do that all. Still, you can think of using mobile phones for this. It would be beneficial. The other option is self-administration.

Reference 18 - 0.21% Coverage

P: For now, as per the question, adolescents are the one who would be benefited. But, if we make few changes in language, it would be fitted for everyone.

Reference 19 - 0.09% Coverage

But for now, I see it more relevant and focused for adolescents.

Reference 20 - 0.07% Coverage

I: So, it helps adolescents?

P: Yes, definitely!

Reference 21 - 1.32% Coverage

P: It is better if the targeted one fills up these questionnaires but if someone on behalf of them should fill up these then it would be difficult I think. Things will come from assumptions if someone else fill up these questionnaires on behalf of adolescents. Sometimes, may be some of the information could come because parents or teachers may know few things that adolescents go through but exact information won't come. If I am the one who feel it, then only I can say it the best. Adolescents shares only few things with others. If there is a best friend of that adolescent then s/he can administrate that adolescent but teachers, health workers or parents won't be able to. I have seen people visiting Primary health Care Centre (PHCC) for consultation with psychiatrists but not in other health facilities. So, the health worker may not know the details of the adolescents to fill it up in their behalf so as the teachers. It will be hypothetical.

Reference 22 - 0.21% Coverage

P: If we have to administer every triggering factor here in the form, then it won't be effective if others except the targeted one fills up the form.

Reference 23 - 0.53% Coverage

I: That means, we can display the result in front of adolescents?

P: Yes, of course. Otherwise they will not feel the importance of filling the form. So, in the result section, there should be such information that could calm down adolescents with high risk of depression. It could benefit them. If we are doing this right now, there could be at least a researcher by their side…

Reference 24 - 0.68% Coverage

P: I mean, there could be a researcher who could give this form to the adolescents and ask them to fill up the form and when the result will pop out, then that researcher could normalize the situation. But if the

adolescent is alone and filling out the form, there is equal chance of them reacting positive as well as negative after seeing the result. There could be risk or it could benefit them. For minimizing the risk, we could add some more information in the high risk section of result.

Reference 25 - 0.50% Coverage

P: If we are targeting adolescents for this and going among the random population it won't be any challenging. If we were to use this tool among the depressed adolescents then may be if would be difficult for us to find the ones who are depressed but as we are targeting random population who are adolescents and not others so, I don't see any challenge here.

<Files\\KII\\ > - § 2 references coded [1.15% Coverage]

Reference 1 - 0.44% Coverage

P: I think target group and adolescent age group…if adolescents are focused for research then they could calculate the risk for quality data.

Reference 2 - 0.71% Coverage

P: The risk could be about the type of tool,…. whether it is electronic or paper wise or something else. Obviously, everything has its own risk or bias. We need to focus on whether the tool is being used by real user or other.

<Files\\KII\\ > - § 24 references coded [17.45% Coverage]

Reference 1 - 1.60% Coverage

Now we will talk about the risk calculator. I think you are also aware about the risk calculator, each country have their own, and if we develop a risk calculator, with all the signs and symptoms of depression in it, who would be capable of using the risk calculator?

P: I think… as you talked about the capable personnel who is a specialist and also about a lay person, so what exactly is the context?

I: If we develop a risk calculator, then who would be capable of using the tool? Which sector would use the tool effectively?

P: I think…

I: Does that require a health personnel or a lay person can also use the risk calculator?

P: As I have been teaching in colleges and as per my understanding, I think we have to include parents, teachers as well as the health workers while developing the risk calculator. And if we do so, everyone can be able to identify the cases of depression and can refer the cases to the health facility. As you asked, if only the health service providers are capable of using the tool, the one involved in health sector are more capable of doing this whereas the others such as physiatrists and psychologists are also capable of doing so. So, not only health related personnel, it would be better if both health and psychology are taken together for this.

Reference 2 - 0.66% Coverage

In this, we have mentioned about prevention. What I think is, there needs to be information added on what person can do at his personal level for the prevention of symptoms of depression. Therefore, if information's are mentioned under different categories like what can be done at personal level, what can be done at family level and what can be done at community level, then I think that will be better, from my own preference. It says about symptoms (umm), symptoms of depression, prevention, treatment (respondent is reading information)

Reference 3 - 0.47% Coverage

Yeah I have understood this. And, I think this is important for all three categories: low, moderate and high. Under many circumstances, when person undergoes treatment then there are many approaches where person can help himself like psycho counselling, psychotherapy, family therapy. These things can be added in formation box as well. I think other information's included are ok.

Reference 4 - 0.34% Coverage

(reading information box) Sound sleep, balanced diet and additional information on behavior. (ummm) Sound sleep, balanced diet, behavioral information (pause). I think we need to add few information on different techniques through which positive thoughts can be generated.

Reference 5 - 0.42% Coverage

Not only in low but also in high. Both categories. Generally, among those suffered from depression, we encounter pattern of negative thoughts. These negative thoughts results in negative attitude. So, it will be helpful if we mention about different techniques that help to minimize negative thoughts or divert one's thoughts from negativity.

Reference 6 - 0.54% Coverage

How will we access in those unreached areas?

I: How do you think this is possible? At present, we are thinking about its usability via app and increasing its accessibility. What do you think can be done for betterment?

P: What I think is: we can develop this tool through mobile app and make its usability in offline mode when once installed and at least people having access to this can know about their status. One way could be this.

Reference 7 - 0.98% Coverage

We have primarily developed this for adolescent but what do you think about its user? Who else could use this? Talking about adolescent they could be literate/ illiterate and can reside in urban vs. rural. So, what do you think who can make use of this? Is this only adolescent or parents/ guardians/ teachers on behalf of them? Please explain in brief.

P: I really like this question which you have asked. I think this can be effective if filled by person suffering from depression. Under many circumstances, we will be observing symptoms prevalent in person but it will be seen objectively. It cannot come through subjectively. In addition, what I want to link this with is, for the sake of information purpose parents/ teachers/ personnel working in health post can also make use of this.

Reference 8 - 0.75% Coverage

From my point of view, for the sake of information purpose I think anyone can benefit from this but I prefer this tool be filled by person suffering or likely to suffer from depression. And, I like the way you have created separate information box which contain information on help seeking and redirecting to different useful links for further tips. In redirect pages, we can find useful information that has relevant information on symptoms of depression, treatment modalities, medication, counseling, psychotherapy, role of family members which will definitely help people associated with sufferer as well.

Reference 9 - 0.37% Coverage

By filling this tool on behalf of adolescent, something can be different. For eg there is difference observed when we visit any health facility to examine the problem by ourselves and ask our parents/ guardians to bring medicines relating to our problem. Obviously, something difference will be seen.

Reference 10 - 1.09% Coverage

So, do you think same set of tool can be used with everyone?

P: No, what I want say is slight difference will definitely show up. Perspectives wise it could be varied among different people. I don't mean to say all set of same questionnaires could be used but few changes can be made when viewing from teacher's side or others. For eg. with teacher we can explore about his perspective on: student actively interrogating with friends previously in classroom but not now, student focusing on his studies previously but negligence shown recently; with guardians/ parents about active or passive involvement in assisting with household chores, being more arguable recently. Therefore, similar responses can be observed for many questions but it can be helpful if we add few questions depending on the type of category we wish to talk to like parents/ guardians, school and community.

Reference 11 - 0.26% Coverage

I: And what about challenges that is associated with its use?

P: One same person can use this tool multiple times. Ok?

I: ummm

P: Is there any alternative to electricity when it goes off while filling this?

Reference 12 - 0.48% Coverage

This is one kind of survey, public opinion. In future, for good cause, at government's policy level also we can discuss about use of this app and its process and how this can be incorporated in any kind of decision making at government's level. Talking further about obstacles, how can this be useful to illiterate one? This will not be useful in those areas where there is no electricity?

Reference 13 - 1.00% Coverage

As said previously, there is trend where we fill tool through verbal communication. The way person expresses himself through self-administration technique differs from the way he expresses to any person who asks him questions. Up to some extent it is different. The most effective way is through self-administration technique where we will be able to measure what we want to and it gives concrete output. But when mediator or someone else plays role of middle person, slight difference will be seen. A little difference will be there. That could be one challenge and this is what I feel. Let us try to observe from our side and see how this work in upcoming days and how this challenge can be minimized. We need to work on how we can make this tool be easily usable among both literate/ illiterate or rich/ poor.

Reference 14 - 1.41% Coverage

Agreed. You have also linked already. Instead of revealing the result directly, when one finishes filling the tool we can ask him about how he would like to predict the result based on his experience. We can follow step of neutralizing for cognitive restructuring in gradual manner before result is revealed and when he comes at acceptance level then we can reveal the result. This is for individual level. But as you have also stated previously, in community like ours people generally have negative attitudes towards mental health problems or mental illness. Therefore, I don't think this needs to be revealed to others. Like, if teachers know about this then he could say this person is like this and that. If parents/ guardians come to know then they will also start to say this and that. This also can happen in the community. Therefore, this should be done from individual level. In addition to what you said, in developed nations, this is quite different. The way they perceive about this issue is quite different from ours. But, therein also there is something like privacy of person. Herein also, I think we should maintain the privacy.

Reference 15 - 0.31% Coverage

What we can do here is, we can mention in the last that if person is feeling very difficult then for treatment or additional services he can seek services from following referral centers located in each province or other. This way it could be easier.

Reference 16 - 0.43% Coverage

Yes. Or after giving information to him then he can go to seek services from service providers from different centers on his own. In condition where parents/ guardians needs to be called upon, service provider could ask with the respondent about who he feels comfortable to talk or share his problem with. I think we can go through that method as well.

Reference 17 - 0.77% Coverage

There is no doubt in making using of the tool from any corner. As said by you, this can be done at school level, at community level. There is no problem but we should be concerned about the selection of setting from where the person will be able to share his problem effectively. More appropriate, to start with, we can go through child clubs or youth clubs where we can find homogenous group of people making common sharing. Talking about priorities, after going through this approach, I think it can be done either in school or college setting!! I feel this way. Or for convenience, it can also be done from health setting.

Reference 18 - 0.27% Coverage

P: What I think is: we can develop this tool through mobile app and make its usability in offline mode when once installed and at least people having access to this can know about their status. One way could be this.

Reference 19 - 0.54% Coverage

P: I really like this question which you have asked. I think this can be effective if filled by person suffering from depression. Under many circumstances, we will be observing symptoms prevalent in person but it will be seen objectively. It cannot come through subjectively. In addition, what I want to link this with is, for the sake of information purpose parents/ teachers/ personnel working in health post can also make use of this.

Reference 20 - 0.58% Coverage

I: As an adolescent one can fill this tool but do you think same set of questionnaires can be filled by parents/ teachers to represent condition of an adolescent?

P: By filling this tool on behalf of adolescent, something can be different. For eg there is difference observed when we visit any health facility to examine the problem by ourselves and ask our parents/ guardians to bring medicines relating to our problem. Obviously, something difference will be seen.

Reference 21 - 1.01% Coverage

P: No, what I want say is slight difference will definitely show up. Perspectives wise it could be varied among different people. I don't mean to say all set of same questionnaires could be used but few changes can be made when viewing from teacher's side or others. For eg. with teacher we can explore about his perspective on: student actively interrogating with friends previously in classroom but not now, student focusing on his studies previously but negligence shown recently; with guardians/ parents about active or passive involvement in assisting with household chores, being more arguable recently. Therefore, similar responses can be observed for many questions but it can be helpful if we add few questions depending on the type of category we wish to talk to like parents/ guardians, school and community.

Reference 22 - 1.01% Coverage

P: As said previously, there is trend where we fill tool through verbal communication. The way person expresses himself through self-administration technique differs from the way he expresses to any person who asks him questions. Up to some extent it is different. The most effective way is through self-administration technique where we will be able to measure what we want to and it gives concrete output.

But when mediator or someone else plays role of middle person, slight difference will be seen. A little difference will be there. That could be one challenge and this is what I feel. Let us try to observe from our side and see how this work in upcoming days and how this challenge can be minimized. We need to work on how we can make this tool be easily usable among both literate/ illiterate or rich/ poor.

Reference 23 – 1.41% Coverage

P: Agreed. You have also linked already. Instead of revealing the result directly, when one finishes filling the tool we can ask him about how he would like to predict the result based on his experience. We can follow step of neutralizing for cognitive restructuring in gradual manner before result is revealed and when he comes at acceptance level then we can reveal the result. This is for individual level. But as you have also stated previously, in community like ours people generally have negative attitudes towards mental health problems or mental illness. Therefore, I don't think this needs to be revealed to others. Like, if teachers know about this then he could say this person is like this and that. If parents/ guardians come to know then they will also start to say this and that. This also can happen in the community. Therefore, this should be done from individual level. In addition to what you said, in developed nations, this is quite different. The way they perceive about this issue is quite different from ours. But, therein also there is something like privacy of person. Herein also, I think we should maintain the privacy.

Reference 24 – 0.77% Coverage

P: There is no doubt in making using of the tool from any corner. As said by you, this can be done at school level, at community level. There is no problem but we should be concerned about the selection of setting from where the person will be able to share his problem effectively. More appropriate, to start with, we can go through child clubs or youth clubs where we can find homogenous group of people making common sharing. Talking about priorities, after going through this approach, I think it can be done either in school or college setting!! I feel this way. Or for convenience, it can also be done from health setting.

<Files\\KII\\ > – § 15 references coded [12.61% Coverage]

Reference 1 – 1.06% Coverage

: Ok, now let's move to our next question which is about the risk calculator. Every country has its own risk calculator, so, if we develop risk calculator in context of our country having list of all the signs and symptoms of depression, who would be best equipped to use such tool effectively?

P: If we develop the tools?

I: Yes, if we develop the risk calculator…

P: Including all the signs and symptoms?

I: Yes.

P: If so, adolescents themselves can use this. Also the parents of adolescents and major people in the community can also use that. The people we discussed about previously could use that and it includes people like us, different psychosocial counsellors and the personnel in the field of psychology as well as health workers can use the tool.

Reference 2 – 0.15% Coverage

I: That means service provider could use it to identify depression properly and it would be beneficial?

P: Yes.

Reference 3 – 0.37% Coverage

P: (Commenting on the questionnaire) Along with *Nishan*, *Chinnaharu* (Both Nepali words used to indicate physical mark)… is this questionnaire self-administered or we go and ask them (Adolescents) about it?

I: What could be best? You can give feedback on it as well.

Reference 4 – 1.57% Coverage

I: Ok. We have targeted adolescents for this. But what could happen if their parents or teachers could fill up this form on behalf of them? How effective would it be?

P: Multiple information is better. It is better to take information of someone from multiple people but then it is necessary for adolescents themselves to fill out this as there are lots of things that they have faced, there are many subjective experiences. I think so.

I: If this form is filled by their parents, do you think there may be lack of information or do you think it would be more effective?

P: It happens. Even in our experience, if we fill up the rating scales from only one respondent then we lack lots of information. So we collect multiple information as far as possible during our assessment, we don't just collect information from one source. We send the same form to teachers and parents, though the versions are a bit different.

I: So, if we give this kind of form to adolescents then we should give the same kind of form to their parents or teachers to collect multiple information?

P: If possible, it is better to collect multiple information.

Reference 5 - 1.36% Coverage

I: You said that you use different version of forms to different respondents. So, are the questionnaire different or…?

P: The possible answers are same but the questionnaire is different. For example, if we give the form to teacher then there are questions about the classroom behaviours of that student or adolescent which is observed by that teacher and for the parents, we give them questionnaire where the home environment are focused while personal information are focused among the adolescents.

I: You link up all of them and give it to them so that the information…

P: And we go for the adolescents for the conclusion at last and use our clinical judgement.

I: Tools you were using till now were screening tools and this one we are about to use is risk prediction tool. So, do you think we should use this in the same modality?

P: If possible, you can do that because it is better. I don't know what do you think but I think it is better.

I: Yes.

P: Yes, it is my view.

Reference 6 - 2.40% Coverage

I2: As we said that it is just a prototype tool that is targeted for adolescents. So, would it be better if we display the result in front of adolescent after they have completed filling the form or should we keep the result confidential where only experts will know it and will consult about it with that adolescent's parents? What could be done for confidentiality? As you said, some may be sensitive and some may perceive such things in a positive way. What do you think could be done to make it in a better way?

P: What age group have you defined as adolescents here in your study?

I2: We have defined 10 to 24 years for adolescents in our study…

P: You have extended it up to 24 years (Laughing)?

I: It is for our project but…

I2: The age group for adolescents is usually up to 19 years.

P: Yes, some have defined it from 9 years, 11 or 12 years…

I2: So, what do you think what could be the defined age group for adolescents? For now, we have kept it as open ended question.

P: It must be understandable for the adolescents if we are giving them these self-administered questions. The way a 9 years old understands and 12 years old understands is different. So, I think, age group from 12 years is fine.

I2: So, it won't be a problem if we use self-administered questionnaires?

P: Yes. And like you said, about disseminating the result to them…it is their right to know about it so we can take consent with parents for displaying results to the individuals/adolescents. For adolescents till the age of 18, we have to take consents from parents for this. First of all, we get to know about it and then we let their parents know this. Then what we can do is, we can ask their parents' and let the adolescents know the result.

Reference 7 - 0.58% Coverage

P: There is also the question asking adolescent that if or not they have left home and ran away somewhere… so, there is a question about the scarcity of food along with the part of clothes, there comes two question alongside but the focus seems in the part of clothes only…

I: So, it is better if we keep both of them as an individual question?

P: Yes but if not, the consequences of both the aspects should be shown.

Reference 8 - 0.52% Coverage

P: Talking about the question where it is asked that if your parents or caregiver ever wished you were never born…it should be rephrased…you will do it right? Instead of *Aasha*, you can write *Apekchya* (Different words used in Nepali for wish).

I: Ok.

P: It is relevant. Adolescents sometimes say "you could have not given birth to me" or "I wish I was never born" kind of things…

Reference 9 - 1.34% Coverage

P: Multiple information is better. It is better to take information of someone from multiple people but then it is necessary for adolescents themselves to fill out this as there are lots of things that they have faced, there are many subjective experiences. I think so.

I: If this form is filled by their parents, do you think there may be lack of information or do you think it would be more effective?

P: It happens. Even in our experience, if we fill up the rating scales from only one respondent then we lack lots of information. So we collect multiple information as far as possible during our assessment, we don't just collect information from one source. We send the same form to teachers and parents, though the versions are a bit different.

I: So, if we give this kind of form to adolescents then we should give the same kind of form to their parents or teachers to collect multiple information?

P: If possible, it is better to collect multiple information.

Reference 10 - 0.69% Coverage

I: You said that you use different version of forms to different respondents. So, are the questionnaire different or…?

P: The possible answers are same but the questionnaire is different. For example, if we give the form to teacher then there are questions about the classroom behaviours of that student or adolescent which is observed by that teacher and for the parents, we give them questionnaire where the home environment are focused while personal information are focused among the adolescents.

Reference 11 - 0.23% Coverage

I: You link up all of them and give it to them so that the information…

P: And we go for the adolescents for the conclusion at last and use our clinical judgement.

Reference 12 - 0.39% Coverage

I: Tools you were using till now were screening tools and this one we are about to use is risk prediction tool. So, do you think we should use this in the same modality?

P: If possible, you can do that because it is better. I don't know what do you think but I think it is better.

Reference 13 - 1.14% Coverage

P: If we look at it positively, we can say that they will be more conscious; they could think about the risk factors and also think about the ways of minimizing them. And again, not everyone's perceptive is same. If someone is anxious then they might get scared after knowing that they are at high risk of depression. They might think about it every time, get scared and may stopped working. There are positive as well as negative parts.

I: What can we do to minimize or reduce such challenge?

P: For reducing this, we have to give them clear statement saying that it is only about the risk factor assessment and it is not sure that you will have depression and also say them that if they want they can go to the experts for preventive measures.

I: So, while displaying result, we have to tell them such things?

P: yes.

Reference 14 - 0.50% Coverage

I2: So, what do you think what could be the defined age group for adolescents? For now, we have kept it as open ended question.

P: It must be understandable for the adolescents if we are giving them these self-administered questions. The way a 9 years old understands and 12 years old understands is different. So, I think, age group from 12 years is fine.

Reference 15 - 0.33% Coverage

I2: Because it could help in prevention, it is better to perceive this in a positive way, isn't it?

P: Yes, exactly. We should not hide any kind of information just because of stigma. We have to share the information as much as we can.

<Files\\KII\\ > - § 22 references coded [22.04% Coverage]

Reference 1 - 2.31% Coverage

I: So, you have looked at the questionnaire. Who could be able to use this tool? For now, it is targeted for adolescents that means, adolescents themselves can fill up this form or some researchers could ask them the questions and get this done. So, would it be effective if parents/guardian or health workers or teachers fill up this form in behalf of their adolescents?

P: If health workers or parents are the ones to fill of the form then we have to add "don't know" as the option for most of the questions. They may not know most of the things adolescents feel. So, there must be options such as "doesn't apply" or "I don't know".

I: If teachers or someone other than adolescents themselves fill up the form then we should add such options?

P: Yes!

I: If we add such options, then would it effective? Should they fill up the form? Or should this form be filled out only by the adolescents as the target group is adolescent?

P: We can't say this without doing research. If we once go in the filed with this question using this to parents, then at least we can say that we could get some kind of information from parents even this tool is developed targeted to adolescents. Or we can see the information given by adolescents and parents and find

out the correlation and say whether or not it is acceptable. We have to change the weightage of each questions accordingly because the way adolescents fill up the form may not be correlated to that of their parents; parents may not know about abuse that had happened with their children or they may not know that their children had fight with someone and they may so no at this point. We could adapt the data accordingly.

I: So, other could also fill up this form on behalf of adolescents?

P: For now, not really!

I: (Laughs) if research shows that then…

P: Doubtful! It could be extended but I won't be able to say that others could fill up the form on behalf of adolescents.

Reference 2 - 0.37% Coverage

I: We have to look at how an adolescent would perceive the result.

P: Yes, it depends on that as well!

I: What could be done for that then?

P: What we can do is, put an option of high risk and include those who are at very high risk of depression but then form other options for mild or moderate as well.

Reference 3 - 0.65% Coverage

I: Can we also choose not to reveal the result with the respondent but tell them to follow some intervention approaches if they are at high risk of depression? In such case only the person who has asked the adolescent to fill up the form will know about the result.

P: Instead of the result of high risk, we can directly mention some intervention for them and tell them that they might be at risk of depression so they have to follow those approaches. Because we are not sure that s/he would surely have depression even if they are at high risk.

Reference 4 - 1.31% Coverage

I: So, we can write some of the ways to prevent depression there?

P: Yes. Sometimes, while trying to prevent, the problem might increase. For example, during and after earthquake in our country, we thought people would be having mental stress and that's why everyone were running here and there for providing services or seeking services, but instead people were more resilient to that incident. So, we have to bring the needy ones as well as focus on the natural healing process and

normalize people. For that…what have been other sing as the result, is that the same like we are using; high and low risk?

I: … (Trying to speak but respondent continued)

P: In physical illness, it is fine. But in case of mental illness, may be the words have to be changed in some ways. We can say some ways for preventing them from getting depression if we found out that they are at high risk of depression. Instead of high risk and low risk, maybe we can find out other alternatives for making it more constructive.

I: This is for both adolescents and their parents/care givers?

P: Yes, for all of them.

Reference 5 - 6.58% Coverage

I: And as you said, for those with high risk, maybe we could provide some information about some interventions for preventing mental health problems and maintaining mentally healthy lifestyles.

P: Yes. According to me, I think its utility could be in school mental health programs where the results could be known by the professionals or the researchers only but still we could provide some kind of general information to every one once they fill out the form. This could be in the case where they might not get to see the result by themselves.

I: Can we do so?

P: Yes!

I: Is there something in research ethics where it says we should reveal the result to the participant or something like that?

P: We could tell them to ask individually and know the result if they want. But we have to tell the generic message to all of them. We can reveal results immediately to those who have access to services near them. For example, if someone is in high risk and they have school counsellor in their school then we can tell them that they are at high risk of depression so that they could immediately go to school counsellor for counselling if needed but where people don't have access to such facilities and yet know the result, they might get scared and might have negative consequences in them. So, for them, we can provide some generic information about mentally healthy life style and preventive measures of depression instead of directly showing them their result of risk. Just imagine, if someone tell you that you are at a high risk of depression then what would be your reaction? How would you feel? You would obviously feel stressed (Laughs) but those who have access to services could be calmed down and might not be a victim of negative consequences. We might also get some cases of high risk of depression where we could look from the parent's or teacher's perceptive and know if they are at risk, because obviously very high risk of depression could be seen from other's view as well. And for the other general population, teachers or nurses or other person could use this tool up on them and know the result but don't reveal it to the adolescent and start the intervention where needed and instead provide generic feedback or message to every one so as to make them aware and prevent depression. Among 100 students at school, there might be 5 of the students who could be at risk but for those 5 we can't risk those 95 students' life because this is a very sensitive issue.

We have to choose some way to do this so that no one could be at risk of negative consequences of using this tool.

I: As you said, it can be used by teachers or nurses or others, so, could it be used by anyone as a researcher?

P: Yes!

I: So, it could be done but for revealing the result…

P: It have to be thought once again about revealing the result. If a person is at very high risk of depression, then researcher or counsellor or the elder one could find a way out for what should be done.

I: Suppose if I am a lay person, will I be able to use this risk prediction tool?

P: Yes, of course! After predicting the risk, the researcher should do according to their ethics. If 5% of the population has high risk of depression, then what should be done should be included in their protocol. There could be only 5% of adolescents who could be at high risk, or let's say 20% would be at high risk after we used this tool among them, but what about those 80% of adolescents who are at normal stressed but not at risk of depression? This is my only concern. For those with high risk as well, we have to tell them that it is normal so there is no any issue for you coming to seek help, we have to convince adolescents this way. There are many people who come to us after they use some self-diagnostic tool from internet and many of them are of anxiety disorder. Usually, students of MBBS and Nursing field feel that they have almost every problems after they study symptoms of each diseases from their course. Teachers of mental health also feels the same.

I: How do you think we could use this tool? Should we use it as a manual version or as you said, should we make it online and make accessible to all of the people?

P: Yes, we can do that as well!

I: Once we make it online, should we let the users know that they are at high risk of depression?

P: If we make this in such a way that one has to request for the result then it would be better I guess.

I: For the risk result?

P: Those who have chosen to fill up this form are those who have been worried about their health or those who have heard about it from different media. So, if we make it that way where one has to request for knowing their result, it would be better.

I: Ok. While showing them the result, we can give them some recommendations?

P: Yes. Everyone should get a generic message to make them aware but specific ones who are at high risk should additionally request for their result even if they get that generic message. We are not sure that the prediction really mean that they are going to have depression sooner or later. Among two of the children of one parent, one might say that s/he has been changed in a good way just because of that one slap from his/her parent but the other might be having problem after that slap. This is philosophical question. So, when we ourselves are not confirmed, why to make people panic? This is only my concern. Or else, we can use this in some specific place but hope that people won't misuse it.

Reference 6 – 2.23% Coverage

: Specific place?

I: So…

P: It should not be left useless once after it is developed.

I: So, the health service providers, people in school level and every other people can use this?

P: Yes! But it should be planned. We should not distribute this tool in a haphazard way.

I: So, if we need to prioritize the area for using this tool, which setting should we target? Where could be the applicability of this tool high?

P: If we are going to do any kind of intervention in school, we can use this tool as well or in the community as well.

I: Would it be useful in the clinical setting also?

P: In the clinical setting, people come once after they face the problem so…

I: Yes, but in some cases, I might go to the health facility because I might have doubt.

P: We can take history of that person and do the procedure then.

I: So, what do you think would be better in clinical setting; risk prediction tool or screening tool?

P: As people visit health facility after they face some kind of problem, so I think we could use screening tool in health facility.

I: Is it?

P: These items come in the case history (Risk factors). We take history because we get confused. So, this tool will be more useful in the community setting. These things which you are planning to include in the risk calculator must be asked during history taking process with the person who come here in our health institution. We have to ask some more details as these are not enough while taking history.

Reference 7 - 0.89% Coverage

P: We can't say this without doing research. If we once go in the filed with this question using this to parents, then at least we can say that we could get some kind of information from parents even this tool is developed targeted to adolescents. Or we can see the information given by adolescents and parents and find out the correlation and say whether or not it is acceptable. We have to change the weightage of each questions accordingly because the way adolescents fill up the form may not be correlated to that of their parents; parents may not know about abuse that had happened with their children or they may not know that their children had fight with someone and they may so no at this point. We could adapt the data accordingly.

Reference 8 - 0.14% Coverage

P: Doubtful! It could be extended but I won't be able to say that others could fill up the form on behalf of adolescents.

Reference 9 - 0.78% Coverage

P: For the high risk, we can put some information for healthy life styles and include some sites which could help them to be mentally healthy and for those with low risk as well, we can say them that they are fine but still mental health problem is such that anyone could get it and there's way to prevent it by following healthy life styles. We can keep such generic message for both of the category and add few more information for those with high risk. In TPO as well, you have some materials for psychosocial health. You can provide such materials to people and ask them to go through those materials and get informed about the mental health issues.

Reference 10 - 0.42% Coverage

P: Yes. According to me, I think its utility could be in school mental health programs where the results could be known by the professionals or the researchers only but still we could provide some kind of general information to every one once they fill out the form. This could be in the case where they might not get to see the result by themselves.

Reference 11 - 2.27% Coverage

P: We could tell them to ask individually and know the result if they want. But we have to tell the generic message to all of them. We can reveal results immediately to those who have access to services near them. For example, if someone is in high risk and they have school counsellor in their school then we can tell them that they are at high risk of depression so that they could immediately go to school counsellor for counselling if needed but where people don't have access to such facilities and yet know the result, they might get scared and might have negative consequences in them. So, for them, we can provide some generic information about mentally healthy life style and preventive measures of depression instead of directly showing them their result of risk. Just imagine, if someone tell you that you are at a high risk of depression then what would be your reaction? How would you feel? You would obviously feel stressed (Laughs) but those who have access to services could be calmed down and might not be a victim of negative consequences. We might also get some cases of high risk of depression where we could look from the parent's or teacher's perceptive and know if they are at risk, because obviously very high risk of depression could be seen from other's view as well. And for the other general population, teachers or nurses or other person could use this tool up on them and know the result but don't reveal it to the adolescent and start the intervention where needed and instead provide generic feedback or message to every one so as to make

them aware and prevent depression. Among 100 students at school, there might be 5 of the students who could be at risk but for those 5 we can't risk those 95 students' life because this is a very sensitive issue. We have to choose some way to do this so that no one could be at risk of negative consequences of using this tool.

Reference 12 - 0.24% Coverage

P: It have to be thought once again about revealing the result. If a person is at very high risk of depression, then researcher or counsellor or the elder one could find a way out for what should be done.

Reference 13 - 1.16% Coverage

P: Yes, of course! After predicting the risk, the researcher should do according to their ethics. If 5% of the population has high risk of depression, then what should be done should be included in their protocol. There could be only 5% of adolescents who could be at high risk, or let's say 20% would be at high risk after we used this tool among them, but what about those 80% of adolescents who are at normal stressed but not at risk of depression? This is my only concern. For those with high risk as well, we have to tell them that it is normal so there is no any issue for you coming to seek help, we have to convince adolescents this way. There are many people who come to us after they use some self-diagnostic tool from internet and many of them are of anxiety disorder. Usually, students of MBBS and Nursing field feel that they have almost every problems after they study symptoms of each diseases from their course. Teachers of mental health also feels the same.

Reference 14 - 0.23% Coverage

I: How do you think we could use this tool? Should we use it as a manual version or as you said, should we make it online and make accessible to all of the people?

P: Yes, we can do that as well!

Reference 15 - 0.84% Coverage

P: Yes. Everyone should get a generic message to make them aware but specific ones who are at high risk should additionally request for their result even if they get that generic message. We are not sure that the prediction really mean that they are going to have depression sooner or later. Among two of the children of one parent, one might say that s/he has been changed in a good way just because of that one slap from his/her parent but the other might be having problem after that slap. This is philosophical question. So,

when we ourselves are not confirmed, why to make people panic? This is only my concern. Or else, we can use this in some specific place but hope that people won't misuse it.

Reference 17 - 0.11% Coverage

P: Yes! But it should be planned. We should not distribute this tool in a haphazard way.

Reference 18 - 0.14% Coverage

P: If we are going to do any kind of intervention in school, we can use this tool as well or in the community as well.

Reference 19 - 0.20% Coverage

P: In the clinical setting, people come once after they face the problem so…

I: Yes, but in some cases, I might go to the health facility because I might have doubt.

Reference 20 - 0.08% Coverage

P: We can take history of that person and do the procedure then.

Reference 21 - 0.16% Coverage

P: As people visit health facility after they face some kind of problem, so I think we could use screening tool in health facility.

Reference 22 - 0.48% Coverage

P: These items come in the case history (Risk factors). We take history because we get confused. So, this tool will be more useful in the community setting. These things which you are planning to include in the risk calculator must be asked during history taking process with the person who come here in our health institution. We have to ask some more details as these are not enough while taking history.

<Files\\KII\\ > - § 17 references coded [17.40% Coverage]

Reference 1 - 2.16% Coverage

I: Ok, we will keep that in mind. Our major concern was the same. We wanted to know about the length of the questionnaire, acceptability of this kind of tools…

P: To develop such tools is nice, it is necessary but…

I: Yes, we only wanted to know if these kinds of questions are…

P: High risk and Low risk? What does that mean? It does not even make sense.

I: As it is just a prototype, we have just displayed the result instead of saying how it is calculated. Once the adolescent respond to each and every questions included here, the risk of depression is calculated and the result is displayed; showing if the adolescent is in high or low risk of depression. And then another screen will also show where such kind of information is provided to the adolescents if they are at high or low risk. We will later go through the process of its calculations and all but for now we just wanted to know if such told are made, then what will happen. Is it good or not?

P: It will be good but for that you have to put questions in one screen if possible. How many questions are there?

I: We have not counted the exact number of questions but…

I2: We have 3 to 4 questions or more under each sections.

P: It will be better if you put 6 to 7 questions in one screen because people will feel bored while doing “next” and “next” and s/he could drop out in the middle.

Reference 2 - 1.02% Coverage

I: Ok. I will ask you few things in short (Laughs). We have made this kind of tool for adolescents, but who others can use this? Could their parents or teachers fill up this tool on behalf of the adolescents?

P: It depends on the target group. If we have targeted adolescents, then they could complete this. It is difficult to use a single scale to multiple users because the way parents look at the situation is different than those of adolescents and the way teachers perceive could be different. So I think adolescent could be the one who could use this tool. And does that adolescent read the end result by themselves? How does it work?

Reference 3 - 2.26% Coverage

: If the adolescent is literate then s/he can just fill up the form by themselves and scroll it to end to read the result. And the next is, on behalf of adolescent we can just ask them the questions and fill out the form and read the result to them. It is done by research. And sometimes, if sometimes I as a parent or teacher use the tool on behalf of my children, then…

P: But it should be used by adolescents themselves.

I: Ok. It is targeted for adolescents so they themselves should use this. But if their parents or teachers fills out this on behalf of them…

P: What's the use of this tool? It is only useful to adolescent?

I: It is targeted for adolescent only for now because it is to be developed for identifying risk of depression among adolescents.

P: What will we suggest the adolescents after s/he completes filling out these questionnaires?

I: If the adolescent is in high risk then there is the information about treatment or intervention and about the referral services for them to use.

P: Do s/he refer themselves to the service providers?

I: Someone is there with them when they complete this set of questionnaire, so, what should be done? Is it better if we display the result to them or not?

P: We have to give them the choice by displaying the results to them.

I2: So, we have to display the end result with them?

P: Yes. S/he may or may not want to go for the treatment, it's their choice.

Reference 4 - 1.20% Coverage

: Should we reveal the result that the individual is at high risk of depression to that individual or should we keep this information confidential and consult with some other people, as there could be the issues of stigma or how would that individual or their family perceive the thing.

P: It depends on which settings we conduct this study. Is it (Not clear) or is it like I could download this tool and look at my risk of depression? It depends on the purpose if the study.

I: So, what could be done? Which could be effective? Could it be used manually, or could we use the electronic version?

P: We could give it to themselves so that they could look at their risk of depression by themselves. If they are at high risk then they will refer themselves.

Reference 5 - 0.56% Coverage

I: So, if we develop such tool, would it be beneficial?

P: Yes!

I: To every level?

P: Sorry?

I: Where could we use this tool as adolescents are engaged in different settings; home school; community…

P: Where the adolescent could confidentially complete these set of questionnaire, we could use it there.

I: Is it?

P: Yes. It should be confidential.

Reference 6 – 1.53% Coverage

I: So, we need not to have any specific setting or prioritize the settings?

P: We have to screen the adolescents and agreed by everyone. There could be different modality for using this in school or home or done by parents, teachers or adolescents themselves, referral by adolescent themselves or referral by someone else, taking consent with parents at first or teachers at first, these are different scenarios, how can we imagine all of them?

I: Yes (Laughs). What is your preference from all these?

P: As I said, it depends on the purpose. Everyone could make a scale for something. But one must already define what they are targeting, why they are targeting and all of the other things. *Yetiikai balchi haneko hoina ni ta* (You are not going without a plan). While preparing for a research, you already should have one research question and go accordingly. It's like, I will go to do something then…

I: Yes! For now, we have targeted adolescents for this.

Reference 7 – 1.73% Coverage

I: So what could be the feedback you could give us? You said it is beneficial for adolescents…

P: There is no any question about the benefits for adolescents with depression. But you should at least be clear about the questions like are you going to target adolescents for this or are you going to target researchers for screening adolescents, are you going to target teachers for screening adolescents, are you going to target parents to screen adolescents, in what settings you are going to use it, are you going to be anonyms or not, are you going to tell the result in front of the individual or not and if you get positive results then are you going to take consent from the parents about it or not? So, these things…what you are doing is, you are putting all these things alongside and thinking to go accordingly with whatever comes

on the way or from the answers of respondents. These are the things that should be pre-determined and according to that you should scale up and proceed. Do you understand what I mean?

I: Yes sir!

P: Your approach and my approach is completely opposite.

Reference 8 – 2.56% Coverage

: We have to incorporate everything you suggested us in the process of developing the tool. The last questions we guess we have is, what could be something which more information could be added in the risk calculator or what could be some changes that could be included here?

P: I have not looked at this clearly so could you just give that to me so that I could see and suggest you about it.

I: Okay!

P: I have to be aware about the research validity and other related things so that I could think about what could be included further. Without knowing all these and just seeing at the questionnaire, I would only be able to say that everything is good for now.

I: I would like to ask you a last question to you. If we identify an adolescent with high risk of depression then what could be some recommendation that we could give them?

P: Again, it depends on the purpose of the study you are doing. Are you doing this for research purpose, are you doing this for screening purpose or are you doing this for treatment purpose, these should be predefined before developing the any scales. But whatever the purpose is, if we find someone with high risk of depression, we need to find some way to recommend them to treatment. If we screen and find 50 people having depression, where would we send them for treatment? We don't have any place for the treatment. Are we going to screen people with depression and then tell them that you don't have any place to go for treatment? This is also a question. So generally, if we find that somebody have a high risk of depression then we need to find some ways to help.

Reference 9 – 1.14% Coverage

I: (Laughs) if someone is in high risk of depression, then it means that, s/he have the likelihood of having depression in certain years of his/her life but…

P: Yes, but what I am asking is do we screen that person for depression if s/he is in high risk of depression?

I: What could be done for such cases?

P: If someone is in high risk of depression then you should screen them and monitor as well.

I: Yes. And what about person with low risk?

P: What I would like to say is, you must first know how accurate is this kind of tool if it says that someone is in high risk of having depression? If someone is predicted to be in low risk of depression then that doesn't mean s/he is really at low risk of depression.

Reference 10 - 0.53% Coverage

P: In general, it is not that good to keep questions like this; few questions in one screen. You should have at least 7 to 8 questions in one screen because clicking "next" and "next" every time is boring. You are giving this to adolescents…nowadays, while making advertisements as well, they are said to make it less than 30 seconds.

Reference 11 - 0.18% Coverage

P: It will be good but for that you have to put questions in one screen if possible. How many questions are there?

Reference 12 - 0.26% Coverage

P: It will be better if you put 6 to 7 questions in one screen because people will feel bored while doing "next" and "next" and s/he could drop out in the middle.

Reference 13 - 0.68% Coverage

P: It depends on the target group. If we have targeted adolescents, then they could complete this. It is difficult to use a single scale to multiple users because the way parents look at the situation is different than those of adolescents and the way teachers perceive could be different. So I think adolescent could be the one who could use this tool. And does that adolescent read the end result by themselves? How does it work?

Reference 14 - 0.08% Coverage

P: But it should be used by adolescents themselves.

Reference 15 - 0.77% Coverage

P: It is not about stigma. Once it is identified that the person is at high risk then we should try to convince them to take interventions. If not once you have to tell them twice thrice or even more. If you know that somebody is at high risk but because of stigma, you can't hide it from them. What would you do if that person commit suicide in the coming days? We must not identify the risk on the first place, but if we are doing so, we have to tell them about their risk with them.

Reference 16 - 0.16% Coverage

P: Where the adolescent could confidentially complete these set of questionnaire, we could use it there.

Reference 17 - 0.58% Coverage

P: We have to screen the adolescents and agreed by everyone. There could be different modality for using this in school or home or done by parents, teachers or adolescents themselves, referral by adolescent themselves or referral by someone else, taking consent with parents at first or teachers at first, these are different scenarios, how can we imagine all of them?

<Files\\KII\\ > - § 4 references coded [3.27% Coverage]

Reference 1 - 1.22% Coverage

No-No, what you can do is…? You can see the current status of everything right now.

I: Current status.

P: Current status … Yah a little bit … you can instead look if the person is in normal range or little more than normal range of depression...Depression ... Isn't this about depression?

I: Yes.

P: Though they might not be depressed but if a bit different from normal then you are can start intervention. About thoughts…you can do intervention about repetitive bad thoughts. If there is fight at home, how it's going to bring… little bit about coping can be taught to child …things like that…educate the parents how

it's going to effect? … You are going to take interventions… such things … on the basis of this … But when will it reach up to people who don't have money. I don't think it will reach.

Reference 2 – 1.22% Coverage

So u know how children are ... So make it a … If you can then make it a mandatory thing… we should do that. Its good we want to see that there is no mental health problem. Because you … all the children came from difficult circumstances. We should talk in such a way that … I think that will be helpful … For parents relationship … There might...a lot of time when there is domestic violence in parents … Its I don't know ... hearing that its more anxiety related. Like if somebody is reading your two questions … may be his / her … Suppose if you go to village someone might have all the children as daughters only. And grandmother hates all of them … but if she have 5 granddaughters and hates all of them it doesn't mean they all will have depression. If it is so scarce source might be in that only.

Reference 3 – 0.39% Coverage

We can keep it as children in difficult circumstances level. So, the people take it…But yeah to see the family condition of a person, to know about him and how he feeling and all is like that, it could be helpful. It could be helpful for screening such things.

Reference 4 – 0.44% Coverage

Well this … let's not say on behalf. If they have a problem with their mother and the mother is given this form to fill, then it is obvious that the mother will say that her child doesn't have any problem with her. Also, you should make it in a language that other adolescents to understand.

<Files\\KII\\ > – § 6 references coded [11.14% Coverage]

Reference 1 – 0.56% Coverage

I: We want to get your feedback if this was practiced.

P: In our context, this is relatively new. So I feel that this will work with the things that it contains. Everyone has access to an android or an iOS version phones. So this will be accessible to most of the people in this country. While they take a look at it when they have internet access, I feel that it will be fruitful for them.

Reference 2 - 1.38% Coverage

I: What do you think about it? It is primarily prepared for the adolescents. Do you feel that it can only be used by the adolescents? Do you think that their parents and teachers should also get to use it? What do you think?

P: I think that it can be used by others as well.

I: The questions…

P: I think that it can be used for other adults apart from the adolescents, who know how to work with it and other people can use it through them as well. That is because we also get the information from it about how to move forward further for both the cases. A number of this information could be in Nepali or in English when we are referring to the website. We might have to work more on the languages so that it is accessible to more people. That could cause a barrier sometimes. Otherwise, this is effective. I also feel that it is a new technique in our context in my personal experience. Even I and the people of our generation are not familiar with it which is why...

Reference 3 - 2.09% Coverage

I: You looked at the questions briefly. And these questions were designed specifically for the adolescents.

P: Yes.

I: So do you think that their parents and teachers could help to predict their risks by filling it up for them? Or do you think that the adolescents need to be directly involved in it for the risk prediction?

P: It depends upon their level of education. We cannot assume that all the adolescents have completed their +2 level of education. So they might not be able to use it. There might be a number of them who are illiterate also. The literate ones could do this on their own. But some might require some help. We need to look at the condition that is there. The area in which we are trying to implement it like the schools could be aided by the teachers there.

I: You said that this could be answered by others because it is there to only reveal the risk that the adolescents are in. It identifies whether they are in high risk or low risk.

P: Yes. It could be useful for others as well. Adolescents are focused on this questionnaire. The questions could be made more general such that it could be used for everyone. When talking about the adults, maybe the aspect of their parents might not be as important. They might have become parents themselves. Their parents might be dependent on them. So such questions could be changed and the same tool that you have could be used for others as well. These questions are focused on the adolescents.

Reference 4 – 3.79% Coverage

: Yes. And what do you feel are its benefits? What could be the potential benefit?

P: One would be the confidentiality. The next would be regarding the self- awareness. Umm… and the high risk… You said that it calculates the risk of depression on its own, didn't you? So, they will be able to know where they are after filling it up on their own. We have information with us in case they seek for help. That is why it will be confidential as well. Umm… next, they will be able to take this if they have the internet access.

I: So you are saying that the questions need to be changed and anyone could use it so that it could be predicted. Do you feel that there are any challenges when using this tool?

P: I have mentioned the issue of language already. And then the other challenges… Thinking about it right now… One thing is that they need to have access to (smart) phones. In order to for it to function well, they need such phones. Only the people who have access to it can use it. There is the chance that it might not be as effective at larger areas sometimes where the risks are greater.

I: Do you mean the entire area?

P: there could be some depression- prone areas for the adolescents. It could also be in some emergency situations because of natural disasters. In such context, if it is not in access to everyone, then others tools could be effective rather than this.

I: At that time, it will show that everyone is at risk?

P: [Interrupting] But I do not think that there are any risks if they have the access. That is because these things stay confidential. If they want to seek help according to their decision because it is not yet

mandatory… umm… Next, all the things and all the instructions will come up as we might move forward… That I why… umm… I am not able to say it exactly right now.

I: You were saying that using it in the disaster- prone areas… I got a little confused by what you said. Are you saying that it will not be effective because everyone will be at risk there?

P: No, just the access. People need at least an android- version mobile phone if they want to use it. But in those areas…

I: It might not be available.

P: In our working area that we select, the number of adolescents could be high.

I: You mean to say that there has to be accessibility. People need to have information about it. They also need to be told that it is confidential.

P: Yes. But in such places, as we discussed before, there are different… Rather than asking them to fill it individually, there might be others at the school like the teachers who might have the android phones. But we need to assure them that these things are confidential.

Reference 5 - 0.26% Coverage

I: Do you think that this tool has to be used from a mobile app only? Or could we print this out and use it in hardcopy formats also?

P: Looking at this, I think that it can be done.

Reference 6 - 3.07% Coverage

I: All right. Is there anything else? It is said that the information for the high risk is this and the information given to the low risk is this. Do you feel that this much information is sufficient for you? Or do you think that the people at high risk need to be told some more things?

P: There is a phone number that has been put here. There are phone numbers and websites that have been mentioned here. These says, we could also add other links that might be there for social media if we have it. If there are toll free numbers then maybe it would make it easier (for patients). It might be easier at times if there are toll free numbers which do not cost anything to call. It would be easier if the contact numbers were separate for the men and the women and the children.

I: Ok. So we could make it gender- sensitive.

P: Yes, making it gender- sensitive would make it easier for them to talk to.

I: Right now, the results of the adolescents are revealed after they enter their answers. There are things like stigma that we need to consider when we reveal the results right there at the moment. They might learn that they are at high risk. What do you think is the possibility that this tool will play a negative role rather than a positive role?

P: That is possible. Two close friends might find that one is at high risk whereas the other is at low risk. But there has to be some awareness before this is carried out. There could be sensitization programs that are carried out where all these things are explained combining the issues of stigma also. We need to let people know that this is for our own well- being. They do not need to tell their friends about it if they do not want to. They should not pressure their friends when asking them about it. We need to be sensitive about these things. Such content should be there at those particular areas. Maybe those things need to be there. Then, they will understand its objective. They will learn how to use it. We will tell them about the process. They need to be told how we will move forward with them after that. If it could be done, then maybe it will be easier to mitigate those challenges.

<Files\\KII\\ > - § 3 references coded [13.41% Coverage]

Reference 1 - 1.43% Coverage

I: Do you think that there would be some barriers of using this kind of tool? We have targeted adolescents for this, do you think there would be any negative consequences for them?

P: We have to make adolescents aware about what the tool is about and try to convince them by giving them proper information about the tool before asking them to fill it out. If we clear out the objectives and purpose of this tool to adolescents then they will give their answers properly and obviously the tool will work and don't have any consequences.

Reference 2 - 7.70% Coverage

I: We have targeted adolescent for now but do you think the result varies or there would be any difference if this tool is filled up by adolescent's parents, teachers or any other person on behalf of the adolescent?

P: This tool have many such questions which is best when the adolescents themselves would fill it up. But in some cases where they are not able to, then we can ask someone with whom the adolescent is comfortable with such as their parents or other care givers to fill this form out. In such case, the adolescents should answer correctly and the one filling this form would tick the answers according to it. It can be done but still if the adolescents themselves would fill this out, then it would be more accurate.

I: Is it? Ok. In some cases, if we have to approach the adolescent's parents to fill-up this form on the behalf of their children but according to their own perspective, what do you think will happen?

P: If parents or other person filling this form doesn't ask the adolescent about what they feel or what is their view to these questions and begin to fill up this by themselves, then the fact can be missed. Parents would fill up the form according to what they see from the outside but would not be able to fill out what exactly their children would feel, they will just fill this form by their own perceptive. Parents could not be aware about many things going on in their children's life such as about the love affairs or the sexual harassment their children are going through because children may not share such things with their parents. So, I think this won't be that accurate if the parents or any other person will fill this form on behalf of their children instead of adolescent themselves filling this out.

I: Ok. So, adolescent themselves should fill this form to make it more accurate?

P: Yes. If adolescents themselves would be able to fill this form then their feelings, their perception and the problem they are facing will come out.

I: We have this section about high risk and low risk which would pop up accordingly once after the adolescent complete this form. Do you think there would be any negative consequence to adolescent if they find out they are at high risk of depression?

P: It depends on how we convince them. If we tell them that being at high risk of depression doesn't mean that you have to panic and stress out because it shows that you have a bit high chances of having depression in your future and for decreasing the risk and preventing depression we are giving you this form where we could identify your risk and do whatever it takes to decrease your risk, then they will understand. Today's kids are very understanding and they will be able to understand what the purpose of this tool is if we clarify them before asking them to fill this form, so there won't be any such negative consequences among them.

Reference 3 – 4.28% Coverage

I: As adolescents are understanding and they won't be stressed out seeing whatever their result is but what about their parents because parents are the ones who are very worried about their children. If they find out that their children are at risk of having depression in some years of their life, what is the chances of them worrying more about it and stressing out?

P: Of course they will be stressed for the time they hear about their children having high risk of depression, but there also comes the same approach of convincing them before telling them that their children are at high risk. We have to tell them that we are using depression risk calculator to predict risk of their children so as to prevent them from being depressed in near future. They might be scared at first but when we try to make them clear and try to convince them, they understand.

I: So, you are saying that this kind of tool would be useful?

P: Yes, it will be.

I: Beside the things you said like they would be stressed out for a while when they know that they are at high risk of depression, are there any challenges or barriers of using this tool?

P: I don’t think there would be any challenges but what I think could be done to make it more effective is, let adolescents fill this form separately and let parents and school teachers also fill the form separately.

I: Is it? Why would it be more effective?

P: If we do so then there might be things which adolescents might have missed which we could grab from that of parents or teachers and vice versa. This way, we won’t be missing any kind of information.

<Files\\KII\\ > - § 11 references coded [16.90% Coverage]

Reference 1 - 1.56% Coverage

I: *(Introduction about the risk calculator and screening tool given. He was not even aware about screening tool and how it functions. So, I explained how screening tool in actual works and how it is different from risk calculator)*

P: (Interrupt) Can I ask one question? Has the tool been developed for everyone or only for adolescent?

I: For now, it has been developed only for adolescents.

P: What I mean to say is, is this for well functioned adolescent or for depressed adolescent?

I: For now, it is for everyone. Since we are trying to assess the risk. That's why, after having assessment done in everyone (all adolescents) then (interrupt)

P: I was thinking, it is for those adolescent who has depression and that gets persistent in the future. So, it's for every adolescent.

I: For now, yes. Because we are just trying to predict the risk. (Showing the prototype tool in mobile).

Reference 2 - 0.38% Coverage

At first, what I thought was, this tool is to be used only amongst depressed adolescent and for finding out their prognosis but since you said it is to be used in general amongst all adolescent, it is a good thing.

Reference 3 - 1.41% Coverage

I: So, in your opinion, where and who do you think can use this kind of tool as the risk calculator tool has been made targeting the adolescent. In your opinion, so, in what kind of situation and places do you think it can be used?

P: At first, as it has been said that the target group is general population rather than the diseased people, so, regarding general adolescent population as said earlier, school is the best place for such a target group. So, we can take the tool to the school in coordination with the school and ask for an hour or so to fill out the questionnaire individually. If we could do it this way, I believed the best spot would be school, as we could meet general adolescent population there and it won't be burden regarding the time as we could take an hour during school hours.

Reference 4 - 2.08% Coverage

I: Ok, so as you have said that the school is the best place to use the risk calculator as adolescent would be found in large number therein but what do you think would be the best technique regarding the administration of the questionnaire, give it to the individual student to fill up or ask the teacher or anyone else to fill it on behalf of students. Since, it has been made to calculate the risk of an individual, what is your opinion on this?

P: On this matter, if you think practically, it would be quite a tedious job for one single individual to fill up forms on behalf of all the adolescents as a population of adolescent in Nepal is huge and would be difficult to cover up all the adolescents. So, I think, filling up is to be done personally but in case of high risk, more focus can be given to them keeping in mind by bringing them in contact to the service care providers. This would be better, I think. Thinking from practical aspects, if it is manageable and could be done, then the best thing would be by maintaining its confidentiality but due to constraint level of manpower and time, prioritizing the higher risk and asking them for follow up, I believe would do good.

Reference 5 - 1.43% Coverage

I: So, you believe confidentiality is an issue that has to be maintained?

P: Yes.

I: And, while filling up the questionnaire which has questions related to personal issues or personal experiences of adolescent, what if the form is filled by other people on behalf of the adolescent. What are the chances of biasness in your opinion?

P: Of course. Biasness will be there. Questionnaire has to be filled by an individual as his/ her problems won't be known by others. As for example, a couple might seem happy from outside but we can't say about their personal issues that they might have among themselves. So, the individual problems and issues are only known to himself/ herself. So, while filling the form if he/ she had any organic diseases with disability then others could say about it but it can't be for sure.

Reference 6 - 0.13% Coverage

I: So, self-administration technique is better in your opinion?

P: Yes.

Reference 7 - 1.78% Coverage

I: So, are there any challenges while filling up the tool even if we implement self-administration technique as you mentioned earlier regarding the limited manpower and time limitation. If so, how can we overcome this?

P: I have said earlier about the manpower and time constraints. As you said about self-administration technique, constraint coming on that would be, ummmm, while filling up the form alone by an individual he/she would have difficulties in understanding few questions. In order to have a proper guidance while filling out the form, a reliable person, let's not say a guardian because guardian may also be influential but totally a neutral person with whom the individual may feel comfortable and confidentiality is ensured, if we find such person, it would be best but that might not be possible always. So, that may remain as one constraint. In the absence of proper guidance, he/she will go on filling up the form and might end up randomly ticking the answers, so the results might be deviated.

Reference 8 - 1.20% Coverage

I: What might be the possible solution for this? As you said, one could be about making availability of person with whom one feels comfortable. What else?

P: umm solutions. It's not possible for everyone. So, initially we need to ask all to fill up the form. After high and low risk has been segregated then one under the high risk may be evaluated further in next round focusing on the individual approach. They may be asked to fill up another form with theme being the same but modifying the questions, changing the language, making them feel as if they are doing different questionnaires. This is to be done for reliability to ascertain as if they are really at high risk or not.

Reference 9 - 0.22% Coverage

I: So, to ensure reliability we can also ask parents and teachers to fill different version of tool?

P: Yes, that can be done.

Reference 10 - 2.03% Coverage

I: Self administration technique will be effective for literate individual and they can do it by themselves but for illiterate and drop out adolescent, what could be done?

P: Ok. So, now you have raised new problem. (Smile) Initially, we plan to go to school but illiterate individuals won't be found in school. So, it would be quite difficult to approach them. Additionally, next reason for them for not going to school would be because of certain background. As a result of which they wouldn’t be going to the school. This can also be the cause of mental illness, to be truthful. Therefore, to reach them certain community programs should be launched in partnership with Nepal government. Only then, it is possible. FCHVs are present at grassroots level. So, in association with FCHVs such targeted population can be reached. Literate individual can be reached through school. Through FCHVs, illiterate population can be reached. Informant would be an adolescent themselves but for filling up the forms, adolescent being illiterate we ourselves have to provide the manpower for that. Sometimes, FCHVs are also illiterate. So, we need to mobilize ourselves.

Reference 11 - 4.68% Coverage

I: And, when we ask an adolescent to fill out the tool then at the end, result is revealed where one could either be at high risk or at low risk. To what extent do you think revealing result as a high risk or low risk to adolescents is appropriate? This is because stigma related stuffs might exist and how will they perceive after knowing the result. For you, do you think it matters? Herein, we are just trying to reveal the risk, so do you think it won't be problematic or do we need to follow certain steps?

P: If someone is given level/tag of depressed, of course, they will obviously be hurt. However, while approaching them, our technique is also diplomatic. We have not diagnosed them, but have just categorized them into high risk. Therefore, this is the diplomatic middle way out which is appropriate (smile). Even if one has depression then we can say him that he is in the high risk and has probability of having depression and these are the steps to follow. We can take him through middle way and even if he has illness then we can drag him to normalization process. Therefore, in reality, I think this approach is appropriate. In addition, at certain point, when their illness and high risk factors will be known, we should ask them to seek consultation and visit health care providers. We can develop this kind of practice on one hand. On the other hand, we can slowly desensitize them by saying you were at high risk and it seems as if you were having difficulties. Your condition could be diagnosed. If he is moving towards normalcy then we can say you were at high risk and by now you have improved. But if their condition is worsening then they have right

to know what they have. Whatever level of distress they are in, How to break the news is one concern but they need to be informed.

I: Ok. This is the last segment of risk calculator questions. We showed you e-version of the risk calculator tool via mobile. Please tell us about your opinion on this approach (electronic version) in terms of accessibility? It is in the form of app now. Do you think its accessibility will be increased this way or are there any alternatives relating to use of risk calculator?

P: It depends on the target population and their location. If we focus on city areas then there won't be any adolescent who do not use mobile. I have previously explained you on how mobile has influenced adolescents (laugh). Therefore, our catchment area and the selection of study site matters. If we are trying to conduct this in remote or rural areas then definitely this might not be appropriate, as mobile is not assessable to all therein. So, making use of hard copes would be best.

<Files\\KII\\ > - § 4 references coded [9.48% Coverage]

Reference 1 - 2.74% Coverage

I: Do you think it is useful?

P: This is very good and you can develop it. It will be useful to study about them.

I: We are developing this tool just targeting the adolescents. Who do you think can use this tool? How appropriate is it to be filled up by schoolteachers, counselor, or anyone who is closely related with adolescents or the parents of adolescents?

P: I think that if we are targeting adolescents, the correct data will be obtained if we can make them fill it because if others answer on their matters, the answer might not be correct. So, I think that it the data will be more accurate if it is filled up by adolescents themselves.

I: Yes data can be more precise if we can obtain from them but there can be some adolescents who are not educated. So, what about involving their parents in such cases?

P: Yes, what you have said is right. There might be various things that adolescents are unaware of and they might not know the risk factors and causes of depression. So, in those cases, if parents will speak, the things can come out.

Reference 2 – 1.19% Coverage

I: Now we are doing it via mobile. Is it right if we do it in this way, fill it in paper or reach to each adolescents and ask the question or do you think it would be better if adolescents fill this up by themselves without having anyone by their side?

P: It will be good if educated adolescents will fill it in their own. But not all get the chance for education in our country. Even if you, who work in this field, will ask them, they will answer you.

Reference 3 – 3.91% Coverage

I: After the adolescents fill up this, we have kept the categories as mild risk or high risk. For example if you were found to have high risk of depression after you filled the information, how would you react? Would you be stressed out because you find out that you are at high risk of having depression or would you consider that you were just at the high risk of having depression and it's just about risk and there is no certainty but need prevention. Or would there be more negative effects?

P: It depends upon how people think but after knowing that, one should think to get rid of it. Not all think in the same way. Some can be negative about it as they now know that they are at high risk of depression while some can think that they should be far away from the cause of depression.

I: So, it depends up on the individual?

P: Yes, it depends upon individual.

I: We talked about showing the result at last. Is it better to show the results to the adolescents who fill the form or should we inform about them to their parents or school?

P: If you reveal the result to the individual, they may not be able to think in correct way at that age of their life. If you can inform school or teachers, it will be even better and we can act for it accordingly. Otherwise, you can inform it to their guardians but if show the result to the children, there can be more negative effects. It means that after the information is filled up, it will be better if we tell the result to the teachers or guardian.

Reference 4 – 1.64% Coverage

I: Is it sufficient if we inform them last as, "being in high risk, you should take initiative for help" or "you are found to have low risk so, you should have healthy food and follow these instructions" or should we add anything?

P: I think, you should focus the high-risk group more. If I have known more about this, I would have been able to tell on this. Neither we need to teach about depression and nor I know its symptoms; therefore, I cannot say more about it. However, you should tell the low risk ones that they are at low risk of depression and address high-risk group more to say about the initiatives they should take.

<Files\\KII\\ > - § 3 references coded [2.35% Coverage]

Reference 1 - 0.27% Coverage

These days, almost every adolescents are into the access of mobile phones, I think that it will be useful.

Reference 2 - 0.63% Coverage

Therefore, after the family is aware, the family members will also look after those who are at high risk or I think it will be better either if we can use them under direct supervision of someone or conduct in the form paper-pencil tests and so on.

Reference 3 - 1.45% Coverage

I: Sir, how useful would it be if we allow the adolescents to fill up that form or make them fill up the form under our, parents, school teachers or anyone's supervision but reveal the result to his or her parents, school teaches or anyone nearer to him or her and inform that he or she is in high risk and need of any sort of help?

P: Yes, that can be better if we can make some mechanisms to track his risk and track his or her parents, person he or she trust, or anyone closer to him or her for its answer. But I don't know how such mechanism could be developed.

<Files\\KII\\ > - § 7 references coded [9.87% Coverage]

Reference 1 – 2.45% Coverage

I: Yes, exactly. So how do you foresee people using this? Like an adolescent, a teacher, a parent—what would your opinion be on that?

P: For using this?

I: For using this, yeah.

P: For using, uh screening tools? Yeah it's ok, uh—one of the very good facts of screening tools is that they have the symptoms, you can capture ok: this is the thing. So it kind of gives them an idea about, uh, depression, even though they don't know about it ok? So standard depression…so it has something, not because—so when we go, and tell them "*this, this, this, this sign is depression*"…but when they do it themselves, then it gives them a more better idea.

Reference 2 – 1.17% Coverage

P: Since we are doing a low-risk potential and a high-risk potential, it should not be in the doctor's office I guess? Because uh, when they're really a problem, I think only they come there; that would be just high-risk ones. But for the low-risk ones, I think youhave to just do it in the community (laughs).

Reference 3 – 0.08% Coverage

P: Or in the schools.

Reference 4 – 1.52% Coverage

I: Ok, so you think they should be in the schools? What do you think about—going back to the high-risk and low-risk, what do you think about them popping up for the adolescent to see? Do you like that idea, do you think it should be shown to other people? How do you think that would work?

P: I think this one, like you said it is more…not for the clinics, I guess. It's more for non-clinical purpose.

Reference 5 – 0.28% Coverage

P: I think a person should be trained, because it can be misused anyways.

Reference 6 - 1.86% Coverage

P: So there should be a training to use this, and people should know what is—should be very true—and you know, sometimes it happens that it's…to get. So you have to train the person. And also, uh, they might be scared, like "ok, if I do this thing they might call me, uh, they might label me 'depressed.'" Or you have to just, uh, explain everyone before using it. I think that is always there when you use a screening tool. So, I think information is most important, and training information.

Reference 7 - 2.52% Coverage

I: Ok. Who do you think should be giving that training?

P: (Pauses).

I: And this is not a yes or no, right or wrong answer (laughs) just whatever you feel—

P: (Laughs) I know, I know. Everyone who wants to use it, I think you people have, uh, an idea about that more than me (laughs). And you must have worked out on it…I have to think, really think hard!

I: (Laughs) No, but it's completely fine! I mean, that's also why we're asking you too, because you have this expertise and you can see where the need—

P: As I said though, I think the teachers will, or community…not um, not that freely available; It can be misused, I don't know…(*trails off inaudibly*)

<Files\\KII\\ > - § 15 references coded [2.96% Coverage]

Reference 1 - 0.30% Coverage

P: I think that would be better, because a lot of the times, what happens is…when things get translated into Nepali, it might not be…it might be difficult for certain kids to understand, because they speak Nepali but they might not understand Nepali as well as we would expect them to.

Reference 2 - 0.05% Coverage

P: Um, if it's for Nepal? There should be an option.

Reference 3 - 0.11% Coverage

P: So they can have it in Nepali or they can have it in English, whichever they are comfortable with.

Reference 4 - 0.08% Coverage

P: Length I think is fine, but then I think the order needs to be worked on.

Reference 5 - 0.19% Coverage

P: Because a lot of things—they just suddenly pop up, you know? Like, "how is your relationship with your mother" should possibly not be the first question that you ask the child.

Reference 6 - 0.31% Coverage

P: It could be something that's more general or subtler, and then you go deeper. Because family-related issues could be stronger for the child to deal with in the beginning. So, if you

ask, "how is your relationship with your mother?"—instead of mother, you probably start with, "your friends."

Reference 7 - 0.15% Coverage

P: Yeah. Because it's there and then...certainly there's a question about abuse. So, that maybe needs a better placement. You need to go in slow.

Reference 8 - 0.37% Coverage

P: First, we can say there is no right or wrong answer, no one is going to know—so that's there. Then we can probably say, "if any of the questions make you feel overwhelmed in a way, then

you can probably stop, and then if you want to talk"—there could be a psychosocial support [resource] or something that goes along with giving the questions.

Reference 9 - 0.19% Coverage

Because, a lot of the questions could possibly be distressing for the child, as the child is filling it out. But, I don't know whether that's—because this is an online thing, right?

Reference 10 - 0.24% Coverage

P: But if there was a statement somewhere, like "when you are filling these out, do you feel— " then you can probably get medical help, you could go to a website, if it's written there. Like, based on "high risk," you know?

Reference 11 - 0.18% Coverage

P: Yeah, I think there were around 15 questions, right? So we could probably go to 20—20 should not be a problem. 20-25; But, maybe more than that, they might get bored.

Reference 12 - 0.06% Coverage

P: Resources as in—what they can read about, or contact--?

Reference 13 - 0.27% Coverage

P: We don't have a mental health helpline; we do have a suicide helpline, but—that might not be the place that a person who has depression would call. It's specific for suicide, but...possibly we could suggest for them to get mental health consultations, or...

Reference 14 - 0.21% Coverage

Nowadays, what's happening is that every school is supposed to have a counsellor, as per the rules. So, praying that that actually happens, the child could probably contact the counsellor in the school.

Reference 15 - 0.25% Coverage

P: Yeah. Go to your school counsellor, you could possibly need a mental health consultation. But...visit a school counsellor or talk to your parents about seeking medical help. Because the child is not going to go by himself or herself.

<Files\\KII\\ > - § 11 references coded [4.74% Coverage]

Reference 1 - 0.39% Coverage

Another is about the uchcha jokhim (High risk) group. This word comes to be like that, upon translation. People can hachkinu (Stress out), if we tell them they are at risk. I think we could rather tell that there is high chance of getting mental health problem. I think that the person will think that he or she is in high risk.

Reference 2 - 0.41% Coverage

P: There are words to be revised in our context. We might also need to link with family rather than only addressing parents because we live in a joint family. Sometimes, they might have good relationship with their parents but the cause of their problems might be the relationship with other family members. We might need to include such things as well.

Reference 3 - 0.78% Coverage

P: It is high-risk but I said if we could change the word (respondent means the words 'high risk') as word can make many differences. But after returning, she did nothing and her family started calling her pagal (mad) but eventually, after 6 months, she got psychosis. Therefore, we should be careful about how we use words. A Words is not bad in itself, but its meaning depends on how we understand and how we can make people understand it. They may consider low as nothing. I think it should include things related to their roles on having high, moderate, or low risk.

Reference 4 - 0.49% Coverage

P: He or she might not understand how you explain them. I think we have to pay attention towards the possible consequence that can result. Using it personally might have some risk factors. When we say that he or she has high risk, if he or she attempts suicide, we happen to send wrong message to the society if they consider that our question resulted to that incident. I think we have to ponder upon such things.

Reference 5 - 0.49% Coverage

P: I wanted to go through this portion. We told about sending to counselor or where they have to go but there can be something, which they can do. If I am in the high-risk group, I have neither counselor nor psychologist. What should I do in from my level for coping with that problem? Some basic information about what they can do while having such problems could be provided to help them manage themselves instantly.

Reference 6 - 0.23% Coverage

P: If the risk is found low they might neglect saying that they do not have depression. Therefore, some information to the low and high-risk group should be provided about what they should do.

Reference 7 - 0.74% Coverage

P: It can be able to be used at school and teachers could possibly use it because they might have identified it. Similarly, in the community, there can be such things in the family and we say for some of the children that they have habit of not telling such things. However, family will know more about their children, as extrovert or introvert more than the teachers in school. Therefore, I think we should have 2-way communication with family. We can know it exactly if I can do it in school and then simultaneously in the house. We should have some teacher's view as well as some family's view so that, we can find out the gap.

Reference 8 - 0.45% Coverage

Still, there are things, giving them to fill the form or making them fill the form. If you give them to fill the form, the person might understand in one way and it depends upon how much you can make them understand. If you can make them understand in detail, he or she writes openly otherwise, until and unless, we can make them clearly understand, information might not come correctly.

Reference 9 - 0.30% Coverage

P: If this tool is understood before using it, obviously there is benefit. If any tool could measure the different factors, it is really good. There are various factors for depression and not only a cause so if it can look at all the aspects, it is good.

Reference 10 - 0.23% Coverage

P: If they are not educated, they might be unable to fill up the information. However, the challenge comes from the viewpoint of the teacher or parent towards the adolescent. We have to focus on that.

Reference 11 - 0.23% Coverage

I: So, it would be better if we could make adolescents fill up the form. P: Yes, correct information might not be obtained from the observer. However, the capability of that person is a second thing.

<Files\\KII\\ > - § 2 references coded [1.69% Coverage]

Reference 1 – 0.37% Coverage

P: The adolescents are there in the schools and the high schools up to +2 students. The adolescents are now referred to the age group of 10- 19 years old. So it would be best in the schools and colleges.

Reference 2 – 1.32% Coverage

P: Even while talking about this tool, the parents also are not able to open up as much because of the number of questionnaires that are there. The children tend to relate punishment when we talk about the teachers. This should be avoided. Since these things are there the nurses or the health personnel that are appointed at the school who looks after them (students) at the school when they have any issues. They will solve all their health related problems. And since this is also a health related issue, what is their openness with their health teachers? So either the health teachers or the health personnel could do it as the children are familiar with them at a level. The children get afraid of the teachers.

<Files\\KII\\ > - § 7 references coded [5.08% Coverage]

Reference 1 – 0.63% Coverage

P: If there is free access to it, then it would be easy to given it in the school also. There is nothing that the teacher has to say more. If the teachers were to write down these links on the blackboard and ask the students to go check it out then that would be enough. That would work because they will have the interest to use it and learn what that is all about. I think that it would be good for that age group.

Reference 2 – 0.36% Coverage

P: I would have an interest if I were that age. I would be interested to check whether any of these things would match me or not. So, I think that I would use it as it looks like a quiz. I would have the tendency to check what I would get.

Reference 3 - 1.00% Coverage

P: Since these collect open answers, the place of information collection could be the school. It is not necessary that it has to be done from a single place only. It could be any. If anyone were to come to the hospital and talk about it then we could check them. If the parents of the child are here and they say that the child has stressful behavior, then we could ask them to encourage them as well. The school teacher could also ask them to get checked. People could tell their own family members and friends about it which means that the source could come from anywhere. It will be easier as they are open answers. The only difference is who answers that.

Reference 4 - 1.16% Coverage

P: Talking about barriers, one thing that could be negative is that you are predicting high risk instead of a diagnosis. It is just a prediction. It is not necessary that the prediction is always accurate. Considering the stigma that it has people should not feel that there is massive (pressure on them) that they are at high risk. If we do not provide them with the adequate tools, then their anxiety will increase if they see that they are at higher risk than their friends who took the same test. We should be prepared about the options that could unfold such as the pre- test counselling. We have to reassure them beforehand even if they were to get the result that they are at high risk. We need to tell the people that it is fine no matter what result they get.

Reference 5 - 0.27% Coverage

P: They are of the adolescent age group and they can get scared simply hearing that they are at high risk. They might not know about it which is why that has to be cleared for them.

Reference 6 - 0.77% Coverage

P: This thing has to be under consideration. That is because if I am feeling depressed, then I might not feel like filling up the form also. So, we might overlook a high risk or even a depressed person. Therefore, there has to be a provision that if anyone sees it then they could also get to feel it on their behalf. The parameters

will be the same. But it should not be restricted to only the patient filling it. There also has to be other objective scales. Otherwise, we will miss out on the severe cases.

Reference 7 – 0.89% Coverage

P: There could be some gaps when the adolescents fill it up as well. They would not really remember their own behavioral issues. It means that they would only be focused in behavior if they give it priority. There might not any be other emotional aspects or distress but they will focus on their profile. I might enter all of the symptoms if I were filling it up myself. But it could be different for others. It would be better to get both those information if you were to look at it from the cluster or the group. It would be the best to get both the information for the overall diagnosis.

<Files\\KII\\ > – § 8 references coded [5.19% Coverage]

Reference 1 – 1.35% Coverage

P: I don't say that it's not feasible because adolescents are viewing many such links and studying from them nowadays. They have practice of viewing many things in internet and studying them. Therefore, it's not that it won't work because it's good but as we discussed before, they should have information regarding mental health and there should be at least someone to tell them about the things they are having now. This is because problems arise when someone does not know about him or her.

Reference 2 – 0.44% Coverage

P: Any of the family member can be there. It is so good and I like it so much and after it become final, please say to me so that I can inform about it to others.

Reference 3 – 0.27% Coverage

P: For that family member, friends, teachers, community or anyone who thinks good of that person.

Reference 4 – 0.16% Coverage

I: So all those people can help him, can't they?

P: Yes

Reference 5 – 1.70% Coverage

P: Wherever there is internet service, using this tool is possible but it is difficult for the place not having internet service. Rather, the health post nowadays, have internet connection therefore we can inform about it to the ministry of health and I think it will be better if we go to community by coordinating with them. Yesterday, we had a supervision and Australian 35:55 also said that TPO is also working in this field. TPO is regarded as a good organization in most of the places and if we go through them, they can let us. They can even print and hand over the form to the clients so that the client can see it.

Reference 6 – 0.48% Coverage

P: For that, it is essential to tell or inform them. They might have small phones but they might not have internet and there are very less who can access to all those services.

Reference 7 – 0.57% Coverage

P: As you have told, we cannot tell that all the community can use this service. In overall, to make this service accessible to all, we should have support from the governmental level accessible to all Nepal.

Reference 8 – 0.23% Coverage

P: So that, whatever rural the place is, the service becomes accessible everywhere.

<Files\\KII\\ > – § 6 references coded [3.69% Coverage]

Reference 1 - 0.72% Coverage

I: How much feasible is it? What is the potential that you see so that it could be applicable in our country, Nepal?

P: It will be. Almost everyone now has a reach to mobile phones. When doing it under the monitoring of the guardians [supervision]…

Reference 2 - 1.33% Coverage

I: The guardians need to conduct the monitoring?

P: Yes, they need to.

I: And why is that?

P: When the monitoring is not done by the guardians, there is the possibility that there could be misuse of the mobile phones. They might not pay attention to it. They might divert their attention to other things on it.

I: So you think that it would be better if the supervision is done by the guardians.

P: Yes, it would be better with the guardians' monitoring.

Reference 3 - 0.20% Coverage

I: So in your opinion, it is easy.

P: Yes, it is easy. It is good.

Reference 4 - 0.67% Coverage

P: It is better if the objectives of the application are made clear beforehand, so that people do not have to worry about whether it is being done negatively. That is why it has to be cleared before to get them motivated (to use it).

Reference 5 - 0.39% Coverage

I: Who do you think should motivate the adolescents?

P: The family members, best friends and school teachers can be their motivators.

Reference 6 - 0.39% Coverage

I: So you feel that it will be easier to fill it up under the observation of the school teachers?

P: Yes, it will be easier as well.

<Files\\KII\\ > - § 4 references coded [9.16% Coverage]

Reference 1 - 1.81% Coverage

P: As this is a type of individual assessment tool and depression, being a sensitive matter in our checklist or information process, I don't see any difficulties to use it in school but the setting of school should be easy to talk and response. Even family can be a better setting but we can ponder upon how the person stays and so on while collecting information. It can be less useful in the clinical setting because the cases which has already been assessed and the severe cases or high or medium risk cases will reach to the clinical setting whose symptoms are already observed by their family. So, those cases are already diagnosed at a level. For professionals, it might be useful to use the tool for final verification. However, if we are targeting large population, school or house can be a better place to use the tool so that individuals can know about their status and risk level.

Reference 2 - 1.79% Coverage

P: I doubt that if others are made to fill up my information, the information may not be good. If teachers will fill the form, information can be biased. Guardians can fill up the form as per their observation but it depends whether the observation of guardian is correct. As far as possible, if the adolescents will fill up the form, the genuine information can be obtained as per their experience. There are questions about the relationship with their parents. If guardians have some doubt, form our (researcher's) prospective, they can think that if they give correct information, they might feel that others will know that their relationship with their child is bad. Similarly, if we teachers to fill up the form, teacher can also be biased at that place. So, in my opinion, I think the real information can be obtained if we let the adolescents to fill up the form themselves.

Reference 3 - 2.37% Coverage

P: I have assumed 2-3 risks. If they do not get immediate support after they know that they are at high risk… however, it has been written there. If I am the person, till now, I have been doing anyway and today after using this format, if you tell me that I am at high risk and I need someone's care and support. I may or not get support at that time. If I get support, my risk can disappear and I get treatment but if I don't get any support, I can suffer more and doubt that I could be severely diseased, restricted to go somewhere, not able to be supported by anyone, not able to speak and so on. This is assumed risk, which can occur to anyone. Secondly, we talked whether to give this form to individual, teacher, or parent. If we give to fill the form to 2 students in school, teachers might *tikatiki* (talking unnecessarily about others/ backbiting) and concern about it. Probably, if we go through research model, we might explain to school about what and how we are doing, and what its objectives are. Even if friends find them filling up the form for depression, they might *hochchhyaune / gijyeune* (tease someone). So, in my assumption, those can be the risks.

Reference 4 - 3.19% Coverage

P: It can have both parts but more likely, this can be positive. It depends upon the way of explaining to people but working and we will make it. If TPO itself is a research organization, there should be a lot of learning. Therefore, we might need to design some methods as it has already been said earlier that we can simplify. But probably it might not be bad to say in such way. However, I think consent will be needed sometimes because we are taking the information of the person who is in risk. All of sudden, if the

consequences don't occur well, more likely it can be easier if we will approach to make him or her ready by informing him or that their information is being taken and asking if we can share some of their information so that in the future, their parents or teachers can help them to get easier. More likely, it can be easier if we make them ready and go on consulting their teachers and parents. Otherwise, if there is counter (opposite), they can say that he or she is diseased or even the teacher's perception can be negative. Teacher take information from you and pass that information negatively. For example, they can say that the loss of interest in studying because he or she had already been in depression. They do not understand the weightage of the message you give and if they tell in such a way, the consequence can be negative and therefore, it is important to edit it in the level. Ethically, we break the confidentiality if we see someone at high risk. Therefore, probably it might be better if we take at least one consent for this risk.

<Files\\KII\\ > - § 4 references coded [5.24% Coverage]

Reference 1 - 1.18% Coverage

P: One is school area as I am working in school and there are problems in school. Another is working places and office because workload and work related tension is also causing depression to people. Therefore, it will be so better if we can use it effectively in the school, work area and the places containing mass of people where we can detect and treat cases according to their risk level (i.e. low or high)

Reference 2 - 2.53% Coverage

P: Not all can have access to mobile and this program can lack this and not be able to involve all the individuals. For example among 20 of us (school nurse), there was a case of suicide in one of our colleague's case. The individual who committed suicide might have depression. Had she found out the cause earlier, had the individual or the family member known about having higher risk, action could have been taken. Therefore, you have to access it to everyone. You can do anything like conducting in all districts. In the district like Kathmandu, every people will know about it as everyone use mobile or know if the organizations are nearby. However, in the remote place, it not easy and for the same, you can make plan like giving sheets to health organization, coordinating with someone or through school nurse so that you can include more individuals for identification.

Reference 3 - 0.70% Coverage

I: You gave a very good suggestion. You also told about the geographical challenges, which can possibly result. How did you feel about this tool in overall? Do you think, using this tool is convenient?

P: Yes, I think it is convenient to use.

Reference 4 - 0.83% Coverage

P: We can put the things, which we could miss out while speaking. We can call a meeting to tell about the app and ask everyone to use it. I saw a possibility to change the view towards mental health because an individual can self-realize about mental health. If an individual can accept…

<Files\\KII\\ > - § 10 references coded [12.24% Coverage]

Reference 1 - 1.24% Coverage

Well….Yes it is useful but in which place matters. Here in *Teaching Hospital* it is useful because educated people come. But if it goes to some other periphery, like in some people from village is surveyed, that child is not able to understand so it may not be useful in periphery. But yes in center it is useful.

Reference 2 - 2.02% Coverage

Firstly, others… we should make it understandable to caretaker or visitors of Children… though they are adolescent some are unable to catch up things so their caretaker should understand first and if they tell us to make it understand to their children as well. But sometimes that become barrier. If the caretakers is the problem…..that is the problem so it is difficult to make the child do it all alone. But if there is good environment with proper privacy setting and good communication then it can be done.

Reference 3 - 1.80% Coverage

I think in hospital setting, hospital setting that also mainly in psychiatric department it is much better. Because who come to psychiatric department is already aware about the understanding of half of the things. And again if this is done he will be more aware of what to do. Yes it is useful in pediatrics also. Hospital settings where care givers are understanding and medical personnel are also able to make it understand, in such places it is good.

Reference 4 - 0.57% Coverage

For that yah in schools …it is better in schools. Much better because population is also very high in schools, so it is better in schools only.

Reference 5 - 2.05% Coverage

Like adolescent we talked about literate and illiterate also. If he/she is illiterate and cannot fill form by themselves can this tool be filled by his/her parents, caretakers or teachers for the information on his/her behalf. Do you think we get different answers or it is fine?

P: Ah….well if there is no problem in the family then there may not be any differences. Even the parents are concerned for their children. So mostly they give truth answers…good answers.

I: So, we can expect no biased answers

P: Maybe

Reference 6 - 0.56% Coverage

One thing is if the child is told that he/she is in high risk that will create problem to them.

I: Stress

P: Stress maybe...That one thing…

Reference 7 - 0.59% Coverage

To reduce that is to make them understand. Communication in a good way that nothing will happen. The communication and counseling part should be good.

Reference 8 – 1.56% Coverage

Like whatever the problem is first to that only...like…You…like others…if a person has been involved in smoking or substance abuse then at first that person should be informed about their problem and make themselves understand about it. Then only we can ask them to seek help. We have to communicate with them first. This (information in the tool) is generalized, so I think it is not enough.

Reference 9 – 1.40% Coverage

es…then after about that last risk you told about that thing may stress out more. So in that case, suppose if I give you to fill up this form. And you filled it up. How would it be if instead if sharing the result of this to the individual and sharing the result to their care taker or school teacher?

P: Well. Yes that can also be done. That is fine.

Reference 10 – 0.47% Coverage

In this case is there any discrimination or challenges regarding stigma?

P: Maybe not in this…No...Because not in this

<Files\\KII\\ > - § 3 references coded [3.47% Coverage]

Reference 1 – 2.33% Coverage

P: We should… How are they involved in decision making, elders… things about participation should be also there, their opinions are being heard at home or not, are they being involved during decision making at home or not. They (adolescents) first learn from home that how do the elders take leadership, how they

lead the home, how they implement the decision making the process. Then it might be from child clubs, from schools… how is their participation in school, if the adolescents made involved in meeting or not while making the decision about children and adolescents, if their perspectives are considered or not, which is also important. Like, we do not get to know sometimes. We grew up in such a manner that when we were a child our suggestions were not considered while making a decision about us. Neither was taken at home nor at school. We could not learn how the elders make decisions, how to speak in front of the mass at that age. Now the youth are forward. Certain/some organizations have been engaged to teach about the decision-making process, perhaps the rights to participation come in that too.

Reference 2 - 0.83% Coverage

P: Now this… This should be known by all. Like I said earlier morning shows the all… risk calculator… it is about facts… this is more or less scientifically based. Adolescents will be in schools, maximum are in schools. So obviously, we should go to the schools. These things should go to the community as well. If this happens, the risk is high…We should conduct the discussion based on the facts…

Reference 3 - 0.31% Coverage

P: Now, this is about adolescents. I think this should be a big issue for today's adolescents. You showed the version… they will understand Nepali.

<Files\\KII\\ > - § 6 references coded [5.12% Coverage]

Reference 1 - 0.69% Coverage

P: If it is filled-up properly, it would be beneficial but if not done carefully, disadvantage is also there. If the software is made but not use by the target group, then the tool would have no meaning. So we need to focus on how to make the adolescents to use it.

Reference 2 - 0.47% Coverage

P: It would be better in all forms. If we would present in the field and distribute then the paper would be fine. If doing from distant, online might be good. All forms are good.

Reference 3 – 0.45% Coverage

I: We said it is targeted to adolescents. But if parents, teachers or close friends fill the forms on their behalf, will there be difference?

P: There will be difference.

Reference 4 – 0.24% Coverage

P: The possibility of biasness is there. There will difference in saying by self and others.

Reference 5 – 2.52% Coverage

P: This is completely imaginative question. In my experience, adolescents do not feel like other will understand their feeling. They feel like they are a different creature, they think they are unique, their feelings are unique, nobody understands their feelings. Thus, first they need to understand what depression is. That might include counselors and all. If they do not understand the first phase, there will be meaning for them about what depression is. So rather than the score, first they need to understand what depression is and what the causes are. We need to make them feel that there is a person who understands their feelings and then only the significance of score would be there. He might fill randomly and the score would be high, the message pop up as a high score and recommends visiting a counselor. He won't understand this. If we first make them understand about depression, they will think that there somebody who understand their feelings.

Reference 6 – 0.74% Coverage

I: What do you think is better, explanation by person or displaying in the screen?

P: That is the difficulties of internet. We are not sure if he understands or not. We will have to try our best to make him understand. Even if we display, we have to make them understand clearly.

<Files\\KII\\ > - § 4 references coded [12.88% Coverage]

Reference 1 - 3.36% Coverage

P: It would be good if such a tool is developed. Our problem is that the tool is developed in western countries and validated in Nepal. The result is good until the paper is published but it does not work after that. Those have been the problems. So we cannot rely that the tool would detect everything. The culture, socio-economic status, external and internal relationship differs between us and westerns. So, the tool effective for you might not work for me because of our culture, background, traditions being different. You might not take that as a problem, but same might be the problem for me. It is very difficult to generalize such things. It is very difficult to resolve psycho-social problem though the means of quantitative tools in Nepal.

Reference 2 - 4.67% Coverage

P: The one thing is they will not share immediately when asked. There is problem of doing through quantitative tools with adolescents, it is biased. When you build rapport and maintain close relation, they might be. In urban areas, people might open-up but in rural areas, people do not openly share (their problems). If somebody has fewer problems, they might open-up. After someone opens up, if they find out that s/he is in problem, in risks and realizes that s/he should undergo the treatment, then some may take it positively as well. But if somebody has not realized this and collides suddenly, then they can take it negatively. S/he is not familiar with depression and if we are unable to make him/her understand that depression is just like other physical problems and considered that the mental health problem is like the person being mad, then that will be destructive. But we can convince them that depression is not a problem and it can be cured by taking medicines for certain period of time. Then, that would have positive impact.

Reference 3 - 1.16% Coverage

P: Not possible. Can it be known about you asking your father? How can they know what is inside you? Even your friends do not know. You do not share several things even with your close friends. Adolescents never share. So the right answer will never come out.

Reference 4 – 3.70% Coverage

P: That should not be done either. I told you earlier, first s/he should be explained that just like the physical problems, mental problem is also not a big issue. At present, these kinds of problems have been detected in you, if you do these things, your problems will be resolved. First s/he should be convinced when you say that these kinds of problems have been seen in you. If you do these things, your problems will be solved. Certain problems have been detected in you, because of these reasons. So, do these things. It is not the big problem. Do not take it otherwise. If the reason behind his/her mental problem is his/her family members or teachers, then only that should be shared with them. If we tell them directly, then they… What if the teacher reveals about it to others? What if you add more stress in family?

<Files\\KII\\ > – § 4 references coded [2.44% Coverage]

Reference 1 – 0.74% Coverage

P: Gap might appear. Only oneself knows about own. Mother, family, teachers and friends might not know all. In some places, they might be hiding too. So it can be biased. It is good if it is done by them.

Reference 2 – 0.75% Coverage

P: It can be done in friends' circle but it is good if done at an individual level. Going to the secured place, only after giving orientation class and making them understand well, they can fill- up properly.

Reference 3 – 0.31% Coverage

P: It is good to do online but online... Online and paper tools, both should be done.

Reference 4 – 0.64% Coverage

P: I found it good, fun. We do not have more tools in Nepal. So I like it. Only taking the tools and screening them, it would be good if awareness class also can be conducted.

<Files\\KII\\ > – § 3 references coded [3.96% Coverage]

Reference 1 – 1.31% Coverage

P: So not as a researcher but me as an adolescent, if I get the opportunity of using this tool I'm thinking what would be on my mind. The first thing that I would stand out is, if I reached the high risk my question would be "what now?" So there is a range of age for adolescent. In that sense, I think different kind of intervention is needed. And there will be a difference between a 12 year old using this tool and a 24 year old using this tool. So, what have you thought about the age?

Reference 2 – 0.81% Coverage

P: So one thing is according to age group and description and another question is, where is this available? As I've understood it is in the internet, right?

I: Now it is in online version.

P: Oh, is it like an app?

I: No, now it's just in online version but we're thinking of developing it into an app.

Reference 3 – 1.84% Coverage

P: So there will be more access because everyone has their smartphone in their hands which is good and they will find out easily by staying home. But its downside is that if we have bandwidth or not because there are very limited mental experts, counsellors and psychiatrists in Nepal, Kathmandu. So if this app is

used in other places than Kathmandu that access will be lesser. For example I figured out that I'm in high risk and I know where to go but there's no access in my village and there are no expertise to go to. And my society stigmatizes mental illness. That means we helped to find out the problem but solution will not be enough for everyone. And that's what I find risky.

There are pros and cons to the online version:

<Files\\KII\\ > - § 2 references coded [1.80% Coverage]

Reference 1 - 1.39% Coverage

I: More than questions, anything more than that. For instance, you said earlier only giving link is not enough, where the (services) can be taken, how they charge for service. That can also be added.

P: Yes that can be added. How to do that? If need to pay...if said this link, then you need to inform, you might need to pay this amount there or it can be free, this has helped. Information about free might come next but we need to think, what would be the role of TPO. To make it function continuously, what would be the sustainable mechanisms? Who is needed to be involved? I think as a stakeholder if the Ministry of Health and Population would be contacted for this forming a...

Reference 2 - 0.41% Coverage

P: Researchers, program implementation and government agencies if coming together sometimes there are working in the non-government sector as well, taking those projects and forming a group might work.

<Files\\KII\\ > - § 7 references coded [8.25% Coverage]

Reference 1 - 0.45% Coverage

P: Questionnaire is one of the best ways to get solutions. There might not be many changes. Questionnaires are prepared mostly finalizing and managing everything.

Reference 2 – 2.59% Coverage

P: Sometimes, children, right answers... For instance, s/he is going through any problems but s/he does not want to show, the data that comes is wrong when not desired to be shown. Due to that, problem... Let us say... how much to be... If s/he has problems but they do not want to show the society or do not want to show it to the next person other than themselves, then as the data does not come, the possibility of challenges is there. The possibility of more problems being created for him/her is also there. Problems emerge everywhere in every place. Talking about the case of the student, the main (technique) is to motivate him/her because that must be properly defined to him/ her about why the problem arises from anything. The right data... S/he can tell the facts when defined properly and move ahead. If the facts are shared, the data that comes would be right and they shall not face many problems in the future.

Reference 3 – 0.25% Coverage

P: That is it. The main is to motivate the student, counseling him/her, why this happens.

Reference 4 – 1.23% Coverage

P: If there would be help, then that would be good because at least there can be anything s/he has not understood. Sometimes the proper answers, facts are not drawn when questions are not understood. So one should be there for help, then reality, in addition, counseling, further let's say... Through motivation, inspiration, they should be suggested that if there is a problem, there is a solution to it. The facts can come out effectively.

Reference 5 – 0.18% Coverage

P: It might not be possible to go into the link in every place.

Reference 6 – 1.09% Coverage

P: That would also be good. Another, what I suggest is that when the adolescents, they are all related to schools. In that case, conducting some kind of one-day seminar, orientation class in schools might also be better. The concerned people, authorities if they could conduct program going themselves, whatever has been targeted to give, what has been attempted to do, the output can come.

Reference 7 – 2.47% Coverage

P: There will not be a problem where there is access to the internet. There will not be a problem to go online and get any information. Where there is no internet access, the related personnel should be present. Mainly, our focus is on unavailable (internet) places. The awareness program must run in such places. It is fine for the city areas as everywhere there are such facilities but the problem has not been witnessed in cities only. There are rural areas that do not have the vehicles. Electricity is not properly available. The Internet has been properly made available even in the cities recently. But if we go to rural areas, we still do not find that. It is not necessary that everybody has smartphones with them. In that case, going there and conducting one or two days' awareness program... Where there is no internet access, being present oneself and if could be aware...

<Files\\KII\\ > – § 2 references coded [3.38% Coverage]

Reference 1 – 1.19% Coverage

P: They can get confused with the questions if they try to fill it up themselves. In one topic maybe things can come mixed up to them. They will be confused about the answers to the questions. That's there should be a person to tell the fact. They also should not hide the fact, you know? In that way it will be easier. It will be easy if there's one mediator.

Reference 2 – 2.19% Coverage

P: It will be very easy if you take this in the form of a program. And you should take this in the form of a program not only in *Kathmandu* but also in other districts. Anyone can suffer from depression, people from

cities, and people from villages. It's not that "this caste suffers from depression but this particular caste won't suffer". It can be caused by different reasons to everyone like: social problems, *sambighatmak* (emotional) problems, mental problems, etc. If these type of things will be internally researched and treated it will be better to prevent children from depression. It will support in making children a better citizen free from depression.

<Files\\KII\\ > - § 5 references coded [5.35% Coverage]

Reference 1 - 1.19% Coverage

I: Our coverage is that this tool should be accessed to everyone. So should we go through the same strategies or is there any other way to make this accessible in different settings?

P: One is website which will be easy for everyone.

Reference 2 - 0.90% Coverage

P: Yes! Another is we can endorse the tools from our secretary and implemented. We can develop a guideline or we can do something so that it can be incorporated in the policies.

Reference 3 - 0.90% Coverage

P: The adolescents may not be ready to answer the questions and that may be a problem. They can think of the mental health negatively. But there may not be so much of challenges.

Reference 4 - 0.57% Coverage

P: No, there won't be any challenge. That should be explained clearly by the head teacher or the health teacher.

Reference 5 – 1.79% Coverage

I: Yes, in the form of recommendation. If we take this in school setting they can take it and give it to the students to fill it up. In that way, teachers are involved. Other than that in what kind of stake holders can we use this?

P: After school, we have health facilities. Likewise we have Mother's group and other clubs, NGOs schools and colleges.

<Files\\KII\\ > – § 1 reference coded [1.31% Coverage]

Reference 1 – 1.31% Coverage

P: Challenge will be there in every work. As the depression has been a well-accepted health condition, there should not be that difficulty. Whenever we work in mass, stigma gets reduced automatically. Stigma is persisting as we have been dealing at individual level. When we go amongst mass, when we into public, when we organize program in the community, stigma gradually reduces. Thus, with this as well, stigma might be reduced sooner; trust over it is a big question.

<Files\\KII\\ > – § 2 references coded [7.76% Coverage]

Reference 1 – 4.94% Coverage

P: When I first heard the word predict, I had thought it would be different. Basically, this is adverse life experiences… What are the adverse life experiences of children at the current time are the proven factors for depression? Further, what I think is score… a total score is at one place but I think the severity of each domain also determines. For example: the person has been through sexual harassment. Everything is fine with him/her but has been through sexual harassment. I have not seen that (risk calculator) in detail. But these sorts of issues should also be given weightage/consideration. This is important. We ask these in clinical history as well. When this ask, children… Are there current symptoms or not… I think this should

be weigh/consider with strength. Children's strength… In a lot of cases, children have excellent coping skills but have stress. What it shows is at high-risk. However, if the child has good psychological strength… That is why I think this should be weighed with strength as well. Along with the risk factors, many supportive factors also exist, right? Should weigh with these things which can negate? That is one thing. The other is these types of tools… I think this should be useful. The referral might increase in our contexts. It might be useful in a sense that though this is not highly specific if could be even sensitive then it should work.

Reference 2 – 2.83% Coverage

P: I think that the interesting in it… The interviewer doing it is in one place. Usually, if a child comes to the interviewer… we always say this whenever we train somebody… It is important… need to work for…The other is… The other version of it could be like a self- rater. At the current time, every child, adolescents as have digital mobiles and all, this self- rater should be used to gather self-rating of own and gather own scores. Further, provide the information automatically that they are in high-risk. I think if that can happen, then it will be useful. It is not that hard to develop another version of this. Mainly, it is about identifying the stress domains of adverse life. If could explore cultural specific adverse… that type of is developed, then it can be useful in another form.

<Files\\KII\\ > – § 2 references coded [1.26% Coverage]

Reference 1 – 0.76% Coverage

I: For instance, if we made this tool to be accepted from the policy level, what can we do to effectively implement that?

P: It needed to be applied from the school level only.

Reference 2 – 0.49% Coverage

P: The awareness is to be done mainly from the school level. And then factories where there is the more population.

<Files\\KII\\ > - § 1 reference coded [1.79% Coverage]

Reference 1 - 1.79% Coverage

I: If our tool is validated and developed, because this targeted to the adolescents, in the implementing phase do you think there will be any challenges to use this in that age group?

P: I think there should not be any challenges. Have you included only the teenagers in the pure adolescents?

I: We have included younger adolescents which is 10 years to older adolescents that is 24 years.

P: I don’t think there will be other challenges.

<Files\\KII\\ > - § 3 references coded [9.40% Coverage]

Reference 1 - 4.20% Coverage

P: In case of your study, you made this and you may give it and bring it back and people may communicate with you saying that they are in risk. But some people will not believe you. I think this should be done only by the study. Model should be made to those who can help to assist like; teachers, friends or friend circle because people believe friends. Either that or health workers. Parents might not be cheered so much. There might be a tool for parents and maybe they can understand. You should see who will be the appropriate person to share and collect the information. Instead of only model you should see different models from school teachers, researchers, friends and family or health workers. Health workers say don’t do these works. They only do it after it comes. Where are the places that adolescents go? One is school another is gym, clubs. Those places are important. Before joining the clubs I think it will be better to talk about their mental health status. This should be identified by the study. (Paused)

Reference 2 - 1.17% Coverage

P: Challenges depends upon how you use the tool. While studying, let's say a researcher uses this tool, when they use this to they should ask about a person from whom they would like to hear about this tool, and be easier for them. These things should be done by the researchers first.

Reference 3 – 4.03% Coverage

I: If we plan to implement this tool through a non government sector and conduct a specific program, there might not be mass coverage and the whole population might not be covered. So, to initiate this from government level or to incorporate it along with the policy for recommendations, do you think we can do anything?

P: In such case, non-governmental sectors are important. Non government sectors prepares the modules by different studies and lessons. They show it to the government by making different modules. They show it to many settings. That module should be prepared by NGOs. I don't think this can be progressed through government sectors according to the present structure. Like we talked about school nurses, right? So that kind of modules should be prepared. So the things about adolescents should be taught in schools and incorporated along. We should search about the opportunity levels and focus. And progress should be done by catching up to the same opportunity.

<Files\\KII\\ > – § 1 reference coded [1.81% Coverage]

Reference 1 – 1.81% Coverage

P: When we were in school, people used to come for the data collection like this. According to the saying of students and friends, most of the information given by the students are not correct. For example; the relationship with parents. Many of them will not try to tell that they do not have a good relation with parents. They try to hide it and they can say that it's very good but there might be many problems. So, the information can be wrong. If a correct information is given, then maybe we can know the high risk or low

risk but in so many cases, they won't give the truth. And if there is no truth there will be a problem to identify and fill the truth.

<Files\\KII\\ > - § 8 references coded [6.40% Coverage]

Reference 1 - 0.76% Coverage

P: Everyone may not be interested, right? It will depend upon how popular it gets because they will want to use it when they see others using it. They will use if it slowly gets popular.

Reference 2 - 0.70% Coverage

P: It can be a bit doubtful because they might scared to express themselves thinking if that information will be known by everyone. So we should be able to convince them.

Reference 3 - 2.09% Coverage

P: We can know about various incidents of depression. The age group of adolescents are technologically advanced, so if we do it through apps there can participation of many adolescents. And if you keep the buttons of link so that if anyone is suffering and needs help it can be easy because they may not want to talk to someone or call someone but if that one click can help them because one click is easier than speaking to the people who hesitate. Of they can seek help than we can reach faster to the change.

Reference 4 - 0.21% Coverage

P: It will be easier if we do this in the schools.

Reference 5 - 0.64% Coverage

P: Yes exactly. They can be sad about that they are in high risk. The high risked person should be taught about the ways to cope and follow up the situation.

Reference 6 - 0.95% Coverage

P: Option should be given after they are in high risk. Further assistance can be provided online to the high risked people and if there can be further counselling about the ways to cope with that situation I think it will be better.

Reference 7 - 0.49% Coverage

P: Everything might not be true because they already hesitate to speak. So, I think the true picture might not appear.

Reference 8 - 0.57% Coverage

P: To use this on adolescents the questions should be short and the options should also be in a language they would be able to understand.

<Files\\KII\\ > - § 11 references coded [4.69% Coverage]

Reference 1 - 0.18% Coverage

There are no one who would not be using mobile these days.

Reference 2 - 0.76% Coverage

P: About adolescents…not every children and adolescents use mobile phones. There is this thing about the age. From which age they are allowed to use mobiles is the first thing and they might not know everything about it. If parents use this, they can!

Reference 3 - 0.21% Coverage

I: So, adolescents can do it with the help of their parents?

P: Yes!

Reference 4 - 0.30% Coverage

I: They might not answer every questions in front of their parents…

P: Yes, they might not do so.

Reference 5 - 0.67% Coverage

P: To know about the risk, the one who have been facing the situation should answer rather than their parents or the teachers. If they are to fill up this tool with someone beside them, I do not think it would be feasible.

Reference 6 - 0.52% Coverage

I: So, it would be feasible for them if we give it to them and ask them to fill it by themselves?

P: Yes. That way, the adolescents can make their decisions by themselves.

Reference 7 - 1.03% Coverage

P: People have a nature of lying to others! We have to create an environment so that they would realize their mistakes and or this we have to conduct different awareness related programs. They will remember about it while they get to fill up the form. So, you have questions with options here. Adolescent can lie about it. Many of them do.

Reference 8 - 0.50% Coverage

P: If so, they will surely fill it up. People are like…they can lie to everyone but not themselves…if they think like this then they can surely fill it up honestly.

Reference 9 - 0.19% Coverage

I: How effective would this be?

P: It will surely be effective.

Reference 10 - 0.10% Coverage

P: Yes…mobile… (Not clear audio)

Reference 11 - 0.23% Coverage

Even if the person fill it with the wrong answers what can we do, isn't it?

<Files\\KII\\ > - § 7 references coded [6.95% Coverage]

Reference 1 - 1.26% Coverage

P: I think we can go through two different methods. In city area, which have access to mobile phones, it is very feasible. But I think we have to think about next plan. We have lots of rural settings in our country, so

I think we have to make another strategy for those in rural settings which do not have access to mobile and technology. And for those who use to mobile phones, I think it will be very useful.

Reference 2 - 1.39% Coverage

P: Firstly, we have to give them the proper information. Children will fill up anything they like, even if that's not correct. Recently, students gave us something saying that it is about their research. we were getting late for our classes, so we looked at each other and how each other have filled the form and then filled our own. So, I think we have to make the students understand the purpose of the tool, then I think they will fill up this right.

Reference 3 - 0.55% Coverage

P: I think going to the schools and letting teachers as their students to fill up the form would be a better option in the community. I think the real data would come in that way.

Reference 4 - 1.12% Coverage

P: At first, we have to convince the students that these problems have been increasing. We have to first give them the concept. For instance, we can take example of Dengue as well. So, we have to tell them first that, if we are not able to identify the problem then it will be a problem for everyone so it is important to write the factual thing here in this tool…

Reference 5 - 0.30% Coverage

I: So, if we do so, it will be effective?

P: Yes! It will be easier for teachers to facilitate.

Reference 6 - 1.39% Coverage

P: Yes, it is really important. Until and unless it does not happen to you, you don't know anything about it. So, I think training is really important. But then again, how many trainings we would take… (P and I laughing)… but as the problem have been increasing and it is creating many problems…everything has been linked to this like behavior, exercises and many more…so I think we should give trainings to teachers making it a separate part in school…

Reference 7 - 0.93% Coverage

P: Along with this, we have to contact the local leaders and representatives for this…we can go through the schools as well as the community leaders…because those community representatives have an image in the community…they have impacts on people…also we can go through the organizational level as well…

<Files\\KII\\ > - § 9 references coded [6.10% Coverage]

Reference 1 - 1.11% Coverage

P: For children…there are many children who don't know how to use mobile phones but still are going in to depression and we might not know. My daughter was facing such problem but I was unaware. There could be many people like me. So, for children it will be better in school. But for those who are a bit older, who is of the age 10 to 17 or something like that and who are having some relation with boys…so, if we keep it in the online version like you showed me, then it will be better.

Reference 2 - 0.42% Coverage

I: So it can be made online and also introduced through the school setting?

P: Yes. There are not such children who don't go to school these days so in school it would be a good idea.

Reference 3 - 0.54% Coverage

P: The child would fill up the thing that s/he knows but sometimes the child can fill up the wrong answer also because they don't still know that what is right and what is wrong. So, there might be some mistakes while filling up this form.

Reference 4 - 0.20% Coverage

P: I don't think they would do this, they won't say that they won't be filling the form.

Reference 5 - 0.04% Coverage

I: Is it?

P: Yes.

Reference 6 - 1.35% Coverage

P: At that time, maybe they will be scared to fill it. There are many things that they have hide from their parents. May be we also have hid many things from our parents at that time. I did love marriage, my parents didn't know about my love affair. In my batch in school, I was decent among all. My *fufu* were only 4 or 5 years older than me and they also used to study with me. So, I was the smaller one and the decent one in the batch but then I was the one to marry without consent of my parents and ran away! So, children of this age group might have hidden many things from their parents.

Reference 7 - 0.56% Coverage

I: So, we also talked about using this tool from school. How comfortable would the students be to fill the form in front of their teachers? Will they open up?

P: The small children would open up but the older age group pf adolescent might not be.

Reference 8 - 0.67% Coverage

I: So, it means it would be better if the third person like us should introduce this tool to the older adolescents and ask them to fill it up?

P: Yes. Teachers are regarded as parents so they won't open up in front of them. They are scared from teachers. So, there should be someone from outside.

Reference 9 - 1.21% Coverage

I: After they fill this form, the result would come out. In case of children below the age 18, we have to previously take consent from parents so obviously if they are at high risk we would be saying it to their parents. But in case of children above the age of 18, what should be done? Should we tell their parents if they are at high risk?

P: Parents should be informed about it anyhow. At the beginning, we have to tell the parents what we are doing. And if they are at high risk than their parents should be informed about it.

<Files\\KII\\ > - § 8 references coded [4.41% Coverage]

Reference 1 - 0.63% Coverage

P: I would like to repeat the same. It is for sure that we generally conclude something on the basis of reference and plan action. There is no doubt on this. We cannot say in 100% that this person is at risk but that might be possible through categorization under different levels. I think this can be implemented. Experience is great thing. If experienced (this refers to qualified health personnel or trained person) personnel use this then we can expect good output.

Reference 2 - 0.74% Coverage

P: I think this type of tool, if developed can be used not only by the client but those who are not suffering as well. It is must when one has already suffered but for those who has not suffered yet can also know in advance. This will be helpful to entire society. It is not relevant in this regards but just sharing. Last time,

when difficulties arises in an engineering field, incorporating those things in curriculum was solution proposed. Therefore, either in school or in other sectors if they are incorporated in curriculum that can be worth.

Reference 3 - 0.67% Coverage

P: To talk about filling procedure, it matters on what ability a person carries, like one is unable to write, one is unable to express, one is more aware of things. But the best part is, if filled on behalf by person who is well aware of psychology can be of help. Else, even if I have been suffering from the problem I might not be able to express or write. So, if this person shares his problem a bit with person who understand psychology, I feel reality could be revealed this way. I feel so.

Reference 4 - 0.30% Coverage

P: Not only this but like you are talking with me, you can discuss with family of sufferers. On the basis of this, you can write more. Right? If you give directly to fill then I don't think you will get appropriate result.

Reference 5 - 0.56% Coverage

P: What I feel is, you have kept high risk, middle risk and low risk but sometimes, it is difficult to exactly differentiate categorically. I think experiences should be added as well. Human intellectual needs to be applied therein. It will be difficult if you try to mark only. It will not only be explained in written form and human intellect is not like mathematics. Understanding at once to what other feels!!!

Reference 6 - 0.65% Coverage

P: One should be in personal touch and as far as possible should try to probe more for depth understanding. This tool is nice of its kind and will give some base. But my request is not to rely on this 100%. Because, this is merely mathematics kind. But human intellectual is something that we feel. Herein, we cannot write how we feel when we look at each other. On the base of this only, will we become able to make decision for someone's life? I don't think that is possible.

Reference 7 - 0.24% Coverage

I: So, According to you, in additional to this tool there must be human intellectual like someone who would (interrupt by respondent)

P: Yes, an experienced person along with.

Reference 8 - 0.62% Coverage

I: So, you don't accept it if it is made available via internet or filling in computer by self?

P: Laugh. I don't agree with this. Who would know if any person is severely depressed inside upon seeing him normal outside? Can it is be known?

I: That is what I am saying about how questionnaire can be filled (Interrupted by respondent)

P: One can tick in negative option for questionnaire. But, if asked in sentimental or sympathetic way then one can express.

<Files\\KII\\ > - § 5 references coded [1.20% Coverage]

Reference 1 - 0.41% Coverage

P: If they fill up by themselves then they will reveal their feelings. Rather than other filling it, if they fill up by themselves then their inner feelings will be put forth and it will be known. So, it would be easy. So, I think it is best for them to fill by themselves.

Reference 2 - 0.05% Coverage

I: you think that way?

P: yes

Reference 3 - 0.35% Coverage

I: But sometimes there happens to be an illiterate adolescent. At such time, should others fill up on their behalf? What do you think of this?

P: Yes, if that is the case, they should ask the adolescent and fill up their response.

Reference 4 - 0.24% Coverage

I: So you think we can use this anywhere like in schools.

P: yes, if this is done at school, they will know it from beginning and it is easier to fill there.

Reference 5 - 0.15% Coverage

P: Yes, if teachers are also involved then they will discuss about this in class and spread further.

1. Code Query Output

<Files\\KII Transcripts\\ > - § 1 reference coded [0.15% Coverage]

Reference 1 - 0.15% Coverage

Respondent: erm..you can also talk to a religious person.

<Files\\KII Transcripts\\ > - § 2 references coded [1.03% Coverage]

Reference 1 - 0.35% Coverage

Respondent: this one you're doing now is periphery. The major task...the major challenge...the main fight you want to fight, is how to implement it

Reference 2 - 0.68% Coverage

Respondent: eh...sane society, a progressive society. Because the people we talking about now here mainly are the future of tomorrow. and the sane of this nation tomorrow, depends on them. If you start planning for them now, at this stage you're preparing them for a better future.

<Files\\KII Transcripts\\ > - § 1 reference coded [0.58% Coverage]

Reference 1 - 0.58% Coverage

Interviewer: do you foresee any challenges with using this? With an adolescent using this? Like is the language okay?

Respondent: what I'm looking at, the the...well, think that has been addressed from the demographic level. You Nigeria is a multi-linguistic eh ehn

<Files\\KII Transcripts\\ > - § 5 references coded [5.45% Coverage]

Reference 1 - 0.61% Coverage

Interviewer: What are we assessing in them now?

Respondent: A parent that is maltreating his or child unknowingly, if a questionnaire is being administer to him or her you'll be able to know and caution him/herself. "Okay this thing I'm doing is not the right thing". They too should be administered questions too for everything to come together at the end of day.

Reference 2 - 1.04% Coverage

Interviewer: What are the potential benefits of using a calculator like this?

Respondent: It is very beneficial. There is no bad aspect of it. In the sense that an adolescent that the questionnaire is being administered to will be able to know the level which he or she is about depression. Because at end of the day he/she after answering the questions will see the result and of course after answering the questions and he sees the result he should be counselled. So as to let him know that come this is your result, so you need to start working on this, working on that so as to avoid depression or in the future.

Reference 3 – 1.29% Coverage

Interviewer: What impact do you think knowing that an adolescent with high risk, how does this impact the adolescent's life?

Respondent: If the adolescent is not assisted or is not helped at that time, it can even make that adolescent to be depressed as at that time. Initially we're looking at future, that in future this adolescent might be depressed or will be depressed. So if he has already known that he has a high risk, it can make such adolescent to be depressed at that time so he needs assistance to let him know that "come, if you do this and did this you won't be depressed. Don't have that mind-set that you'll be depressed" let him/her follow those steps their supposed to follow. In a nutshell, such adolescent needs an assistance or a counsellor to help.

Reference 4 – 1.21% Coverage

Interviewer: Does knowing an adolescent is high risk affect how teachers interact with those students, or if a parent knows that their adolescent is high risk, then what effect would that have on parent-adolescent interactions?

Respondent: To me, it shouldn't be something that erm…okay since you've seen that this person has a high risk, then you start treating that person badly or you start erm…you should be of help to do the person. The teacher should be of help to that adolescent, the parent should be of help, should take it as a project that "ha come this erm adolescent must not go astray" so start erm helping the adolescent immediately, don't stigmatize the adolescent, you know start treating him/her badly.

Reference 5 – 1.29% Coverage

Interviewer: How can risk calculator data be used, for example, should it be used to inform risk reduction interventions, referral, provision of resources, what type of resources?

Respondent: My dear sister a lot of questions has been asked. Since you came now you've asked me a lot of questions. We've talked about the school setting in relation with the adolescent, we talked about the home setting, we talked about the health sector, we talked about the government. So I believe these result you're getting from this questionnaire shouldn't be restricted. For instance, only the government alone or be restricted to school alone. Every one of

them should make intervention. So they should be aware of this thing, of the result that you get out of this questionnaire.

<Files\\KII Transcripts\\ > - § 4 references coded [4.74% Coverage]

Reference 1 - 1.25% Coverage

Respondent: It helps us to have easy…to be able to easily detect if a child or if an adolescent will actually have depression. Instead of you perambulating, once you're able to get all of these things at least you know. Just like in the medical field, when somebody is telling you I'm vomiting, my head is aching me, I'm feeling dizzy, I missed my period, the next indicator is that you are pregnant you understand. So I think it'll help us we don't have to spend longer time than we are supposed to use. Even the adolescent himself or herself is seeing this, he/she would understand that oh so that means this is what is wrong with me because you're doing it together. When you're reading the question to the adolescent, or you give it to the adolescent and let the person…look at the analysis of what we have done concerning your responses. This means this is what is happening to you, so how can we help you.

Reference 2 - 1.15% Coverage

Respondent: Such a person…the next thing you want to ask is "you see what the response is now, that means you're at very high risk of depression". At that point you now make the person see the risk of depression. The immediate benefit is that you want this thing to end, but the truth is that let's look at the consequences on your family members, on your loved ones, on these and that and at the end…do you want us to proffer a solution or you still want to take it like that. You understand it's just like a pregnant woman walk into a facility and you tell the person you have high blood pressure, this can affect you and your baby or you have ectopic pregnancy. You and your baby are at risk, what do you want us to do? Provided the person has seen the result of the test, the next thing is what is the way forward. So it will help.

Reference 3 - 1.25% Coverage

Respondent: Definitely yes. If I discover that this person is at high risk of depression, number one personally I won't just allow the person go back home the way the person has come. We need to really work on the intellect of the person, if the need be for us to request the presence of the immediate family members I think we should do it, a prompt response has to be given to such . You don't allow somebody like slip off our hands and go you understand. You getting somebody who is HIV positive now…and the person is really angry in his spirit or her spirit that "ha gbogbo awon to fun mi ni HIV yii lo ma ko" (I am going to make sure I give everyone this HIV) you don't allow such a person go into the street like that. You need to really work on that person and even track all the people that the person had had sex with if it is possible, so that you can test all of them to prevent the prevalence of HIV.

Reference 4 – 1.09% Coverage

Respondent: All of the things you have mentioned are supposed to…but then I know you have to start from somewhere. And in starting from somewhere, I think the basic thing that you can do is for you to first identify those stakeholders that you think you should train, so the when you now get people that are at risk or that are depressed, you the right channel to channel them to. So that we don't just go, look for people that are depressed. When you now get them where do they go to. You just get them for getting them sake, no, but at least let there be resources available, let there be centres that people will go to. Train the people that will man those places, then identify people that are depressed so that they can now go to those people and at the end of the day everybody is happy.

<Files\\KII Transcripts\\ > – § 3 references coded [2.91% Coverage]

Reference 1 – 0.46% Coverage

Respondent: I think this just like err a statistics on how to quantify the high risk or people that are going through depression and err statistics of how you know one can take care of it and all that. But if this can be really administered, it'll really help and goes a long way.

Reference 2 – 1.05% Coverage

Respondent: Well erm, is gonna affect the adolescent in the sense that you know, for you to know you're in high risk definitely you get scared you understand. and again that's the reason someone should be with him/her just to tell him fine you might be at high risk but you're in the safe hands, these are the things that you are meant to be doing now, you need my help, you need a help of so, so and so, you need to stay away from so so things, you need to do this, you need to do that. So that is why it is important for someone to be beside them just to you know counsel them immediately if they know that they are at high risk for depression.

Reference 3 – 1.40% Coverage

Respondent: I think when it gets to stage when a child is feeling that form, I think, I wanted to say this before that erm it'll be better for the child not to know if he or she is at high risk or low risk. Let it be known to the person attending to the child. So the person attending to the child will know how to…and because sometimes if they know, it might trigger up some other things you understand. so that's why it is even better sometime for them not to… fine they may be filling the form, but when it comes to that aspect where it's going to calculate if they are at high risk or low risk, it should be best known the person that is attending to them. Because normally children they are very sensitive, it is what they see they react to, it is what they hear they react on. So I believe keeping it away from them or the society will really help a lot.

<Files\\KII Transcripts\\ > - § 2 references coded [3.92% Coverage]

Reference 1 - 1.82% Coverage

Respondent: The potential benefit is…I mean one of it is that it'll enable us to know those who have high risk or low risk. And those ones that will require immediate intervention. So this will enable us to know and it'll also give us an insight into…so we can know for how long an individual has been having mental health issues. Then it will also give us a picture to what goes on within an individual social milieu. It will also let us know a little be about the family dynamics. It will also let us know whether there has been some psychosocial issues within the family. It will enable us to know whether there has been any form of physical abuse or emotional abuse or parental neglects you know and all that.

Reference 2 - 2.10% Coverage

Respondent: So that is possible. That was why I did say that people who will administer this questionnaires should also be trained too. And then that training should be inclusive of what to say and what not to say. So if an individual have adequate information about a particular illness, you know that person that person can become an expert patient. So that she'll know what to do and what not to do and where to seek for help, whether to seek for immediate help or delay it and still watch out. And then a little bit of talk therapy can also douse the tension, can also reassure the individual that is at high risk to take things gently and all that. Information is powerful so when an individual has enough information to work on, the issue of self-stigma will not be anything to…I mean that would pose a serious concern.

<Files\\KII Transcripts\\ > - § 2 references coded [2.16% Coverage]

Reference 1 - 0.47% Coverage

Interviewer: What are the potential benefits of using a calculator like this?

Respondent: The benefit is that you get the real statistics of err the real answer that will give you a pointer to a child that you have depression or you don't have depression.

Reference 2 - 1.70% Coverage

Interviewer: How can risk calculator data be used, for example, should it be used to inform risk reduction interventions, referral, provision of resources, what type of resources?

Respondent: Well you know a particular data is used for a particular client. Definitely after getting all these information, it'll serve as pointer to how we can handle these clients individually in other to move out of their depression. These data can be well developed because it's just like you going to a doctor and lying to a doctor that this this is happening to you. The doctor will administer wrong drugs to you. But if you can come out and let the doctor know this is what is

really wrong with you, then you're ready to be helped. Definitely there's no how the doctor will give you a wrong medication. Now that this data is giving us the full details of a particular client, I believe it will go a long way in solving that client's problem.

<Files\\KII Transcripts\\ > - § 1 reference coded [1.19% Coverage]

Reference 1 - 1.19% Coverage

Interviewer: What do you think are the potential benefits of using this risk calculator?

Respondent: It would help them in the sense that they'll now know…as in they will be aware of their society. They'll have the knowledge of what is going on around them and the will have knowledge of what err life can bring to them.

<Files\\KII Transcripts\\ > - § 1 reference coded [2.39% Coverage]

Reference 1 - 2.39% Coverage

R: ah! It will really help our youth since our youth are the leaders of tomorrow. We will have a better society for the youth, it will really help them it will even help their leadership role. You know some of these children, it is not as if they don't have the capacity to lead but because of fear is the fear is the lack of err having err expressing oneself that they're not given the opportunity. You understand that makes them to withdraw back into their shell. It will bring out the best in them and they will even be able to realise I have this potential in me and they will build on it. The parents and the teachers too can do it. Everything must not be academics. You see a child that loves singing and you ask the child to keep quiet because you want her to have A1 in what do they call it , first class in the university. They have different potentials that we can help, it might even be what led to that depression. Maybe he child loves singing and they will be shutting he child up both in school and at home but the child can do both, if they have any school activities out the child were he can perform that potential in him he believe should not be buried

<Files\\KII Transcripts\\ > - § 2 references coded [0.98% Coverage]

Reference 1 - 0.37% Coverage

I: this was designed for an adolescent to use. Do you think it's something other people can use maybe a parent or a school counsellor for example?

R: Definitely….

Reference 2 - 0.61% Coverage

R: if a school counsellor uses this, it shows the high risk or low risk...because there are different.it goes to different aspects of depression. Symptoms of depression. if he/she is reserved, sleeplessness...i think it points to different aspects.so it's very okay

<Files\\KII Transcripts\\ > - § 1 reference coded [0.90% Coverage]

Reference 1 - 0.90% Coverage

Respondent: Parents can, uhm health workers can and guidance counsellors can. Most of the times it's actually parents that are actually meant to actually assess and observe. And when it's a guidance counsellor or teacher that's actually identifying, then the risk factor is already getting high but if parents can actually observe on time then actually it's still okay. Then most times I think children would prefer it that is actually their parents that actually notice they have issues.

<Files\\KII Transcripts\\ > - § 1 reference coded [0.76% Coverage]

Reference 1 - 0.76% Coverage

Interviewer: what are the potential benefits of using a calculator like this?

Respondent: well you know, by the time the responses are completed, they're calculated, the result that is obtained should be useful enough to help in sorting out the issue of depression. Then if it is well graded and calculated.

<Files\\KII Transcripts\\ > - § 5 references coded [5.86% Coverage]

Reference 1 - 1.10% Coverage

Interviewer: you think the parents, teachers and health workers should also use it?

Respondent: parents, teachers, health workers all of them can use it.

Interviewer: okay. So where should they use it and when would the use it?

Respondent: to me when you have the feeling, or when you're trying to identify somebody is feeling somehow depressed at an early age is better use at early age than when it has gotten out of hand. So it will better to use it earlier.

Reference 2 - 0.83% Coverage

Interviewer: so what do you think are the potential benefits of using a calculator like this?

Respondent: the benefits are; it helps us to get rid of depression if we have one.

Interviewer: because you'll identify it early.

Respondent: yeah. It also prevents depression. It prevents depression because you will know what is going on with someone

Reference 3 - 1.16% Coverage

Interviewer: so now let's assume an adolescent has gone through this risk calculator and the result is that the adolescent has a high risk of depression. Do you think this will impact them in any way knowing that they have a high risk of depression?

Respondent: yeah it will impact them, because once you know that you have high risk of depression, then you can easily you know, maybe all sort of things that you do that make you sad, you easily cut them out. Or meet medical specialist.

Reference 4 - 1.15% Coverage

Interviewer: so it can help positively. Is there any negative aspect to knowing that you're at a higher risk of depression in future?

Respondent: there's also a negative aspect.

Interviewer: in what sense?

Respondent: because sometimes, you know you, something you conceive it become a part of you. Once you know that you're at high risk of having this, then you begin, I think to me even worsen the case. Because if you know it, you'll now be thinking, thinking over and over again.

Reference 5 - 1.62% Coverage

Interviewer: so how can the data we get from this risk calculator be used? For example, should we use it to create risk reduction interventions, should we use it to provide resources for the identification and management of depression early in adolescents? Like how do you think this data should be used? Of what benefit will this data be to us?

Respondent: eh the benefit is if it gets, as soon as it gets to the right hands, then they should be able to you know, make use of it.

Interviewer: in what sense?

Respondent: make use of it like identifying the major problems and being able to take it or transfer it to the necessary desk and eventually treat the depressed person.

<Files\\KII Transcripts\\ > - § 5 references coded [1.16% Coverage]

Reference 1 - 0.16% Coverage

I think the benefit is just that we can use it to survey. We can use it as a survey just to know how depressed these adolescents are.

Reference 2 - 0.27% Coverage

Well it will help us to know the potential benefits of those that might be depressed in the future. And at times having get to know those ones that might be depressed, such people we can follow them up almost immediately.

Reference 3 - 0.42% Coverage

Well, it should change for good. If you have been a parent or a mother that has been hostile, that has not been following up with such child, that means you have a second chance. Will it not better for you to call that child now and prevent depression or you allow such child to go into depression then you get…then you want to treat him/her. No

Reference 4 - 0.15% Coverage

Are there any concerns on risk calculator storage and accessibility? Respondent: It depends on how you want to administer it.

Reference 5 - 0.17% Coverage

I think the information gathered from here, should first of all be used for awareness. Awareness first, then any other thing can follow up.

<Files\\KII Transcripts\\ > - § 4 references coded [2.45% Coverage]

Reference 1 - 0.22% Coverage

Err it will show at a glance what the adolescents in the society are faced with. Their response either yes or no, it will show how close they are to their parents, it will show how they are being molested and the rest.

Reference 2 - 0.55% Coverage

Does knowing an adolescent is high risk affect how teachers interact with those students, or if a parent knows that their adolescent is high risk, then what effect would that have on parent-adolescent interactions? Respondent: Yes. When you know that a child is being depressed… Interviewer: No at high risk for depression not that the child is depressed. Respondent: Then the parents should quickly work on that child. Interviewer: Does it affect how they interact with the child either the teacher or the parents? Respondent: No.

Reference 3 - 1.48% Coverage

Yes. That's why the admin should not be there. When the questionnaire is administered, and collected back so you now do your survey, the risk factor you understand, those that are on high risk. So what I suggest is a seminar should be organized to address everybody at the same time, so that one does not feel that "they know me that is why they are calling the seminar" do you understand. I've gone to a church program, I'm a children teacher in the church. So we had a provincial program for teens and err children. So in the seminar, a group of …all these people that take care of children and teens, they were the people that organized that program, the church invited them. Just like what you said they administered questionnaires to the teenagers aside their parents, so when the children they filled the questionnaire, they asked their questions and everything, they now brought it, they counselled the children they left. They now brought their questions to the parents, in fact that day it was very hot. The moderators started reading out the questions to the parents, what the children were writing about their parents, about their uncles, about their daddies, even some pastors in the church and the whole place got "a wire" in fact. So the parents were counselled, they did not mentioned any child. They now said the parents should go back home and talk to their children, know who their children is.

Reference 4 - 0.20% Coverage

The data gotten from the questionnaire, the essence is to curb depression. So whatever suggestion is being from the questionnaire, I think they should use it in order to move the society forward.

<Files\\KII Transcripts\\ > - § 7 references coded [4.45% Coverage]

Reference 1 - 0.39% Coverage

okay basically, you'd be able to notice those that had the risk of becoming depressed in the future and those that have low risk of depression also.

Reference 2 - 0.21% Coverage

yes ones we find out they have high risk, you'll be able to treat them on time.

Reference 3 - 0.69% Coverage

what impact do you think knowing that an adolescent with high risk, how does this impact the adolescent's life?

Respondent: such an adolescent would in the first instance, you know would be stigmatized. Will draw back because of such information he/she knows.

Reference 4 - 0.70% Coverage

yes definitely. Such an adolescent, will be given special treatment by the teacher. Because such a teacher knowing that this student has high risk of depression would not want such a child to go into depression. So would be treated specially compared to other child.

Reference 5 - 0.66% Coverage

first of all, the interaction would be better, if it was not better before it would you know, improve because any parent that definitely cares for his/her child would want the good of the child. So the child would be well interacted with by the parent.

Reference 6 - 0.38% Coverage

are there any concerns on risk calculator storage and accessibility?

Respondent: yes is should be accessed by only personnel not just anybody.

Reference 7 - 1.43% Coverage

yes basically, for the data to be created in the first place is for it to uhm, solve problems. So basically the data gotten from such an evaluation should be kept number one, then should be used on it each uhm, based on the risk, whether it's high risk or low risk. An adolescent found to be high risk should be contacted, taken care of you know, spoken to always, get information about this person, then talk to the adolescent from time to time. You know, invite this adolescent, provide resources for the adolescent and so on and so forth.

<Files\\KII Transcripts\\ > - § 7 references coded [5.86% Coverage]

Reference 1 - 0.87% Coverage

Respondent: We have been talking since that we want to actually reduce the risk of depression. So with this questionnaire we can actually go, this questionnaire can actually go a long way in reducing the number of people who will think of suicide, and then you know after being depressed.

Reference 2 - 1.29% Coverage

Respondent: Anybody that find out that he/she is in high risk of depression, if you are able to talk to such a student you know, I feel it will go a long way. All these things we are saying, they need to be talked to, they need to know the end result of being depressed and all that. With the word of God and generally let them hear of people who fall into depression and what happens to them and all that. I think that will help.

Reference 3 - 0.64% Coverage

When you know that you have a problem, the problem is half solved. So if they know that they actually have depression or tendency to be fall into depression I think it will go a long way in helping such a person.

Reference 4 - 0.99% Coverage

Does knowing an adolescent is high risk affect how teachers interact with those students, or if a parent knows that their adolescent is high risk, then what effect would that have on parent-adolescent interactions?

Respondent: No it does not affect it. All we need to do is just to help those that are depressed to come out of it.

Reference 5 - 1.14% Coverage

Respondent: I don't think so because if there is awareness, you know we talked about awareness. Let everybody know, if everybody is aware they would rather help instead of just putting that person in a particular place and say "hmm, this one let the person stay on his own or her own". Will prefer to help such a person to come out of it than for us to stigmatize such a person.

Reference 6 - 0.44% Coverage

Respondent: There are so many ways you can actually keep these that from being…just keep it within the reach of those that actually need to use it.

Reference 7 – 0.50% Coverage

Respondent: We should use it to train. When you train, those that are trained will create awareness and it will reduce the number of people that fall into depression.

<Files\\KII Transcripts\\ > - § 2 references coded [2.17% Coverage]

Reference 1 – 1.73% Coverage

the children. Maybe when he is teaching it, when he wants to buttress his point, that is how somebody, that is how she, do you get it now? But it should be purely professional.it should be limited to the professionals alone. let them use it so that they will get the desired result and be able to help the children. You know in this mental health we are talking about there's an aspect they call bi polar. Deal with both depression and the manic one. If you allow teacher to handle it and you know is either high or low It can last for weeks or month, if it is high, the same thing, if it is low, if you allow non-professional to handle this question, it can still bring what we are talking to the life of these children cause they wont handle it very well.

I: they wouldn't handle it properly

R: the way a professional will handle it so that is why it should be left to be handled by professionals

Reference 2 – 0.44% Coverage

yes you can... yes you can use it. It depends on the person who is presenting It. you know they said presentation matters, the person presenting it, if the person should present it very well and convince him, yes it can be used.

<Files\\KII Transcripts\\ > - § 5 references coded [5.32% Coverage]

Reference 1 – 0.77% Coverage

I think it's very beneficial, because from the response you get you'll be able to identify people that have the tendency of being depressed in future or those that are already suffering from depression.

Reference 2 – 0.91% Coverage

Interviewer: What impact do you think knowing that an adolescent with high risk, how does this impact the adolescent's life?

Respondent: Yes, it will enable the person to seek for help at that particular moment to that it will not trigger.

Reference 3 - 1.37% Coverage

Interviewer: Does knowing an adolescent is high risk affect how teachers interact with those students?

Respondent: Yes. It will. It make me to come closer, bring the child closer to me and you know make that child my friend so that the child will open up. Then I'll be using, as in talking to the child about how to overcome it with the little knowledge I have.

Reference 4 - 1.49% Coverage

Interviewer: If a parent knows that their adolescent is high risk, then what effect would that have on parent-adolescent interactions?

Respondent: It will. If the parents has been informed and knows that this what the child is going to face if it is not curbed, I think there will be a change in attitude towards the parents' behaviour to the child. The parents will call the child back to...

Reference 5 - 0.78% Coverage

Respondent: To me the final analysis of the research should be used not only to intervene in depression, should also be used to tell the government to come in too in assisting depressed students or persons

<Files\\KII Transcripts\\ > - § 5 references coded [1.76% Coverage]

Reference 1 - 0.39% Coverage

Respondent: well, from the calculator now, if a child has more of the positive he knows the answer already, if you have the negative you know the answer already. It's a very straight forward questionnaire that will easily give them result.

Reference 2 - 0.21% Coverage

Respondent: well, it will help them to be able to detect if they are depressed and also know what to do to combat the situation.

Reference 3 - 0.39% Coverage

Respondent: yes, the interaction has to be positive. Because a child that is suffering from that situation needs more of attention, more of care, more of love. Even if it's to take such child as your own so that you'll be able to assist.

Reference 4 – 0.53% Coverage

Respondent: yes it will, because negatively it will bring separation. Because the parent will not be able to gain the right attention of the child. But on the part of the parent, they should try as much as possible to assist through bringing the child close to themselves. Not at any point in time letting her be on her own.

Reference 5 – 0.26% Coverage

Respondent: it's a research work, it's a way to identify people that are depressed. I think the solution should be the focus. What do we do after identifying?

<Files\\KII Transcripts\\ > - § 4 references coded [4.40% Coverage]

Reference 1 – 0.98% Coverage

Respondent: It'll go a long way in curbing err...reducing the issue of depression in adolescents.

Interviewer: How so?

Respondent: If it being administered, at least you'll have the statistics, if the risk is high or low and it'll make them to know what to do, how to go about it.

Reference 2 – 0.22% Coverage

Respondent: Not really. It'll make me to even draw him closer.

Reference 3 – 1.66% Coverage

Interviewer: If a parent knows that their adolescent is high risk, then what effect would that have on parent-adolescent interaction?

Respondent: Yes. Because some parents are not being patient and they don't have that time to know what the child is going through especially those that are working class parents. Before they come back the adolescents is sleeping, before the adolescent wake up they ate out. So it tend to create enmity between the parents and the adolescent.

Reference 4 – 1.55% Coverage

Respondent: Like after getting all the data, put it together, take the statistics, how the...the rate if it is high. There are process, follow the due process going to...maybe the leader in the

community first, to the government, to let them know this is what is at hand. This is the stage adolescent depression is in Nigeria. And we have some solutions...because we cannot state problems without providing solutions. Tell the government then...

<Files\\KII Transcripts\\ > - § 1 reference coded [0.67% Coverage]

Reference 1 - 0.67% Coverage

R: This one, instead of going one by one, you can make it networking.

I: How so?

R: You can send it and get your feedback immediately. I think that's one of the advantages.

<Files\\KII Transcripts\\ > - § 3 references coded [6.55% Coverage]

Reference 1 - 2.16% Coverage

Interviewer: Okay. So do think that, in terms of you as a clinician, do you think it's appropriate, do you think it's acceptable, do you think it's something that can use in your clinic for example?

Respondent: I think so, very much so especially if it's something that has been validated, I think it's something we can use and coincidentally, those are most of the questions I ask my adolescents, you know, their relationship with their parents, maybe not so much but you'll have an idea when you're looking at them saying it. But yes I think it's something that can be used in the clinical setting.

Reference 2 - 1.25% Coverage

Interviewer: Okay, what do you think are the potential benefits for us using a risk calculator as opposed to using screening tools to make diagnosis?

Respondent: I think a risk calculator will help us identify early those children that risk of being depressed and we can actually put things in place to make sure they don't actually get depressed.

Reference 3 - 3.13% Coverage

Interviewer: Do you think that erm, there are any negative consequences for using the risk calculator for example, let's look at maybe stigma for using it in an adolescent?

Respondent: Erm, I mean if it's used appropriately and erm generally. So if, for example, if you come to hospital and they say that "oh we're doing mental health whatever, so everybody has to fill this" maybe, well I don't know. I suppose there's always stigma to mental health issues and if because you're high risk I'm having to put some things in place, yes there might be some stigma attached to it but it depends on how we go about it, how we present it to the people, that this

doesn't mean that you are depressed, but so that you are not depressed. So that even the people at the low risk end, also get some form of intervention. So everybody gets intervention then the stigma is reduced.

<Files\\KII Transcripts\\ > - § 1 reference coded [1.46% Coverage]

Reference 1 - 1.46% Coverage

Interviewer: what are the potential benefits for using a calculator like this for potential... immediate consequences? What are the benefits and consequences of using this thing?

Respondent: the benefits, is that we'd catch them young, we would be able to address whatever the case is, at it is occurring or even before it occurs. Now the downside is that stigma might even start without the child becoming depressed. Then if it's a child that enjoy playing the sick role, they'll just fall into it. Everybody will pampy them. Do you understand, ...so I think that is that.

<Files\\KII Transcripts\\ > - § 2 references coded [2.07% Coverage]

Reference 1 - 0.50% Coverage

Respondent: Well, it should be positive. The parents should at least be happy that it's being detected early so that it doesn't go to…

Reference 2 - 1.58% Coverage

Respondent: To improve the adolescents' lives. Yes part of it goes to policies, but I don't want to leave it to government alone. Individually, people can step in, NGOS, a lot of organizations can help in so many ways. A child that is being depressed a lot of things can be done to help the child. We'll just look into the aspect that is making the child depressed. If it is family, the school, then adjustment would be made.

<Files\\KII Transcripts\\ > - § 1 reference coded [0.90% Coverage]

Reference 1 - 0.90% Coverage

Respondent: Easy access to before it get to the severe stage…early detection of some symptoms. It might not even be depression, but actually up for other illness. "I have been feeling some kind of way and I think there's something wrong, although I did this..." yes it could help.

<Files\\KII Transcripts\\ > - § 1 reference coded [0.92% Coverage]

Reference 1 - 0.92% Coverage

Interviewer: What do you think are the potential benefits of using this risk calculator?

Respondent: Early detection. If that's one benefit, early detection which would of course engender prompt diagnosis and treatment. 'cause once you screen someone positive, you can follow up quickly to make the definitive diagnosis and hence introduce treatment. I think that's a big plus.

<Files\\KII Transcripts\\ > - § 1 reference coded [1.34% Coverage]

Reference 1 - 1.34% Coverage

Interviewer: Do you foresee any challenges for using this risk calculator in Nigeria?

Respondent: Well I won't say anything is peculiar to Nigeria apart from, because this generation now, a lot of people tend to speak more English. Even if you say you want to make Yoruba version, you don't know how many adolescents can speak Yoruba these days. Even the ----- speaks English these days.

<Files\\KII Transcripts\\ > - § 3 references coded [8.47% Coverage]

Reference 1 - 2.08% Coverage

I: Yeah. Website you can go to. But then, another thing is, do you think we should allow other people use it other than the adolescent, can it be used by a parent, can it be used by schools perhaps?

R: So that means you need to have the parent version and the adolescent version, which someone goes into the thing and it says that okay are you using it for yourself or on behalf of somebody? So that will direct you on where to go. Whether the caregiver version or interested family & friends version or you can call it whatever. It has to be cool, you understand. Family & friends version, "the me" version. Then of course the language will now change depending on the version, you can explore around that. Then if you're a teen or a parent, you can be directed to get attention.

Reference 2 - 1.29% Coverage

I: Maybe if you had access to more resources rather than just the risk calculator questions, you can read and understand better. If it's a parent they too can read and understand better and some of these resources give you some skill on how to cope with your child if you think your child has high risk. It might give you a direct guide on where to go if you need to seek help.

R: So are you creating a website for them to go to? Or you're going to leave them with some random person?

Reference 3 - 5.11% Coverage

I: By the time we're done, each country would have to modify what the outcome would be. If there's a high risk for Nigeria, we'll have to decide what to do.

R: But when you say go to this website,

I: This prototype.

R: Yes, so this website you want to direct them to whose website is it? Is it your website? Is it the IDEA website?

I: No. It should be like, I think the goal here is can it be a country thing

R: We're in this country, we know that these things don't exist. So except IDEA is going to put up a depression website for adolescents. That's why I'm saying why are you doing this? You must be able to think through the process. Where is it going to end? So someone has a high risk, you say okay, you have a high risk, go to some nonsense website that doesn't have any, it just say depression is so and so, it hasn't given any value. But you have a high risk, visit our IDEA adolescent depression website where we can talk about high risk and how you can reduce the risk by doing what, what do you do when you want to reduce risk? You must increase the protective factors. You must balance protective factors and risk factors. Okay this child has a risk you know, let's try and improve your interaction with the child, try and improve parent child interaction. Speak to our counsellor in school if you're feeling left out, you know. Have a solution for all those risk factors. All the risk factors you're putting in this questionnaire, tabulate solutions for it. So check out the table and find protective factors you can begin to work on. So you've given them something they can do with the information and if this fails after a period of whatever then remember you've not diagnosed, then you can put a contact number. So step 1, look at the protective and risk factors scale, then step 2, speak to an expert if step 1 doesn't work. Because this on its own is useless without a connective user friendly site.

<Files\\KII Transcripts\\ > - § 4 references coded [6.48% Coverage]

Reference 1 - 0.86% Coverage

Interviewer: Who do you see using this calculator (parents, teachers, health workers?) Where or when would they use it?

Respondent: I think so. I think erm the authority figures over the adolescents can also use it for maybe if they noticed some of the symptoms in this adolescent, they might be able to complete it and seek appropriate help for the individual.

Reference 2 - 1.99% Coverage

Interviewer: Do you foresee any challenges for using this questionnaire? If so how might we address them?

Respondent: Erm, I think some of the challenges might just be that when we're giving it to the adolescent, we need to make sure that erm they are in…maybe they are alone, their authority figures are not around to supervise them completing it. Because sometimes the presence of the authority figures can influence the responses these children can give. Also make sure their peers are not around them because some of them might want to say "let me see what to wrote" "let me see what you ticked" you know, and you might be able to get the true picture of things. So it's important that maybe when you want to give them, you put them in separate rooms where they're alone and can easily give you erm the responses as they truly feel.

Reference 3 - 2.41% Coverage

Interviewer: Does knowing an adolescent is high risk affect how teachers interact with those students, or if a parent knows that their adolescent is high risk, then what effect would that have on parent-adolescent interactions?

Respondent: I think to an extent yes because erm…it depends I think if you give the teachers appropriate education, I think it might not affect the teacher's behavior towards the child. So before we give this questionnaire out at all, we need to really educate the populace, the people who we're giving it to, we have to educate them that because an adolescent has depression, you don't need to change… your behavior or your attitude towards the child shouldn't change. If not the child will not be…other children will not be sincere with you when they're completing their questionnaire. And you should be an agent for help, an agent for change as an educator. Shouldn't be the one adding more to the problem of that adolescent. I think education should be able to deal with that aspect.

Reference 4 - 1.22% Coverage

Interviewer: How can risk calculator data be used, for example, should it be used to inform risk reduction interventions, referral, provision of resources, what type of resources?

Respondent: It can be used to influence policies, it can be used to influence change and it can get other sectors to maybe collaborate with us. If we have maybe more adolescents with high risk, we can approach NGOs and they might be more inclined in giving us all we need, the support we need to help these adolescents. I think so.

<Files\\KII Transcripts\\ > - § 3 references coded [3.18% Coverage]

Reference 1 - 0.71% Coverage

Interviewer: Does knowing an adolescent is high risk affect how teachers interact with those students, or if a parent knows that their adolescent is high risk, then what effect would that have on parent-adolescent interactions?

Respondent: Well, I wouldn't know but I suspect it might.

Reference 2 – 1.59% Coverage

Respondent: In a negative way, in the sense that they may now begin to, what is the word I'll use to describe this thing…when you erm medicalize otherwise usual behaviour. So the child is upset with the teacher and is a bit withdrawn, and there's "like he is high o, I think he is depressed". There is that risk there, which is why I was saying that the questionnaire should not be available to the random teacher, but a teacher who is the one working with adolescents. So who has a better ability to, in a nuanced manner understand what information that he/she is getting mean, rather than a random teacher that everything will be medicalized.

Reference 3 – 0.88% Coverage

Respondent: Well, there is this erm programs that are supposed to build resilience and err because since it is not a disorder it is a risk, what you can only be working on is promoting resilience. Identify the factors that can promote resilience in children that have erm history of not even depression, that have history of a turbulent childhood as it were.

<Files\\KII Transcripts\\ > - § 4 references coded [4.22% Coverage]

Reference 1 – 0.91% Coverage

Respondent: if we allow adults to use the calculator, we'd probably be able to do a retrograde diagnosis, to be able to find out why they're where they are now. So people who going through depression will probably remember at some point in their life when they had this. So yeah, it helps to also establish that this factors are valid.

Reference 2 – 0.85% Coverage

Respondent: it could or it could actually either cause stigma because you say ah child has depression or ah this child has a high of depression and react negatively to what it ..or it may help you identify that oh this child has been ---- from depression, help you look for ways to solve it before it even happens.

Reference 3 – 0.94% Coverage

Respondent: it depends on the kind of training the teachers have had. If they've been trained on what to look out for, how to seek care and what to do, it can be helpful. But if they haven't, they would either panic and look for the first way out to isolate the child or they make seek professionals to come and assist them or something like that.

Reference 4 – 1.51% Coverage

Respondent: every data is good data. So from the top to bottom, it helps to plan uhm, the kind of services you want to make available for policy makers, programmers, for example, if they have access to the data, they can say oh this is what is going on in this community, or in this state or in this secure area, plan for it and make the solutions available. For school it's also useful because when they get access to it it can also help change the way they provide their curriculum for example the way they address the kids. So yeah every data is good data.

<Files\\KII Transcripts\\ > - § 2 references coded [3.27% Coverage]

Reference 1 - 1.02% Coverage

I: If an adolescent uses this tool and then realizes that he falls in the high risk for depression. Do you think it will affect the adolescent? The knowledge that they said I am at high risk for developing depression I the future?

R: I think the first thing would be how well they understand what depression it. That will determine how they take it.

I: that will determine their reaction

R: I mean for a medical person, [laugh] but for a non-medical person

Reference 2 - 2.25% Coverage

I: if we use this risk calculator for an adolescent and maybe he gets high risk, do you think there's a possibility that there might be stigma? Like if a teacher uses it and there's high risk the, teacher might start treating the adolescent differently because the adolescents is at high risk. Do you think that is a possibility?

R: it depends on the teacher, an ignorant teacher a loving teacher a frustrated teacher [laugh] it depends on the teacher.

I: so it can be good or bad for the teacher depending on how bad the teacher is

R: can be good or bad… yes.

I: how do you think the data gathered from using this risk calculator can be used? Do you think the data gathered from something like this can effect intervention being made at policy level?

R: yes it can, data generally, as long as you can get accurate data. Because with data issues are always with accuracy of the data. If you say its interviewer administered what are the biases there like I said they will tell you what you want to hear

<Files\\KII Transcripts\\ > - § 3 references coded [2.98% Coverage]

Reference 1 – 1.23% Coverage

Interviewer: Who do you see using this calculator (parents, teachers, health workers?) Where or when would they use it?

Respondent: When you look at parents my challenge will be with parent. Teachers yes. Parents some of these questions, "did they beat you with marks" (laugh) I can imagine my father asking me that question, I'd say no even though he has done that several times, I dare not say yes. It might be a bit difficult for parents to administer it to their own children. But definitely teachers, health workers can.

Reference 2 – 0.49% Coverage

Interviewer: What are the potential benefits of using a calculator like this?

Respondent: One, it opens us up to...we have an opportunity to do more assessment, better intervention, faster intervention now.

Reference 3 – 1.26% Coverage

Interviewer: How can risk calculator data be used, for example, should it be used to inform risk reduction interventions, referral, provision of resources, what type of resources?

Respondent: Data is data, data is raw information, raw stuff. You can use it for so many things from experience. It can be used for every one of these issues. Though in erm....at least for an initial assessment, then also we need to collect data from different care forms you know, especially with the professionals to guide what we are doing here further.

<Files\\KII Transcripts\\ > - § 3 references coded [1.90% Coverage]

Reference 1 – 0.26% Coverage

R: yes they should, the child should...you have to train them before they start, so if

I: before they start to use

Reference 2 – 0.46% Coverage

R: exactly it will help them. It may not be for all the teachers. For Instance, but for the key err officers like the counsellors. The one that will see, because the line of authority is also very important.

Reference 3 – 1.18% Coverage

R: yeah it should it should but if you look at the insensitivity of our government and all that I can assure you err what other people have been doing. I know some would have done this and all that, they would have present it to the government and all that. I can assure you since it's not something that will bring money to their table or where they can embezzle and all that I can assure you they will throw it away but if we have a sincere government that is ready to tackle that it will go a long way in addressing that issue.

Code Query Output

<Files\\KII Transcripts\\ > - § 1 reference coded [0.53% Coverage]

Reference 1 - 0.53% Coverage

Respondent: it will also help to know how this adolescent relating amongst themselves, the families, you know. And then you'll be able to also know the IQ of this patients. You know along the line.

<Files\\KII Transcripts\\ > - § 5 references coded [3.62% Coverage]

Reference 1 - 1.46% Coverage

Respondent: you know the problem with things like this is, we have this creative mind, but the implementation of all these things. You know you have...you have...started something how do you turn it to reality. In this case turning this to reality is not like producing a fan. we have technology of...how to add something together to make a fan work. Yeah? But to put them together and make it work, will become...is a major problem. These are some of the things that we really need to implement. Uhm, how to now get it working is...is my fear and that is what you should thats the...that's the major task

Reference 2 - 0.35% Coverage

Respondent: this one you're doing now is periphery. The major task...the major challenge...the main fight you want to fight, is how to implement it

Reference 3 - 0.91% Coverage

Respondent: lay man cannot, we need to like do serious erm...break down to make it easily errm... understandable for the lay man, because we are the professionals we can understand,

some people will be like ki lo n so gan...ki lo n so...ko ti ye mi (what is he/she saying? I don’t understand any of it) you know all these kind of things...I don't know if you understand Yoruba?

Reference 4 – 0.40% Coverage

Interviewer: okay. So are there any negative consequences of using this calculator? Do you think there are any negative consequences?

Respondent: stigma...is stigma.

Reference 5 – 0.51% Coverage

Respondent: ah...won ma mo asiri mi (my secrets will be known). Then, I am sorry, will I call that negative, the psychiatric doctors will have less work to do eventually. They might not like this kind of thing.

<Files\\KII Transcripts\\ > – § 1 reference coded [0.84% Coverage]

Reference 1 – 0.84% Coverage

Interviewer: so what additional resources do you or instructions do you think we need whenever...when we are going to the field with this thing? What additional instructions or resources should we go along with?

Respondent: you have the consent for?

Interviewer: yes.

Respondent: if they can read it and give their consent which is the ethical consideration for an average person.

<Files\\KII Transcripts\\ > – § 1 reference coded [0.94% Coverage]

Reference 1 – 0.94% Coverage

Interviewer: Also are there any privacy concerns on high risk status is handled? Can results be stigmatizing if leaked or exposed?

Respondent: Of course.

Interviewer: Are there any concerns on risk calculator storage and accessibility?

Respondent: I believe the result of the questionnaire should be handled and be kept secret by you the interviewers. Definitely you’ll have your group, your team. So definitely you’ll like to discuss, have this erm discussions about these questionnaires. It should be amongst the interviewers it shouldn’t be leaked out.

<Files\\KII Transcripts\\ > - § 4 references coded [2.12% Coverage]

Reference 1 - 0.28% Coverage

Interviewer: Do you foresee any challenges for using this questionnaire? If so how might we address them?

Respondent: No provided it's free, it's not something that you have to pay for, I think it's okay.

Reference 2 - 0.20% Coverage

Interviewer: Also are there any privacy concerns on high risk status is handled? Can results be stigmatizing if leaked or exposed?

Respondent: Yes.

Reference 3 - 0.66% Coverage

Interviewer: Are there any concerns on risk calculator storage and accessibility?

Respondent: Number one thing is that you need to have a very secured pass for it number one maybe for your…it's just like is an android phone just like every other person uses android number on, you're protecting the data and the information very well either using password and all that. Number two during the period of are you transcribing or translating, what's the next thing that you people do?

Reference 4 - 0.97% Coverage

Respondent: When you're transcribing also you make sure that you're using professionals. You don't just quacks on the street because this person knows how to listen and write something and then you now took the person to go ahead and transcribe you know. You try to get a professional. Then number there I think erm even the stakeholders involved in the research and the policy making also, you have to…I don't want to say an oath now but you ned to sign an agreement that this thing is just for research purpose only, strictly confidential so that at the end of the day we'll make sure that it's for us to be able to impact lives, to make some good policies and decisions because you know lives are involved.

<Files\\KII Transcripts\\ > - § 5 references coded [5.42% Coverage]

Reference 1 - 0.32% Coverage

Respondent: Well I think someone should be beside them. Because even if an adolescent commits a crime by way of law, I think a guardian, parent should be there even when he/she is writing statement.

Reference 2 – 1.05% Coverage

Respondent: Well erm, is gonna affect the adolescent in the sense that you know, for you to know you're in high risk definitely you get scared you understand. and again that's the reason someone should be with him/her just to tell him fine you might be at high risk but you're in the safe hands, these are the things that you are meant to be doing now, you need my help, you need a help of so, so and so, you need to stay away from so so things, you need to do this, you need to do that. So that is why it is important for someone to be beside them just to you know counsel them immediately if they know that they are at high risk for depression.

Reference 3 – 2.41% Coverage

Respondent: Well, for a professional I don't think it should change. You should treat the child accordingly, don't say because the child is going through depression you know, you treating the child other way round you're causing more problem to the child. You thread softly with them so that it won't trigger out some other things. Because sometimes, there are some words they wouldn't want to hear at that point in time it'll trigger out some other things. They need to be treated accordingly and they need to be treated softly.

The parents too, that's why it is good for them to be sensitized that okay now that you r child is going through this, these are the things that you need to know about the child, when the child is doing this these are the things you need to tell…unlike the one I did the last time, I had to counsel the parents too. These are the things you've been doing that the child has been seeing. So it goes a long way, because the one I did last I noticed that each time she does those things, she is depressed.

They said one of the brother will be beating her and all that. I said no she doesn't need that for now, because for the brother doing that it's triggering more other things in her. So for now she needs to be treated, now after we've counselled the child, these are the things you need to be doing when she gets home now in other for her to come out from the depression. You know it takes a gradual process is not a day thing, it's not a magic.

Reference 4 – 0.24% Coverage

Interviewer: Also are there any privacy concerns on high risk status is handled? Can results be stigmatizing if leaked or exposed?

Respondent: Yes.

Reference 5 – 1.40% Coverage

Respondent: I think when it gets to stage when a child is feeling that form, I think, I wanted to say this before that erm it'll be better for the child not to know if he or she is at high risk or low

risk. Let it be known to the person attending to the child. So the person attending to the child will know how to…and because sometimes if they know, it might trigger up some other things you understand. so that's why it is even better sometime for them not to… fine they may be filling the form, but when it comes to that aspect where it's going to calculate if they are at high risk or low risk, it should be best known the person that is attending to them. Because normally children they are very sensitive, it is what they see they react to, it is what they hear they react on. So I believe keeping it away from them or the society will really help a lot.

<Files\\KII Transcripts\\ > - § 2 references coded [2.68% Coverage]

Reference 1 - 0.58% Coverage

Interviewer: Do you think there will be stigma associated with knowing that you're at high risk for depression?

Respondent: Stigma is not inward, it is from outward. Discrimination comes from outside and I don't envisage such.

Reference 2 - 2.10% Coverage

Respondent: So that is possible. That was why I did say that people who will administer this questionnaires should also be trained too. And then that training should be inclusive of what to say and what not to say. So if an individual have adequate information about a particular illness, you know that person that person can become an expert patient. So that she'll know what to do and what not to do and where to seek for help, whether to seek for immediate help or delay it and still watch out. And then a little bit of talk therapy can also douse the tension, can also reassure the individual that is at high risk to take things gently and all that. Information is powerful so when an individual has enough information to work on, the issue of self-stigma will not be anything to…I mean that would pose a serious concern.

<Files\\KII Transcripts\\ > - § 2 references coded [1.47% Coverage]

Reference 1 - 0.32% Coverage

Interviewer: Also are there any privacy concerns on high risk status is handled? Can results be stigmatizing if leaked or exposed?

Respondent: Of course it can be stigmatizing.

Reference 2 - 1.15% Coverage

Interviewer: Are there any concerns on risk calculator storage and accessibility?

Respondent: I think confidentiality should be able to be in place because if any illness, we have to look at stigmatization and when your client is being stigmatized does not really show what

you're doing on that client. Because the client might not really get out of that illness if not taken proper care of. Stigmatization is the key word and that keeping that record should be in total full confidentiality, I think is what we should look at. It's an information that gives details of a patient and should not be disclosed to any third party.

<Files\\KII Transcripts\\ > - § 2 references coded [2.10% Coverage]

Reference 1 - 1.13% Coverage

Interviewer: okay. So now, do you think there should be any issues with privacy, as regards to adolescents who are at high risk of depression? Like if this result is leaked, do you think this will be stigmatizing, will cause stigma?

Respondent: it will surely cause it. Surely.

Interviewer: so do you think

Respondent: so this should be...be somehow. This should be treated secretly.

Interviewer: the result of the risk calculator?

Respondent: it should be confidential.

Reference 2 - 0.97% Coverage

Interviewer: okay. So are there any concerns on how we should store our data? And how it should be accessible?

Respondent: well, to me, the data should be handled by erm...by specialist. Specialist in the sense that these people who are the ones in charge of treating or administering the treatment to this particular set of depressed people. So it should not be something that would be leaked out to anybody.

<Files\\KII Transcripts\\ > - § 4 references coded [0.88% Coverage]

Reference 1 - 0.23% Coverage

Most challenges that we face with questionnaire is people might not say the truth. But when you're administering something like this, it has to be a confidential. It has to be very confidential.

Reference 2 - 0.18% Coverage

Interviewer: So that's how we overcome the fact that they might not say the truth to make it confidential?
Respondent: Yeah, it has to be confidential.

Reference 3 - 0.20% Coverage

Interviewer: Also are there any privacy concerns on high risk status is handled? Can results be stigmatizing if leaked or exposed?

Respondent: It should not get leaked

Reference 4 – 0.26% Coverage

Respondent: If you have a better society, where everybody already know about depression, there won't be stigmatization. Interviewer: So in this society now, you're saying there might be stigmatization? Respondent: Yes.

<Files\\KII Transcripts\\ > – § 1 reference coded [0.38% Coverage]

Reference 1 – 0.38% Coverage

also are there any privacy concerns on high risk status is handled? Can results be stigmatizing if leaked or exposed?

Respondent: yes it can be.

<Files\\KII Transcripts\\ > – § 1 reference coded [1.73% Coverage]

Reference 1 – 1.73% Coverage

the children. Maybe when he is teaching it, when he wants to buttress his point, that is how somebody, that is how she, do you get it now? But it should be purely professional.it should be limited to the professionals alone. let them use it so that they will get the desired result and be able to help the children. You know in this mental health we are talking about there's an aspect they call bi polar. Deal with both depression and the manic one. If you allow teacher to handle it and you know is either high or low It can last for weeks or month, if it is high, the same thing, if it is low, if you allow non-professional to handle this question, it can still bring what we are talking to the life of these children cause they wont handle it very well.

I: they wouldn't handle it properly

R: the way a professional will handle it so that is why it should be left to be handled by professionals

<Files\\KII Transcripts\\ > – § 2 references coded [2.16% Coverage]

Reference 1 – 1.70% Coverage

Interviewer: Also are there any privacy concerns on high risk status is handled? Can results be stigmatizing if leaked or exposed?

Respondent: It depends on the result. To me it depends on the result. If the analysis has been leaked and is made known to the public, there is tendency o, that some that are ignorant of what depression is all about will start stigmatizing others that "this one maybe soonest the person will go and commit suicide".

Reference 2 – 0.46% Coverage

Interviewer: Are there any concerns on risk calculator storage and accessibility?

Respondent: It should be confidential.

<Files\\KII Transcripts\\ > - § 3 references coded [2.06% Coverage]

Reference 1 - 0.88% Coverage

Respondent: well, you know, the child tends to grow up with a sense of inferiority complex. When you discover that, it's like when you have a particular sickness, you feel somehow that you're not uhm, qualified to be in a certain gathering or certain group. So when a child discovers that, it can result into inferiority complex thereby making them feel that they are not healthy or they are not worthy to be in a particular this thing. But with the help of the treatment and the solutions provided, I think he will be able to get over it.

Reference 2 - 0.53% Coverage

Respondent: yes it will, because negatively it will bring separation. Because the parent will not be able to gain the right attention of the child. But on the part of the parent, they should try as much as possible to assist through bringing the child close to themselves. Not at any point in time letting her be on her own.

Reference 3 - 0.66% Coverage

Respondent: of course. You know, children are so...they have this soft mind for anything that happens to them. When you expose something that is confidential to them they tend to hate you. They can never trust you again. So what you just need to is to let that thing be confidential. If there's any reason for you to now refer you can tell them okay o, we want to refer you this. They will have the consent.

<Files\\KII Transcripts\\ > - § 3 references coded [2.22% Coverage]

Reference 1 - 1.07% Coverage

Interviewer: What impact do you think knowing that an adolescent with high risk, how does this impact the adolescent's life?

Respondent: It will deepen...it will even create more depression in that adolescent.

Interviewer: So what do we do now to address that?

Respondent: By seeking for professional help.

Reference 2 - 0.69% Coverage

Respondent: Yes. Because when the information should get leaked, you know adolescents being who they are, they kind of want to use it to be bullying or making that child to feel as if he is worthless.

Reference 3 – 0.45% Coverage

Respondent: If it is paper a d biro it can get leaked, destroyed. I think it should be online ..even online the risk is there too.

<Files\\KII Transcripts\\ > – § 2 references coded [2.79% Coverage]

Reference 1 – 1.28% Coverage

Respondent: Confidentiality will be appreciated in this instance. And also the teachers must have gone through a training at least let them know their stand on this not to use it against them. And for them to have come out, they already identified that they need help. So the teachers should guide them towards that, achieving the help they need.

Reference 2 – 1.51% Coverage

Respondent: Yes, because of the stigma associated with depression. And of course individual differences are there, so you'll have some child that might want to bully or make jest of that depressed child. It'll might have a negative impact. But the responsibility of the teacher or the health worker is to make the child see that you need help, and it is being appreciated that you've come out to ask for it.

<Files\\KII Transcripts\\ > – § 1 reference coded [1.15% Coverage]

Reference 1 – 1.15% Coverage

Respondent: Yes, and where do I come in in that, at the end point. You talk to this person, call this number, and you know it's something we see on the advert of only American advertisements because they are very skewed towards that. Yes, we're now in a place where everybody has a cell phone but don't forget that there's the fear factor and there's the stigmatization factor where "I'll call, what if they ask my name? And mummy ever hears Oh my God!"

<Files\\KII Transcripts\\ > – § 1 reference coded [1.24% Coverage]

Reference 1 – 1.24% Coverage

Respondent: Absolutely. That is why again that links to what I was saying that either the adolescent himself/herself, and there should be further sites where the adolescent can get more information. When we are talking about adolescent, we are talking about somebody who is between 10 and 17, they are old enough to be able to their own stuff.

<Files\\KII Transcripts\\ > - § 7 references coded [10.56% Coverage]

Reference 1 - 0.11% Coverage

Respondent: people can fake the answers.

Reference 2 - 4.05% Coverage

Respondent: so for example, now instead of calling it a risk calculator for depression, I think one of the things people will think about is, ah you want to calculate my risk for depression, they would, very intelligent people will give you the answer they think you want to hear not necessarily the answer that apply. So unfortunately, if you don't say you want to check on depression, it probably will be deceptive. I'll give an example, so we went to motor parks, doing an evaluation in motor parks health and safety program. And we informed the, you know you have to visit the parks first, to tell the heads of the park, oh we're coming to your park to come and do a free health or whatever, but we're also going to connect units and poles to test for so and so. You don't if they ask what you want to use it for how to ---. They can tell their staff, their members. So that day when you are coming to do the evaluation, they'll now not drink that day, they'll not smoke anything. So you'll get false positive results or false negative results because you'll think that oh in this motor part, nobody drinks or they don't use any kind of substance when it's not true. Because they knew we were coming to sensitize them. So that is one challenge that might attach. However, how would you now find out for instance, where you say oh erm...I want to check for these things but I don't want you to change from your normal pattern of living. I don't want you to change from your normal lifestyle.

Reference 3 - 1.10% Coverage

Respondent: how'd you fix that? And I think it's one of the challenges we still have to deal with. For young people in this ---- we may not coin it risk calculator for depression. You can give it another name, you can say erm..how well do you know yourself? Or Google me, I don't know something fancy, something that will be catchy. Or know yourself or you know something not necessarily pointing to that.

Reference 4 - 2.44% Coverage

Respondent: knowing that you have a high risk of depression, it's, is like a screening for example, if you do a screening for a particular type of diseases, learning that you're positive for a certain type of condition, will probably make you even more depressed quickly. Or it'll just make you more aware or careful. But I think for adolescents the young people who are still learning tools, learning coping mechanisms, it's hard to predict where it will fall. It's hard to predict where it'll fall. For example now, if you have a parent who has glaucoma, and you say it can be passed down you know, it can be hereditary. A d then the younger person now does a test that says that

oh you have of getting glaucoma. Either the person responds by making healthier choices, to prevent them from having it or, they just give up and say oh well everyone does not have it to lose this would likely happen.

Reference 5 – 0.47% Coverage

Respondent: it could or it could actually either cause stigma because you say ah child has depression or ah this child has a high of depression and react negatively to what it

Reference 6 – 1.10% Coverage

Respondent: it depends on who is administering the...and I think it's important for them to know that especially if you don't put any stigma around it. You don't make them feel like it is a very big deal, even though it's important. They're more is to deal with. But if they feel that anybody can have access to the results, they'll probably not be willing to do it or give you the wrong answers.

Reference 7 – 1.30% Coverage

Respondent: online platforms will allow them to do it by themselves in their own privacy. They may decide do it at home or in one corner somewhere. Paper based may be a problem because, if they sit down together to answer the question and they say oh what did you write and you write the same as the other person would not tell you the truth about what's going on at home because they don't want anybody else to know. I think any method that would promote privacy would be better.

<Files\\KII Transcripts\\ > – § 2 references coded [2.91% Coverage]

Reference 1 – 1.55% Coverage

Interviewer: Do you foresee any challenges for using this questionnaire? If so how might we address them?

Respondent: The legal aspect. Especially for an adolescence, one that is below 18 years old, the consent of the parents. That's one thing that you do see. There are two ways that they can...they can work with the schools so that it is a part of the package that the parents sign the form. I know that in my daughter's school, there's health day they do screening for eye, the dentist comes and so on and so forth. So I basically approved them, they give me forms to approve such. So you can work with the schools so that you get consent of the parents.

Reference 2 – 1.36% Coverage

Interviewer: Also are there any privacy concerns on high risk status is handled? Can results be stigmatizing if leaked or exposed?

Respondent: There was this same issue with the HIV pandemic you know. Initially in the process, we were using codes then after a while because of err so much enlightenment we just realized that if somebody comes for HIV test just write the name. There was no need then because we have done so much work in the society. So we need to look at that you understand me. Once there's massive enlightenment some of these questions will not need to come up

<Files\\KII Transcripts\\ > - § 4 references coded [3.67% Coverage]

Reference 1 - 1.18% Coverage

I: so we are looking now that we have this information, if an adolescent were to fill this thing in your facility for example and is at high risk for depression. Do you think that would affect the way people look at the adolescent? Do you think that people would start to treat that person differently?

R: in the home or generally

I: let's use your facility for example

R: No, it’s not possible. Perhaps because most of us are professional more or less one way or the other we are trained to deal with these children so…..

Reference 2 - 0.89% Coverage

R: the outsiders that is the situation. Most of them are not trained to identify such they err and they are not trained to somehow deal with that. Anybody that deals with that could be Inappropriately. It could be because of one experience or the other but since there's no err a line cut information given to them to identify based on they can only work with what they have. It may not really work

Reference 3 - 0.41% Coverage

They tend to relax and give you more and better information but if you don’t know the err, what you are using that data for, they may be tense and they want to tell you want you want

Reference 4 - 1.20% Coverage

R: sigh! Definitely it will even, if it were to be adult it will. But like I said the the the reason why we are doing this should be known to them err, they should be assured that even if you are the highest high risk of err person there its good for you because what we are doing is going to address this and make you a better person and all that. Definitely you will not be sad but if he

doesn't know the outcome or the consequence of what he is doing the tense maybe there and it will affect him more and out him into more depression.

1. Code Query Output

<Files\\KII Transcripts\\ > - § 1 reference coded [0.82% Coverage]

Reference 1 - 0.82% Coverage

Interviewer: okay. Then for the low one, research has shown that you have low of depression. With low risk, it is important to take care of your mental health and well-being, blah blah blah. So do you think we should add more to this or this is fine for the adolescents to read?

Respondent: it's okay.

<Files\\KII Transcripts\\ > - § 13 references coded [7.71% Coverage]

Reference 1 - 1.33% Coverage

Respondent: ...there is one that very very significant which is erm...have you ever seen adult in your home...hold on, hold on. Have ever seen adult in your home fighting, you see that leaves a very negative state, erm, stigma in any child, maybe because of what I went through when I was...when I was young there's this experience that's still at the back of my head like yesterday, when my dad and mom were fighting in the home and my dad was like I want to push her on the floor then go drive the car on and you know run over her. I remember he was...

Reference 2 - 0.70% Coverage

Respondent: so, you know and I was a very ...I think must have been 5 or 6 years old, but I still remember it till today. You know, so, this will...this can change the lifestyle of anybody depending on kind of person you are. You understand so, of course these questions are very necessary.

Reference 3 - 0.18% Coverage

Respondent: definitely am sure there will be need for review or...inclusion

Reference 4 - 0.41% Coverage

Respondent: well I can't...is not something I can really say all the...I can't remember all the things I read. But of course, there will always be something to add to it.

Reference 5 - 0.91% Coverage

Respondent: I think the questions are very lenient. But am sure they'll be need for...or do you go to school and you are able to like meet...meet up with what your other mates are able to meet up with? For example, have you been asked to provide some something and you could not provide it because your parents or somebody who doesn't care about you has refused to make provision.

Reference 6 - 1.23% Coverage

Respondent: Now, have you ever drank alcohol smoke cigarettes, or marijuana or use any other drugs? As a question, that question is not complete for me because if you now say have you ever drank alcohol, smoked cigarettes for what reason? yeah is it because you wanted to measure up to a standard that you felt you were not up to? or because you were being bullied for ced to do so. It's just like this one now you've said, this first question, did someone ever try to touch you in sexual way against your will..

Reference 7 - 1.64% Coverage

Interviewer: then section 6, now gives like a bit of information. That research has shown that you've answered. Your answer has shown the future risk of depression, if any of the questions you responded positively are causing you this stress, it is possible to seek help. So do you think we should add more information to this one? Remember this is just a prototype.

Respondent: yes I know. I know. This one you're doing, this is not how we do it on. You're supposed to dedicate a training, where we'd be able to sit down and write not looking at it...apart what you've written, I'll now do my own addition or rephrase some of the things you have written here. Do you understand?

Reference 8 - 0.29% Coverage

Respondent: of course, the...the ability to provide such forum is...might be the challenge you're having, I understand.

Reference 9 - 0.20% Coverage

Respondent: well, for now...but I know...I know they'll always be something to add...

Reference 10 - 0.35% Coverage

Interviewer: okay. So what do you think about the length of the questions? Is it okay? Is it too long?

Respondent: yeah..I think it is precise.

Reference 11 - 0.21% Coverage

Respondent: I think is precise even though I know that there's still need for addition...

Reference 12 - 0.11% Coverage

Respondent: all the questions are appropriate

Reference 13 - 0.15% Coverage

Respondent: it might not be enough, but they are appropriate.

<Files\\KII Transcripts\\ > - § 1 reference coded [7.80% Coverage]

Reference 1 - 7.80% Coverage

Interviewer: so they are 7 sections. Like we already told you, it's between Nigeria, Nepal, UK and Brazil.

Respondent: okay.

Interviewer: so this section one talks about the sociodemographic characteristics. Pick your language, gender, age, right not you're acting like you're the adolescent. Because this is supposed to be for the adolescents to answer by themselves.

Respondent: okay.

Interviewer: so how old are you? Your ethnicity? Family experiences now. How is your relationship with your mother? How is your relationship with your father? How is the relationship between your parents? Have you ever been separated from your parents for a long time, and cared for by someone else? Have you ever seen adults in your home fighting with each other or fighting with children in your home? Did someone in your family or household ever beat you in a way that left marks or required some medical care? Have you ever run away from home for more than one night? Have you ever not had enough food to eat, have you ever not have enough food to eat, or had to wear dirty or torn clothes because there were no other clothes? Have you ever thought or felt that your parents wished you were never born? Sorry... So uhm, have you ever thought or felt that someone in family hated you? So for this family experiences, do you think this is okay? Are the questions appropriate? Do you think we should add more questions? Or do you have e issues with any of the questions?

Respondent: now I don't have any issue.

Interviewer: so for the school and social experiences. In a usual week do you meet up with friends to chat and do things together? Have you had to drop out of school or repeat a grade? In the past year have you gotten into a physical fight in which somebody got hurt? Is this also okay for the school and social experiences? You have no issue with it?

Respondent: no.

Interviewer: We should not add any other thing?

Respondent: no it's fine.

Interviewer: other experiences, Did someone ever tried to touch you in a sexual way or ask that you touch them against your will? Have ever drank alcohol? Smoked cigarettes or marijuana? Or did you used any other drugs? Is this also good?

Respondent: yes.

Interviewer: so section 5 is now where the scores are calculated. We now know if the adolescent has a high risk or a low risk. Section 6 now gives like a, an information on high risk of depression. It say that research has shown that items you answered are associated with future risk of depression. If any of these questions you responded positively to are causing you distress, it is possible to seek help. You can talk about your distress with an adult you trust, a school counselor or a health professional. You can also contact the following blah blah blah. To learn more about the symptoms, prevention and treatment go to so so and so. Is this information to okay for someone who is high risk?

Respondent: yeah.

Interviewer: you don't think we should add anymore?

Respondent: no.

Interviewer: then for someone who is low risk, it just says that, research has shown that you have low for depression. With low risk, it is important to take care of your mental health and well-being. You can go to www.this this this to learn more about about healthy sleep, diet and behaviour. So do you think this is okay?

Respondent: yes

Interviewer: so what do you think about...what was your first impression about this risk calculator?

Respondent: well, it's good. It's good at treat for...simple...

Interviewer: okay. So is the length appropriate?

Respondent: yes.

<Files\\KII Transcripts\\ > - § 4 references coded [3.68% Coverage]

Reference 1 - 1.41% Coverage

Respondent: When it is administered to adolescents in the society, it would make us to know whether such adolescent is a potential somebody that is going through depression or is going to go through depression in future. It has been categorized to high risk and low risk so as to render help on time. Because the reason why the questionnaire will be administered is to render assistance that's the basic reason…because I believe the questionnaire will be administered. Because if at the end of the day, the adolescent in question has answered those questions, at the end of the day after answering all the questions will make us to categorize the risk as low or high. If it is low, definitely just to keep the adolescent informed about depression and all that. But if its high, quick assistance or quick help is needed for such adolescents.

Reference 2 - 0.49% Coverage

Interviewer: Length of questionnaire? -too much or too little?

Respondent: No it is okay. And it is very explanatory.

Interviewer: Do you think it can be easily used?

Respondent: Exactly.

Interviewer: Are the questions acceptable and appropriate?

Respondent: It is to Nigeria. They are.

Reference 3 - 1.24% Coverage

Interviewer: Do you foresee any challenges for using this questionnaire? If so how might we address them?

Respondent: Yes the challenge I foresee is you know, is not all adolescents that are educated. Some of them will read it and will not understand well, even some of them that are educated they might not understand well that. You know I talked about the age range of adolescents, you now start administering this kind of questionnaire to a 10-year-old boy/girl. The way a 19-year-old boy/girl will be different. So interpretation too matters and how the adolescent understands it. Okay it is the work of the erm interviewer. In this case now, if the adolescent finds it difficult to understand well, then the interviewer breaks it down.

Reference 4 - 0.54% Coverage

Interviewer: What additional information would you like to know for someone who has high risk or low risk?

Respondent: I think it's okay. You know if a questionnaire is too voluminous it will bore the respondent. So it's better we make it brief. These questions are rich, very rich so I believe to me you know it's okay.

<Files\\KII Transcripts\\ > - § 5 references coded [1.31% Coverage]

Reference 1 - 0.25% Coverage

Respondent: For me I think with what we've seen here, it's really erm assesses potential depression in adolescents that's number one. It's a good tool for us to assess depression.

Reference 2 - 0.16% Coverage

Interviewer: Length of questionnaire? -too much or too little

Respondent: No it's appropriate, for me it's appropriate.

Reference 3 - 0.18% Coverage

Interviewer: Are the questions acceptable and appropriate?

Respondent: I think it's acceptable for everybody, it's fair to all.

Reference 4 - 0.20% Coverage

Respondent: For me I think it is right. However, if you now feel you want to ask about the environment of the client now you could add it to it.

Reference 5 - 0.53% Coverage

Respondent: You know when we were talking about the people that…what are those factors that causes depression, your immediate environment can also be part of it. But then maybe if they have answered this one, that could now be the secondary question maybe yes, but I think…but it's very short and straight forward you don't have to start looking at the dictionary before you understand.

<Files\\KII Transcripts\\ > - § 3 references coded [0.45% Coverage]

Reference 1 - 0.18% Coverage

Interviewer: Length of questionnaire? -too much or too little

Respondent: I think to me they are appropriate.

Reference 2 - 0.16% Coverage

Interviewer: But this is not crime.

Respondent I know. This is no crime it still matters a lot.

Reference 3 - 0.11% Coverage

Respondent: I think the information being given for now is still okay.

<Files\\KII Transcripts\\ > - § 3 references coded [2.29% Coverage]

Reference 1 - 0.23% Coverage

Respondent: The length is appropriate. And then each question is apt, direct to you know…

Reference 2 - 0.24% Coverage

Respondent: All the questions are appropriate and they should be acceptable for the adolescent.

Reference 3 - 1.82% Coverage

Respondent: The potential benefit is…I mean one of it is that it'll enable us to know those who have high risk or low risk. And those ones that will require immediate intervention. So this will enable us to know and it'll also give us an insight into…so we can know for how long an individual has been having mental health issues. Then it will also give us a picture to what goes on within an individual social milieu. It will also let us know a little be about the family dynamics. It will also let us know whether there has been some psychosocial issues within the family. It will enable us to know whether there has been any form of physical abuse or emotional abuse or parental neglects you know and all that.

<Files\\KII Transcripts\\ > - § 3 references coded [1.39% Coverage]

Reference 1 - 0.32% Coverage

Respondent: It's a good questionnaire. And I think it'll go a long way to get the real information about how to view or how to know if an adolescent is really fighting depression

Reference 2 - 0.37% Coverage

Interviewer: Length of questionnaire? -too much or too little

Respondent: It's appropriate.

Interviewer: Are the questions acceptable and appropriate?

Respondent: They are appropriate and acceptable.

Reference 3 - 0.69% Coverage

Interviewer: What additional information would you like to know for someone who has high risk or low risk?

Respondent: of course I think it's appropriate because if you feel you are on high risk of depression, you can go through this and then ask yourself that question and see if is right. Or you go through that site, definitely you'll visit a professional. I think it's okay.

<Files\\KII Transcripts\\ > - § 2 references coded [17.83% Coverage]

Reference 1 - 13.50% Coverage

Interviewer: Part of our research is, we're trying to develop what we're calling a risk calculator. It's being designed for adolescents to self-assess themselves and see if they're high risk for depression or low risk for depression. So we'd like to go through it with to and at the end you let us know what you think about the… so this is our risk calculator, this is the prototype, it's designed for adolescents at the moment. We've the option of translating to other languages, for starters that's one, gender, age, we'd add other options depending on how…people's opinion is varied now, ethnicity would adopt for each country. For us now it won't be white of black, it'll be maybe Yoruba, Igbo, hausa, all around. Erm the first section will be family experiences, because we think that the family is an important factor. How is your relationship with your mother?

Respondent: Good.

Interviewer: No without answer, I just want you to go through it with me. How is your relationship with your father? The adolescent will pick which ones. How is the relationship between your parents? Have you ever been separated from your parents for a long time and cared for by someone else? Have you ever seen adults in your home fighting with each other or fighting the children in your home? Did someone in your family or household ever beat you in a way that left marks or required medical care? Of course the adolescent will pick which one. Have you ever run away from home for more than one night? Have you ever not had enough food to eat at home or had to wear dirty or torn clothes because you didn't have any other clothes? Have you ever thought or felt that your parents wished you were never born? Have you ever thought or felt that someone in your family hated you? So that's for family experiences. School experiences… in a usual week do you meet up with friends to chat or do things together? Have you had to drop out of school or repeat a grade? In the past year have gotten into a physical fight in which someone got hurt? Other

experiences, did someone ever try to touch you in a sexual way, or asked you to touch them against your will? Have you ever drank alcohol, smoked cigarettes or marijuana or used any other drugs? So depending on what that person has answered, it'll show whether high risk or low risk. This section will show you what to do, it's just an option now. Research has shown that items you answered are associated with future risk of depression. If any of the question you responded to positively are causing you distress, it is possible to seek help. You can talk about your distress with an adult you trust, a school counsellor, or a health professional. So you can contact, of course we would have a helpline, but a proper website. To learn more about symptoms, prevention, treatment go to…this is another website, so this website will contain adequate information about what to do, what symptoms and that. If you are at low risk; research has shown that you have low risk of depression. With low risk, it is important to take care or your mental health and well-being. You can go to this website to learn more about healthy sleep, we want to ensure that even if you're high risk or low risk, you get access to information that is good for you as an adolescent. If you know someone with several risk factors in this survey, encourage them to seek help from and adult they trust, health worker, or a school counsellor. So this our risk calculator that we've developed so far, so we'd like your opinion. What do you think about this risk calculator? Do you think that the length of questions is okay or it's too long or it's too short?

Reference 2 - 4.34% Coverage

Respondent: It's too short.

Interviewer: It's too short. So you think that we should add more questions?

Respondent: Yes.

Interviewer: Okay, can you give us an example of questions we should add?

Respondent: As in, in the family this thing, it still needs to explore more on the siblings not only parents alone. Siblings too can cause this thing, they can be rivals at home so we have to know the levels of their relationship with siblings in the extended family, because abuse, first this thing…the risk fact is your family, the uncles, the whatever that you put at home. "How close is the relationship between your child and other family members that are staying in your house?" The school, the teachers…the school authority they needs to be involved in it. Is not erm…in bullying the child, in punishment, there are to be corporal punishments and this thing so that the risk factors will be low the school. The school too should be explored.

Interviewer: We should ask more questions about…

Respondent: The school yeah.

Interviewer: Do you think these questions are appropriate for use by adolescents?

Respondent: Yes, it's a simple question for adolescents to use.

<Files\\KII Transcripts\\ > - § 7 references coded [3.99% Coverage]

Reference 1 - 0.09% Coverage

R: you didn’t talk about neglect and abandon

Reference 2 - 0.25% Coverage

R: it’s okay because if you give them bulky questions they will get tired and might not even provide the right information

Reference 3 - 0.91% Coverage

R: there are acceptable and appropriate but like I told you earlier it’s not all children that are in school. So for the ones who are not literate how do you want to go about it because for the age you started from14 to 17, we have some 12 year old learning one trade or the other. If the age is not there they will they will tell you is not for them so let’s capture our age from the age we are working with. That will even help in our statistics.

Reference 4 - 0.20% Coverage

R: hmm let me scroll down, especially the question for home and err. What of your other siblings

Reference 5 - 0.92% Coverage

R: yeah because some parents tend to show love to one without the other one. Is it cordial? Do you love yourselves? Can you … what are your relationship? Under there, can you share secret with your siblings? Can you tell your sibling what is going on with you? You know so many people they have friend they tell outside even when they commit abortion they prefer to confide in their outside than their siblings. They ask relationship with your parents?

Reference 6 - 0.13% Coverage

R: can’t we ask are you being brought up under a single parent

Reference 7 - 1.49% Coverage

R: yes is a single parent is it he man that’s taking care of the children or the mother that’s is err have custody of the children. Then another thing that we can ask are you allowed to also contribute to issue in your home that means do they carry you along. You know there are some homes like that, no matter how small they are you want to travel? Where do you want to travel to?? you want to do this how should we do it? Do you like it? Carry them along. That will also

build a healthy relationship and good mental health. They tend to develop their brain, you bring your own suggestion. Do they go for recreation, maybe picnics you understand, during Christmas or do your parents take you out. That also brings about bonding.

<Files\\KII Transcripts\\ > - § 3 references coded [2.86% Coverage]

Reference 1 - 1.34% Coverage

R: No I think the length is okay because when it becomes too long it may become boring for them. The only...i wanted to comment on e'en the house, the family aspect, you know people that abuse are usually close to the family. So if the person (stutter)….the adolescent you asked if he or she has been away for a long time. i just wanted to ask if he/she has been neglected inside the house. Many of them, they just lock them in and say okay stay at home you can't go anywhere. Finish this and there's nobody to actually, no company to keep in the house. There are many of them like that

Reference 2 - 1.26% Coverage

R: no, you know, they've lived...how did they treat you, where you were and even inside the home, some people are in the home but they are neglected in the home. Take for example if you go to Parkview, most of them don't come out of the house. They are always inside. If they have to go out, the driver takes them out but they are always inside. Nobody to relate with, maybe the house helps. Some don't even have but they are in the house. Have they left them in the house for a duration? How long have you been alone in the house? That was my thought.

Reference 3 - 0.25% Coverage

I: do you think the questions are acceptable? Do you think they are appropriate for that age group?

R: yes yes

<Files\\KII Transcripts\\ > - § 4 references coded [2.04% Coverage]

Reference 1 - 0.57% Coverage

Respondent: It’s actually good but in terms of our own settings, I believe we should include the environmental factors in terms of where they live which is very important. Then being Nigeria is a religious society, so we should actually add the religious factors into it. So those are the two aspect I believe…

Reference 2 - 0.15% Coverage

Respondent: It is actually okay but I believe more questions can be added to it.

Reference 3 - 0.25% Coverage

Respondent: there's no uhm… how would I put it? The respondent can easily fill this without hiding any information so it's actually okay.

Reference 4 - 1.06% Coverage

so I believe this risk calculator has to be assessable to everybody. Once you notice that this is what you going through, you yourself might actually believe okay let me actually seek help. I think individuals should be educated on who psychiatrist is, who a psychologist is because the moment they hear psychiatrist they'll be "hmm hmm not in our family, we've never visited this" they are always scared. I think hearing a psychologist is even more acceptable or a guidance counsellor, a social worker is more acceptable, but once they just name that, okay you've lost it.

<Files\\KII Transcripts\\ > - § 2 references coded [7.87% Coverage]

Reference 1 - 7.11% Coverage

Interviewer: so there are 7 sections. First section is your sociodemographic background. You know I said it's between UK, Brazil, Nepal and Nigeria. So you pick what country you're from, your age, gender, ethnicity. The second section talks about family experiences. How is your relationship with your mother? How is your relationship with your father? How is the relationship between your parents? Have you ever been separated from your parents for a long time, and cared for by someone else? Have you ever seen adults in your home fighting with each other or fighting with children in your home? Did someone in your family or household ever beat you in a way that left marks or required some medical care? Have you ever run away from home for more than one night? Have you ever not had enough food to eat at home or had to wear dirty or torn clothes because there were no other clothes? Have you ever thought or felt that your parents wished you were never born? Have you ever thought or felt that someone in family hated you? Section 3 talks about the school and social experiences, so in a usual week do you meet up with friends to chat and do things together? Have you had to drop out of school or repeat a grade? In the past year have you gotten into a physical fight in which somebody got hurt? Section 4 now talks about other experiences, Did someone ever tried to touch you in a sexual way or ask that you touch them against your will? Have ever drank alcohol, smoked cigarettes or marijuana or used any other drugs? Section 5 is now where uhm, based on the answers you have choose above, you're graded into into high risk for depression or low risk for depression. Section 6 now gives you like a small talk on high risk for depression. So it says that research has shown that items you answered are associated with a future risk of depression. If any of these questions you responded positively to are causing you distress, it is possible to seek help. You can talk about your distress with an adult you trust, a school counselor or a health professional. You can also contact the following numbers. The same thing goes uhm, someone that has low risk for depression. It's; research has shown that you have low for depression. With low risk, it is

important to take care of your mental health and well-being. And so on and so forth. What is your initial response to the risk calculator?

Respondent: it's okay.

Interviewer: what do you mean by it's okay?

Respondent: it has been properly designed you know. To get the basic answers that are required.

Interviewer: length of questionnaire? -too much or too little

Respondent: it's appropriate. It's not to long. It shouldn't be longer than this.

Interviewer: are the questions acceptable and appropriate?

Respondent: yes they are appropriate.

Interviewer: are they acceptable?

Respondent: yes they are acceptable.

Reference 2 - 0.77% Coverage

Interviewer: we will definitely put that into consideration. Thank you very much. What additional information would you like to know for someone who has high risk?

Respondent: it is okay.

Interviewer: what additional information would you like to know for someone who has low risk?

Respondent: it is perfect.

<Files\\KII Transcripts\\ > - § 3 references coded [2.54% Coverage]

Reference 1 - 1.13% Coverage

Respondent: eh, to me it is very innovative.it is very innovative because it gives at someone to actually know the level. The level of or the, the level of depression. It kind of helps you know. Or if you're not depressed, if it something that can develop earlier or sorry later in life. So this will take you through and. You'll know that, oh through these my answers here, I can be sure I am not depressed or I can be sure I'll not be a depressed person. Even in the future.

Reference 2 - 0.81% Coverage

Interviewer: okay. So, do you think the questionnaire is too long, is it too short or is it just appropriate?

Respondent: is appropriate. Is not much.

Interviewer: okay. Do you think the questions are appropriate and acceptable? Or do you think we should change some of the questions?

Respondent: to me everything I have seen here is okay.

Reference 3 - 0.59% Coverage

Interviewer: okay. So what additional information do you think we should add for high risk or low risk individuals?

Respondent: to me, I think everything is there. The possible outcome, then how you can reach them, then website. Everything is okay.

<Files\\KII Transcripts\\ > - § 6 references coded [1.01% Coverage]

Reference 1 - 0.41% Coverage

Respondent: It's a good one. It's very good. It's very nice. Interviewer: Why is it good? Why is it nice? Respondent: Because the err...those questions there, they are actually survey questions that if at the end you were able to he quite a number of a particular questions, that means you're able to identify people with high and low risk

Reference 2 - 0.08% Coverage

It's not too short, it's not too long. For me it's just appropriate.

Reference 3 - 0.06% Coverage

They are appropriate questions. Yes there are...

Reference 4 - 0.08% Coverage

Of course yes. No problem. And the language there is very very easy.

Reference 5 - 0.09% Coverage

So there's no problem with language understanding either? Respondent: Yes.

Reference 6 - 0.29% Coverage

For me it is okay, because most people get to... especially when it comes to filling questionnaire and it is more cumbersome they won't read it. So the feedback or the err risk factors there's not too much and not too short. Just appropriate.

<Files\\KII Transcripts\\ > - § 3 references coded [0.30% Coverage]

Reference 1 - 0.07% Coverage

Respondent: I think it's well covered. The questionnaire is covered

Reference 2 - 0.10% Coverage

It's okay. The questionnaire is well covered, all the areas about depression was taken care of.

Reference 3 - 0.13% Coverage

Interviewer: Are the questions acceptable and appropriate? Respondent: Yes. These are the things that they face in the society.

<Files\\KII Transcripts\\ > - § 4 references coded [1.76% Coverage]

Reference 1 - 0.81% Coverage

I think it good.

Interviewer: can you expatiate?

Respondent: I think is actually, uhm, the questionnaire has been able to you know, draw out the questions in areas that could make a child depressed. And those answers the child will answer to points and well calculate well, making depression in Nigeria...

Reference 2 - 0.14% Coverage

it is not too long, it's accurate, it's not too short.

Reference 3 - 0.26% Coverage

are the questions acceptable and appropriate?

Respondent: yeah I think they are okay. Yes they are.

Reference 4 - 0.55% Coverage

what additional information would you like to know for someone who has high risk?

Respondent: no

Interviewer: what additional information would you like to know for someone who has low risk?

Respondent: no

<Files\\KII Transcripts\\ > - § 4 references coded [2.43% Coverage]

Reference 1 - 0.83% Coverage

You said there is contact for whoever is at low risk and whoever is at high risk. I feel it is okay. If you feel you fall into any of those categories you can seek help. All we want to do is to eradicate depression so that there will be a stop to this killing or err suicide.

Reference 2 - 0.35% Coverage

Interviewer: Length of questionnaire? -too much or too little

Respondent: No it is okay. The questionnaire is okay.

Reference 3 - 1.02% Coverage

: No there's no issue with the questions. But there's even actually one there that I feel it's good to ask. The one that has to do with when you were growing up maybe there was actually somebody that tried to touch you or tell you to touch them. It's actually happening and I want people to answer such questions so that they can be helped.

Reference 4 - 0.24% Coverage

Respondent: Like I said earlier, the question is okay. The information is okay.

<Files\\KII Transcripts\\ > - § 1 reference coded [2.21% Coverage]

Reference 1 - 2.21% Coverage

yes it is. they are very good, at least by the time a child answer those question [stutters] correctly and adequately, as a counsellor or health officer or whatever, you will be able to design to know the type of depression such a child is having either low or high

I: or high risk

R: but in that question I noticed there is a particular place where you said have they ever slept without eating?

I: is it possible that they have not had enough food to eat?

R: to eat or

I: or you have had to wear

R: it should be two question in a one

I: so you feel that we should split that question into two

R: yes

I: ask about the food separately from the clothes

R: the clothes because some child can be well fed but

I: they do not have clothes

R: they do not have cloth doesn’t mean, they will have, they can be well fed, they might not have clothes but doesn’t mean they will wear torn clothes and some child might not be well fed at home and they will not have clothes, it can be both. But in most of it, it’s always, it should be separate because majority of our parents strive hard to give, to feed their children than buying them clothes.

<Files\\KII Transcripts\\ > - § 6 references coded [4.68% Coverage]

Reference 1 - 0.74% Coverage

What I think about it is...it's like a questionnaire trying to get some traits that will manifest whether one has a high tendency of being depressed in future or low tendency of being depressed.

Reference 2 - 0.33% Coverage

From the questions I have seen it is a good clue to identifying one that is depressed.

Reference 3 - 0.47% Coverage

It's not too much. I think you've talked about the family, the school, but you didn't talk about the religious aspect of it

Reference 4 - 0.63% Coverage

under the religious aspect of it like, maybe "have you experienced violence in the church?" "Have you been demoralized in church by the sermon or those around you?"

Reference 5 - 1.91% Coverage

Interviewer: So are there any other questions you think should be added apart from the religious aspect?

Respondent: The social aspect in the society. Maybe you talk about the stigmatization. "Have you been stigmatized?"

Interviewer: Stigmatization of what are we asking about now?

Respondent: Class status. Then even bullying "have you been bullied?"

Interviewer: So bullying is one of the risk factors?

Respondent: Yes.

Interviewer: Are the questions acceptable and appropriate?

Respondent: Yes.

Reference 6 - 0.61% Coverage

Interviewer: What additional information would you like to know for someone who has high risk or low risk?

Respondent: It's appropriate. I think they are okay.

<Files\\KII Transcripts\\ > - § 5 references coded [1.94% Coverage]

Reference 1 - 0.18% Coverage

Respondent: actually some of them are what actually leads to depression. Especially for children. It's is good.

Reference 2 - 0.31% Coverage

Respondent: they are not too long, but we can also... I didn't see anything about peer, peer influence or their relationship with people. Or maybe they've been exposed to drugs and all that.

Reference 3 - 0.15% Coverage

Interviewer: do you think it can be easily used?

Respondent: of course. This is very simple.

Reference 4 - 0.24% Coverage

Interviewer: do you foresee any challenges for using this questionnaire? If so how might we address them?

Respondent: at all. Just yes or no answer.

Reference 5 - 1.06% Coverage

Interviewer: what additional information would you like to know for someone who has high risk?

Respondent: that was I said try to put more of peer.. you know children stay, although they stay well with their parents. They have more interactions with their peers.

Interviewer: what I'm asking is that this information on high risk for depression, is this okay or should we add more information on high risk for depression?

Respondent: that is what I am say now. The question will determine whether you're going to add more. You get now. If you have more of questions that relate to their interaction with their peers, i think it will now affect the...

<Files\\KII Transcripts\\ > - § 2 references coded [0.58% Coverage]

Reference 1 - 0.31% Coverage

Respondent: It's okay, there's no need for adding any question or anything on it. Is okay.

Reference 2 - 0.27% Coverage

Respondent: Yes. They are self-explained, it's simple and English. It's okay.

<Files\\KII Transcripts\\ > - § 2 references coded [0.94% Coverage]

Reference 1 - 0.53% Coverage

R: It’s average. I think more caution should be added to it actually to get accurate something. Well I think this one is random question.

Reference 2 - 0.41% Coverage

R: Depression? Apart from all these ones here? Family background is there. I will say more on peer groups.

<Files\\KII Transcripts\\ > - § 2 references coded [11.03% Coverage]

Reference 1 - 8.70% Coverage

Interviewer: Did someone in your family or household ever beat you in a way that left marks or required medical care? Have you ever run away from home for more than one night? Have you ever not had enough food to eat at home or had to wear dirty or torn clothes there were no other clothes to wear? Have you ever thought or felt that your parents wished you were never born? Have you ever thought or felt someone in your family hated you? So school and social experiences, in a usual week, do you meet up with friends to chat and do things together? Have you ever had to drop out of school or repeat a grade? This is other questions, did somebody ever touch you in a sexual way or asked you to do them against your will? Have you ever drank alcohol, smoked cigarettes or used any kind of substance? So now after answering all that, depending on the answers, it would either show high risk or low risk. If it's high risk, this is what it says; research has shown that items you answered are associated with future risk of depression. If any of the questions you responded positively are causing you distress, you can seek help, you can talk about your distress with an adult, someone in school, or a health professional. So IDEA will have our own helpline here, and then if its low risk for example, it will show; research has shown that you have low risk for depression. With low risk, it's still important to take care of your mental health and well-being. You can go to… we have our own website that has details about how to maintain healthy lifestyle.

Respondent: You're not going to ask any questions about family history of depression? Because you don't it's a…

Interviewer: No this is how our own idea. We're saying now that what do you think about this calculator? Are there things that we should add, remove from the risk calculator?

Respondent: Ehn that's why I am asking whether you would want to consider adding a question about family history. Have there ever been anyone in the family that has been you know…

Interviewer: What do you think about the questions? Do you think the length is too much or too little?

Respondent: No, they're easily understood.

Reference 2 - 2.32% Coverage

Interviewer: So you think it's better for the adolescent is the one … alright uhm, do you foresee any challenges for using this risk calculator?

Respondent: Just the regular ones of people fearing stigma and not answering truthfully. And you're more likely to get the truth if they self-administer, if somebody else is administering it, they will not tell you the truth. The other thing, it doesn't contain any symptom; do you feel hopeless or helpless or do you feel like suicidal or, I think there are things that you might want to find a way to phrase and put in I don't know.

<Files\\KII Transcripts\\ > - § 4 references coded [8.18% Coverage]

Reference 1 - 1.67% Coverage

Interviewer: So erm, since we've looked at what the risk calculator looks like, that is the prototype, I would like to know what your initial response to the risk calculator is. What do you think about it?

Respondent: Well, I think it's good. Most of the questions are appropriate for adolescents but as we were looking at it, I pointed out some things that might not be appropriate for this environment and we need to ask more environment appropriate questions.

Reference 2 - 2.16% Coverage

Interviewer: Okay. So do think that, in terms of you as a clinician, do you think it's appropriate, do you think it's acceptable, do you think it's something that can use in your clinic for example?

Respondent: I think so, very much so especially if it's something that has been validated, I think it's something we can use and coincidentally, those are most of the questions I ask my adolescents, you know, their relationship with their parents, maybe not so much but you'll have an idea when you're looking at them saying it. But yes I think it's something that can be used in the clinical setting.

Reference 3 - 2.78% Coverage

Interviewer: Is there any other thing that you think we should have asked the high risk person or anything that we should add to someone who is low risk?

Respondent: I can't think of anything right now on top of my head.

Interviewer: What additional questions should we add that would better our chances of identification?

Respondent: Did you ask about bullying in school?

Interviewer: No, we just asked about if they've gotten into a fight. But we didn't ask about that.

Respondent: So maybe that's my deal because they wouldn't always fight but it's just that everybody is playing with each other and they're not playing with them or somebody is teasing them or making fun of them, so that might be something we can coin. I don't know how to phrase that sentence.

Reference 4 - 1.57% Coverage

Interviewer: But you think asking about bullying specifically should be included in the risk calculator?

Respondent: Yes, I think so and also how many children are in the family. Reason being that if they are the middle child or I don’t know, if there are more boys in the house than girls. Maybe she’s the girl that does everything, I don’t know. Maybe position in the family if that is a risk factor, yeah. Did we talk about polygamy?

<Files\\KII Transcripts\\ > - § 6 references coded [12.78% Coverage]

Reference 1 - 4.15% Coverage

Interviewer: Do you have any issues with any of the questions?

Respondent: yes. why are they close ended?

Interviewer: oh! I can't answer that actually, because this is just a prototype. So you have a problem with the fact that they close ened? What would you have preferred?

Respondent: you sometimes, people just answer what you want them to answer. Especially when you have close ended questions. So I just tell you no. I know there is nothing here -----. So erm, maybe you prefer some parts of it to be written. The adolescent should be able to express themselves. Because you know most times We still get to know if an adolescent is at risk is already depressed by what the child writes. So I think, I don't know if there are other provisions for things like that.

Interviewer: that's why I am asking you. We'll take that into consideration. Are there any other questions you'll like to add or these questions okay?

Respondent: they are okay. So it don't look like probing to much.

Interviewer: and social experiences. In a usual week do you meet up with friends to chat and do things? Have you had to drop out of school or repeat a grade? In the past year have you gotten into a physical fight in which somebody got hurt? So is this fine?

Respondent: yes. There are.

Interviewer: the questions are appropriate? Should we add more questions? Do you have any other questions you think we should add under this school and social experiences? You don't have to o but if do, is really like yro hear it.

Respondent: (laughs) okay. Erm.. you're asking do you meet up with friends? Why not start with do you even have friends?

Reference 2 - 2.20% Coverage

Interviewer: (laughs). So section 4 talking about Other experiences, Did someone ever tried to touch you in a sexual way or ask that you touch them against your will? Have ever drank alcohol? Smoked cigarettes or marijuana? Or did you used any other drugs? So is this fine?

Respondent: yeah it's fine, but...okay well, looking at 10 to 18. Okay it quite...

Interviewer: are you sure? You are not sure. I don't think ...what problems do you have with this?

Respondent: the part of touching you in a sexual way...

Interviewer: no we looking at risk factors. That can be a risk factor for a child getting depressed. Sexual abuse.

Respondent: yes. True but, okay..but they should be able to understand what you actually mean there.

Interviewer: then section 5 is now where we calculate based on what you scored above. If the adolescent has a high risk or low risk.

Reference 3 - 3.47% Coverage

Interviewer: then for the low risk, it just shows that research has shown that you have a low risk for depression. With low risk, it is important to still take of your mental health and well-being. You can go to www. ------ to learn more about healthy sleep, diet and behaviors. So is this also fine?

Respondent: yes.

Interviewer: so now what is your first impression of this calculator? What was the first thing...is it a good tool? Are you impressed by it or what do you think about the calculator?

Respondent: it's okay hmmm, I think it's quite shallow.

Interviewer: okay. In what sense?

Respondent: like the questions are the very normal everyday questions. Really there are sometimes they could mixing in the life of someone and yet the person doesn't come down with depression. I was thinking there were going to be like real pointers like maybe... looking at the symptoms of depression.

Interviewer: we are not looking for a child that is depressed, we are looking for a child that is at risk. So it is the risk factors that are there.

Respondent: yes but if I am always sad but I am not depressed. I am just sad, not depressed. So you should be able to explore why am... always sad. It doesn't mean that I'm depressed already. Like I know growing up I used to feel the moody but I am not depressed. I wasn't really a very cheerful person. It could be a

Reference 4 - 1.92% Coverage

Interviewer: risk factor

Respondent: yes. And maybe because I got lucky I started checking up all those things. Do you understand? But not everybody will be..

Interviewer: understand that

Respondent: yes. So I think maybe one or two things that really have to do with depression should...not really that we'll look for all the signs and symptoms but just one or...two or.

Interviewer: two questions

Respondent: yes. All those low moods, do you just find yourself crying for nothing. Some of us sit down...will just get hungry to cry, just feel like crying

Interviewer: (laughs) said hungry to cry...

Respondent: (laughs) there are times you feel that way. It doesn't mean you're depressed. But it could actually lead to something. Do you understand?

Reference 5 - 0.95% Coverage

Interviewer: I get your point.

Respondent: so I think we should include one thing about depression.

Interviewer: so what do you think about the length? Is it too long or is it too short? Or is it perfect?

Respondent: it's perfect because they might not, you know aside the fact that they have too much energy to burn, they might not really want to you to take their time.

Reference 6 - 0.10% Coverage

Respondent: yeah. They're appropriate.

<Files\\KII Transcripts\\ > - § 1 reference coded [1.39% Coverage]

Reference 1 - 1.39% Coverage

Respondent: You know is in 2 ways, of course I want to believe they are not going to do it and abandon it. At least since there is a helpline for them to contact. And ones they've noticed that okay I'm high risk for them have taken this question, go through it, they want to believe that okay I need help. So the helpline and the website provided there will make them do good.

<Files\\KII Transcripts\\ > - § 6 references coded [5.66% Coverage]

Reference 1 - 0.10% Coverage

Respondent: why is mother the first one?

Reference 2 - 0.26% Coverage

Respondent: Yes. And because you know you have to be very cultural…I know that you're trying your best

Reference 3 - 1.69% Coverage

I've seen a family that the school teacher or a lesson teacher was molesting the child. They told the mother, the mother said "because that person is in my church", they covered everything up. And you, so that's what even makes the child like "Wow! Because you just want to cover this up" So there's a lot of issues, but somebody has to start something from somewhere, you don't always have to follow what is the norm. We can actually do these things and let people now you know fall behind. It's not a bad thing yes why not, I don't think anything should be changed in that end point. I think you should stand on your standard and I think people will fall in line.

Reference 4 - 1.16% Coverage

However, in the family experience, I think there's one question that's okay, when you asked "Has anybody asides from your immediate family members live with you for a particular period of time" because you find out that even as a result of these interviews you see that a family member will comes from the…for 1 month, 6 months, 1 year and they come and destroy the life the children. They molest this children, so I think it's also a very viable question.

Reference 5 - 0.90% Coverage

Then this school experience, sorry can you go back to that school there's something that…ehn ehn now you can ask that "Do you like the school that you're attending?" or "Do you like going to school" I'm peculiarly with that school, because that child might be experiencing a lot of issues in that school that might lead even to the depression in itself.

Reference 6 - 1.54% Coverage

Respondent: Yes, because they're not very blunt. I think there are things that have direct answers so they don't need to think too much about it. The only place where they might have problem is where you asked "has anybody touched you?". I think you should have the validity scale where you'll know if this person is telling the truth so if the person has answered like 8 in a particular way and now goes no in that, we can query it. I don't know if you understand because most people will not answer.

Interviewer: They won't want to tell the truth.

Respondent: They'll not, I can tell you that for free.

<Files\\KII Transcripts\\ > - § 2 references coded [1.77% Coverage]

Reference 1 - 1.04% Coverage

Respondent: Yeah I was going to ask how many questions are you…because when you --- from adolescents, they just want something very short. At the same time, I actually wanted something that maybe that could be included, maybe change of environment. "do they tend to move from one place to another, a lot?" or change of school.

Reference 2 - 0.72% Coverage

Respondent: Yes. Grief! If they've experienced loss of someone close to them or loss of pet as well, although that might not be the African way but loss of someone close to them or change of environment like I said earlier on.

<Files\\KII Transcripts\\ > - § 7 references coded [9.01% Coverage]

Reference 1 - 2.51% Coverage

Interviewer: Okay. So erm, what do you think about the risk calculator?

Respondent: I think it's good and I see that it covers to a large extent, experiences erm, around abuse, drug use. My concern is that erm, how easy or how feasible would it be to elicit abuse and drug use but of course it's anonymous so it should be easier. Is there a way the questions can be rephrased?

Interviewer: Can you suggest something to us?

Respondent: Off the top of my head right now, I can't come up with a very smart suggestion but I can think about it and get back to you. What I do when I see adolescents and I try to get sexual history, history of erm, induced abortion and the likes, the pregnancy history and all of that. From adolescents, what I do, I try to be very direct and I ask them "so how many abortions have you had now?" and so there won't be an opportunity to lie. It works like magic all the time and sometimes suggest "5 times abi?" and then they'll say "no, no, no, just once". So I really do not know how to put it that kind of

Reference 2 - 0.72% Coverage

Interviewer: you think we should reword these two questions?

Respondent: I think so in order to be able to elicit…'cause response bias is a going to be high for this too. A d erm, the tendency to just give you what you want to hear, to say no "hmmm, no" their co-essence it going to be quite high.

Reference 3 - 0.43% Coverage

Interviewer: Do you think these questions are acceptable or they are appropriate for adolescents?

Respondent: Yes, they are, but how do you elicit a honest answer is my concern.

Reference 4 - 0.58% Coverage

Interviewer: Do you think there are any challenges in using this to identify depression in adolescents. If there are, how do you think we can overcome them?

Respondent: The only challenge like I mentioned earlier is the response bias thing.

Reference 5 - 0.53% Coverage

Interviewer: where you think that people will not… the response will not be truthfully.

Respondent: Yes for some sensitive items here. And there are some I feel that you could get informants erm, opinion here. Like get

Reference 6 - 2.96% Coverage

Interviewer: rather than asking yes or no, you want them to…

Respondent: No. like for instance, this same tool, could be completed by an informant, on behalf of … so this is the teacher saying "this is what I feel", "this is my opinion about this child". Has he dropped out of school, has he being in a fight, or a parent," has he stayed away from home" parents can actually tell you that if the child has stayed away from, has spent nights away from home. Have you ever run away from home for more than one night? Parents can answer a yes or no to that. Again in an environment where parents tend to always pick good on the children, they may also say no. So that they don't say you're not raising your child well. The challenge is getting honest answers.

Interviewer: Do you think there are any questions we should have added that we didn't ask?

Respondent: I don't know if I missed erm, family history of depression.

Interviewer: no we didn't do that. You think we should ad that too?

Respondent: Yes. More questions that are indirect. Maybe that try to show erm, behaviour. “are you usually angry” erm, apart from “are you moody”, erm, interpersonal relationship with others in schools, maybe I missed that as well.

Reference 7 - 1.26% Coverage

Interviewer: No, we just asked if erm, his had fights, does he meet up with friends.

Respondent: Yes. Then the bullying bit too.

Interviewer: We should ask about bullying here?

Respondent: Yes, I missed that as well.

Interviewer: okay.

Respondent: “How often do you and your friends enjoy hanging out with drinks” looks like nice not like a direct question. You’re on the spot, if I say yes to yes to this you’re going somewhere, you’re going to be punished. You’re going to be exposing yourself.

Interviewer: Okay.

<Files\\KII Transcripts\\ > - § 5 references coded [6.95% Coverage]

Reference 1 - 1.00% Coverage

Respondent: Well I think it’s okay. However, there are some areas that I think maybe you need to add one or two things. One, the classification should have been “High, Moderate or Low risk”. Then two there is one part that you said “Have you ever thought you were never part of the family”

Reference 2 - 2.44% Coverage

Interviewer: “Have you ever wished or thought or felt that your parents wished you were never born?” or “thought or felt that someone hated you”

Respondent: I think that one should be added that you were born in another family

Interviewer: Okay. That means the child, thinking wishing that he belongs to a family not…

Respondent: Not particularly that one. Because I’ll actually experience one like that, a child in the UK. And I was there and told the mom like “I wish I was not born into this family.” That was the way the child thinks at that time. He said it out, I heard him and I was like “wow!”. I felt like entering the ground that day. Because erm, she was actually my sister. So I think you can…

Reference 3 - 0.67% Coverage

Interviewer: It has to be rephrased.

Respondent: Yeah rephrase or added to…

Interviewer: As a separate question?

Respondent: Yeah. As a separate question. Apart from that, everything is fine.

Reference 4 - 0.49% Coverage

Interviewer: Do you think these question will be acceptable and do you think they are appropriate for the age we're targeting?

Respondent: Yes

Reference 5 - 2.35% Coverage

Interviewer: they might not be need for translation. Okay. Do you think they're any other questions we should ask for somebody who, do you think there is any other factor we didn't talk about for somebody who might be high risk for depression?

Interviewer: Do you think there are other questions we should ask?

Respondent: How about the use of drugs? Is it…

Interviewer: Yes, it's there.

Respondent: No problem, but these things I think they can be used now. However, over time you might still find one or two things when you start using them. You might not be able to identify it now, but whatever it is, you can have a modified version to update the current one you're using.

<Files\\KII Transcripts\\ > - § 6 references coded [11.13% Coverage]

Reference 1 - 1.35% Coverage

I: Okay, so we're going through the risk calculator and have you ever had or not had enough food to eat, or had to wear dirty or torn clothes because you had no other clothes to wear?

R: So I'm asking that why torn clothes? What's the issue with torn clothes?
I: Don't you think it's an indication of poverty?

R: Is poverty an indication for depression?

I: Maybe a risk factor

R: When I think about risk factors for depression, it gives us risk and protective factors. I'm not sure poverty is one of them.

Reference 2 - 3.42% Coverage

I: Of low socioeconomic status.

R: Poor people are more depressed than rich people? Think about it. I am sure that's correct. You know, you must be thinking about this because you may wear torn clothes, your parents don't have it but they may be giving you so much love, they're supportive, they're there and torn clothes also have degrees you know. I mean I'm sure if I check maybe I wore a torn cloth at some point in time and I'm not sure I was…do you understand? So as fine as you are I'm sure you must have worn torn you know. So someone endorses that and he gets a score for that. It may be a false thing, so I'm sure. You may want to look for another indicator, if it's poverty, let even talk about poverty. Is poverty necessarily a risk factor for depression? We're thinking about a family history, we're thinking about childhood experiences, we're thinking about family dysfunction, parent child relationships, you know school experiences. Those should be your risk factors. I've heard some of them but you know just think about that but even if you insist you want to put poverty there, then maybe you should find another way that is a bit more universal you know and unfortunately I can't help you. I'm sorry, I can't think of how to rephrase it right now at the top of my head.

Reference 3 - 0.31% Coverage

R: Let's go back to your low risk, is this done? I don't want to just be talking, can you still make adjustment to it?

Reference 4 - 3.51% Coverage

I: Oh yeah, definitely. That's why I said I'm going to ask you questions because we want to know what you think about the questions.

R: I think you need to have a list of you know, like a checklist of risk factors and try and…a comprehensive checklist of risk factor and from those risk factors, then draw out some of these your questions because if you don't work from that, you'd be would be everywhere, you know. Like I didn't hear anything about academic performance or competence. So that's a huge risk factor you know. A child that has been struggling and not feeling at par or whatever, I don't know or not feeling included, did you just change school recently, did just have a recent change in something, any recent loss event or transition, change of schools, I don't know how you're going to rephrase them**,** just make two or three questions about transition, changes going on in the child's life, parent just remarried, mother just died or they just moved to a foreign place, they've been changing schools, have you changed school severally. So I'd think that a good way to do it would be how that say maybe 15-20 risk factors and begin to check them off and have

questions that explore around that and then you can now wait and see how to interpret it to a total score except you have a limit of number of items.

Reference 5 - 1.78% Coverage

I: No, we're trying to, we don't want the questionnaire to be too long but it's important that we actually have the correct questions for the risk calculator so that if even with the high risk, then we're sure that we did ask the proper questions and for the low risk. So even from this risk calculator, do you think if we're able to improve on what we have, it's a good idea to have something like that?

R: Yeah, I think so. It's useful, erm so but you need to then really think about when you tell someone you're high risk, what does that mean to…what value? So we must be thinking of how to utilize that explanation going forward. What do you tell a high risk person?

Reference 6 - 0.76% Coverage

I: So here we're saying now that, this is just a prototype. There's a child line you can call for further questions,

R: child, you're calling an adolescent a child, you know they're not going to like it.

I: there's a helpline that you can call.

R: like a team hotline or something.

<Files\\KII Transcripts\\ > - § 2 references coded [5.41% Coverage]

Reference 1 - 3.26% Coverage

Interviewer: So what do you think about the risk calculator? What is your opinion about it?

Respondent: I think it's good idea, I like the fact that erm it allows us to put indigenous language in it. But I think that you can add more languages, especially because yes we're in the south-west, if we are starting with Lagos I think we should use the 3 major languages initially because there are some people too that erm maybe might be more comfortable with own indigenous language. However, somebody might want to argue that the government is providing free education, so the average adolescent should be able to speak English. But we found out that they are also some for example, some of these Igbo traders some of them can't speak English. So they might actually prefer their own indigenous language. The truth is as free as education is, some adolescents are still not going to school. They are hidden in peoples' homes working as maids, working as you know erm service boys and all of that. So we could erm maybe go an extra mile to get the other indigenous languages.

Interviewer: Length of questionnaire? -too much or too little

Respondent: It's better short because of the attention span of adolescents. They don't have time to fill any long questionnaire they won't give you the…you won't get the appropriate response from them because they get bored easily.

Reference 2 - 2.15% Coverage

Interviewer: What do you think about when we give these adolescents they use the screening tool and then it shows that they are at high risk, do you think that that can kind of tilt that adolescent towards…

Respondent: Depression?

Interviewer Yeah.

Respondent: Oh no I don't think so. I don't think because the child…I don't think so. I think the questionnaire is going to erm...at least, it makes the child a little bit more aware "oh I wasn't thinking in that direction like before but I can have depression in future or whatever" and there are already information put on there to show the child that you can go seek help in this particular place or through this particular means. I think it'll even help the child you know the adolescent to know that okay I'm going to put it at the back of my mind or copy this number somewhere that incase I have need for it in future, I could call up the helpline.

<Files\\KII Transcripts\\ > - § 6 references coded [5.50% Coverage]

Reference 1 - 2.04% Coverage

Respondent: Well, I think erm, with my understanding of child and adolescent erm depression, it's covers erm a significant number of things that we have seen with erm depression. So I could tell that if anyone answered yes to majority of those questions, especially an adolescent, there's a lot of stuff going on with them between conduct problems and we know that there's this uncanny association between depression and conduct problems which I thing I mentioned earlier when I was talking that we do see behavioral issues. So and of course we also saw some of these things about erm trauma, which I also mentioned especially the one that is sexual in nature and the one that involved your primary support system which is the family and all of that. So essentially I think this may have captured fairly well some of the risk.

Reference 2 - 0.22% Coverage

Respondent: Yeah the questions are okay, none of them appeared to be in any way, way off

Reference 3 - 0.11% Coverage

Respondent: It looks pretty straightforward.

Reference 4 - 0.53% Coverage

Respondent: I think there's one area that I would feel that we should…although I was listening but I am not sure whether it is there. Which is things like having to leave with other people who are not your parents.

Reference 5 - 0.54% Coverage

Interviewer: I think there's something like that. "Have you ever been care for by someone else for a long time"

Respondent: Okay. That's the only thing that came to my mind I didn't realize that it was already captured.

Reference 6 - 2.06% Coverage

Respondent: Well, I think that is where we may want to bolster the information a bit more. That the fact that you have the high risk does not mean that you must develop depression. And that the reason why we are having this is so that we can be able to provide you some guide on how to avoid depression. And most people who answer many of these questions are at risk of depression, but that everybody will develop depression. So this is just, just being able to provide more information so that it won't be distressing. And of course the low risk also need to be aware that the fact that you're low risk, that there are people who have been low risk and their risk of depression just sky rocket with just one single life event. So at the end of the day, anybody will still be provided with information that can build their resilience.

<Files\\KII Transcripts\\ > - § 6 references coded [5.82% Coverage]

Reference 1 - 0.24% Coverage

Respondent: I think it's, it addresses a lot of the things that we talked about already.

Reference 2 - 2.70% Coverage

Respondent: I think the other to probably add is, maybe of they're going through blah blah blah, where do you go for help, who do you see for help. Or where do you seek for help from. So if you run away from home for one night, where do you go? Do pass the night out with friends, do you go out with people at the bridge or did you go to another family members house? Erm...if you use substance, alcohol or whatever, who you get them from? From a friend, from a family member, from a guy that's on the road, from the local dealer? How did you know about these things? Did you learn it from the social media? Your friends talk about it and that's how you knew where to get them from...uhm how do you feel about your parents fighting? I mean it's one

thing see your parents fighting or they're separated. Does it make you sad? Does it make you happy? Because both ways it means something. If your parents are divorced and they're fighting, if you're sad about it's normal. If you're happy about it...

Reference 3 - 0.45% Coverage

Respondent: so I think apart from identifying the factors, also trying to understand their own response to those factors will also add to be able to calculate the risk

Reference 4 - 0.21% Coverage

Respondent: eh...they are acceptable and appropriate for people who can read.

Reference 5 - 1.10% Coverage

Respondent: how'd you fix that? And I think it's one of the challenges we still have to deal with. For young people in this ---- we may not coin it risk calculator for depression. You can give it another name, you can say erm..how well do you know yourself? Or Google me, I don't know something fancy, something that will be catchy. Or know yourself or you know something not necessarily pointing to that.

Reference 6 - 1.12% Coverage

Respondent: I mean, they're both important, things like your response to this things, will be very important. So apart from the risk factor themselves, your reaction to those risk factors is very helpful. Because you can have someone who answer towards having a risk of depression, and the person may actually be very resilient and not be, or not suffered depression at any point in their lives grow up you know.

<Files\\KII Transcripts\\ > - § 1 reference coded [3.20% Coverage]

Reference 1 - 3.20% Coverage

R: so for the parents' part like I said earlier, he or she may not have any

I: so what do you think we should ask

R: I don't know, maybe, I don't want to use, are you living with your parents? Are your parents alive? I am just thinking just to see if that parent child. Is it there? Because some of them might be living with their grand mums, step mum

I: some other kind of care giver

R: some might even be in the orphanage that are feeling this and they have a mum there so I don't know how we will put it, slash caregiver slash whatever

I: do you think the questions are appropriate and will get us the information we are looking for?

R: I am not too sure. Like in the social part, there's nothing on use of social media, how long do they spend on social media? Are they exposed to social media? If they take away your phone or whatever will you feel, just to see how connected they are with err…in terms of peer relationship, do you easily get err pressured by people or whatever. I don't know how you are going to coin it but something to do with how they cope with err peer pressure. And I don't know if the question was asked, if you had something really bad that happened to you, something private, who would you talk to? Or nobody?

I: do you have someone you can confide in?

R: when you have something really private and personal you know. Because if you have somebody, but if you now say no argh! That's an issue

<Files\\KII Transcripts\\ > - § 4 references coded [2.94% Coverage]

Reference 1 - 0.71% Coverage

Respondent: It's good I'd have wanted to include err, the area of comparisons especially looking at the Nigerian environment. You'll discover the major challenge is parents comparing one child to another sibling. Or somebody that is doing well, could be in school "look at your mate they are doing this"

Reference 2 - 0.81% Coverage

Interviewer: So we should ask question on that?

Respondent: Yes, that's a question needs to be asked. Especially looking at the Nigerian context.

Interviewer: Length of questionnaire? -too much or too little

Respondent: No no it's okay, it's appropriate.

Interviewer: Are the questions acceptable and appropriate?

Respondent: Yes, definitely.

Reference 3 - 0.24% Coverage

Interviewer: Do you think it can be easily used?

Respondent: Yes, if course. The questions are direct.

Reference 4 - 1.17% Coverage

Interviewer: What additional information would you like to know for someone who has high risk or low risk?

Respondent: I think it is important that the adolescent knows that he needs to go for further care. But my only concern is that word "you are high risk" do you understand, that is my only concern. I'd have rather wanted it to be that "based on the answer that you've given, it is important you go for further care" do you understand. Because my fear is self-stigmatization. That's just my fe

<Files\\KII Transcripts\\ > - § 4 references coded [3.73% Coverage]

Reference 1 - 0.84% Coverage

R: But like errr, no no have you ever run away from the home?

I: Yes for more than one night

R: You said its either yes or no. what about if you tick yes

I: Yes that is what I am saying

R: what is the reason?

I: Okay you want us to ask why

R: Further. yes because that will you know if you probe further that is when you will now know err different reasons why it happen

Reference 2 - 0.61% Coverage

R: Yes, but my reservation is it seems the question is just open or closed. It's either you say yes or no that may not give more information about it, not give you more information about it. So it needs to be, if you give yes, you must give reasons or why it happen like that

Reference 3 - 1.58% Coverage

R: except if it's translate to other languages and it has to be like a supportive err, that will, that will help, that's the way I see it, it will have a have a more or less a guide. Perhaps somebody is there to guide them to do that. For instance now, if you bring, usually have research here conducted on these ones that we have here and most of the time I used to tell them they write all sorts of big grammar and all that coming to them

I: [laughter]

R: and I can assure you err like 40% of them can't read and write because they have been on the streets for long. They have not been to the four walls of the school and all that so that's why you see most of them starting 16 14 15 in nursery class

Reference 4 - 0.70% Coverage

R: you know naturally, if you want to administer all these. You need to give them the confidence and reason why you are doing that. You want to help them and all that. If you give that assurance in the first place, they tend to be relaxed especially if you even give them assurance of confidentiality and all that.

1. Code Query Output

<Files\\KII Transcripts\\ > - § 3 references coded [0.50% Coverage]

Reference 1 - 0.18% Coverage

Respondent: erm..teachers should be put through on how to use it.

Reference 2 - 0.19% Coverage

Respondent: I am not sure of parents. You might not get good results.

Reference 3 - 0.14% Coverage

Respondent: but teachers should be able to use this.

<Files\\KII Transcripts\\ > - § 6 references coded [2.69% Coverage]

Reference 1 - 0.10% Coverage

Respondent: of course the professionals.

Reference 2 - 0.91% Coverage

Respondent: lay man cannot, we need to like do serious erm...break down to make it easily errm... understandable for the lay man, because we are the professionals we can understand, some people will be like ki lo n so gan...ki lo n so...ko ti ye mi (what is he/she saying? I don't understand any of it) you know all these kind of things...I don't know if you understand Yoruba?

Reference 3 – 0.55% Coverage

Interviewer: Do you think teachers and parents should also be allowed to use this calculator for their children or students? Remember you said something about mental health assessment.

Respondent: yes

Reference 4 – 0.54% Coverage

Respondent: in schools, in fact that's why I am....that's what I am just thinking now. That's the best way to make it useful. But...how making that...how do you want to go by it? For example, where do you want to start from?

Reference 5 – 0.18% Coverage

Interviewer: what challenges?

Respondent: ...which is the implementation...

Reference 6 – 0.42% Coverage

Respondent: illiteracy and lack of public support and the state support. There has to be support... everybody must key into this project to make...to make...to enable it work.

<Files\\KII Transcripts\\ > – § 4 references coded [3.66% Coverage]

Reference 1 – 0.66% Coverage

Interviewer: do you think parents and teachers should also be able to use this for the children? Or should only be the adolescents using this?

Respondent: because it’s very simple and can be, they can understand it. I think it should be

Interviewer: fine for the adolescents.

Respondent: it should be.

Reference 2 – 0.73% Coverage

Interviewer: so what do you think are the potential benefits of using such calculator?

Respondent: well, it would help in the identification, the it will make our work easier. Instead of just assuming or just thinking this would give us the idea of what we are looking for. And the...the type of riak we're... whether low or high risk.

Reference 3 – 0.58% Coverage

Interviewer: do you foresee any challenges with using this? With an adolescent using this? Like is the language okay?

Respondent: what I'm looking at, the the...well, think that has been addressed from the demographic level. You Nigeria is a multi-linguistic eh ehn

Reference 4 – 1.69% Coverage

Interviewer: would you think we should do it in another language?

Respondent: no no what I'm saying is...what I'm looking at is, in a situation where by you need to ah, apply it to someone who cannot understand or speak English, how do we do that?

Interviewer: so, maybe we should also do this in the local languages.

Respondent: you know you're not only targeting educated individuals. So if language issue...you understand, ehn ehn..then.. there's uhm, there is one area that talks about the sexual and stuffs like that

Interviewer: yes. That's erm other experiences.

Respondent: did someone ever tried to touch you in a sexual way, or ask you to touch them against your will? Okay, I think that's okay. Just wanted to... it's straight forward and it's not ambiguous.

<Files\\KII Transcripts\\ > - § 6 references coded [5.94% Coverage]

Reference 1 - 1.41% Coverage

Respondent: When it is administered to adolescents in the society, it would make us to know whether such adolescent is a potential somebody that is going through depression or is going to go through depression in future. It has been categorized to high risk and low risk so as to render help on time. Because the reason why the questionnaire will be administered is to render assistance that's the basic reason…because I believe the questionnaire will be administered. Because if at the end of the day, the adolescent in question has answered those questions, at the end of the day after answering all the questions will make us to categorize the risk as low or high. If it is low, definitely just to keep the adolescent informed about depression and all that. But if its high, quick assistance or quick help is needed for such adolescents.

Reference 2 - 0.58% Coverage

Interviewer: Who do you see using this calculator (parents, teachers, health workers?) Where or when would they use it?

Respondent: Exactly. There's no issue. Can also administer it. Then also, those parents, teachers and health workers too should be administered questionnaire too though the questions will be different from all this questions.

Reference 3 - 1.24% Coverage

Interviewer: Do you foresee any challenges for using this questionnaire? If so how might we address them?

Respondent: Yes the challenge I foresee is you know, is not all adolescents that are educated. Some of them will read it and will not understand well, even some of them that are educated they might not understand well that. You know I talked about the age range of adolescents, you now start administering this kind of questionnaire to a 10-year-old boy/girl. The way a 19-year-old boy/girl will be different. So interpretation too matters and how the adolescent understands it. Okay it is the work of the erm interviewer. In this case now, if the adolescent finds it difficult to understand well, then the interviewer breaks it down.

Reference 4 – 1.59% Coverage

Interviewer: You know I said it is supposed to be self-administered. So for adolescents that are supposed to use it themselves, how do we overcome the challenge of them not understanding it if there's nobody to explain to them?

Respondent: On different occasions we've had situation whereby erm questionnaire will be given out and at the end of the day, the respondent will throw it away or will just ask somebody or will just be ticking even without taking time to understand those questions one after the other. So to avoid such, I believe the interviewer should…

Interviewer: So it should be interviewer based not self-administered now?

Respondent: Well it's vice versa. You can self-administer it in a way that if you notice that this adolescent is educated and would understand. In the other way, when you see that this adolescent is not educated of course it shouldn't be self-administered in that so as for us to get positive response.

Reference 5 – 0.63% Coverage

Interviewer: Do you think we should we should maybe produce this questionnaire in local languages also?

Respondent: Exactly. That's one of the things that came to my mind initially. For instance, somebody that does not understand English is only Yoruba that he/she knows how to speak or understands, some people it's only Igbo. So it should be done in local languages also.

Reference 6 – 0.49% Coverage

nterviewer: What modalities can the risk calculator be administered that is in-person, administered by a nurse or health professional, or self-administered etc, paper/pen, online platform?

Respondent: I believe you should all modalities, that's how you can get a very rich and good erm result.

<Files\\KII Transcripts\\ > - § 4 references coded [1.86% Coverage]

Reference 1 – 0.22% Coverage

Interviewer: Do you think it can be easily used?

Respondent: Yes. So but then how do you intend to do that?

Interviewer: We'll get there.

Respondent: Oh okay.

Reference 2 – 0.52% Coverage

Respondent: They can also use it. Like what I mentioned, that was why I was asking that how do you intend to make them use it, because you might have an adolescent who cannot read it, you might have an adolescent who do not have access to a phone, so in such a situation you tend to want someone who is counselling or someone who is doing the psychotherapy to administer it to him

Reference 3 – 0.37% Coverage

Respondent: Even if you provide it in local languages, a child who cannot read cannot read. That's the simple truth you know. It's just like this inform consent also, it could be oral, you can read it to the verbally, also it can be handwritten or something so it depends.

Reference 4 – 0.75% Coverage

Respondent: We can use all the modality. However we can also do it online. And then if you're doing t online, please let there be a helpline number there, it can even be a toll free or let somebody be…I don't know how they do it but if there's going to be an interface, like anybody who answers the questions online and the person is high risk, let there be a way we can assess such a person so don't just allow the person go like that so that we can assess the person, give a call through to the person and see how such a person can be helped.

<Files\\KII Transcripts\\ > – § 5 references coded [3.17% Coverage]

Reference 1 – 0.32% Coverage

Respondent: Well I think someone should be beside them. Because even if an adolescent commits a crime by way of law, I think a guardian, parent should be there even when he/she is writing statement.

Reference 2 – 0.44% Coverage

Respondent: Should be there asking the questions you understand**.** because sometimes some of them might not really understand the meaning of the questions so someone should be there to really explain what it means so that they won't really go and give the wrong answers.

Reference 3 – 1.02% Coverage

Respondent: The only challenge I foresee is the adolescent trying to comprehend every detail, content of the message. That is why it is good for someone to be around them to…the ones they don't understand, put them through, let them understand what this question is talking about. It'll really assist them. That I think is the only challenges and sometimes again, language barrier. Because some of them might not be able to comprehend English. Well maybe they can translate in broken language or Yoruba. Maybe they can translate them in other major languages, Nigerian languages. Maybe Yoruba, Igbo, Hausa and basically broken.

Reference 4 – 1.05% Coverage

Respondent: Well erm, is gonna affect the adolescent in the sense that you know, for you to know you're in high risk definitely you get scared you understand. and again that's the reason someone should be with him/her just to tell him fine you might be at high risk but you're in the safe hands, these are the things that you are meant to be doing now, you need my help, you need a help of so, so and so, you need to stay away from so so things, you need to do this, you need to do that. So that is why it is important for someone to be beside them just to you know counsel them immediately if they know that they are at high risk for depression.

Reference 5 – 0.35% Coverage

Respondent: I think doing it in paper and biro will be because most of them might really understand or might not be able to have access to phones and computers and all that. So doing it like a questionnaire right?

<Files\\KII Transcripts\\ > - § 3 references coded [3.09% Coverage]

Reference 1 - 0.30% Coverage

Respondent: It should for all…I mean from adolescents upward. For adults, parents, for teachers to use on adolescents.

Reference 2 - 0.69% Coverage

Respondent: Uhm, okay. I feel that this erm…for this calculator to be effective, there should be a need for the people that would administer it should also get a short training on mental health issues on depression particularly. Before they can now start administering it.

Reference 3 - 2.10% Coverage

Respondent: So that is possible. That was why I did say that people who will administer this questionnaires should also be trained too. And then that training should be inclusive of what to say and what not to say. So if an individual have adequate information about a particular illness, you know that person that person can become an expert patient. So that she'll know what to do and what not to do and where to seek for help, whether to seek for immediate help or delay it and still watch out. And then a little bit of talk therapy can also douse the tension, can also reassure the individual that is at high risk to take things gently and all that. Information is powerful so when an individual has enough information to work on, the issue of self-stigma will not be anything to…I mean that would pose a serious concern.

<Files\\KII Transcripts\\ > - § 3 references coded [2.90% Coverage]

Reference 1 - 0.95% Coverage

Interviewer: Do you think it can be easily used?

Respondent: Yeah it can.

Interviewer: Who do you see using this calculator (parents, teachers, health workers?) Where or when would they use it?

Respondent: I think as a health professional, we can use it to assess an adolescent. But for parents to use it, I don't know. You know a professional knows what to do or what to say at a particular time. But the time the parents start asking questions and the child might not want to give the right answers to the parents.

Reference 2 – 0.72% Coverage

Interviewer: Do you foresee any challenges for using this questionnaire? If so how might we address them?

Respondent: The only challenge I see there is, I think you talked about the language and it is between 4 countries. In Nigeria you know we have so many tribes and some might not, English speaking might not be the in thing, but if it comes in another dialect it will make a better result.

Reference 3 – 1.23% Coverage

Interviewer: What modalities can the risk calculator be administered that is in-person, administered by a nurse or health professional, or self-administered etc, paper/pen, online platform?

Respondent: Well, the usually way is through a professional, because in that regard the issue of confidentiality will play. This social media "do it yourself" these days, I don't know how we can achieve it if the err keeping such data can be so secrecy that nobody will have access to it except the patient itself. If that can be achieved fine, but if it cannot be achieved, I think the going through the health professionals will still be a major way of keeping all this information.

<Files\\KII Transcripts\\ > – § 1 reference coded [3.28% Coverage]

Reference 1 – 3.28% Coverage

Interviewer: Another thing, this was designed for an adolescent to use. Do you think other people should be able to use it, for example teachers, parents, health workers?

Respondent: Yes, a parent can use it, a teacher can use it. Even his thing…health workers.

Interviewer: Where do you think health workers can use it?

Respondent: Clinic. Like the Tuesday and Thursday clinic, our health talk. We can use it as a pep talk for our clients and their relative.

Interviewer: What about school, when do you think they should use it?

Respondent: In school they can organize this thing…it can be a seminar for them, maybe a Monday whatever or maybe one afternoon you'll inform the school ahead you want to come and give of the awareness of what this thing is all about. It can be on a Friday afternoon when they are ready to go home, so it'll sink in them and get them through the weekend.

<Files\\KII Transcripts\\ > - § 1 reference coded [1.37% Coverage]

Reference 1 - 1.37% Coverage

R: parents too will, we should also draw one for parents so they wil also know where they are lacking in the upbringing of their children

I: they too can be there, give answers to questions they can identify

R: they can identify that okay I am not doing too well in this are because you asked a question do you eat enough or not eat enough. The parent can tell you I provided food

I: they might not know its not enough

R; and when they ask and the child say I still need they tell you to shut up that what I have. But they need to know the advantage of feeding the chid very well and that will not allow the child to pick up other social vices like stealing or lying

<Files\\KII Transcripts\\ > - § 1 reference coded [0.55% Coverage]

Reference 1 - 0.55% Coverage

R: i don't think. In fact, it’s long due. For me, it’s long due because so many adolescents are depressed, nowhere to share and this is, even if it's computer based or if there is a link to this and it's well set up. I think it's very good

<Files\\KII Transcripts\\ > - § 2 references coded [1.54% Coverage]

Reference 1 - 0.48% Coverage

Respondent: should increase especially in terms of, because a lot of people are actually going through it without them actually knowing. I think it should not only be assessable only to parents. I think the risk calculator should be assessable to everybody.

Reference 2 - 1.06% Coverage

so I believe this risk calculator has to be assessable to everybody. Once you notice that this is what you going through, you yourself might actually believe okay let me actually seek help. I think individuals should be educated on who psychiatrist is, who a psychologist is because the moment they hear psychiatrist they’ll be “hmm hmm not in our family, we’ve never visited this” they are always scared. I think hearing a psychologist is even more acceptable or a guidance counsellor, a social worker is more acceptable, but once they just name that, okay you’ve lost it.

<Files\\KII Transcripts\\ > - § 3 references coded [2.01% Coverage]

Reference 1 - 0.18% Coverage

Interviewer: do you think it can be easily used?

Respondent: of course.

Reference 2 - 0.73% Coverage

Interviewer: who do you see using this calculator (parents, teachers, health workers?) Where or when would they use it?

Respondent: it should cut across.

Interviewer: so you think parents, teachers, health workers should be able to use it on adolescents?

Respondent: Of course they should.

Reference 3 - 1.11% Coverage

Interviewer: do you foresee any challenges for using this questionnaire? If so how might we address them?

Respondent: like for instance what do you do about tose that are not educated?

Interviewer: so think we should make this in what other form apparently from this one meant for literate people?

Respondent: yes, you know that for those that are illiterate, isn't it possible to interview? Like to conduct oral interview and get their response.

<Files\\KII Transcripts\\ > - § 8 references coded [5.94% Coverage]

Reference 1 - 0.58% Coverage

Interviewer: okay. Do you think this calculator can be easily used by the adolescent?

Respondent: sure it can be used by the adolescent. Is just the matter of just answering the question and letting it calculate it for you. So it is very easy.

Reference 2 - 0.64% Coverage

Interviewer: so apart from the adolescent using this calculator, who else do you see using it? Should the parents, teachers or health workers use, administer this questions for an adolescent or should it only be used by an adolescent?

Respondent: I think all sectors...

Reference 3 - 1.44% Coverage

Interviewer: so do you foresee any challenges with using this questionnaire? Are there any problems? Do you think there are issues with using this questionnaire?

Respondent: eh, the main problem there to me is literacy.

Interviewer: okay. So what do you think we should do?

Respondent: an illiterate person may not be able to use it him or herself, except the something is being administered on him or her by someone. So what an improvement to do would be a kind of, of eh..oral stuff. Or voice, if he can have this thing bring done in a voice system that instead of reading them, something is asking...

Reference 4 - 0.60% Coverage

Interviewer: say it out.

Respondent: saying it out. What is this, then the person can actually answer.

Interviewer: or maybe we can also make it in vernacular.

Respondent: vernacular or other languages.

Interviewer: other languages.

Respondent yeah.

Reference 5 - 0.82% Coverage

Interviewer: uhm, so what modalities can this risk calculator be administered? I mean should it in person, should be administered by a nurse or a health professional or should it be self-administered?

Respondent: I think the best is from a medical specialist. Medical specialist or a counselor. A community health worker or a counselor like that.

Reference 6 - 0.73% Coverage

Respondent: it's better than instead of the person using it because, if the person is administering for him or herself, it may get to a particular stage or level the person may stop as a result of some questions which the person may not feel happy about. So it is better, or it is best to administer it on.

Reference 7 - 0.92% Coverage

Interviewer: okay. So do you think we should leave it based on paper and pen like me actually printing out a questionnaire for you to fill or should we do it on an online platform or any other platform?

Respondent: an online platform will go a long way than paper. You know it is very fast, fast than paper. In space of one minute you can get to, you get over six. So online platform...

Reference 8 - 0.20% Coverage

Interviewer: so you think online platform is better.

Respondent: I think it's better.

<Files\\KII Transcripts\\ > - § 5 references coded [2.80% Coverage]

Reference 1 - 0.49% Coverage

One thing about questionnaires is if there's no feedback there won't be result. That means the adolescent cannot use it for him/herself. So there should be an adult, a teacher, a counsellor or a health worker, even health personnel, those ones...they should be the one to use it for a depressed person. And the most...why I'm happy about is you can use it even for an adult. It can be used for an adult.

Reference 2 - 0.23% Coverage

Most challenges that we face with questionnaire is people might not say the truth. But when you're administering something like this, it has to be a confidential. It has to be very confidential.

Reference 3 - 0.47% Coverage

Err that's why I said there should be follow up. Between you and I, I already know that I have high risk of being depressed in the future and there's an adult that's always there...Bose o come o, you know between you and I you have filled this and this is the result of the questionnaire that you have filled...there should be feedback. You have to tell the person the result especially...

Reference 4 - 0.27% Coverage

Interviewer: So how would that make the person feel, knowing that he has a high risk for depression?

Respondent: He shouldn't feel bad, after all, he/she is not depressed. And the essence of knowing is for you to prevent it.

Reference 5 - 1.34% Coverage

It should not be a self-administered questionnaire that means the adolescent cannot administer by themselves. So it should be health worker, or teacher, counsellor or anybody that has been trained to identify, should be administered by those people. The last question which is should it be online, paper...okay because I wouldn't say it should be pen and paper whatever. Because at times documents to can be missing placed. And such an information like this is supposed to be kept. There should never be a time that if you have to refer to that person's file, that you won't get to see the file. Maybe you can do it online. Just create a platform under this organization or whatever for each patient that you have...sorry under the organization just create a page for each patients online. Then when the person is around, the person can just go, you would ha e created the erm the username and password for that person. He/she can just go to the room, there is a computer, it's between himself/herself. So the only person that can access that information is the person that is administering the questionnaire.

<Files\\KII Transcripts\\ > - § 7 references coded [2.97% Coverage]

Reference 1 - 0.09% Coverage

Interviewer: Do you think it can be easily used? Respondent: Yes. The English are okay.

Reference 2 - 0.28% Coverage

Who do you see using this calculator (parents, teachers, health workers?) Where or when would they use it?

Respondent: Yes. They should to administer it to them. From there you'll draw opinion and know what they are going through. The question is well prepared for them.

Reference 3 - 0.13% Coverage

Interviewer: Do you foresee any challenges for using this questionnaire? If so how might we address them?

Respondent: No.

Reference 4 - 0.29% Coverage

What additional information would you like to know for someone who has high risk or low risk?

Respondent: From my own opinion it's okay. When questionnaires are too cumbersome you will not be to answer them. The questions are straight forward and not too cumbersome. It's okay.

Reference 5 - 0.36% Coverage

It'll…if by the time they fill it and they now discover, it now depends on the person administering it to follow up. It's going to affect them. I'd say yes and I'd say no. Yes they'll know where they belong to in the society. No because for the ones that are counselled on time it'll bring down that risk. So the questionnaire will it carry their name?

Reference 6 - 1.48% Coverage

Yes. That's why the admin should not be there. When the questionnaire is administered, and collected back so you now do your survey, the risk factor you understand, those that are on high risk. So what I suggest is a seminar should be organized to address everybody at the same time, so that one does not feel that "they know me that is why they are calling the seminar" do you understand. I've gone to a church program, I'm a children teacher in the church. So we had a provincial program for teens and err children. So in the seminar, a group of …all these people that take care of children and teens, they were the people that organized that program, the church invited them. Just like what you said they administered questionnaires to the teenagers aside their parents, so when the children they filled the questionnaire, they asked their questions and everything, they now brought it, they counselled the children they left. They now brought their questions to the parents, in fact that day it was very hot. The moderators started reading out the questions to the parents, what the children were writing about their parents, about their uncles, about their daddies, even some pastors in the church and the whole place got "a wire" in fact. So the parents were counselled, they did not

mentioned any child. They now said the parents should go back home and talk to their children, know who their children is.

Reference 7 – 0.35% Coverage

I think that online is even good. You know adolescents they like everything online. So if possible too you can go to schools, you can go to hospitals where they have children sections administer questionnaires, the children, they will say their mind. One thing about children is that they don't pretend they say it the way it is in their mind.

<Files\\KII Transcripts\\ > - § 7 references coded [2.66% Coverage]

Reference 1 – 0.40% Coverage

do you think it can be easily used?

Respondent: yes, it's easily understood.

Interviewer: by every adolescent?

Respondent: yes every adolescent. Yes.

Reference 2 – 0.30% Coverage

who do you see using this calculator (parents, teachers, health workers?)

Respondent: yes they should be able to.

Reference 3 – 0.30% Coverage

Where or when would they use it?

Respondent: in school, in the house, in the community, in the church, in mosque.

Reference 4 – 0.72% Coverage

do you foresee any challenges for using this questionnaire? If so how might we address them?

Respondent: not really, except for the fact that if a child refuses to even answer the question in the first instance.

Interviewer: so is that the only challenge?

Respondent: yes.

Reference 5 - 0.57% Coverage

okay so basically uhm, such a child should be you know, educated on the benefits of such a calculator. When the child knows the benefit, definitely the child will answer the questions. Educate the child in the impact.

Reference 6 - 0.15% Coverage

I think in every way in other for it to get to everybody.

Reference 7 - 0.21% Coverage

online platform, paper and pen questionnaire, interview and so on and so forth.

<Files\\KII Transcripts\\ > - § 6 references coded [3.41% Coverage]

Reference 1 - 0.20% Coverage

Interviewer: Do you think it can be easily used?

Respondent: Yes.

Reference 2 - 0.15% Coverage

Respondent: Everybody can use this questionnaire.

Reference 3 – 0.99% Coverage

Respondent: As a teacher in my school, we can give them the students in the class to actually answer them and we take them back from there. Like the health workers too can go around schools, they can go to churches as well. You can even go to this err Shoprite and all that, to malls and give them to students and collect it back.

Reference 4 – 0.73% Coverage

Respondent: You know I am teacher I will always look at my school system. To me there is no challenge. I have some students that I can actually give this questionnaire to and they will be very happy to answer it. But I don't know about outside.

Reference 5 – 0.87% Coverage

Respondent: Just like I told you, if a corper (National Youth Service Corp Members) walks up to them, somebody dressed like they ha! and all that relate to them and all that I don't think there will be challenges. Because they would want to to and you know answer them and do what they want.

Reference 6 – 0.47% Coverage

Respondent: Both teachers, the health workers. Pen and paper is even better you know when you give them paper with pencil they answer the questions better.

<Files\\KII Transcripts\\ > - § 3 references coded [2.82% Coverage]

Reference 1 – 0.16% Coverage

yes, yes, yes, yes, can use it. It's very good and it's a welcoming question and idea

Reference 2 – 1.67% Coverage

In this kind of setting, if we use this risk calculator for an adolescent and maybe he gets high risk, do you think there's a possibility that there might be stigma? Like if a teacher uses it and there's high risk the, teacher might start treating the adolescent differently because the adolescents is at high risk. Do you think that is a possibility?

R: you know this question is not meant for teachers

I: you think it is better that it's a counsellor, someone that is trained

R: yes… it should be a…It's a professional question, meant for, to be handled by professional himself like a counsellor, you know your work, you know the ethics of your work, you know the dos and don'ts. It's your work so when you administer such a question on your wards, on your client, you know the answer, the result of it, start and end with you but a teacher can use it against

Reference 3 – 0.99% Coverage

do you think that we should have paper based ones instead of just the computer based?

R: yes yes because majority of our child of adolescent uses

I: paper and biro

R: that is when you get desired results and some of them most especially public school, don't even know how to touch computer. To even answer it might be a little bit difficult for them. So if it's in paper and pen, they can write and they will be able to tick either yes or no and from here the professional will analyse the result and work on it.

<Files\\KII Transcripts\\ > – § 7 references coded [5.36% Coverage]

Reference 1 – 0.90% Coverage

Interviewer: Do you think it can be easily used?

Respondent: It can. Any literate person it will be very...

Interviewer: So what about illiterates?

Respondent: Which means you need to interpret; you need to explain what it means to...

Reference 2 - 0.58% Coverage

I don't think the teacher can use it because a teacher...we are talking about depression in adolescents not in adults and teachers they have passed...

Reference 3 - 0.10% Coverage

Yes, a teacher can use it.

Reference 4 - 0.41% Coverage

So teachers, erm parents, health workers should be able to administer it on the adolescent?

Respondent: Yes.

Reference 5 - 0.86% Coverage

In the church they can, all this youth forums when they are having...even harvest, thanks giving, those ones that are youths, harvest. Any social worker can come there and distribute the questionnaires to the adolescents alone.

Reference 6 - 1.02% Coverage

Yes. Honesty.

Interviewer: So how might we address it?

Respondent: It still making the person to know the reason why. That's creating that awareness. If the awareness is created and it's confidential, nobody you know, the person will be free to bear his or her mind

Reference 7 – 1.48% Coverage

Interviewer: What modalities can the risk calculator be administered, that is in-person, administered by a nurse or health professional, or self-administered etc., paper/pen, online platform?

Respondent: To me it can't be done by only one person because it will not go round. You'll not have enough data, many people will not be sampled. I think you can use as many modalities as possible.

<Files\\KII Transcripts\\ > – § 3 references coded [1.31% Coverage]

Reference 1 – 0.21% Coverage

Respondent: yes the teachers can have this recommended to the children. And it can also be used by them. The adolescents themselves.

Reference 2 – 0.78% Coverage

Respondent: every means of communication should be adopted. You have people that don't have time to read papers. And we ha EA people that don't come online, like those people on the street now. You know, ask them to come and be reading paper they'll just look at you like what is this one. You can use word of mouth to tell them. You know there are some that cannot read. Even this English that you put here is jargons to them, so they need Yoruba. Some need hausa, some need igbo.

Reference 3 – 0.31% Coverage

Respondent: of course. Of course, if it can be done. It will be helpful. Because we ahve different tribes in Nigeria that doesn't speak English. Some people their own dialect and all of that.

.

<Files\\KII Transcripts\\ > - § 3 references coded [3.30% Coverage]

Reference 1 - 1.19% Coverage

Respondent: Everyone in involved. Because some of the err...the parents that have an adolescent at home, the teachers also they have, and the health care...they are also parents and caregivers so they should have one or two of those adolescents at home. She can be able to use it to see if their adolescent is in depression or their community.

Reference 2 - 1.65% Coverage

Respondent: I think using of the whole modalities.

Interviewer: Why should we use all the whole modalities?

Respondent: Because in every err this thing... the hospital will have adolescent there that will also go for maybe checkup and so forth. Maybe parents they have adolescents at home, in schools there are adolescents so you'll be able to get a larger information, than being restricted to only one modality. To give you let me say a wider err, how will I put it sef...

Reference 3 - 0.45% Coverage

Respondent: If it is paper a d biro it can get leaked, destroyed. I think it should be online ..even online the risk is there too.

<Files\\KII Transcripts\\ > - § 2 references coded [1.67% Coverage]

Reference 1 - 1.07% Coverage

I: Who do you think, like parents, teachers, health workers, asides adolescents themselves since it was designed for adolescents to use themselves, do you think it can be used easily by parents, teachers and health workers?

R: Ah, three of them na. Once you can read or write.

Reference 2 – 0.60% Coverage

R: Ehn, disadvantages is more on hardcopy. You know it's not all the questionnaires you can get nowadays but if it is online something and you have a target.

<Files\\KII Transcripts\\ > - § 1 reference coded [1.28% Coverage]

Reference 1 – 1.28% Coverage

Interviewer: Another thing we wanted you to know is, this prototype was developed for adolescent to use. Do you think other people should be able to use it, for example maybe teachers, parents, counsellors for example?

Respondent: I'm not sure it would be easy, I think it's something that should be self-administered.

<Files\\KII Transcripts\\ > - § 1 reference coded [0.74% Coverage]

Reference 1 – 0.74% Coverage

Interviewer: You as a paediatrician, who else do you think should have access to using a tool like this?

Respondent: A nurse, a teacher, a counsellor in church or wherever they go to, yes. So such people.

<Files\\KII Transcripts\\ > - § 1 reference coded [1.64% Coverage]

Reference 1 – 1.64% Coverage

Interviewer: so who do you see using this calculator apart from the adolescents? Should a parent be allowed to use this on their child? Should teachers be allowed to use this for students? Or should it only be the adolescent answering this questions by themselves?

Respondent: erm, everybody should be able to access this calculator, so that you have...'cause in creating awareness, I should know what I'd be looking out for. So if I have something like this at the back of my mind, consciously or unconsciously I could just work with it and I be able able to like, this child needs closer monitoring and all that. So I think everybody can use it.

.

<Files\\KII Transcripts\\ > - § 2 references coded [1.42% Coverage]

Reference 1 - 1.01% Coverage

Interviewer: Do you foresee any challenges for using this questionnaire? If so how might we address them?

Respondent: Access to internet.

Interviewer: What if we had it available in paper copy? You think that's a good thing to do?

Respondent: Yes, you can make it both.

Reference 2 - 0.41% Coverage

Respondent: Well, all children maybe they'll will take it class by class. All children should go through this.

<Files\\KII Transcripts\\ > - § 2 references coded [0.48% Coverage]

Reference 1 - 0.10% Coverage

Respondent: Okay. This one is in Yoruba.

Reference 2 – 0.38% Coverage

Respondent: Yes, if you train them. I think anybody seen as a respective figure can actually you know use it and you know apply it to the adolescent.

<Files\\KII Transcripts\\ > - § 2 references coded [2.28% Coverage]

Reference 1 – 1.23% Coverage

Respondent: Yes, it's something where you can be used to assess, have they started noticing this particular…it might even be the teacher that uses this and just want to be sure before he/she reports to the school counsellor or to the parents. Like okay I actually did an assessment of this child and I think he/she might need help. So it's something teachers and parents can also use.

Reference 2 – 1.05% Coverage

Respondent: Well, it depends, like what I saw, it's like I don't have the so many versions. Like the language and also, it depends on educational level. Access to internet. It's going to be an application that's going to be offline where you can just download and doesn't have to be something that has to do with use of data…

<Files\\KII Transcripts\\ > - § 1 reference coded [1.00% Coverage]

Reference 1 – 1.00% Coverage

Interviewer: Another thing, this was designed for an adolescent to use. Do you think other people should be able to use it, for example teachers, parents, health workers?

Respondent: So interviewer administered, definitely erm, if you want to keep up appearances you'd not sit with your parents or your teacher that you do drugs. So I think it's better self-administered or administered by a mental health expert.

<Files\\KII Transcripts\\ > - § 1 reference coded [3.92% Coverage]

Reference 1 – 3.92% Coverage

Interviewer: Another thing, this was designed for an adolescent to use. Do you think other people should be able to use it, for example teachers, parents, health workers?

Respondent: Yes.

Interviewer: Okay, where do you think this would fit in the scheme of things, for a teacher, or for a health care professional, or for a parent?

Respondent: I think all of the above.

Interviewer: where do you think they can be used?

Respondent: where?

Interviewer: Yes, that's the health care professional for example, how does it fit into your consultation? A teacher at what point do you think that the teacher can use?

Respondent: you know; you must have seen one or two things. For a health care professional, at times you might see cases and you're not sure whether this patient is depressed or not or you're having doubt, so you can use the questionnaire sort of and maybe that will be more authentic in assessing the patient. And also, the risk calculator can even be routinely given to students in schools irrespective of their child's behaviour at the beginning and end of the term so that they can also be able to catch them early.

<Files\\KII Transcripts\\ > – § 4 references coded [6.18% Coverage]

Reference 1 – 0.85% Coverage

Interviewer: Do you think it can be easily used? Are the questions acceptable and appropriate?

Respondent: Yes, I think the questions are acceptable and appropriate. They were easy to follow the language was actually…I think brought to their level because it's important, they are the one using the questionnaire so it's important we speak their language.

Reference 2 – 1.99% Coverage

Interviewer: Do you foresee any challenges for using this questionnaire? If so how might we address them?

Respondent: Erm, I think some of the challenges might just be that when we're giving it to the adolescent, we need to make sure that erm they are in…maybe they are alone, their authority figures are not around to supervise them completing it. Because sometimes the presence of the authority figures can influence the responses these children can give. Also make sure their peers are not around them because some of them might want to say "let me see what to wrote" "let me see what you ticked" you know, and you might be able to get the true picture of things. So it's important that maybe when you want to give them, you put them in separate rooms where they're alone and can easily give you erm the responses as they truly feel.

Reference 3 – 2.38% Coverage

Interviewer: What modalities can the risk calculator be administered that is in-person, administered by a nurse or health professional, or self-administered etc., paper/pen, online platform?

Respondent: I think what we can do is, apart from maybe giving them in their schools, in religious homes, we can also put this risk assessor maybe make into an app. You know children really love gaming, their almost always with their phones maybe you can make into an app, or we can also put it on social media like maybe Instagram, Facebook, or have a WhatsApp group because those children are always with their gadgets. You can make into papers for maybe when you're taking it to their schools or to religious homes or something. You can, but fear about paper apart from thinking about the environment is that the children might just loose it, they might not be able to remember the helpline or where to seek help. So I really actually prefer for it to be an app or to be put on the social media it will help.

Reference 4 – 0.96% Coverage

Interviewer: Alright, is there any other thing you'll like to add?

Respondent: Erm yes, I noticed that in the questionnaire you just wrote helpline, I think the helpline should be toll-free, because adolescents apart from trying to call their friends they really don't want to talk to anybody and be paying for the call. I think you should make it toll-free so that the helpline…they can easily access it.

<Files\\KII Transcripts\\ > - § 4 references coded [4.36% Coverage]

Reference 1 - 0.28% Coverage

Respondent: I think it's…adolescent will get lost if it is longer than this. So I think this is just long enough.

Reference 2 - 0.21% Coverage

Respondent: As it is it's something that any adolescent with basic literacy can follow.

Reference 3 - 3.06% Coverage

Respondent: Nobody can answer these questions for an adolescent. Teachers and parents can only benefit from this is if they see these questions, they might be able to understand a bit more what are the risk for depression. But nobody can answer this for the adolescent because it's a personal experience for the adolescent. I think in instances where it is…I don't see parents doing this but I'm thinking more of anonymous people or third parties that are working with adolescents. Teachers are working with adolescents, not every random teacher but teachers that are designated to play the role of working with an adolescent. And also things like health workers within the community and the rest of them, they should be able to administer it to an adolescent. But then it's more useful when there already an indication, not just "you come and answer" the fact that I answered a lot of them doesn't necessarily mean that I am a depression risk. It's more useful when already there is concern, they now want to say okay let me use this to even check whether this patient is at risk for depression. Unlike when the adolescent is answering it directly, any adolescent whether you have issues or you don't have issues can go on and check this.

Reference 4 - 0.81% Coverage

Respondent: Well it can be paper or pen, it can be online, but then it's better that it is self-administered. It is only in very unusual circumstances, for example an adolescent is not able to read, does not have access to internet, and all of that. But the more individualized this is, the more likely it's going to be useful.

<Files\\KII Transcripts\\ > - § 5 references coded [3.95% Coverage]

Reference 1 - 0.21% Coverage

Respondent: eh...they are acceptable and appropriate for people who can read.

Reference 2 - 0.11% Coverage

Respondent: what about an audio version?

Reference 3 - 0.73% Coverage

Respondent: it makes the work easier anybody can use it. So you don't need a trained specialist to be able to administer it. And I think that's the best part. The part, that you can have people who have minimum training administer the tool to be able to calculate risk.

Reference 4 - 1.30% Coverage

Respondent: online platforms will allow them to do it by themselves in their own privacy. They may decide do it at home or in one corner somewhere. Paper based may be a problem because, if they sit down together to answer the question and they say oh what did you write and you write the same as the other person would not tell you the truth about what's going on at home because they don't want anybody else to know. I think any method that would promote privacy would be better.

Reference 5 - 1.60% Coverage

Respondent: self-administered but through different platforms. So either you want to go and see your school counselor you understand and maybe be uses the opportunity to be able to administer that as part of the routine things that he is going to do while counseling or provide access to it. Since everybody has internet anyway, maybe put their links in different parts in their

schools where you can just take it by yourself. But the teachers for example will encourage them to participate and maybe give them some incentive if they participate, they'd just not have access to their result.

<Files\\KII Transcripts\\ > - § 2 references coded [4.82% Coverage]

Reference 1 - 2.99% Coverage

I: you know we said this was designed for the adolescents but do you think that other people can use? Is it a tool other people can use?

R: as in a tool generally or this tool

I: this risk calculator.

R: they may have to modify because adults are different from children.

I: what I mean is like a parent using to assess their child, like a teacher using it to assess their students. It's still for adolescents but

R: but who would answer the question, they would answer it for them?

I: the teacher can administer it too

R: so it won be self-administered anymore

I: that I what I am asking, what do you think about that? It's originally designed to be self-administered but what do you think about it?

R: Interview based style. Maybe it won be like a formal interview, the person should somehow have the questions in his head, so maybe let's go out to eat and then you just ask I mean if you really had something personal would you be able to share with me?

I: that's the parent

R: that's the parent, if I take your phone away from you how, you know .you won't make it like a formal interview. You take the person out, relax, some fun place that a child would be able to tell you the truth. Because those people they can lie [laugh]. It's like they almost know the answer you are expecting and they will give it to you

Reference 2 – 1.83% Coverage

I: but even things can be made up on self-administered

R: self-administered you may get a better response especially if you have a very good introduction, no name no kind of thing and let them just know that because the stuff I did on reproductive health was quite, so making them know that I am not using your name, just doing this so we can help other young people. Some of their answers were amazing. So it depends on how…

I: so you think self-administered is better for us to be able to get accurate information

R: for young people, yes as I said you may also have issues with adolescents who are not well educated, they cannot read

I: so the risk calculator might not be good for people who are not educated?

R: yes because if they can't interpret these things very well, they will just mark anything for you

<Files\\KII Transcripts\\ > - § 3 references coded [3.49% Coverage]

Reference 1 - 1.55% Coverage

Interviewer: Do you foresee any challenges for using this questionnaire? If so how might we address them?

Respondent: The legal aspect. Especially for an adolescence, one that is below 18 years old, the consent of the parents. That's one thing that you do see. There are two ways that they can...they can work with the schools so that it is a part of the package that the parents sign the form. I know that in my daughter's school, there's health day they do screening for eye, the dentist comes and so on and so forth. So I basically approved them, they give me forms to approve such. So you can work with the schools so that you get consent of the parents.

Reference 2 - 1.01% Coverage

Interviewer: What modalities can the risk calculator be administered, that is in-person, administered by a nurse or health professional, or self-administered etc. paper/pen, online platform?

Respondent: Online platform is important because erm, we are looking at adolescents only, but definitely we see other young adults, that's why I use the word young adults. Young adults fall outside 19bhears, from 20, 21 to about 24 abi?

Reference 3 - 0.93% Coverage

Interviewer: Yes 24.

Respondent: If they lay hands on the questionnaire they can fill it also, or we can use it as a basis to start care for them. So definitely yes, it should be online.

Interviewer: Should we also still leave it as pen and paper or only online?

Respondent: All forms. All modalities please, so that self-administered should be there, then err the teachers should be able to....

<Files\\KII Transcripts\\ > - § 8 references coded [7.13% Coverage]

Reference 1 - 0.06% Coverage

R: That line, that toll line

Reference 2 - 1.36% Coverage

R: Yes I think that will make it more information gathering. Yes I am looking at the language, but like you said, you said it can be err translated to other languages and all that, if not errr I think the the questions are simplified enough for anybody to understand but if you look at the literacy level in the country. Yes, there's still need, still need for you to still simplify it more. Then err can if possible they translate it to other language that may be easier for anybody to answer. Apart from that, the tollgate, the toll line you talk about is it three digit or it will be normal phone gsm number

Reference 3 - 0.23% Coverage

R: Okay because by the time you, let it be like short toll free that will be easier for anybody to pick

Reference 4 - 1.28% Coverage

R: And apart from that, you should have like an information on err err signs, err for err err hearing impaired people, eye impaired and all that so there should be that too

I: We should have versions for people who have physical challenges

R: Yes yes because I must tell you they are the ones that are more into depression. Their situation, because of stigma and all that put them in a lot of err bad situation that lead to all these mental health and all that so if possible translate it to err sign language or physically impaired friendly errr so it go a long way yeah

Reference 5 - 0.91% Coverage

R: Definitely that's the in thing, how many people have access to computer, even if they have access to it, do they have access to internet and all that? but most important one like the social media we talk about. Most of us have one way or the other to buy data on our phone and all that if there's any or if possible without data, having data you can assess it.

I: download it

R: yes that will be fine

Reference 6 - 1.58% Coverage

R: except if it's translate to other languages and it has to be like a supportive err, that will, that will help, that's the way I see it, it will have a have a more or less a guide. Perhaps somebody is there to guide them to do that. For instance now, if you bring, usually have research here conducted on these ones that we have here and most of the time I used to tell them they write all sorts of big grammar and all that coming to them

I: [laughter]

R: and I can assure you err like 40% of them can't read and write because they have been on the streets for long. They have not been to the four walls of the school and all that so that's why you see most of them starting 16 14 15 in nursery class

Reference 7 - 0.99% Coverage

R: nursery class just for them to be able to know how to read and write. Just imagine you bring err such err they will not be able to read, so we assist most of the time, the officers assist in administering these err questionnaire. If it's possible that somebody can be there to guide to, assist in administering it, it will go a long way, if not they just put anything there and it may not give you the true picture of what exactly you need.

Reference 8 - 0.73% Coverage

R: yes so I think if it’s possible for somebody to guide in using them and all that, it’s possible here but I don’t know how possible outside but it should be possible in the school too and since we are talking about starting from the school err err it should be working if err those that are assigned to it are ready to do that.

<Files\\Healthcare Workers\\Policy Makers\\Transcription > - § 5 references coded [19.14% Coverage]

Reference 1 - 5.56% Coverage

It is. But, what I think is interesting, which kind of jars with me with your questionnaire is that when we take the names of the students who have scored over 20 back to school, about 50% of them, the school say, yep we would definitely see that, that would be the child that we would identify with, and about 50% are the hidden children. And they are often your high achievers. So, I'm not saying that ACEs don't occur in those students, but they're probably less likely to see it. And my concern is, from looking at this questionnaire, is it still feels a little bit like we're saying, if you come from a deprived area, from a dysfunctional family, you're going to get depression. And actually, that's not necessarily the outcome. I would be interested to correlate this to data in adult mental health, so from a demographic of adult mental health, if we were to look at those people in their childhood, what would they have scored in this. Because that for me would be the bit, because I just feel, particularly, we've got a huge shift in our culture that is, we've got a very different cohort of young people and parents and different styles of parents coming through than what we had even five years ago. So, obviously things like social media are having a really high impact on both our parents and our children. But this is our first generation of that. And what young people are saying to us, you know, a lot of their negative thoughts that they're holding onto or focussing on, come from their immediate negative interaction with what's going on in social media. So, not looking good enough, their friends having more fun, not being part of the gang, you know, that type of thing. Which we know, you know, you can focus on but actually there's very little evidence that that's the truth. But that's where young people hang their hat. And it just concerns me that we, it just feels like we're taking historic thought processes and we're not bringing them up to date with what our, where we presently are.

Reference 2 - 2.31% Coverage

Yeah. So I do think that the indicators that you're asking, but actually, the other thing for me is that often, if you look at the kind of family experiences and things that you've put in this questionnaire, they will be the things that will impact on the synapses on the brain, won't they? They're all the things that will impact on their emotional intelligence and their resilience, potentially. And I have a concept, I would say it's my concept, of if you, if you live in a deprived socially inept household, actually, does that child have more resilience than a child that is being brought up in a very middle class, giving, resource rich household. That actually, that child will have, potentially will have, more emotional intelligence but less resilience. And will that impact on the demographic of our future depressive cohort?

Reference 3 - 1.05% Coverage

So, I get where they've come from, but actually, ACEs as a model is quite old now in relation to—I think it's a great thing, don't get me wrong, I think ACEs is really brilliant and I can really see how ACEs makes a difference to outcomes for children, I do get that—but I think there's a whole other cohort that we're not grasping. Which is our middle class affluent families.

Reference 4 - 1.21% Coverage

But that’s why I think you’d need to look at the questions that you’re asking because they are based on the ACEs and quite a lot of the children that will tick these things, not all, but a bigger cohort will be your dysfunctional families, your disruptive children, your disengaged children, and actually, my concern is that you're missing a whole cohort of silent achievers who actually are not coping with their emotional wellbeing.

Reference 5 - 9.02% Coverage

Yeah. They’re either the middle of the road, you know, the kids that are just achieving, they’re doing ok, or they’re the high achievers. And I think this is something that the ACEs model won't capture. It’s where you’ve got high achieving families, families that the parents are doing very, very well, but feel very stressed about the risk of their children not doing well. So if you take it right the way back, I read a piece of research this week and interestingly, had a conversation with somebody that reinforced it yesterday with a parent around children entering nursey with minimal self-care. Not being able to dress. And the assumption was that that was in deprived areas. When actually, it isn’t, it’s in affluent areas. And it’s because you’ve working parents, limited morning time, needing to get the children out of the door to nursery care, not having the space or the calmness to teach dressing, washing, doing your shoe laces. So, it’s easier to do it for those children and not then giving them the space. We’ve got a limited space of time, I need to get out of the house, I need to get them to childminder. And they just won't get dressed. And she said, that’s my trigger. And I said, how is it making you feel? And she said, I feel really stressed and really agitated. And I said, but what’s your thought process behind that? And the thought was all about herself and needing to be somewhere. But the expectation of the parent, who is high achieving themselves, wanting their children to be high achievers, but actually just not thinking about, well actually, you know, let’s think of that differently, Monday to Friday I'm going to dress them for now, I'm going to be less stressed, they’re going to be less stressed, but at the weekend, we’re going to teach them some self-care skills. But actually, the pressure that’s putting on those children, and we’ve got children who are in nursery from 3 months old, you know? Constantly having that pressure until they’re 21, coming out of university. That’s huge for those children, it’s huge. And if we look at where our biggest suicide is, it’s in young men, 18 to 25, often achieving young men. So why are we looking at ACEs to identify who’s at risk of depression?

<Files\\Healthcare Workers\\Policy Makers\\Transcription > - § 3 references coded [6.18% Coverage]

Reference 1 - 2.76% Coverage

and, you know, and, sort of, understanding at the individual level what people then sort of take from potentially having that label as being someone at high-risk of depression, or potentially getting a label of currently being depressed. Like, is that something that they then think they’ve got to carry around with them for the rest of their life? And, you know, I don’t think we necessarily have the answers to these things at the moment, and it’s not necessarily a conversation that we’ve had more widely. So, I think it’s mainly because of them being particularly young. But, also, in terms of where society is currently at in terms of the language and the conversation that we have around mental health problems. And there was a real resistance to the idea that you can cure mental health problems, and that it’s something

innate in you, and if you're identified as having one of these problems, that is a part of you for the rest of your life, and, erm. Which may be valid, it may not be valid, it's probably different for different people. Erm, and different people probably take different things from that, and take different sort of levels of importance from that as well

Reference 2 - 1.70% Coverage

Erm, I guess also, you know, these are quite broad categories, right? You've got your high-risk or your low-risk, erm, given some of the factors that we know to be very highly correlated at least with these high or low risks of depression, and they're only some of the factors as well, so it's sort of a lose indication, erm, which I guess could be problematic. You might, you know, depending on what the sensitivities are, and how the questions sort of get scored, potentially, people that maybe are quite resilient for other reasons are being sort of potentially marked as being at high-risk of depression, and ethically what does that mean. Erm, in terms of actually using the tool, as far as I can see, I think that it's quite straightforward. Erm, but, again, I think it's only looking at a certain number of measures in the context of what we know to be important in risk of developing depression.

Reference 3 - 1.72% Coverage

And, I think, something that occurred to me right at the beginning actually, was that potentially, are the questions quite, do they need to be quite context specific? And, it's something we see in terms of, again, there's this real need and desire and want to scale up these interventions, that have been seen to be really successful in one particular location, but actually, I think in a lot of cases you really need to understand the contextual specifics of the location in which you want to roll out that intervention. So, that might be something that's seen in terms of the sorts of questions that are asked, or, how they're asked, or-. Also, another thing that we see is the sorts of language that people use to communicate these sorts of, erm, conditions, they can vary wildly, erm, across different communities, different cultures, different countries, so that's something, maybe, to think about, as well.

<Files\\Healthcare Workers\\Policy Makers\\Transcription > - § 1 reference coded [1.55% Coverage]

Reference 1 - 1.55% Coverage

You know, there was a whole of stuff about having a, I guess a mature conversation about mental health, erm, in young people, and I think they're having that conversation. I'm not sure it's mature.

<Files\\Healthcare Workers\\ > - § 4 references coded [1.45% Coverage]

Reference 1 - 0.29% Coverage

With clinicians using those, it's just…interpreting the result in a certain way, that's not helpful. So, it's just always, as with any questionnaire, like not taking that as the diagnosis, but just as an idea about what this young person might be at risk of.

Reference 2 - 0.59% Coverage

Well, just the usual like mis-missingmisdiagnosing something. I think with lots of young people that, well, lots of young people that are anxious particularly, that we see, and we end up also thinking that they may have undiagnosed autism. And I know that negative thinking is quite common, like depressive type thinking, is quite common in children with autism as well, so there might be a risk there of it saying that they're very depressed, but whether it can pick up on other things that might be going on as well.

Reference 3 - 0.29% Coverage

Risk of depression. If family members have—if there's a family history of depression—and what that means for the family. Do the children know that their family members might be—have depression—or, what's the...like what's the family story about that?

Reference 4 - 0.28% Coverage

I'm trying to think about the family. I don't know, it's family dynamics isn't it? And what that, ugh, what that means. I mean, because often there is a history—a family history of depression—so, that would be—which could be positive or negative.

<Files\\Healthcare Workers\\ > - § 2 references coded [0.41% Coverage]

Reference 1 - 0.18% Coverage

You know, because we also find that a lot of parents have mental health difficulties. Maybe they've had negative experiences of our mental
health services, or maybe just sort of went under the radar

Reference 2 - 0.23% Coverage

I think barriers are; if...if parents have got their own mental health issues and they haven't had the treatment, or say they've gone under the radar, or if they've had a bad experience of it, then they'd be thinking "oh well I went there, my kid's not going there".

<Files\\Healthcare Workers\\ > - § 3 references coded [2.47% Coverage]

Reference 1 - 0.72% Coverage

-I think this is something that has been discussed a lot recently. I think when somebody has— when somebody is identified as at high risk, that can then—sometimes—that can be slightly overstated. And exaggerated.
And once a person has a label of being at high risk then the—a downside, can be that there can be quite a lot of staff anxiety around them and decisions that can be made might often be quite conservative, quite risk averse, and not always in the best interests of, say, improving the persons situation, but more containing the risk.

Reference 2 - 1.12% Coverage

Well, the obvious barrier that I can think of is just lack of awareness about risk. Lack of awareness about, so this is in the context of depression, so lack—lack of awareness about mental health conditions, and associated factors like risk, is probably a large barrier. Maybe the largest barrier perhaps.
I: How do you think we can kind of start to overcome that?
P: Well, I think there are a lot of campaigns already that raise awareness. I think, you know, as there are many campaigns to raise awareness about mental health conditions, I think that's really vital. That's—that's probably going to do some good, to make people more aware. So that's both a barrier and a facilitator, I guess. Yeah, I can't think much beyond the many ways in which one might raise awareness. I'm wondering if that doesn't really say very much? It's sort of obvious.

Reference 3 - 0.64% Coverage

I think perhaps, also, it can be quite a difficult subject for people. And, perhaps naturally, it's a subject people will feel quite uncomfortable with and may sort of avoid. So, understandably some people may—might find that quite an upsetting topic, or might find it a topic that they just don't want to talk about. I think that can be a bit of a barrier…people maybe want to, there may be some people who just want to look the other way and pretend that such a thing does not exist.

<Files\\Healthcare Workers\\ > - § 6 references coded [2.41% Coverage]

Reference 1 - 0.29% Coverage

Like, the risks that—the risks with any tool, like it shouldn't be totally relied on, it kind of answers differently. Erm, yeah, so that you'd miss out some people, or flag up other people that didn't need. Yeah.

Reference 2 - 0.47% Coverage

I think that people are sometimes scared of identifying things early, because there's already huge waiting lists and, like, if you kind of start finding people, then that's quite a scary thought for some practitioners. But then that—then it's, and there's kind of no way of knowing if it would've, you can't predict whether it would have really…

Reference 3 - 0.35% Coverage

Like, we think that early intervention will help, but like—and preventative work would help—but is there a way of actually, kind of, quantifying that? Because you can't do both, with the same person and work it out. Yeah. So, I guess people might be afraid.

Reference 4 - 0.27% Coverage

Yeah. So, yeah, maybe people being frightened that they'll create more work than they could cope with. And I suppose it would be a way of finding, making it quite like easy to administer. Yeah. I:

Reference 5 - 0.44% Coverage

Yeah. So, for the—I think it might have to be sep—I don't know! I actually don't know! Like, and it's such a terrible way of thinking it being, you know, like part of a…it's like really unhealthy to think, like, (whispers) "we mustn't find out, because that could cause so much trouble!" But that is actually what happens.

Reference 6 - 0.59% Coverage

Because we’ve been doing lots of work on the waiting list recently, and people have very funny fears about like waiting lists and like unearthing stuff that they don’t want to know about. I think that that could be similar, like there is a kind of fear, then again—I don’t know how you’d get around that. Maybe by—because you would, you would. You would find people who hadn’t been kind of noticed, wouldn’t you? If you were looking.

<Files\\Healthcare Workers\\> - § 2 references coded [1.20% Coverage]

Reference 1 - 0.54% Coverage

So, definitely for families where…where there’s a—for different reasons, lots of families have different views on things like depression for instance—and that might be a barrier to sort of accepting that people are sort of high risk or low risk. Or families where there’s a high burden of depression in…in parents, or in other family members as well, that could make things quite…quite difficult, in order to sort of, yeah, access families to be able to sort of put work in place.

Reference 2 - 0.66% Coverage

But, again, that’s not the be all and end all. I think that there still is a lot to be done in terms of increasing awareness and education around mental health problems and depression. And I think, on the one hand, although increasing awareness of mental health problems itself is one thing, I think what we’ve started to sometimes tended to see is that just awareness on its own hasn’t necessarily been a good thing. Because I think that increases people’s sensitivity to thinking that maybe they’re suffering from problems that aren’t—aren’t necessarily what they are struggling with.

<Files\\Healthcare Workers\\> - § 3 references coded [2.09% Coverage]

Reference 1 - 0.54% Coverage

And I think it’s too early for midwives to be doing it, because then you’re going on predictors and you don’t know whether they’re going to be subject—in my mind it’s things like ACEs and stuff like that, that are sort of—and of course family history is important, but everyone knows that already anyway. So, it’s not rocket science. You don’t need a screening questionnaire to know whether somebody’s got a family history of bipolar for instance or something. Yeah.

Reference 2 - 0.85% Coverage

So, if they’re being enticed to have almost no sleep through screens being ever more compelling, if they’re being enticed to overeat and are becoming physically more and more unfit and overweight so they’re not taking exercise—those things can have a much bigger effect on their chances of getting depressed than a bit of fiddling around the edges that the family might do. And, you know, if both parents are unemployed, and, you know, there's unexplained physical symptoms in one parent, so the children have to become a carer, and maybe the other one’s depressed and mentally unavailable for the child, I mean—I don’t know—I mean you don’t need a screening questionnaire to know that that’s just a disaster waiting to happen.

Reference 3 - 0.70% Coverage

That’s the thing. I mean, that's the difficulty for me is most—most of these children, you can predict who’s at risk of getting depression, if you just took a cohort and you were given data about their ACEs, their socio-economic status. You can say without a screening tool, “Ok, I—he’s more at risk, she’s more at risk, he’s more-” you know, like that.
And you don’t—you don’t need—well unless the tool is just a way of kind of helping those that don’t know how to look for those things to look for them. And I'm not sure what…what kind of level of scrutiny the tool is going to involve anyway, so.

<Files\\Healthcare Workers\\> - § 2 references coded [2.01% Coverage]

Reference 1 - 0.35% Coverage

Acceptability, as I say, I think there’s some challenge around the type of question being asked, but then I don’t know how one asks them any differently? You know?

Reference 2 - 1.66% Coverage

And again, you know, is it—it’s only so useful in how you act on it. There is no utility in being told you’re high-risk for depression unless you do something about it. And again, there is a subtle difference here between, you know—and you’ve said that it’s only if you're experiencing things that are causing you distress (pointing to the high-risk information in prototype)—and I think that’s important because, obviously, being told you’re high-risk for depression is very different from being told you’re depressed, and what follows suggests that they are depressed. So, speak to Childline and check out this website. Well, preventing depression is good, speak to Childline, again, you know they might not need to speak to Childline yet, but I guess they’ve got information should it become an issue.

<Files\\Healthcare Workers\\> - § 3 references coded [1.70% Coverage]

Reference 1 - 0.38% Coverage

I don’t think in any way it would be a benefit for a parent, because that just gives them more things to worry about, more things to, you know, think might be a problem. And if you’re ticking boxes that say, “we—we’re in a low income, we’re in a deprived area, we’ve got this, we’ve got that”, it doesn’t necessarily mean that your child is going to have mental health issues but it does mean that—or depression—but it does mean they’re probably more susceptible to it.

Reference 2 - 0.53% Coverage

But this is where it’s difficult putting people in boxes—they’ve got to be a broad—they’ve got to be quite broad in that way. So, I imagine why all this research is going on so that you can sort of identify—identify the right criteria really—because as it stands at the minute, I don’t think that that could be done without this sort of research. Because I think it would probably, it might, you know—if a parent, if some of the parents that I see, that send their kids over here—if they were doing that sort of thing, they’d either say

be really worried, or they'd say "no, that's not my child". And it would be one or the other—maybe deny it completely.

Reference 3 - 0.79% Coverage

Because there's not an answer. I mean, and people—people get very scared when they think that somebody's cutting themselves or they're going to kill themselves—and people don't like to use that sort of language. They like to say, "do you think", you know, "have you thought about not being here anymore".
And that's a bit like when you're in hospital and you're taught to say that somebody has died— actually use the word death and died—because if you say, if you use flowery language, it doesn't always go in. It doesn't always get the meaning that you want across, and I think people are scared of what happens if someone says to me, "I'm cutting myself" or, "yes, I've thought about suicide".
And I think part of that is also the culture that we've got nowadays is that people are scared of not reporting, reporting too much, breaking confidentiality, of what they're supposed to do because, you know, there's ramifications for them—rather than the young person—as well.

<Files\\Healthcare Workers\\> - § 3 references coded [1.65% Coverage]

Reference 1 - 0.23% Coverage

So, the questions that we have that are relating to depression are already patient driven, or chronic disease driven, rather than full screening. Because full screening comes with down sides of false positives, really.

Reference 2 - 0.79% Coverage

Mmm. It's a concise way of putting something isn't it? It's difficult because if you put it any other way, it becomes—it can become a bit woolly. I think—I think, there's a—I mean a lot of people who are quite low are going to come from, often, lower socio-economic classes, often and I think within that group, there's a very anti-authoritarian feel, or attitude, really. And I think sometimes, in the prison system quite a lot of the time I spend telling people we are actually quite worried about you, and we're not the officers—we're not the criminal justice system—we are a little bit separate from that, and we actually are worried about you. And that might be quite a nice tone to bring through in the high-risk side of things.

Reference 3 - 0.64% Coverage

But, yeah, it is a big responsibility. But I think everybody's quite scared of that responsibility because everybody feels like they're going to have it thrown back in their faces if they take an interest. Everybody's covering their back and passing the buck all the time, aren't they? When actually, having the time to sit down with them and sort something out—because it is complex—because if you don't go and tell the parent, "Huh, your child's depressed", I mean the parenting is probably poor for them to be in that situation in the first place. So, what do you—they're not even going to be able to deal with it.

<Files\\Healthcare Workers\\> - § 1 reference coded [0.75% Coverage]

Reference 1 - 0.75% Coverage

And I thought, well that must have been really difficult for the girl, the young girl, and then that's—that was them kind of saying they didn't know what to say, or how to say it, so they avoided her. And then obviously that has the knock-on effect to compounding that girl's feelings of being insecure and her confusion about the whole situation.

<Files\\Healthcare Workers\\> - § 1 reference coded [0.44% Coverage]

Reference 1 - 0.44% Coverage

Well I think these are really hard topics to talk about, you know? And I'm very much aware that because I've been doing this for a long time, like I can easily ask really hard questions about people's personal, psychological well-being. But that doesn't mean that other people find that easy to tolerate, you know?

<Files\\Healthcare Workers\\> - § 5 references coded [4.08% Coverage]

Reference 1 - 0.50% Coverage

So, yeah, I don't know—and I guess—I don't know if it's stigma; whether people are still afraid of a child saying, "I'm going to kill myself, I'm going to self-harm", you know—obviously that kind of thing we get a lot of that—there's lots of children saying those things, and referrals coming straight through. And I think social workers panic and it's like a bit of a knee-jerk reaction—they feel that they're not able to—they're not going to be able to cope or manage with the distress, or be able to offer the appropriate support?

Reference 2 - 0.36% Coverage

I guess it depends on the professional, and I think a lot of it is down to their sort of ability to…feel comfortable with the subject, or with mental health. And then I guess that really depends on their kind of—their own experiences—their own life experiences, and what mental health means to them. It's quite a personal thing isn't it? In a lot of ways, I think, mental health

Reference 3 - 2.03% Coverage

It sort of felt like safeguarding—some of the questions were sort of—sounded quite like safeguarding. And I kind of wondered, because I guess the thing is you don't want it to be too long, do you? Because I guess it would put a young person off, and I guess are you trying to aim for a snapshot, sort of trying to make it quite short?

But I, there was a couple of things that I wrote down that I wondered. Because there wasn't any mention of anything around, sort of parents—parenting, sort of—parents mental health. What else was there? And about parents drinking or taking drugs, but I guess I don't know whether you'd ask a young person about that, or whether they would know? It could be a tricky one couldn't it! Because I think from my experience, a lot of young people that come to us with mental health issues often have parents who have mental health issues of their own, or have drug and alcohol issues.

Or who might not have been in a physical fight like the question suggests, but may have been prone to years of bullying in other ways? It's very kind of yes or no—the questionnaire—and it may be that the child hasn't had a fight but that they've actually been bullied through the internet for months, or been emotionally abused, or called names for years.

And also, poverty and accommodation and, sort of—I know they're on there, there was a question about food and going hungry—but, I wondered about accommodation? Because that features—it's quite a big

one on our, sort of, with us as well—around sort of children in poverty, kind of living in really sort of inadequate accommodation with parents who have mental health issues. And I didn't sort of see any questions that might relate to that.
And also, children with additional needs? Because we get a lot of children with additional needs who also have a lot of anxiety and who get socially excluded from school, who then sort of fall into a depression. Then learning disabilities, health issues, young carers. And also, the looked after children population as well? But I guess if you asked all those questions, it would become a bit of an essay then, wouldn't it?

Reference 4 - 0.80% Coverage

Yeah. I guess you'd need—I guess with anything, it's like with anything isn't it—I guess it wouldn't stand alone. I guess we all rely on different ways of measuring risk and working with risk, so, like with us here, we use SDQs [Strengths and Difficulties Questionnaire] but we also use CGAS [Children's Global Assessment Scale] and HONOSCA [Health of the Nation Outcome Scales for Children and Adolescents] and then we use subscales.
So rather than relying on one questionnaire, we tend to use a variety of different things to kind of get a picture of, kind of, what might be going on, or what might be the cause, the severity, etc. I think it's a really good idea in principal. I think it's, you know, a really interesting project that's well needed—I think there is a gap there—but yeah. I guess it's just kind of how it reaches young people?

Reference 5 - 0.39% Coverage

Because I guess the thing is as well, with the questionnaire, because they're going through adolescence, you know, that they might be a bit biased? Or they might be having a bit of a bad day,
so the following day could look very differently if they filled it out. So, I don't know, I guess, the parent might also want to share information if they have concerns. And the professional, the teacher, or the GP.

<Files\\Parents\\Tracnscription > - § 4 references coded [3.74% Coverage]

Reference 1 - 0.20% Coverage

Or they may not want to open that can of worms without a reason, this could be a potential reason.

Reference 2 - 0.31% Coverage

Because I mean, it's of benefit to the school, if you've got kids who are at high risk of depression to check in with them. I mean, massively important.

Reference 3 - 2.87% Coverage

I wonder though, there'd be some, like our other research groups works with maltreatment, with maltreated kids, and I'm wondering if, there could be a chance like, obviously, the parent themselves would never see the questions that were being asked, but I think there would be some elements of like, dredging up difficult situations and potentially if the child gives that back and it's like, oh I did this online quiz and I remembered about how dad was beating you up, or etc. etc. It could potentially open up

some very sensitive things within a family, and then, yeah, I think that would have to be maybe thought about or navigated. Yeah. Yeah. Like, within our family, obviously, it's not an issue, but like have you ever seen adults fighting in your home or fighting with other children in your home, and then you ask them to tick that and then they go and they're like, oh I did this questionnaire, it was asking about this. I just wonder as some of our more vulnerable parents, how they would feel about that. Like, we've had parents who have gotten grumpy with us about asking some of these kinds of questions with their kids because it's dredged up sensitive things that they've kind of put to bed. And that they prefer their child not to have to relive and revisit, so, I can imagine for some families they would, they could potentially find this difficult for the kid to go through.

Reference 4 - 0.36% Coverage

Yeah, maybe. Because I just feel like once you've opened the can of worms, I guess I just always think back to like, you know the duty of care and the risk involved in asking.

<Files\\Parents\\Transcription > - § 9 references coded [35.92% Coverage]

Reference 1 - 1.64% Coverage

No, I feel that you, the risk really misses the most important aspect because, in a way, general social isolation, I mean, you could define differently. It could be in terms of the family just don't have any friends so they don't go out much, so they really just stay home and that's it. Erm, now, that's different from, erm, bullies and discrimination kind of negative experiences. Because you can be socially isolated but not experience bullies or none of this, and you can have quite a social, active life and experience all these events.

Reference 2 - 16.07% Coverage

If I get upset and raise my voice to my children, then I will, they know why. So, they always understand that they did something wrong, or something that is not appropriate. So, even when you define fights or so, I mean when you define fights are you talking about arguing, usual arguing? Are you talking about, I don't know, physically abusing the other partner? Physically abusing the child? And, I can see the question on whether or not they require medical care, or so, I feel even that question, well I'm talking too much from a research point of view, rather than as a parent! As a parent, again, if, if I slap, well I wouldn't slap, no, no, no, not now that they are teenagers, but when, as a baby, or so. Erm, yeah, I suppose how would you? Would I report? No I suppose I wouldn't report if I would just kind of give them like a mild slap or something like [imitates slap], you know, just touching them a bit, I don't think I would say that well, I did. I don't know how my children would, would sort of see that though? I suppose if, if, erm, if I suppose there is no reason, I just go and slap them, or like, well, no, slapping is not the right word because it's too harsh, like something like, no, just basically kind of maybe I don't know, like physically pushing them or doing something maybe that I don't know. I suppose if they couldn't explain, probably, I can see that they would, erm, report it. But there's an interesting theory kind of related to that with physical abuse, so physical punishment. So, but it's an issue in terms, I don't know how they would see, they would interpret anything physically when it comes to it? Erm. I don't think you ask

anything about sexual abuse? You touch about this, you ask the question about whether somebody touched you, or things. Again, well what would that mean? So, for instance, like now and again, when I lay in bed, my son comes and lays in bed with me. Not, I suppose in certain cultures, you're aware of, that's totally inappropriate. In our culture, because we are more expressive emotionally, it's fine for, you know, even if it's a teenager son to come and lay in bed with me, like. I mean that's I think, it's also another issue, but, again, I don't know how much you can expand on this if, if I think from a teenager point of view, maybe some of the questions would benefit from maybe further sort of detailing or maybe giving some examples? What, what your, what the question is about rather than just, erm, reporting. Because I- my feeling is that if they, sometimes they, how can I say, for the arguments sake, my wife, I don't know, or I tell her off for certain things, now if the question, they, maybe they misunderstand it as parents fighting, he say yes they fight, and they might respond positive to quite a few of your questions and then they come as high risk score for depression, but in theory they are fine, you know, so nothing is wrong with them. I think, that's where probably it might influence a bit the classification but that's. I mean, again, it's a personal opinion, just trying to, erm, remember some aspects of my children's behaviour and in the way, how they see things differently according to, erm, their kind of mental attitude and attitude towards relationships in general.

Reference 3 - 1.41% Coverage

In terms of, you know, either, erm, not necessarily bullying, but kind of being a bit, erm, bullish, probably the way, or just, erm, I don't know what is the right word, maybe it is, but I feel that one definitely, I would capture the, erm, the school and then maybe parental support. I didn't get much of the question in terms of, how much support and understanding they get from parents, sorry, I might have missed completely some of the questions that you asked-

Reference 4 - 2.19% Coverage

So, again, that's the other aspect, I suppose, in terms of, I don't know even, is housing, like, or the area where they live as well, like? In terms of depression, no I suppose it's a bit, yeah, it still comes down to friends if I think about it. Friends and the school context. How well they are doing.

Reference 5 - 2.57% Coverage

Would she understand all of the questions? Possibly. Probably. She could understand, but as I said, I think my main kind of concern was in terms of how they would interpret some of the questions, in terms of family or parents, erm, fighting or so. Is it- As I said, it's just fighting the main, the first thing that comes into mind with my children, like physical fighting, would they call it sort of really arguing, or so, as a fight? That's it I suppose, it would be, but that's again, as I feel that you might have some culturally biased responses there in terms of, in certain cultures, it's ok for instance for parents to maybe raise their voice or tell off the other one, in other cultures maybe less. So, I think that's an issue, maybe that's just trying to help them understand what kind of events you might be interested to get from them.

Reference 6 - 3.25% Coverage

Oh yeah, it's that sort of self-, kind of self-perception, you know, appearance, anything in terms of how they self-image? I think that's, erm, another aspect maybe that you might, it might capture a lot of some of the actual experiences that you're asking them to report. But maybe some of them might be less likely to, erm, disclose some of the questions you're asking them, or something, whether if you ask a question in terms of more how they feel about themselves, like their self-image, in terms of physical appearance? Erm. So, I feel maybe that's one question which might not be necessarily related to, directly, to depression, but it can be something that maybe it might capture some certain other aspects that maybe you might not capture through the questions you ask.

Reference 7 - 0.94% Coverage

Yes and no. I think I would have issues in a way, if they would disclose anything about maltreatment, or so, which, erm, there wouldn't be anyone to kind of help them or explain the question or what does this mean. I think that I would worry a bit myself in terms of, well, how that reflects on me as a parent.

Reference 8 - 5.16% Coverage

But I can see that I suppose if you're, if, you know, you're a parent that, you know, and you might misunderstand some of the question or what you are trying to get, I can see them being unhappy to allow their children to report, erm, to fill in some of these questions. So, yeah. I think, yeah, I would, as I said, I might not be the most representative person, or parent to speak to, but, erm, I can see, I can feel that yeah, probably certain things, if my children would misreport, I would feel uneasy about. I would feel well, this is not actually what goes on. Now, that's the kind of thing that in a way it kind of made me feel that I'm a very kind of strict, or severe, parent, or so, and that, I think is in a way, there would be something similar that probably I feel that some of the parents might feel about some of the questions. I think, of course, everything that is related to the family, I suppose as a parent sometimes, if, if you are the ideal parent, you never sort of do anything like that, it's fine, but if you now and then, you, sort of, are a bit harsh, or so, you punish your children or so, you might feel a bit, well, no because I don't want to kind of, erm, be seen as, as a you know, that I'm, I don't know, abusing my children, or I don't provide the best, or a safe environment for them, so yeah. I suppose to answer your question, yeah, I suppose I can, I would be happy with certain reservation probably.

Reference 9 - 2.70% Coverage

But, if the case with parents, and those are low risk again, but I feel like the intermediary group of children who are maybe, might be on the border, or, I think that's quite an interesting one and probably might be the largest number of teenagers, that, erm, probably it would be quite interesting to understand, erm, who among those kind of move into the high risk, or, stay like, you know, go into the low risk. I think that that's an issue that is the same with some of the cardiovascular risk scores, in terms of, they are quite poor at discriminating amongst those who are, which is the largest group, so they are quite good at picking up the 10, 20% at high risk, and they are quite good at kind of, again, excluding the

10, 20% at low risk of cardiovascular disease, whereas those who are in the middle, the rest like 60, 80%, they are very poor at sort of trying to discriminate.

<Files\\Parents\\Transcription > - § 4 references coded [12.46% Coverage]

Reference 1 - 2.88% Coverage

It's hard to say, really. The thing is, I'm sort of worried in a way that if things get, you know, if [my daughter] goes down I'm a bit concerned about people like social services and things because it also affects the family—the parents as well. Because, you know, they, there is always the criticism that these things always stem within the parents, parenting and home life and everything like that, so it is a worry being a parent. Because, you know, I get such a, it's, that is the worry. Instead of finding help for [my daughter] it's sort of making the situation worse for the parents, you know? Because all these things, it just goes un—that's the way these systems work at the moment, which is not really—it purports to help the child but at the same time it sort of tries to destroy the impact of family, you know? And I've seen this happen with others, other parents, you know, that I spoke to.

Reference 2 - 5.03% Coverage

Yes, it is. It's just things seem to be, some things can just escalate totally out of hand. So, I have seen things. Instead of support, it's just, you know, I don't know, things just seem to—well, this is the way the system works at the moment. It's a very difficult thing bringing a child up in this kind of, you know, life at the moment. It's very difficult. Especially when you're a single parent, you know. You just want the very best for that child, and that's all. Sorry, rambling on a bit!

Reference 3 - 0.95% Coverage

Yes, it's always the parents that are always to blame if a child is not doing well at school or whatever, you know? It's quite frightening and ferocious, it is, the parenting situation, you know? The way the laws and things, you know, it's just really bad. And it's not really helpful in any way.

Reference 4 - 3.60% Coverage

I think so. As I said, it can do. Coming out, there'd be far more analysis of, after you've answered all the questions, at what risk is the child. It's just a bit—it is good to make an informed decision, but it's just making sure that it makes the right decision because it's too, you know, I'm sure you've already been through it all already. So, low risk or high risk, that's—yeah. And then, you know, what happens if suddenly you're at high risk? I mean, suddenly the panic button suddenly sets people off, "Oh my god, my child is really in that—that state? Right now? Where she would really now need the requirements of all these services?" You know? I don't know, it's a difficult one, I'm hoping fortunately with my child that she will just overcome all this and just do her best. But, yeah, I'm not really quite sure, really. But, you know, it is a trial, if we don't test these things, you'll never know will you? But as I said, I don't think they

can actually do this on their own—there would have to be somebody working along to tick these boxes for them and all that. Hmm. Sorry, I'm a bit sort of lost over what to say.

<Files\\Parents\\Transcription > - § 6 references coded [19.37% Coverage]

Reference 1 - 3.15% Coverage

I thought it was very basic. Really basic. Some of these questions could literally just be, are you in care? Ok. Yes. You know? That could just be it. Yeah. I thought there would be more questions, to be fair. And a bit more personal, about how do you feel on a regular basis, or, just, questions like this. But, as I said, a lot of these questions, not saying that everyone in care would necessarily fall into these categories, but, yeah. It just seems quite basic. I'd expect more questions, more in depth, questions about the emotions, justifying depression, or else, yeah. And, especially at that age, as well. How's your relationship with your parents? I mean, isn't it most teenagers that hate their parents, anyway? Or don't think they agree with them? So. Yeah. (*laughs*) I mean, how's your relationship with your parent, your mother, *overall* maybe. Not, how's your relationship with your mother today? You know? They could hate them today. Or on other days it could be very good. So, yeah, that's my general thought on it.

Reference 2 - 2.91% Coverage

Yeah! And if it does do it, it would be quite worrying if, well, not only that, I think a lot, I mean, I don't know how they score it, so I can't really say, but I can imagine, depending on what day you actually score this, there'll be a lot of children who are high risk. And I imagine a lot of kids will be like, "Oh, I'm going to suffer from depression", not dismissing that because a lot of depression does happen, but I can imagine a lot of people will be coming up with high risk of depression and that's not true. Well, it might be, but I just think it's basic, basically. And in terms of the high risk, I would expect, I was hoping it would be a bit more than just pointing them into a direction of just ChildLine, or Preventing Depression (*indicating a website*). Yeah. I mean, I suppose it is what it is, there's nothing really you can do about it, obviously you can't say, "Call me" or, but yeah. That's my general thought on that question.

Reference 3 - 4.21% Coverage

Well, one you might be experiencing problems. But, I also think a lot of these questions, it just depends on how you feel on a given day. As I said, in terms of your relationship with your mother, in terms of your relationship with your father. Have you ever run away from home for instance for more than one night, again, if someone's in care, or someone, you can imagine they probably are more likely to be a runaway from home, or, you know, had some sort of, you know, been beaten and stuff. So, then it just means that everyone in care is going to be depressed, which, or will later on be depressed, and, yeah. So, I just think there needs to be just more questions, just more in depth questions, about how do you feel on a given day for instance, or, yeah. I don't know, I just think there can be more questions about their feelings and have you ever thought about, you know, hurting yourself, or, I mean, school and social experiences, have you dropped out of school, or repeated a year, could there be more? Yeah. Could there not be more about school and social experiences? Do you have someone you can talk to, for instance? Yeah. I mean, whoever done this, obviously they know better than I do, but I would expect

there to be more for instance on school, and social experiences. Just more questions about are you doing well in school, yeah. That’s just my thoughts.

Reference 4 - 5.72% Coverage

At the way, as it currently stands…I don’t think it’s necessarily serious enough to be…this could be on an NHS website, for instance. Not saying that it’s not serious, I know I just said it’s not being serious, this could be on an NHS website, for instance. It could also be in whatever young children are interested in, I don’t know! (*laughs*) On social media? But then I think it’s not something to be taken lightly, to just put on social media, but then if you need it to be accessible, then it should be out there as widely as possible. But, I think, as it, I just think it’s a lot to be something that’s not necessarily that serious, with a high risk, to just use that, I think it needs to have a bit more depth to it. If, I mean, telling someone that they could be at high risk of depression, that’s quite a lot for a 12 year old, for instance, to handle. And especially reading this now, I would definitely shouldn’t be for anyone, a parent, actually maybe a parent, but it should definitely not be for anyone that’s younger than 12 to complete, because there’s just not a lot of information. And when do they…It doesn’t even say what depression is, for instance, in this high risk (*gestures to the relevant section on the risk calculator*), so they can identify it when they are depressed, and then they could contact them. I mean, are you saying that they should…I mean…it’s saying high risk of depression, but this is about will they, future risk of depression, like just say, if any of the questions you responded positive to are causing you distress, so I suppose it’s now and later. So, it’s, just reading that, I would say, even more brings the idea that it should have some of those NHS questions into this. Yeah. I think it should definitely be on the NHS website. But, that's the only place that I can really think it sits and I’d be comfortable with it.

Reference 5 - 1.66% Coverage

Yeah! It’s a heavy issue! And it’s a heavy issue and it’s something that people are talking about a lot nowadays which is all really positive, and that's why I think this as, even risk calculator for depression, I just think, the term depression should not be taken lightly. And, just reading some of these questions, I just don’t think it necessarily shows that gravitas—that importance—of what the question is. It just seems, I mean, there’s 17 questions, and very quick questions as well. I just think there could be more depth, that’s all.

Reference 6 - 1.71% Coverage

Yeah. Yeah. I mean, some of these questions are really, you know, quite hard, and some kids would find them quite difficult to answer. But, yeah. I just think there could be more questions. Because it’s about how external factors, not about how they’re feeling, or what they are going through necessarily, I mean, yeah. I just feel it’s all external and not about their thoughts and feelings. And I think, I don’t know, I'm not an expert, but I would think that also contributes as to how they will feel, or what theire at risk of depression will be like.

<Files\\Parents\\Transcription > - § 4 references coded [3.91% Coverage]

Reference 1 - 1.27% Coverage

It seemed quite stark in terms of the options that it was offering people. To be honest, I don't know. Because I've not looked at anything like that before, so I'm not, it seems quite a quick way of deciding a high or a low risk of depression but I imagine if people answered positively or negatively, I'm not sure which I mean now, but if the result said high risk of depression, it might be fairly obvious from the routes that have taken you there. If you see what I mean?

Reference 2 - 0.68% Coverage

I think some of those questions were pretty brutal in terms of, you know, if you're a parent, and if the, if some of the causes of the depression are as they are in the tool, you probably wouldn't feel comfortable because it would expose you as a parent.

Reference 3 - 0.67% Coverage

I suppose it could be used at schools, but again it's hard to imagine, for me, anyway, the kind of environment within a school that would enable a child to feel safe enough to use something like that. Because it's quite stark information you're asking.

Reference 4 - 1.28% Coverage

I know I would find it acceptable, but then that's with hindsight I guess, and I know what we've been through. To be honest, I don't know. I really don't know. I think British people have a reputation for wanting to keep things quite private, so they might find that too intrusive, I don't know. I think it would acceptable, to me.

<Files\\Parents\\Transcription > - § 3 references coded [2.92% Coverage]

Reference 1 - 0.85% Coverage

Yeah, apart from the thing I said that I didn't think it made it clear whether that was, you know, to say fighting, what does that mean kind of thing? And I expect, when you said at the beginning, I expected at the end for it to do something more than just give you like an email address and a telephone number.

Reference 2 - 0.96% Coverage

Oh absolutely, yeah. The only thing I think, but then maybe that's because I'm looking at it through my lens is I would think those things would be obvious that that would be high risk. But then obviously, as I'm saying, I'm looking at it through my lens which is possibly very different to someone who has no experience of depression or anything else.

Reference 3 - 1.10% Coverage

Because I think, as I say, I live in a world where depression is fairly normal, and nearly everybody in my family's suffered from depression, so to us it's something we've dealt with for a long, long time. But I know when I speak to other parents who have just got, you know, never had that experience, when the kids are just being kind of kids, just being teenagers, it's kind of really misinterpreted.

<Files\\Parents\\Transcription > - § 2 references coded [1.33% Coverage]

Reference 1 - 0.60% Coverage

Mmm. Because this is the family one? (*looking through risk* calculator) No, that's school. So maybe, so that's family (*reading risk calculator*) so the one where, let me see. I suppose that might cover all that, but maybe like, do you have friends, or do you, even if you do, do you, because some people think they have friends and they don't have friends.

Reference 2 - 0.73% Coverage

Well, if you asked the children then they might lie. Because they might, if someone did something bad to them, maybe they were asked not to say anything, I don't know. Or, so then you might think, oh no, that's alright and then, so you've said nothing's happened and you think, ok they're not really at risk. Like. Because I suppose you can't, unless you know, so I suppose they can lie. Or you've caught them doing it before. Yeah.

<Files\\Parents\\Transcription > - § 1 reference coded [0.51% Coverage]

Reference 1 - 0.51% Coverage

But I'm not sure that this is a first port of call. I feel this is quite, it's quite challenging, I think. And I think could stir up all sorts of emotions, and, yeah, yes. I would say I would be cautious.

<Files\\Parents\\Transcription > - § 2 references coded [0.94% Coverage]

Reference 1 - 0.49% Coverage

Well, from what you said, I thought it was going to be more in depth. And, so it was less detailed than I expected it to be. And, yeah, I suppose a lot of questions to begin with are the things that you might think of that would make you at risk.

Reference 2 - 0.45% Coverage

Well, I suppose if it’s a part of something bigger, that’s, you know, it’s going to be trivialised even if it’s not. Somebody’s going to find it on a website somewhere and post it and laugh at it and use it in a trivial way.

<Files\\Parents\\Transcrirption > - § 1 reference coded [2.50% Coverage]

Reference 1 - 2.50% Coverage

Well, you can miss the high risk, maybe, I don’t know, if you just use that. But I think it’s again, it’s a screening tool, and you use it as like this, but sometimes you can, I don’t know, you can say by answering these, how many, 20 questions (*laughs*) I know that people thought about it, but you can say like how come, by answering 20 questions and then someone say, ok you don’t have a risk, or maybe you have a low risk of, “But I feel…”, like “I feel really bad!”. Like, what does it, like what does it mean? Do I only have a lower risk, or. I'm thinking also, like if you don’t think you have something and then, oh, you are in a high risk for depression, and “Oh, I never thought about that!”. Again, it’s not out of the blue, but I think that sometimes, and also maybe you can miss people in between, I'm not sure, but, yeah.

<Files\\School Workers\\Policy Makers\\Transcription > - § 4 references coded [3.89% Coverage]

Reference 1 - 0.95% Coverage

And it could be quite, yeah, stigmatising. Because, yeah. And some of the, a lot of these risk factors are through no fault of the, you know, not through the child’s own doing, and yet they are being stigmatised for maybe their upbringing and things they have no control over.

Reference 2 - 1.39% Coverage

And actually should we be treating every young person like that, because it’s so prevalent anyway. And you don’t have to have all those risk factors to actually develop depression, you don’t have to have any, it could just be a biological thing. It also doesn’t ask in the risk calculator about—does it ask about any family members? Immediate family members that have suffered from mental health issues?

Reference 3 - 0.56% Coverage

I think that would be quite useful, because obviously it’s, there is genetics involved in that. So, I think that would be quite important as well as a risk factor.

Reference 4 - 0.98% Coverage

Yeah. Ok. I think there’s something in having a question about whether someone in your family has suffered from a diagnosed mental health condition. I think that, because that is actually a sign, especially if one of your parents have been diagnosed, I think that’s definitely a factor.

<Files\\School Workers\\Policy Makers\\Transcription > - § 2 references coded [5.71% Coverage]

Reference 1 - 1.82% Coverage

I wouldn't be sure what this would add above and beyond a kind of conversation with a student, you know, a qualitative approach to understanding who a student is, you know, their kind of relationships, you know, familial, socio-economic, cultural relationships that they come from, which, you know, if it's done well by a teacher or a youth worker or, you know, by you know, even by health workers, this sort of approach, I think, you know, is very valuable too. And I guess my concern would be that if we were seeking to replace the sort of qualitative contextual conversations which I think teachers and parents and others would be having with their, with the adolescent then I think that could be a concern as well.

Reference 2 - 3.89% Coverage

I think it really depends on how you go about it. So, I would be concerned that if you introduce this as an intervention as it currently is, you know, in a very kind of widespread way, there is such inequality in society that you can imagine some people finding this incredibly helpful and then saying, "Oh, mum, I've been, you know, found to be high risk of depression." And that leading to private counselling or something like that, that must be, maybe it will be very, very useful for people who have the resources, the support, to deal with this, but I would be very surprised if that was a common experience for many young people. Particularly those who lack resources to, you know, the support, or whatever it is to actually, or the confidence to be able to share this information or to act on this information, so, yeah, I think there is a concern that it might drive further differentiation, you know, along the lines of inequalities that we already are aware of. And I think that, yeah, it sort of, in the absence of broader attempts in public health to kind of act on inequalities and to, you know, invent ways of actually finding ways of providing access to healthcare to the people who probably need it most. Because we're still not very good at doing that. Yeah, in the absence of those broader attempts, there are, you know, real concerns. But, you know, these are the kind of concerns that you can level at healthcare more generally, so I don't know if this, this is probably not just to do with this particular thing.

<Files\\School Workers\\Policy Makers\\Transcription > - § 6 references coded [12.70% Coverage]

Reference 1 - 3.44% Coverage

I felt that it was a little, I did feel that it was a bit blunt if I'm honest. Erm, I, I would be slightly anxious about using it with some of our pupils because I would be concerned about how they might feel having completed something like that? Digitally. Erm. There were quite a lot of trigger points I think in there if you're somebody that is experiencing depression or difficult relationships with people, I mean, I, I understand that these questions are important in terms of clarifying some of those things? But I would be worried about a child just sitting in front of a computer and having to think about how the relationship is with their father, particularly if, say, their father is abusive or violent to them, or, erm, harming them in any other, in any other kind of way. And then obviously talking about, have you seen adults in your home fighting and so on, it just, the, I, again, I just, in terms of, if I was dealing with a

child, say they wanted to talk to me and they said well actually I'm having a really difficult time at home, erm, I wouldn't want to lead them? And some of these questions are potentially quite leading? 'Have you ever seen adults in your home fighting with each other'? I mean it's, it's sort of, important to clarify I suppose what, what those relationships are like at home, but it might lead some children to feel that they needed to report certain things and then that might not be their, their experience I suppose?

Reference 2 - 1.92% Coverage

Yeah, I suppose, I mean if I had a pupil, if I had a pupil with me that I thought actually, I'm slightly concerned about their emotional well-being, they might be vulnerable to depression, then, I mean, I'm lucky that I'm afforded a lot of time in my week to be able to deal with these sorts of, these sorts of things, whereas I appreciate that in some contexts that may not be possible. So, for me, that would be possible. But at the same time, I wouldn't, I just wouldn't ask some of these questions. Because if I said to a pupil, for instance, 'did someone in your family or household ever beat you'? And they said yes, erm, I would be on dodgy ground if that then had to, I mean I would have to then make a referral to social services and, having asked that question, the police wouldn't be very happy with me.

Reference 3 - 3.56% Coverage

Ok, so in terms of, all schools are required to follow the guidance that's called keeping children safe in education, erm, which is kind of the, the, the UK Government's Department for Education interpretation of the Children Act and responses to the kind of safeguarding culture and frameworks, and that's very clear that one should never ask a child a leading question. Now, it's fine to ask children clarification questions, but one shouldn't be directing them in terms of, you know, things like 'have you ever run away from home for one night'? Did you need to, has anyone beat you? Erm, do you have, not have enough food to eat? These kind of things. Because that's considered quite leading? And therefore, if a child said yes to any of those things, I would be required to make a referral to the local authority and if a child had been harmed, or, so on, then it would be under section 47 which is the most serious category, and therefore there would be a strategy meeting with the police. Now, if I've asked those sort of questions, the police would be saying well actually we can't use any of that evidence, in terms of supporting that child around, within the safeguarding framework? So, none of that information would be deemed valid, and would compromise the potential criminal investigation. So, you know, if a child is being beaten up and I've asked that sort of question then actually I could be potentially putting at risk the support that could be put in place for the best interests of that child.

Reference 4 - 1.03% Coverage

So, if I was meeting with a child, and I thought I was concerned about their welfare. I mean, you know, I might say to them things like, you know, tell me how things are at home? Tell me what it's like. Describe, describe what home is like. You know? Erm, who, you know, who supports you at home? Erm. Those sorts of questions, so I'm just, I'm looking for clarification but it's open for the child to describe to me what things are like.

Reference 5 - 1.85% Coverage

I think people are still quite cautious about giving people a diagnosis or a label. So, I think if someone was to, perhaps, be showing symptoms of depression, I think amongst some people, you know, some parts of the staff, well you might think well, I, you clearly, he's clearly stressed, he's anxious, he's not sleeping well, you know, he appears to have lost weight, doesn't seem very happy, erm, you know if you said to them, well do you think he might be experiencing depression? Oh well, I, you know, he's only nine, or whatever, or he's eleven, or thirteen, I'm not sure, you know, I think some people would be reluctant to draw that conclusion because they would see that either as giving a child a label or something deficient, that, you know, that sort of seems quite serious.

Reference 6 - 0.91% Coverage

but I still think there's a lot of work to do there, to, kind of, shift that. And a part of that is a cultural thing in terms of adults, as well? I think children are probably better at talking about mental health than adults are actually. I think there's probably more stigma attached to mental health issues amongst the adult population than there is amongst children themselves. So.

<Files\\School Workers\\Policy Makers\\Transcription > - § 6 references coded [20.26% Coverage]

Reference 1 - 8.05% Coverage

Yeah. I mean, it's very straightforward isn't it? It's a very simple, it's a very simple survey, and there's no kind of room for ambiguity. I suppose, I mean it's really hard, I know that questionnaires are notoriously difficult to construct but things like "How is your relationship with your mother?" So you've got very good, good, regular, but somebody's, I mean I suppose it is all just subjective, but maybe that's the point, it should be subjective in that sense. So, but you know, what would regular be? You know. Where does regular fit on the spectrum, you know? Because one person's regular might be another person's very good. So, that might be a difficult one. The relationship between your parents, I think that that might be quite a hard, it could either be very, very easy for young people or quite difficult, because if they're fighting and hitting each other, then it's quite clear that it might be bad, although having said that, if that's what you've grown up with, that's what you might think, that that's normal. So, and actually, they might have moments where they laugh with each other, so they think oh well then it's regular, because all men hit their wives. Or, do you know? That kind of thing. So, yeah. But then, you know, I was thinking that that's probably my perspective, or our perspective, you know, me and my husband because we didn't grow up in that type of environment, so, in that sense, I think it could be a— it's quite a hard one, if you're a young person, you don't have the experience of other ways of being. Yeah. "Have you ever run away from home?" I mean, these are quite straightforward, aren't they? Yeah. I mean, "Have you ever thought or felt that your parents or caregivers wish you'd never—" So, I suppose, but then I suppose if it's subjective, it's just what they think, it doesn't really matter in that sense, because it's what the individual thinks, so it's from their own personal viewpoint. Yeah.

Reference 2 - 4.04% Coverage

I mean, yeah, that's, I suppose that's part of like, you know, you might not get very accurate information. And then if you have a child that's like, and also, remember kids will be kids and some of them will just like take the piss, you know, and they'll just like write all the negative things and they're completely at high risk, and they're not, they're just, you know, being really challenging, you know? So, but then those that are absolutely. And the problem is that we have already with children, is the, it's not, the problem is not with the children, it's with the adults and how they view the children. A lot of children that are in certainly different kind of ethnic groups, for example, we know this from the research, different genders, you know, already adults, or society, has a deficit view of these particular children. So, you don't want to kind of pile that on, you know, to kids, especially those that are genuinely at risk, you know? So that need to be built up rather than kind of, you know, this is all, I suppose the whole aspect of social justice, that in one, on one hand, yes, you want to kind of help those that are marginalised and make sure that they've got access to all of the resources that everyone else has, and the support that they need, but on the other hand, you're doing them a disservice by singling them out and saying well you're this, so you need that, you know. So I guess that would be my only kind of critique.

Reference 3 - 1.71% Coverage

I think it's quite interesting, the whole concept of screening. And I have quite mixed views about screening because, certainly in terms of health care, you know, it's been, you know, really beneficial for people when you're looking at kind of screening for physical ailments and disease and what-have-you. I think it's much harder to screen for emotional issues or psychological issues in quite the same way, because it's never quite clear cut, and certainly when they're young people. You know, their experiences, they're still very young, they're still developing and it's, things can change, you know, over time.

Reference 4 - 2.86% Coverage

And, you know, the danger is, as you, as we were saying, that it can kind of stigmatise people, and it can lead to a more deficit view of people. So, I'm in two minds about screening for these types of issues, you know. I mean, I think the whole preventative things is very very important, but I think it should be a much more collective, social thing, you know, and the way that, you know, our society is structured is that it's fine for some people and it's not for others and there doesn't seem to be a collective, unified approach to it, unlike in some of the countries like in Scandinavia where they have a slightly different view to this, but they have higher taxes and they have a much stronger kind of welfare system in place which we don't have, well we do, but it's not nearly as strong as it should be, so, so I guess I'm slightly torn with it, you know? I think the idea is really good, and it's really important that we look out for young people, but how we go about doing it, we just have to be really, really careful.

Reference 5 - 1.40% Coverage

I don't think, if I was a parent, I'm not sure how happy I would feel about it. For all the reasons that I just said, you know? Because I think as parents, you know, you need to be talking to your kids all the time,

you know, and finding out, and I know now that it’s harder than ever when they lock themselves in their room with their social media, but, I'm not sure whether a tick boxes are particularly useful. Maybe some parents will find it so, I think if I was a parent, I'd be quite wary about it.

Reference 6 - 2.21% Coverage

Yeah. I think it really depends on the school, on the teacher, on the class, on the phase of schooling, younger teachers, obviously in primary schools for example, with younger children, I mean we know that parents are a lot more involved in their children’s education when they’re a lot younger, they have a lot more regular contact with teachers and as they get older, that tends to slide off, and if you have children, for example, that are from very challenging backgrounds, you know, some parents might feel very grieved by this, and you know, parents that have had really bad experiences at school too, might not want to be told by their teachers how their child is feeling or what their child should do or have, or what-have-you, you know? So, I think it totally depends on the relationship.

<Files\\School Workers\\Transcription - § 6 references coded [11.20% Coverage]

Reference 1 - 2.60% Coverage

Because (*laughs*) anything to do with medical anything, whether it’s mental health or physical health, they’re too frightened, they don’t want to be involved, they don’t want to take that responsibility. It’s the responsibility of it they don’t like. So, they’d rather a medical professional did that sort of thing. So, it probably would be that kind of thing, but school nurses, so school nurses working for the NHS, or whoever the NHS devolves that responsibility to, have to have a clinic, a pop in clinic every week. So one clinic a week in every secondary school they cover. So, secondary age kids do have the opportunity to go and see the school nurse at least once a week, school nurses, they can make appointments for the school nurse to come in for a one to one session with someone on a day that’s not their drop in. So, there’s really good access to the school nurses, so that would probably be the place that the tool would be used, so the child’s showing behavioural problems etc, a referral to the school nurse, they’ve already got the system in place for referrals and in that, it would be quite easy to use. Because they’re used to using these sorts of tools. Does that make sense?

Reference 2 - 3.76% Coverage

I think that kids use, being given this tool, kids can use such things to, oh how shall I say it, exaggerate their own symptoms? To, for whatever end, whether it’s to have some time off school, whether it’s to get their parents off their back, you know, whether it’s, you know, I’ve got depression, you can’t tell me to tidy my room. You know? I mean, that’s making it really basic, but kids are so manipulative, you have to be one step ahead the whole time. So, they will use something like this to manipulate whoever they’re trying to manipulate. And I had that lot in the secondary school that I worked at, I would have kids fabricating anxiety attacks etc. to leave a class when it was getting a bit too heavy because they hadn’t done their homework, or they hadn’t prepared for a test. So, suddenly they’d be having an anxiety attack because they had a test, and in fact, get them back to the medical room, there’s nothing wrong with them, they’d just put it on. So, I can imagine them fabricating this. So, it depends on what

the questions are, so, whatever it would be you're looking for, then they'll go, ooo there's a sign, I can do that. I also have children who were very seriously mentally ill at the same time, so I mean not all kids fabricate, not all kids try and do it. But, yeah. I mean, when you've got children coming to you saying that they think that their head's become detached from their neck, then I think you start to really worry. But, at the same time, you're thinking, are you trying to pull the wool over my eyes or not? So, it's quite easy, to figure out teenagers and when they're lying, but they will use anything to manipulate people. So. That's a very long-winded answer to your question.

Reference 3 - 1.47% Coverage

Sometimes clinicians just look at the tool and don't actually look at the person sitting in front of them. And, you know, the tool is a tool, it's not the be all and end all, it's not the whole thing. You do have to actually take your eyes off the paper and look up at the person there, and I find there is a risk that the clinician is just ticking the boxes or not ticking the boxes as the case may be. But actually not looking at the whole picture and seeing the child. And maybe the child doesn't fit the criteria of the tool, but still is at risk. And you have to use your experience and your knowledge to see beyond the tool and look at the child. Does that make sense?

Reference 4 - 0.64% Coverage

So, I think there's a risk of them becoming dependant on the tool and not actually using their other skills to determine whether the child's at risk. And also, over diagnosing, being so frightened that they're going to miss something that they tick all the boxes. So, it could go either way.

Reference 5 - 1.45% Coverage

But then again, you know, I don't know whether people would want that responsibility. Because they're too frightened. People are too scared of that responsibility. But if they were all aware that this was a tool to signpost them to somebody who would take on the responsibility of doing it—but I think every group, where you have adolescents, whether it's scouts, guides, church, the local running club, sports clubs, they all have a duty of care. So, I don't know about supporting a child through the, actually using the tool, I don't know, you might get one person in each group that would be willing to take it on. But I don't know, I don't know how it would work.

Reference 6 - 1.27% Coverage

They don't want to do the wrong thing. They don't want to label this child, you know? They don't want to—they're too frightened of that responsibility, of being the person that says this child's got a problem. You know? They don't want that responsibility. It's too big. Labelling a child with a mental health problem, or saying I think this child may have a mental problem or be at risk of, you know, it's like oh, well am I right? Do I? Don't I? What are people going to think about me when I do that? Are the parents going to be angry if I point out that their child has a problem?

<Files\\School Workers\\Transcription > - § 2 references coded [3.48% Coverage]

Reference 1 - 1.23% Coverage

Stigma, I think, more than anything. I think people are scared. I think people are scared of it. And people don't necessarily, who are experiencing it want to be associated with somebody who experiences a mental health issue, I don't think it helps everything that you see in the media, a lot of the time, around mass murdered being, having mental health issues, and all about that, the extreme point. But I think it's just that massive stigma around it that frightens people I think.

Reference 2 - 2.26% Coverage

Mixed. I think it depends on, I think it depends on their previous experience, really. And previous contact with social workers. If they've had a history where they've been involved with children's services on child protection, their response to a social worker going into the family home might be very different. But I think it's just about your approach as a professional and, you know, we're here to support and any work they do with us in my role is completely voluntary, it's not statutory, so it's, I think that building relationship is absolutely key in our role. And, you know, some are, they're really grateful, they just want some support, they're not bothered, they're just so grateful that somebody's identified that they need support. So, they're really on board, but then there's others that can be quite challenging dependent on their experiences.

<Files\\School Workers\\Transcription> - § 3 references coded [2.31% Coverage]

Reference 1 - 0.26% Coverage

Yeah, it's really big. Really, really big. Yeah. Because you can get someone to do a questionnaire, but you can't guarantee they'll be honest, you know, so.

Reference 2 - 1.50% Coverage

And also, just the means to, you know, if there's no system, whether it's just a form that you fill in, also if things are lengthy I think it's a barrier. So it's like really, you know, and you have to give all your information, you know, that could be something that doesn't kind of allow you to be confidential, I think, could be a barrier. Because again, it's kind of a thing where people might not feel like, yeah, maybe scared of kind of getting in trouble. That was a very big theme when I was in the care industry. Like, people were scared, there was a kind of hypervigilance of the local authority, or of the social workers, so people were always scared when they were out with their clients, or out with people, so what would happen is that they would act, they would kind of cover up their behaviour as opposed to just being natural or normal, you know I saw that a lot in the care industry. Yeah.

Reference 3 - 0.55% Coverage

Yeah. Yeah. I think so. But also I think it's to do with people not wanting to kind of like almost shame if you feel a little bit maybe insecure about your own mental health as a parent or as a family, yeah, you might not want to explore that in the child. You might feel, maybe—I'm not sure. But, yeah, I think definitely stigma.

<Files\\School Workers\\Transcription > - § 2 references coded [4.20% Coverage]

Reference 1 - 2.12% Coverage

And, you know, I mean, I guess you spoke about some behavioural factors that might indicate depression, I understand there may well be a statistical correlation but there is a slight squeamishness that it might end up labelling people from certain socioeconomic classes. But then, I guess, those from certain socioeconomics classes have a high risk of being depressed. I think there is evidence that depression correlates with socioeconomic status, to some extent. So, yeah. I think it's that kind of, the difficulty perhaps of, I mean, again, I guess it's always, you know, in certain forms of therapy, I guess labelling someone as depressed would be a negotiated thing between, you know, a medical professional and the person themselves. And I guess in other, perhaps more psychiatric models, it might be more just a diagnostic thing of, you know, you meet this criteria, you are depressed. But here, that would be after a clinical one on one kind of consultation.

Reference 2 - 2.08% Coverage

So, I wonder, there's kind of a, the kind of slight difficulty of, you know, if a student did this say at school somewhere and then went home and said "Oh, we did this computer programme and it said I've got a high risk of getting depression", or students would probably go home and say "This computer programme told me I'm depressed" which is probably the kind of thing they'd tell their parents. What potentially, that could be potentially problematic, you know. Because I guess, you know, this is the difference between a diagnosis coming, and I know it's kind of a risk of something, but I mean if this was something that students were directed to and it's kind of like, well, we've talked about mental health today, here's a tool that, you know, looks at the risk factors that might put you at risk of depression. Is there more next steps? Is there? (*Indicates the high and low risk sections on the calculator*)

<Files\\School Workers\\Transcription > - § 2 references coded [1.82% Coverage]

Reference 1 - 0.95% Coverage

Yeah, definitely. Especially the word depressed. Yeah, I think it is used quite a lot. And I think it's, I think, you know, probably has been on the rise for quite a while and that is a, you know, a teenager thing, you know, teenagers being over-dramatic, so. But it's interesting that that word is used rather than worried or anxious or overwhelmed, you know? It is a word with a mental health, you know, well it is a mental health disorder.

Reference 2 - 0.87% Coverage

I guess another concern would be is within a school, is you, within my role for example, I don’t have contact with parents. But I have, they, the students will infrequently feedback to their parents. So, I guess it would be ensuring that there isn't a conversation going home along the lines of, “So and so at school said that I'm depressed”. And causing unnecessary worry and concern in relation to that.

<Files\\School Workers\\Transcription > - § 4 references coded [2.46% Coverage]

Reference 1 - 1.09% Coverage

Yeah. You’ve got to have a relationship because a lot of the times a lot of the families that we work with have had so many different professionals work with them and it’s like a revolving door because there isn't consistency a lot of the time, for lots of different reasons—especially when it’s like social care, and things like that. And they do have a negative view of services, they really do, and understandably so.

Reference 2 - 0.17% Coverage

I suppose people don’t want to know the answers (*laughs*). Yeah.

Reference 3 - 0.42% Coverage

Yeah, and I think sometimes people don’t want to ask those difficult questions, do they? And maybe that’s part of it, that sort of ignorance is bliss, in a way.

Reference 4 - 0.78% Coverage

But then, others don’t want that question, those questions asked. You know, like if they’ve gone home and said, oh yeah, Mrs Whatever was asking me if I've ever been sexually touched this morning (*laughs*), you’d have a parent on the phone, you know, asking why that was, sort of what that was about.

<Files\\School Workers\\Transcription > - § 2 references coded [0.94% Coverage]

Reference 1 - 0.23% Coverage

Ok. So, my first response is that young people tend to like to be asked these questions.

Reference 2 - 0.72% Coverage

In my experience. So it’s, rather than, you know, adults might find it difficult to answer those questions, particularly British, you know the British culture of, kind of, like, you know, erm, stiff upper lip and all that? It doesn’t seem to apply so much to young people, I think?

<Files\\School Workers\\Transcription> - § 4 references coded [6.73% Coverage]

Reference 1 - 1.58% Coverage

Interesting questions. Not the sort of questions that probably we would ask our students, if we were asking about their wellbeing. We probably wouldn't think to ask about relationship with parents, and I mean there's some there relating to sexual stuff there—we probably wouldn't ask that either. And it's almost made me wonder if we'd be scared to ask those questions. So, it's interesting. Interesting. Yeah. Yes. It's made me think about the kind of questions we ask our students. Just in that short space of doing it.

Reference 2 - 1.36% Coverage

Let's just go back here, like this question here, did someone try to touch you in a sexual way. I mean, I would anticipate potentially, complaints from our parents coming in if we asked our students a question like that. And that could open a potential can of worms for us. And people would probably be worried about asking a question like that because of the perhaps potential comeback from parents. It's quite a probing question to ask students.

Reference 3 - 2.04% Coverage

It's quite a personal question, innit? Why would they object to it? They shouldn't really, should they? Because if they're doing the right thing, then kids are going to have to know, but the facts of life are that some kids aren't going to answer no to that. And I guess there would be a fear of the parents that they're sort of parenting is being questioned. And the quality of their parenting is being questioned. So, I guess that's one, perhaps one concern about why we wouldn't ask that as a school. But I mean if it's an outside organisation then, you know, there's different sort of remits and people know that they can ask questions and it's like well that's fine.

Reference 4 - 1.75% Coverage

My worry would be, how accurate is this? What if it's not accurate? What if it comes out with someone being a high risk and they're not actually? So, might there be a question around accuracy and then, you know, and kids are impressionable people, that's why they learn so much. So, what if they're being given potentially quite a strong impression of who they are as a person, and what if that's not right. It's really, really important that the outcome is perhaps, as I said, you know, young people are impressionable and it is vital that this doesn't do any further damage.

<Files\\School Workers\\Transcription > - § 5 references coded [8.81% Coverage]

Reference 1 - 1.53% Coverage

Oh, it depends. Yeah, it depends. Some, some families really want the help, and they recognise it and are almost so keen and just, "Ah!" there with open arms, saying thank you. Especially maybe if they've

had a less positive experience at a previous school, other families, yes, other families are harder. But, generally, I have to say, once they realise that the agenda isn't against them, that we're not criticising them or their, it is often the parents, you know, their parenting, but we are literally trying to support them and the families have come on board, and they realise that actually this is, you know, supporting their children in the future and I think it's the children's future, so, it takes some of them, it takes a bit longer than other. You know, it's lots of emailing and phone calls, so. Yeah.

Reference 2 - 2.71% Coverage

And I think it's that feeling that, oh, they're blaming, my child's got a label, they're blaming me and it's getting, and it's self-guilt and that, oh, you know, has my child got da, da, da, and as I say, some parents are quite receptive and they've known for ages, since infancy that there is something with their child and they haven't been able to, and they've struggled to get anyone to take it seriously and so they're so grateful. And obviously, other parents very much have tried not to, you know, I've got parents very often who claim that their children are absolutely perfect at home, there's absolutely nothing wrong with them in the home environment, therefore it is the school's fault, therefore it's something we're doing wrong. And yet, we know, we read between the lines, and it's not as straightforward as that. So, it is hard and then they almost resent that feeling of yeah, a label, oh gosh, you know, my child's got, you know, I can't even think, sort of ASD, they don't, and they don't like it. Because obviously, it's so complex, you don't necessarily, labels are a bit of an area of controversy I suppose as well about how useful they are. For some parents they're very useful because they think, now my child will understand why they find reading hard, now my child can see that it's not their fault, whereas for other parents, like, yeah, a feeling of blame, of guilt, have I done something wrong, that kind of thing. So.

Reference 3 - 2.81% Coverage

I think it's a really valuable tool, I think you will get probably people who say, you know, you'll get members of staff maybe or some parents may not want that for their child, I suppose. If the child, the problem is if you want to get parental consent, I guess, you'd have to do that first, and you will get parents who may think, well, no, I don't, you know, what do you mean, why do we need to identify something that's not even there. So, people who perceive you as, whilst we know why we're doing it, there will be obviously people who say, well, you know, you're trying to make a problem where there isn't one yet. And I guess a negative consequence could be if a child feels a bit upset about some of the questions, or feels a bit concerned or, you know, or it brings up feelings and things that they've perhaps buried, you know, they've perhaps forgotten that they've seen a physical fight or that actually, mum had whacked them really hard and you know, something really nasty happened when they were 4 or 5 and I suppose it could bring up feelings and you'd perhaps have to be conscious that they might need some follow up support. Yeah, afterwards, or, as I say, it could be, you know, you get a negative response from parents. Even, I'll be honest here, probably members of staff, who might think well, you know, let's just not add to the already ever growing number of diagnoses and problems etc that we're dealing with. So, that's sort of what I can see off the top of my head, really.

Reference 4 - 0.85% Coverage

Yeah. I think it is. I think there is a lot of feeling at the moment, and this is in the special needs world, it's in the teaching staff, is that feeling of let's not make them, yeah, feel more concerned about themselves. Let's not make them feel less worthwhile, or there's that child thinking "Oh my god, is there something wrong with *me* that nobody else has to worry about?" And I think staff often are quite protective of our more vulnerable children

Reference 5 - 0.91% Coverage

But that, I think there would be people who would say, for goodness sake, let's not worry about it, whereas actually, we know it's crucial to start, it's crucial to know beforehand. But obviously then, what would you label them? You can't then give them a label, you can't say, pre-depression person, I don't know, what would be the label? What, how would they, you'd have someone going how are they going to use that knowledge as a preventative measure, I suppose. Rather than later on.

<Files\\School Workers\\Transcription > - § 7 references coded [8.13% Coverage]

Reference 1 - 2.64% Coverage

I think it's in the media a lot. We're back to media, about the social media. It is a lot in there. I'm on Facebook, I'm happy to be on Facebook, I find it a useful tool, there's so many people who do the, you know, pass this on, people suffer from anxiety, they are suffering and nobody will pass this on now and all this, and you're like, no I'm not because I think it's gone round and round so many times that, hang on a minute, you know? I don't know, it's a buzz thing, it's a trend, it's a bit like, and I might come across as a really nasty person, but my belief in these charity events—it's a fad. You know? If you're not doing it, there's something wrong with you, and it's like, no I'm not doing it because I prefer to put my tenner in the bank and give it to charity separately than having to run and show, "Look at me! Look at me!" so, I think it is, again, it's down to social media. It's about the flag waving. It's about people wanting to show, oh I'm depressed, or I'm really aware of depression, and anxiety and oh, isn't it terrible, I couldn't tell you exactly what it is, but there's something. It's a buzz word, isn't it? It's a bit like climate change at the moment, or Brexit. It's the—Ooo depression, and it's ok, it's good that we're all talking about it and yeah, let's chat about it and be more happy to do it. But I think there's a lot of people out there who think they're depressed who probably aren't, if that makes sense.

Reference 2 - 0.46% Coverage

Yeah. It's a trendy thing. And that's what I worry about with kids cutting themselves, I think there's been a period, I think we're out of it now, possibly, but there has been a period a good few years ago where I think it was trendy for kids to do that.

Reference 3 - 0.65% Coverage

Because kids, you know, I would think you should perhaps if you're cutting yourself, you're not going to show up about it. But we had kids wearing short skirts on purpose on a trip to show all their scratches. And to me, that seemed against the principle. But that might be me and my ill-understanding of it, I don't know.

Reference 4 - 1.27% Coverage

I mean some of them will think, oh yeah, yeah, I'm depressed, let's have a look at it, and then they'll fill it in, you know? And it depends on their moods at the time, I mean, the relationship with mothers and fathers, that changes everyday for a teenager! You know? I can talk to them, and it's like, no it's bad, we've fallen out, yeah, it's regular, just you know, yeah. It's tricky. I don't know. I don't know. I think it might be a good tool maybe for a doctor, in that respect. If you're ticking it like that, so you're sat there with your doctor and have a chat about it and went through it. But obviously, you know, the teenagers in with the doctor, normally go with their mum or their dad, so yeah. Hmm.

Reference 5 - 1.40% Coverage

Sometimes there's a danger of these things highlighting things that don't exist, if you know what I mean. You've probably gone down that route yourself when you're working out questionnaires, sometimes, you can get them to say that things are happening that aren't. I don't know if that's in it, with again, maybe the kids not answering it fair or straight. So, it would lead to, you know, and that I suppose, if it leads to just a chat with somebody on child line, there's no harm in that anyway. Yeah. If it's all leading to good, it's about talking at the end of the day, and you know, being able to understand your feelings and realise there's nothing wrong with it, like said, being sad or being exceptionally, you know. And whether it's leading to depression is another matter.

Reference 6 - 0.69% Coverage

Yeah, I do. I just think that some parents and teachers would have issues with some of the questions because I think, when you're dealing with anything that's—child protection at school, we're told not to ask any leading questions. And obviously all these questions on here are really leading questions, aren't they? With a yes, no answer. So that's where the boundary happens, really.

Reference 7 - 1.02% Coverage

It might help a few. It might help a few, and obviously helping one or two is better than none at all. I think it's impossible to think that you're going to help everybody. Because everybody's individual, and is different, so you'd have a different way of doing it. Some, like I say, even if they were, you know, were suffering from depression, they might just fill this in and be silly about it, just because they don't want to own it. They don't want to acknowledge it. They don't want to, yeah. Some of them might even like it, if that sounds crass, but you know. Mmm.

<Files\\Social Workers\\Policy Makers\\Transcription > - § 2 references coded [3.17% Coverage]

Reference 1 - 0.45% Coverage

The only thing that I would say is that, you know the question where you’re talking about kind of touched somebody against their will? It’s a bit of a kind of a contentious question the way you're asking it, I thought.

Reference 2 - 2.72% Coverage

Because, young people now talk about consent, don’t they? And I don’t know if they know what against their will means. The terminology is now much more around consent. And then, I was thinking about, often, it’s actually preteens that are abused, aren’t they? And they are often compliant in that because they don’t know that they can't be, and they don’t have the power to do so. So, I'm just thinking about the question around, because if you asked somebody against their will, or with their consent, that might kind of skew it a little bit, for those who have been abused in that way. We know that statistically, that’s a huge population of kids that we don’t get to tap into, there’s a lot more people, I mean the children’s commissioner has done that huge piece of research a little while ago, where she talked about, kind of statistically speaking, there’s one child in every classroom that’s currently being sexually abused. Which is horrific when you think about it. But, if you think about that as our population in the UK, that’s about 1 in 30, that are currently being abused. So, yeah, if you think about that statistic, let’s say you were doing this as a universal, there’s going to be 1 in 30 kids who you’re asking that question to that if you ask it in a different way, they might say yes or no depending on how you’re asking it.

<Files\\Social Workers\\Policy Makers\\Transcription > - § 6 references coded [19.78% Coverage]

Reference 1 - 5.39% Coverage

must admit, my initial response was a bit sceptical in terms of acceptability. I suppose it seems, I suppose I, ugh, maybe I've got a bit of a generalised, again, this isn't about using a kind of medical screening tool for something which is I suppose I would regard as more of a social problem. So, not that I, I wouldn’t at all deny that depression is an illness. And that some people have it worse than others and so on. And it is possible to identify people who have really serious illness of depression, and they would need treatment and those treatments would include some quality, you know, I'm not sort of, in that sense I'm not, I'm not anti-medical, but this seems to me like a screening for possible diagnosis or risk of diagnosis which seems to me potentially misplaced. I say social problem in a sense that, I mean pretty much all of the factors that you mentioned there are to do with divorce and relationships and social in location aren’t they, really. Things like are you an ethnic minority, have you come from an adverse family situation, so those are social circumstances, you know coming from an ethnic minority itself is not a thing, but we know that in lots of societies ethnic majorities tend to marginalise minorities and you get experiences of racism and there’s all of these kinds of social or even political issues and I suppose I'm a little, yeah, so I'm a bit sceptical about identifying risks of experiencing those issues as a route into treatment.

Reference 2 - 2.73% Coverage

I suppose I'd be concerned that if the emphasis is on identifying kids who may not think they've got a problem, maybe that's not the way to go. And also, I suppose risk screening does feel a bit deterministic, doesn't it, I mean, what social scientists would call the ecological fallacy of, because these things are associated at a population level with risk that they necessarily then affect an individual, you know, some of the individuals who may score very highly on the tool may be nonetheless actually be quite resilient individuals, you know, they do have difficult circumstances and they do fall into some of those population categories, combinations into sections and categories and a potential risk, but actually they don't find, you know

Reference 3 - 2.35% Coverage

But the fact that his behaviour is causing problems to others probably has come to the attention of teachers and others, and often, you know, schools are putting in quite a lot of support for such kids and acknowledging that they do have pretty tough lives, we're seeing that they usually do. So, even without a screening tool, there is a sense of risk, some other people involved in young people's lives would be identifying problems anyway, and already are anyway. But, no, I think as I'm taking about it, I'm thinking maybe there is a space for this screening tool, but yeah, it wouldn't necessarily be my starting point or my main emphasis.

Reference 4 - 2.34% Coverage

Yeah. I think, actually there's an awful lot of suspicion in the social care field about screening and risk identification of anything. So, you know, in the field of child welfare, children's social care, children in need, child protection, those kinds of issues, there's quite a resistance to quantitative risk scoring. Quite a resistance partly because of the whole, you'll get this in other professional fields too, about it undermining the autonomy and discretion of the practitioner. You know, I don't want to be told by a score what the risk is, I want to make my own mind up and I know best having met a family, what the risk really is.

Reference 5 - 2.78% Coverage

Partly that, and partly also a lot of concern about false positives because what you have in social care is you've already got excessive number of referrals. So, you've already got far too many families being kind of on the radar of social services. And anything which is going to result in even more being put forward, would probably be, would generally be thought to be unhelpful, or I suppose that's about feasibility probably as much as about acceptability, that, you know, we want fewer families to be identified, not more, really. And there would be a fear about false positives, I'm not talking about the depression, young people's depression risk tool here, I'm talking more generally, I'm talking much more broadly about the use of risk tools in general.

Reference 6 - 4.18% Coverage

Yeah. I think there would be, now this may not be born out, but there would be a fear that it was just going to identify lots more kids that are at risk. Yeah. And, so, there's quite a lively debate about the emphasis on adverse childhood experiences in current policy. For example, some people think that's a generally helpful view emphasis in that, you know, it allows you to identify kids who might need a bit more help a bit more early on, other people feel it's just net widening, you know, and it's just going to bring lots more families into the child protection system where actually they're just going to be policed and not given much help. So, what your stance is on that I suppose partly depends on your view of the help. Now, it's different in terms of young people's mental health because you are talking there about trying to help people, rather than going into people's families and taking their kids off them, you know. But, yes, over, a fear of over-intervention I suppose would be there, would be around in social work. But, I'm talking more generally about children's services now, and not specifically about child mental health.

<Files\\Social Workers\\Policy Makers\\Transcription > - § 2 references coded [11.97% Coverage]

Reference 1 - 7.10% Coverage

I don't see why not at all. It's not asking any extremely kind of, it's not asking for details. It's not asking for the kind of information that might put children off, or that they would need somebody to be with them to complete it. Do you see what I mean? So, it's the sort of thing that I could quite easily see being used in schools. I've written papers which have used, you know, asked questions that are more detailed than this one is looking at. So, I think it has really quite a lot of potential. Within social care, the experiences are, that I've had over, kind of recently, have been to do with children who've been experiencing trauma of some kind. And often to do with sexual abuse or kind of violence in the family, that kind of thing, and the issue there actually tends to be more the concern of practitioners in asking these questions, or in giving the kind of inventories for them to complete. Because they've been sort of worried about the children getting upset about it. But, to be honest about it, I think that more of this is in the minds of the practitioner than in the young person themselves.

Reference 2 - 4.87% Coverage

Well, yes. Because as I say, I think it's primarily motivated out of people being, thinking, worrying that the children are being upset. You mustn't ask them if they've experienced sexual abuse because confronting that issue may up-head them. That's the kind of thinking that seems to be behind it. I mean, having said all this, you see, the [National Children's Charity] have done telephone surveys, or you know, engaged organisations opinion polling type organisations to go and contact tonnes of children in order to find out about the extent of child abuse, including child sexual abuse and so on in this country. And, you know, they get thousands of responses to this, so it's, you know, these things aren't impossible. People make too much of them in my view.

<Files\\Social Workers\\Transcription > - § 2 references coded [2.78% Coverage]

Reference 1 - 0.81% Coverage

Yeah. It can, it's exploring why and what makes them feel that their child doesn't or that they don't need that support. And often it's a misunderstanding or a misbelief, or a feeling of blame, or that we're blaming them for what happened and why their child is like that, and I guess it's being alongside them and I think acknowledging as well that yeah, they're feeling that at the moment really helps them, and like yeah, ok, alright, and it takes time sometimes.

Reference 2 - 1.97% Coverage

And, I guess with families, I wonder if it's information, it's lack of information, lack of understanding, not knowing what different services do, and I find that, for example, families that I work with, if there's a family support worker in a school, brilliant, they'll work with them. If there's a family support worker in social services mmm-mmm (*shakes heads to indicate no*) and they do the same thing essentially! But it's buying, you know, into that, ok this is like school, this is normal, this is, I'll go and they'll help me out, come in to social services, or come into CAMHS, Oh God, this isn't where all families go, and this isn't my normal environment, and, yeah, and I think, yeah, that's important in terms of reaching out. I do think context comes into, in terms of being meaningful, comes into it an awful lot. You know, a kid will always meet me in McDonalds, but they wont meet me in an office, and it's, services have got to fit in with young people's lives and families lives. Because they, young people, especially when there's no support, they're not going to come to us. So, yeah, I think that's, yeah.

<Files\\Social Workers\\Transcription > - § 1 reference coded [2.02% Coverage]

Reference 1 - 2.02% Coverage

School, maybe. Or, I don't know how many medicals they've—I don't know that they have any medical interventions now—or hospitals I guess, as well. Like, if somebody comes in to A&E, I think having experience of an A&E situation that went disastrously wrong, because everybody just believed what the young person said. But actually, they were really coerced, and so their version of something that had happened was what they'd been told to say. And, it didn't match the setting or the actual injury, but it was like, well, they're an older child, so they must be telling the truth. And I think that's a difficult one for adolescents as well, because when they're hurt in someway, that, all the emotional and psychological stuff is coming into it as well. And so they were very much pushed into a particular set of responses.

<Files\\Social Workers\\Transcription > - § 1 reference coded [1.18% Coverage]

Reference 1 - 1.18% Coverage

I think potentially. I mean, my experience is that some teachers are quite nervous of having these conversations. Even when you kind of do the sort of general, like we do, like mental health awareness sessions with teachers and stuff, even if you go through the general sort of how to talk with a child when they're having this type of conversation with you, I think they generally get the theory of it, but they still

wouldn't particularly be comfortable doing it. That's why they've chosen to teach rather than do what I do (*laughs*), do you know what I mean? But I don't know. I think so. I think particularly teachers

<Files\\Social Workers\\Transcription > - § 5 references coded [11.13% Coverage]

Reference 1 - 3.14% Coverage

Well, it depends what your predictors are. I suppose, at the end of the day, if these are the areas that are predicting it, they're the areas that you have to cover, aren't they? So, about the relationship with the parents, how their relationship would have been, you know, being separated from your family and being attachment disordered, that sort of area, that comes under domestic abuse, I suppose if you're covering it within adults fighting in the house. I think that—I mean, I don't know quite if I like the wording that you've got there. Did someone in your family or household ever beat you? I think that's a bit evocative, a bit emotive language, you know? Maybe just that you could say, you know, did you ever receive injuries from a parent that required medical care. I think, you know, "beat you" would be a bit of a strong word. Yeah, in a way, again a bit with the "dirty clothes or torn clothes", that's a little bit, because a lot of the kids that we work with may have torn clothes, as I said, or dirty clothes, but it doesn't necessarily mean that they're being abused. Or it doesn't necessarily mean that they're, you know, it might just be that actually parents can't afford to wash their clothes all the time, you know. And in this day and age, water costs a lot, if you've got families that have got lots of children, I mean, I work with families of 6 kids, and they haven't always cleaner clothes, they are torn clothes because they get handed down to the next child. Again, I think that one, "Have your parents ever felt they wished you were never born?", is a bit subjective. I think most kids would probably say at that point, yeah, I think my parents wished I was never born. But how do you really know that? Do you know what I mean? I mean with my own kids, I know that they're going to go "Oh, yeah, bet my mum never wished I was born", but it's like, it's not true is it? So, I think that's a bit of a subjective question.

Reference 2 - 0.56% Coverage

They're all going to be very different, I would've thought. I would have thought that the issues in each place, the context is going to matter. Is that why then these are very basic questions, to cover all areas, yeah. Because I would have thought in Nigeria, things like social media maybe quite a bit different, or Nepal, necessarily than England.

Reference 3 - 1.59% Coverage

Yeah, I think probably. I just don't, I'm not quite getting why, I mean I understand that you're trying to develop a tool that will then be able to identify depression in all young people, but I think there's too many factors within it. I think there's far too many factors that it's not going to be a catch all—or this isn't going to be something that you can, as I say, go into a school and actually give out to all these children, and calculate and say, right, ok, you 25% over here are going to get depression, the other 75% aren't. Because I just feel that there's going to be so many different layers of factors. As you said, like gender, I mean, you know, it's higher for males, isn't it? It's higher depression. I don't know if, see I think it's about using it afterwards to say, right, ok, so it's already started, or this is what your risk is now that

this has happened. Because what's going to be the outcome of the tool? What are you going to be developing as an intervention?

Reference 4 - 3.59% Coverage

Yeah, yeah, I think definitely for these sorts of questions. So where was it, that one, that's quite an obvious one, isn't it, if somebody's tried to touch you in a sexual way. But then again, actually, that could be, again, see no it could be. Because, there's very, so "did someone ever try to touch you in a sexual way", yes, was this person over 18, no it was like, do you know what I mean? It might be normal, again, teenage antics, round the back of the bike sheds at school, somebody—do you know what I mean? So, you've just said there literally, yeah, about, so it could just be something that actually was very, very—well was it an adult, or was it something that was properly sexually inappropriate? Have you ever drank alcohol? Yes. How many units? Or how many cigarettes do you smoke on a daily basis? How often do you use marijuana? What other drugs? Because I just think, as I say, I think, over 14 you're going to be finding lots of kids who've experimented in all of these sorts of ways, but it will be very normalised, normal behaviour. Yeah. I mean that one's again, quite, when you think about it, have you ever seen adults in your home fighting with each other, or fighting with children? What does that word mean? Are we talking about full on fist fights? Or are we talking about shouting at each other? Because it may be that actually the kids are shouting at the parents, and the parents are shouting back at the kids, but it's not necessarily—you know, it's again, have you done your homework, and then kids being kids, they think fighting, that's it, it's argument, isn't it? So what are you, which type of fighting are you trying to draw out there, you know. Is it yes, then was it just normal, that normal sort of family rows? (*laughs*) Yeah. I do think it's quite yes or no, isn't it? They're all quite black or white, and now actually, looking back over them, you sort of start to, some of them do feel a little bit. I mean, how does this one work, these two, so within causing, or being an indicator of depression? (*indicates the questions "Have you ever thought or felt that your parents or caregivers wished you were never born?" and "Have you ever thought or felt that someone in your family hated you?"*)

Reference 5 - 2.25% Coverage

That's really, really subjective and kids of 14, so that sort of age may feel that their parents, you know, but actually their parents are doing the best job they can do and they're trying everything, but actually being a kid who generally just thinks everybody hates you, "Oh my god, the world hates me, yes of course my parents think they wished I was never born". I think you maybe need to—maybe that one needs to be, "Have your parents ever *told* you…" because actually again, subjectively, thinking about it, the majority of kids will think that. Especially if the parents are trying to do the best for them, in some senses, you know? You know, make sure that you do this, make sure you go to school on time, da, da, da, da, "Oh god, you just wish I had never been born, why am I in your life", you know, that sort of thing. I think it's sort of, and again, the same with that.

<Files\\Social Workers\\Transcription > - § 1 reference coded [1.34% Coverage]

Reference 1 - 1.34% Coverage

I don’t think anyone is. I don’t think you can really, I think, I don’t think that you can really, I think, I don’t think that you can actually guess these things. I think that, I mean there are some indicators, I guess. But those are really, and working with children’s services, working with children in care for example, are very likely because they’re not with their care givers. People who are very much isolated from society, are at risk. But I think that it’s, so for example, children in care system, are most likely children who are in gangs, probably. But they’re the other extreme. And other than that, you just can’t tell, you know. And even, all the children I have working with, I have something like 4 children working with me in the care system, I cannot say that any of them are going to develop depression, you know? It’s just not that easy to tell, yet. I think once they leave their foster care placements, things like that, they might develop depression, simply due to their circumstances and isolation, not just their history. They just don’t have anything. So, that's the primary problem for them that will make them feel so isolated and lonely.

<Files\\Social Workers\\Transcription > - § 1 reference coded [2.37% Coverage]

Reference 1 - 2.37% Coverage

I guess that it, yeah, whether it considers all the different areas and in greater detail for a young person. Erm. So, I think that it’s great at kind of highlighting those that might be, or it could potentially be good for highlighting, yes, at risk, low risk, but I think we can't take that as face value. And actually, sometimes, you know, there might be a young person that only ticks one box, but actually it’s to a significant extent and therefore would have a significant impact on, you know, their likelihood of, of developing depression. Erm. So, I, yeah. I would be cautious of kind of just taking it at face value, and therefore ignoring, you know, those that might be considered, through a scale or a tick box exercise, that might be considered low risk but actually there’s something going on in their lives that doesn’t fit into these boxes very neatly! Erm, that would be my concern. Erm. Yeah. If it picks everything up, and accurately detects, you know.

<Files\\Social Workers\\Transcription> - § 1 reference coded [1.64% Coverage]

Reference 1 - 1.64% Coverage

It’s not the kind of—I would have thought, with some of these questions, the answers to that would already be known. Like, from a social worker should already know how the relationship, unless it’s kind of like some, the first time they’ve met the child, that they would actually know the answers to these. You know, have they been separated, or been cared for, has there been fighting in the home, some of these things they would already know.

<Files\\Social Workers\\ > - § 7 references coded [3.14% Coverage]

Reference 1 - 0.58% Coverage

And I suppose linking in with what you're doing, it kind of—you want to do something that's got an evidence base and that is helpful and informed by research, but—what am I trying to say? Kind of, it's very difficult, going back to what I was saying earlier about locating the problem in the individual. It's really, I don't know, can't quite get my head around how you'd frame it. Where you don't necessarily want to identify the young person as the problem. That's what I'm grappling with. I don't know how you'd manage that in an app! (laughs) Maybe it would be the difficulty (laughs).

Reference 2 - 0.37% Coverage

I'm just wondering if you were looking at a psychosocial-bio model, which fits in with kind of social work type work, if you felt that the risk of depression was mainly caused by the environmental factors like those, would you be looking to challenge those environmental factors? Would that be enough? You know, to mitigate the risk of developing depression. I don't know.

Reference 3 - 1.19% Coverage

Oh, again, I think unless churches and religious groups – I think a lot of our families have strong religious beliefs and so they really listen to their pastors or imams. So, I guess it's raising awareness through those groups. I think that's potentially more tricky with religious organisations because they have, might have, their own ideas about what emotional well-being is, possibly connected to religious or spiritual ideas. And just thinking that, I was just trying to get them to think about how when parents lose their temper, and how to help them selfsoothe. And I really wasn't expecting it and it really got me thinking about how we then pitch some of these ideas, how they fit in with religious—how can we respect religious ideas, but also want to give some evidence-based education (laughs). So, it's tricky! I'm trying to think about community groups.

Reference 4 - 0.35% Coverage

Yes! Language is hugely important, isn't it? I think that thing about social constructionism, so that says language constructs reality. So, I think coming back to what you were saying earlier, the word "depression" constructs a reality, you know? Some cultures don't have the word depression—other languages. Back in time, was it called melancholy?

Reference 5 - 0.11% Coverage

Yeah. So, the word "depression" constructs a reality about what it is, and it locates it in the individual. So…

Reference 6 - 0.41% Coverage

I'd hope so! On the radio yesterday they were talking about—they had a couple of headteachers phoning saying that one of the barriers to teaching lately was kids going home telling their parents they had a detention, and the parents storming down to the school and challenging the teachers! (laughs) So, I'd like to think that parents would be alright about that, but I expect that some parents wouldn't want that.

Reference 7 - 0.13% Coverage

So, it's getting that message out to parents about why this is of benefit to their families, and not the nanny state interfering.

<Files\\Social Workers\\ > - § 3 references coded [0.78% Coverage]

Reference 1 - 0.38% Coverage

So, it's—I think some of the ones that might be a little bit more tricky for them to answer might be, you know, how's your relationship with mum—your mum is a bit more kind of like, I don't know, you've only got a couple of options and it's a bit like, well, I hate her today, but I liked her yesterday. So that's a bit more difficult to get quite right, isn't it? But I don't know how you get around that.

Reference 2 - 0.20% Coverage

Only you're reliant on the information that you're given, aren't you? So, some people who are, you know, anxious, are going to sort of put in what they need to get that high-risk thing to try and get the service.

Reference 3 - 0.20% Coverage

And then you're obviously going to get others where you're not going to get the right data because they don't want you to know there's something going on, so you're always going to get the anomalies aren't you, so.

<Files\\Social Workers\\ > - § 10 references coded [5.82% Coverage]

Reference 1 - 0.61% Coverage

Mmm. I guess it would be around their understanding of what depression is as well isn't it? If they do a questionnaire like that, and get a response of, "You're at high-risk of depression", what is their understanding of that? Is that going to completely freak them out? "If I'm going to end up with depression, what is depression"—and I think that needs to be part of the consideration before they undertake the screening tool.

Reference 2 - 0.54% Coverage

And what their experience is or not of depression. If they've got a parent that suffers with depression, or a family member, where they've seen it first-hand, it could be positive in that they've
seen that they've received support. Or it could be that, you know, deep depressions, or suicides in the family could have an impact thinking, "That could be me". It's a lot to consider.

Reference 3 - 0.42% Coverage

For me, I thought it was quite clear. I mean we tended to just put good for each of them didn't we, but; very good, good, regular, bad. It's the same with any kind of scale, questionnaire, where they have a limited number of options isn't it? It depends how they define it; regular, bad, good.

Reference 4 - 0.38% Coverage

I think the length of the questions is good, pretty clear. (reading the risk calculator again) "Have you run away?" They're quite yes, no answers I think, most of them. I guess they could, yeah, there could be another option for other or sometimes, or potentially.

Reference 5 - 0.56% Coverage

I think managing, as I said, managing the outcome of it—the responses—I think needs a lot of thought. I can imagine that you could get a lot of young people, you know, if they're at high-risk or if they then think or tell people, "Oh, I've got depression". So, it needs a lot, you know, a bit of a health warning about what this actually is, that it is a tool and it's not a diagnosis. Yeah.

Reference 6 - 0.25% Coverage

And obviously, it depends on the answers that they give. If they're torn between a couple and they give one way or the other, that could determine the whole outcome of the result.

Reference 7 - 0.84% Coverage

I just think if parents have got a professional to discuss with, it might be more reassuring for the child or the parent, but all families are different, aren't they? They may have had their own experience and they want to highlight to see if any support is required, they might be quite capable of being proactive and seeking out themselves. I guess I'm thinking in my professional role that families and the children that I work with, you know, you do get that, but you get more of families where they're in distress, or they're not managing on their own, so they're needing extra support.

Reference 8 - 0.59% Coverage

You know, if depression is influenced by experience and life events, that could potentially, if they were looking like they could develop depression, there are a lot of things you could change. Yeah, I think a lot of parents wouldn't want their child—they would maybe see this, you know, risk tool as saying that they will have something, and they don't want to think about that, or don't agree that that is the case.

Reference 9 - 0.55% Coverage

I think if—personally, I think if people have had their own experiences, or know of someone who has had depression, they're going to have a much more—a much deeper understanding. But it's not always the same for everybody—I think some people still have the view of, you know, people feeling sorry for themselves, or just get on with it, or that kind of attitude, which isn't always helpful.

Reference 10 - 1.07% Coverage

"How's your relationship with your mother? Your father? What's their relationship like?" I think, although some of the, you know—those discussions are had between social worker and child—I think if this is a screening tool, I do think parents, a lot of parents would feel like this is kind of a
blame tool. If it comes out as high risk, are you saying it's my fault, kind of thing. I do think a lot of parents wouldn't want to go there. "Parents fighting." You know, if social care, local authority social workers are involved, it's likely that a lot of these— some of these—will be the situation for the children;

running away, difficulties at school. So, it obviously depends on the individual, some would welcome support, but I do think many may not.

<Files\\Healthcare Workers\\Policy Makers\\Transcription > - § 5 references coded [8.47% Coverage]

Reference 1 - 3.65% Coverage

I think something that we're often quite wary about at the moment, in terms of what can feasibly be done, in terms of treating and managing mental health problems is, it's almost like, it's that ethical quandary and balance between we know that these conditions effect a huge number of people, and it's really important to identify them, but we're also aware that there's so many people that at the moment are not identified as necessarily being at risk, or even having developed these conditions. And, I think one of the things I worry about slightly is that unless we have enough capacity within our mental health services to actually be able to properly capture and really assist these people, that's the sort of risk that, like the alarm bell that immediately goes off in my mind. But that also absolutely doesn't mean that we shouldn't be trying to help the people that have the problem. So, I think, it just opens up an additional question around resourcing and making sure that if we are capturing potentially huge numbers of people that might be identified as high-risk individuals, how do we ensure that we then have something in place to ensure that we can actually help them in a meaningful way? And not, potentially, highlight to them that they might have a problem that they then can't actually seek help for. Which I think is like, the first thing that sort of springs to mind. I mean, I really like the idea of it, in principle? Erm, and obviously arming people with the, erm, language, can be really important sometimes. And even just recognising that there are places that you can go, and you, these things that you might not necessarily, that might be normal, in terms of your life experience up to then, can be something that you might want to seek help for. So, I like the idea in principle, but I think it opens up, sort of, ethical and resource related questions for me, would be my sort of first instinct on it. Yeah.

Reference 2 - 1.63% Coverage

I guess, thinking about a young person, who potentially might not have ever spoken to anybody, potentially, about some of the things that they're then answering questions about, also, just makes you think, you know, there's a potential, you know, safeguarding issue. Erm, in terms of, you know, if someone's been sexually abused, is still being sexually abused, has been beaten by a parent, or another person in their life, erm, there's sort of legal and safeguarding issues involved as well. And, sort of, I don't know, ethically what position that puts, erm, whoever's running this project in? Er, and sort of signposting, or, making sure that services are available to capture people that might be in a vulnerable position. It, sort of, just, sort of, flags potentially really negative, harmful, dangerous things that might still be impacting on people's lives.

Reference 3 - 1.76% Coverage

So, I think maybe the biggest challenges are, potentially highlighting a lot of- So I think, potentially a lot of people are going to be highlighted as being at high-risk of depression, and equally we know that environmental factors are incredibly important, but equally they are only part of the story. Erm, and I think these mainly get at those. Erm, so again, as I sort of mentioned before, this sort of potentially highlighting even more people that are needing potentially to go through vastly overstretched services, and, I think there's some evidence to suggest that actually, being on a waiting list can actually

sometimes cause more harm in terms of depressive symptoms, than, erm, sort of just being given a placebo. Because you're sort of driving up that expectation, that actually you do need help, it's just that right now we don't have the help to be able to give you. Erm, so I think that's potentially a big problem.

Reference 4 - 1.09% Coverage

I mean, I guess what springs to mind is that we know there's a big sort of biological and genetic component, as well, erm, and I guess which is some, to some extent, got at, potentially, with the relationship type questions, but, I'm not sure that I'd actually want to see it go in that direction? Because it's, I don't think that's what this tool is really about? It's sort of identifying people that might be environmentally at risk, erm, and actually, the genetic route I think is way too early to sort of, sort of, be thinking about that sort of thing, or like signposting.

Reference 5 - 0.35% Coverage

Yeah. I think. Sort of touching on what I said before, I think broadly, yes. My main concern is that not necessarily being able to help immediately, people that might be at high risk.

<Files\\Healthcare Workers\\Policy Makers\\Transcription > - § 7 references coded [10.69% Coverage]

Reference 1 - 3.21% Coverage

Well, it's not research that I've done directly. But, immediately, you get to the data capture issues, and how data is stored and people's rights to protect their personal data and how you link data about people's mental health status with their physical health records, you know, their physical health records, and actually their actual physical health record inside the NHS if that makes sense. So, we've done quite a lot of work, and it's not us, it's the work of our [research centre] in [area of the UK], looking at how you integrate patient reported measures with their NHS record. And there's a study called [name] which is the first in the UK to allow patient reported data to be integrated with your health records, and that's for people with [physical health condition], so those people can self report their health status and it can be taken into account by a consultant rheumatologist. We think that's a world first, actually. Or a UK first. But obviously, for people, you know, people, I guess if you're diabetic and everyday you do your own blood sugar readings, it would be brilliant for that data to be able to be seen by your GP, by your consultant. But we don't allow patient reported data to be integrated very often in this country, and data around people's mental health status should be, you know, you should be able to do that.

Reference 2 - 2.00% Coverage

No, I just think people are hesitant about talking. I think people are hesitant about, I mean, I don't know, I haven't ever worked with children in a, you know, I'm not a social care worker, or a member of the police, and others will probably give you better feedback. But, I would suspect that if you're asking somebody to fill in something that says this experience has happened to me, and identify a member of their family, that's quite a big hurdle to get over, but what if that fed back? What if you've got a legal

duty to intervene? I mean, there's so much child safeguarding and stuff that goes on in the world now, what would you do if somebody answered that question? Or wrote in a verbatim comment box after it, and this is continuing and I'm 17, you know? I guess there's some quite big ethics around asking some of those questions.

Reference 3 - 1.65% Coverage

Yeah. And about your, I mean, there are lots of different scenarios in research where, so [National Charity] did a huge piece around unintended consequences. So, what you do if you are, you might have somebody in a research study that's involved some imaging of their brain or their back, nothing to do with cancer, but as part of the clinical intervention in the study, you find that this person's got a cancer tumour or a brain tumour or something else wrong with them, what's your responsibility to disclose that to them, or not. So, you've got a similar situation here, but what's your protocol if somebody reports this to the study? What's their expectation and how do you respond to that.

Reference 4 - 1.30% Coverage

I don't know. I don't know if. And I guess it's, I guess the question comes back to then what do you do and how do you feed the information back to people. Because if they fill in this score and it say you're at high risk. What do you do? And does it help you to know that you're high risk, if there isn't anything that can help you. If it's not going to stop your parents having a bad relationship and one of them being abusive, how does it help? Is it like, is it an equivalent of the genetic test for the thing that you can't then get rid of?

Reference 5 - 1.74% Coverage

Well, that's dependent on putting an intervention into a system that has the support available for people, isn't it? And we know it doesn't. We know that adolescents we've identified with mental health problem are sat on waiting lists for ages for psychological treatment and support. But I'm not trying to sound like I don't think we should help people think about their mental health, because I absolutely think we should. We should use that spate of systems better. But I suppose you need to think about, if you think, you know, you need someone like the [National Council of Ethics] to help you think about that aspect of what do you do when you help people to identify a problem that there isn't then necessarily a solution for.

References 6-7 - 0.79% Coverage

I'm just thinking about, I'm actually just thinking about other areas of health where you help identify things, you know, and the ethics of doing that when you know there might not be a solution. But that's, you know, genetic screening is one, the people who take part in research trials is another, we've talked about both of those.

<Files\\Healthcare Workers\\ > - § 1 reference coded [0.58% Coverage]

Reference 1 - 0.58% Coverage

Yeah. It's very difficult to say. I can see the function of it, and, I suppose the question I'm asking you is: if…if there was, if you raised awareness in schools, made this more part of everyday discussion, does that increase the likelihood that people might seek help in the event they feel they need it? Is this going to increase the likelihood that people will go to see their GP if they're feeling depressed? I don't know. It could do.

<Files\\Healthcare Workers\\ > - § 8 references coded [3.54% Coverage]

Reference 1 - 0.48% Coverage

I think sharing it with young people would be bloody difficult. I mean, I think that—I think the potential for use in the wrong way is huge. So that's why I've said I don't think they should, and it sounds like you're running a kind of Stalinist state if you say that. But I think in the, you know, it's a, it's a bit like genetic test—I mean it's like genetic testing, isn't it? It's a sort of real mixed blessing.

Reference 2 - 0.74% Coverage

Well, it's all that kind of deep mind stuff, isn't it? I mean that's the kind of, oh, you know, you get the adolescents to fill in the stuff and you're—you're then producing profiles on everybody. What? They get sent to the CCG? Hah! Well. I mean, you know, it's not beyond the realms of possibility with the way we're going. I mean it isn't beyond the realms of possibility that there could be you know, this data could be mined via Google—and you'd—by large health authority delivery organisations, so I don't know. I mean, I think the sky's the limit quite honestly, with that one. But…I would hope it doesn't particularly go that way.

Reference 3 - 0.51% Coverage

Which is what Kids Company was sort of trying to intervene on—because of that issue of the child having the experience of somebody recognising their situation, their predicament, is intolerable. Knowing that somebody has seen it for what it is, and yet nothing changes because it's been reported to the local authority, or it hasn't, but there's no response. So then that leaves the child feeling even more bereft and abandoned really.

Reference 4 - 0.55% Coverage

So, that—that's why in a way, there's a big question about well, if, you know, what's the point of getting the screen going for this country when you can't even meet the needs of the people that have significant mental health problems? And where the government's saying, "oh," by whatever it is, "2021 - we'll try and meet the needs of 35% of the population that have needs." I mean, so it's a bit of a bad time to sort of be trying to bring something like this on really.

Reference 5 - 0.31% Coverage

Well, I mean just a whole different kind of level of funding. Because otherwise there's no service that can deal with the kids that are going to be picked up by this. They're just not there. Even in the third sector, they've had their funding cut and cut and cut.

Reference 6 - 0.41% Coverage

As…as for the secret monitoring of the whole society, and feeding that data back to general practise and the local acute trust, well, yes of course it might be quite handy, but we're a long way off that kind of stealth health management I'd have thought. HMOs would love it, wouldn't they? And health maintenance organisations in the States, I'm sure.

Reference 7 - 0.26% Coverage

Yeah. Because I think it will affect people's insurance policies if people knew about it. If you know about it yourself, you'd have to—in theory, you'd have to—reveal it on forms. So. Could be quite compromising, really.

Reference 8 - 0.30% Coverage

I think there are much more straightforward ways of going about it. Which is sort of the ones I've talked about. Which is, you know: what people can see with their eyes open. And where they're trying to get help, but they just can't get it at the moment.

<Files\\Healthcare Workers\\ > - § 6 references coded [5.53% Coverage]

Reference 1 - 0.92% Coverage

putting myself in the shoes of someone who's using the tool, yeah, possibly some quite sensitive questions that I might not be particularly happy to answer.
And, I guess also, wondering what's going to happen with the information I'm giving. But, from a kind of clinical, academic point of view, and my professional perspective, yeah, I guess I was just thinking all these questions like why—why did they chose the particular things they did.

Reference 2 - 0.88% Coverage

But in a school? Maybe. Then again, maybe this is me making it all too academic, but one would have to think about the criteria for screening. Like, do we have an intervention, do we have a referral pathway? There's some signposting here, so maybe this is enough? But I guess the worry with these things is always, you know, what do you do with the folk who are high risk? But then I guess it's being used anonymously, right?

Reference 3 - 1.66% Coverage

And again, you know, is it—it's only so useful in how you act on it. There is no utility in being told you're high-risk for depression unless you do something about it. And again, there is a subtle difference here between, you know—and you've said that it's only if you're experiencing things that are causing you distress (pointing to the high-risk information in prototype)—and I think that's important because, obviously, being told you're high-risk for depression is very different from being told you're depressed, and what follows suggests that they are depressed. So, speak to Childline and check out this website. Well, preventing depression is good, speak to Childline, again, you know they might not need to speak to Childline yet, but I guess they've got information should it become an issue.

Reference 4 - 0.73% Coverage

But a wee bit like coming back to the whole like criteria for screening type stuff, I don't—I do feel like it would be an easier sell if parents felt that support was in place for the child. Whether that be they felt empowered to do that, or the school was providing that. So, I think it would be more acceptable if they felt that support was in place.

Reference 5 - 0.27% Coverage

Yeah, I mean that's your criteria for screening isn't it? There's no point in looking for stuff if you can't do anything about it.

Reference 6 - 1.08% Coverage

Well, there's data protection isn't there? Yeah...Yeah, you're essentially using people's data to gain intelligence about them—so you're using people's data to target them with adverts. People do that all the time! (laughs)
Yeah, there's lots of big philosophical questions around that, isn't there? Should we be doing that? Should we not? Arguably, this is for a much higher goal than advertising (laughs). So, I don't have a definite answer to that. I don't feel like I've had many definitive answers to your questions!

<Files\\Healthcare Workers\\> - § 1 reference coded [0.35% Coverage]

Reference 1 - 0.35% Coverage

It depends. It depends what you're going to use it for. It depends if it's something that is going to be a label because people don't want to be labelled—some people do—some people like to have the label of whatever illness it is that they've got. But it really depends on what happens with that data that you're getting, and whether it's going to be used as a...as a benefit, or as screening, as a prevention, as an early intervention.

<Files\\Healthcare Workers\\> - § 4 references coded [3.43% Coverage]

Reference 1 - 1.31% Coverage

Well, lots of things, like for example, we do prostate cancer screening: if you screen people for prostate cancer, it doesn't change how many people die of prostate cancer. It just changes how many people we investigate, and how many people we give the subsequent complications of investigation to. And then, if we find cancers that would never have caused them any issues, then we go on and treat them even more, and then they've got all the side effects of treating a cancer that was never going to kill them.
So, screening has to be quite carefully thought out. So, prostate cancer screening is a waste of time, but breast cancer and cervical screening are incredibly worthwhile. So, I think just screening— blanket screening—is quite dicey because you might end up with—well you often are going to end up with—a huge amount of workload and then what are you going to do with all these people that have then said that they're high risk?

With—you're going to run into problems with that, I think. Because, as you know, probably—well I assume you do—is that we've got very little in the way of child and adolescent mental health services anyway. So, if they're not psychotic, then they're probably not going to get involved with anybody at the moment.

Reference 2 - 0.33% Coverage

as long as that…as long as that higher risk outcome has something which you can use it for—then…then it's worthwhile I think. As a screening situation, I think you would be left with lots of people who have had a bad day and you've suddenly got thousands and thousands of people—what are you going to do with that info?

Reference 3 - 1.34% Coverage

If it could tie into access to something like that, someway, whether it was in school, outside school, something like that—as long as it could tie into something that could help them. Otherwise it does just blend in—it might just blend into another screening outcome, that's you going "Great! Now I don't know what to do with this person!". Because that's quite often the problem, that is, that's the biggest…that's the biggest hurdle. When I have a 15 or 16-year-old who's depressed, if they go to a good school, like—I can't remember what the kid's school is with the purple blazers, but if they go to a good school—and they've got a school nurse, or they've got a counsellor, fantastic! You just write back to the school. But if they go to any other school, then you're stuck. Because you can't send them to CAMHS.
And if—particularly if they're the real high needs—the highest need ones who've got, who are poorly literate, no support systems, and just in chaos—the real disasters waiting to happen—if they've not already happened, you've just got nothing for them. So, finding out that they're high risk—we can probably tell that anyway, but it gives you something a bit more concrete I suppose. But then, if you've got no resources to deal with them, then what do we use it for?

Reference 4 - 0.46% Coverage

I don't think you'd have much job getting them on board with the screening question, I think their question would be "Well, when are we going to use this?" I think that would be a big hurdle. And then, yeah, like I said, I think their two questions would be; when are we going use this and what are we going to do with the answer? Because unless you've got both of those with satisfactory answers, the whole thing becomes a bit pointless.

<Files\\Healthcare Workers\\ > - § 1 reference coded [0.75% Coverage]

Reference 1 - 0.75% Coverage

God, that's a huge question. (laughs) Well, I don't think it's totally preventable. I mean, I know that might be a little bit controversial, but I think there are times when it's not preventable—where it just needs to be treatable. And I suppose one of the things that I find most difficult is that some of those higher tiered level services are not properly funded, so, that's a massive issue. I mean, they're just—there's a massive weighting list which is just intolerable if you're a parent of a child who's really, really struggling.

<Files\\Parents\\Tracnscription> - § 4 references coded [6.42% Coverage]

Reference 1 - 1.23% Coverage

And, yeah, then I think the only other risk is you've told someone you're high risk, and if they're not equipped to get help, then where does that leave them? You know, it leaves them a little bit out to see knowing that they've got problems, but they don't have either the resources or the means or the, you know, not necessarily the inclination, but the ability to access help in some cases. So, you've given them this package and now they don't know what do with it necessarily. I mean, I think, you know, having the resources there is great. But I don't know if all children will access that.

Reference 2 - 0.86% Coverage

So, yeah, again, it's the feasibility and what do you do with that information once you have it. I guess that's the thing, what would any of these groups do with this information once they have it? What would a school do with this information once they have it? Or actually, no, I guess the school wouldn't really even know, because this would be anonymous, wouldn't it, or this would be private to the individual.

Reference 3 - 1.68% Coverage

Yeah, I'm not sure, I think I was just thinking about, sort of, the high-risk thing again. It's sort of like, is that just telling you something you already know and then if you were already feeling really low, would that not just make you feel worse in some way, sort of having something really sort of slightly impersonal tell you some stuff that you already know about your life, you know? I don't know. I feel like, yeah, it could potentially just have the impact of sort of being like, ok, well, great, now I've got that confirmed, you know, I know my life's a bit crap, but now I know for sure it is, yeah. There was just sort of a hint of like, what would the impact be on varying levels of depression and then would there be, sometimes, some people who would have a harder time with it than others. So, yeah.

Reference 4 - 2.65% Coverage

And so, this is the kind of thing I always think about when I look at measures like this. Is there actually, is there some kids who would actually be made worse by having something like this. Is there any risk that children who maybe don't have support, or don't have resources, would they actually be made worse by knowing this and not having any real way of dealing with it? So, yeah. That's just. So, when you say sort of dealing with it sensitively, I think, once, you know, once you have this information, as a kid, what do you do with it? You know? Some kids are very resourceful and would be like, yeah, definitely, this is cool, this has confirmed things for me, I know I need to call child line, I'm going to get some help for myself, but just the state of being depressed means (*laughs*) I don't know. Are they all help seeking at that point? And if they're not help seeking because they're depressed then what is the benefit of doing that? What is the benefit of doing that for that person? I don't know. Sorry, I feel like that's putting a dampener on it, I still see its value and stuff, I just think, yeah. If you're already in a vulnerable state where you may be less help-seeking, does knowing it encourage that in any way, or does it make you just feel worse, I don't know!

<Files\\Parents\\Transcription > - § 4 references coded [5.26% Coverage]

Reference 1 - 1.58% Coverage

Ok. Let me talk about the low risk, for instance. Low risk, for instance, is "this is showing that you have a low risk of depression", however, you can also talk about for instance, about sadness, and perhaps, or you might be feeling low, or, this doesn't necessarily lead to depression, and it'd probably, I don't know, link them to a site about how to get out of feeling low, if you do feel low, not saying they necessarily will feel low. Just the difference between depression and, you know, feeling sad or low.

References 2-3 - 1.95% Coverage

But even then, I just don't think, if you're high risk of depression, where is this data being used. It's just like, go to Childline. Most children know to go to Childline, it's nothing new, it's always on the, it's everywhere in schools, go and see ChildLine. And speak to a trusted, someone you trust, a school counsellor, or a health professional…It's like, it's like saying, you're at high risk of depression, and go to these sources that they already know about, and then that's it. You've just let someone know that, *children* know, that they're at high risk of depression, and then it's just, and we'll leave you to it now! (*laughs*)

Reference 4 - 1.74% Coverage

So, obviously, I think it's really important for schools to be on board, and perhaps guide them in this direction, but unless they have the resources, then they might as I say, just spot the loud ones, and then miss the other. I mean, I suppose if you spot one person, that's still good, but, then what? (*laughs*) It still goes back to then what? You've found out that you're at, you know what I mean, that you might be at high risk of depression, but then, what? What do you do? (*laughs*) But it just depends how the calculator is worked out. Yeah. That's how I feel.

<Files\\Parents\\Transcription > - § 3 references coded [3.42% Coverage]

Reference 1 - 2.36% Coverage

What else would I like to see? I'd like to see, and this sounds really churlish (*laughs*). Well, who do you talk to? You have to have a safe environment to talk to somebody. And I feel all these things that say it's good to talk, find someone to talk to, open up to someone. Well, who? So, you then go to the National Health GP or whatever, and you get a list of things and then your nearest drop in centre is 7 or 8 miles away and you need 3 buses and 2 taxis to get there and then it's closed in the evening. So, who do you go and talk to? Or, you go on a waiting list, you have to be evaluated by a psychiatrist before you can be referred to one of these places and then it's at a peculiar hour and you've got to be in a group of other people as well.

Reference 2 - 0.47% Coverage

Yeah. Yeah. I think so. And you need to know something's going to be done about it, don't you? If I tell this person this about my private life, it's a big risk, potentially.

Reference 3 - 0.59% Coverage

Well, I'm finding it a bit mind-boggling, really. I just think it could go all over the place, really. So, unless there were really strong interventions to protect, make sure the child was very protected and believed and…

<Files\\Parents\\Transcription > - § 1 reference coded [0.58% Coverage]

Reference 1 - 0.58% Coverage

Yeah. Yeah. But then still, even if you're aware of it, and it's, you still need somewhere to go with it, don't you? And if there's nowhere to go with it, then it doesn't help much that you've got more awareness.

<Files\\Parents\\Transcription > - § 1 reference coded [0.83% Coverage]

Reference 1 - 0.83% Coverage

well I can see a few. Erm. You know, obviously one of this is, is that if someone does access this screening without that sort of support, or any clear follow up, like I said, that could cause them some, you know, some distress? That might make the situation a bit worse without really giving them a clear outlet for it?

<Files\\Parents\\Transcrirption > - § 2 references coded [2.18% Coverage]

Reference 1 - 0.89% Coverage

No. Just thinking that it is challenging (*laughs*). Because, at first, when you showed it to me, I thought, oh, like, yeah, it's not very complicated to answer. But then, what do you do, what is the next step, like, when you have the, ok you are in high risk, what do you do with that information.

Reference 2 - 1.29% Coverage

I don't know. Like, I think, I'm not sure if it's a policy, also we talked about it, about education. But to learn more about the symptoms, so if you, because maybe parents also need to see that, you know what I mean? And then, yeah, but I'm thinking, again, I think that as a screening tool it is good. But then, what you do, what will you do with the results. I think, now that I'm thinking about it, that might be a bit tricky.

<Files\\School Workers\\Policy Makers\\Transcription > - § 2 references coded [7.41% Coverage]

Reference 1 - 4.01% Coverage

So, I guess, I mean, yeah. Of course it can be done. What I've seen from your risk calculator already, that could be linked on social media, there could be an app with this on. So, yeah, anything can be done through social media. I guess when you say looking at people that are at risk, you'd be getting them to fill out a questionnaire about themselves and then you'd be telling them they're at risk. I guess it would kind of be like looking at the purpose of that, and making sure that if they did identify as being at risk, there's actually a care plan, a support plan in place. Because at the moment I feel like pupils with mental health needs as well as social and emotional needs, I think that the external services are really lacking and it's not very clear cut. When we support someone, once you've identified they're either at risk, or they have depression or anxiety, it's not very clear the support that's in place. So it's not, yeah, very clear cut. So, I think the danger might be telling a teenager that they're at risk if they're not actually having any resources in place for them with that.

Reference 2 - 3.40% Coverage

I guess using, whether it's a calculator or a tick box, that kind of thing, I guess it is useful in being able to identify targets, vulnerable groups, you know, vulnerable young people to depression and making sure that there is support plans, but there has to be resources also out there to be able to do that effectively. If there's no resources then it's almost like, why do we need to know who's most at risk if we're not actually going to put anything in place for them anyway. And it does make you question, is it better that they know what the symptoms are rather than whether they're going to be at risk for it if there's actually nothing they can actually do about the things putting them at risk. If that's all out of their control. I mean, is this for the young people or is this for the adults that are supporting them, I don't know. So, yeah. I think just the purpose, yeah, I'm not sure what the purpose would be if you didn't have the resources there to put support in place.

<Files\\School Workers\\Policy Makers\\Transcription > - § 3 references coded [4.83% Coverage]

Reference 1 - 1.35% Coverage

think, yeah, it's acceptable as long as obviously students, adolescents are, you know, consenting to it, obviously. You would want students to feel that this is something they could do. I think any kind of blanket attempt to screen, you know, screen adolescents for depression, which, you know, isn't always, we screen patients for all sorts of other, you know, diseases, illnesses, conditions, however you want to frame it, but you can frame it in clinical terms, I think screening people for depression would be highly problematic.

Reference 2 - 1.55% Coverage

And I’m really concerned that, you know, in the current context of, you know, rising social inequality, for, you know, poverty affecting huge numbers of children, you know the kind of instrumentalization of schools and the kind of transactional nature of school culture, you know, reduction in public health services and mental health services for young people, I would be really concerned that simply highlighting risk of depression without providing students with a means to mitigate that risk, you know, and wider stakeholders to mitigate that risk, it raises probably more problems than it solves, I think.

Reference 3 - 1.93% Coverage

I think it’s really important to do this kind of work, but I think it’s important to do it well and not to do it in isolation or to do it in a kind of narrow way, which, because I think doing it in a narrow way, in an isolated way as a sort of quantitative exercise in gathering data, it raises serious ethical issues and could possibly make matters worse for young people. But that’s not to say that this isn't important because I think the mental health of young people is clearly an important issue. And I think it’s an issue which lots of stakeholders agree, we need to take seriously, so we do need to do work in this area, but I wonder what kind of work we need to do and how we can best go about acting on the information that we find as well. So, yeah.

<Files\\School Workers\\Transcription > - § 2 references coded [5.04% Coverage]

Reference 1 - 2.56% Coverage

yeah, so I see kind of like being stigmatised, you know? Because, especially, I’m imaging in a mainstream environment, if the results, or if they’re all doing their screen at the same time, then the students will be like, oh what did you get? What was your result? You know what I mean? And then feeling a bit stigmatised. Especially if you already have, are already coming from a difficult situation, it kind of just confirms, you know, how different you are, perhaps, to your peers. And we don’t want to, we definitely don’t want to isolate anyone. So I see the risk of kind of being, feeling a bit stigmatised. Potential like confidentiality issues. Or, I don’t know whether students are going to know what their results are, or is it going to be communicated to the parents, but I think that they’re, yeah, at risk of kind of violation of boundaries, really. Not violation of boundaries, but wanting to know, yeah, it depends whether students are going to know what their results are. Or whether it’s just communicated to the parents. But then sometimes, each child is different, like, and I think the way that I was kind of raised is that like there’s adult stuff and child stuff, but some children it’s like parents communicate everything. So, if, you know, something is told or something, yeah, is told to a parent, then they will know, and yeah, sorry, I’m kind of just thinking about the communication aspect of it. And how things are going to be relayed back to the child especially. In terms of probably, that relates again to the stigma.

Reference 2 - 2.47% Coverage

I think you will have, people will have both. Some people will be like, I’m all for it, and then some people will be not so much for it. I wonder if like, even if you did get that early identification, does it then mean that, would that then equal success? I suppose you have to think about what success looks like anyway, when it comes to intervening and helping children who show signs that they could get

depression, but I think like would that necessarily—just because we've identified it and are aware of the fact that they could become depressed, would they, does that necessarily mean that we will successfully treat them or help them? So, yeah. I'm just thinking in the case of perhaps people I have known, if it were identified, would it, I suppose I'm just thinking about what the help would have looked like. It depends what the help would've looked like. In terms of people being accepting, I think yeah. I think both. I think people would like the idea, but I think it would just depend what then happens after you've been identified. In terms of screening, yeah. I think…yeah. I don't know. I'm trying to imagine if I had a child and I got a letter and it said, would you be happy for your child to be screened for depression. I think I'd be ok with it, but I don't think everyone would be ok with it. But I think that there are some parents that would be like, no, you know, we're fine, we don't need, my child doesn't have any mental illness, so I think it would be a big spectrum.

<Files\\School Workers\\Transcription > - § 1 reference coded [2.12% Coverage]

Reference 1 - 2.12% Coverage

Yes. As long as—it would depend I think on what would then come out of it at the other end. So, if we discovered, yes, this person is at great risk of depression in adolescence—what then? What's available to that person, and what would it do? Because unless there is an action at the end—something we can do to prevent, or to help reduce the risk, there's no point. And so, as long as no one's, if we could use it as something to prevent or reduce the risk of—we would use it without a shadow of a doubt. But to just know a high risk or a low risk is irrelevant if we can't do anything to help.

<Files\\School Workers\\Transcription > - § 2 references coded [3.26% Coverage]

Reference 1 - 1.15% Coverage

Yeah, I mean, I think something more, I mean I know this is not the project but next steps would be, I imagine, I mean, if there were some kind of online CBT thing or some kind of resource that was like "And here's something you can now do to", or something, "Evidence suggests these things might mitigate the risk", you know, whether it's exercise or whatever things, even just some simple. Because I imagine if you're left with that as your final screen as, you know, as a child, it's kind of like, that would trouble me.

Reference 2 - 2.11% Coverage

Yeah, more addition, because it feels kind of one's just left. High risk of depression, might want to ring childline! Well, yeah. And then, I can imagine that, you know, if I did that say in a form time, and then said ok, bell's gone, off you go to maths, and you're like, you've just told me I have a high risk of depression, and presumably, I've no idea, but at least a small percentage of most classes would get this flagged up, or you know, you might get one or two children in your class who would get that. And would you, I mean, I wouldn't like to do it in a place where they can discuss results or, because that could be really, if they could see each others' results, that could be very stigmatising. So, I mean, I certainly yeah, wouldn't do it as a group. I mean, I guess you could direct it to do it in their own time. But, yeah, then I

might worry slightly about would they have the support when they got that, ringing childline's quite a blunt tool.

<Files\\School Workers\\Transcription > - § 1 reference coded [1.41% Coverage]

Reference 1 - 1.41% Coverage

I think the risk would be is opening up that can of worms and not having—can of worms is maybe not the right phrase—but not having the support to deal with that afterwards. So, not being able to immediately trigger a referral to a support service, or to have someone on hand to be able to talk about it. Otherwise, it's you know, you raise a doubt in a young person's mind, and if there's no one there to deal with that, then I think that might be, cause some distress as well.

<Files\\School Workers\\Transcription > - § 4 references coded [2.55% Coverage]

Reference 1 - 0.11% Coverage

But it's then, what do you do with that?

Reference 2 - 0.84% Coverage

No, because what I'm thinking is that you get all this information, so they might be saying, yeah, I smoke weed every weekend and yes I was touched in a sexual way, and my parents don't get on, like you've got all that and you've calculated that risk, but then what do you do with all that information once you've got it?

Reference 3 - 1.13% Coverage

Yeah. And people understanding like, do you need to refer on to other services, do you need to, if someone's been touched in a sexual way by like say their uncle, then obviously the police need to be involved and things like that. And it's about being sure that people are able to follow through, because if a child made a big disclosure, and then you're kind of like, right, ok, it needs to be more than a paper exercise in a sense.

Reference 4 - 0.47% Coverage

Yeah. Yeah, I do think so. But it needs to be able to sort of take the information and signpost onto the right services and have that there, accessible, at the time of completing it.

<Files\\School Workers\\Transcription > - § 4 references coded [5.28% Coverage]

Reference 1 - 1.08% Coverage

I think it’s really valuable, but I have, I have a, a real bug bear about young people, erm, answering quizzes and things to identify trends, but if you’re not going to do anything with them? So, to me, so I’m, I would feel very bad for a child who had filled this out and flagged stuff and it didn’t go anywhere, you know? And they weren’t then contacted by a service to, say, to offer them counselling. If they needed it.

Reference 2 - 0.85% Coverage

Yeah. Definitely. I-, it should, I believe that it would only be ethical really to roll it out to people if it then would trigger some support. Because, I think it’s really, erm, dangerous to get into a situation where you’ve got a young person saying this happened to me and effectively, kind of, not being listened to and actioned.

Reference 3 - 0.69% Coverage

That’s what I would, I mean, it’s safeguarding isn’t it? And you’re not safeguarding the child if you’re not listening to what they need and, you know, acting on that and getting them help. The worst thing in the world would be for them to cry for help, and not to get it.

Reference 4 - 2.66% Coverage

Yes. Definitely. Yeah. I mean. I think, erm, not to be political again, but I think the whole ‘every child matters’ agenda kind of laid the foundations for a, more of a kind of, and more recently then obviously, early help, there is more of a focus, more of an understanding from everybody that the earlier you support somebody, and the more prevention you can do, you know, the better. So I definitely think that there’s a place for it in society, yeah. I think society is crying out for it, to be honest. But only if the support then comes with it, do you know what I mean? I think. But the problem is, there has to be a point where you’re identifying it, but you’re not necessarily able to meet the needs so that you can then use all of that data to campaign, or, to highlight the need. Erm. You know? Before you can necessarily access the level of support that is required. Which is totally understandable, but it’s just really painful to be somebody who works in the middle bit where people’s needs have been identified but not met. You know?

<Files\\School Workers\\Transcription > - § 1 reference coded [1.37% Coverage]

Reference 1 - 1.37% Coverage

It would certainly highlight those that require help, wouldn’t it. But then, where do they go for that help? What does that help look like? If I come out as a high risk person, I'm thinking, oh no, ok I need to speak to someone, I need to do this and how do I do that? What are my next steps? So I think that would be really important, is what to do next? Who do I go see? What do I do? Do I see my doctor? Do I phone this number? What’s the follow up?

<Files\\Social Workers\\Policy Makers\\Transcription > - § 2 references coded [1.58% Coverage]

Reference 1 - 0.71% Coverage

The only tiny questions, I wouldn’t say it’s a negative consequence, but the only thing I thought of is one of the questions asks you about did somebody beat you or leave a mark. Now that’s a safeguarding concern. So, if they then flag up yes, how is that information captured and followed up?

Reference 2 - 0.88% Coverage

I guess the challenges will be to get schools to recognise why it would be worthwhile for them to spend time doing this. And what schools then worry about, ok let’s say they do screen for all these things, what do we then do with that when we’ve got that information, because there aren’t any services to help the children is what school’s say. I'm not, depending on where they are in the UK, so I guess that might be a challenge.

<Files\\Social Workers\\Policy Makers\\Transcription > - § 2 references coded [3.52% Coverage]

References 1-2 - 3.52% Coverage

Or, not so much treatment, this is very early stage prevention, I realise, prevention rather than treatment of actual depression. But, nonetheless, I would see as a much more acceptable approach some kind of combination of an offer to the population of young people as to who needs help, like a kind of primary care offer to the population if you like. So, rather than we’ve identified you as having a future risk, as in you’re fine now, that’s fine, we don’t need to go anywhere near you, however, if you would like some help, because of some aspects of your mental health, then we will find some preventative help for you. I think the problem we have at the moment is that an awful lot of kids who don’t get anywhere near the CAMHS threshold just don’t get very much help. Yeah, so responding to kids saying they need/want help rather than identifying ones who may at a future point come into risk of a certain category would seem to be the more sensible approach.

<Files\\Social Workers\\Policy Makers\\Transcription > - § 1 reference coded [1.63% Coverage]

Reference 1 - 1.63% Coverage

So, in other words, what I'm thinking is that it’s probably not a great idea to do screening and identify lots of children and then not have a service that can actually help them. So, in that sense you would have to look at what would happen subsequently.

<Files\\Social Workers\\Transcription> - § 2 references coded [1.74% Coverage]

Reference 1 - 1.24% Coverage

I guess lots of young people would probably come out at high risk. And making it meaningful, what you’re doing and ability to not then be spreading too thinly. And I think I guess I’m thinking from a

resources point of view, I think it would be great, but if I was thinking at work, well we have very high thresholds because of the resources. So, how are you going to make that meaningful and then young people, if young people, I don't know, if you were doing group work or whatever, and there's people in their peer group, in their school, the right people to be putting them with, would that create further issues? They're in one group, they're in another, kids talk, making them feel a bit more left out.

Reference 2 - 0.50% Coverage

No, that's exactly right. Yeah, it's what do you do? Ok, well just go to CAMHS, it'll be fine. And yeah, and this is very, very experienced practitioners as well, and it's not through lack of not wanting to help. I think it's just, through lack of just, what do you do? You know. Yeah.

<Files\\Social Workers\\Transcription > - § 1 reference coded [3.36% Coverage]

Reference 1 - 3.36% Coverage

Well, certainly the professionals will be—are anxious about it. I mean I, because you think, oh yeah, I'm doing a really good referral for CAMHS because that will push it through and they're, you know, it's just, and then somebody got sent an appointment. Well, supposedly, and they never got it. But she worked full time, so to take the child, she needed to have somebody ring her, not send her a letter, and so she missed it, and then it's like oh cross that person off the list, because they didn't attend the appointment! (*laughs*) And it's like, "But she never got the letter! Ever!" So, it's—I think there is this concern amongst professionals about where do adolescents get the help? If they're feeling down, when we know that they're quite unlikely to phone up their GP and when they—we know that parents will do that and not get an appointment, it's quite hard to imagine that any teenager if they plucked up the courage to do that and then they get told that well, you can't have an—when, oh no, you can't come then, you've got to come back another time. So, maybe something like this on an easy access route for young people is better. I'm sort of talking myself round it really, because you just think, if you were that age, or that person, and you rang up and somebody blanks you, or puts you off, chances are well you're just going to go, well, what's the point?

<Files\\Social Workers\\Transcription > - § 2 references coded [4.45% Coverage]

Reference 1 - 1.52% Coverage

The sort of lower level stuff isn't really taken that seriously, they're kind of moved around, so I guess it's really good to identify these things, but yeah it's what you do with it afterwards and how you deal with that label, and whether you use it as a label just not a sort of indicator and try and turn it into a positive that, okay so these are the things you're feeling, what do we do about it? And kind of try and tip it on it's head, rather than a, I think if it was in the wrong hands, like I said, I think it could be like a, a kid could be like "Oh, I've been told I'm going to get depression, so this is how I am now, I'm not going to do this that or the other because I'm going to get depression". I don't know. So, I just sort of verbally spewed up on you there didn't I! (*laughs*)

Reference 2 - 2.94% Coverage

I think that’s difficult. I thought that for ages, because a few years ago I was like oh, we should have like a Twitter account, a Facebook account, like it might encourage like parents and children to talk to us, but I guess my worry, obviously being part of a local authority I’d have to run through everything with what was ok, what was the whole department, like if I could track down who in charge of it—so that’s a stumbling block! (*laughs*) But also I’d worry about privacy. Like people might choose to use those platforms in a less private way and then you’ve got a big argument happening, parents with social media, there’s probably simple ways for this not to happen but I'm not on Facebook or anything anymore—but I was worried about things becoming public, or people yeah putting things in the wrong place and then you get this massive debate and everyone’s putting their two-penny’s worth in. And actually they’re not coming from a background of I don’t know, I don’t want to be disrespectful to our families, because obviously they know themselves better than anyone else, but I think sometimes you get a family just throwing unhelpful advice into the mix. And I would be worried about it becoming a bit of a pool, but if it was done privately then I don’t see any issue with it. It’s, yeah, managing the potential for things to become more publicly debated. And then once it’s out there, it’s out there, isn’t it? You can’t get it back. But that’s me having no clue about IT! (*laughs*) That’s probably not even an issue! (*laughs*)

<Files\\Social Workers\\Transcription > - § 2 references coded [6.81% Coverage]

Reference 1 - 3.46% Coverage

So, yeah, I don’t think there’s anything wrong with actually using the tool to help support other outcomes. Such as, you know, as I say, socio-educative work, or even a referral online, you know, if you’ve got high, and you’re saying well the likelihood is that you’re going to develop depression after 18, well what are we going to do? Are we then just going to leave that child and say ok, well you’re going to develop depression after you’re 18, but now we’re going to do nothing with you. You need to have something in place, there needs to be a plan of what you’re actually going to do with those young people when they hit high. Because otherwise what is the aim of it, isn’t it? You know, what are going to do? We need to do that. But the problem is that the services that we have at the moment, you wouldn’t get, you know, you’re not going to get, you know, you’re not going to get seen, you know, the waiting list is so long. I mean, if we say on a, from this, we’re saying that you really need a referral into CAMHS to deal with that, or is there going to be things that could be put in place whilst we’re waiting, or whilst that’s happening, you know, that we see that you’ve got a risk for depression, so let’s do some mindfulness, let’s do sort of these techniques, let’s work on that sort of help and support you at this level, at the lower, earlier level. And then if it seems like self-harm and other sort of stuff going on, then you’d need that referral in, but there needs to be something in between doesn’t there? Between us doing this and saying you’re high, you’re high risk, and then what do you do with that information? You know, it’s just like, ok, you’re high risk, goodbye, then that’s not going to help is it? And then you can’t really, like with Childline, if you’ve got Childline on there, they’re not going to be able to take all the calls (*laughs*). Everyone going, “Oh my god, I’ve got depression, what do I do!”. It’d be like, you know—there

needs to be a plan in place, doesn't there, that if you're getting high but then how to deal with it, like a management plan of some sort that a professional could then work with.

Reference 2 - 3.36% Coverage

but then I suppose the only thing with that is then you opening up the fact that you might get a lot of disclosures, you know, people who do suddenly disclose. So, then you're going to have to, I suppose, that's going to be a concern there, because you know you've got a high risk of depression on there and a low risk of depression, what are you going to do if a young person tells you that they smoke so they did say yes, I smoke marijuana and I take crack cocaine, what are you going to do? Are you going to go, Ok, than you very much, none of the rest of it has been a high risk, you've actually got a low risk of depression, but we're now not going to address that risk. Or, a child says to you, say you've just done this with a 14 years old, and they go, have you ever been touched in a sexually inappropriate way, and they go, yes. And they've never made that disclosure before, and then you as a practitioner say, can you tell me a little bit more about that? Ok, well the 45 year old man next door has been doing x, y, z to me for the last 3—what are you going to do? You know, you're going to need to think of safeguarding then safeguarding concerns within that, I think. Because one, they may not disclose, you know, then you're just going to get no's. But if you get a young person who discloses to you as a professional, you're going to have to put something on there about safeguarding that that's going to have to be shared with the appropriate agency. Or, you know, if they say to you, yes, my dad's been having sex with me for the last 5 years, you're going to have to do something aren't you? There's going to have to be something on here about confidentiality, about that this is going to be, how it's going to be shared, how it's going to be used, and if you answer these questions in a certain way that raises a concern, so if you've got 14 year old that does tell you that they're drinking a bottle of vodka a night, what are you going to do with that information? You need to be able to do something with that information, don't you? Yes, so that would be a concern.

<Files\\Social Workers\\Transcription> - § 7 references coded [14.32% Coverage]

Reference 1 - 5.13% Coverage

Well, I suppose my worry would be my very initial response was, if you identify a whole heap of children who are at risk of depression, which I would say, if you're going to do that, just by the work that I do, I would say you've got no chance! That this would come back with zero. What are you going to do with those young people? Because then you have a duty of care for those young people if you've identified them. Or somebody does. Because if you had a person that you'd identified as at risk of depression, and that person in two years time went on to commit suicide and nobody had done anything for them, they could quite rightly say that you knew I was at risk of depression, why didn't you do something? So, I think that would be one of your major problems, would be if you identify a whole load of people and they don't get any support, actually how does that help anybody? I don't think it would be news to know that we have a whole load of young people who do have mental health problems. Or that there are—what was it I read recently? It was statistics on suicide I think, or something like that, it was something like this but not from you. And the statistics for young people actually having had any kind of—oh it's to

do with trauma! And even where children have PTSD, they haven’t necessarily had an intervention. And that’s with, you know, with that level of trauma.

Reference 2 - 1.49% Coverage

I can't remember where the information came from, but it was quite shocking actually. And that was adolescents. So that would be my worry is that you’re going to identify all these people and they won't get any support. Because I know how hard it is to get anybody into CAMHS, because they are just absolutely over-loaded. So, if you haven’t got the resources, is it right to identify all these people?

Reference 3 - 1.59% Coverage

Yeah, I think you could get people who are not mental health workers to use it, providing when they did it, somebody’s going to get some kind of response. Because if they took the time to do this with a child and nothing happened, they’re not going to bother again, are they? So I think that’s the key, isn't it? Something has to happen when you do this. Or, it has to be presented as a massive great case for providing something.

Reference 4 - 1.82% Coverage

Yeah. Because that’s easier to achieve than trying to respond to all these depressed people, isn't it? To make sure they don’t get depressed in the first place is probably easier to achieve than trying to provide resources for, you know, however many thousands of potentially depressed young people you’re going to find. I’m not saying don’t do it, but my worry is that you’re not going to have the resources to deal with your results. I could be wrong with that, but that would be my worry.

Reference 5 - 0.58% Coverage

Yeah. And maybe this is what that would help to predict. Although I still think that you would struggle to find a solution for them, or a service for them.

Reference 6 - 2.02% Coverage

An intervention available. So, I mean, somebody might not need it, they might not want it, you can't make people accept a service, can you? But to just do this and then not have anything to offer them is a pointless exercise really, isn't it? The only thing it would serve would be to flag up the need, wouldn’t it? But that doesn’t help that young person does it? It might help you to collate all your information to present it to somebody, but that young person who has then identified as being potentially depressed, but hasn’t got a service…

Reference 7 - 1.67% Coverage

No. I suppose it's just that, yes, this is a useful idea, providing that there is some way of dealing with, you know, people who are identified at high risk of depression, or you know, that there is some kind of intervention to prevent that. I mean, being identified as low risk is quite good, isn't it? You might go, "Oh, thank goodness for that!" But, you know, someone, I mean, would you tell them? Are you going to tell them what the results are?

<Files\\Social Workers\\> - § 3 references coded [1.40% Coverage]

Reference 1 - 0.30% Coverage

Again, the only thing—I'm just thinking as I'm saying it I'm just thinking—the only thing I'm thinking is that the questions about sexual abuse and domestic abuse that are in there, if they tick yes to those, obviously they may never have disclosed that before. So, there may be, the schools may have an issue with that.

Reference 2 - 1.04% Coverage

And also, in general, there may be a safeguarding issue with that. If that's the first time a child's disclosing that, what happens to that information and, you know—because that also might be the child sort of saying yes, this has happened to me. But is that something that they're sharing with someone, is that something that's just going into a system and getting lost because it's—because again, if we were doing that on the trauma one, we would do it on a paper copy, and they would come out as sort of questions that we would go back and ask the child about.
So, we've got like highlighted questions that if they tick anything other than nothing on it, that we would go back and sort of ask them if there's something that they want to talk about. But, if they're filling it in on a computer screen, I don't know what happens to that information, so. And if I was filling it in with a young person, I would be concerned that they might have ticked yes, and that I might not known that. Because I don't know, yeah. Because they may feel like they have told somebody something, but they've not heard it.

Reference 3 - 0.07% Coverage

Yeah. So, there's something in there about a safeguarding issue as well.

<Files\\Social Workers\\> - § 5 references coded [3.35% Coverage]

Reference 1 - 0.47% Coverage

I think so. I just think there might be a bit of concern—reluctance, concern. I think currently as well, things are identified, but there isn't enough support. So, I think that would be a worry that, yes—this may highlight that there's this need, but what is going to fill that? Particularly around mental health for young people.

Reference 2 - 0.96% Coverage

So, I guess it's about identifying, you know, if early help should be provided, but what would that look like. Because I think if you're highlighting to children and young people potentially you could

suffer with depression—and the family as well—and they are on board with that and they are like “Ok, let’s, you know, try and minimise that risk from happening”, but we’re not, you know, there’s no signposting, there’s no help to do that—I think that would just cause more anxiety really. And frustration. If you’re telling us that we could potentially prevent something, but we can't! (laughs) It’s a bit like what’s the point in this if you’re not going to offer anything.

Reference 3 - 0.10% Coverage

Yeah. Because otherwise you’re highlighting a need and there's no plan.

Reference 4 - 0.34% Coverage

I just think things as a whole seem to be getting worse really! So, I don’t know how we go about changing that really. Or maybe it’s just more spoken about now, I don’t know. Young people, I just think their future looks quite difficult.

Reference 5 - 1.49% Coverage

I think that’s the difficulty, because like I was saying, some young people that are struggling or starting to think, you know, might have some early indicators, might not speak to people. They might be more introvert and isolated and therefore would they, you know, if you’ve got young people for example that aren’t going to school, or are feeling anxious about whatever, you know, you could have—domestic abuse quite often we see it. They don’t want to be separated from mum; they stay at home, they’ve overprotective, they don’t go to school, they’re not going to access something like this in school.
But then, if they’re doing it online on their own, are they then going to be able to seek out that support, or is it just going to be reinforced to them and actually, you know, “I'm not in a good place”, or “There’s something wrong with me”, or “This is going to get worse”, and feed into it. That’s my only concern, if they’re doing it on their own, what would the outcome of that be? Would it help them, or will it hinder them, really?

<Files\\Healthcare Workers\\Policy Makers\\Transcription > - § 1 reference coded [1.59% Coverage]

Reference 1 - 1.59% Coverage

And the model seems, although the money has gone to the CCGs, so straightaway we're saying it's a medical problem, what message are we giving to our children? We're telling them they're ill, constantly. Why are we trying to put them into a medical model, you know? We should be preventing them from ever needing the medical model. So, to put the money into the CCGs, the clinical commissioning groups, to me, even that in itself, gives a subliminal message that our children are ill. They're not. They just need resilience, for most of them, they just need some resilience.

<Files\\Healthcare Workers\\Policy Makers\\Transcription > - § 4 references coded [13.79% Coverage]

Reference 1 - 6.51% Coverage

Yes, I think there is, so years ago, which was looking at early interventions around schizophrenia which is obviously far more burdensome, it's far more extreme if you like, but a lot the sort of issues there were exactly those sort of issues, you know, do you label somebody early by doing this? Erm, and therefore, somebody will obviously, will you get false positives, and you get a false positive and then, you know, that somebody's identified as being high risk of depression, does that affect their behaviour, does that affect the systems behaviour and is that negative or are there sort of unintended consequences of that? So I think part of your work would need to sort of at least reassure people that that's not the case, or you've got mechanisms in place to manage that risk.

Reference 2 - 5.07% Coverage

so I think that's true, you know, about this idea that we're sort of creating a mental health problem by talking about the mental health problem. So, do you sort of stimulate issues by just raising them? Now, that's really difficult to deal with because, you know, the counter argument there is we haven't talked about it in the past and everybody was suffering quietly and now we're addressing it, erm, so, erm, yeah, so I think it's difficult but I think having some, erm, you know being aware of those challenges and sort of making that part of the research project would be quite important.

References 3-4 - 2.21% Coverage

Yeah, and by acknowledging that there is this risk of, erm, labelling, for want of a better word, so that's the biggest risk, and the consequences of the labelling may both be to the individual but to the system and so how do you try to mitigate that risk, erm, where appropriate.

<Files\\Healthcare Workers\\> - § 2 references coded [0.65% Coverage]

Reference 1 - 0.50% Coverage

I think I'm certainly very aware of, you know, young people and what they think of our service, and about how you explain stuff to them, and how you validate how they're feeling and going, you know, some young people are—I think—I…I say to them, well, you know, "I'm going to let you go out there. You maybe need a bit of treatment a bit later on, but go live your life, go do your GCSEs, go do that", and then you can sort of—because I think that's the right thing for some of them—because I think some of them will get sucked into just wanting to be a patient.

Reference 2 - 0.15% Coverage

Yeah. I think there are risks. I think, again, if you go back to young people's personalities it might, it may become their identity. If they haven't got anything else.

<Files\\Healthcare Workers\\> - § 5 references coded [2.83% Coverage]

Reference 1 - 0.19% Coverage

It could…yeah…it could affect, yeah. It could mean that if you knew that you were—could be a kind of self-fulfilling prophecy almost. Yeah.

Reference 2 - 0.56% Coverage

But, yeah. Yeah, it does—there is a danger of it being a self-fulfilling prophecy, if you're told that you're… It's like with those, you know people do those DNA tests and lots of people are like "oh, I'd never want to know"…maybe you should be allowed the option not to know? So maybe it should be self— rather than—but then it's not finding out people who wouldn't be found out? I don't know. It's a tricky one.

Reference 3 - 1.49% Coverage

I don't know. I don't know how…I don't know how I feel. I feel like—I feel like it should, I…I don't know. That's—so there's sort of—so, this is recently, like sort of thinking how it would just be nice if it was normalised that we all feel—because when you think about how arbitrary it is to like be diagnosed with depression, it kind of is quite meaningless in the end, when you think about like how it's done.
And then, so maybe it should just be normalised that like we all have, sometimes, we cope with things better than other times and sometimes we can't cope and sometimes we feel really low and you know, like, more of a kind of like broader, like, kind of—there's so many different things that affect how we feel and how we're managing and rather than, it being…
I don't know if, like, all the kind of attempts to, like, destigmatise mental health actually do what they're trying to do as well as they are. I think—I don't know. The whole thing about like the invisible, broken, I don't know. I don't…I don't know. I'm trying to, sort of, still working out what I feel about that.

Reference 4 - 0.43% Coverage

Yeah and like they have this kind of odd, because it's—the ill—this idea of it being an illness is kind of a metaphor, like it's…illness is a metaphor, but it's—I don't think it's necessarily a metaphor. I know it is kind of, like. I don't know how much of it is a metaphor, and I don't know if the metaphor works.

Reference 5 - 0.15% Coverage

Yeah. So, I don't know if the attempts to reduct—reduce stigma actually will do, or do what they're trying to do.

<Files\\Healthcare Workers\\> - § 2 references coded [1.17% Coverage]

Reference 1 - 0.54% Coverage

I think, potentially—I think—especially if these—everybody sort of flagged up somebody as particularly high risk for instance, that could, I suppose…I suppose it depends on what that means for the adolescent. Does that mean they might sort of misconstrue that that means that they have depression or they're definitely going to get it one day. And that might—it might sort of change the way they maybe interact with other people or sort of go about things day to day as well.

Reference 2 - 0.64% Coverage

I think labels mean a lot to young people as well, and people who sort of identify with them in a good way and a bad way, so, it would take a lot of thought as to how that was approached, I think. Mmm. Potentially thinking about how adults use the labels when they go through screening tools as well, so people would be put in sort of high risk of cardiovascular disease, for instance, maybe looking at how…how they approach those sort of labels and how useful it is, or not useful it is, to be given the information about high risk or low risk. But…I don't know.

<Files\\Healthcare Workers\\> - § 6 references coded [2.58% Coverage]

Reference 1 - 0.40% Coverage

Well, same as any screening tool. You know, you get false positives, you have unnecessary morbidity. You have the pathologisation of somebody who doesn't need to be pathologised, particularly at the age and state that they're at—that it's better to have a narrative which is just about life circumstance than there's something wrong with you.

Reference 2 - 0.57% Coverage

I think—I think that's a real risk. Because I think in adolescence, they—the capacity to adhesively kind of identify with…with particular roles and stereotypes is…is much greater and there's that search for kind of identity and meaning. And we certainly see in some young people that come here that they're—they're overwhelming choice of identity is mental health patient. When actually there's lots of other identities they could be choosing, but that's the one they're gravitating towards.

Reference 3 - 0.57% Coverage

And I could imagine, those that want to sort of avoid the…challenges, of thrashing through all the other aspects of identity—gender, sexuality, whatever—might then slip in the kind of "oh, woe is me, I'm going to get depression later, I need to be-" and also the slight little ornery aspect of it, "I need to be-", "Mother, don't treat me like that! I'm at risk of getting depression! How dare you ask me to clear my room up!" You know, that kind of terrible bullshit sort of response to-.

Reference 4 - 0.31% Coverage

So, they could use it against their parents as well, and against their teachers “You can’t possibly give me this much homework! I’m one of those children that’s at risk of developing depression!” It could just be (makes vomit noise) disaster! In the hands of some…

Reference 5 - 0.42% Coverage

I’m sure it would be perfectly acceptable to lots of parents who worry about their children. But there—as I’ve said, there’s—that’s sort of mixed up with that whole kind of letting go of your…your child in adolescence thing. And thinking, you know, it’s the end of the world when they won’t eat breakfast. So, it’s in—sort of could be in—that category, as well. So.

Reference 6 - 0.31% Coverage

And…and apart from that, it would just be the delight of the worried well. They would just be going online, finding it, and going “Oh, ah, think I’m going to get depressed!”. I mean, just the horror—the horror, really—in that respect, in terms of the wider community.

<Files\\Healthcare Workers\\> - § 1 reference coded [0.59% Coverage]

Reference 1 - 0.59% Coverage

Ok, it’s not quite a label because you’re only high-risk for depression, you’re not being told you are depressed. But there is of course that danger that, you know, you’re told you’re high-risk for depression, therefore that equals you’re depressed in the mind of the adolescent.

<Files\\Healthcare Workers\\> - § 5 references coded [4.13% Coverage]

Reference 1 - 0.71% Coverage

Well, for our young people here they worry about stigma; they worry about a reputation, they worry about not getting jobs, they worry about not getting to the right university. Some of them who are not well will think “Well, if everyone knows about it…”, or “They’ve already written me off and said I’ve got depression, what’s the point then? What’s the point of carrying on?”
So, some of them who are at the tipping point have to be careful about what—how you sort of feed things back to them, and how you discuss things, because you never know when they’re going to tip into, you know, the down side in the depression or the suicidal thoughts, all that sort of thing. So, although you don’t want kid gloves all the time, and you can't—you can't never tell them negative things, or, you know, sort of have those sort of conversations—it’s just about how it’s packaged.

Reference 2 - 0.73% Coverage

Well, the thing is I don’t. I don’t know if—I mean there’s clinical depression and there’s low mood and there’s, you know, feeling a bit unhappy. So, if it’s a screening tool for clinical depression, or for low mood, it, you know, there’s a difference. Because I’ll often get students sent to me by a teacher saying that they’re depressed when actually they’re just having normal, you know, adolescent, human feelings and

you are actually allowed to be a bit miserable. It just depends on how long it's going on for and if it's affecting the rest of your life. So, I think language is quite important—of how these things are talked about. Because we talk about making it normal, and it's not normal to want to kill yourself and to think that there's no fun in everything every day. But it is normal to feel really miserable when you wake up in the morning because something's happened and it's upset you.

Reference 3 - 0.27% Coverage

And it's—we don't allow the kids sometimes to have that normal, because then they don't have the—they don't have the flipside of when things are really great, because we're always telling them that you shouldn't feel sad. So it's, you know—it sort of messes up what their emotions are allowed to be. We shouldn't medicalise everything.

Reference 4 - 0.96% Coverage

Yeah. I mean, from my—thing is, people will try to medicalise everything—they'll try to medicalise the fact that a student has said that they can't sleep for three days when they're actually just, you know, staying up at night later—they've had six hours sleep and then they've, you know, been late for school. So, that doesn't mean that they're depressed, that doesn't mean that there's anything medically wrong with them, they just, you know, went to bed late and got up late. Or the lessons are boring, or the classroom's too hot, or they've just had their dinner it's, you know, post-lunch, boring lesson, all of those sorts of things. So, it really, yeah—I think that there is a tendency to over medicalise normal life. And the internet does that for us as well. So, you look up something because you've got a lump on your arm, or you know, you've got a rash that's come up and instantly you've got something medical that's terrible and you need to go to a doctor. That's what I think, that's part of our battle is: we've got to make it normal to talk about stuff, but also make it normal to have feelings and emotions and be sad and happy or crying or all of that stuff.

Reference 5 - 1.47% Coverage

A balancing act for everybody, yeah, I think. Because we don't, it's—we want the kids to talk to us, we want their friends to tell us what's going on, we want their parents to talk to us as well, we all talk to each other—but what we don't want is for them to be running to us every five minutes, you know. Because something's happened and they're upset and not be able to have to the tools to deal with that.
So, we would never say, "Oh, for goodness sake, it was just this that happened", but we'd have to then spend time talking through "So, why do you think you felt like that? And why do you think they said that? And why do you think it's upset you? So, you're having normal feelings, aren't you?" And they're like, "Oh, yeah!" And it's the same with anger for the young men as well, particularly. It's fine. It's fine, everyone feels angry, it's fine to feel like that but it's what you're doing about it and it's whether or not it's affecting everything, it's whether you're controlling it or do you not like the feeling you have when you feel like that. Does it happen all the time?
There's a point where it gets medical, there's a point where all of these things could be a medical problem, but initially, for the vast majority, it's not. And it's just how to manage that, or how to deal with adolescent emotion. And I think a lot of grown-ups forget what it was like to be an adolescent. Who wants to get out of bed? Who wants to go and sit in a classroom? You know, you'd rather be out with your friends, or you'd rather be out experimenting, and you know—the adolescent brain, there's so much research done on it now that...that sort of shows us that they're incapable of gauging risk. That's what they're designed to do is to take risks and to have these hormonal surges and things like that.

<Files\\Healthcare Workers\\> - § 5 references coded [3.16% Coverage]

Reference 1 - 0.99% Coverage

Mmm. I think it—I think today's culture expects everybody, well, promotes everybody to be perfect constantly. And everybody that you see outside of yourself projects that image, and it's not...it's not thought of as acceptable to even have any sort of low mood and I find that they either spiral out of control worrying about worrying, or feel like depression is sort of something that you catch? A bit like a cold. And, have quite unusual ideas in that way, like "Oh my god I'm depressed",
and then you think, as if somebody would be saying "Omg have I got hepatitis", or something like that! (laughs)
It—it's not...it's not a—it doesn't seem to be looked at so much by them as a...as a big accumulation of, well I can't think of the word, but collection of all of what's going on in their life. It seems more to be like, I've got it or I don't. And that grey area where most people live, it's not really something that they seem to understand too much.

Reference 2 - 0.73% Coverage

That, I think, high risk of depression, yeah, ok, it does have that slightly—which I find a little bit— that catching depression sound to it a little bit. You know, you're high risk of HIV if you're a man who has sex with men, yeah that's true because you are, but it's not—that's a binary situation isn't it? You've either got HIV or you don't. It's, depression—I find, is a...is a difficult one—because there's already that confusion of: I've got it, or I don't. And I think high risk of depression is good, but it's almost like you're nearing the line and suddenly, you might cross over that line, and then everything is going to fall to bits—where the grey area that is—it's the bit where everybody is.

Reference 3 - 0.58% Coverage

Like, nobody has got no depression, and nobody has got all the depression. Well, some people do, but it's...it's—yeah, most of them are just going to be feeling a bit bad. And maybe if they're feeling a bit bad, one of the big reasons—which all of those questions are—is because they don't have any confiding relationships, perhaps making there a good suggestion of where you can find those. You know, we're worried about you, and here's where you can find somebody to help you, because we're interested. Because I think that's what they feel society isn't.

Reference 4 - 0.60% Coverage

I think it's a similar argument to the knife crime though—is it increasing or are we just reporting it more? I think it's a—you know, you hear in the media, how this is the safest time—on the one hand this is the safest time in history, and on the other hand, America is at war with seven countries and there's five stabbings this week. So, I don't know what the actual prevalence is. Was everybody always depressed, and they just thought this was part of being married? Or—which I think was the case a lot! (laughs) I think it's, yeah—it's hard to...it's hard to say, really.

Reference 5 - 0.26% Coverage

Yeah. The same thing again, is it normal to be a bit low? Or is it? Has that always been there? Or now are we going "Well, we're not having this! This is unacceptable! We should never feel like this!". But if you don't feel low, then you can't feel high.

<Files\\Healthcare Workers\\> - § 2 references coded [0.77% Coverage]

Reference 1 - 0.43% Coverage

Yeah, yeah. And I guess it's an ongoing question with it because it's different if you say—if you took a test where you might be at risk of cancer, or they can do a lot of tests on babies now can't they, to identify, "Oh, they might get this disease when they're older". So, what if you then do it— and you can't always prevent people from feeling depressed, I don't think—sometimes it's a normal, well, it's a reaction, isn't it? To these events.

Reference 2 - 0.34% Coverage

Yeah. Yeah, I think that is it. The symptoms aren't always obvious, or the symptoms will vary so much from person to person, whereas in physical health you can generally pinpoint these symptoms
that you call this disease, and then this is what you do, and it's very clear cut and they do have a lot of those pathways to support them with that process.

<Files\\Healthcare Workers\\> - § 2 references coded [1.03% Coverage]

Reference 1 - 0.78% Coverage

Because then I guess, you know, the other issue is you have the ones who are not clinically depressed but who want to be seen that way. And that's the flip side of the kind of the cohort we're talking about, which, you know, they want to be seen as not coping, and they—and I know this sounds terrible, but I mean I do come across this a lot—you know, the kids that's kind of like their very typical identity. Like, they are depressed. They are duds. They are in crisis. They need a lot of support, you know. They're the ones that are crying in the bathroom and…yeah.

Reference 2 - 0.25% Coverage

Well, I have an opinion about why that is, and my opinion is that their needs are not being met elsewhere, you know? And/or, they don't know to ask for the support that they need. I

<Files\\Healthcare Workers\\> - § 1 reference coded [0.46% Coverage]

Reference 1 - 0.46% Coverage

Yeah. And I don't know what that's about because—I mean when I was a young person at school, I don't remember—I mean I guess I had my child head on then—I don't remember it being such an issue where so many young people were being referred to mental health services that were going through life events. Because we don't provide counselling here, we have counselling services on the island, so I'm not quite sure why people don't feel equipped to deal with that?

<Files\\Parents\\Tracnscription > - § 2 references coded [0.80% Coverage]

References 1-2 - 0.80% Coverage

Yeah, personally, no, I think it's great. I think it'd be great for a lot of kids to do this. I do sometimes wonder about the kids who are slightly more paranoid or OCD if they're sort of faced with a high-risk diagnosis, whether they would like start freaking out and be like, "Oh my god!", you know? (*laughs*) Whether it escalates some things with them. But, yeah, no, I think it's ok.

<Files\\Parents\\Transcription > - § 1 reference coded [1.30% Coverage]

Reference 1 - 1.30% Coverage

they probably, it's a start, to try to give out a questionnaire, but, as you know, the problem with depression is that it's not a stable trait and the issue is in a way, at what stage in a child or adolescent's kind of life, certain events are likely to impact how they feel, and I feel those are some of the more, when they change schools, exams, and the transition I suppose from, again, from leaving school to, to adult life.

<Files\\Parents\\Transcription > - § 1 reference coded [2.20% Coverage]

Reference 1 - 2.20% Coverage

That's, we're classified as a nanny state, it's all those things, but I don't know, sometimes, if you tamper with something too much you could end up defeating the object and this is the problem. Things can escalate and I know it's still early stages now, but I just want the best for her, you know? And I don't want things getting worse, you know. I don't know. It is a delicate stage right now, to keep her, you know, it's a very delicate stage when she's in this condition. And we see cognitive behavioural treatments out there, but at this stage, I really want her to just try and overcome it, you know, without having to be under pressure to go through all these kind of various things.

<Files\\Parents\\Transcription > - § 2 references coded [3.26% Coverage]

Reference 1 - 1.78% Coverage

I definitely think, not necessarily stigmatising, as I say, I do think depression is talked about a lot now. I mean, I don't know how kids react nowadays to depression. They all seem a lot more woke nowadays! So, I'm not sure whether it's stigmatising them, but I think it does, having a risk calculator for depression, does create that, could create that self-fulfilling prophecy. Which is why I think it needs to have, I mean, as I say, I don't know how they work it out, that's why I think it, but, that's why I think these questions seem quite basic in terms of what's there.

Reference 2 - 1.48% Coverage

I was just…and I was like, oh she's not at that stage, we're not in America. She, it's just her personality and she will hopefully get out of it.

<Files\\Parents\\Transcription > - § 1 reference coded [0.84% Coverage]

Reference 1 - 0.84% Coverage

If they know about it, maybe. They might think, oh I'm going to get depression. Yeah, they might think, if you're at high risk. Maybe. It depends how they think, as well, how their family, how their support network is. Yeah, because if they haven't got, they might just think, oh I'm going to get depressed, not saying that will make them depressed, if they think that way and then they just think, oh I'll just have to accept it. I don't know.

<Files\\Parents\\Transcription > - § 1 reference coded [1.19% Coverage]

Reference 1 - 1.19% Coverage

There's a lot of awareness now, there are a lot of people who are saying, "Oh, I'm depressed", so there isn't stigma, but there is a lot of people who aren't depressed who are saying, oh I've got mental health issues and oh blah blah, because it's a bit trendy. She talks about it being trendy to have mental health problems. Which is very, that is quite alarming really for the people who really do have mental health issues, because obviously they're then taking up time and space that should be used for other people who actually do have problems.

<Files\\Parents\\Transcription > - § 1 reference coded [0.82% Coverage]

Reference 1 - 0.82% Coverage

Yeah! I would still, you know, be talking to a cousin and have them casually just say like, 'oh yeah, well, you know, when you were crazy'. And it's like ah, yeah! That was what, twenty-six years ago? And that's still like a part of my label, because we were all teenagers then, you know?

<Files\\School Workers\\Policy Makers\\Transcription> - § 3 references coded [3.32% Coverage]

Reference 1 - 1.45% Coverage

I'm just trying to think because we've got quite a few children that are quite anxious at school, and I think this would probably make them feel a bit more anxious if they thought they were going to develop it, and it might cause them to have the psychological effects that they, you know, they might be more likely to develop it if they're thinking in that way. Because obviously, the thinking process…Yeah. I'm not sure.

Reference 2 - 0.76% Coverage

Yeah. Yeah, definitely. Because I think even though these are definitely risk factors, they clearly are the risk factors for young people developing depression, I think, yeah, it could be like a self-fulfilling prophecy.

Reference 3 - 1.11% Coverage

So yeah, I think it has to be used, it would have to be used with more caution because, yeah, it could be just a self-fulfilling prophecy and it could affect their resilience as well and their ability to think positively if they think that, oh I'm just going to get that anyway. It could make them feel depressed! (*laughs*)

<Files\\School Workers\\Policy Makers\\Transcription > - § 1 reference coded [2.22% Coverage]

Reference 1 - 2.22% Coverage

I think my concern might be that if you then present students who are filling in these questionnaires with a sort of, I almost want to say diagnosis of risk, then that in itself could contribute, that could be harmful potentially for students to see that. Especially if, you know, if they're already having a difficult time because if the, you know, what they've taken, the indicators, you know, the issues of abuse, or if they're feeling like they're not valued or whatever then, to then be told that you could be at risk of depression or you know, even high risk of depression, I think it's potentially ethically quite complicated. So, that is something that I would be concerned about. I think, without wanting to suggest that the kinds of data that you could collect are not valuable, because I think it almost certainly is very valuable if it's used in a particular way.

<Files\\School Workers\\Policy Makers\\Transcription > - § 1 reference coded [1.23% Coverage]

Reference 1 - 1.23% Coverage

Yeah, I mean, I think that we certainly need more focus on these particular issues of mental health issues. And really identify by what we mean by poor mental health or mental health issues. And certainly in young people, you know, it's very easy to confuse what is just a young person or a small person, a young, you know, a very young person having a tantrum or being upset about a thing and when they've really got serious, serious problems.

<Files\\School Workers\\Transcription > - § 1 reference coded [1.28% Coverage]

Reference 1 - 1.28% Coverage

I can't see any, because I can only see that it can be a good thing in our line of work really. But, I suppose, I wonder if maybe it creates that, like you know, I suppose just because you're at risk of, doesn't mean you're definitely going to develop depression. I suppose it's a risk, isn't it? And I don't know whether it might push you down that, I don't know if it's self fulfilling prophecy, well, that's what I've been told, it's going to happen, so that's kind of the path that you end up going down.

<Files\\School Workers\\Transcription > - § 3 references coded [1.41% Coverage]

References 1-2 - 0.90% Coverage

And another risk is kind of just a bit of a like, not placebo, but self-fulfilling prophecy of, you know, and you do see it, to be honest, when students know that they have a particular diagnosis, they over identify with it. And I think they feel comfortable. I think it makes them feel comfortable. But that is probably a big fear of this kind of, oh yeah, he said that I'm likely to be depressed, and you know. I hear students kind of say things like that quite plainly. So, yeah, I think those are probably the main few that come to mind.

Reference 3 - 0.51% Coverage

I think it depends what the help would look like. Because I think what you don't want to do is you don't want it to become like a self-fulfilling prophecy, and you don't want to, yeah. And then also, yeah, I think that's the main thing, you don't want it to become like a self-fulfilling prophecy. And, yeah.

<Files\\School Workers\\Transcription> - § 2 references coded [3.94% Coverage]

Reference 1 - 0.93% Coverage

But no, you know, I guess my only other concern would be for a young person who is very, very vulnerable to be told that they are at high risk of depression who would then—because I do know one of our young people who completely play on that. In a negative way.

Reference 2 - 3.01% Coverage

Well, we have a number of young people who are, the sort of, you'd call them incapacitated to some extent, whereby they will play on every illness, you know? The sort of young person who gets looked after when they're ill, so they always make themselves ill. They like the, I don't want to say attention, but they like the drama that comes from an incident, you know? They're not well, they've got this, they've had that. And they play on it, and they really, they get positivity from the responses of their friends, you know, "Oh you poor thing!", you know? And they're getting that negative reinforcement, I suppose, that they enjoy. But that would be a very minority of young people who would be, "Oh, I'm at high risk of depression! I've got depression! I've got this, I've got that…", just so they would get, you know, that pull on Instagram

<Files\\School Workers\\Transcription > - § 4 references coded [9.90% Coverage]

Reference 1 - 1.57% Coverage

I guess young people themselves. I mean, it's only, although self-identification I guess is always, you know, has its limitations, you know. And I guess insight into ones own emotional state and

understanding of depression as a clinical label, then, you know, feeling sad for a few days probably doesn’t count as depression, but, so, I mean, certainly children’s voices are important. And perhaps even just the idea of labelling and whether, you know, and I think that’s perhaps one point which would be interesting to consider, the kind of, you know, if this tool labels someone as, I guess you’re labelling them as at risk of depression, but if they are not experiencing it, would that potentially be problematic.

Reference 2 - 0.77% Coverage

Yeah. I mean, I mean it’s hard because I think there is more awareness of mental health these days, which is a brilliant, really powerful thing, like, you know, that’s great. But I think there’s also the difficult line between kind of medicalising things that are kind of normal sadness, or medicalising things that are kind of social kind of issues.

Reference 3 - 1.73% Coverage

I wonder what it would be like to have some data fed into a machine and for it to come back, not that I'm anti-machines or anything, but it’s kind of, what that, how that might be experienced, or how that might, you know, what that might be like for the individual who’s diagnosed, you know, especially if it contrasts with how they feel. Or, if you’re saying, well, I feel fine, but all these factors suggest that I might be at a risk of developing depression, I mean, I guess it’s good to know, I guess in similar ways to, there was a while when there was a discussion of, I don’t know what the state of the art is but of genetic factors that predisposed one to depression. And I thought, would I want to know if these were things in my life? I don’t know. Would that predispose you more?

Reference 4 - 5.83% Coverage

I mean, I guess it’s, you know, people, children will have to be told, you know, you are going to be told, it is going to be good to preface it with kind of quite a bit of caveating of kind of the nature of risk, and I think this is one of the difficult things, it’s something we try to work a lot with in science education, but it’s so hard of kind of saying actually, you know, science isn't a, these aren’t dead set truths, and, but it’s really hard to communicate this, because young people might think, well here’s a computer programme, computer programme’s are always right, it’s told me I'm going to be depressed, and that’s not what it’s saying. It’s saying, giving the factors, but that’s quite a hard thing to explain and I wonder whether there could be more prefacing of that than just some kind of, in student friendly language, you know? Because, you know, like a minority report kind of thing, where it’s you know, you will be depressed, you’ve said these things, and to become a self-fulfilling prophecy. Which could be quite powerful, couldn’t it? If you came to a computer based tool that says something. I mean, I guess they’re used to doing kind of quizzes and things like you do in magazines, mostly A, so, but, and pointing out to them that it’s just, in child friendly language, of saying look, you know, it’s, this is just probabilistic, that’s not very child friendly language, this is kind of you know, there’s, this is going to indicate what similar people who gave similar responses, quite a lot of them end up developing depression, this might be useful for you to think about, it doesn’t mean you are going to develop depression, but you might want to think about putting some things in place that reduce the likelihood of that happening. So, I think, thinking

about, I think that ending very carefully, because I think it's that kind of, you know, you immediately read the high risk of depression in big letters, and I know it doesn't say, it says high risk, but I think, especially for someone, I mean, if someone, because anxiety and depression correlate don't they? And it's kind of like, if you see that, if that makes you more anxious or depressed, quite an interesting ethical thing of would it be, is it better for them to know or is it, could you say something like, you know, the results of this survey have suggested doing the following things might help, rather than, without putting the, I don't know. I guess I'd kind of want to know, but then it's kind of, I'd worry about how knowing would make me feel. And if, yeah, if it just said, based on your answers, you might try the following things, yeah, I don't know.

<Files\\School Workers\\Transcription> - § 1 reference coded [0.81% Coverage]

Reference 1 - 0.81% Coverage

Well, not to others if, so long as it's done kind of with sensitivity and kind of in confidence. Erm. But, self, you know, you could create a self-fulfilling prophecy, couldn't you? You know. It would be very difficult to monitor how much self-fulfilling prophecy would play a part in any kind of outcome. I imagine.

<Files\\School Workers\\Transcription> - § 1 reference coded [1.41% Coverage]

Reference 1 - 1.41% Coverage

Yeah, ultimately. You give someone a complex about what they're not. So the accuracy of the tool is quite important. And if you've got someone who has got quite low self-esteem, perhaps not very confident, and that test doesn't come out accurate, what's it going to do to that individual? Well, it's going to make it worse, isn't it? So where you're perhaps doing something to help support that person, that supportive measure might actually not be so supportive.

<Files\\School Workers\\Transcription> - § 3 references coded [3.71% Coverage]

Reference 1 - 2.00% Coverage

Maybe letting the students know what depression is. I think there's a risk of amongst teenagers, and adults, of saying, "Oh I'm depressed" and not actually being, what is depression? That is, kids are very easy to say, they'll say, "Oh, I'm going to kill myself, then", and you think hang on a minute, they've said that, do I take that with a pinch of salt or is that something I need to highlight to pass onto somebody, you know? Because it's just a phase, they go through a phase of saying it in certain, you know, or they'll say, oh I'm going to kill you, and you think, well hang on a minute, how serious is that. And they all go around saying, "Oh, I'm depressed". It's a very common thing among teenagers to say they're depressed, so whether they are or not…you know? The definition of how you're depressed, what are you doing that's depression? Adults are the same. You know? I can say to kids, there's a need to be sad sometimes, sad is different to being depressed, you know, we're all allowed to be sad. I think we monitor too much of our feelings sometimes, I feel it's, we should be happy all the time! And no

Reference 2 - 0.92% Coverage

I think as a teenager, they're after labels anyway, aren't they? It's a period of time where you're trying to fit in somewhere, or make your own—"this is me". I think some of them want that, if that sounds too cynical, I don't know. I think being labelled as at risk of depression could make some of them worse. So, maybe it needs to be careful with the, what the risk calculator is called, maybe. Maybe not calling it a risk calculator for depression, I don't know. It depends on the kid, you know, where they are.

Reference 3 - 0.79% Coverage

Well, you know when you're feeling really down, sometimes it's nice to feel really down. And I know that sounds silly, but I think sometimes it does, sometimes you need that. You know? Why do we watch sad films, you know? You know, why do we watch horror films? Why do we scare ourselves on rollercoasters? I think we need as humans to experience different emotions. Obviously, with depression, it's taking an emotion to an extreme. But, hmm.

<Files\\Social Workers\\Transcription > - § 2 references coded [3.28% Coverage]

Reference 1 - 2.32% Coverage

I think. Well adolescents, I'm thinking more about adolescents by definition want to identify with their peers, want to belong, want to develop their own identity and, erm, personality, and in that process, if they're struggling, can latch onto, you know, something that makes them feel recognised and sometimes I think that can magnify some of the behaviours, in order to feel a sense of belonging. But that sort of comes back to you know why, why are they not, erm, deriving a sense of identity and containment and support from their parents, or their family? Erm, so it's, you know, totally normal to be looking outside of family but also I think a lot of depressed adolescents are in a really fragile position because they don't feel that there is anyone at home looking out for them, being the sort of, erm, secure base. Erm. Yeah.

Reference 2 - 0.96% Coverage

I think, it, erm, as I said, I think over pathologizing is a problem. I think, it depends on how the, you know, you're approaching that young person, and what that young person understands to be your, erm, your beliefs about them. Erm, I think, yeah, really dependent on how it's presented, and how support is provided, and where it's provided.

<Files\\Social Workers\\Transcription> - § 2 references coded [2.03% Coverage]

Reference 1 - 1.36% Coverage

And also, I think, if you were to say to young people for example, there's some young people that I work with and they get a diagnosis of something when they're at a more severe scale, and then their

behaviour more—so acting out—meet that diagnosis even more than before and I wonder if you kind of said, I don't know, you did when they were 11 before there was issues, actually we're a bit worried as an adult you might get—become depressed, then that's in their mind then, and how does that impact their self-esteem and their ability to cope? You know, and their perception of mental health as well, like "Oh, I must get depressed at some point" and then they have a bad day and then they think they're depressed and then, so I think that could be just how that would impact.

Reference 2 - 0.67% Coverage

Yeah. I think that there's risks of, I think, say for example, you're in the high risk group, and then you might, I think they could become more acutely aware that they're in the high risk group, and I've got all these issues actually, and this is why, and making things into an issue that aren't an issue at the moment, but then does it push it more in the forefront of their mind?

<Files\\Social Workers\\Transcription> - § 2 references coded [2.51% Coverage]

Reference 1 - 1.17% Coverage

I think, well I think a lot of parents would like to. And I think some of the children would like it. I've come across a few that are like "I want it recognised, that I feel like rubbish". But I don't know, it's always risky, because there's always risk with labels and sort of, there's that self-fulfilling prophecy element to this, isn't there? Like "Oh, I'm going to get depressed, so what's the point?" "I'm going to get depressed anyway, because all of this stuff happened to me and I feel like this". So, I guess that's a risk to this type of venture. Yeah, I kind of see 50/50 with children and families.

Reference 2 - 1.35% Coverage

Yeah, yeah, that's it, it's always like a really fine line isn't it? Between validation and trying to kind of I don't know, yeah, give them a bit of hope, or it's just the whole self-fulfilling thing again, I think. But I think definitely a lot of them would like that validation. I think, I think it's just silly isn't it, that at 18, things change, when actually, childhood to an adolescent and adulthood, it's just like a spectrum and different people hit different points at different times. To put this sort of mark on it, that you know, we can't diagnose you until this point, when actually, a child could be feeling absolutely awful for years before they reach 18. It does seem daft to separate it.

<Files\\Social Workers\\Transcription> - § 2 references coded [6.46% Coverage]

Reference 1 - 2.68% Coverage

Yeah. I guess that's the thing as well, is that where you've put that, you've put some in brackets and then you've put like cigarettes or marijuana, again, that might not mean anything to them, because they call it by a different word. You know, young people, have different terms that they all would use, like it

could be weed or whatever it might be, that they might not necessarily know that that's what it's called. So like I said, I guess it might just be tough to get them to admit to something like that on a, yeah. And then when I clicked through to the low and high risk, so the low risk I felt was, you know, it was quite good, it says actually yeah, you're at low risk of depression but it's also important to think about and you've got a link to take them somewhere to help them link up with something which I thought was really good. With the high risk, I guess it's just about, I don't know, I think the hard way of writing that making them feel that it's ok, that they've experienced those things, and that "Oh, no. I'm not going to be mad when I'm older!" (*laughs*) It's just one of those that again, it's a tough way to convey that in and in word, I think.

Reference 2 - 3.78% Coverage

I guess it's just, like I said, the only negative might be about the high risk, about how that, the impact of that on hearing that as a young person, and thinking that there's something wrong with them. But sort of, or that they take it on, they adapt it as a label and they're telling everybody that they are depressed, and then letting that dictate what then happens from then on, that they're going to sort of make it a self-fulfilling prophecy. So, I guess that's again why it's good to have it explained to them in more of an appropriate way, that's why I'm just thinking in terms of the wording. Because then it is less likely for that to happen, you know, I've worked with lots of young people, even adults, who heard mention of a potential word that they might potentially have, but in their heads it's cemented that that's what they've got and they definitely haven't. So, yeah, I guess I think though it's more of a beneficial thing than a negative. I guess for any kind of supportive tool that there is out there, there's ways that a young person could use that in the wrong way, if you see what I mean? So, it's like anything, isn't it? If you teach them, you know, about, I don't know, they learn about self-harming in terms of prevention and then they go and try it because they heard of it, it's like, you know, the small number that are doing anything like that in comparison to the ones it would benefit for. So yeah, I think, like I say, I think it's thinking about not making them feel as though there's something wrong with them, I guess is the biggest risk. I've just rambled on for a while basically to try and say that point! (*laughs*)

<Files\\Social Workers\\Transcription> - § 5 references coded [9.63% Coverage]

Reference 1 - 2.45% Coverage

I think it's just really a problematic term. I think over the years and a lot of that time has been with children and families, and I'd say—the majority of that time—and I'd say that—and I've worked with adults as well, homeless adults, adults with psychological issues—and I'd say that overall working class people and ethnic minorities can't make sense of those terminologies. And I would a lot of professionals don't—struggle to make sense of that terminology, so I think it's a problematic terminology which I wouldn't want to use with families unless they have a clinical diagnosis of depression. I've worked with many, many ethnic minority women, Asian women, who are clinically diagnosed with depression and they are some of the highest—highly functional people I've ever met in my entire life. And, you know, I know there's this thing where, "well, you can have depression and you can have this", but actually for me, mental—it's not a mental, a mental health problem—it's a mental illness. It's the far end. So, if something is really inhibiting you from functioning in your life, then that's really serious. And yes, if you

do have issues which you want to talk about and you need help and you're functional in all other areas of your life, that is right too, but at the end of the day, there needs to be some sort of indicator and demarcation of what makes depression. And sometimes I think it just becomes something about identity politics. And I don't know why, coming from two different cultures, speaking two different languages, the medicalisation and academic language around feelings, sadness, loss, further alienates people. So, you know, when I'm well let's give to a client another name for depression, you know, and then they really try to break it up and there's this real, people get told they have something. And that can be really unhelpful too. It can stop them from working through a lot of their trauma and just living with things the best that they can, you know, rather than try to escape them which often isn't going to help, you know. It's not feasible.

Reference 2 - 1.77% Coverage

I think we just live—British culture, shall I say, I'm generalising, but Anglo-Saxon culture in the South is really repressed culture. Right? It's an extremely repressed culture, and there's a lot of projection that happens. So, for example, Asian families are secretive and private. That's not my experience. It's actually really open and probably really well known for using services. Well, they use a lot of services, that's why they have a lot of issues, that's really problematic. You know, and there's, it's a very problem-loaded, language-loaded, and risk-loaded culture. And things are quite black and white and that's, you know, people, it's not about how feasible, a lot of people don't want to change the language because it actually means them talking about their feelings and them being in touch, and that's the professionals, that's everybody, and I think that it's just a reflection of their own, it's not the system, but it's how they feel and experience the world. I think there's a lot of loneliness, there's a lot of sadness, a lot of isolation out there, and I think it's quite difficult for people to access other people. And I think that they will in turn, that will link to them being depressed. If you look at other world, if you've been to other countries, people don't have this terminology, "depressed". You know? And I think you have to be careful, when, working with adolescents, that should not be a way to form their identity. It can be helpful at sometimes, but most of the time it's an incredibly unhelpful word.

Reference 3 - 2.68% Coverage

Mmm. I think they're major. I've just worked with so many, so for example, I work with children, well, young people, women in their twenties, thirties, well twenties, and men in their twenties, who have no understanding why they were diagnosed in their teens with personality disorders, with depression. And they're not. They're extremely well-functioning human beings. And it's just social services has got like this thing over them. Which, and therefore they see me. And I'm like well, you seem fine. You know? You seem, and I've really had to, they've had psychiatrists do this and I've had to write it up saying I don't agree with this because a, b, c, d, school says they're fine with their child, you know, their parents, they have good relationships, they're working, I've observed them, the social worker says they're fine, so that's why I don't agree with this diagnosis. So, I just would never diagnose teenagers. I don't even believe in giving medication, I don't believe in any of that stuff. I'm not really—the worry is that we're going to move to the sort of, how the states are in medicalising children. And most of their behaviours are social, and I think one of the things is that people are quite offended when I say this, but the reality is if you go to Singapore, if you go to Bangladesh, children don't behave like this. Don't like—I know it's

like “Oh, well in your culture”, it’s like no, this is my culture too. Children don’t behave like this. You know? And, and, you know, doing housework isn’t child abuse for children. You know, doing like, stuff I’ve heard is, and the, we also need to acknowledge this is a society where people don’t want children to be children, they want them to be adults, they want them to be sexualised from quite a young age, and liberated and free and having boyfriends and they get lots of mixed messages, and people who aren’t even their parents are giving advice to people about how they should raise their kids, or even if they are parents, you know what I mean? And I see that a lot and I just sort of think, you're some of the most clueless people I’ve ever met in my entire life. They’re fine, they’ve got boundaries, they go to school, you know, and yet, they give sort of, want to give psychological advice on how they should raise their children, and it’s fascinating. And really unhelpful.

Reference 4 - 1.30% Coverage

Everything’s becoming really hyper—that’s the shift—things are becoming quite medicalised. Crazily so. And what’s happening is everybody, like there’s an obsession with mental health, and what does that even mean? And you have Lloyds TSB who are doing it, people think this great—I don’t! I’m like what the heck does that mean? Teaching children like gender? This is such an abstract term, why do 8 years need to know about gender? When I was 8, all I worried about was that if my best friend was still my best friend, or she was best friends with someone else. If I was going to get that dress that I wanted to wear. These are things that are just basic things that I worried about, you know, if my parents are going to let me go on the school trip or not. I don’t care about gender and identity, and this fear of difference. You know? I just don’t, I think it’s crazy, and the next generation, I feel so sorry for them! Talk about confusing them! I mean, why are we, teaching complex concepts to children? It’s just really unhealthy.

Reference 5 - 1.44% Coverage

and then what happens, the danger is people’s identity might be embodied into these things, “I’m depressed” because the advantage, so the whole concept, the whole advantage of being an individual, which I thought was normal is trying to create an identity is a very difficult thing to do by yourself, and loads of people struggle with it, so they go on and on about being depressed, or having, being gluten intolerant, or being a vegan, you know, and they’re militant about it and I'm like who the hell cares that you’re a vegetarian, you go to India, like that’s most of the population, like they don’t go on and on about being vegetarian or vegan or. I’ve got this one friend who goes on and on about being a dad, even though his ex-wife is the main carer, and it’s like, do you know I’ve got people who are dads all the time, they don’t go on and on about it. So, I think that’s the other thing about their selves, people have to experience having lots of different relationships in terms of their family, or the community, or they will have real, not a great sense of self. Which is where the social media comes in. Because they’re not really having a real, you know, relationship with anyone. Anyway, I’ll stop ranting, you’ve been so patient to listen to

<Files\\Social Workers\\Transcription > - § 1 reference coded [2.57% Coverage]

Reference 1 - 2.57% Coverage

I think that's a difficult question. I think it would be challenged if we were to start thinking about, kind of, profiling so to speak, children, based on all their demographics, and putting them into categories of high risk, low risk, medium risk, I'm not sure that that would go down well. Erm. How, family, yeah, it is about who's role that would be as well. Erm. And how that process would be negotiated would be, I think, would determine how comfortable as a society we would be with that, erm, yeah. Because I think there's a lot of labels that are placed on children, and then whether then, you know, a risk of mental health label is put on a child's head from early on, I'm not sure how that would be, yeah, I don't think it would go down well! Erm. Especially, you know, I think, yes, we've made a lot of progress with mental health and stigmatisation, but I think there's, we've got a long way to go to see it on par with kind of physical health, erm, so yeah. I'm not sure the kind of route of, erm, almost profiling, would feel comfortable.

<Files\\Social Workers\\Transcription> - § 2 references coded [6.15% Coverage]

Reference 1 - 3.36% Coverage

The purpose of it, and what would happen next. Because this potentially would be quite anxiety provoking. Risk calculator for depression? "What, I've got depression?!" It sounds quite scary in itself, doesn't it? So, it would need some kind of information with it as well, wouldn't it? About what, you know, maybe something about depression itself, and why it's important to identify it, and the sorts of things that, you know, can cause, it may be just a general little, we have leaflets to go with our services that kind of tell the child a bit about it before they attend so they know what to expect. So, it might be about what to expect? You know, this is, we're asking you to complete this because…, and depending on the outcome, this is what might happen next… Because otherwise, they're not really giving informed consent, are they? To just complete a form without knowing what you might do with it.

Reference 2 - 2.79% Coverage

Oh, that's true. Yeah. I suppose you could have this on say the [mental health] website, or I don't know whether they might already have their own stuff. I know there's some really useful things on there. Or maybe on the Childline website, or something. Somewhere where young people go who might, as long as it has something with it that tells them what they need to do about it. Or it, you know, rather than they just fill this in and then think, "Oh my god, I'm at high risk of depression. I'm just like my mother!" Or, you know. "Just like my father!" Or, you know. "Oh god, I'm going to end up on tablets for the rest of my life". And all the other worries that they might have about the word depression. Because it's not a happy-clappy word, is it?

<Files\\Social Workers\\> - § 1 reference coded [0.29% Coverage]

Reference 1 - 0.29% Coverage

: I think there could be both sides. Because I think in some cultures, some peer group cultures, I think it might be quite a cool thing (laughs) to have a diagnosis! I think there's…I don't know, anecdotally, maybe in—just in the clients I work with obviously—high need, high risk families.

<Files\\Social Workers\\> - § 7 references coded [3.09% Coverage]

Reference 1 - 0.29% Coverage

I think that's quite challenging, isn't it? Because I think with the high-risk stuff, what you're saying is they still don't actually have it at the moment—we're concerned about where they're going. So, you don't want to go too far because you don't want them to kind of become anxious and worried about things.

Reference 2 - 0.50% Coverage

I think for some young people; they will carry that. I think they will. Because some of them are sort of wired that way anyway—that they've got an anxiety about it. Particularly if they've got a parent who's got a mental health issue. So, some of them will already be concerned that, you know, there's a—they're predisposed to having problems or—and some of them who are already like you know, perhaps predisposed to be a bit anxious and things anyway, will then see something like that and go, "Well that's it, that's my future".

Reference 3 - 0.43% Coverage

I don't know how you get around that because the fact that they're kind of already looking that stuff up, the fact that they're, you know, they've got that kind of history means that that's kind of within them anyway. But yeah, that will almost become, almost a bit of a self-fulfilling prophecy probably, for some of them. But then, you have to weight that against the others that will then, you know, well then you can then put in preventative stuff, so.

Reference 4 - 0.50% Coverage

Yeah. I think so. Yeah. And, I think for some—I'm just trying to think of some of the young people, you know how to help the young people that I'm thinking of—and I think for those, for them, they are different in some way, and they kind of know that. And so, this would be something else that would make them different. But I guess, that's kind of part of their identity in a way. It's kind of part of their story and their narrative. And then that's up to whoever is working with them on it, to kind of explore that with them really.

Reference 5 - 0.96% Coverage

So, yeah, because we're just about to start life-story work here with looked after children as well, so that's obviously huge for a lot of those young people. Because they don't—some of them don't have any relationships with their parents anymore, and some have very strained relationships with their parents. But obviously, they've still got—are carrying their genetics—and for those that, you know, have got parents that misused alcohol and drugs, and had sort of severe mental health disorders and, you know, experienced a lot of domestic abuse and things, they've got major fears about what that means for them. So, if they've got parents with massive mental health disorders and then they come up as high-risk,

that would be quite worrying for them because they'd be like "See, I knew I was just like my dad", or "I knew I was just like my mum". Whereas then we can turn that into, "But we can do something about that, because now you have an awareness, and your life is different because you're not in that situation".

Reference 6 - 0.01% Coverage

.

Reference 7 - 0.41% Coverage

I think the only kick back you might get from some areas, just thinking kind of, you know, devil's advocate, is that some people might have an issue with it being young people and there being like a future potential label put on them. You know, you're going to get some people who are like you know, "Oh, you're predicting negative things for their future", and them seeing that as being a bad thing as opposed to being a preventative thing.

<Files\\Social Workers\\> - § 1 reference coded [0.43% Coverage]

Reference 1 - 0.43% Coverage

Depends on the research and the stuff to back it up, I suppose, isn't it? Because if it's just something plucked out of the air, some parents may think, you know, who are you to say, or how do you know that this is a viable indicator, I guess. You know, labelling my child potentially at this young age.
