# Supplementary material for: No prediction without prevention: A global qualitative study of attitudes toward using a prediction tool for risk of developing depression during adolescence
Source: Glob Ment Health (Camb). 2025 Jan 3;11:e129. doi: 10.1017/gmh.2024.136 (PMC11704374; doi:10.1017/gmh.2024.136)
Supplement: Kohrt et al. supplementary material [file S2054425124001365sup001.zip › Supplemental File 4_Themes by country and quotes.pdf]

Supplemental File 4: Themes by country and additional participant quotes

BENEFITS OF RISK CALCULATOR

Themes and subthemes	Brazil	Nepal	Nigeria	United Kingdom
**BENEFICIAL**				
Increase help-seeking among adolescents	9 adolescents	8 adolescents		
Motivate adolescents to engage in more self-care	1 adolescent	8 adolescents		
Lead to reduced suffering in society			2 social workers, 2 teachers	
Reduce future risk of depression		38 adult stakeholders	20 adult stakeholders	
Improve services (promote service collaboration, raise awareness, and lead to more uniformity in risk assessment)				9 healthcare practitioners, 8 school counselors, 4 policymakers

"People that have low risk will feel good and maybe are going to try helping others. And, people with high risk maybe will take care of themselves better." (Depressed adolescent, Brazil, BR-4)

"I think [the risk calculator] is good. [The risk calculator] is enough to know if the person has or will have depression." (Depressed adolescent, Brazil, BR-9)

"It is a faster, more practical, individual way. It is a personal answer for people to identify and become aware of themselves and seek help, which in general does not happen in its own. I think it will give the person an idea because it will identify the person: ah, I have these options, I can get help…" (School principal, Brazil, BR-12)

"You'll get to know if you are in [high risk] or not. [Then] get help on what we should do, and get more help on preventing it… Like, what can we do in this stage, whom to share the information with, and how can we be protected?" (Depressed adolescent, Nepal, NP-39)

"We hear about people committing suicide due to depression. If they see this, go through the link, take counseling, they might come out of depression. So, I find it helpful." (Non-depressed adolescent, Nepal, NP-34)

"If I find out I have high risk of depression, I will be more careful. I will try to self-motivate. I will try to avoid tension. I will try to build a positive attitude… I will share with others… If others don't have suggestions for me on how to prevent depression, I will search for information on the internet. No matter what, we need to have a positive attitude." (Non-depressed adolescent, Nepal, NP-35)

"Eh, [we need a] sane society, a progressive society. Because the people we talking about now mainly are the future of tomorrow. The sanity of this nation tomorrow depends on them. If you start planning for them now at this stage, you're preparing them for a better future." (Social worker, Nigeria, NG-02)

"So, with this questionnaire we can actually go—this questionnaire can actually go—a long way in reducing the number of people who will think of suicide and then you know." (Teacher, Nigeria, NG-19)

"I always appreciated when I did get information about students' home lives, and I knew what was going on. That made my life a lot easier…So, in that sense, it would be really useful to have something that, you know, was evidenced-based, and you didn't have to do your own kind of qualitative interpretation of the data." (Social worker, UK, UK-39)

UNDERSTANDABLE

Themes and subthemes	Brazil	Nepal	Nigeria	United Kingdom
**UNDERSTANDABLE**				
More awareness about mental health among young generation			1 policymaker	1 social worker, 10 healthcare practitioners, 6 school counselors, 6 policymakers
Need to raise awareness that depression is treatable	6 healthcare practitioners	3 adolescents		12 healthcare practitioners, 8 school counselors, 5 policymakers

Themes and subthemes	Brazil	Nepal	Nigeria	United Kingdom
Need for education about depression for adult stakeholders, including parents, teachers, and healthcare workers		2 adult stakeholders	5 adult stakeholders	5 healthcare practitioners, 5 school counselors, 3 policymakers, 8 parents
Misunderstanding that the risk calculator is intended to be used to detect current depression	8 adolescents, 3 adult stakeholders	3 adolescents	15 adult stakeholders	1 healthcare practitioner, 1 social worker
Difficulty with understanding probability and need for lay language to explain risk	1 psychiatrist	3 adolescents, 1 healthcare practitioner	6 adult stakeholders	8 parents, 6 healthcare practitioners, 8 social workers, 2 policymakers
Local data on risk is needed before use so that risk numbers are calibrated to the local context		4 policymakers, 1 healthcare practitioner		6 healthcare practitioners, 4 school counselors, 4 policymakers
Need for tool to be available in diverse languages and suitable for use with low literacy populations		3 adult stakeholders	4 adult stakeholders	
Healthcare practitioners, teachers, and counselors could be trained to administer and explain the tool in a way that would not be distressing	11 adolescents, 27 adult stakeholders	11 adults	1 social worker	12 healthcare practitioners, 10 school counselors, 4 policymakers

Need to assure mental health literacy for depression before administering risk calculator

“[Using the risk calculator] depends upon the choice of words. When [a result is provided as] high risk, content like information that depression can be cured should be included, rather than just on normal things like nutrition and help-seeking. Otherwise, if there isn’t information about treating depression, the fear will be there. We are scared to even look at the results when we take normal astrology [horoscope]… While giving links [to information on depression], there should be one for help, another link to understand about depression… Rather than trying to write it all there, put the link there, and they can also visit different websites.” (Depressed adolescent, Nepal, NP-43)

"If only the parents are told about it, then how will they handle their children is important. It is not necessary that all the parents are able to understand this? We all know that there are very few parents in Nepal who sufficiently understand these things. That is why we need to inform the parents, as well the students, about what [depression] is." (School nurse, Nepal, NP-026)

“…You want this thing to end, but the truth is that let’s look at the consequences on your family members, on your loved ones, on these and that and at the end,… [If they understand the consequences of depression on their family], then we can offer a solution… and they will take it. You understand it’s just like a pregnant woman who walks into a facility, and you tell the woman she has high blood pressure, this can affect you and your baby or you may have an ectopic pregnancy. You and your baby are at risk, what do you want us to do? Provided the person has seen the result of the test, the next thing they want is the way forward. So, it will help.” (Social Worker, Nigeria, NG-05)

“And one of the things I find so refreshing about my kids is how openly they talk about [depression]. I do, you know, compared to when I was that age, you know, you wouldn’t speak, it was just a taboo topic, mental health. So, I do think we’re in so much of a better place today than we were then.” (Healthcare worker, UK, UK-77)

“But most parents probably wouldn’t have that knowledge, or you know, even know what to do with it if they did recognize [depression].” (Parent, UK, UK-19)

“[We need] something that kind of gets parents on board, erm, in a way that doesn’t get a lot of knee jerk resistance to it? Kind of like vaccinations have developed… And in a way that just makes it a part of looking after your health, just a sort of part of your preventative care, you know? Get your five a day, get your mental health screening, you know?” (Parent, UK, UK-82)

Need for education to understand difference between having a disease compared to having risk for a disease

"First of all, they will become anxious. The children will get scared that they are at high risk fearing that something bad will happen to them. Rather than just giving them the results, we need to explain what the disease is and what the possibility of getting it is. We need to speak positively with them and tell them that there is a chance that they will not suffer from it. When talking to the children, we should go from simpler to complex. That is how we should explain to the adolescents. We need to reassure them. We need to tell them that they are not going to lose their lives if they have it like other physical illnesses. They can take counselling and medication for it. This does not mean that they will have this disease for sure but they are only at risk. We should tell them about the things that they need to avoid to reduce their chances of getting it. If we are able to counsel them as such, then they will be able to relate it a little." (School nurse, Nepal, NP-26)

“The negative aspect is that they get scared of the risks. They will get more depressed thinking [because they were told] they have risk of depression.” (Non-depressed adolescent, Nepal, NP-35)

"If someone is said to be in risk now, you have to look for them after 5 years or 6 years and you have to do it with a lot of people. Well, that will be research and after that knowing how valid and reliable it is, then after that maybe you can train people. " (School counselor, Nepal, NP-10)

“Well, I think that is where we may want to bolster the information a bit more. That the fact that you have the high risk does not mean that you must develop depression. And that the reason why we are having this is so that we can be able to provide you some guidance on how to avoid depression. And most people who answer many of these questions are at risk of depression, but that [doesn’t mean] everybody will develop depression. So, this is just—just being able to provide more information so that it won’t be distressing. And, of course, the low risk [adolescents] also need to be aware that the fact that you are low risk, that there are people who have been low risk and their risk of depression could just skyrocket with just one single life event. So, at the end of the day, anybody will still be provided with information that can build their resilience.” (Healthcare worker, Nigeria, NG-35)

“Well, same as any screening tool. You know, you get false positives, you have unnecessary morbidity. You have the pathologization of somebody who doesn’t need to be pathologized, particularly at the age and state that they’re at. It’s better to have a narrative which is just about life circumstance than there’s something wrong with you.” (Healthcare worker, UK, UK-21)

“But sort of, or that they take it on, they adapt it as a label and they’re telling everybody that they are depressed, and then letting that dictate what then happens from then on, that they’re going to sort of make it a self-fulfilling prophecy.” (Social worker, UK, UK-51)

“Maybe some stats on how common [depression] is. So that they know it’s not just them. It’s good to know: okay, 1 in 2 people, 1 in 3 people suffer from depression, or children with these stats go on to develop depression, kind of. So that they know that it’s not abnormal. You know, it’s just, yeah. You can have depression, that kind of thing, just to get rid of that stigma.” (Parent, UK, UK-29)

Need for administration with adolescents with low literacy

“You might have an adolescent who cannot read it, you might have an adolescent who does not have access to a phone, so in such a situation you tend to want someone who is counselling or someone who is doing the psychotherapy to administer it to him… It could be oral, you can read it to them verbally.” (Social worker, Nigeria, NG-49)

Recommendation for a trained person to administer a risk prediction tool

“I think it will depend on how this interview gets to him. I think if she, if she has information before… Because, of course, we know that there is a difference between you answering an interview without … having nothing yet, you know, no pre-information, and when you have. But, maybe, if he understands why this is being done and he has that understanding, he will want—'Ah, I want to know’. Right, because he may be questioning himself, who knows, just thinking about it, then I will say that he has a high risk, okay, so maybe I have to take care of myself, I have to be alert.” (Teacher, Brazil, BR-09)

“I think that in the case of my students, if they used [the risk calculator], it would generate a lot of, I don’t know, restlessness. The question is that they already have a broken bond with their family, so, in this case, I think it would be very triggering, they would have to deal with a lot. There would be a lot of questions, because they are very related to things they have lived.” – (Social worker, Brazil, PAS-5)

"I think that... That teachers should show it so the adolescent himself would be able to want to know more, to understand and do the calculator, this calculator." (Depressed adolescent, Brazil, US-5)

"That was why I did say that people who will administer this questionnaire should also be trained too. And then that training should be inclusive of what to say and what not to say. So, if an individual has adequate information about a particular illness, you know that person can become an expert patient. So, that she'll know what to do and what not to do and where to seek for help, whether to seek for immediate help, or delay it and still watch out. And then a little bit of talk therapy can also douse the tension, can also reassure the individual that is at high risk to take things gently and all that. Information is powerful so when an individual has enough information to work on, the issue of self-stigma will not be anything to... I mean that would pose a serious concern. (Social Worker, Nigeria, NG-07)

"GPs [general practitioners] and medical professionals, especially GPs, they... can neutralize the stigma of mental health. Just because they're the sort of like the go-to authority for all medical conditions, so if you've got your GP basically saying this is a condition, this can be treated, this is how it's treated, that can really soften any kind of preconceptions that people can come to with, "oh it's just a bad attitude, and you can just...decide to not be like this". They, obviously, they're very helpful because...they have sort of a clear path of what next? Because they're exactly the people that you'd be wanting to go to for your sort of first steps of advice and being able to check in as things went on." – (Parent, UK, UK-82)

CONFIDENTIAL

Themes and subthemes	Brazil	Nepal	Nigeria	United Kingdom
CONFIDENTIAL				
Adolescents provide assent and guardians provide consent		2 adolescents, 3 healthcare practitioners, 3 social workers	3 adult stakeholders	
Only the parent/guardian is responsible for consent			9 adult stakeholders	1 parent said require parental consent.
Schools can inform parents through integration into general health screenings held at schools			1 policymaker	

Themes and subthemes	Brazil	Nepal	Nigeria	United Kingdom
It is acceptable to inform parents about the risk calculator, but their permission is not needed for an adolescent to complete it; an opt-out procedure can be used		7 adults		12 healthcare practitioners, 8 school counselors, 3 policymakers
All responses are kept confidential	7 adolescents	2 adolescents; 15 adults	3 adult stakeholders;	10 healthcare workers, 7 school counselors, 4 policymakers, 9 parents
Adolescents might falsely inflate their risk exposers to get more attention from teachers and family	1 social worker	4 adolescents		
Adolescents can self-administer the risk calculator		6 adult stakeholders	7 adult stakeholders	10 healthcare practitioners, 7 school counselors, 4 policymakers
Adolescents should not complete the risk calculator, instead, it should be completed by teachers, religious leaders, or other adults supporting the adolescents		7 adult stakeholders	30 adult stakeholders	
Parents are unlikely to answer honestly because of questions about the relationships with parents and the questions related to maltreatment		1 social worker	1 policymaker	8 healthcare practitioners, 11 school counselors, 11 parents, 3 policymakers
Issues of maltreatment need to be reported		1 adolescent, 2 adult stakeholders	20 adult stakeholders	
Telling adolescents that they are high risk will predispose them to getting depressed; it is a 'self-fulfilling' prophecy	1 psychiatrist	3 adolescents	6 adult stakeholders	11 healthcare practitioners, 9 school counselors, 4 policymakers
Adolescents should not have access to the results, instead, give the information to a counselor or trusted adult who can help the adolescent engage in preventive behaviors		7 adult stakeholders; 7 adolescents	20 adult stakeholders	

Themes and subthemes	Brazil	Nepal	Nigeria	United Kingdom
Teachers and parents should receive the results so they can treat the adolescents better			19 adult stakeholders	
Parents would think more negatively about their children if they found out that their child was high risk		3 adult stakeholders		
Teachers would treat the adolescent negatively if they knew the adolescent was high risk			1 adult stakeholder	
Policymakers and officials having access to aggregate information to identify schools or regions with high rates of high-risk youth		11 adult stakeholders	17 adult stakeholders	
Health insurance companies, employers, and educations should have access to the information		14 adult stakeholders	9 adult stakeholders	21 adult stakeholders

Confidentiality when using a prediction tool

"Because [the risk calculator] has facts that happen to most of the adolescents, and they probably don't tell anyone. But if it has confidentiality, if you tell them that you are not going to tell anyone, the person is going to trust you and probably will answer everything with the truth. They won't lie anywhere, won't deny anything, because they will be trusting this." (Depressed adolescent, Brazil, BR-22)

"It depends upon the individuals, I guess. Some people—they just want to keep their information to themselves. But there are some people who are actually sharing with other people too. So, both [free disclosure or withholding information] are possibilities." (Depressed adolescent, Nepal, NP-37)

"It should not be a self-administered questionnaire that means the adolescent cannot administer by themselves. So it should be health worker, or teacher, counsellor or anybody that has been trained to identify, should be administered by those people." (Teacher, Nigeria, NG-16)

"Yes, because of the stigma associated with depression. And, of course, individual differences are there, so you'll have some children that might want to bully or make jest of that depressed child. It'll might have a negative impact. But the responsibility of the teacher or the health worker is to make the child see that you need help, and it is being appreciated that you've come out to ask for it." (Healthcare Worker, Nigeria, NG-28)

Who would provide consent for adolescents completing a prediction tool

"About adolescents below 18 years, information should be shared with parents. If above 18, then it is better to let them know by themselves as we assume they are legally matured." (Social worker, Nepal, NP-37)

“The legal aspect. Especially for an adolescent, one that is below 18 years old, the consent of the parents is needed. That's one thing that you do see. There are two ways that they can—they can work with the schools so that it is a part of the package that the parents sign the form. I know that in my daughter's school, there's health day when they do screenings for eyes, the dentist comes, and so on and so forth. So, I basically approved them, they give me forms to approve such. So, you can work with the schools so that you get consent of the parents.” (Policy Maker, Nigeria, NG-39)

“…making sure parents are onboard. I think that’s the crucial thing because if you don’t communicate with parents constantly, they’re going to get, they’re going to be far less happy and actually, probably, because I think a lot of them would be really pleased that this measure is being put in place.” (School worker, UK, UK-85)

Recommendation to share results with adult stakeholders

"According to their results, they need to sit down with their guardians and talk to them. It means that their results have to be disclosed in a minor form first. And then we should disclose the major implications when their guardian is with them. The adolescents will also feel that they need to tell their guardians now. They will feel that it is a decision that they have taken themselves." (Healthcare worker, Nepal, NP-27)

“It depends on the kind of training the teachers have had. If they've been trained on what to look out for, how to seek care and what to do, it can be helpful. But if they haven't, they would either panic and look for the first way out to isolate the child, or they may seek professionals to come and assist them or something like that.” (Policymaker, Nigeria, NG-37)

“Such an adolescent will be given special treatment by the teacher. Because such a teacher knowing that this student has high risk of depression would not want such a child to go into depression. So would be treated specially compared to other child.” (Teacher, Nigeria, NG-18)

“Because I guess, in a way, they need to be involved—the parents and the other professionals—in kind of the young person’s recovery and treatment as well, perhaps for it to work. They need—at some level—to be supportive as well. And quite often, a lot of the work we do involves parents and other agencies—we try and use other agencies to kind of support—in all areas of the child’s life.” (Healthcare worker, UK, UK-91)

Do not share results with adolescents

“I see more of us caregivers using it than teenagers. Because then you already know the story, you already can ... and you will access it, you will have a sense, right, of what will be the result. But, then [the adolescent] will not have much contact with it. I even think that it would not be good for teenagers to access it, too, it would be something more for adults because ... sometimes he will end up believing that it is something that is not, and create paranoia, something.” (Social worker, Brazil, BR-35)

“It will be good [to share with parents]. If we share with [an adolescent], she will have tension. If shared with guardians, they will try making their child feel good. Their child will feel that people care about them. A kind of positive attitude is developed. If shared with others [and not the adolescent], she won’t know that she is likely to have depression and then she will not develop tension. Others will show her love knowing this and try to care. Due to this, the negative feelings clear out. (Non-depressed adolescent, Nepal, NP-35)

“Doesn’t [a high-risk result] seem like an excuse for the person? Maybe I will be backing out from my job or stop studying and blame my condition and say that it is because I am at risk of depression?... We have to counsel them from a different approach and tell them that it is not a problem and instead approach their parents and tell them that their children are at high risk of depression.” (Depressed adolescent, Nepal, NP-11)

"We might also get some cases of high risk of depression where we could look from the parent’s or teacher’s perceptive and know if they are at risk, because obviously very high risk of depression could be seen from other’s view as well. And for the other general population, teachers or nurses or other person could use this tool up on them and know the result, but don’t reveal it to the adolescent. And, start the intervention where needed, and instead [of giving the actual results to an adolescent] provide generic feedback or messages to everyone so as to make them aware and prevent depression." (Psychologist, Nepal, NP-08)

“I think when it gets to stage when a child is feeling that form, I think, I wanted to say this before that, erm, it’ll be better for the child not to know if he or she is at high risk or low risk. Let it be known to the person attending to the child. So, the person attending to the child will know how to—and because sometimes if they know, it might trigger some other things, you understand. So, that’s why it is even better sometime for them not to—fine they may be filling the form, but when it comes to that aspect where it’s going to calculate if they are at high risk or low risk, it should be best known the person that is attending to them. Because normally children they are very sensitive, it is what they see, they react to, it is what they hear, they react on. So, I believe keeping it away from them or the society will really help a lot.” (Social Worker, Nigeria, NG-06)

“I don’t know whether it might push you down that, I don’t know if it’s self-fulfilling prophecy, well, that’s what I’ve been told, it’s going to happen, so that’s kind of the path that you end up going down.” (School worker, UK, UK-12)

Do not share results with adult stakeholders

"Parents may also be anxious and may get over involved in their children's life because of fear; they may not allow their children to go out because of fear. But for this, we have to provide them proper information." (Psychologist, Nepal, NP-05)

"...In communities like ours people generally have negative attitudes towards mental health problems or mental illness. Therefore, I don't think this needs to be revealed to others. Like, if teachers know about this then he could say this person is like this and that. If parents and guardians come to know then they will also start to say this and that. This also can happen in the community. Therefore, this should be done from individual level... I think we should maintain the privacy." (Healthcare worker, Nepal, NP-04)

"Now the downside is that stigma might even start without the child becoming depressed. Then if it's a child that enjoy playing the sick role, they'll just fall into it. Everybody will pamper them. Do you understand, ...so I think that is that." (Healthcare worker, Nigeria, NG-27)

Do not share results with insurance companies

"I think it will affect people's insurance policies if people knew about it. If you know about it yourself, you'd have to—in theory, you'd have to—reveal it on forms. So, it could be quite compromising, really." (Healthcare worker, UK, UK-021)

Need for training to assure confidentiality

"The [risk calculator] would be delivered in schools. And it would be necessary to train the ones who apply it. Because if we realize the adolescent is being a victim to abuse, we already had situations like this, so situations like abuse, and then anything that happens with the adolescent is because of the school. If the school, even if not on purpose, leaks the information, then other students talk, then the situation gets worse, right? So, we have to deal with this, with good training." (Social worker, Brazil, BR-12)

Share results in aggregated format with policy makers

"Everyone will get the information and it is also useful later to make policies and programs in ideal budget." (Policymaker, Nepal, NP-47)

"Like after getting all the data, put it together, take the statistics, how the—the rate if it is high. There are processes. Follow the due process going to—maybe the leader in the community first, to the government, to let them know this is what is at hand. This is the stage adolescent depression is in Nigeria. And, we have some solutions. Because we cannot state problems without providing solutions. Tell the government then." (Teacher, Nigeria, NG-23)

ACTIONABLE

Themes and subthemes	Brazil	Nepal	Nigeria	United Kingdom
**ACTIONABLE**				
For risk calculators to be used, the results had to be actionable; youth, families, schools, health workers, policy makers, and others need to be able to do something about the prediction information	37 adult stakeholders	5 adolescents 27 adults		8 healthcare practitioners, 8 school counselors, 6 parents, 5 policymakers
If an adolescent or parent were told of high risk, but not provided with resources for prevention then this was not helpful and potentially more harmful than not knowing the risk	37 adult stakeholders	5 adult stakeholders		1 healthcare practitioner
It is not only knowing the risk but rather having evidence-based practices to feel in control, so that adolescents don't give up	6 adult stakeholders	1 adolescent, 4 social workers		
Need for multi-level information on prevention: what can the individual do on their own, what can the family do, what can educational institutions do, and at the systems level, what can policy makers do		18 adult stakeholders		
If an adolescent had high risk, they could just go to a counselor or mental health professional who would tell them how to prevent depression		5 adult stakeholders, 6 adolescents		
There are no evidence-based prevention services in their settings	1 policymaker, 1 psychiatrist, 8 healthcare practitioners	7 healthcare practitioners, 5 policymakers	3 adult stakeholders	8 healthcare practitioners, 8 school counselors, 5 policymakers
Adolescents would look online in YouTube and social media and get directed to prevention strategies that were not evidence-based	3 adolescents; 5 adult stakeholders	2 adolescents; 15 adults	8 adult stakeholders;	2 healthcare workers, 7 school counselors, 1 parents

Themes and subthemes	Brazil	Nepal	Nigeria	United Kingdom
Religious institutions could provide support for high-risk adolescents			3 adult stakeholders	1 social worker
Even if preventive services were available, adolescents would not use them		8 adult stakeholders		
Any prevention program needs to be made available throughout the country		2 policymakers	14 adult stakeholders	8 healthcare practitioners, 7 school counselors, 4 policymakers
Developing preventive programs would take away from the resources provided for treatment	1 parent; 8 healthcare practitioners; 3 policymakers	16 adult stakeholders	8 adult stakeholders	9 healthcare practitioners, 11 school counselors, 5 policymakers, 9 parents, 7 social workers
Austerity measures make it unfeasible to develop preventive services in the healthcare system, and in the education system, teachers are already too overburdened to provide preventive services				11 healthcare workers, 11 school counselors, 9 social workers, 6 policymakers, 6 parents,

Prediction tool needs to be accompanied by prevention services

“What happens is the following. So, I have my—my diagnosis of high risk for depression. But, then it is a lot about the luck of this adolescent, that he will look for another resource and not the original resource [where he answered the risk calculator]. It would be better to look for [help] and say, like, “Look, I have already answered this and then it said I have high risk.” ...And then it goes to somewhere and then goes to another one, they end up on a waitlist. It could even be dangerous, because the person is already in a high risk of depression.” (Teacher, Brazil, BR-10)

"We have to provide them the service if needed, as this is in our ethics as well." (Parent of depressed adolescent, Nepal, NP-001)

"We can reveal results immediately to those who have access to services near them... Just imagine, if someone tells you that you are at a high risk of depression then what would be your reaction? How would you feel? You would obviously feel stressed, but those who have access to services could be calmed down and might not be a victim of negative consequences." (Psychologist, Nepal, NP-008)

“Hmm, maybe I’d want to know about some consultants. And, the thing is, who is available to help you? There has to be someone that you can ask more about this. Different people have different confusions about everything, so maybe there has to be someone—a different contact—to ask more about it. (Non-depressed adolescent, Nepal, NP-18)

“…at first, when you showed it to me, I thought, oh, like, yeah, it’s not very complicated to answer. But then, what do you do, what is the next step, like, when you have the, ok you are in high risk, what do you do with that information.” (Parent, UK, UK-97)

“But then still, even if you’re aware of it…you still need somewhere to go with it, don’t you? And if there’s nowhere to go with it, then it doesn’t help much…” (Parent, UK, UK-19)

“I think some accurate literature about what that means that’s simple, to the point, you know, no jargon and all that, but just in layman’s terms would be helpful to accompany something like that to give to parents and alleviate some of their fears around that. So that if they were to come out high risk, that there is support that is available, and it’s not just for assessing as high risk and then off you go, but that actually, it’s about intervening early and getting that support in place to avoid them getting to a crisis point.” (School worker, UK, UK-12)

“I believe that it would only be ethical really to roll it out to people if it then would trigger some support. Because, I think it’s really, erm, dangerous to get into a situation where you’ve got a young person saying this happened to me and effectively, kind of, not being listened to and actioned.” (Parent, UK, UK-68)

“And I’m really concerned that, you know, in the current context of, you know, rising social inequality, for, you know, poverty affecting huge numbers of children, you know the kind of instrumentalization of schools and the kind of transactional nature of school culture, you know, reduction in public health services and mental health services for young people, I would be really concerned that simply highlighting risk of depression without providing students with a means to mitigate that risk, you know, and wider stakeholders to mitigate that risk, it raises probably more problems than it solves, I think.” (School worker, UK, UK-044)

Assumption that mental health professionals can provide services for at-risk adolescents

"By using such a tool, they could be able to refer themselves to the service centers. Even the government has planned to keep one nurse as a school counsellor in each school, where adolescents could go and talk about their risk of having depression once they know about it after using this tool. So, these are the benefits." (Healthcare worker, Nepal, NP-008)

"If we conduct the assessment using the questionnaires that you have prepared, then we will offer them counselling if we learn that they are at risk. They will become aware if we let them know that they are at risk. They will be aware about the things that they should not do after they learn that they are at risk. If we are able to make even a single client who is at the risk of depression aware, then it is a big thing because we will be preventing the loss of a life." (School nurse, Nepal, NP-026)

"We can reveal results immediately to those who have access to services near them. For example, if someone is in high risk and they have school counsellor in their school then we can tell them that they are at high risk of depression so that they could immediately go to school counsellor for counselling if needed. But, where people don't have access to such facilities and yet know the result, they might get scared and might have negative consequences in them. So, for them, we can provide some generic information about mentally healthy life style and preventive measures of depression instead of directly showing them their result of risk." (Psychologist, Nepal, NP-008)

Recommendation for self-help online resources

"Psychological coping is being so much simplified nowadays that it is not necessary to visit health workers all the time. My point is people might not come. In several settings, self-help packages are available. ...There is a need for developing online-based self-help materials, which might be YouTube videos, chat-bot, and so on. The discussion is on a process to include issues like self-help in evolving digital—targeting urban, and peri-urban adolescents. So, in this, children identified the high-risk on their own and the link for self-help can be there..." (Policy maker, Nepal, NP-049)

Recommend structural changes for prevention

"We can't change the person, you know—if you're changing the personal level, then you're never going to make sustained changes. So, we go in and we sort of change the individual family, well, we do some work with the individual family, and then we go away, and then it comes back. That's not what needs to change. We need to be changing at the structural level." (Social worker, UK, UK-054)

No prevention resources available

"If it is online, only the ones who actually look for it are going to find it. The people that won't look for it are probably the ones who are at risk for developing depression. They won't find it. And in schools, like, if it depends on some governmental policy, it's really hard to be implemented, because there are many schools that don't even have money to print it." (Depressed adolescent, Brazil, BR-8)

"At the same time, it is a matter of practical reality that at the moment, at least in Brazil, but I believe that in the world, we do not have—there is a great lack of mental health professionals, let alone in our country, so we barely and barely manage to identify cases already in depression, already with the syndrome, and make the appropriate treatment. There is a lack of medication, a lack of psychotherapy, in droves, and a lack of case identification, a lack of looking at the cases we already have. So, it is very difficult for us to achieve. Of course, prevention is still important because it possibly reduces this overload. But at the same time, in terms of Brazil, it is very difficult for us to imagine yet." (Parent, Brazil, US-9)

"I think, I think my answer is not very encouraging, but I will tell you the truth, which I imagine is what you want from me. I think it is completely unrealistic in the public health system." (Psychiatrist, Brazil, BR-52)

"Let's say the risk is identified. The person maybe in high risk, medium risk, or low risk. After knowing that, where will the person go and find the service? We talked so much about how there is no service available for depression. That's why what we say in public health: if you don't have the solution, you should not see the problem. " (Policymaker, Nepal, NP-52)

"...And the fact that there is no expertise in this country is out of our hand. So, the problem is figured out but we don't know what should be done next because it's not within our control. It is easy find out the problems. It will be hard to find the solutions." (Social worker, Nepal, NP-41)

"Our expectation is that the people will come if the service is available, which I think is almost false. Nevertheless, the point is that at least the service should be there. Our current focus is one who comes should get services. We are needed to do that as we do not have services now. Our challenges will be when people do not come despite services being available." (Policymaker, Nepal, NP-49)

"We ask them to go there, that is very good considering that they are not taking service as they were not known. But even if we let them know, they go there, and if the service is available or not, how is the charge, time, opening time there. Think about the privacy, how assured they can be? Considering all this, the matter is if they will go there or not." (Social worker, Nepal, NP-042)

"I think schools are completely over-worked and overwhelmed and are taking on more and more of a therapeutic role, and resources need to go into that." (Social worker, UK, UK-01)

"It's not like we've got some CAMHS provision for people that are in the early stages of stuff. That's not there. So why on earth, if I were a GP, would I want to screen for stuff because I can't even get the people with severe depression and significant self-harming a CAMHS appointment? So, I mean, I would just be completely uninterested in primary care about this, ...because I'd just think, well it's extra work for no benefit for me or the patient." (Healthcare worker, UK, UK-21)

"I mean, obviously, it should be, I would say, local community centres, after school activities, and CAMHS. But, I just don't think any of them are, at the moment, have got enough anything to do, to add something extra." (School worker, UK, UK-19)

"...the NHS is very good at keeping people alive, it's not very good at keeping them well, or preventing them from getting unwell. You know, it's always a firefight response. So, you know, when the young person comes to A&E [Accident and Emergency], having self-harmed, or is suicidal—everyone pays attention. Anything further, anything that precedes that, anything that is downstream is less of an interest." (Healthcare worker, UK, UK-24)
